# MScanner: a classifier for retrieving Medline citations

**DOI:** 10.1186/1471-2105-9-108

**Published:** 2008-02-19

**Authors:** Graham L Poulter, Daniel L Rubin, Russ B Altman, Cathal Seoighe

**Affiliations:** 1 UCT NBN Node, Department of Molecular and Cell Biology, University of Cape Town, Cape Town, South Africa; 2 Stanford Medical Informatics, Stanford University, San Francisco, USA; 3 Department of Bioengineering and Department of Genetics, Stanford University, San Francisco, USA

## Abstract

**Background:**

Keyword searching through PubMed and other systems is the standard means of retrieving information from Medline. However, ad-hoc retrieval systems do not meet all of the needs of databases that curate information from literature, or of text miners developing a corpus on a topic that has many terms indicative of relevance. Several databases have developed supervised learning methods that operate on a filtered subset of Medline, to classify Medline records so that fewer articles have to be manually reviewed for relevance. A few studies have considered generalisation of Medline classification to operate on the entire Medline database in a non-domain-specific manner, but existing applications lack speed, available implementations, or a means to measure performance in new domains.

**Results:**

MScanner is an implementation of a Bayesian classifier that provides a simple web interface for submitting a corpus of relevant training examples in the form of PubMed IDs and returning results ranked by decreasing probability of relevance. For maximum speed it uses the Medical Subject Headings (MeSH) and journal of publication as a concise document representation, and takes roughly 90 seconds to return results against the 16 million records in Medline. The web interface provides interactive exploration of the results, and cross validated performance evaluation on the relevant input against a random subset of Medline. We describe the classifier implementation, cross validate it on three domain-specific topics, and compare its performance to that of an expert PubMed query for a complex topic. In cross validation on the three sample topics against 100,000 random articles, the classifier achieved excellent separation of relevant and irrelevant article score distributions, ROC areas between 0.97 and 0.99, and averaged precision between 0.69 and 0.92.

**Conclusion:**

MScanner is an effective non-domain-specific classifier that operates on the entire Medline database, and is suited to retrieving topics for which many features may indicate relevance. Its web interface simplifies the task of classifying Medline citations, compared to building a pre-filter and classifier specific to the topic. The data sets and open source code used to obtain the results in this paper are available on-line and as supplementary material, and the web interface may be accessed at .

## Background

### Ad-hoc information retrieval

Information retrieval on the biomedical literature indexed by Medline [1] is most often carried out using ad-hoc retrieval. The PubMed [2] boolean search engine is the most widely used Medline retrieval system. Other interfaces to searching Medline include relevance ranking systems such as Relemed [3] and systems such as EBIMed [4] that perform information extraction and clustering on results. Certain web search engines such as Google Scholar [5] also index much of the same literature as Medline. Alternatives to ordinary queries include the related articles feature of PubMed [6], which returns the Medline records most similar to a given record of interest, and the eTBlast [7] search engine which ranks Medline abstracts by their similarity to a given paragraph of text.

### Supervised learning for database curation

Ad-hoc retrieval in general has proven inefficient for the task of identifying articles relevant to databases that require manual curation of entries from biomedical literature, such as the Pharmacogenetics Knowledgebase (PharmGKB) [8], and for constructing corpora for automated text mining systems such as Textpresso [9,10]. It is difficult to design an expert boolean query (the knowledge engineering approach to document classification [11]) that recalls most of the relevant documents without retrieving many irrelevant documents at the same time, when there are many document features that potentially indicate relevance.

The case of many relevant features is, however, effectively handled using supervised learning, in which a text classifier is inductively trained from labelled examples [12,13]. Several databases have therefore used supervised learning to filter Medline for relevant documents [14], a recent example being the Immune Epitope Database (IEDB) [15]. IEDB researchers first used a sensitive PubMed query several pages in length to obtain a Medline subset of 20,910 records. The components of the query had previously been used by IEDB curators, whose manual relevance judgements formed a "gold standard" training corpus of 5,712 relevant and 15,198 irrelevant documents. Different classifier algorithms and document representations were evaluated under cross validation, and their performance compared using the area under the Receiver Operating Characteristic (ROC) curve [16]. The best of the trained classifiers is to be applied to future results of the sensitive query to reduce the number of documents that have to be manually reviewed.

Supervised learning has also been used to identify Medline records relevant to the Biomolecular Interaction Network Database [17], the ACP Journal Club for evidence based medicine [18], the Textpresso resource [9], and the Database of Interacting Proteins (DIP) [19]. Classification may also be performed on full-text articles as in the TREC 2005 Genomics Track [20], and Cohen [21] provides a general-purpose classifier for the task. Most classifiers have been developed for filtering sets of a few thousand Medline records, but it is possible to classify larger subsets of Medline and even the whole Medline database. A small number of methods have been developed for larger data sets, including an ad-hoc scoring method that has been tested on a stem cell subset of Medline [22], the PharmGKB curation filter [23], and the PubFinder [24] web application derived from the DIP curation filter [19]. However, tasks submitted to the PubFinder site in mid-2006 are still processing and the maintainers are unreachable. In some cases, text mining for relationships between named entities is used instead of supervised learning to judge relevance – for example in the more recent curation filter developed for the DIP [25]. The most closely related articles [6] to individual articles in a collection have also been used to update a bibliography [26] or a database [27].

### Comparison of information retrieval approaches

Approaches to retrieving relevant Medline records for database curation have included ad-hoc retrieval (boolean retrieval in particular), related article search, and supervised learning. Pure boolean retrieval systems like PubMed return (without ranking) all documents that satisfy the logical conditions specified in the query. The vector space models used by web search engines rank documents by similarity to the query, and probabilistic retrieval models rank documents by decreasing probability of relevance to the topics in the query [28]. Related article search retrieves documents by their similarity to a query document, which can be accomplished by using the document as a query string in a ranking ad-hoc retrieval system tuned for long queries [6,29]. Overlap in citation lists has also been used as a benchmark for relatedness [29]. The method used in PubMed related articles [6] directly evaluates the similarity between a pair of documents over all topics (corresponding to vocabulary terms) using a probabilistic model. Supervised learning trains a document classifier from labelled examples, framing the problem of Medline retrieval as a problem of classifying documents into the categories of "relevant" and "irrelevant". Classifiers may either produce ranked outputs or make hard judgements like a boolean query [12]. Statistical classifiers, such as the Naïve Bayes classifier used here, use the same Probability Ranking Principle as probabilistic ad-hoc retrieval systems [28]. Ranked classifier results may loosely be considered to contain documents closely related to the relevant examples as a whole.

### Overview of MScanner

We have developed MScanner, a classifier of Medline records that uses supervised learning to identify relevant records in a non-domain-specific manner. The user provides only relevant citations as training examples, with the rest of Medline approximating the irrelevant examples for training purposes. Most classifiers are developed for particular databases, a limitation that we address by demonstrating effectiveness in multiple domains and providing facilities to evaluate the classifier on new inputs. We make it easier to use text classification by providing a web interface and operating on all of Medline instead of a Medline subset. To attain the high speeds necessary for online use, we used an optimised implementation of a Naïve Bayes classifier, and a compact document representation derived from two feature spaces in the Medline record metadata, namely the Medical Subject Headings (MeSH) and the journal of publication (ISSN). The choice of the MeSH feature space is informed by a previous study [23], in which classification using MeSH features performed well on PharmGKB citations. We describe the use of the classifier, present example cross validation results, and evaluate the classifier on a gold standard data set derived from an expert PubMed query.

## Results

### Web interface workflow

**Figure 1:**
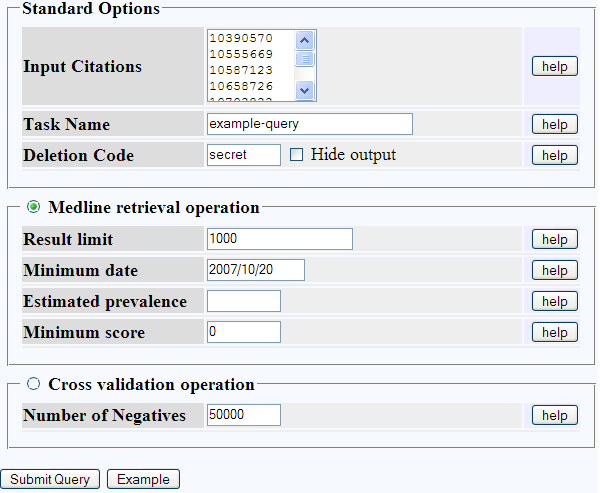
**Web interface**. Submission form for Medline retrieval and cross validation. Relevant training examples are provided as a list of PubMed IDs.

The web interface, shown in Figure 1, takes as input a list of PubMed IDs representing the relevant training examples. In the case of a database curated from published literature, the PubMed IDs can be extracted from line-of-evidence annotations in the database itself. An existing domain-specific text mining corpus or bibliography may also serve as input. The classifier is then trained, in order to calculate support scores for each distinct term in the feature space (see Methods and Table 1). It uses the input corpus to estimate term frequencies in relevant articles, and the remainder of Medline to estimate term frequencies in irrelevant articles. The remainder of Medline provides a reasonable approximation of the term frequencies in irrelevant articles, provided the frequency of relevant articles in Medline is low. The trained classifier then ranks each article in Medline by score (log of the odds of relevance) and returns articles scoring greater than 0, subject to an upper limit on the number of results.

**Table 1:** Feature scores for PG07.

Score	*R*	$\bar{R}$	*p*(*F*_*i *_= 1|*R*)	*p*(*F*_*i *_= 1|$\bar{R}$)	*z*_*i*_	Type	Term String
8.27	56	140	3.5E-2	9.0E-6	1.3E-5	issn	1744–6872 (Pharmacogenet. Genomics)
7.36	137	855	8.6E-2	5.5E-5	6.4E-5	issn	0960-314X (Pharmacogenetics)
7.24	41	287	2.6E-2	1.8E-5	2.1E-5	issn	1470-269X (Pharmacogenomics J.)
6.85	6	62	3.8E-3	4.0E-6	4.4E-6	mesh	Organic Anion Transport Polypeptide C
5.95	20	509	1.3E-2	3.3E-5	3.4E-5	issn	1462–2416 (Pharmacogenomics)
5.88	31	847	1.9E-2	5.4E-5	5.6E-5	mesh	Steroid 16-alpha-Hydroxylase
5.84	70	1986	4.4E-2	1.3E-4	1.3E-4	mesh	Cytochrome P-450 CYP2D6
5.84	2	57	1.3E-3	3.7E-6	3.8E-6	mesh	Glucuronic Acids
5.79	13	390	8.2E-3	2.5E-5	2.6E-5	mesh	Mephenytoin
5.78	114	3434	7.1E-2	2.2E-4	2.3E-4	mesh	Pharmacogenetics
5.69	1	33	6.3E-4	2.1E-6	2.2E-6	mesh	Methenyltetrahydrofolate Cyclohydrolase
5.54	7	268	4.4E-3	1.7E-5	1.8E-5	mesh	Xeroderma Pigmentosum Group D Protein
5.53	2	78	1.3E-3	5.0E-6	5.1E-6	mesh	Methylthioinosine
5.42	5	216	3.1E-3	1.4E-5	1.4E-5	mesh	Organic Anion Transporters, Sodium-Independent

The support scores for feature occurrence, for retrieval using PG07. "*R*" denotes |*R* ∩ Fi = 1| and "$\bar{R}$" denotes |$\bar{R}$ ∩ Fi = 1|, which are the number of example and rest-of-Medline articles with each feature. p(Fi = 1|*R*) and p(Fi = 1|$\bar{R}$ are the posterior probabilities for feature occurrence, using zi as the prior count.

**Figure 2:**
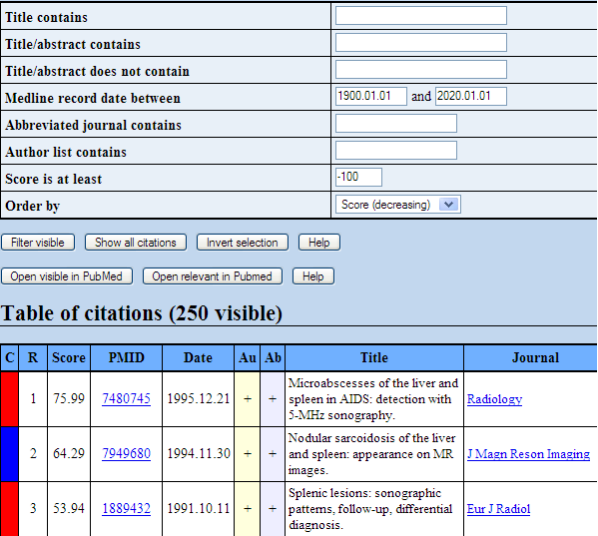
**Results page**. The first page of results when trained on the PG07 corpus. The page contains JavaScript for sorting and searching within results, saving manual selections to disk and opening selected results in PubMed.

The results pages, an example of which is shown in Figure 2, contain full abstracts and links to PubMed, and feature a JavaScript application for instantaneous filtering and sorting by different fields. The pages also have a facility for manually marking relevant abstracts to open in PubMed or save to disk. The complete output directory can be downloaded as a zip file. Additionally, the front output page lists the MeSH terms with the greatest Term Frequency/Inverse Document Frequency [30], which provides potentially useful information about the nature of the input set and suggests useful keywords to use with a search engine. In total, the whole-Medline classification takes 60–90 seconds to return results on a Sun Fire 280R, which is comparable to web services such as NCBI BLAST. The core step of calculating classifier scores for the over 16 million articles in Medline has been optimised down to 32 seconds.

The submission form allows some of the classifier parameters to be adjusted. These include setting an upper limit on the number of results, or restricting Medline to records completed after a particular date (useful when monitoring for new results). More specialised options include the estimated fraction of relevant articles in Medline (prevalence), and the minimum score to classify an article as relevant. Higher estimated prevalence produces more results by raising the prior probability of relevance (see Methods), while higher prediction thresholds return fewer results, for greater overall precision at the cost of recall.

### Cross validation protocol

The web interface provides a 10-fold cross validation function. The input examples are used as the relevant corpus, and up to 100,000 PubMed IDs are selected at random from the remainder of Medline to approximate an irrelevant corpus. In each round of cross validation, 90% of the data is used to estimate term frequencies, and the trained classifier is used to calculate article scores for the remaining 10%. Graphs derived from the cross validated scores include article score distributions, the ROC curve [16] and the curve of precision as a function of recall. Metrics include area under ROC and average precision [30].

Below, we applied cross validation to training examples from three topics (detailed in Methods) and one control corpus, to illustrate different use cases. The PG07 corpus consists of 1,663 pharmacogenetics articles, for the use case of curating a domain-specific database. The AIDSBio corpus consists of 10,727 articles about AIDS and bioethics, for the case of approximating a complex query or extending a text mining corpus. The Radiology corpus consists of 67 articles focusing on splenic imaging, for the case of extending a personal bibliography. The Control corpus consists of 10,000 randomly selected citations, and exists to demonstrate worst-case performance when the input has the same term distribution as Medline. We derived the irrelevant corpus for each topic from a single corpus, Medline100K, of 100,000 random Medline records. For each topic, we create the irrelevant corpus by taking Medline100K and subtracting any overlap with the relevant training examples. This differs from the web interface, which generates an independent irrelevant corpus every time it is used. A summary of the cross validation statistics for the sample topics is presented in Table 2.

**Table 2:** Cross validation statistics.

**Statistic**	**PG07**	**Radiology**	**AIDSBio**	**Control**
**# Relevant**	1663	67	10727	10000
**# Irrelevant**	99986	100000	99927	99955
**Prevalence**	0.01636	0.00067	0.09702	0.09095

**ROC Area**	0.9754	0.9923	0.9913	0.4975
**ROC Std Error**	0.0020	0.0047	0.0004	0.0030
**Averaged Precision**	0.693	0.711	0.924	0.090
**Break-Even**	0.652	0.642	0.884	0.089

Summary of the cross validation training sets and performance metrics. Prevalence is the fraction of the data that is relevant, and break-even is point where cross validation precision equals recall.

### Distributions of article scores

**Figure 3:**
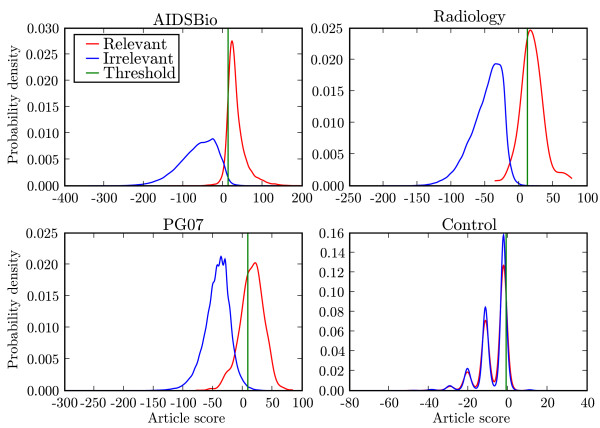
**Article score distributions**. For each topic, a pair of article score distributions are shown for the relevant articles (red curve) and the irrelevant articles (blue curve). The vertical lines mark the score threshold that has precision equal to recall in each case. Irrelevant articles were derived from Medline100K in each case.

The article score distributions for relevant and irrelevant documents for each topic are shown in Figure 3. We have marked with a vertical line the score threshold that would result in equal precision and recall. The areas above the threshold represent the true and false positive rates, while areas below the threshold represent true and false negative rates [16]. The low prevalence of relevant documents in Medline for a given topic of interest places stringent requirements on acceptable false positive rates when the classifier is applied to all of Medline. For example, a score threshold capturing 90% of relevant articles and 1% of irrelevant articles yields only 8% precision if relevant articles occur at a rate of one in a thousand. For our sample topics, the article score distributions for AIDSBio and Radiology were better separated from their irrelevant corpus than for PG07. As expected, the distribution of the Control corpus overlapped entirely with the irrelevant articles, indicating no ability to distinguish the control articles from Medline.

### Receiver Operating Characteristic

**Figure 4:**
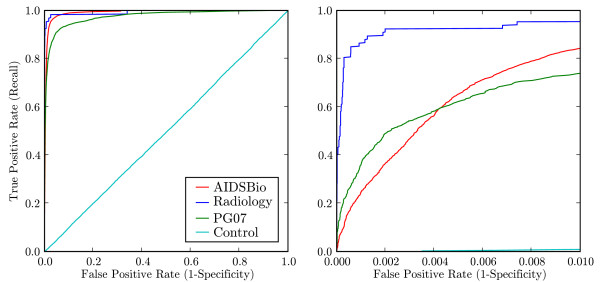
**Receiver Operating Characteristic**. ROC curve cross validation of each sample topic against 100,000 irrelevant articles. Because Medline retrieval requires low false positive rates, we have shown the ROC curve up to 1% false positives on the right.

The ROC curve [16] for each topic is shown in Figure 4. We summarise the ROC using the area under curve (AUC) statistic, representing the probability that a randomly selected relevant article will be ranked above a randomly selected irrelevant article. We calculated the standard error of the AUC using the tabular method of Hanley [31]. Worst-case performance was obtained for the Control corpus, as expected, with equal true and false positive rates and 0.5 falling within the standard error of the AUC. In the theoretical best case, all relevant articles would be retrieved before any false positives occur (top left corner of the graph). The AUC for PG07 in Table 2 (0.9754 ± 0.0020) was significantly lower than the AUC for AIDSBio (0.9913 ± 0.0004) and Radiology (0.9923 ± 0.0047), which did not differ significantly. The lower AUC for PG07, and the poorer separation of its score distribution from Medline background, may be because pharmacogenetics articles discuss the interaction of a drug and a gene (requiring the use of relationship extraction [32]), which may not always be represented in the MeSH feature space.

### Precision under cross validation

**Figure 5:**
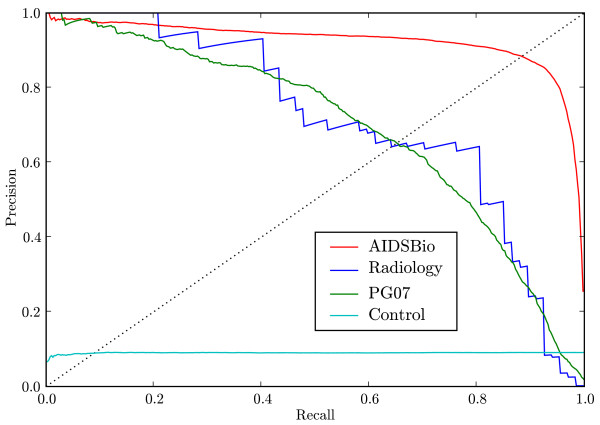
**Precision as a function of recall**. Cross validation precision as a function of recall for each sample topic. The points where precision equals recall occur at the intersection with the dotted diagonal line.

We evaluated cross validation precision at different levels of recall in Figure 5, where the precision represents the fraction of articles above the prediction threshold that were relevant. To summarise the curve we evaluated precision at each point where a relevant article occurred and averaged over the relevant articles [30]. The averaged precisions for AIDSBio, Radiology and PG07 in Table 2 were 0.92, 0.71 and 0.69 respectively. As an overall summary, the Mean of Averaged Precisions (MAP) [30] across the three topics was 0.77. In Additional File 1 we provide 11-point interpolated precision curves for these topics and for the IEDB tasks below, to facilitate future comparisons to our results. As expected for the Control corpus, precision at all thresholds was roughly equal to the 9% prevalence of relevant articles in the data. AIDSBio and Radiology had comparable ROC areas, but the averaged precision for Radiology was much lower than for AIDSBio. This is because prevalence (prior probability of relevance) is much lower for Radiology than AIDSBio: 0.067% vs 9.7% in Table 2. For a given recall and false positive rate, precision depends non-linearly on the ratio of relevant to irrelevant documents, while ROC is independent of that ratio [33].

### Performance in a retrieval situation

To evaluate classification performance in a retrieval situation we compared the performance of MScanner to the performance of an expert PubMed query that was used to identify articles for the Immune Epitope Database (IEDB). We made use of the 20,910 results of a sensitive expert query that had been manually split into 5,712 relevant and 15,198 irrelevant articles for the purpose of training the IEDB classifier [15]. MeSH terms were available for 20,812 of the articles, of which 5,680 were relevant and 15,132 irrelevant. The final data set is provided in Additional File 2. To create training and testing corpora, we first restricted Medline to the 783,028 records completed in 2004, a year within the date ranges of all components of the IEDB query. For relevant training examples we used the 3,488 relevant IEDB results from before 2004, and we approximated irrelevant training examples using the whole of 2004 Medline. We then used the trained classifier to rank the articles in 2004 Medline.

**Figure 6:**
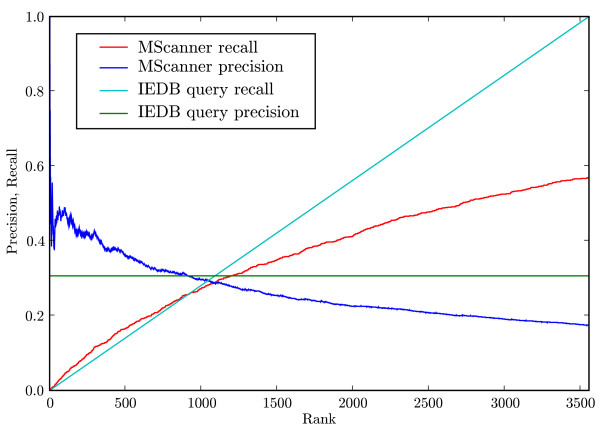
**Comparison to the IEDB expert query**. Precision and recall as a function of rank, comparing MScanner and the IEDB query at the task of retrieving IEDB-relevant articles from 2004 Medline. MScanner was trained on the pre-2004 relevant results of the IEDB query.

We compared precision and recall as a function of rank for MScanner and the IEDB boolean query in Figure 6, for the task of retrieving IEDB-relevant citations from 2004 Medline. The IEDB query had 3,544 results in 2004 Medline, of which 1,089 had been judged relevant and 2,465 irrelevant, for 30.6% precision and 100% recall (since the data set was defined by the query). Since the IEDB query results were unranked, we assumed constant precision for plotting its curves. Up until about 900 results, MScanner recall and precision are above those of the IEDB query. At 3,544 results, MScanner's relative recall was 57% and its precision was 17.4%. Precisions after retrieving *N *results are as follows: P10 = 50%, P50 = 44%, P100 = 49%, P200 = 44% and P500 = 37%.

### Performance/speed trade-off

We also compared MScanner to the IEDB classifier on its cross validation data, to evaluate the trade-off between performance and speed. The IEDB uses a Naïve Bayes classifier with word features derived from a concatenation of abstract, authors, title, journal and MeSH, followed by an information gain feature selection step and extraction of domain-specific features (peptides and MHC alleles). Using cross-validation to calculate scores for the collection of 20,910 documents, the IEDB classifier obtained an area under ROC curve of 0.855, with a classification speed (after training) of 1,000 articles per 30 seconds. MScanner, using whole MeSH terms and ISSN features, obtained an area under ROC of 0.782 ± 0.003, with a classification speed of approximately 15 million articles per 30 seconds. However, the prior we used for frequency of term occurrence (see Methods) is designed for training data where the prevalence of relevant examples is low. The prevalence of 0.27 in the IEDB data is much higher than the prevalences in Table 2, and using the Laplace prior here would improve the ROC area to 0.825 ± 0.003 but degrade performance in cross validation against Medline100K. The remaining difference in ROC between MScanner and the IEDB classifier reflects information from the abstract and domain-specific features not captured by the MeSH feature space. All ROC AUC values on the IEDB data are much lower than in the sample cross validation topics. This is because it is more difficult to distinguish between relevant and irrelevant articles among the closely related articles resulting from an expert query, than to distinguish relevant articles from the rest of Medline.

## Discussion

### Uses of supervised learning for Medline retrieval

Supervised learning has already been applied to the problem of database curation and the development of text mining resources. However, using a web service like MScanner to perform supervised learning is a simple operation compared to constructing a boolean filter, gold standard training set, and custom-built classifier. MScanner may supplement existing workflows that use a pre-filter query by detecting relevant articles inadvertently excluded by the filter. Another possibility is using MScanner in place of a filter query when one is unavailable, and confirming relevance by passing on the results to a stronger classifier or an information extraction method such as that used by the Database of Interacting Proteins [25]. Supervised learning can also be used in other scenarios where relevant training examples are readily available and the presence of many relevant features hinders ad-hoc retrieval. For example, individual researchers could leverage the documents in a personal bibliography to identify additional articles relevant to their research interests.

### Performance evaluation

MScanner's performance varies by topic, depending on the degree to which features are enriched or depleted in relevant articles compared to Medline. The relative performance on different corpora also depends on the evaluation metric used. For example, ROC performance on PG07 shows lower overall ability to distinguish pharmacogenetics articles from Medline, but the right hand sub-plot of Figure 4 shows higher recall at low false positive rates on PG07 than AIDSBio. Besides the topic itself, the size of the training set can also influence performance. For the complex topics curated by databases, many relevant examples may be needed to obtain good coverage of terms indicating relevance. Narrower topics, such as the Radiology corpus, require fewer training examples to obtain good estimates of the frequencies of important terms. Too few training examples, however, will result in poor estimates of term frequencies (over-estimates, or failure of important terms to be represented), degrading performance. The use of a random set of Medline articles as the set of irrelevant articles in training (Medline100K in the use cases we presented) can also influence performance in cross validation. It can inflate the false positive rate to some extent because it contains relevant articles that are not part of the relevant training set.

The score distributions for the Control corpus (Figure 3) were somewhat anomalous, with multiple narrow modes. This is due to the larger irrelevant corpus derived from Medline100K containing low-frequency features not present in the Control corpus. Each iteration of training therefore yielded many rare features with scores around -8 to -10. The four narrow peaks correspond to the chance presence of 0, 1, 2 or 3 of those features, which were influential because other features scored close to zero. In non-random corpora (AIDSBio, PG07 and Radiology), the other non-zero features dominate to produce broader unimodal distributions. Removing features unique to Medline100K reduced the Control distribution to the expected single narrow peak between -5 and +5.

### Document representations

We represented Medline records as binary feature vectors derived from MeSH terms and journal ISSNs. These are separate feature spaces: a MeSH term and ISSN consisting of the same string would be not be considered the same feature. Medline provides each MeSH term in a record as a descriptor in association with zero or more qualifiers, as in "Nevirapine/administration & dosage". To reduce the dimensionality of the feature space we treat the descriptor and qualifier as separate features. We detected 24,069 distinct MeSH features in use, and 17,191 ISSN features, for an average of 13.5 features per record. The 2007 MeSH vocabulary comprises 24,357 descriptors and 83 qualifiers. Of the journals, about 5,000 are monitored by PubMed and the rest are represented by a only few records each. An advantage of the MeSH and ISSN feature spaces is that they allow a compact document representation using 16-bit features, which increases classification speed. MeSH is also a controlled vocabulary, and so does not have word sense ambiguities like free text. However the vocabulary does not cover all concepts, and covers some areas of biology and medicine (such as medical terminology) more densely than others. Also, not every article has all relevant MeSH terms assigned, and there is a tendency for certain terms to be assigned to articles that just discuss the topic, such as articles "about dental research" rather than dental research articles themselves [34].

Performance can be improved by adding an additional space of binary features derived from the title and abstract of the document. Not relying solely on MeSH features would also enable classification of Medline records that have not been assigned MeSH descriptors yet. The additional features would, however, reduce classification speed due to larger document representations, introduce redundancy with the MeSH feature space, and require a feature selection step. The IEDB classifier [15] avoids redundancy by concatenating the abstract with the MeSH terms and using a single feature space of text words. Binary features should model short abstracts relatively well, although performance on longer texts is known to benefit from considering multiple occurrences of terms [35,36].

MeSH annotations and journal ISSNs are domain-specific resources in the biomedical literature. The articles cited by a given article (although not provided in Medline) are another domain-specific resource that may prove useful in retrieval tasks, in addition to their uses in navigating the citation network. For example, the overlap in citation lists has been used as a benchmark for article relatedness [29]. In supervised learning, it may be possible to incorporate the number of co-citations between a document and relevant articles, or to use the citing of an article as a binary feature.

## Conclusion

MScanner inductively learns topics of interest from example citations, with the aim of retrieving a large number of topical citations more effectively than with boolean queries. It represents an advance on previous tools for Medline classification by performing well across a range of topics and input sizes, by making available implementation source code, and by operating on all of Medline fast enough to use over a web interface. As a non-domain-specific classifier, it has a facility for performing cross validation to obtain ROC and precision statistics on new inputs. MScanner should be useful as a filter for database curation where a sensitive filter query and customised classifier are not already available, and in general for constructing large bibliographies, text mining corpora and other domain-specific Medline subsets.

## Methods

### Bayesian classification

MScanner uses a Naïve Bayes classifier, which places documents in the class with the greatest posterior probability, and is derived by assuming that feature occurrences are conditionally independent with respect to the class variable. In the multivariate Bernoulli document model [35], each document is represented as a binary vector, *f *= (*f*_1_, *f*_2_,...,*f*_*k*_), with 1 or 0 specifying the presence or absence of each feature. The score of the article is the logarithm of the posterior probability ratio for the article being relevant versus irrelevant, which reduces to a sum of feature support scores and a prior score:

$$\begin{array}{lll} S(f) & = & \log\frac{p(F=f|R)p(R)}{p(F=f|\bar{R})p(\bar{R})}\\ & = & \log\prod_{i=1}^{k}\frac{p(F_{i}=f_{i}|R)}{p(F_{i}=f_{i}|\bar{R})}+\log\frac{p(R)}{p(\bar{R})}\\ & = & \sum_{i=1}^{k}Y(F_{i}=f_{i})+\log\frac{p(R)}{1-p(R)} \end{array}$$

The feature support scores [37] are:

$$Y(F_{i}=f_{i})=\log\frac{p(F_{i}=f_{i}|R)}{p(F_{i}=f_{i}|\bar{R})}$$

The greatest support scores for occurring features are shown in Table 1, when the classifier has been trained to perform PG07 retrieval. For computational efficiency, the non-occurrence support scores, *Y*(*F*_*i *_= 0), are simplified to a base score (of an article with no features) and a small adjustment for each feature that occurs.

We estimate the prior probability of relevance *P*(*R*) using the number of training examples divided by the number of articles in Medline, and the classifier predicts relevance for articles with *S*(*f*) ≥ 0. The prior and minimum score for predicting relevance may also be set on the web interface.

### Estimation of feature frequencies

We use posterior estimates for *p*(*F*_*i *_= *f*_*i*_|*R*) and *p*(*F*_*i *_= *f*_*i*_|$\bar{R}$). We choose the prior probability *z*_*i *_of observing the feature to be the fraction of articles in all of Medline in which the feature occurs. The weight of the prior is equivalent to one article worth of evidence, resulting in probabilities of the following form:

$$p(F_{i}=1|R)=\frac{p(R\cap (F_{i}=1))}{p(R)}=\frac{|R\cap (F_{i}=1)|+z_{i}}{|R|+1}$$

And similarly for *p*(*F*_*i *_= 1|$\bar{R}$). Probabilities for non-occurrence of features are of the form *p*(*F*_*i *_= 0|*R*) = 1 - *p*(*F*_*i *_= 1|*R*). Bayesian classifiers normally use a Laplace prior [35], which specifies one prior success and one prior failure for each feature. However, the Laplace prior performs poorly here because of class skew in the training data: when irrelevant articles greatly outnumber relevant ones it over-estimates *P*(*F*_*i *_= 1|*R*) relative to *P*(*F*_*i *_= 1|$\bar{R}$), in particular for terms not observed in any relevant examples.

### Data structures enabling fast classification

MScanner's classification speed is due to the use of a Bayesian classifier, a compact feature space, and a customised implementation. Training in retrieval tasks is made much faster by keeping track of the total number of occurrences of each term in Medline. The MeSH and ISSN feature spaces fit in 16-bit feature IDs, and each Medline record has an average of 13.5 features. Including some overhead, this allows the features of all 16 million articles in Medline to be stored in a binary stream of around 600 MB. A C program takes 32 seconds to parse this file and calculate article scores for all of Medline, returning those above the specified threshold. The rest of the program is written in Python [38], using the Numpy library for vector operations. Source code is provided in Additional File 3.

For storing complete Medline records, we used a 22 GB Berkeley DB indexed by PubMed ID. It was generated by parsing the Medline Baseline [39] distribution, which consists of 70 GB XML compressed to 7 GB and split into files of 30,000 records each. During parsing, a count of the number of occurrences of each feature in Medline is maintained, ready to be used for training the classifier. To look up feature vectors in cross validation, we use a 1.3 GB Berkeley DB instead of the binary stream.

### Construction of PG07, AIDSBio, Radiology and Medline100K

The PG07, AIDSBio and Radiology corpora provided in Additional File 4 are from different domains and are of different sizes, to illustrate the different use cases mentioned in the results. The PG07 corpus comprises literature annotations taken from the PharmGKB [8] on 5 February 2007. The AIDSBio corpus is the intersection of the PubMed AIDS [40] and Bioethics [41] subsets on 19 October 2006. The Radiology corpus is a bibliography of 67 radiology articles focusing on the spleen, obtained from a co-worker of DR's. The corpora exclude records that do not have status "MEDLINE", and thus lack MeSH terms. The Medline100K corpus consists of 100,000 randomly selected Medline records, with completion dates up to 21 January 2007, which is also the upper date for the Control corpus of 10,000 random citations. The size of Medline100K was chosen to provide a good approximation of the Medline background, while containing few unknown relevant articles.

## Availability and requirements

• **Project Name: **MScanner

• **Home Page: **

• **Operating Systems: **Platform independent

• **Programming Languages: **Python, JavaScript, C

• **Minimum Requirements: **Internet Explorer 7, Mozilla Firefox 2, Opera 9, or Safari 3

• **License: **GNU General Public License

## Authors' contributions

GP and CS in collaboration with DR and RA conceived of the goals for MScanner, including a web interface and refining the classifier formulation. GP programmed the MScanner software and web interface, developed and carried out experiments to analyse MScanner's performance with feedback from CS, DR and RA, and wrote the manuscript drafts. CS supervised the research and reviewed all drafts of the manuscript. All authors read and approved the final draft of the paper.

## Supplementary Material

Additional file 1 **11-point precision-recall curves**. 11pointcurves.pdf is a PDF file containing a table of 11-point interpolated precision curves for all experiments in the paper. The interpolated precision at a specified recall is the highest precision found for any value of recall greater than or equal to the specified recall. Click here for file

Additional file 2 **Corpora used in the IEDB comparison**. iedb.zip is a ZIP archive containing text files, where each line contains the PubMed ID and completion date of a Medline record. iedb-all-relevant.txt and iedb-all-irrelevant.txt are the relevant and irrelevant cross validation corpora used in the IEDB cross validation. iedb-pre2004-relevant.txt are the relevant training examples for the retrieval comparison. iedb-2004-relevant.txt and iedb-2004-irrelevant.txt are the manually evaluated IEDB query results from 2004 Medline. PubMed IDs for 2004 Medline may be obtained using the PubMed query 2004 [DateCompleted] AND medline [sb]. Click here for file

Additional file 3 **Source code for MScanner**. mscanner-20071123.zip is a ZIP archive containing the Python 2.5 source code for MScanner, licensed under the GNU General Public License. It also contains API documentation in HTML format. Updated versions will be made available at . Click here for file

Additional file 4 **Sample cross validation corpora**. corpora.zip is a ZIP archive containing text files for the PG07, AIDSBio, Radiology, Control and Medline100K sample corpora. Each line contains the PubMed ID and completion date of a Medline record. Click here for file
