# Supplementary material for: MScanner: a classifier for retrieving Medline citations
Source: BMC Bioinformatics. 2008 Feb 19;9:108. doi: 10.1186/1471-2105-9-108 (PMC2263023; doi:10.1186/1471-2105-9-108)

11-point interpolated precision curves

Recall	0.0	0.1	0.2	0.3	0.4	0.5	0.6	0.7	0.8	0.9	1.0
AIDSBio	1.00	0.98	0.97	0.96	0.95	0.94	0.94	0.93	0.91	0.87	0.10
Radiology	1.00	1.00	1.00	0.93	0.93	0.71	0.68	0.65	0.64	0.24	0.00
PG07	1.00	0.97	0.93	0.88	0.85	0.79	0.70	0.62	0.46	0.26	0.02
Control	0.09	0.09	0.09	0.09	0.09	0.09	0.09	0.09	0.09	0.09	0.09
IEDB validation	0.75	0.72	0.72	0.70	0.67	0.64	0.58	0.52	0.42	0.35	0.27
IEDB query	1.00	0.44	0.36	0.28	0.23	0.21	0.16	0.12	0.08	0.04	-

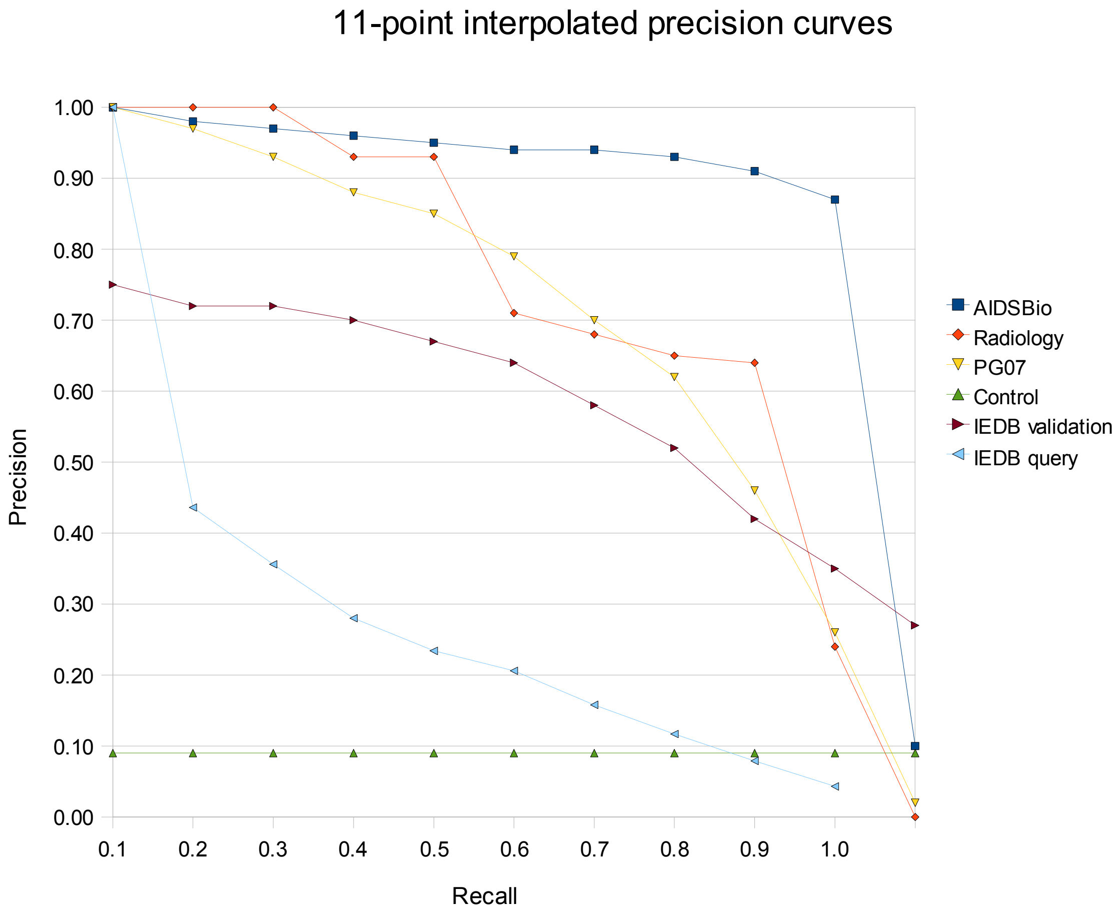

Supplement: Additional file 1 — 11-point precision-recall curves. 11pointcurves.pdf is a PDF file containing a table of 11-point interpolated precision curves for all experiments in the paper. The interpolated precision at a specified recall is the highest precision found for any value of recall greater than or equal to the specified recall. [file 1471-2105-9-108-S1.pdf]
