# Supplementary material for: MScanner: a classifier for retrieving Medline citations
Source: BMC Bioinformatics. 2008 Feb 19;9:108. doi: 10.1186/1471-2105-9-108 (PMC2263023; doi:10.1186/1471-2105-9-108)
Supplement: Additional file 3 — Source code for MScanner. mscanner-20071123.zip is a ZIP archive containing the Python 2.5 source code for MScanner, licensed under the GNU General Public License. It also contains API documentation in HTML format. Updated versions will be made available at . [file 1471-2105-9-108-S3.zip › mscanner/core/templates/citations.tmpl]

#\* Table of citations
$cite\_table -- HTML for citation table
$dataset -- Name of data set
$mode -- 'input' or 'output'
$report\_length -- Number of citations in this page of the report
$cur\_idx -- Index into $filelist of the current file name
$filelist -- List of names of all HTML files in the set
\*#
#from mscanner.configuration import rc

#if $mode == "input"
Input Citations for $dataset
#else
Result Citations for $dataset
#end if

#if $rc.link\_headers
## Link to original JS and CSS instead of including them
#set $linkpath = $rc.templates.relpath().replace('\\','/')
#else
#end if


# #if $mode == "input" Input Citations for $dataset #else Output Citations for $dataset #end if

Navigation:
## Link to the previous file in the list
#if $cur\_idx > 0
Previous 
#end if
## Link to all preceding files
#for idx in range($cur\_idx)
#echo $idx+1# 
#end for
## Link to this file
**#echo $cur\_idx+1#** 
## Link to all following files (NOTE: also used by the result-appender)
#for idx in range($cur\_idx+1, len($filelist))
#echo $idx+1# 
#end for
## Link to the next file in the list
#if len($filelist) > $cur\_idx+1
Next
#end if

This report contains $report\_length citations.
#if $mode == "input"
These are the input citations, used to train MScanner.
#else if $mode == "output"
These are result citations.
#end if

Warning: page scripts have not run - all things clickable will not
function until they do. If the page is taking a while to load, consider
downloading the zip file of results instead. If your browser is saying that
there is blocked content, click the alert and "Allow blocked content".

Extra Features

Extra features: it is possible to save/load manual
classifications (assigned by clicking in the "C" column) to file. To use this
feature, save a local copy of this file (e.g. using "Save As" or from the zip
archive) and open it in Mozilla Firefox or Internet Explorer 7. To use
another feature where later results pages can be appended to the table, open
the file in Mozilla Firefox.

|  |  |
| --- | --- |
| Title contains |  |
| Title/abstract contains |  |
| Title/abstract does not contain |  |
| Medline record date between | and |
| Abbreviated journal contains |  |
| Author list contains |  |
| Score is at least  | |
| Order by | Score (decreasing) Date (decreasing) Journal (increasing) Author (increasing) |

Filter visible 
Show all citations 
Invert selection 
Help

*Filter visible* hides any currently visible rows that do not
match the filter criteria. *Show all citations* unhides all hidden
rows. *Invert selection* makes the visible rows hidden and the
hidden rows visible.

The "Medline record date" is not the date of publication: it is the
date on which the completed Medline citation record became available,
so that recently added citations can be filtered for.

The title and abstract filters are case-insensitive regular
expressions. They match substrings, but have many additional features.
For example, "rat" matches both "rat" and "aberration", but "\brat\b" will
only match "rat" ("\b" means "word boundary"). To search for both "rat" and
"human", first filter for "rat", then filter for "human".

Open visible in PubMed 
Open relevant in Pubmed 
Help

Opens PubMed in a new window, for the citations that are visible below.
The second button instead opens those citations which have been marked
relevant by clicking once in "C" column (colours changes to red ...)).

Clicking a second time explicitly marks the citation as irrelevant (...). Grey citations ( ...) are not
manually classified".

Save Classifications 
Load Classifications 
Help

#set save\_target = $filelist[$cur\_idx].namebase+".csv"

These buttons will save or load classifications (the marks in the "C"
column as to whether a citation is relevant or irrelevant) to a file
called $save\_target.

Append next result file 
Next up is: . 
Help

Append citations from later results files to the bottom of this one.

## Table of citations ($report\_length visible)

$cite\_table

MScanner © 2007 Graham Poulter
