# Supplementary material for: MScanner: a classifier for retrieving Medline citations
Source: BMC Bioinformatics. 2008 Feb 19;9:108. doi: 10.1186/1471-2105-9-108 (PMC2263023; doi:10.1186/1471-2105-9-108)
Supplement: Additional file 3 — Source code for MScanner. mscanner-20071123.zip is a ZIP archive containing the Python 2.5 source code for MScanner, licensed under the GNU General Public License. It also contains API documentation in HTML format. Updated versions will be made available at . [file 1471-2105-9-108-S3.zip › mscanner/core/templates/notfound.tmpl]

#\*
$dataset -- Title of the data set to print
\*#
#from mscanner.configuration import rc

MScanner error for $dataset

#if $getVar("linkpath", None)
#else
#end if


# MScanner error for $dataset

#include str($rc.templates/"invalid.tmpl")

MScanner © 2007 Graham Poulter
