# Supplementary material for: MScanner: a classifier for retrieving Medline citations
Source: BMC Bioinformatics. 2008 Feb 19;9:108. doi: 10.1186/1471-2105-9-108 (PMC2263023; doi:10.1186/1471-2105-9-108)
Supplement: Additional file 3 — Source code for MScanner. mscanner-20071123.zip is a ZIP archive containing the Python 2.5 source code for MScanner, licensed under the GNU General Public License. It also contains API documentation in HTML format. Updated versions will be made available at . [file 1471-2105-9-108-S3.zip › mscanner/core/templates/results.tmpl]

#\* Index to the query outputs
$QM -- QueryManager instance used for the query
\*#
#from mscanner.configuration import rc

MScanner query results: $QM.dataset

#if $rc.link\_headers
## Link to original JS and CSS instead of including them
#set $linkpath = $rc.templates.relpath().replace('\\','/')
#else
#end if
#def help(name) #if $name is not None ? #end if |#end def
#def hr(name, value)| $value | | |
#end def

# MScanner query results: $QM.dataset

## Query Results

|  |  |
| --- | --- |
| Number of results | #echo len($QM.results) # |$help(None)
#if len($QM.results) > 0| Lowest scoring result | #echo "%.5f" % $QM.results[-1][0] # |$help(None)
| Abstracts of results | $rc.report\_result\_citations |$help("h\_result\_citations")
$hr("h\_result\_citations", """Complete abstracts of the
articles in Medline predicted to be relevant (limited number per page).""")| Abstracts of all results | ZIP file or $rc.report\_result\_all |$help("h\_results\_all")
$hr("h\_results\_all", """All result abstracts on a single page.
(can take a long time to load - downloading the zip file is recommended)""")
#end if| Abstracts of input examples | $rc.report\_input\_citations |$help("h\_input\_citations")
$hr("h\_input\_citations", """Complete abstracts of the
Medline records given as relevant training examples. They have been
ranked by classifier scores.""")| Feature score table | ##ZIP file or $rc.report\_term\_scores |$help("h\_csv")
$hr("h\_csv", """CSV spreadsheet detailing the calculation of
the feature support scores.""")| PubMed IDs of results | $rc.report\_result\_scores |$help("h\_result\_scores")
$hr("h\_result\_scores", """PubMed IDs and scores of classifier predictions,
ranked by decreasing score.""")| PubMed IDs of inputs | $rc.report\_input\_scores |$help("h\_positives")
$hr("h\_positives", """PubMed IDs and scores of the relevant training examples.""")| Started at |#import time $time.strftime("%Y/%m/%d %H:%M:%S GMT", $time.gmtime($QM.timestamp)) |$help("h\_timestamp")
$hr("h\_timestamp", "Time at which query was started")| Finished at |#import time $time.strftime("%Y/%m/%d %H:%M:%S GMT", $time.gmtime()) |$help("h\_written")
$hr("h\_written", """Time at which this file was written.""")
#if isinstance($QM.featinfo.pseudocount, float)| Pseudocount | $QM.featinfo.pseudocount |$help("h\_pseudocount")
$hr("h\_pseudocount", """Prior frequency (estimated background frequency)
of feature occurrences.""")
#end if| Base score | $QM.featinfo.base |$help("h\_base")
$hr("h\_base", """The log likelihood ratio of an empty article (one in
which every feature failed to occur).""")| Prior score | $QM.featinfo.prior |$help("h\_prior")
$hr("h\_prior", """The log of the prior probability ratio for
an article being relevant versus irrelevant (added to log likelihood ratio
to obtain the final score). Equals the logit of the estimated
prevalence of relevant articles in Medline (which may be estimated
from the input size or specified separately).""")| Limit | $QM.limit |$help("h\_limit")
$hr("h\_limit", """The maximum number of results to include.""")
#if $QM.threshold is not None| Threshold | $QM.threshold |$help("h\_threshold")
$hr("h\_threshold", """Default Naive Bayes classification threshold is
zero. This threshold is the minimum log probability ratio for
predicting an article to be relevant.""")
#end if
#if $QM.mindate is not None| Minimum date | $QM.mindate |$help("h\_mindate")
$hr("h\_mindate", """The minimum date considered when parsing Medline
(both when making feature counts, and when querying)""")
#end if
#if $QM.maxdate is not None| Maximum date | $QM.maxdate |$help("h\_maxdate")
$hr("h\_maxdate", """The maximum date considered when parsing Medline
(both when making feature counts, and when querying)""")
#end if

## Print statistics about data set size and feature occurrences
#set global $stats = $QM.featinfo.stats
#include str($rc.templates/"features.tmpl")

## Features with high TF.IDF

Features with TF.IDF above 0.2 or 0.3 could make good keywords. TF.IDF is term frequency times
inverse document frequency, where we treat the set of input citations as a
single document

| TF-IDF | Type | Term | Term ID | Score | Pos | Neg |
| --- | --- | --- | --- | --- | --- | --- |
#set best\_tfidfs = $QM.featinfo.get\_best\_tfidfs(20)
#for termid, tfidf, (term, termtype), score, pos, neg in best\_tfidfs:| #echo "%.2f"%$tfidf# | $termtype | $term | $termid | #echo "%.2f"%$score# | $pos | $neg |
#end for termid

#set notfound\_pmids = $QM.notfound\_pmids
#include str($rc.templates/"invalid.tmpl")

MScanner © 2007 Graham Poulter
