# Supplementary material for: MScanner: a classifier for retrieving Medline citations
Source: BMC Bioinformatics. 2008 Feb 19;9:108. doi: 10.1186/1471-2105-9-108 (PMC2263023; doi:10.1186/1471-2105-9-108)
Supplement: Additional file 3 — Source code for MScanner. mscanner-20071123.zip is a ZIP archive containing the Python 2.5 source code for MScanner, licensed under the GNU General Public License. It also contains API documentation in HTML format. Updated versions will be made available at . [file 1471-2105-9-108-S3.zip › mscanner/core/templates/validation.tmpl]

#\* Validation output page
$VM -- Instance of ValidationBase
\*#
#from mscanner.configuration import rc
#set $vectors = $VM.metric\_vectors ## PerformanceVectors instance
#set $range = $VM.metric\_range ## PerformanceRanges instance
#set $t = $VM.metric\_range.average ## PerformanceMetrics instance

MScanner validation results: $VM.dataset

#if $rc.link\_headers
## Link to original JS and CSS instead of including them
#set $linkpath = $rc.templates.relpath().replace('\\','/')
#else
#end if
#def help(name) #if $name is not None ? #end if |#end def
#def hr(name, value)| $value | | |
#end def

# MScanner validation results: $VM.dataset

## Global information

| Timestamp |#import time $time.strftime("%Y/%m/%d %H:%M:%S GMT", $time.gmtime($VM.timestamp)) |$help("h\_timestamp")
$hr("h\_timestamp", """Date and time at which the query was submitted.""")| Feature score table | ##ZIP file or $rc.report\_term\_scores |$help("h\_csv")
$hr("h\_csv", """CSV spreadsheet detailing the calculation of
the feature support scores.""")| Relevant PubMed IDs | $rc.report\_positives |$help("h\_positives")
$hr("h\_positives", """List of PubMed IDs of the relevant training
examples. Dividing the file into 10 parts yields the cross
validation folds.""")| Irrelevant PubMed IDs | $rc.report\_negatives |$help("h\_negatives")
$hr("h\_negatives", """List of PubMed IDs of the irrelevant
examples (randomly sampled from Medline). Dividing the file into 10
parts yields the cross validation folds.""")| Number of folds | $range.nfolds |$help("h\_nfolds")
$hr("h\_nfolds", """Number of partitions into which the relevant
and irrelevant data sets were split.""")| Prior score | $VM.featinfo.prior |$help("h\_prior")
$hr("h\_prior", """The log ratio of relevant to irrelevant articles
in the cross validation data. This prior log ratio is added to
log likelihood ratios to obtain posterior article scores.""")| Base score | $VM.featinfo.base |$help("h\_base")
$hr("h\_base", """The log likelihood ratio of an empty article (one in
which every feature failed to occur).""")| Average Precision | #echo "%.5f" % $vectors.AvPrec # |$help("h\_avprec")
$hr("h\_avprec", """Precision averaged over all ranks where
an article is retrieved.""")| Area under ROC curve (AUC) | #echo "%.5f" % $vectors.W # |$help("h\_roc")
$hr("h\_roc", """Area under the graph of the true
positive rate versus false positive rate. Equals the probability
that a randomly selected relevant article will be ranked above
a randomly selected irrelevant article.""")| Standard Error of AUC | #echo "%.5f" % $vectors.W\_stderr # |$help("h\_roc\_stderr")
$hr("h\_roc\_stderr", """Standard error of the area
under the ROC curve. Calculated using the method of Hanley (1982).""")

#if not $hasattr($VM, "pred\_low")

## Confusion Matrix at threshold ?

The columns of the confusion matrix are actual categories of the
documents, and the rows are the predicted categories. Hover the mouse over
each of the squares for a full description of the quantity, and the formula
for calculating it.

|  | | Actual | | Totals | Rates |
| --- | --- | --- | --- | --- | --- |
|  |  | Relevant | Irrelevant |  |  |
| Predicted | Relevant' | TP=$t.TP | FP=$t.FP | P'=#echo $t.TP+$t.FP# | PPV=#echo "%.2f"%$t.PPV# |
|  | Irrelevant' | FN=$t.FN | TN=$t.TN | N'=#echo $t.TN+$t.FN# | NPV=#echo "%.5f"%t.NPV# |
| Totals | | P=$t.P | N=$t.N | $t.A | Prev=#echo "%.5f"%$t.prevalence# |
| Rates | | TPR=#echo "%.2f"%$t.TPR# | FPR=#echo "%.5f"%$t.FPR# |  | Acc=#echo "%.5f"%t.accuracy# |

## Precision, Recall and F measure

| Score threshold | #echo "%.2f" % $range.threshold # |$help("h\_threshold")
$hr("h\_threshold", """Articles with log probability ratio
scores higher than this are predicted positive. Either the closest
article score to zero is used, or we chose a threshold to
maximise F score or Utility (which is the case if we achieve
the F score or Utility maxima mentioned below).""")| **Precision (PPV)** π=TP/(TP+FP) | $range.fmt\_stats("precision") |$help("h\_prec")
$hr("h\_prec", """Proportion of predicted positives which are true
positives.""")| **Recall (True Positive Rate / Sensitivity)** | $range.fmt\_stats("recall") |$help("h\_recall")
$hr("h\_recall", """Proportion of positives which were correctly predicted
to be positive.""")| **F1-Measure (α=0.5)** (2\*ρ\*π/(ρ+π)) | $range.fmt\_stats("fmeasure") |$help("h\_f1")
$hr("h\_f1", """Harmonic mean of recall and precision at the threshold
corresponding to the maximum α-weighted F-Measure.""")| **F-Measure (α=$vectors.alpha)** (1/(α/π+(1-α)/ρ)) | $range.fmt\_stats("fmeasure\_alpha") |$help("h\_fma")
$hr("h\_fma", """The F measure evaluated using
the given alpha. 0 <= α <= 1 controls the weight of
precision. When α=0.5, *F=F1*.""")| **Maximum possible F1-Measure** | #echo "%.3f" % $vectors.FM.max() # |$help("h\_max\_f1")
$hr("h\_max\_f1", """This is the F\_1 measure that would be achieved if we
had set α=0.5""")| **Break-Even** (where precision=recall) | #echo "%.3f" % $vectors.breakeven # |$help("h\_break\_even")
$hr("h\_break\_even", """Shared value at the point where Recall = Precision =
F1-measure. Typically the F1-Measure at break-even is slightly lower than the
maximum F1-Measure.""")

## Utility ?

Utility is a weighted sum of True and False positives. A false
positive has utility -1, and a true positive has utility ur,
by default equal to N/P (the assumption being that returning all the
articles should result in utility of zero).

Hence, U = (ur \* TP - FP)/Umax where
Umax = ur \* P is the maximium achievable
utility. If ur defaults to N/P this reduces to
U=(TP/P)-(FP/N).

| **Utility (ur=#echo "%.2f" % $vectors.utility\_r #**) | $range.fmt\_stats("utility") |
| **Maximum possible utility** | #echo "%.3f" % $vectors.U.max() # |

## Miscellaneous Performance Measures

| **Prevalence in cross validation** P/(P+N) | #echo "%.5f" % $t.prevalence # |$help("h\_prevalence")
$hr("h\_prevalence", """Proportion of training data which was positive.""")| **False Positive Rate (FPR)** FPR=FP/(TN+FP)=1-TNR | $range.fmt\_stats("FPR", places=5) |$help("h\_fpr")
$hr("h\_fpr", """Proportion of negatives which were incorrectly predicted
to be positive.""")| **Specificity (TNR)** TNR=TN/(TN+FP)=1-FPR | $range.fmt\_stats("specificity", places=5) |$help("h\_specificity")
$hr("h\_specificity", """Proportion of negatives which were correctly
predicted to be negative.""")| **Error Rate** (FP+FN)/(P+N)=1-Accuracy | $range.fmt\_stats("error", places=5) |  |
| **Enrichment** (= precision/prevalence) | $range.fmt\_stats("enrichment") |$help("h\_enrich")
$hr("h\_enrich", """Precision over prevalence. This is is how much better this
classifiers precision is over a classifier which calls everything positive.""")

#end if cross validation results

#set global $stats = $VM.featinfo.stats
#include str($rc.templates/"features.tmpl")

## Performance graphs

#if $hasattr($VM, "pred\_low")

### Predicted performance

We predict recall and precision as a function of rank
in a retrieval situation (whole-Medline) for two values of prevalence
(fraction of Medline that is relevant) We assume there are between
${VM.pred\_low.relevant} and ${VM.pred\_high.relevant} relevant records
remaining in Medline (which has ${VM.pred\_low.total} records). We
use true and false positive rates to estimate the number of true
and false positives at each rank.

#end if query prediction graph

### Document score distributions

Normalised histograms (sum of bar areas normalised to 1), approximating
probability distributions for relevant and irrelevant article scores. Good
performance is associated with clean separation of the distributions.

### Feature score distribution

Normalised histogram approximating the probability distribution
for feature feature scores (after training on all available data).

#if not hasattr($VM, "pred\_low")

### ROC Curve

True Positive Rate versus False Positive Rate. The closer
to the top left the curve gets, the better. Worst case is a diagonal
line (true positives increasing at the same rate as false
positives).

### Precision-Recall Curve

Precision as a function of Recall. The recall corresponding
to the chosen threshold is marked with a vertical line.
Worst case is a horizontal line at the level of prevalence.

### F measure versus threshold

Precision, Recall and F-measure as a function of threshold.
The chosen threshold is marked with a vertical line.

#end if cross validation results

#set $notfound\_pmids = $VM.notfound\_pmids
#include str($rc.templates/"invalid.tmpl")

MScanner © 2007 Graham Poulter
