# Supplementary material for: MScanner: a classifier for retrieving Medline citations
Source: BMC Bioinformatics. 2008 Feb 19;9:108. doi: 10.1186/1471-2105-9-108 (PMC2263023; doi:10.1186/1471-2105-9-108)
Supplement: Additional file 3 — Source code for MScanner. mscanner-20071123.zip is a ZIP archive containing the Python 2.5 source code for MScanner, licensed under the GNU General Public License. It also contains API documentation in HTML format. Updated versions will be made available at . [file 1471-2105-9-108-S3.zip › mscanner/help/api/Cheetah.CacheRegion.CacheRegion-class.html]

xml version="1.0" encoding="ascii"?


Cheetah.CacheRegion.CacheRegion


| Trees | Indices | Help | | MScanner | | --- | |
| --- | --- | --- | --- | --- |

|  |  |  |  |
| --- | --- | --- | --- |
| Cheetah :: CacheRegion :: CacheRegion :: Class CacheRegion | |  | | --- | | [hide private] | | [frames] | no frames] | |

# Class CacheRegion

  
  

A `CacheRegion` stores some `CacheItem` instances.

This implementation stores the data in the memory of the current
process. If you need a more advanced data store, create a cacheStore
class that works with Cheetah's CacheStore protocol and provide it as the
cacheStore argument to \_\_init\_\_. For example you could use
Cheetah.CacheStore.MemcachedCacheStore, a wrapper around the Python
memcached API (http://www.danga.com/memcached).  
  


|  |  |  |  |
| --- | --- | --- | --- |
| |  |  | | --- | --- | | Nested Classes | [hide private] | | |
|  | \_cacheItemClass  A CacheItem is a container storing:... |


|  |  |  |  |
| --- | --- | --- | --- |
| |  |  | | --- | --- | | Instance Methods | [hide private] | | |
|  | |  |  | | --- | --- | | \_\_init\_\_(self, regionID, templateCacheIdPrefix=`'``'`, cacheStore=None) |  | |
|  | |  |  | | --- | --- | | clear(self)  drop all the caches stored in this cache region |  | |
|  | |  |  | | --- | --- | | getCacheItem(self, cacheItemID)  Lazy access to a cacheItem |  | |
|  | |  |  | | --- | --- | | isNew(self) |  | |


|  |  |  |  |
| --- | --- | --- | --- |
| |  |  | | --- | --- | | Method Details | [hide private] | | |

|  |  |  |
| --- | --- | --- |
| |  |  | | --- | --- | | getCacheItem(self, cacheItemID) |  |   Lazy access to a cacheItem  Try to find a cache in the stored caches. If it doesn't exist, it's created. Returns a `CacheItem` instance. |

  


| Trees | Indices | Help | | MScanner | | --- | |
| --- | --- | --- | --- | --- |

|  |  |
| --- | --- |
| Generated by Epydoc 3.0beta1 on Fri Nov 23 09:13:20 2007 | http://epydoc.sourceforge.net |
