# Supplementary material for: MScanner: a classifier for retrieving Medline citations
Source: BMC Bioinformatics. 2008 Feb 19;9:108. doi: 10.1186/1471-2105-9-108 (PMC2263023; doi:10.1186/1471-2105-9-108)
Supplement: Additional file 3 — Source code for MScanner. mscanner-20071123.zip is a ZIP archive containing the Python 2.5 source code for MScanner, licensed under the GNU General Public License. It also contains API documentation in HTML format. Updated versions will be made available at . [file 1471-2105-9-108-S3.zip › mscanner/help/api/Cheetah.Compiler.MethodCompiler-class.html]

xml version="1.0" encoding="ascii"?


Cheetah.Compiler.MethodCompiler


| Trees | Indices | Help | | MScanner | | --- | |
| --- | --- | --- | --- | --- |

|  |  |  |  |
| --- | --- | --- | --- |
| Cheetah :: Compiler :: MethodCompiler :: Class MethodCompiler | |  | | --- | | [hide private] | | [frames] | no frames] | |

# Class MethodCompiler

  
  

```
GenUtils --+
           |
          MethodCompiler
```

Known Subclasses:
:   AutoMethodCompiler

---


|  |  |  |  |
| --- | --- | --- | --- |
| |  |  | | --- | --- | | Instance Methods | [hide private] | | |
|  | |  |  | | --- | --- | | \_\_init\_\_(self, methodName, classCompiler, initialMethodComment=None, decorator=None) |  | |
|  | |  |  | | --- | --- | | \_\_str\_\_(self) |  | |
|  | |  |  | | --- | --- | | \_appendToPrevStrConst(self, strConst) |  | |
|  | |  |  | | --- | --- | | \_endCallArg(self) |  | |
|  | |  |  | | --- | --- | | \_setupState(self) |  | |
|  | |  |  | | --- | --- | | \_unescapeCheetahVars(self, theString)  Unescape any escaped Cheetah \$vars in the string. |  | |
|  | |  |  | | --- | --- | | \_unescapeDirectives(self, theString)  Unescape any escaped Cheetah \$vars in the string. |  | |
|  | |  |  | | --- | --- | | addAssert(self, expr) |  | |
|  | |  |  | | --- | --- | | addBreak(self, expr) |  | |
|  | |  |  | | --- | --- | | addChunk(self, chunk) |  | |
|  | |  |  | | --- | --- | | addClosure(self, functionName, argsList, parserComment) |  | |
|  | |  |  | | --- | --- | | addContinue(self, expr) |  | |
|  | |  |  | | --- | --- | | addDel(self, expr) |  | |
|  | |  |  | | --- | --- | | addEcho(self, expr, rawExpr=None) |  | |
|  | |  |  | | --- | --- | | addElif(self, expr, dedent=True, lineCol=None) |  | |
|  | |  |  | | --- | --- | | addElse(self, expr, dedent=True, lineCol=None) |  | |
|  | |  |  | | --- | --- | | addExcept(self, expr, dedent=True, lineCol=None) |  | |
|  | |  |  | | --- | --- | | addFilteredChunk(self, chunk, filterArgs=None, rawExpr=None, lineCol=None) |  | |
|  | |  |  | | --- | --- | | addFinally(self, expr, dedent=True, lineCol=None) |  | |
|  | |  |  | | --- | --- | | addFor(self, expr, lineCol=None) |  | |
|  | |  |  | | --- | --- | | addIf(self, expr, lineCol=None)  For a full #if ... |  | |
|  | |  |  | | --- | --- | | addInclude(self, sourceExpr, includeFrom, isRaw) |  | |
|  | |  |  | | --- | --- | | addIndentingDirective(self, expr, lineCol=None) |  | |
|  | |  |  | | --- | --- | | addMethComment(self, comm) |  | |
|  | |  |  | | --- | --- | | addMethDocString(self, line) |  | |
|  | |  |  | | --- | --- | | addOneLineIf(self, expr, lineCol=None)  For a full #if ... |  | |
|  | |  |  | | --- | --- | | addPSP(self, PSP) |  | |
|  | |  |  | | --- | --- | | addPass(self, expr) |  | |
|  | |  |  | | --- | --- | | addPlaceholder(self, expr, filterArgs, rawPlaceholder, cacheTokenParts, lineCol, silentMode=False) |  | |
|  | |  |  | | --- | --- | | addRaise(self, expr) |  | |
|  | |  |  | | --- | --- | | addRawText(self, text) |  | |
|  | |  |  | | --- | --- | | addReIndentingDirective(self, expr, dedent=True, lineCol=None) |  | |
|  | |  |  | | --- | --- | | addRepeat(self, expr, lineCol=None) |  | |
|  | |  |  | | --- | --- | | addReturn(self, expr) |  | |
|  | |  |  | | --- | --- | | addSet(self, expr, exprComponents, setStyle) |  | |
|  | |  |  | | --- | --- | | addSilent(self, expr) |  | |
|  | |  |  | | --- | --- | | addStrConst(self, strConst) |  | |
|  | |  |  | | --- | --- | | addTernaryExpr(self, conditionExpr, trueExpr, falseExpr, lineCol=None)  For a single-lie #if ... |  | |
|  | |  |  | | --- | --- | | addTry(self, expr, lineCol=None) |  | |
|  | |  |  | | --- | --- | | addUnless(self, expr, lineCol=None) |  | |
|  | |  |  | | --- | --- | | addWhile(self, expr, lineCol=None) |  | |
|  | |  |  | | --- | --- | | addWriteChunk(self, chunk) |  | |
|  | |  |  | | --- | --- | | addYield(self, expr) |  | |
|  | |  |  | | --- | --- | | appendToPrevChunk(self, appendage) |  | |
|  | |  |  | | --- | --- | | cleanupState(self)  Called by the containing class compiler instance |  | |
|  | |  |  | | --- | --- | | closeFilterBlock(self) |  | |
|  | |  |  | | --- | --- | | commitStrConst(self)  Add the code for outputting the pending strConst without chopping off any whitespace from it. |  | |
|  | |  |  | | --- | --- | | dedent(self) |  | |
|  | |  |  | | --- | --- | | docString(self) |  | |
|  | |  |  | | --- | --- | | endCacheRegion(self) |  | |
|  | |  |  | | --- | --- | | endCallRegion(self, regionTitle=`'``CALL``'`) |  | |
|  | |  |  | | --- | --- | | endCaptureRegion(self) |  | |
|  | |  |  | | --- | --- | | handleWSBeforeDirective(self)  Truncate the pending strCont to the beginning of the current line. |  | |
|  | |  |  | | --- | --- | | indent(self) |  | |
|  | |  |  | | --- | --- | | indentation(self) |  | |
|  | |  |  | | --- | --- | | isErrorCatcherOn(self) |  | |
|  | |  |  | | --- | --- | | methodBody(self) |  | |
|  | |  |  | | --- | --- | | methodDef(self) |  | |
|  | |  |  | | --- | --- | | methodName(self) |  | |
|  | |  |  | | --- | --- | | methodSignature(self) |  | |
|  | |  |  | | --- | --- | | nextCacheID(self) |  | |
|  | |  |  | | --- | --- | | nextCallRegionID(self) |  | |
|  | |  |  | | --- | --- | | nextCaptureRegionID(self) |  | |
|  | |  |  | | --- | --- | | nextFilterRegionID(self) |  | |
|  | |  |  | | --- | --- | | setCallArg(self, argName, lineCol) |  | |
|  | |  |  | | --- | --- | | setErrorCatcher(self, errorCatcherName) |  | |
|  | |  |  | | --- | --- | | setFilter(self, theFilter, isKlass) |  | |
|  | |  |  | | --- | --- | | setMethodName(self, name) |  | |
|  | |  |  | | --- | --- | | setMethodSignature(self, signature) |  | |
|  | |  |  | | --- | --- | | setting(self, key) |  | |
|  | |  |  | | --- | --- | | startCacheRegion(self, cacheInfo, lineCol, rawPlaceholder=None) |  | |
|  | |  |  | | --- | --- | | startCallRegion(self, functionName, args, lineCol, regionTitle=`'``CALL``'`) |  | |
|  | |  |  | | --- | --- | | startCaptureRegion(self, assignTo, lineCol) |  | |
|  | |  |  | | --- | --- | | turnErrorCatcherOff(self) |  | |
|  | |  |  | | --- | --- | | turnErrorCatcherOn(self) |  | |
|  | |  |  | | --- | --- | | wrapCode(self) |  | |
| **Inherited from `GenUtils`**: `addGetTextVar`, `genCacheInfo`, `genCacheInfoFromArgList`, `genCheetahVar`, `genNameMapperVar`, `genPlainVar`, `genTimeInterval` | |


|  |  |  |  |
| --- | --- | --- | --- |
| |  |  | | --- | --- | | Method Details | [hide private] | | |

|  |  |  |
| --- | --- | --- |
| |  |  | | --- | --- | | addIf(self, expr, lineCol=None) |  |  For a full #if ... #end if directive |

|  |  |  |
| --- | --- | --- |
| |  |  | | --- | --- | | addOneLineIf(self, expr, lineCol=None) |  |  For a full #if ... #end if directive |

|  |  |  |
| --- | --- | --- |
| |  |  | | --- | --- | | addTernaryExpr(self, conditionExpr, trueExpr, falseExpr, lineCol=None) |  |  For a single-lie #if ... then .... else ... directive <condition> then <trueExpr> else <falseExpr> |

  


| Trees | Indices | Help | | MScanner | | --- | |
| --- | --- | --- | --- | --- |

|  |  |
| --- | --- |
| Generated by Epydoc 3.0beta1 on Fri Nov 23 09:13:20 2007 | http://epydoc.sourceforge.net |
