# Supplementary material for: MScanner: a classifier for retrieving Medline citations
Source: BMC Bioinformatics. 2008 Feb 19;9:108. doi: 10.1186/1471-2105-9-108 (PMC2263023; doi:10.1186/1471-2105-9-108)
Supplement: Additional file 3 — Source code for MScanner. mscanner-20071123.zip is a ZIP archive containing the Python 2.5 source code for MScanner, licensed under the GNU General Public License. It also contains API documentation in HTML format. Updated versions will be made available at . [file 1471-2105-9-108-S3.zip › mscanner/help/api/Cheetah.Compiler.ModuleCompiler-class.html]

xml version="1.0" encoding="ascii"?


Cheetah.Compiler.ModuleCompiler


| Trees | Indices | Help | | MScanner | | --- | |
| --- | --- | --- | --- | --- |

|  |  |  |  |
| --- | --- | --- | --- |
| Cheetah :: Compiler :: ModuleCompiler :: Class ModuleCompiler | |  | | --- | | [hide private] | | [frames] | no frames] | |

# Class ModuleCompiler

  
  

```
SettingsManager._SettingsCollector --+    
                                     |    
       SettingsManager.SettingsManager --+
                                         |
                              GenUtils --+
                                         |
                                        ModuleCompiler
```

---


|  |  |  |  |
| --- | --- | --- | --- |
| |  |  | | --- | --- | | Nested Classes | [hide private] | | |
|  | classCompilerClass |
|  | parserClass  This class is a StateMachine for parsing Cheetah source and sending state dependent code generation commands to Cheetah.Compiler.Compiler. |
| **Inherited from `SettingsManager._SettingsCollector`** (private): `_ConfigParserClass` | |


|  |  |  |  |
| --- | --- | --- | --- |
| |  |  | | --- | --- | | Instance Methods | [hide private] | | |
|  | |  |  | | --- | --- | | \_\_getattr\_\_(self, name)  Provide one-way access to the methods and attributes of the ClassCompiler, and thereby the MethodCompilers as well. |  | |
|  | |  |  | | --- | --- | | \_\_init\_\_(self, source=None, file=None, moduleName=`'``DynamicallyCompiledCheetahTemplate``'`, mainClassName=None, mainMethodName=None, baseclassName=None, extraImportStatements=None, settings=None)  MUST BE CALLED BY SUBCLASSES |  | |
|  | |  |  | | --- | --- | | \_\_str\_\_(self) |  | |
|  | |  |  | | --- | --- | | \_addActiveClassCompiler(self, classCompiler) |  | |
|  | |  |  | | --- | --- | | \_finishedClasses(self) |  | |
|  | |  |  | | --- | --- | | \_getActiveClassCompiler(self) |  | |
|  | |  |  | | --- | --- | | \_initializeSettings(self)  A hook that allows for complex setting initialization sequences that involve references to 'self' or other settings. |  | |
|  | |  |  | | --- | --- | | \_popActiveClassCompiler(self) |  | |
|  | |  |  | | --- | --- | | \_setupCompilerState(self) |  | |
|  | |  |  | | --- | --- | | \_spawnClassCompiler(self, className, klass=None) |  | |
|  | |  |  | | --- | --- | | \_swallowClassCompiler(self, classCompiler) |  | |
|  | |  |  | | --- | --- | | addAttribute(self, attribName, expr) |  | |
|  | |  |  | | --- | --- | | addComment(self, comm) |  | |
|  | |  |  | | --- | --- | | addImportStatement(self, impStatement) |  | |
|  | |  |  | | --- | --- | | addImportedVarNames(self, varNames) |  | |
|  | |  |  | | --- | --- | | addModuleDocString(self, line)  Adds a line to the generated module docstring. |  | |
|  | |  |  | | --- | --- | | addModuleGlobal(self, line)  Adds a line of global module code. |  | |
|  | |  |  | | --- | --- | | addModuleHeader(self, line)  Adds a header comment to the top of the generated module. |  | |
|  | |  |  | | --- | --- | | addSpecialVar(self, basename, contents, includeUnderscores=True)  Adds module \_\_specialConstant\_\_ to the module globals. |  | |
|  | |  |  | | --- | --- | | classDefs(self) |  | |
|  | |  |  | | --- | --- | | compile(self) |  | |
|  | |  |  | | --- | --- | | getModuleCode(self) |  | |
|  | |  |  | | --- | --- | | getModuleEncoding(self) |  | |
|  | |  |  | | --- | --- | | importStatements(self) |  | |
|  | |  |  | | --- | --- | | importedVarNames(self) |  | |
|  | |  |  | | --- | --- | | moduleConstants(self) |  | |
|  | |  |  | | --- | --- | | moduleDocstring(self) |  | |
|  | |  |  | | --- | --- | | moduleFooter(self) |  | |
|  | |  |  | | --- | --- | | moduleHeader(self) |  | |
|  | |  |  | | --- | --- | | setBaseClass(self, baseClassName) |  | |
|  | |  |  | | --- | --- | | setCompilerSetting(self, key, valueExpr) |  | |
|  | |  |  | | --- | --- | | setCompilerSettings(self, keywords, settingsStr) |  | |
|  | |  |  | | --- | --- | | setModuleEncoding(self, encoding) |  | |
|  | |  |  | | --- | --- | | setShBang(self, shBang) |  | |
|  | |  |  | | --- | --- | | specialVars(self) |  | |
|  | |  |  | | --- | --- | | timestamp(self, theTime=None) |  | |
|  | |  |  | | --- | --- | | wrapModuleDef(self) |  | |
| **Inherited from `SettingsManager.SettingsManager`**: `copySettings`, `deepcopySettings`, `getConfigString`, `hasSetting`, `setSetting`, `setting`, `settings`, `updateSettings`, `updateSettingsFromConfigFile`, `updateSettingsFromConfigFileObj`, `updateSettingsFromConfigStr`, `updateSettingsFromPySrcFile`, `updateSettingsFromPySrcStr`, `writeConfigFile`  **Inherited from `SettingsManager.SettingsManager`** (private): `_createConfigFile`, `_defaultSettings`  **Inherited from `SettingsManager._SettingsCollector`**: `normalizePath`, `readSettingsFromConfigFile`, `readSettingsFromConfigFileObj`, `readSettingsFromContainer`, `readSettingsFromModule`, `readSettingsFromPySrcFile`, `readSettingsFromPySrcStr`  **Inherited from `SettingsManager._SettingsCollector`** (private): `_getAllAttrsFromContainer`, `_isContainer`  **Inherited from `GenUtils`**: `addGetTextVar`, `genCacheInfo`, `genCacheInfoFromArgList`, `genCheetahVar`, `genNameMapperVar`, `genPlainVar`, `genTimeInterval` | |


|  |  |  |  |
| --- | --- | --- | --- |
| |  |  | | --- | --- | | Class Variables | [hide private] | | |
| **Inherited from `SettingsManager._SettingsCollector`** (private): `_sysPathLock` | |


|  |  |  |  |
| --- | --- | --- | --- |
| |  |  | | --- | --- | | Method Details | [hide private] | | |

|  |  |  |
| --- | --- | --- |
| |  |  | | --- | --- | | \_\_getattr\_\_(self, name)  *(Qualification operator)* |  |   Provide one-way access to the methods and attributes of the ClassCompiler, and thereby the MethodCompilers as well. WARNING: Use .setMethods to assign the attributes of the ClassCompiler from the methods of this class!!! or you will be assigning to attributes of this object instead. |

|  |  |  |
| --- | --- | --- |
| |  |  | | --- | --- | | \_\_init\_\_(self, source=None, file=None, moduleName=`'``DynamicallyCompiledCheetahTemplate``'`, mainClassName=None, mainMethodName=None, baseclassName=None, extraImportStatements=None, settings=None)  *(Constructor)* |  |  MUST BE CALLED BY SUBCLASSES Overrides: SettingsManager.SettingsManager.\_\_init\_\_ *(inherited documentation)* |

|  |  |  |
| --- | --- | --- |
| |  |  | | --- | --- | | \_initializeSettings(self) |  |  ``` A hook that allows for complex setting initialization sequences that involve references to 'self' or other settings.  For example:       self._settings['myCalcVal'] = self._settings['someVal'] * 15         This method should be called by the class' __init__() method when needed.        The dummy implementation should be reimplemented by subclasses. ```   Overrides: SettingsManager.SettingsManager.\_initializeSettings *(inherited documentation)* |

|  |  |  |
| --- | --- | --- |
| |  |  | | --- | --- | | addModuleGlobal(self, line) |  |  Adds a line of global module code. It is inserted after the import statements and Cheetah default module constants. |

  


| Trees | Indices | Help | | MScanner | | --- | |
| --- | --- | --- | --- | --- |

|  |  |
| --- | --- |
| Generated by Epydoc 3.0beta1 on Fri Nov 23 09:13:20 2007 | http://epydoc.sourceforge.net |
