# Supplementary material for: MScanner: a classifier for retrieving Medline citations
Source: BMC Bioinformatics. 2008 Feb 19;9:108. doi: 10.1186/1471-2105-9-108 (PMC2263023; doi:10.1186/1471-2105-9-108)
Supplement: Additional file 3 — Source code for MScanner. mscanner-20071123.zip is a ZIP archive containing the Python 2.5 source code for MScanner, licensed under the GNU General Public License. It also contains API documentation in HTML format. Updated versions will be made available at . [file 1471-2105-9-108-S3.zip › mscanner/help/api/Cheetah.Parser._HighLevelParser-class.html]

xml version="1.0" encoding="ascii"?


Cheetah.Parser.\_HighLevelParser


| Trees | Indices | Help | | MScanner | | --- | |
| --- | --- | --- | --- | --- |

|  |  |  |  |
| --- | --- | --- | --- |
| Cheetah :: Parser :: \_HighLevelParser :: Class \_HighLevelParser | |  | | --- | | [hide private] | | [frames] | no frames] | |

# Class \_HighLevelParser

  
  

```
SourceReader.SourceReader --+    
                            |    
              _LowLevelParser --+
                                |
                               _HighLevelParser
```

---

This class is a StateMachine for parsing Cheetah source and sending
state dependent code generation commands to
Cheetah.Compiler.Compiler.  
  


|  |  |  |  |
| --- | --- | --- | --- |
| |  |  | | --- | --- | | Instance Methods | [hide private] | | |
|  | |  |  | | --- | --- | | \_\_init\_\_(self, src, filename=None, breakPoint=None, compiler=None) |  | |
|  | |  |  | | --- | --- | | \_applyExpressionFilters(self, expr, exprType, rawExpr=None, startPos=None)  Pipes cheetah expressions through a set of optional filter hooks. |  | |
|  | |  |  | | --- | --- | | \_eatDefOrBlock(self, directiveName) |  | |
|  | |  |  | | --- | --- | | \_eatMultiLineDef(self, directiveName, methodName, argsList, startPos, isLineClearToStartToken=False) |  | |
|  | |  |  | | --- | --- | | \_eatRestOfDirectiveTag(self, isLineClearToStartToken, endOfFirstLinePos) |  | |
|  | |  |  | | --- | --- | | \_eatSingleLineDef(self, directiveName, methodName, argsList, startPos, endPos) |  | |
|  | |  |  | | --- | --- | | \_eatToThisEndDirective(self, directiveName) |  | |
|  | |  |  | | --- | --- | | \_filterDisabledDirectives(self, directiveName) |  | |
|  | |  |  | | --- | --- | | \_initDirectives(self) |  | |
|  | |  |  | | --- | --- | | assertEmptyOpenDirectivesStack(self) |  | |
|  | |  |  | | --- | --- | | cleanup(self)  Cleanup to remove any possible reference cycles |  | |
|  | |  |  | | --- | --- | | configureParser(self)  Is called by the Compiler instance after the parser has had a settingsManager assigned with self.setSettingsManager() |  | |
|  | |  |  | | --- | --- | | eatAttr(self) |  | |
|  | |  |  | | --- | --- | | eatBlock(self) |  | |
|  | |  |  | | --- | --- | | eatBreakPoint(self)  Tells the parser to stop parsing at this point and completely ignore everything else. |  | |
|  | |  |  | | --- | --- | | eatCache(self) |  | |
|  | |  |  | | --- | --- | | eatCall(self) |  | |
|  | |  |  | | --- | --- | | eatCallArg(self) |  | |
|  | |  |  | | --- | --- | | eatCapture(self) |  | |
|  | |  |  | | --- | --- | | eatClosure(self) |  | |
|  | |  |  | | --- | --- | | eatComment(self) |  | |
|  | |  |  | | --- | --- | | eatCompiler(self) |  | |
|  | |  |  | | --- | --- | | eatCompilerSettings(self) |  | |
|  | |  |  | | --- | --- | | eatDecorator(self) |  | |
|  | |  |  | | --- | --- | | eatDef(self) |  | |
|  | |  |  | | --- | --- | | eatDefMacro(self) |  | |
|  | |  |  | | --- | --- | | eatDirective(self) |  | |
|  | |  |  | | --- | --- | | eatEOLSlurpToken(self) |  | |
|  | |  |  | | --- | --- | | eatEncoding(self) |  | |
|  | |  |  | | --- | --- | | eatEndDirective(self) |  | |
|  | |  |  | | --- | --- | | eatErrorCatcher(self) |  | |
|  | |  |  | | --- | --- | | eatExtends(self) |  | |
|  | |  |  | | --- | --- | | eatFilter(self) |  | |
|  | |  |  | | --- | --- | | eatIf(self) |  | |
|  | |  |  | | --- | --- | | eatImplements(self) |  | |
|  | |  |  | | --- | --- | | eatInclude(self) |  | |
|  | |  |  | | --- | --- | | eatMacroCall(self) |  | |
|  | |  |  | | --- | --- | | eatMultiLineComment(self) |  | |
|  | |  |  | | --- | --- | | eatPSP(self) |  | |
|  | |  |  | | --- | --- | | eatPlaceholder(self) |  | |
|  | |  |  | | --- | --- | | eatPlainText(self) |  | |
|  | |  |  | | --- | --- | | eatRaw(self) |  | |
|  | |  |  | | --- | --- | | eatSet(self) |  | |
|  | |  |  | | --- | --- | | eatShbang(self) |  | |
|  | |  |  | | --- | --- | | eatSimpleExprDirective(self, directiveName, includeDirectiveNameInExpr=True) |  | |
|  | |  |  | | --- | --- | | eatSimpleIndentingDirective(self, directiveName, callback, includeDirectiveNameInExpr=False) |  | |
|  | |  |  | | --- | --- | | eatSlurp(self) |  | |
|  | |  |  | | --- | --- | | handleEndDef(self) |  | |
|  | |  |  | | --- | --- | | parse(self, breakPoint=None, assertEmptyStack=True) |  | |
|  | |  |  | | --- | --- | | popFromOpenDirectivesStack(self, directiveName) |  | |
|  | |  |  | | --- | --- | | pushToOpenDirectivesStack(self, directiveName) |  | |
|  | |  |  | | --- | --- | | setupState(self) |  | |
| **Inherited from `_LowLevelParser`**: `getAssignmentOperator`, `getCacheToken`, `getCallArgString`, `getCheetahVar`, `getCheetahVarBody`, `getCheetahVarNameChunks`, `getCheetahVarStartToken`, `getCommentStartToken`, `getDefArgList`, `getDirectiveEndToken`, `getDirectiveStartToken`, `getDottedName`, `getEOLSlurpToken`, `getExpression`, `getExpressionParts`, `getIdentifier`, `getMultiLineCommentEndToken`, `getMultiLineCommentStartToken`, `getOperator`, `getPSPEndToken`, `getPSPStartToken`, `getPlaceholder`, `getPyToken`, `getSilentPlaceholderToken`, `getTargetVarsList`, `isLineClearToStartToken`, `matchAssignmentOperator`, `matchCheetahVarInExpressionStartToken`, `matchCheetahVarStart`, `matchCheetahVarStartToken`, `matchColonForSingleLineShortFormDirective`, `matchCommentStartToken`, `matchDirective`, `matchDirectiveEndToken`, `matchDirectiveName`, `matchDirectiveStartToken`, `matchEOLSlurpToken`, `matchExpressionPlaceholderStart`, `matchIdentifier`, `matchMultiLineCommentEndToken`, `matchMultiLineCommentStartToken`, `matchOperator`, `matchPSPEndToken`, `matchPSPStartToken`, `matchPyToken`, `matchTopLevelToken`, `matchVariablePlaceholderStart`, `setSetting`, `setSettingsManager`, `setting`, `settings`, `transformToken`, `updateSettings`  **Inherited from `_LowLevelParser`** (private): `_initializeSettings`, `_makeCheetahVarREs`, `_makeCommentREs`, `_makeDirectiveREs`, `_makePspREs`, `_raiseErrorAboutInvalidCheetahVarSyntaxInExpr`  **Inherited from `SourceReader.SourceReader`**: `__getitem__`, `__getslice__`, `__len__`, `advance`, `atEnd`, `atStart`, `breakPoint`, `checkPos`, `filename`, `find`, `findBOL`, `findEOL`, `getLine`, `getNonWhiteSpace`, `getRowCol`, `getRowColLine`, `getWhiteSpace`, `getc`, `gotoBookmark`, `hasBookmark`, `isLineClearToPos`, `lineNum`, `matchNonWhiteSpace`, `matchWhiteSpace`, `matches`, `peek`, `pos`, `read`, `readTo`, `readToEOL`, `rev`, `rfind`, `setBookmark`, `setBreakPoint`, `setPos`, `splitlines`, `src`, `startswith`, `ungetc`, `validPos` | |


|  |  |  |  |
| --- | --- | --- | --- |
| |  |  | | --- | --- | | Class Variables | [hide private] | | |
|  | \_directiveHandlerNames = `{'from': 'addImportStatement', 'impor...` |
|  | \_simpleExprDirectives = `['pass', 'continue', 'stop', 'return',...` |
|  | \_simpleIndentingDirectives = `['else', 'elif', 'for', 'while', ...` |
| **Inherited from `_LowLevelParser`** (private): `_settingsManager` | |


|  |  |  |  |
| --- | --- | --- | --- |
| |  |  | | --- | --- | | Method Details | [hide private] | | |

|  |  |  |
| --- | --- | --- |
| |  |  | | --- | --- | | \_\_init\_\_(self, src, filename=None, breakPoint=None, compiler=None)  *(Constructor)* |  |   Overrides: SourceReader.SourceReader.\_\_init\_\_ |

|  |  |  |
| --- | --- | --- |
| |  |  | | --- | --- | | \_applyExpressionFilters(self, expr, exprType, rawExpr=None, startPos=None) |  |  ``` Pipes cheetah expressions through a set of optional filter hooks.  The filters are functions which may modify the expressions or raise a ForbiddenExpression exception if the expression is not allowed.  They are defined in the compiler setting 'expressionFilterHooks'.  Some intended use cases:   - to implement 'restricted execution' safeguards in cases where you    can't trust the author of the template.   - to enforce style guidelines       filter call signature:  (parser, expr, exprType, rawExpr=None, startPos=None)  - parser is the Cheetah parser    - expr is the expression to filter.  In some cases the parser will have    already modified it from the original source code form.  For example,    placeholders will have been translated into namemapper calls.  If you    need to work with the original source, see rawExpr.          - exprType is the name of the directive, 'psp', or 'placeholder'. All    lowercase.  @@TR: These will eventually be replaced with a set of    constants.  - rawExpr is the original source string that Cheetah parsed.  This    might be None in some cases.  - startPos is the character position in the source string/file    where the parser started parsing the current expression.  @@TR: I realize this use of the term 'expression' is a bit wonky as many  of the 'expressions' are actually statements, but I haven't thought of  a better name yet.  Suggestions? ``` |

|  |  |  |
| --- | --- | --- |
| |  |  | | --- | --- | | configureParser(self) |  |  Is called by the Compiler instance after the parser has had a settingsManager assigned with self.setSettingsManager() Overrides: \_LowLevelParser.configureParser *(inherited documentation)* |

|  |  |  |
| --- | --- | --- |
| |  |  | | --- | --- | | eatBreakPoint(self) |  |   Tells the parser to stop parsing at this point and completely ignore everything else. This is a debugging tool. |

  


|  |  |  |  |
| --- | --- | --- | --- |
| |  |  | | --- | --- | | Class Variable Details | [hide private] | | |

|  |  |
| --- | --- |
| \_directiveHandlerNames   Value:  |  | | --- | | ``` {'from': 'addImportStatement', 'import': 'addImportStatement'} ``` | |

|  |  |
| --- | --- |
| \_simpleExprDirectives   Value:  |  | | --- | | ``` ['pass',  'continue',  'stop',  'return',  'yield',  'break',  'del',  'assert', ... ``` | |

|  |  |
| --- | --- |
| \_simpleIndentingDirectives   Value:  |  | | --- | | ``` ['else',  'elif',  'for',  'while',  'repeat',  'unless',  'try',  'except', ... ``` | |

  


| Trees | Indices | Help | | MScanner | | --- | |
| --- | --- | --- | --- | --- |

|  |  |
| --- | --- |
| Generated by Epydoc 3.0beta1 on Fri Nov 23 09:13:21 2007 | http://epydoc.sourceforge.net |
