# Supplementary material for: MScanner: a classifier for retrieving Medline citations
Source: BMC Bioinformatics. 2008 Feb 19;9:108. doi: 10.1186/1471-2105-9-108 (PMC2263023; doi:10.1186/1471-2105-9-108)
Supplement: Additional file 3 — Source code for MScanner. mscanner-20071123.zip is a ZIP archive containing the Python 2.5 source code for MScanner, licensed under the GNU General Public License. It also contains API documentation in HTML format. Updated versions will be made available at . [file 1471-2105-9-108-S3.zip › mscanner/help/api/Cheetah.Template.TemplatePreprocessor-class.html]

xml version="1.0" encoding="ascii"?


Cheetah.Template.TemplatePreprocessor


| Trees | Indices | Help | | MScanner | | --- | |
| --- | --- | --- | --- | --- |

|  |  |  |  |
| --- | --- | --- | --- |
| Cheetah :: Template :: TemplatePreprocessor :: Class TemplatePreprocessor | |  | | --- | | [hide private] | | [frames] | no frames] | |

# Class TemplatePreprocessor

  
  

This is used with the preprocessors argument to
Template.compile().

See the docstring for Template.compile

\*\* Preprocessors are an advanced topic \*\*  
  


|  |  |  |  |
| --- | --- | --- | --- |
| |  |  | | --- | --- | | Instance Methods | [hide private] | | |
|  | |  |  | | --- | --- | | \_\_init\_\_(self, settings) |  | |
|  | |  |  | | --- | --- | | preprocess(self, source, file)  Create an intermediate template and return the source code it outputs |  | |

| Trees | Indices | Help | | MScanner | | --- | |
| --- | --- | --- | --- | --- |

|  |  |
| --- | --- |
| Generated by Epydoc 3.0beta1 on Fri Nov 23 09:13:21 2007 | http://epydoc.sourceforge.net |
