# Supplementary material for: MScanner: a classifier for retrieving Medline citations
Source: BMC Bioinformatics. 2008 Feb 19;9:108. doi: 10.1186/1471-2105-9-108 (PMC2263023; doi:10.1186/1471-2105-9-108)
Supplement: Additional file 3 — Source code for MScanner. mscanner-20071123.zip is a ZIP archive containing the Python 2.5 source code for MScanner, licensed under the GNU General Public License. It also contains API documentation in HTML format. Updated versions will be made available at . [file 1471-2105-9-108-S3.zip › mscanner/help/api/class-tree.html]

xml version="1.0" encoding="ascii"?


Class Hierarchy


| Trees | Indices | Help | | MScanner | | --- | |
| --- | --- | --- | --- | --- |

|  |  |  |  |
| --- | --- | --- | --- |
|  | |  | | --- | | [hide private] | | [frames] | no frames] | |

**[ Module Hierarchy
| Class Hierarchy ]**  

# Class Hierarchy

- **Cheetah.CacheRegion.CacheItem**:
  *A CacheItem is a container storing:...*
- **Cheetah.CacheRegion.CacheRegion**:
  *A `CacheRegion` stores some `CacheItem` instances.*
- **Cheetah.Compiler.GenUtils**:
  *An abstract baseclass for the Compiler classes that provides
  methods that perform generic utility functions or generate pieces
  of output code from information passed in by the Parser
  baseclass.*
  - **Cheetah.Compiler.ModuleCompiler**
  - **Cheetah.Compiler.ClassCompiler**
    - **Cheetah.Compiler.AutoClassCompiler**
  - **Cheetah.Compiler.MethodCompiler**
    - **Cheetah.Compiler.AutoMethodCompiler**
- **Cheetah.SettingsManager.\_SettingsCollector**:
  *An abstract base class that provides the methods SettingsManager uses to
  collect settings from config files and SettingsContainers.*
  - **Cheetah.SettingsManager.SettingsManager**:
    *A mixin class that provides facilities for managing application
    settings.*
    - **Cheetah.Compiler.ModuleCompiler**
- **Cheetah.SourceReader.SourceReader**
  - **Cheetah.Parser.\_LowLevelParser**:
    *This class implements the methods to match or extract ('get\*')
    the basic elements of Cheetah's grammar.*
    - **Cheetah.Parser.\_HighLevelParser**:
      *This class is a StateMachine for parsing Cheetah source and
      sending state dependent code generation commands to
      Cheetah.Compiler.Compiler.*
- **Cheetah.Template.TemplatePreprocessor**:
  *This is used with the preprocessors argument to
  Template.compile().*
- **ConfigParser.RawConfigParser**
  - **ConfigParser.ConfigParser**
    - **Cheetah.SettingsManager.ConfigParserCaseSensitive**:
      *A case sensitive version of the standard Python
      ConfigParser.*
- **UserDict.DictMixin**
  - **mscanner.medline.Shelf.Shelf**:
    *A shelf built upon a bsddb DB object.*
- **mscanner.core.Plotter.Plotter**:
  *Implements the plots used in MScanner*
  - **mscanner.core.Plotter.DensityPlotter**:
    *Adds plotting of estimated Probability Density Functions for
    article and feature scores.*
- **mscanner.core.QueryManager.QueryManager**:
  *Class for performing a single query*
- **mscanner.core.Validator.CrossValidator**:
  *Cross-validated calculation of article scores.*
  - **mscanner.core.Validator.LeaveOutValidator**:
    *Instead of N-fold cross validation, this class performs leave
    out one validation in which all but one of the citations is used to
    train the feature scores, which are then used to calculate the
    score of the left out document.*
- **mscanner.core.metrics.PerformanceMetrics**:
  *Performance metrics derived from a particular confusion
  matrix.*
- **mscanner.core.metrics.PerformanceRange**:
  *Given a threshold, find the minimum and maximum for the
  precision, recall across the validation folds.*
- **mscanner.core.metrics.PerformanceVectors**:
  *Contains vectors of performance metrics at all possible
  threshold*
- **mscanner.core.metrics.PredictedMetrics**:
  *Predict the performance metrics vectors for query results
  knowing only the true and false positive rates in testing.*
- **mscanner.fastscores.FeatureCounter.FeatureCounter**:
  *Class for calculating feature counts in a subset of Medline.*
- **mscanner.fastscores.ScoreCalculator.ScoreCalculator**:
  *Different methods for calculating the scores of all documents in
  the database.*
- **mscanner.htdocs.controller.FrontPage**:
  *Front page of the site*
- **mscanner.htdocs.forms.Form**:
  *Programmatically construct a form*
- **mscanner.htdocs.forms.Validator**:
  *Generic validator to pass to an Input or Form constructor.*
  - **mscanner.htdocs.forms.RegexValidator**:
    *Tests that the value matches a particular regular expression*
- **mscanner.htdocs.queue.QueueStatus**:
  *Describes the current state of the queue*
- **mscanner.htdocs.templates.contact\_logic.ContactPage**:
  *Form to contact the webmaster*
- **mscanner.htdocs.templates.output\_logic.OutputPage**:
  *Page linking to outputs*
- **mscanner.htdocs.templates.query\_logic.QueryPage**:
  *Submission form for queries or validation*
- **mscanner.htdocs.templates.status\_logic.StatusPage**:
  *Lists the current status of MScanner and a given task.*
- **mscanner.htdocs.testing.FormPage**:
  *Form testing page, on http://localhost:8080/form*
- **mscanner.htdocs.testing.HelloPage**:
  *Simple page, e.g.*
- **mscanner.medline.Article.Article**:
  *Database record for a Medline citation.*
- **mscanner.medline.Databases.Databases**:
  *The main interface to Medline used by the rest of the
  program.*
- **mscanner.medline.FeatureDatabase.FeatureDatabase**:
  *Database for which PubMed ID is the key and array of Feature IDs
  are values*
- **mscanner.medline.FeatureMapping.FeatureMapping**:
  *Persistent mapping between string features and feature IDs*
- **mscanner.medline.FeatureStream.FeatureStream**:
  *Class for reading/writing a binary stream of Medline records,
  consisting of PubMed ID, record completion date and a vector of
  Feature IDs for features present in the record.*
- **mscanner.medline.MedlineCache.MedlineCache**:
  *Class for updating the Article DB, FeatureMapping,
  FeatureDatabase, FeatureStream, PMID list, and FileTracker.*
- **object**:
  *The most base type*
  - **Cheetah.Servlet.BaseServlet**
    - **Cheetah.Servlet.Servlet**:
      *This class is an abstract baseclass for
      Cheetah.Template.Template.*
      - **Cheetah.Template.Template**:
        *This class provides a) methods used by templates at runtime and b)
        methods for compiling Cheetah source code into template classes.*
        - **mscanner.htdocs.templates.page.page**
          - **mscanner.htdocs.templates.status.status**
          - **mscanner.htdocs.templates.contact.contact**
          - **mscanner.htdocs.templates.query.query**
          - **mscanner.htdocs.templates.front.front**
          - **mscanner.htdocs.templates.output.output**
  - **set**:
    *set(iterable) --> set object*
    - **mscanner.medline.FileTracker.FileTracker**:
      *A persistent set for tracking of processed files.*
  - **file**:
    *file(name[, mode[, buffering]]) -> file object*
    - **mscanner.core.iofuncs.FileTransaction**:
      *Transaction for Cheetah templates to output direct-to-file.*
  - **mscanner.htdocs.forms.Input**:
    *Represents input widgets in the form*
    - **mscanner.htdocs.forms.Password**:
      *Widget for a password input*
    - **mscanner.htdocs.forms.Checkbox**:
      *Widget for a checkbox input*
    - **mscanner.htdocs.forms.File**:
      *Widget for a file input*
    - **mscanner.htdocs.forms.Dropdown**:
      *Widget for <select> dropdown box*
    - **mscanner.htdocs.forms.Textbox**:
      *Widget for a text input*
    - **mscanner.htdocs.forms.Textarea**:
      *Widget for a <textarea>*
    - **mscanner.htdocs.forms.Button**:
      *Widget for a button.*
    - **mscanner.htdocs.forms.Radio**:
      *Widget for a set of radio buttons*
    - **mscanner.htdocs.forms.Hidden**:
      *Widget for a hidden input*
  - **mscanner.core.ValidationManager.ValidationBase**:
    *Base class for all validation operations.*
    - **mscanner.core.ValidationManager.CrossValidation**:
      *Carries out N-fold cross validation.*
  - **mscanner.core.FeatureScores.FeatureScores**:
    *Feature score calculation and saving, with choice of calculation
    method, and methods to exclude certain kinds of features.*
  - **dict**:
    *dict() -> new empty dictionary.*
    - **mscanner.core.Storage.Storage**:
      *Dictionary supporting d.foo attribute access to keys.*
      - **mscanner.core.Storage.RCStorage**:
        *Dictionary with attribute access and auto-calling of stored
        functions.*
  - **Cheetah.CacheStore.AbstractCacheStore**
    - **Cheetah.CacheStore.MemoryCacheStore**
  - **exceptions.BaseException**:
    *Common base class for all exceptions*
    - **exceptions.Exception**:
      *Common base class for all non-exit exceptions.*
      - **exceptions.StandardError**:
        *Base class for all standard Python exceptions that do not
        represent interpreter exiting.*
        - **exceptions.ValueError**:
          *Inappropriate argument value (of correct type).*
          - **Cheetah.Utils.WebInputMixin.NonNumericInputError**

| Trees | Indices | Help | | MScanner | | --- | |
| --- | --- | --- | --- | --- |

|  |  |
| --- | --- |
| Generated by Epydoc 3.0beta1 on Fri Nov 23 09:13:20 2007 | http://epydoc.sourceforge.net |
