# Supplementary material for: MScanner: a classifier for retrieving Medline citations
Source: BMC Bioinformatics. 2008 Feb 19;9:108. doi: 10.1186/1471-2105-9-108 (PMC2263023; doi:10.1186/1471-2105-9-108)
Supplement: Additional file 3 — Source code for MScanner. mscanner-20071123.zip is a ZIP archive containing the Python 2.5 source code for MScanner, licensed under the GNU General Public License. It also contains API documentation in HTML format. Updated versions will be made available at . [file 1471-2105-9-108-S3.zip › mscanner/help/api/identifier-index.html]

xml version="1.0" encoding="ascii"?


Identifier Index


| Trees | Indices | Help | | MScanner | | --- | |
| --- | --- | --- | --- | --- |

|  |  |  |  |
| --- | --- | --- | --- |
|  | |  | | --- | | [hide private] | | [frames] | no frames] | |

**[
Identifiers
| Deprecations
]**  

|  |  |
| --- | --- |
| Identifier Index | [ A B C D E F G H I J K L M N O P Q R S T U V W X Y Z \_ ] |

|  |  |  |  |  |  |  |  |  |  |  |  |  |  |
| --- | --- | --- | --- | --- | --- | --- | --- | --- | --- | --- | --- | --- | --- |
| A | |  |  |  | | --- | --- | --- | | add()  (in FileTracker) | addatts()  (in Input) | attrget()  (in mscanner.htdocs.forms) | | add\_article()  (in FeatureMapping) | Article  (in mscanner.medline) | AutoClassCompiler | | add\_articles()  (in MedlineCache) | Article  (in mscanner.medline.Article) | AutoMethodCompiler | | add\_directory()  (in MedlineCache) | article\_list  (in Databases) |  | |
 B | |  |  |  | | --- | --- | --- | | base  (in page) | body()  (in page) | buttonpressed()  (in mscanner.htdocs.forms) | | bincount()  (in Plotter) | Button  (in mscanner.htdocs.forms) |  | | C | |  |  |  | | --- | --- | --- | | c\_counts()  (in FeatureCounter) | compare\_trec\_genomics()  (in mscanner.scripts.validate) | contents()  (in query) | | CacheItem | Complex0  (in mscanner.scripts.latexplots) | contents()  (in status) | | CacheRegion | Complex16  (in mscanner.scripts.latexplots) | contents\_outer()  (in page) | | Checkbox  (in mscanner.htdocs.forms) | Complex8  (in mscanner.scripts.latexplots) | controller  (in mscanner.htdocs) | | checkbox\_validator  (in mscanner.htdocs.forms) | ConfigParserCaseSensitive | core  (in mscanner) | | CitationTable  (in mscanner.core) | contact  (in mscanner.htdocs.templates) | counter\_path  (in FeatureCounter) | | CitationTable()  (in mscanner.core.CitationTable) | contact  (in mscanner.htdocs.templates.contact) | create\_dbenv()  (in MedlineCache) | | close()  (in Databases) | contact\_logic  (in mscanner.htdocs.templates) | CrossValidation  (in mscanner.core.ValidationManager) | | close()  (in FeatureDatabase) | ContactForm  (in mscanner.htdocs.templates.contact\_logic) | CrossValidator  (in mscanner.core.Validator) | | close()  (in FeatureStream) | ContactPage  (in mscanner.htdocs.templates.contact\_logic) | cscore\_dll()  (in ScoreCalculator) | | close()  (in Shelf) | contents()  (in contact) | cscore\_pipe()  (in ScoreCalculator) | | close\_logfile()  (in mscanner.core.iofuncs) | contents()  (in front) | custom\_show()  (in mscanner.scripts.latexplots) | | compare\_iedb\_query()  (in mscanner.scripts.retrievaltest) | contents()  (in output) |  | | compare\_iedb\_valid()  (in mscanner.scripts.validate) | contents()  (in page) |  | | D | |  |  |  | | --- | --- | --- | | d  (in Form) | delcode\_validator  (in mscanner.htdocs.templates.query\_logic) | do\_query()  (in mscanner.scripts.query) | | Databases  (in mscanner.medline) | delete\_output()  (in mscanner.htdocs.queue) | do\_subdirplots()  (in mscanner.scripts.latexplots) | | Databases  (in mscanner.medline.Databases) | delitem()  (in FeatureDatabase) | do\_testplots()  (in mscanner.scripts.latexplots) | | dataset\_map  (in mscanner.scripts.query) | DensityPlotter  (in mscanner.core.Plotter) | doctype()  (in page) | | dataset\_map  (in mscanner.scripts.validate) | descriptor\_keys  (in mscanner.htdocs.queue) | DONE  (in QueueStatus) | | dataset\_validator  (in mscanner.htdocs.templates.query\_logic) | divide\_safe  (in mscanner.scripts.latexplots) | Dropdown  (in mscanner.htdocs.forms) | | Date2Integer()  (in mscanner.medline.FeatureStream) | dll\_path  (in ScoreCalculator) | dump()  (in FeatureMapping) | | date\_is\_valid()  (in mscanner.htdocs.templates.query\_logic) | do\_iedb()  (in mscanner.scripts.latexplots) | dump()  (in FileTracker) | | dbhelper  (in mscanner.scripts) | do\_publication()  (in mscanner.scripts.latexplots) |  | | E | |  |  |  | | --- | --- | --- | | errors  (in Form) | extraheaders()  (in page) | extraheaders()  (in status) | | executable\_path  (in ScoreCalculator) | extraheaders()  (in query) |  | | F | |  |  |  | | --- | --- | --- | | fastscores  (in mscanner) | FeatureStream  (in mscanner.medline.FeatureStream) | footer()  (in page) | | FeatureCounter  (in mscanner.fastscores) | File  (in mscanner.htdocs.forms) | footer\_text()  (in page) | | FeatureCounter  (in mscanner.fastscores.FeatureCounter) | FileTracker  (in mscanner.medline) | Form  (in mscanner.htdocs.forms) | | FeatureCounts()  (in mscanner.core.FeatureScores) | FileTracker  (in mscanner.medline.FileTracker) | form\_defaults  (in mscanner.htdocs.templates.query\_logic) | | FeatureDatabase  (in mscanner.medline) | FileTransaction  (in mscanner.core.iofuncs) | form\_row()  (in query) | | FeatureDatabase  (in mscanner.medline.FeatureDatabase) | fill()  (in Form) | form\_template  (in mscanner.htdocs.testing) | | FeatureMapping  (in mscanner.medline) | Float0  (in mscanner.scripts.latexplots) | FormPage  (in mscanner.htdocs.testing) | | FeatureMapping  (in mscanner.medline.FeatureMapping) | Float16  (in mscanner.scripts.latexplots) | forms  (in mscanner.htdocs) | | FeatureScores  (in mscanner.core) | Float8  (in mscanner.scripts.latexplots) | front  (in mscanner.htdocs.templates) | | FeatureScores  (in mscanner.core.FeatureScores) | floor\_divide  (in mscanner.scripts.latexplots) | front  (in mscanner.htdocs.templates.front) | | FeatureStream  (in mscanner.medline) | fmt\_stats()  (in PerformanceRange) | FrontPage  (in mscanner.htdocs.controller) | | G | |  |  |  | | --- | --- | --- | | gaussian\_kernel\_pdf()  (in DensityPlotter) | GET()  (in StatusPage) | getitem()  (in FeatureDatabase) | | GET()  (in FrontPage) | GET()  (in FormPage) | getvalue()  (in FileTransaction) | | GET()  (in ContactPage) | GET()  (in HelloPage) | gplot()  (in mscanner.scripts.latexplots) | | GET()  (in OutputPage) | get\_best\_tfidfs()  (in FeatureScores) |  | | GET()  (in QueryPage) | get\_type\_mask()  (in FeatureMapping) |  | | H | |  |  |  | | --- | --- | --- | | head()  (in page) | help\_delcode()  (in query) | help\_prevalence()  (in query) | | header()  (in page) | help\_limit()  (in query) | Hidden  (in mscanner.htdocs.forms) | | header\_text()  (in page) | help\_mindate()  (in query) | htdocs  (in mscanner) | | headertitle()  (in front) | help\_minscore()  (in query) | html()  (in page) | | HelloPage  (in mscanner.htdocs.testing) | help\_numnegs()  (in query) |  | | help\_dataset()  (in query) | help\_positives()  (in query) |  | | I | |  |  |  | | --- | --- | --- | | index\_for()  (in PerformanceVectors) | interactive  (in mscanner.scripts.latexplots) | items()  (in Shelf) | | Input  (in mscanner.htdocs.forms) | invert  (in mscanner.scripts.latexplots) | iteritems()  (in FeatureDatabase) | | Int0  (in mscanner.scripts.latexplots) | iofuncs  (in mscanner.core) | iteritems()  (in Shelf) | | Integer2Date()  (in mscanner.medline.FeatureStream) | ischecked()  (in mscanner.htdocs.forms) |  | | K | |  |  |  | | --- | --- | --- | | keys()  (in FeatureDatabase) | keys()  (in Shelf) |  | |  |  |  | | L | |  |  |  | | --- | --- | --- | | latexplots  (in mscanner.scripts) | listkeys()  (in mscanner.scripts.dbhelper) | load\_stats()  (in mscanner.scripts.latexplots) | | LeaveOutValidator  (in mscanner.core.Validator) | load()  (in FeatureMapping) | logit()  (in mscanner.htdocs.queue) | | left\_shift  (in mscanner.scripts.latexplots) | load\_articles()  (in mscanner.medline.Databases) |  | | M | |  |  |  | | --- | --- | --- | | mainloop()  (in mscanner.htdocs.queue) | MedlineCache  (in mscanner.medline) | metrics\_for()  (in PerformanceVectors) | | make\_partitions()  (in CrossValidator) | MedlineCache  (in mscanner.medline.MedlineCache) | ModuleCompiler | | mask\_nonpositives()  (in FeatureScores) | MemoryCacheStore | mscanner\_dir  (in mscanner.scripts.latexplots) | | matrix\_for()  (in PerformanceVectors) | MethodCompiler |  | | medline  (in mscanner) | metrics  (in mscanner.core) |  | | N | |  |  |  | | --- | --- | --- | | no\_valid\_pmids\_page()  (in mscanner.core.iofuncs) | notnull  (in mscanner.htdocs.forms) |  | | NonNumericInputError | npoints  (in mscanner.scripts.latexplots) |  | | O | |  |  |  | | --- | --- | --- | | open()  (in mscanner.medline.Shelf) | output  (in mscanner.htdocs.templates) | OutputForm  (in mscanner.htdocs.templates.output\_logic) | | open\_logfile()  (in mscanner.core.iofuncs) | output  (in mscanner.htdocs.templates.output) | OutputPage  (in mscanner.htdocs.templates.output\_logic) | | outdir  (in mscanner.scripts.latexplots) | output\_logic  (in mscanner.htdocs.templates) |  | | P | |  |  |  | | --- | --- | --- | | page  (in mscanner.htdocs.templates) | plot\_feature\_histogram()  (in Plotter) | Plotter  (in mscanner.core.Plotter) | | page  (in mscanner.htdocs.templates.page) | plot\_fmeasure()  (in Plotter) | pmid\_dates()  (in mscanner.scripts.dbhelper) | | parse\_date()  (in mscanner.htdocs.templates.query\_logic) | plot\_precision()  (in Plotter) | populate\_test\_queue()  (in mscanner.htdocs.queue) | | parse\_medline\_xml()  (in Article) | plot\_precision()  (in mscanner.scripts.latexplots) | position()  (in QueueStatus) | | parse\_pmids()  (in mscanner.htdocs.templates.query\_logic) | plot\_predictions()  (in Plotter) | POST()  (in ContactPage) | | parsebool()  (in mscanner.htdocs.queue) | plot\_rank\_performance()  (in mscanner.scripts.retrievaltest) | POST()  (in OutputPage) | | Password  (in mscanner.htdocs.forms) | plot\_roc()  (in Plotter) | POST()  (in QueryPage) | | PerformanceMetrics  (in mscanner.core.metrics) | plot\_roc()  (in mscanner.scripts.latexplots) | POST()  (in FormPage) | | PerformanceRange  (in mscanner.core.metrics) | plot\_score\_density()  (in DensityPlotter) | PredictedMetrics  (in mscanner.core.metrics) | | PerformanceVectors  (in mscanner.core.metrics) | plot\_score\_density()  (in mscanner.scripts.latexplots) | print\_page()  (in OutputPage) | | pformat()  (in mscanner.htdocs.testing) | plot\_score\_histogram()  (in Plotter) | py\_counts()  (in FeatureCounter) | | plot\_featscore\_histogram()  (in mscanner.scripts.latexplots) | plot\_score\_histogram()  (in mscanner.scripts.latexplots) | pyscore()  (in ScoreCalculator) | | plot\_feature\_density()  (in DensityPlotter) | Plotter  (in mscanner.core) |  | | Q | |  |  |  | | --- | --- | --- | | query  (in mscanner.htdocs.templates) | query\_logic  (in mscanner.htdocs.templates) | QueryPage  (in mscanner.htdocs.templates.query\_logic) | | query  (in mscanner.htdocs.templates.query) | QueryForm  (in mscanner.htdocs.templates.query\_logic) | queue  (in mscanner.htdocs) | | query  (in mscanner.scripts) | QueryManager  (in mscanner.core) | QueueStatus  (in mscanner.htdocs.queue) | | query()  (in QueryManager) | QueryManager  (in mscanner.core.QueryManager) |  | | R | |  |  |  | | --- | --- | --- | | Radio  (in mscanner.htdocs.forms) | RegexValidator  (in mscanner.htdocs.forms) | render()  (in Textarea) | | RCStorage  (in mscanner.core.Storage) | remainder  (in mscanner.scripts.latexplots) | render()  (in Textbox) | | read\_descriptor()  (in mscanner.htdocs.queue) | render()  (in Button) | render\_errors()  (in Form) | | read\_featscores()  (in mscanner.scripts.latexplots) | render()  (in Checkbox) | renderlabel()  (in Input) | | read\_pmids()  (in mscanner.core.iofuncs) | render()  (in Dropdown) | renderlabel()  (in Radio) | | read\_pmids\_array()  (in mscanner.core.iofuncs) | render()  (in File) | report\_predicted()  (in CrossValidation) | | read\_pmids\_careful()  (in mscanner.core.iofuncs) | render()  (in Form) | report\_validation()  (in CrossValidation) | | read\_scores()  (in mscanner.core.iofuncs) | render()  (in Hidden) | respond()  (in page) | | read\_scores\_array()  (in mscanner.core.iofuncs) | render()  (in Input) | retrievaltest  (in mscanner.scripts) | | regen\_article\_list()  (in mscanner.scripts.dbhelper) | render()  (in Password) | right\_shift  (in mscanner.scripts.latexplots) | | regen\_stream()  (in mscanner.scripts.dbhelper) | render()  (in Radio) | RUNNING  (in QueueStatus) | | S | |  |  |  | | --- | --- | --- | | score()  (in ScoreCalculator) | setitem()  (in FeatureDatabase) | status  (in mscanner.htdocs.templates.status) | | ScoreCalculator  (in mscanner.fastscores) | Shelf  (in mscanner.medline) | status\_logic  (in mscanner.htdocs.templates) | | ScoreCalculator  (in mscanner.fastscores.ScoreCalculator) | Shelf  (in mscanner.medline.Shelf) | statusblock()  (in page) | | scores\_bayes()  (in FeatureScores) | smooth()  (in mscanner.scripts.latexplots) | StatusForm  (in mscanner.htdocs.templates.status\_logic) | | scores\_noabsence()  (in FeatureScores) | source\_dir  (in mscanner.scripts.latexplots) | StatusPage  (in mscanner.htdocs.templates.status\_logic) | | scores\_of()  (in FeatureScores) | SplitValidation()  (in mscanner.core.ValidationManager) | stdheaders()  (in page) | | scores\_rubin()  (in FeatureScores) | start\_logger()  (in mscanner.core.iofuncs) | Storage  (in mscanner.core) | | scripts  (in mscanner) | stats  (in FeatureScores) | Storage  (in mscanner.core.Storage) | | select\_lines()  (in mscanner.scripts.dbhelper) | stats\_for()  (in PerformanceRange) |  | | set\_txn()  (in Shelf) | status  (in mscanner.htdocs.templates) |  | | T | |  |  |  | | --- | --- | --- | | task\_does\_not\_exist()  (in mscanner.htdocs.templates.query\_logic) | Textbox  (in mscanner.htdocs.forms) | title()  (in query) | | task\_exists()  (in mscanner.htdocs.templates.query\_logic) | tfidf  (in FeatureScores) | title()  (in status) | | TemplatePreprocessor | threshold\_maximising()  (in PerformanceVectors) | topmenu()  (in page) | | templates  (in mscanner.htdocs) | title()  (in contact) | toprocess()  (in FileTracker) | | TestForm  (in mscanner.htdocs.testing) | title()  (in front) | true\_divide  (in mscanner.scripts.latexplots) | | testing  (in mscanner.htdocs) | title()  (in output) | truepos\_vs\_rank()  (in mscanner.scripts.retrievaltest) | | Textarea  (in mscanner.htdocs.forms) | title()  (in page) |  | | U | |  |  |  | | --- | --- | --- | | update  (in mscanner.scripts) | update\_mscanner()  (in mscanner.scripts.update) | urls  (in mscanner.htdocs.testing) | | update()  (in FeatureScores) | urls  (in mscanner.htdocs.controller) |  | | V | |  |  |  | | --- | --- | --- | | valid()  (in RegexValidator) | validate()  (in Input) | ValidationManager  (in mscanner.core) | | valid()  (in Validator) | validate()  (in mscanner.scripts.validate) | Validator  (in mscanner.core) | | validate  (in mscanner.scripts) | validates()  (in Form) | Validator  (in mscanner.htdocs.forms) | | validate()  (in CrossValidator) | validation()  (in CrossValidation) | values()  (in Shelf) | | validate()  (in LeaveOutValidator) | ValidationBase  (in mscanner.core.ValidationManager) |  | | W | |  |  |  | | --- | --- | --- | | WAITING  (in QueueStatus) | write\_lines()  (in mscanner.core.iofuncs) | writeBody()  (in output) | | wintitle()  (in page) | write\_pmids()  (in mscanner.core.iofuncs) | writeBody()  (in query) | | write()  (in FeatureStream) | write\_report()  (in QueryManager) | writeBody()  (in status) | | write\_citations()  (in mscanner.core.CitationTable) | write\_scores()  (in mscanner.core.iofuncs) | writeln()  (in FileTransaction) | | write\_csv()  (in FeatureScores) | writeBody()  (in contact) |  | | write\_descriptor()  (in mscanner.htdocs.queue) | writeBody()  (in front) |  | | \_ | |  |  |  | | --- | --- | --- | | \_\_call\_\_()  (in FileTransaction) | \_\_init\_\_()  (in ValidationBase) | \_CHEETAH\_genTimestamp  (in output) | | \_\_call\_\_()  (in Form) | \_\_init\_\_()  (in CrossValidator) | \_CHEETAH\_genTimestamp  (in page) | | \_\_CHEETAH\_docstring\_\_  (in mscanner.htdocs.templates.contact) | \_\_init\_\_()  (in FileTransaction) | \_CHEETAH\_genTimestamp  (in query) | | \_\_CHEETAH\_docstring\_\_  (in mscanner.htdocs.templates.front) | \_\_init\_\_()  (in PerformanceMetrics) | \_CHEETAH\_genTimestamp  (in status) | | \_\_CHEETAH\_docstring\_\_  (in mscanner.htdocs.templates.output) | \_\_init\_\_()  (in PerformanceRange) | \_CHEETAH\_src  (in contact) | | \_\_CHEETAH\_docstring\_\_  (in mscanner.htdocs.templates.page) | \_\_init\_\_()  (in PerformanceVectors) | \_CHEETAH\_src  (in front) | | \_\_CHEETAH\_docstring\_\_  (in mscanner.htdocs.templates.query) | \_\_init\_\_()  (in PredictedMetrics) | \_CHEETAH\_src  (in output) | | \_\_CHEETAH\_docstring\_\_  (in mscanner.htdocs.templates.status) | \_\_init\_\_()  (in FeatureCounter) | \_CHEETAH\_src  (in page) | | \_\_CHEETAH\_genTime\_\_  (in mscanner.htdocs.templates.contact) | \_\_init\_\_()  (in ScoreCalculator) | \_CHEETAH\_src  (in query) | | \_\_CHEETAH\_genTime\_\_  (in mscanner.htdocs.templates.front) | \_\_init\_\_()  (in Dropdown) | \_CHEETAH\_src  (in status) | | \_\_CHEETAH\_genTime\_\_  (in mscanner.htdocs.templates.output) | \_\_init\_\_()  (in Form) | \_CHEETAH\_srcLastModified  (in contact) | | \_\_CHEETAH\_genTime\_\_  (in mscanner.htdocs.templates.page) | \_\_init\_\_()  (in Input) | \_CHEETAH\_srcLastModified  (in front) | | \_\_CHEETAH\_genTime\_\_  (in mscanner.htdocs.templates.query) | \_\_init\_\_()  (in Radio) | \_CHEETAH\_srcLastModified  (in output) | | \_\_CHEETAH\_genTime\_\_  (in mscanner.htdocs.templates.status) | \_\_init\_\_()  (in RegexValidator) | \_CHEETAH\_srcLastModified  (in page) | | \_\_CHEETAH\_genTimestamp\_\_  (in mscanner.htdocs.templates.contact) | \_\_init\_\_()  (in Validator) | \_CHEETAH\_srcLastModified  (in query) | | \_\_CHEETAH\_genTimestamp\_\_  (in mscanner.htdocs.templates.front) | \_\_init\_\_()  (in QueueStatus) | \_CHEETAH\_srcLastModified  (in status) | | \_\_CHEETAH\_genTimestamp\_\_  (in mscanner.htdocs.templates.output) | \_\_init\_\_()  (in contact) | \_CHEETAH\_version  (in contact) | | \_\_CHEETAH\_genTimestamp\_\_  (in mscanner.htdocs.templates.page) | \_\_init\_\_()  (in front) | \_CHEETAH\_version  (in front) | | \_\_CHEETAH\_genTimestamp\_\_  (in mscanner.htdocs.templates.query) | \_\_init\_\_()  (in output) | \_CHEETAH\_version  (in output) | | \_\_CHEETAH\_genTimestamp\_\_  (in mscanner.htdocs.templates.status) | \_\_init\_\_()  (in page) | \_CHEETAH\_version  (in page) | | \_\_CHEETAH\_src\_\_  (in mscanner.htdocs.templates.contact) | \_\_init\_\_()  (in query) | \_CHEETAH\_version  (in query) | | \_\_CHEETAH\_src\_\_  (in mscanner.htdocs.templates.front) | \_\_init\_\_()  (in status) | \_CHEETAH\_version  (in status) | | \_\_CHEETAH\_src\_\_  (in mscanner.htdocs.templates.output) | \_\_init\_\_()  (in Article) | \_CHEETAH\_versionTuple  (in contact) | | \_\_CHEETAH\_src\_\_  (in mscanner.htdocs.templates.page) | \_\_init\_\_()  (in Databases) | \_CHEETAH\_versionTuple  (in front) | | \_\_CHEETAH\_src\_\_  (in mscanner.htdocs.templates.query) | \_\_init\_\_()  (in FeatureDatabase) | \_CHEETAH\_versionTuple  (in output) | | \_\_CHEETAH\_src\_\_  (in mscanner.htdocs.templates.status) | \_\_init\_\_()  (in FeatureMapping) | \_CHEETAH\_versionTuple  (in page) | | \_\_CHEETAH\_srcLastModified\_\_  (in mscanner.htdocs.templates.contact) | \_\_init\_\_()  (in FeatureStream) | \_CHEETAH\_versionTuple  (in query) | | \_\_CHEETAH\_srcLastModified\_\_  (in mscanner.htdocs.templates.front) | \_\_init\_\_()  (in FileTracker) | \_CHEETAH\_versionTuple  (in status) | | \_\_CHEETAH\_srcLastModified\_\_  (in mscanner.htdocs.templates.output) | \_\_init\_\_()  (in MedlineCache) | \_confusion\_matrix()  (in PerformanceRange) | | \_\_CHEETAH\_srcLastModified\_\_  (in mscanner.htdocs.templates.page) | \_\_init\_\_()  (in Shelf) | \_confusion\_vectors()  (in PerformanceVectors) | | \_\_CHEETAH\_srcLastModified\_\_  (in mscanner.htdocs.templates.query) | \_\_iter\_\_()  (in FeatureDatabase) | \_crossvalid\_scores()  (in ValidationBase) | | \_\_CHEETAH\_srcLastModified\_\_  (in mscanner.htdocs.templates.status) | \_\_iter\_\_()  (in FeatureStream) | \_curve\_areas()  (in PerformanceVectors) | | \_\_CHEETAH\_version\_\_  (in mscanner.htdocs.templates.contact) | \_\_iter\_\_()  (in Shelf) | \_directiveHandlerNames  (in \_HighLevelParser) | | \_\_CHEETAH\_version\_\_  (in mscanner.htdocs.templates.front) | \_\_len\_\_()  (in FeatureScores) | \_get\_performance()  (in ValidationBase) | | \_\_CHEETAH\_version\_\_  (in mscanner.htdocs.templates.output) | \_\_len\_\_()  (in FeatureDatabase) | \_HighLevelParser | | \_\_CHEETAH\_version\_\_  (in mscanner.htdocs.templates.page) | \_\_len\_\_()  (in FeatureMapping) | \_init\_featinfo()  (in ValidationBase) | | \_\_CHEETAH\_version\_\_  (in mscanner.htdocs.templates.query) | \_\_len\_\_()  (in Shelf) | \_load\_donelist()  (in QueueStatus) | | \_\_CHEETAH\_version\_\_  (in mscanner.htdocs.templates.status) | \_\_repr\_\_()  (in Storage) | \_load\_input()  (in QueryManager) | | \_\_CHEETAH\_versionTuple\_\_  (in mscanner.htdocs.templates.contact) | \_\_repr\_\_()  (in Article) | \_load\_input()  (in CrossValidation) | | \_\_CHEETAH\_versionTuple\_\_  (in mscanner.htdocs.templates.front) | \_\_setattr\_\_()  (in Storage) | \_load\_maps()  (in QueueStatus) | | \_\_CHEETAH\_versionTuple\_\_  (in mscanner.htdocs.templates.output) | \_\_setitem\_\_()  (in FeatureDatabase) | \_load\_results()  (in QueryManager) | | \_\_CHEETAH\_versionTuple\_\_  (in mscanner.htdocs.templates.page) | \_\_setitem\_\_()  (in Shelf) | \_load\_tasklist()  (in QueueStatus) | | \_\_CHEETAH\_versionTuple\_\_  (in mscanner.htdocs.templates.query) | \_\_str\_\_()  (in RCStorage) | \_mainCheetahMethod\_for\_contact  (in contact) | | \_\_CHEETAH\_versionTuple\_\_  (in mscanner.htdocs.templates.status) | \_\_str\_\_()  (in Storage) | \_mainCheetahMethod\_for\_front  (in front) | | \_\_contains\_\_()  (in QueueStatus) | \_\_str\_\_()  (in page) | \_mainCheetahMethod\_for\_output  (in output) | | \_\_contains\_\_()  (in FeatureDatabase) | \_article\_features()  (in MedlineCache) | \_mainCheetahMethod\_for\_page  (in page) | | \_\_contains\_\_()  (in Shelf) | \_averaged\_precision()  (in PerformanceVectors) | \_mainCheetahMethod\_for\_query  (in query) | | \_\_deepcopy\_\_()  (in Validator) | \_breakeven()  (in PerformanceVectors) | \_mainCheetahMethod\_for\_status  (in status) | | \_\_del\_\_()  (in QueryManager) | \_calculate\_min\_max()  (in PerformanceRange) | \_make\_confusion\_vectors()  (in PerformanceRange) | | \_\_del\_\_()  (in ValidationBase) | \_CHEETAH\_\_instanceInitialized  (in contact) | \_make\_feature\_info()  (in QueryManager) | | \_\_del\_\_()  (in Shelf) | \_CHEETAH\_\_instanceInitialized  (in front) | \_make\_pseudovec()  (in FeatureScores) | | \_\_delattr\_\_()  (in Storage) | \_CHEETAH\_\_instanceInitialized  (in output) | \_make\_results()  (in QueryManager) | | \_\_delitem\_\_()  (in Shelf) | \_CHEETAH\_\_instanceInitialized  (in page) | \_mask\_scores()  (in FeatureScores) | | \_\_getattr\_\_()  (in RCStorage) | \_CHEETAH\_\_instanceInitialized  (in query) | \_mergescores()  (in PerformanceVectors) | | \_\_getattr\_\_()  (in Storage) | \_CHEETAH\_\_instanceInitialized  (in status) | \_random\_subset()  (in CrossValidation) | | \_\_getitem\_\_()  (in Form) | \_CHEETAH\_genTime  (in contact) | \_ratio\_vectors()  (in PerformanceVectors) | | \_\_getitem\_\_()  (in QueueStatus) | \_CHEETAH\_genTime  (in front) | \_roc\_error()  (in PerformanceVectors) | | \_\_getitem\_\_()  (in FeatureDatabase) | \_CHEETAH\_genTime  (in output) | \_save\_results()  (in QueryManager) | | \_\_getitem\_\_()  (in FeatureMapping) | \_CHEETAH\_genTime  (in page) | \_simpleExprDirectives  (in \_HighLevelParser) | | \_\_getitem\_\_()  (in Shelf) | \_CHEETAH\_genTime  (in query) | \_simpleIndentingDirectives  (in \_HighLevelParser) | | \_\_init\_\_()  (in FeatureScores) | \_CHEETAH\_genTime  (in status) | \_update\_featscores()  (in ValidationBase) | | \_\_init\_\_()  (in Plotter) | \_CHEETAH\_genTimestamp  (in contact) | \_validate()  (in Form) | | \_\_init\_\_()  (in QueryManager) | \_CHEETAH\_genTimestamp  (in front) | \_write\_report()  (in ValidationBase) | |

  

| Trees | Indices | Help | | MScanner | | --- | |
| --- | --- | --- | --- | --- |

|  |  |
| --- | --- |
| Generated by Epydoc 3.0beta1 on Fri Nov 23 09:13:20 2007 | http://epydoc.sourceforge.net |
