# Supplementary material for: MScanner: a classifier for retrieving Medline citations
Source: BMC Bioinformatics. 2008 Feb 19;9:108. doi: 10.1186/1471-2105-9-108 (PMC2263023; doi:10.1186/1471-2105-9-108)
Supplement: Additional file 3 — Source code for MScanner. mscanner-20071123.zip is a ZIP archive containing the Python 2.5 source code for MScanner, licensed under the GNU General Public License. It also contains API documentation in HTML format. Updated versions will be made available at . [file 1471-2105-9-108-S3.zip › mscanner/help/api/module-tree.html]

xml version="1.0" encoding="ascii"?


Module Hierarchy


| Trees | Indices | Help | | MScanner | | --- | |
| --- | --- | --- | --- | --- |

|  |  |  |  |
| --- | --- | --- | --- |
|  | |  | | --- | | [hide private] | | [frames] | no frames] | |

**[ Module Hierarchy
| Class Hierarchy ]**  

# Module Hierarchy

- **mscanner.core**: *The core MScanner modules*
  - **mscanner.core.CitationTable**: *Writes HTML pages with interactive citation tables*
  - **mscanner.core.FeatureScores**: *Calculates feature scores from occurrence counts*
  - **mscanner.core.Plotter**: *Plotting functions for all graphs produced in cross
    validation.*
  - **mscanner.core.QueryManager**: *Environment for performing query-based analyses.*
  - **mscanner.core.Storage**: *Dictionary subclasses supporting dotted access*
  - **mscanner.core.ValidationManager**: *Environment for performing cross-validation-based analyses*
  - **mscanner.core.Validator**: *Cross-validation and performance statistic calculation*
  - **mscanner.core.iofuncs**: *I/O functions - for reading and writing certain file
    formats.*
  - **mscanner.core.metrics**: *Calculates performance statistics given the scores of the
    positive and negative citations*
- **mscanner.fastscores**: *Utilities for rapidly calculating article scores and feature
  counts across a subset of Medline.*
  - **mscanner.fastscores.FeatureCounter**: *Calculates the number of occurrences of each feature for all
    articles in Medline between two dates.*
  - **mscanner.fastscores.ScoreCalculator**: *Calculates citation scores*
- **mscanner.htdocs**: *Package for running the MScanner web interface*
  - **mscanner.htdocs.controller**: *Controller for the MScanner web interface*
  - **mscanner.htdocs.forms**: *Programmatic form construction and validation*
  - **mscanner.htdocs.queue**: *Queueing facility for the web frontend*
  - **mscanner.htdocs.templates**
    - **mscanner.htdocs.templates.contact**
    - **mscanner.htdocs.templates.contact\_logic**: *web.py handler for the contact page*
    - **mscanner.htdocs.templates.front**
    - **mscanner.htdocs.templates.output**
    - **mscanner.htdocs.templates.output\_logic**: *web.py handler for the output listing page*
    - **mscanner.htdocs.templates.page**
    - **mscanner.htdocs.templates.query**
    - **mscanner.htdocs.templates.query\_logic**: *web.py handler for the query submission page*
    - **mscanner.htdocs.templates.status**
    - **mscanner.htdocs.templates.status\_logic**: *web.py handler for the status page*
  - **mscanner.htdocs.testing**: *Simple tests using the web.py framework*
- **mscanner.medline**: *Modules for creating, reading and updating the MScanner
  representation of Medline*
  - **mscanner.medline.Article**: *Provides the Article class*
  - **mscanner.medline.Databases**: *For consumers of the database, this opens FeatureDatabase, FeatureMapping and the article list*
  - **mscanner.medline.FeatureDatabase**: *Maps PubMed IDs to feature vectors*
  - **mscanner.medline.FeatureMapping**: *Provides a mapping between features and integer IDs*
  - **mscanner.medline.FeatureStream**: *A class for rapid iteration over the records in Medline.*
  - **mscanner.medline.FileTracker**: *A simple persistant set of file names*
  - **mscanner.medline.MedlineCache**: *For updating the databases of articles and features*
  - **mscanner.medline.Shelf**: *Persistent shelf backed by Berkeley DB*
- **mscanner.scripts**: *Executable scripts, some of which perform utility functions, and
  others which carry out analyses for the MScanner paper results.*
  - **mscanner.scripts.dbhelper**: *Utility functions for working with files containing lists of
    PubMed IDs, and Berkeley DBs containing pickled Articles, and for
    regenerating the FeatureStream and article list.*
  - **mscanner.scripts.latexplots**: *Draws publication-quality plots for use in the paper*
  - **mscanner.scripts.query**: *Performs queries using datasets from the MScanner paper*
  - **mscanner.scripts.retrievaltest**: *Performs retrieval test analysis, where a subset of the input is
    used to query, and the results are compared against the remainder
    of the input.*
  - **mscanner.scripts.update**: *Update the MScanner database with new articles*
  - **mscanner.scripts.validate**: *Calculates cross validation results for the MScanner paper.*

| Trees | Indices | Help | | MScanner | | --- | |
| --- | --- | --- | --- | --- |

|  |  |
| --- | --- |
| Generated by Epydoc 3.0beta1 on Fri Nov 23 09:13:20 2007 | http://epydoc.sourceforge.net |
