# Supplementary material for: MScanner: a classifier for retrieving Medline citations
Source: BMC Bioinformatics. 2008 Feb 19;9:108. doi: 10.1186/1471-2105-9-108 (PMC2263023; doi:10.1186/1471-2105-9-108)
Supplement: Additional file 3 — Source code for MScanner. mscanner-20071123.zip is a ZIP archive containing the Python 2.5 source code for MScanner, licensed under the GNU General Public License. It also contains API documentation in HTML format. Updated versions will be made available at . [file 1471-2105-9-108-S3.zip › mscanner/help/api/mscanner.core-module.html]

xml version="1.0" encoding="ascii"?


mscanner.core


| Trees | Indices | Help | | MScanner | | --- | |
| --- | --- | --- | --- | --- |

|  |  |  |  |
| --- | --- | --- | --- |
| Package mscanner :: Package core | |  | | --- | | [hide private] | | [frames] | no frames] | |

# Package core

source code  
  
The core MScanner modules  
  


|  |  |  |  |
| --- | --- | --- | --- |
| |  |  | | --- | --- | | Submodules | [hide private] | | |
| - **mscanner.core.CitationTable**: *Writes HTML pages with interactive citation tables* - **mscanner.core.FeatureScores**: *Calculates feature scores from occurrence counts* - **mscanner.core.Plotter**: *Plotting functions for all graphs produced in cross   validation.* - **mscanner.core.QueryManager**: *Environment for performing query-based analyses.* - **mscanner.core.Storage**: *Dictionary subclasses supporting dotted access* - **mscanner.core.ValidationManager**: *Environment for performing cross-validation-based analyses* - **mscanner.core.Validator**: *Cross-validation and performance statistic calculation* - **mscanner.core.iofuncs**: *I/O functions - for reading and writing certain file   formats.* - **mscanner.core.metrics**: *Calculates performance statistics given the scores of the   positive and negative citations* |

  


| Trees | Indices | Help | | MScanner | | --- | |
| --- | --- | --- | --- | --- |

|  |  |
| --- | --- |
| Generated by Epydoc 3.0beta1 on Fri Nov 23 09:13:20 2007 | http://epydoc.sourceforge.net |
