# Supplementary material for: MScanner: a classifier for retrieving Medline citations
Source: BMC Bioinformatics. 2008 Feb 19;9:108. doi: 10.1186/1471-2105-9-108 (PMC2263023; doi:10.1186/1471-2105-9-108)
Supplement: Additional file 3 — Source code for MScanner. mscanner-20071123.zip is a ZIP archive containing the Python 2.5 source code for MScanner, licensed under the GNU General Public License. It also contains API documentation in HTML format. Updated versions will be made available at . [file 1471-2105-9-108-S3.zip › mscanner/help/api/mscanner.core.citationtable-module.html]

xml version="1.0" encoding="ascii"?


mscanner.core.CitationTable


| Trees | Indices | Help | | MScanner | | --- | |
| --- | --- | --- | --- | --- |

|  |  |  |  |
| --- | --- | --- | --- |
| Package mscanner :: Package core :: Module CitationTable | |  | | --- | | [hide private] | | [frames] | no frames] | |

# Module CitationTable

source code  
  
Writes HTML pages with interactive citation tables  
  


---

**Author:**
Graham Poulter <http://graham.poulter.googlepages.com>

**Copyright:**
2007 Graham Poulter

**License:**
This program is free software: you can redistribute it and/or
modify it under the terms of the GNU General Public License as
published by the
Free Software Foundation, either version 3 of the License, or (at
your option)
any later version.
This program is distributed in the hope that it will be useful, but
WITHOUT ANY
WARRANTY; without even the implied warranty of MERCHANTABILITY or
FITNESS FOR A
PARTICULAR PURPOSE. See the GNU General Public License for more
details.
You should have received a copy of the GNU General Public License
along with
this program. If not, see <http://www.gnu.org/licenses/>.


|  |  |  |  |
| --- | --- | --- | --- |
| |  |  | | --- | --- | | Functions | [hide private] | | |
|  | |  |  | | --- | --- | | write\_citations(mode, dataset, citations, fname, perfile)  Writes a set of HTML files containing citation records | source code | |
|  | |  |  | | --- | --- | | CitationTable(startrank, citations)  Create an HTML table of citations (uses ElementTree) | source code | |


|  |  |  |  |
| --- | --- | --- | --- |
| |  |  | | --- | --- | | Function Details | [hide private] | | |

|  |  |  |
| --- | --- | --- |
| |  |  | | --- | --- | | write\_citations(mode, dataset, citations, fname, perfile) | source code |  Writes a set of HTML files containing citation records Parameters:  - **`mode`** - 'input' or 'output' - **`dataset`** - Dataset title to print at the top of the page - **`citations`** - List of (score, Article) in descending order of score - **`fname`** - Basic name for output files. ../results.html becomes   ../results.html, ../results\_02.html, ../results\_03.html etc.) - **`perfile`** - Number of citations per file (the very last file may however   have up to 2\*perfile-1 citations) |

|  |  |  |
| --- | --- | --- |
| |  |  | | --- | --- | | CitationTable(startrank, citations) | source code |   Create an HTML table of citations (uses ElementTree) We use Cheetah when there is more HTML than logic, and ElementTree when there is more logic than HTML. The old Cheetah template was getting cluttered from all the logic. This way also outputs less whitespace. Parameters:  - **`startrank`** - Rank of the first article in the table - **`citations`** - Iterable of (score, Article) in decreasing order of score  Returns:  HTML string for the <table> element containing citations |

  


| Trees | Indices | Help | | MScanner | | --- | |
| --- | --- | --- | --- | --- |

|  |  |
| --- | --- |
| Generated by Epydoc 3.0beta1 on Fri Nov 23 09:13:20 2007 | http://epydoc.sourceforge.net |
