# Supplementary material for: MScanner: a classifier for retrieving Medline citations
Source: BMC Bioinformatics. 2008 Feb 19;9:108. doi: 10.1186/1471-2105-9-108 (PMC2263023; doi:10.1186/1471-2105-9-108)
Supplement: Additional file 3 — Source code for MScanner. mscanner-20071123.zip is a ZIP archive containing the Python 2.5 source code for MScanner, licensed under the GNU General Public License. It also contains API documentation in HTML format. Updated versions will be made available at . [file 1471-2105-9-108-S3.zip › mscanner/help/api/mscanner.core.citationtable-pysrc.html]

xml version="1.0" encoding="ascii"?


mscanner.core.CitationTable


| Trees | Indices | Help | | MScanner | | --- | |
| --- | --- | --- | --- | --- |

|  |  |  |  |
| --- | --- | --- | --- |
| Package mscanner :: Package core :: Module CitationTable | |  | | --- | | [hide private] | | [frames] | no frames] | |

# Source Code for Module mscanner.core.CitationTable

```
  1  """Writes HTML pages with interactive citation tables""" 
  2   
  3  from __future__ import with_statement 
  4  from __future__ import division 
  5   
  6  from mscanner.configuration import rc 
  7  from mscanner.core import iofuncs 
  8  from Cheetah.Template import Template 
  9   
 10  import warnings 
 11  warnings.simplefilter("ignore", UserWarning) 
 12   
 13   
 14  __copyright__ = "2007 Graham Poulter" 
 15  __author__ = "Graham Poulter <http://graham.poulter.googlepages.com>" 
 16  __license__ = """This program is free software: you can redistribute it and/or 
 17  modify it under the terms of the GNU General Public License as published by the 
 18  Free Software Foundation, either version 3 of the License, or (at your option) 
 19  any later version. 
 20   
 21  This program is distributed in the hope that it will be useful, but WITHOUT ANY 
 22  WARRANTY; without even the implied warranty of MERCHANTABILITY or FITNESS FOR A 
 23  PARTICULAR PURPOSE. See the GNU General Public License for more details. 
 24   
 25  You should have received a copy of the GNU General Public License along with 
 26  this program. If not, see <http://www.gnu.org/licenses/>.""" 
 27   
 28   


29 -def write_citations(mode, dataset, citations, fname, perfile):


30      """Writes a set of HTML files containing citation records 
 31       
 32      @param mode: 'input' or 'output' 
 33       
 34      @param dataset: Dataset title to print at the top of the page 
 35   
 36      @param citations: List of (score, Article) in descending order of score 
 37   
 38      @param fname: Basic name for output files. ../results.html becomes 
 39      ../results.html, ../results_02.html, ../results_03.html etc.) 
 40   
 41      @param perfile: Number of citations per file (the very last file  
 42      may however have up to 2*perfile-1 citations) 
 43      """ 
 44      # List of ranks where each file's citation records start 
 45      starts = range(0, len(citations), perfile) 
 46      # If the last file is less than half full, we concat to second-last 
 47      if len(starts)>1 and (len(citations)-starts[-1]) < (perfile//2): 
 48          del starts[-1] 
 49      # List of HTML files containing the citations 
 50      from path import path 
 51      fnames = [path(fname.basename())] # First file is the basic name 
 52      fnames += [path(fname.namebase + ("_%02d" % x) + fname.ext) 
 53                  for x in range(2, 1+len(starts))] 
 54      values = dict() 
 55      page = Template( 
 56          file=str(rc.templates/"citations.tmpl"),  
 57          filter="Filter", searchList=[values]) 
 58      for count, start in enumerate(starts): 
 59          if count+1 < len(starts): 
 60              towrite = citations[start:start+perfile] 
 61          else: 
 62              towrite = citations[start:] 
 63          values.update(dict( 
 64              cite_table = CitationTable(start+1, towrite), 
 65              dataset = dataset, 
 66              mode = mode,  
 67              report_length = len(towrite), 
 68              filelist = fnames, 
 69              cur_idx = count)) 
 70          with iofuncs.FileTransaction(fname.dirname()/fnames[count], "w") as ft: 
 71              page.respond(ft)

 72           
 73   
 74   


75 -def CitationTable(startrank, citations):


76      """Create an HTML table of citations (uses ElementTree) 
 77       
 78      We use Cheetah when there is more HTML than logic, and ElementTree when 
 79      there is more logic than HTML. The old Cheetah template was getting 
 80      cluttered from all the logic. This way also outputs less whitespace. 
 81       
 82      @param startrank: Rank of the first article in the table 
 83   
 84      @param citations: Iterable of (score, Article) in decreasing order of score 
 85       
 86      @return: HTML string for the <table> element containing citations 
 87      """ 
 88      from xml.etree.cElementTree import ElementTree, Element, SubElement 
 89      table = Element("table", id="citations") 
 90      ncols = 9 # Number of columns in the table 
 91      cg = SubElement(table, "colgroup") 
 92      SubElement(cg, "col", {"class":"classification"}) 
 93      SubElement(cg, "col", {"class":"rank"}) 
 94      SubElement(cg, "col", {"class":"score"}) 
 95      SubElement(cg, "col", {"class":"pmid"}) 
 96      SubElement(cg, "col", {"class":"date"}) 
 97      SubElement(cg, "col", {"class":"author"}) 
 98      SubElement(cg, "col", {"class":"abstract"}) 
 99      SubElement(cg, "col", {"class":"title"}) 
100      SubElement(cg, "col", {"class":"journal"}) 
101      thead = SubElement(table, "thead") 
102      tr = SubElement(thead, "tr") 
103      SubElement(tr, "th", title="Classification").text = "C" 
104      SubElement(tr, "th", title="Rank").text = "R" 
105      SubElement(tr, "th").text = "Score" 
106      SubElement(tr, "th").text = "PMID" 
107      SubElement(tr, "th").text = "Date" 
108      SubElement(tr, "th", title="Author").text = "Au" 
109      SubElement(tr, "th", title="Abstract").text = "Ab" 
110      SubElement(tr, "th").text = "Title" 
111      SubElement(tr, "th").text = "Journal" 
112      tbody = SubElement(table, "tbody") 
113      ncbi = "http://www.ncbi.nlm.nih.gov/entrez/query.fcgi?" 
114      ncbi_pmid = ncbi+"cmd=Retrieve&db=pubmed&list_uids=" 
115      ncbi_jour = ncbi+"CMD=search&DB=journals&term=" 
116      for idx, (score, art) in enumerate(citations): 
117          pmid = str(art.pmid) 
118          tr = SubElement(tbody, "tr", {"class":"main"}, id="P"+pmid) 
119          # Classification 
120          SubElement(tr, "td").text = " " 
121          # Rank 
122          SubElement(tr, "td").text = str(idx+startrank) 
123          # Score 
124          SubElement(tr, "td").text = "%.2f" % score 
125          # PMID 
126          td = SubElement(tr, "td") 
127          SubElement(td, "a", href=ncbi_pmid+pmid).text = pmid 
128          # Date the record acquired "Medline" status 
129          td = SubElement(tr, "td") 
130          td.text = "%04d.%02d.%02d" % art.date_completed 
131          # Expand Author button 
132          td = SubElement(tr, "td") 
133          td.text = "+" if art.authors else " " 
134          # Expand Abstract button 
135          td = SubElement(tr, "td") 
136          td.text = "+" if art.abstract else " " 
137          # Title 
138          td = SubElement(tr, "td") 
139          td.text = art.title 
140          # ISSN 
141          td = SubElement(tr, "td") 
142          a = SubElement(td, "a") 
143          a.text = " " 
144          if art.issn: 
145              a.set("href", ncbi_jour+art.issn) 
146              a.text = art.journal if art.journal else art.issn 
147          # Expanded authors 
148          tr = SubElement(tbody, "tr", {"class":"author"}) 
149          td = SubElement(tr, "td", {"colspan":str(ncols)}) 
150          td.text = " " 
151          if art.authors: 
152              for initials, lastname in art.authors: 
153                  if initials: td.text += initials + " " 
154                  if lastname: td.text += lastname + ", " 
155          # Expanded Abstract 
156          tr = SubElement(tbody, "tr", {"class":"abstract"}) 
157          td = SubElement(tr, "td", {"colspan":str(ncols)}) 
158          td.text = " " 
159          if art.abstract:                         
160              td.text = art.abstract 
161      import cStringIO 
162      s = cStringIO.StringIO() 
163      # Tell silly etree to use UTF-8 and not "us-ascii" for output 
164      ElementTree(table).write(s, "utf-8")  
165      return s.getvalue()

166
```

  


| Trees | Indices | Help | | MScanner | | --- | |
| --- | --- | --- | --- | --- |

|  |  |
| --- | --- |
| Generated by Epydoc 3.0beta1 on Fri Nov 23 09:13:24 2007 | http://epydoc.sourceforge.net |
