# Supplementary material for: MScanner: a classifier for retrieving Medline citations
Source: BMC Bioinformatics. 2008 Feb 19;9:108. doi: 10.1186/1471-2105-9-108 (PMC2263023; doi:10.1186/1471-2105-9-108)
Supplement: Additional file 3 — Source code for MScanner. mscanner-20071123.zip is a ZIP archive containing the Python 2.5 source code for MScanner, licensed under the GNU General Public License. It also contains API documentation in HTML format. Updated versions will be made available at . [file 1471-2105-9-108-S3.zip › mscanner/help/api/mscanner.core.cscore-module.html]

xml version="1.0" encoding="ascii"?


mscanner.core.cscore


| Trees | Indices | Help | | MScanner | | --- | |
| --- | --- | --- | --- | --- |

|  |  |  |  |
| --- | --- | --- | --- |
| Package mscanner :: Package core :: Package cscore | |  | | --- | | [hide private] | | [frames] | no frames] | |

# Package cscore

source code  
  
Calculates citation scores  
  


---

**Author:**
Graham Poulter <http://graham.poulter.googlepages.com>

**Copyright:**
2007 Graham Poulter

**License:**
This program is free software: you can redistribute it and/or
modify it under the terms of the GNU General Public License as
published by the
Free Software Foundation, either version 3 of the License, or (at
your option)
any later version.
This program is distributed in the hope that it will be useful, but
WITHOUT ANY
WARRANTY; without even the implied warranty of MERCHANTABILITY or
FITNESS FOR A
PARTICULAR PURPOSE. See the GNU General Public License for more
details.
You should have received a copy of the GNU General Public License
along with
this program. If not, see <http://www.gnu.org/licenses/>.


|  |  |  |  |
| --- | --- | --- | --- |
| |  |  | | --- | --- | | Functions | [hide private] | | |
|  | |  |  | | --- | --- | | pyscore(docs, featscores, offset, limit, threshold=None, exclude=`[``]`)  Get scores for given documents | source code | |
|  | |  |  | | --- | --- | | pyscore\_adaptor(docstream, numdocs, featscores, offset, limit, safety, threshold=None, exclude=`[``]`)  Calls pyscore, given arguments suitable for cscore\_pipe/dll | source code | |
|  | |  |  | | --- | --- | | cscore\_pipe(docstream, numdocs, featscores, offset, limit, safety, threshold=None, exclude=`[``]`)  Calculate article scores by piping to the cscore program | source code | |
|  | |  |  | | --- | --- | | cscore\_dll(docstream, numdocs, featscores, offset, limit, safety, threshold=None, exclude=`[``]`)  Calculate article scores, using ctypes to call cscores | source code | |
|  | |  |  | | --- | --- | | score(docstream, numdocs, featscores, offset, limit, safety, threshold=None, exclude=`[``]`)  Default score calculation function (parameters as for cscore\_pipe) | source code | |
|  | |  |  | | --- | --- | | choose\_score()  Select the fastest available score calculator and assign it to the module variable score | source code | |


|  |  |  |  |
| --- | --- | --- | --- |
| |  |  | | --- | --- | | Function Details | [hide private] | | |

|  |  |  |
| --- | --- | --- |
| |  |  | | --- | --- | | pyscore(docs, featscores, offset, limit, threshold=None, exclude=`[``]`) | source code |   Get scores for given documents We iterates over docs to yield scores. Skips members of exclude, and returns up to to limit results. Parameters:  - **`docs`** - Iterator over (integer doc ID, array of feature ID) pairs - **`featscores`** - Array of feature scores (mapping feature ID to score) - **`offset`** - Arbitrary amount to add to citation score - **`limit`** - Max number of results to return - **`exclude`** - PMIDs to exclude from scoring  Returns:  Iteration of (score, PMID) pairs |

|  |  |  |
| --- | --- | --- |
| |  |  | | --- | --- | | cscore\_pipe(docstream, numdocs, featscores, offset, limit, safety, threshold=None, exclude=`[``]`) | source code |   Calculate article scores by piping to the cscore program The cscore program processes a feature stream to return a (score, pmid) pairs as a binary stream. Parameters:  - **`docstream`** - Path to file containing feature vectors for documents to   score, in mscanner.medline.FeatureDatabase.FeatureStream   format. - **`numdocs`** - Number of documents in the stream of feature vectors. - **`featscores`** - Vector of feature score doubles - **`offset`** - Arbitrary amount to add to citation score - **`limit`** - Maximum number of results to return. - **`safety`** - Number of spare results in processing (because some might be   members of exclude) - **`exclude`** - PMIDs to remove from cscore results - **`threshold`** - Cutoff score for including an article in the results  Returns:  List of (score, PMID) pairs in decreasing order of score |

|  |  |  |
| --- | --- | --- |
| |  |  | | --- | --- | | cscore\_dll(docstream, numdocs, featscores, offset, limit, safety, threshold=None, exclude=`[``]`) | source code |  Calculate article scores, using ctypes to call cscores Parameters:  - **`docstream`** - Path to file containing feature vectors for documents to score   (formatted as in   mscanner.medline.FeatureDatabase.FeatureStream) - **`numdocs`** - Number of documents in the stream of feature vectors. - **`featscores`** - Vector of feature score doubles - **`offset`** - Arbitrary amount to add to citation score - **`limit`** - Maximum number of results to return. - **`safety`** - Number of spare results in processing (because some might be   members of exclude) - **`exclude`** - PMIDs to remove from cscore results - **`threshold`** - Cutoff score for including an article in the results  Returns:  List of (score, PMID) pairs in decreasing order of score |

  


| Trees | Indices | Help | | MScanner | | --- | |
| --- | --- | --- | --- | --- |

|  |  |
| --- | --- |
| Generated by Epydoc 3.0beta1 on Thu Nov 08 18:36:47 2007 | http://epydoc.sourceforge.net |
