# Supplementary material for: MScanner: a classifier for retrieving Medline citations
Source: BMC Bioinformatics. 2008 Feb 19;9:108. doi: 10.1186/1471-2105-9-108 (PMC2263023; doi:10.1186/1471-2105-9-108)
Supplement: Additional file 3 — Source code for MScanner. mscanner-20071123.zip is a ZIP archive containing the Python 2.5 source code for MScanner, licensed under the GNU General Public License. It also contains API documentation in HTML format. Updated versions will be made available at . [file 1471-2105-9-108-S3.zip › mscanner/help/api/mscanner.core.cscore-pysrc.html]

xml version="1.0" encoding="ascii"?


mscanner.core.cscore


| Trees | Indices | Help | | MScanner | | --- | |
| --- | --- | --- | --- | --- |

|  |  |  |  |
| --- | --- | --- | --- |
| Package mscanner :: Package core :: Package cscore | |  | | --- | | [hide private] | | [frames] | no frames] | |

# Source Code for Package mscanner.core.cscore

```
  1  """Calculates citation scores""" 
  2   
  3  from __future__ import division 
  4  from mscanner.configuration import rc 
  5  import logging as log 
  6  import numpy as nx 
  7   
  8   
  9  __copyright__ = "2007 Graham Poulter" 
 10  __author__ = "Graham Poulter <http://graham.poulter.googlepages.com>" 
 11  __license__ = """This program is free software: you can redistribute it and/or 
 12  modify it under the terms of the GNU General Public License as published by the 
 13  Free Software Foundation, either version 3 of the License, or (at your option) 
 14  any later version. 
 15   
 16  This program is distributed in the hope that it will be useful, but WITHOUT ANY 
 17  WARRANTY; without even the implied warranty of MERCHANTABILITY or FITNESS FOR A 
 18  PARTICULAR PURPOSE. See the GNU General Public License for more details. 
 19   
 20  You should have received a copy of the GNU General Public License along with 
 21  this program. If not, see <http://www.gnu.org/licenses/>.""" 
 22   
 23   


24 -def pyscore(docs, featscores, offset, limit, threshold=None, exclude=[]):


25      """Get scores for given documents 
 26       
 27      We iterates over docs to yield scores. Skips members of exclude, and 
 28      returns up to to limit results. 
 29       
 30      @param docs: Iterator over (integer doc ID, array of feature ID) pairs 
 31   
 32      @param featscores: Array of feature scores (mapping feature ID to score) 
 33       
 34      @param offset: Arbitrary amount to add to citation score 
 35       
 36      @param limit: Max number of results to return 
 37       
 38      @param exclude: PMIDs to exclude from scoring 
 39       
 40      @return: Iteration of (score, PMID) pairs 
 41      """ 
 42      results = [(-100000, 0)] * limit 
 43      import heapq 
 44      ndocs = 0 
 45      log.debug("Calculating article scores") 
 46      marker = 0 
 47      for idx, (docid, features) in enumerate(docs): 
 48          if idx == marker: 
 49              log.debug("Scored %d citations so far", idx) 
 50              marker += 100000 
 51          score = offset + nx.sum(featscores[features]) 
 52          if (threshold is None or score >= threshold) and docid not in exclude: 
 53              ndocs += 1 
 54              if score >= results[0][0]: 
 55                  heapq.heapreplace(results, (score,docid)) 
 56      if ndocs < limit: 
 57          limit = ndocs 
 58      return heapq.nlargest(limit, results)

 59   
 60   


61 -def pyscore_adaptor(docstream, numdocs, featscores, offset, 
 62                      limit, safety, threshold=None, exclude=[]):


63      """Calls pyscore, given arguments suitable for cscore_pipe/dll""" 
 64      from mscanner.medline.FeatureDatabase import FeatureStream 
 65      docs = FeatureStream(open(docstream, "rb")) 
 66      try: 
 67          return pyscore(docs, featscores, offset, limit, threshold, exclude) 
 68      finally: 
 69          docs.close()

 70   
 71   


72 -def cscore_pipe(docstream, numdocs, featscores, offset, 
 73                  limit, safety, threshold=None, exclude=[]):


74      """Calculate article scores by piping to the cscore program 
 75       
 76      The cscore program processes a feature stream to return a (score, pmid) 
 77      pairs as a binary stream. 
 78       
 79      @param docstream: Path to file containing feature vectors for documents to 
 80      score, in L{mscanner.medline.FeatureDatabase.FeatureStream} format. 
 81       
 82      @param numdocs: Number of documents in the stream of feature vectors. 
 83       
 84      @param featscores: Vector of feature score doubles 
 85       
 86      @param offset: Arbitrary amount to add to citation score 
 87   
 88      @param limit: Maximum number of results to return. 
 89       
 90      @param safety: Number of spare results in processing (because 
 91      some might be members of exclude) 
 92       
 93      @param exclude: PMIDs to remove from cscore results 
 94   
 95      @param threshold: Cutoff score for including an article in the results 
 96   
 97      @return: List of (score, PMID) pairs in decreasing order of score 
 98      """ 
 99      import struct 
100      import subprocess as sp 
101      p = sp.Popen( 
102          [rc.cscore_path, docstream, str(numdocs),  
103           str(len(featscores)), str(limit+safety), str(offset)], 
104          stdout=sp.PIPE, stdin=sp.PIPE) 
105      p.stdin.write(featscores.tostring()) 
106      s = p.stdout.read(8) 
107      count = 0 
108      # Go through results in decreasing order to filter them 
109      while s != "": 
110          score, pmid = struct.unpack("fI", s) 
111          if (threshold is None or score > threshold) and pmid not in exclude: 
112              yield score, pmid 
113              count += 1 
114              if count >= limit: 
115                  break 
116          s = p.stdout.read(8) 
117      p.stdout.close()

118   
119   


120 -def cscore_dll(docstream, numdocs, featscores, offset, 
121                 limit, safety, threshold=None, exclude=[]):


122      """Calculate article scores, using ctypes to call cscores 
123       
124      @param docstream: Path to file containing feature vectors for documents to 
125      score (formatted as in mscanner.medline.FeatureDatabase.FeatureStream) 
126       
127      @param numdocs: Number of documents in the stream of feature vectors. 
128       
129      @param featscores: Vector of feature score doubles 
130       
131      @param offset: Arbitrary amount to add to citation score 
132       
133      @param limit: Maximum number of results to return. 
134       
135      @param safety: Number of spare results in processing (because 
136      some might be members of exclude) 
137       
138      @param exclude: PMIDs to remove from cscore results 
139   
140      @param threshold: Cutoff score for including an article in the results 
141   
142      @return: List of (score, PMID) pairs in decreasing order of score 
143      """ 
144      from ctypes import cdll, c_int, c_char_p, c_float, c_double 
145      from itertools import izip 
146      import numpy as nx 
147      # Set up arguments and call cscore2 function using ctypes 
148      carray = lambda x: nx.ctypeslib.ndpointer(dtype=x, ndim=1, flags='CONTIGUOUS') 
149      cscore = cdll.LoadLibrary(rc.cscore_dll) 
150      cscore.cscore.argtypes = [  
151          c_char_p, c_int, c_int, c_int, c_float, 
152          carray(nx.float64), carray(nx.float32), carray(nx.int32) ] 
153      o_scores = nx.zeros(limit+safety, dtype=nx.float32) 
154      o_pmids = nx.zeros(limit+safety, dtype=nx.int32) 
155      cscore.cscore( 
156          docstream, numdocs, len(featscores), limit+safety, offset, 
157          featscores, o_scores, o_pmids) 
158      # Go through results in decreasing order to filter them 
159      count = 0 
160      for score, pmid in izip(o_scores, o_pmids): 
161          if (threshold is None or score > threshold) and pmid not in exclude: 
162              yield score, pmid 
163              count += 1 
164              if count >= limit: 
165                  break

166   
167   
168  score = pyscore_adaptor 
169  """Default score calculation function (parameters as for L{cscore_pipe})""" 
170   


171 -def choose_score():


172      """Select the fastest available score calculator and assign it 
173      to the module variable L{score}""" 
174      global score 
175      try: 
176          import ctypes 
177          if rc.cscore_dll.isfile(): 
178              score = cscore_dll 
179      except ImportError: 
180          pass 
181      if score == pyscore_adaptor and rc.cscore_path.isfile(): 
182          score = cscore_pipe

183
```

  


| Trees | Indices | Help | | MScanner | | --- | |
| --- | --- | --- | --- | --- |

|  |  |
| --- | --- |
| Generated by Epydoc 3.0beta1 on Thu Nov 08 18:36:51 2007 | http://epydoc.sourceforge.net |
