# Supplementary material for: MScanner: a classifier for retrieving Medline citations
Source: BMC Bioinformatics. 2008 Feb 19;9:108. doi: 10.1186/1471-2105-9-108 (PMC2263023; doi:10.1186/1471-2105-9-108)
Supplement: Additional file 3 — Source code for MScanner. mscanner-20071123.zip is a ZIP archive containing the Python 2.5 source code for MScanner, licensed under the GNU General Public License. It also contains API documentation in HTML format. Updated versions will be made available at . [file 1471-2105-9-108-S3.zip › mscanner/help/api/mscanner.core.FeatureScores-module.html]

xml version="1.0" encoding="ascii"?


mscanner.core.FeatureScores


| Trees | Indices | Help | | MScanner | | --- | |
| --- | --- | --- | --- | --- |

|  |  |  |  |
| --- | --- | --- | --- |
| Package mscanner :: Package core :: Module FeatureScores | |  | | --- | | [hide private] | | [frames] | no frames] | |

# Module FeatureScores

source code  
  
Calculates feature scores from occurrence counts  
  


---

**Author:**
Graham Poulter <http://graham.poulter.googlepages.com>

**Copyright:**
2007 Graham Poulter

**License:**
This program is free software: you can redistribute it and/or
modify it under the terms of the GNU General Public License as
published by the
Free Software Foundation, either version 3 of the License, or (at
your option)
any later version.
This program is distributed in the hope that it will be useful, but
WITHOUT ANY
WARRANTY; without even the implied warranty of MERCHANTABILITY or
FITNESS FOR A
PARTICULAR PURPOSE. See the GNU General Public License for more
details.
You should have received a copy of the GNU General Public License
along with
this program. If not, see <http://www.gnu.org/licenses/>.


|  |  |  |  |
| --- | --- | --- | --- |
| |  |  | | --- | --- | | Classes | [hide private] | | |
|  | FeatureScores  Feature score calculation and saving, with choice of calculation method, and methods to exclude certain kinds of features. |


|  |  |  |  |
| --- | --- | --- | --- |
| |  |  | | --- | --- | | Functions | [hide private] | | |
|  | |  |  | | --- | --- | | FeatureCounts(nfeats, featdb, docids)  Count occurrenes of each feature in a set of articles | source code | |


|  |  |  |  |
| --- | --- | --- | --- |
| |  |  | | --- | --- | | Function Details | [hide private] | | |

|  |  |  |
| --- | --- | --- |
| |  |  | | --- | --- | | FeatureCounts(nfeats, featdb, docids) | source code |  Count occurrenes of each feature in a set of articles Parameters:  - **`nfeats`** - Number of distinct features (length of `docids`) - **`featdb`** - Mapping from document ID to array of feature IDs - **`docids`** - Iterable of document IDs whose features are to be counted  Returns:  Array of length `nfeats`, containing occurrence count of each feature |

  


| Trees | Indices | Help | | MScanner | | --- | |
| --- | --- | --- | --- | --- |

|  |  |
| --- | --- |
| Generated by Epydoc 3.0beta1 on Fri Nov 23 09:13:20 2007 | http://epydoc.sourceforge.net |
