# Supplementary material for: MScanner: a classifier for retrieving Medline citations
Source: BMC Bioinformatics. 2008 Feb 19;9:108. doi: 10.1186/1471-2105-9-108 (PMC2263023; doi:10.1186/1471-2105-9-108)
Supplement: Additional file 3 — Source code for MScanner. mscanner-20071123.zip is a ZIP archive containing the Python 2.5 source code for MScanner, licensed under the GNU General Public License. It also contains API documentation in HTML format. Updated versions will be made available at . [file 1471-2105-9-108-S3.zip › mscanner/help/api/mscanner.core.FeatureScores-pysrc.html]

xml version="1.0" encoding="ascii"?


mscanner.core.FeatureScores


| Trees | Indices | Help | | MScanner | | --- | |
| --- | --- | --- | --- | --- |

|  |  |  |  |
| --- | --- | --- | --- |
| Package mscanner :: Package core :: Module FeatureScores | |  | | --- | | [hide private] | | [frames] | no frames] | |

# Source Code for Module mscanner.core.FeatureScores

```
  1  """Calculates feature scores from occurrence counts""" 
  2   
  3  from __future__ import division 
  4  import numpy as nx 
  5   
  6  from mscanner import update, delattrs 
  7  from mscanner.core.Storage import Storage 
  8   
  9   
 10  __copyright__ = "2007 Graham Poulter" 
 11  __author__ = "Graham Poulter <http://graham.poulter.googlepages.com>" 
 12  __license__ = """This program is free software: you can redistribute it and/or 
 13  modify it under the terms of the GNU General Public License as published by the 
 14  Free Software Foundation, either version 3 of the License, or (at your option) 
 15  any later version. 
 16   
 17  This program is distributed in the hope that it will be useful, but WITHOUT ANY 
 18  WARRANTY; without even the implied warranty of MERCHANTABILITY or FITNESS FOR A 
 19  PARTICULAR PURPOSE. See the GNU General Public License for more details. 
 20   
 21  You should have received a copy of the GNU General Public License along with 
 22  this program. If not, see <http://www.gnu.org/licenses/>.""" 
 23   
 24   


25 -class FeatureScores(object):


26      """Feature score calculation and saving, with choice of calculation method, 
 27      and methods to exclude certain kinds of features. 
 28       
 29      @group Set via constructor: featmap, pseudocount, mask, make_scores, get_postmask 
 30       
 31      @ivar featmap: L{FeatureMapping} object 
 32       
 33      @ivar pseudocount: Prior psuedocount to use for features, or None 
 34      to use feature counts equal to Medline frequency. 
 35       
 36      @ivar mask: Either None or a boolean array to mask some features scores 
 37      to zero (this is to exclude features by category, not by score). 
 38       
 39      @ivar make_scores: Method used to calculate the feature scores. 
 40       
 41      @ivar get_postmask: Method used to calculate a dynamic mask 
 42      array once the feature scores are known. 
 43       
 44       
 45      @group Set by update: pos_counts, neg_counts, pdocs, ndocs, prior 
 46       
 47      @ivar pos_counts: Array of feature counts in positive documents 
 48       
 49      @ivar neg_counts: Array of feature counts in negatives documents 
 50       
 51      @ivar pdocs: Number of positive documents 
 52       
 53      @ivar ndocs: Number of negative documents 
 54       
 55      @ivar prior: Bayes prior to add to the score.  If None, estimate 
 56      using the ratio of relevant to irrelevant articles in the data. 
 57   
 58       
 59      @group Set via make_scores: scores, pfreqs, nfreqs, base 
 60       
 61      @ivar scores: Score of each feature 
 62     
 63      @ivar pfreqs: Numerator of score fraction 
 64       
 65      @ivar nfreqs: Denominator of score fraction 
 66       
 67      @ivar base: Value to be added to all article scores 
 68      """ 
 69   


70 -    def __init__(self,  
 71                   featmap, 
 72                   pseudocount=None, 
 73                   mask=None, 
 74                   make_scores="scores_bayes", 
 75                   get_postmask=None):


76          """Initialise FeatureScores object (parameters are instance variables)""" 
 77          make_scores = getattr(self, make_scores) 
 78          if isinstance(get_postmask, basestring): 
 79              get_postmask = getattr(self, get_postmask) 
 80          prior = 0 
 81          update(self, locals())

 82   
 83   


84 -    def scores_of(self, featdb, pmids):


85          """Calculate vector of scores given an iterable of PubMed IDs. 
 86           
 87          @param featdb: Mapping from PMID to feature vector 
 88          @param pmids: Iterable of keys into L{featdb} 
 89          @return: Vector containing document scores corresponding to the pmids. 
 90          """ 
 91          off = self.base + self.prior 
 92          sc = self.scores 
 93          return nx.array([off+nx.sum(sc[featdb[d]]) for d in pmids], nx.float32)

 94       
 95   


96 -    def __len__(self):


97          """Number of features""" 
 98          return len(self.featmap)

 99   
100   


101 -    def update(self, pos_counts, neg_counts, pdocs, ndocs, prior=None):


102          """Change the feature counts and numbers of documents, clear 
103          old score calculations, and calculate new scores.""" 
104          if prior is None: 
105              if pdocs == 0 or ndocs == 0: 
106                  prior = 0 
107              else: 
108                  prior = nx.log(pdocs/ndocs) 
109          base = 0 
110          update(self, locals()) 
111          self.make_scores() 
112          self._mask_scores() 
113          delattrs(self, "_stats", "_tfidf")

114   
115   


116 -    def scores_bayes(s):


117          """Document generated using multivariate Bernoulli distribution. 
118           
119          Feature non-occurrence is modeled as a base score for the 
120          document with no features, and an adjustment to the  
121          feature occurrence scores.""" 
122          s._make_pseudovec() 
123          # Posterior term frequencies in relevant articles 
124          s.pfreqs = (s.pseudocount+s.pos_counts) / (1+s.pdocs) 
125          # Posterior term frequencies in irrelevant articles 
126          s.nfreqs = (s.pseudocount+s.neg_counts) / (1+s.ndocs) 
127          # Support scores for bernoulli successes 
128          s.present_scores = nx.log(s.pfreqs/s.nfreqs) 
129          # Support scores for bernoulli failures 
130          s.absent_scores = nx.log( (1-s.pfreqs)/(1-s.nfreqs) ) 
131          # Conversion to base score (no terms) and occurrence score 
132          s.base = nx.sum(s.absent_scores) 
133          s.scores = s.present_scores - s.absent_scores

134   
135   


136 -    def scores_noabsence(s):


137          """Calculates document probability as product of log likelihood ratios, 
138          with pseudocount weight equal to one article.""" 
139          s.base = 0 
140          s._make_pseudovec() 
141          s.pfreqs = (s.pseudocount+s.pos_counts) / (1+s.pdocs) 
142          s.nfreqs = (s.pseudocount+s.neg_counts) / (1+s.ndocs) 
143          s.scores = nx.log(s.pfreqs) - nx.log(s.nfreqs)

144   
145   


146 -    def scores_rubin(s):


147          """Models document as product of log likelihood ratios, using MLE 
148          feature probabilities - replacing zeroes with 1e-8""" 
149          s.base = 0 
150          s.pseudocount = 0 
151          s.pfreqs = s.pos_counts / float(s.pdocs) 
152          s.nfreqs = s.neg_counts / float(s.ndocs) 
153          s.pfreqs[s.pfreqs == 0.0] = 1e-8 
154          s.nfreqs[s.nfreqs == 0.0] = 1e-8 
155          s.scores = nx.log(s.pfreqs) - nx.log(s.nfreqs)

156   
157   


158 -    def _make_pseudovec(s):


159          """Calculates a pseudocount vector based on background frequencies 
160          if no constant pseudocount was specified""" 
161          if s.pseudocount is None: 
162              s.pseudocount = \ 
163               nx.array(s.featmap.counts, nx.float32) / s.featmap.numdocs

164   
165   


166 -    def _mask_scores(self):


167          """Set some feature scores to zero, effectively excluding them 
168          from consideration.  Uses L{mask} and L{get_postmask}""" 
169          if self.mask is not None: 
170              self.pfreqs[self.mask] = 0 
171              self.nfreqs[self.mask] = 0 
172              self.scores[self.mask] = 0 
173          if self.get_postmask: 
174              self.scores[self.get_postmask()] = 0

175   
176   


177 -    def mask_nonpositives(s):


178          """Mask for features not represented in the positives 
179           
180          @return: Boolean array for masked out features 
181          """ 
182          return s.pos_counts == 0

183   
184   
185      @property  


186 -    def stats(self):


187          """A Storage instance with statistics about the features 
188           
189          The following keys are present: 
190              - pos_occurrences: Total feature occurrences in positives 
191              - neg_occurrences: Total feature occurrences in negatives 
192              - feats_per_pos: Number of features per positive article 
193              - feats_per_neg: Number of features per negative article 
194              - distinct_feats: Number of distinct features 
195              - pos_distinct_feats: Number of of distinct features in positives 
196              - neg_distinct_feats: Number of of distinct features in negatives 
197          """ 
198          try:  
199              return self._stats 
200          except AttributeError:  
201              pass 
202          s = Storage() 
203          s.pdocs = self.pdocs 
204          s.ndocs = self.ndocs 
205          s.num_feats = len(self) 
206          s.pos_occurrences = int(nx.sum(self.pos_counts))  
207          s.feats_per_pos = 0.0 
208          if self.pdocs > 0: 
209              s.feats_per_pos = s.pos_occurrences / s.pdocs  
210          s.neg_occurrences = int(nx.sum(self.neg_counts)) 
211          s.feats_per_neg = 0.0 
212          if self.ndocs > 0: 
213              s.feats_per_neg = s.neg_occurrences / s.ndocs  
214          s.pos_distinct_feats = len(nx.nonzero(self.pos_counts)[0])  
215          s.neg_distinct_feats = len(nx.nonzero(self.neg_counts)[0])  
216          self._stats = s 
217          return self._stats

218   
219   
220      @property 


221 -    def tfidf(self):


222          """Vector of TF-IDF scores for each feature 
223           
224          Cache TF-IDF scores for terms, where for term frequency (TF) we treat 
225          the positive corpus as a single large document, but for inverse 
226          document frequency (IDF) each citation is a separate document.""" 
227          try:  
228              return self._tfidf 
229          except AttributeError:  
230              pass 
231          self._tfidf = nx.zeros(len(self.pos_counts), dtype=float) 
232          # Document frequency 
233          docfreq_t = self.pos_counts+self.neg_counts 
234          # Number of documents 
235          N = self.pdocs+self.ndocs # number of documents 
236          # Inverse Document Frequency (log N/df_t) 
237          u = (docfreq_t != 0) 
238          idf = nx.log(N / docfreq_t[u]) 
239          # Term frequency 
240          tf = (self.pos_counts[u] / nx.sum(self.pos_counts)) 
241          # Calculate TF.IDF 
242          self._tfidf[u] = tf * idf 
243          return self._tfidf

244   
245   


246 -    def get_best_tfidfs(self, count):


247          """Construct a table about the terms with the best TF.IDF 
248           
249          @param count: Number of rows to return 
250           
251          @return: List of  
252          (Term ID, TFIDF, (term, term_type), term score, pos count, neg count) 
253          """ 
254          from heapq import nlargest 
255          best_tfidfs = nlargest( 
256              count, enumerate(self.tfidf), key=lambda x:x[1]) 
257          return [ (t, tfidf, self.featmap[t], self.scores[t],  
258                    self.pos_counts[t], self.neg_counts[t]) 
259                    for t, tfidf in best_tfidfs ]

260   
261   


262 -    def write_csv(self, stream):


263          """Write features scores as CSV to an output stream""" 
264          stream.write(u"score,positives,negatives,numerator,"\ 
265                       u"denominator,pseudocount,termid,tfidf,type,term\n") 
266          s = self 
267          s.tfidf 
268          if not isinstance(s.pseudocount, nx.ndarray): 
269              pseudocount = nx.zeros_like(s.scores) + float(s.pseudocount) 
270          else: 
271              pseudocount = s.pseudocount 
272          for t, score in sorted( 
273              enumerate(s.scores), key=lambda x:x[1], reverse=True): 
274              if s.mask is not None and s.mask[t]: 
275                  continue 
276              stream.write( 
277                  u'%.3f,%d,%d,%.2e,%.2e,%.2e,%d,%.2f,%s,"%s"\n' %  
278                  (s.scores[t], s.pos_counts[t], s.neg_counts[t],  
279                   s.pfreqs[t], s.nfreqs[t], pseudocount[t], t, 
280                   s.tfidf[t], s.featmap[t][1], s.featmap[t][0]))

281   
282   
283   


284 -def FeatureCounts(nfeats, featdb, docids):


285      """Count occurrenes of each feature in a set of articles 
286   
287      @param nfeats: Number of distinct features (length of L{docids}) 
288   
289      @param featdb: Mapping from document ID to array of feature IDs 
290   
291      @param docids: Iterable of document IDs whose features are to be counted 
292   
293      @return: Array of length L{nfeats}, containing occurrence count of each feature 
294      """ 
295      counts = nx.zeros(nfeats, nx.int32) 
296      for docid in docids: 
297          counts[featdb[docid]] += 1 
298      return counts

299
```

  


| Trees | Indices | Help | | MScanner | | --- | |
| --- | --- | --- | --- | --- |

|  |  |
| --- | --- |
| Generated by Epydoc 3.0beta1 on Fri Nov 23 09:13:22 2007 | http://epydoc.sourceforge.net |
