# Supplementary material for: MScanner: a classifier for retrieving Medline citations
Source: BMC Bioinformatics. 2008 Feb 19;9:108. doi: 10.1186/1471-2105-9-108 (PMC2263023; doi:10.1186/1471-2105-9-108)
Supplement: Additional file 3 — Source code for MScanner. mscanner-20071123.zip is a ZIP archive containing the Python 2.5 source code for MScanner, licensed under the GNU General Public License. It also contains API documentation in HTML format. Updated versions will be made available at . [file 1471-2105-9-108-S3.zip › mscanner/help/api/mscanner.core.FeatureScores.FeatureScores-class.html]

xml version="1.0" encoding="ascii"?


mscanner.core.FeatureScores.FeatureScores


| Trees | Indices | Help | | MScanner | | --- | |
| --- | --- | --- | --- | --- |

|  |  |  |  |
| --- | --- | --- | --- |
| Package mscanner :: Package core :: Module FeatureScores :: Class FeatureScores | |  | | --- | | [hide private] | | [frames] | no frames] | |

# Class FeatureScores

source code  
  

```
object --+
         |
        FeatureScores
```

---

Feature score calculation and saving, with choice of calculation
method, and methods to exclude certain kinds of features.  
  


|  |  |  |  |
| --- | --- | --- | --- |
| |  |  | | --- | --- | | Instance Methods | [hide private] | | |
|  | |  |  | | --- | --- | | \_\_init\_\_(self, featmap, pseudocount=None, mask=None, make\_scores=`'``scores_bayes``'`, get\_postmask=None)  Initialise FeatureScores object (parameters are instance variables) | source code | |
|  | |  |  | | --- | --- | | scores\_of(self, featdb, pmids)  Calculate vector of scores given an iterable of PubMed IDs. | source code | |
|  | |  |  | | --- | --- | | \_\_len\_\_(self)  Number of features | source code | |
|  | |  |  | | --- | --- | | update(self, pos\_counts, neg\_counts, pdocs, ndocs, prior=None)  Change the feature counts and numbers of documents, clear old score calculations, and calculate new scores. | source code | |
|  | |  |  | | --- | --- | | scores\_bayes(s)  Document generated using multivariate Bernoulli distribution. | source code | |
|  | |  |  | | --- | --- | | scores\_noabsence(s)  Calculates document probability as product of log likelihood ratios, with pseudocount weight equal to one article. | source code | |
|  | |  |  | | --- | --- | | scores\_rubin(s)  Models document as product of log likelihood ratios, using MLE feature probabilities - replacing zeroes with 1e-8 | source code | |
|  | |  |  | | --- | --- | | \_make\_pseudovec(s)  Calculates a pseudocount vector based on background frequencies if no constant pseudocount was specified | source code | |
|  | |  |  | | --- | --- | | \_mask\_scores(self)  Set some feature scores to zero, effectively excluding them from consideration. | source code | |
|  | |  |  | | --- | --- | | mask\_nonpositives(s)  Mask for features not represented in the positives | source code | |
|  | |  |  | | --- | --- | | get\_best\_tfidfs(self, count)  Construct a table about the terms with the best TF.IDF | source code | |
|  | |  |  | | --- | --- | | write\_csv(self, stream)  Write features scores as CSV to an output stream | source code | |
| **Inherited from `object`**: `__delattr__`, `__getattribute__`, `__hash__`, `__new__`, `__reduce__`, `__reduce_ex__`, `__repr__`, `__setattr__`, `__str__` | |


|  |  |  |  |
| --- | --- | --- | --- |
| |  |  | | --- | --- | | Instance Variables | [hide private] | | |
| Set via constructor | |
|  | featmap  FeatureMapping object |
|  | get\_postmask  Method used to calculate a dynamic mask array once the feature scores are known. |
|  | make\_scores  Method used to calculate the feature scores. |
|  | mask  Either None or a boolean array to mask some features scores to zero (this is to exclude features by category, not by score). |
|  | pseudocount  Prior psuedocount to use for features, or None to use feature counts equal to Medline frequency. |
| Set by update | |
|  | ndocs  Number of negative documents |
|  | neg\_counts  Array of feature counts in negatives documents |
|  | pdocs  Number of positive documents |
|  | pos\_counts  Array of feature counts in positive documents |
|  | prior  Bayes prior to add to the score. |
| Set via make\_scores | |
|  | base  Value to be added to all article scores |
|  | nfreqs  Denominator of score fraction |
|  | pfreqs  Numerator of score fraction |
|  | scores  Score of each feature |


|  |  |  |  |
| --- | --- | --- | --- |
| |  |  | | --- | --- | | Properties | [hide private] | | |
|  | stats  A Storage instance with statistics about the features |
|  | tfidf  Vector of TF-IDF scores for each feature |
| **Inherited from `object`**: `__class__` | |


|  |  |  |  |
| --- | --- | --- | --- |
| |  |  | | --- | --- | | Method Details | [hide private] | | |

|  |  |  |
| --- | --- | --- |
| |  |  | | --- | --- | | \_\_init\_\_(self, featmap, pseudocount=None, mask=None, make\_scores=`'``scores_bayes``'`, get\_postmask=None)  *(Constructor)* | source code |  Initialise FeatureScores object (parameters are instance variables) Overrides: object.\_\_init\_\_ |

|  |  |  |
| --- | --- | --- |
| |  |  | | --- | --- | | scores\_of(self, featdb, pmids) | source code |  Calculate vector of scores given an iterable of PubMed IDs. Parameters:  - **`featdb`** - Mapping from PMID to feature vector - **`pmids`** - Iterable of keys into `featdb`  Returns:  Vector containing document scores corresponding to the pmids. |

|  |  |  |
| --- | --- | --- |
| |  |  | | --- | --- | | scores\_bayes(s) | source code |   Document generated using multivariate Bernoulli distribution. Feature non-occurrence is modeled as a base score for the document with no features, and an adjustment to the feature occurrence scores. |

|  |  |  |
| --- | --- | --- |
| |  |  | | --- | --- | | \_mask\_scores(self) | source code |  Set some feature scores to zero, effectively excluding them from consideration. Uses mask and get\_postmask |

|  |  |  |
| --- | --- | --- |
| |  |  | | --- | --- | | mask\_nonpositives(s) | source code |  Mask for features not represented in the positives Returns:  Boolean array for masked out features |

|  |  |  |
| --- | --- | --- |
| |  |  | | --- | --- | | get\_best\_tfidfs(self, count) | source code |  Construct a table about the terms with the best TF.IDF Parameters:  - **`count`** - Number of rows to return  Returns:  List of (Term ID, TFIDF, (term, term\_type), term score, pos count, neg count) |

  


|  |  |  |  |
| --- | --- | --- | --- |
| |  |  | | --- | --- | | Instance Variable Details | [hide private] | | |

|  |
| --- |
| priorBayes prior to add to the score. If None, estimate using the ratio of relevant to irrelevant articles in the data. |

  


|  |  |  |  |
| --- | --- | --- | --- |
| |  |  | | --- | --- | | Property Details | [hide private] | | |

|  |
| --- |
| stats A Storage instance with statistics about the features The following keys are present:  - pos\_occurrences: Total feature occurrences in positives - neg\_occurrences: Total feature occurrences in negatives - feats\_per\_pos: Number of features per positive article - feats\_per\_neg: Number of features per negative article - distinct\_feats: Number of distinct features - pos\_distinct\_feats: Number of of distinct features in   positives - neg\_distinct\_feats: Number of of distinct features in   negatives   Get Method:  *unreachable*.stats(self) - A Storage instance with statistics about the features |

|  |
| --- |
| tfidf Vector of TF-IDF scores for each feature Cache TF-IDF scores for terms, where for term frequency (TF) we treat the positive corpus as a single large document, but for inverse document frequency (IDF) each citation is a separate document. Get Method:  *unreachable*.tfidf(self) - Vector of TF-IDF scores for each feature |

  


| Trees | Indices | Help | | MScanner | | --- | |
| --- | --- | --- | --- | --- |

|  |  |
| --- | --- |
| Generated by Epydoc 3.0beta1 on Fri Nov 23 09:13:21 2007 | http://epydoc.sourceforge.net |
