# Supplementary material for: MScanner: a classifier for retrieving Medline citations
Source: BMC Bioinformatics. 2008 Feb 19;9:108. doi: 10.1186/1471-2105-9-108 (PMC2263023; doi:10.1186/1471-2105-9-108)
Supplement: Additional file 3 — Source code for MScanner. mscanner-20071123.zip is a ZIP archive containing the Python 2.5 source code for MScanner, licensed under the GNU General Public License. It also contains API documentation in HTML format. Updated versions will be made available at . [file 1471-2105-9-108-S3.zip › mscanner/help/api/mscanner.core.iofuncs-module.html]

xml version="1.0" encoding="ascii"?


mscanner.core.iofuncs


| Trees | Indices | Help | | MScanner | | --- | |
| --- | --- | --- | --- | --- |

|  |  |  |  |
| --- | --- | --- | --- |
| Package mscanner :: Package core :: Module iofuncs | |  | | --- | | [hide private] | | [frames] | no frames] | |

# Module iofuncs

source code  
  
I/O functions - for reading and writing certain file formats.  
  


---

**Author:**
Graham Poulter <http://graham.poulter.googlepages.com>

**Copyright:**
2007 Graham Poulter

**License:**
This program is free software: you can redistribute it and/or
modify it under the terms of the GNU General Public License as
published by the
Free Software Foundation, either version 3 of the License, or (at
your option)
any later version.
This program is distributed in the hope that it will be useful, but
WITHOUT ANY
WARRANTY; without even the implied warranty of MERCHANTABILITY or
FITNESS FOR A
PARTICULAR PURPOSE. See the GNU General Public License for more
details.
You should have received a copy of the GNU General Public License
along with
this program. If not, see <http://www.gnu.org/licenses/>.


|  |  |  |  |
| --- | --- | --- | --- |
| |  |  | | --- | --- | | Classes | [hide private] | | |
|  | FileTransaction  Transaction for Cheetah templates to output direct-to-file. |


|  |  |  |  |
| --- | --- | --- | --- |
| |  |  | | --- | --- | | Functions | [hide private] | | |
|  | |  |  | | --- | --- | | write\_pmids(filename, pmids)  Write list of PMIDs one per line to file | source code | |
|  | |  |  | | --- | --- | | read\_pmids(filename)  Yield PubMed IDs listed one per line in a file. | source code | |
|  | |  |  | | --- | --- | | read\_pmids\_array(filename)  Read array of PubMed IDs one per line from file | source code | |
|  | |  |  | | --- | --- | | read\_pmids\_careful(filename, include=None, exclude=`[``]`)  Reads array of PubMed IDs one per line, with checking. | source code | |
|  | |  |  | | --- | --- | | write\_lines(filename, items, desc=None, sep=`'``\t``'`)  Basic function for writing sequence of items to text files | source code | |
|  | |  |  | | --- | --- | | write\_scores(filename, pairs, sort=False)  Write scores and PubMed IDs to file | source code | |
|  | |  |  | | --- | --- | | read\_scores(filename)  Yield (score, pmid) pairs from file written by write\_scores | source code | |
|  | |  |  | | --- | --- | | read\_scores\_array(filename)  Reads a file written by write\_scores | source code | |
|  | |  |  | | --- | --- | | no\_valid\_pmids\_page(filename, dataset, pmids)  Print an error page when no valid PMIDs were found | source code | |
|  | |  |  | | --- | --- | | start\_logger(console=True, logfile=True)  Set up logging to file or console | source code | |
|  | |  |  | | --- | --- | | open\_logfile(filename, logname=`'``'`, mode=`'``a``'`)  Add a file handler to a logger using my default format. | source code | |
|  | |  |  | | --- | --- | | close\_logfile(handler, logname=`'``'`)  Remove and close a log file previously added with open\_logfile. | source code | |


|  |  |  |  |
| --- | --- | --- | --- |
| |  |  | | --- | --- | | Function Details | [hide private] | | |

|  |  |  |
| --- | --- | --- |
| |  |  | | --- | --- | | read\_pmids(filename) | source code |  Yield PubMed IDs listed one per line in a file. Empty lines and lines starting with # are ignored. |

|  |  |  |
| --- | --- | --- |
| |  |  | | --- | --- | | read\_pmids\_careful(filename, include=None, exclude=`[``]`) | source code |  Reads array of PubMed IDs one per line, with checking. Parameters:  - **`include`** - Only return members of this set (other PubMed IDs are   considered "broken"). - **`exclude`** - Do not return members of this set  Returns:  Arrays for result, broken and excluded PubMed IDs |

|  |  |  |
| --- | --- | --- |
| |  |  | | --- | --- | | write\_lines(filename, items, desc=None, sep=`'``\t``'`) | source code |  Basic function for writing sequence of items to text files Parameters:  - **`filename`** - Name of file to write to - **`items`** - Sequence of items convertible using str(). Tuples are written   as separated values. - **`desc`** - Optional string to write at the top of the file - **`sep`** - Separator for values |

|  |  |  |
| --- | --- | --- |
| |  |  | | --- | --- | | write\_scores(filename, pairs, sort=False) | source code |  Write scores and PubMed IDs to file Parameters:  - **`pairs`** - Iterable over (score, PMID) - **`sort`** - If True, write them in decreasing order of score |

|  |  |  |
| --- | --- | --- |
| |  |  | | --- | --- | | read\_scores\_array(filename) | source code |  Reads a file written by write\_scores Parameters:  - **`filename`** - Path to file from which to read the pmid,score  Returns:  An array of PubMed IDs, and an array of scores |

|  |  |  |
| --- | --- | --- |
| |  |  | | --- | --- | | no\_valid\_pmids\_page(filename, dataset, pmids) | source code |  Print an error page when no valid PMIDs were found Parameters:  - **`filename`** - Path to output file - **`pmids`** - List of any provided PMIDs (all invalid) |

|  |  |  |
| --- | --- | --- |
| |  |  | | --- | --- | | start\_logger(console=True, logfile=True) | source code |  Set up logging to file or console Parameters:  - **`console`** - If True, log to the console. - **`logfile`** - If True, log to rc.logfile. |

|  |  |  |
| --- | --- | --- |
| |  |  | | --- | --- | | open\_logfile(filename, logname=`'``'`, mode=`'``a``'`) | source code |  Add a file handler to a logger using my default format. Parameters:  - **`filename`** - File to write to - **`logname`** - Name of log to write to (default '') - **`mode`** - File open mode (default 'a')  Returns:  The logging.FileHandler instance |

|  |  |  |
| --- | --- | --- |
| |  |  | | --- | --- | | close\_logfile(handler, logname=`'``'`) | source code |  Remove and close a log file previously added with open\_logfile. Does nothing if called a second time. Parameters:  - **`handler`** - FileHandler instance to remove - **`logname`** - Name of the logger (defaults to '') |

  


| Trees | Indices | Help | | MScanner | | --- | |
| --- | --- | --- | --- | --- |

|  |  |
| --- | --- |
| Generated by Epydoc 3.0beta1 on Fri Nov 23 09:13:20 2007 | http://epydoc.sourceforge.net |
