# Supplementary material for: MScanner: a classifier for retrieving Medline citations
Source: BMC Bioinformatics. 2008 Feb 19;9:108. doi: 10.1186/1471-2105-9-108 (PMC2263023; doi:10.1186/1471-2105-9-108)
Supplement: Additional file 3 — Source code for MScanner. mscanner-20071123.zip is a ZIP archive containing the Python 2.5 source code for MScanner, licensed under the GNU General Public License. It also contains API documentation in HTML format. Updated versions will be made available at . [file 1471-2105-9-108-S3.zip › mscanner/help/api/mscanner.core.iofuncs-pysrc.html]

xml version="1.0" encoding="ascii"?


mscanner.core.iofuncs


| Trees | Indices | Help | | MScanner | | --- | |
| --- | --- | --- | --- | --- |

|  |  |  |  |
| --- | --- | --- | --- |
| Package mscanner :: Package core :: Module iofuncs | |  | | --- | | [hide private] | | [frames] | no frames] | |

# Source Code for Module mscanner.core.iofuncs

```
  1  """I/O functions - for reading and writing certain file formats.""" 
  2   
  3  from __future__ import with_statement 
  4  from __future__ import division 
  5   
  6  import numpy as nx 
  7  from itertools import izip 
  8   
  9  __copyright__ = "2007 Graham Poulter" 
 10  __author__ = "Graham Poulter <http://graham.poulter.googlepages.com>" 
 11  __license__ = """This program is free software: you can redistribute it and/or 
 12  modify it under the terms of the GNU General Public License as published by the 
 13  Free Software Foundation, either version 3 of the License, or (at your option) 
 14  any later version. 
 15   
 16  This program is distributed in the hope that it will be useful, but WITHOUT ANY 
 17  WARRANTY; without even the implied warranty of MERCHANTABILITY or FITNESS FOR A 
 18  PARTICULAR PURPOSE. See the GNU General Public License for more details. 
 19   
 20  You should have received a copy of the GNU General Public License along with 
 21  this program. If not, see <http://www.gnu.org/licenses/>.""" 
 22   
 23   


24 -def write_pmids(filename, pmids):


25      """Write list of PMIDs one per line to file""" 
 26      with open(filename, "w") as f: 
 27          f.write("\n".join(str(s) for s in pmids))

 28   
 29   


30 -def read_pmids(filename):


31      """Yield PubMed IDs listed one per line in a file. Empty lines and lines 
 32      starting with # are ignored.""" 
 33      with open(filename) as f: 
 34          for line in f: 
 35              sline = line.strip() 
 36              if sline == "" or sline.startswith("#"): 
 37                  continue 
 38              yield int(sline.split()[0])

 39   
 40   


41 -def read_pmids_array(filename):


42      """Read array of PubMed IDs one per line from file""" 
 43      return nx.array(read_pmids(filename), nx.int32)

 44   
 45   


46 -def read_pmids_careful(filename, include=None, exclude=[]):


47      """Reads array of PubMed IDs one per line, with checking. 
 48   
 49      @param include: Only return members of this set (other PubMed IDs are 
 50      considered "broken"). 
 51   
 52      @param exclude: Do not return members of this set 
 53       
 54      @return: Arrays for result, broken and excluded PubMed IDs 
 55      """ 
 56      results = [] 
 57      broken = [] 
 58      excluded = [] 
 59      for pmid in read_pmids(filename): 
 60          if include is not None and pmid not in include: 
 61              broken.append(pmid) 
 62          elif pmid in exclude: 
 63              excluded.append(pmid) 
 64          else: 
 65              results.append(pmid) 
 66      return tuple(nx.array(a, nx.int32) for a in [results,broken,excluded])

 67   
 68   


69 -def write_lines(filename, items, desc=None, sep="\t"):


70      """Basic function for writing sequence of items to text files 
 71       
 72      @param filename: Name of file to write to 
 73   
 74      @param items: Sequence of items convertible using str().  Tuples 
 75      are written as separated values. 
 76       
 77      @param desc: Optional string to write at the top of the file 
 78   
 79      @param sep: Separator for values 
 80      """ 
 81      with open(filename, "w") as f: 
 82          if desc is not None: 
 83              f.write(desc.strip()+"\n") 
 84          for item in items: 
 85              if hasattr(item, "__iter__"): 
 86                  f.write(sep.join([str(x) for x in item])) 
 87              else: 
 88                  f.write(str(item)) 
 89              f.write("\n")

 90   
 91   


92 -def write_scores(filename, pairs, sort=False):


93      """Write scores and PubMed IDs to file 
 94       
 95      @param pairs: Iterable over (score, PMID) 
 96   
 97      @param sort: If True, write them in decreasing order of score 
 98      """ 
 99      sorted_pairs = sorted(pairs, reverse=True) if sort else pairs 
100      filename.write_lines("%-10d %f" % (p,s) for s,p in sorted_pairs)

101   
102   


103 -def read_scores(filename):


104      """Yield (score, pmid) pairs from file written by L{write_scores}""" 
105      with open(filename, "r") as f: 
106          for line in f: 
107              sline = line.strip() 
108              if sline == "" or sline.startswith("#"): 
109                  continue 
110              splits = sline.split() 
111              yield float(splits[1]), int(splits[0])

112       
113   


114 -def read_scores_array(filename):


115      """Reads a file written by L{write_scores} 
116       
117      @param filename: Path to file from which to read the pmid,score 
118   
119      @return: An array of PubMed IDs, and an array of scores""" 
120      scores, pmids = izip(*read_scores(filename)) 
121      return nx.array(pmids,nx.int32), nx.array(scores,nx.float32)

122   
123   


124 -def no_valid_pmids_page(filename, dataset, pmids):


125      """Print an error page when no valid PMIDs were found 
126       
127      @param filename: Path to output file 
128   
129      @param pmids: List of any provided PMIDs (all invalid) 
130      """ 
131      from Cheetah.Template import Template 
132      from mscanner.configuration import rc 
133      with FileTransaction(filename, "w") as ft: 
134          page = Template(file=str(rc.templates/"notfound.tmpl")) 
135          page.dataset = dataset 
136          page.notfound_pmids = pmids 
137          page.respond(ft)

138   
139   


140 -class FileTransaction(file):


141      """Transaction for Cheetah templates to output direct-to-file. 
142       
143      Cheetah defaults to DummyTransaction which creates a huge list and 
144      joins them up to create a string.  This is way slower than writing to 
145      file directly. 
146       
147      Usage:: 
148          with FileTransaction("something.html","wb") as ft: 
149              Template().respond(ft) 
150      """ 
151       


152 -    def __init__(self, *args, **kw):


153          """Open the file, same parameters as for the builtin""" 
154          file.__init__(self, *args, **kw) 
155          self.response = self

156   


157 -    def writeln(self):


158          """Write a line of output""" 
159          self.write(txt) 
160          self.write('\n')

161   


162 -    def getvalue(self):


163          """Not implemented""" 
164          return None

165   


166 -    def __call__(self):


167          return self

168   
169   
170   


171 -def start_logger(console=True, logfile=True):


172      """Set up logging to file or console 
173      @param console: If True, log to the console. 
174      @param logfile: If True, log to rc.logfile.""" 
175      # Configure the root logger to print everything 
176      from mscanner.configuration import rc 
177      import logging 
178      rootlog = logging.getLogger() 
179      rootlog.setLevel(logging.DEBUG) 
180      format = logging.Formatter("%(asctime)-9s %(levelname)-8s %(message)s", "%H:%M:%S") 
181      # Configure primary file logger 
182      if logfile: 
183          filelog = logging.FileHandler(rc.logfile, "a") 
184          filelog.setFormatter(format) 
185          rootlog.addHandler(filelog) 
186      # Configure logging to console 
187      if console: 
188          console = logging.StreamHandler() 
189          console.setFormatter(format) 
190          rootlog.addHandler(console)

191   
192   


193 -def open_logfile(filename, logname="", mode="a"):


194      """Add a file handler to a logger using my default format. 
195      @param filename: File to write to 
196      @param logname: Name of log to write to (default '') 
197      @param mode: File open mode (default 'a') 
198      @return: The logging.FileHandler instance 
199      """ 
200      import logging 
201      logger = logging.getLogger(logname) 
202      handler = logging.FileHandler(filename, mode) 
203      handler.setFormatter(logging.Formatter( 
204          "%(asctime)-9s %(levelname)-8s %(message)s", "%H:%M:%S")) 
205      logger.addHandler(handler) 
206      return handler

207   
208   


209 -def close_logfile(handler, logname=""):


210      """Remove and close a log file previously added with L{open_logfile}.  Does 
211      nothing if called a second time. 
212      @param handler: FileHandler instance to remove 
213      @param logname: Name of the logger (defaults to '') 
214      """ 
215      import logging 
216      logger = logging.getLogger(logname) 
217      if handler in logger.handlers: 
218          logger.removeHandler(handler) 
219      if not handler.stream.closed: 
220          handler.close()

221
```

  


| Trees | Indices | Help | | MScanner | | --- | |
| --- | --- | --- | --- | --- |

|  |  |
| --- | --- |
| Generated by Epydoc 3.0beta1 on Fri Nov 23 09:13:25 2007 | http://epydoc.sourceforge.net |
