# Supplementary material for: MScanner: a classifier for retrieving Medline citations
Source: BMC Bioinformatics. 2008 Feb 19;9:108. doi: 10.1186/1471-2105-9-108 (PMC2263023; doi:10.1186/1471-2105-9-108)
Supplement: Additional file 3 — Source code for MScanner. mscanner-20071123.zip is a ZIP archive containing the Python 2.5 source code for MScanner, licensed under the GNU General Public License. It also contains API documentation in HTML format. Updated versions will be made available at . [file 1471-2105-9-108-S3.zip › mscanner/help/api/mscanner.core.iofuncs.FileTransaction-class.html]

xml version="1.0" encoding="ascii"?


mscanner.core.iofuncs.FileTransaction


| Trees | Indices | Help | | MScanner | | --- | |
| --- | --- | --- | --- | --- |

|  |  |  |  |
| --- | --- | --- | --- |
| Package mscanner :: Package core :: Module iofuncs :: Class FileTransaction | |  | | --- | | [hide private] | | [frames] | no frames] | |

# Class FileTransaction

source code  
  

```
object --+    
         |    
      file --+
             |
            FileTransaction
```

---

Transaction for Cheetah templates to output direct-to-file.

Cheetah defaults to DummyTransaction which creates a huge list and
joins them up to create a string. This is way slower than writing to
file directly.

Usage:

```
   with FileTransaction("something.html","wb") as ft:
       Template().respond(ft)
```

  
  


|  |  |  |  |
| --- | --- | --- | --- |
| |  |  | | --- | --- | | Instance Methods | [hide private] | | |
|  | |  |  | | --- | --- | | \_\_init\_\_(self, \*args, \*\*kw)  Open the file, same parameters as for the builtin | source code | |
|  | |  |  | | --- | --- | | writeln(self)  Write a line of output | source code | |
|  | |  |  | | --- | --- | | getvalue(self)  Not implemented | source code | |
|  | |  |  | | --- | --- | | \_\_call\_\_(self) | source code | |
| **Inherited from `file`**: `__delattr__`, `__enter__`, `__exit__`, `__getattribute__`, `__iter__`, `__new__`, `__repr__`, `__setattr__`, `close`, `fileno`, `flush`, `isatty`, `next`, `read`, `readinto`, `readline`, `readlines`, `seek`, `tell`, `truncate`, `write`, `writelines`, `xreadlines`  **Inherited from `object`**: `__hash__`, `__reduce__`, `__reduce_ex__`, `__str__` | |


|  |  |  |  |
| --- | --- | --- | --- |
| |  |  | | --- | --- | | Properties | [hide private] | | |
| **Inherited from `file`**: `closed`, `encoding`, `mode`, `name`, `newlines`, `softspace`  **Inherited from `object`**: `__class__` | |


|  |  |  |  |
| --- | --- | --- | --- |
| |  |  | | --- | --- | | Method Details | [hide private] | | |

|  |  |  |
| --- | --- | --- |
| |  |  | | --- | --- | | \_\_init\_\_(self, \*args, \*\*kw)  *(Constructor)* | source code |  Open the file, same parameters as for the builtin Returns:  ``` file object ```  Overrides: file.\_\_init\_\_ |

  


| Trees | Indices | Help | | MScanner | | --- | |
| --- | --- | --- | --- | --- |

|  |  |
| --- | --- |
| Generated by Epydoc 3.0beta1 on Fri Nov 23 09:13:21 2007 | http://epydoc.sourceforge.net |
