# Supplementary material for: MScanner: a classifier for retrieving Medline citations
Source: BMC Bioinformatics. 2008 Feb 19;9:108. doi: 10.1186/1471-2105-9-108 (PMC2263023; doi:10.1186/1471-2105-9-108)
Supplement: Additional file 3 — Source code for MScanner. mscanner-20071123.zip is a ZIP archive containing the Python 2.5 source code for MScanner, licensed under the GNU General Public License. It also contains API documentation in HTML format. Updated versions will be made available at . [file 1471-2105-9-108-S3.zip › mscanner/help/api/mscanner.core.metrics-pysrc.html]

xml version="1.0" encoding="ascii"?


mscanner.core.metrics


| Trees | Indices | Help | | MScanner | | --- | |
| --- | --- | --- | --- | --- |

|  |  |  |  |
| --- | --- | --- | --- |
| Package mscanner :: Package core :: Module metrics | |  | | --- | | [hide private] | | [frames] | no frames] | |

# Source Code for Module mscanner.core.metrics

```
  1  """Calculates performance statistics given the scores of the positive and 
  2  negative citations 
  3   
  4  @note: Order of confusion matrix is *always* TP, TN, FP, FN (do not mess up on 
  5  this!) 
  6   
  7  """ 
  8   
  9  from __future__ import division 
 10  import numpy as nx 
 11  import copy # used in PerformanceRanges 
 12   
 13  from mscanner import update 
 14  from mscanner.core.Validator import CrossValidator 
 15   
 16  __copyright__ = "2007 Graham Poulter" 
 17  __author__ = "Graham Poulter <http://graham.poulter.googlepages.com>" 
 18  __license__ = """This program is free software: you can redistribute it and/or 
 19  modify it under the terms of the GNU General Public License as published by the 
 20  Free Software Foundation, either version 3 of the License, or (at your option) 
 21  any later version. 
 22   
 23  This program is distributed in the hope that it will be useful, but WITHOUT ANY 
 24  WARRANTY; without even the implied warranty of MERCHANTABILITY or FITNESS FOR A 
 25  PARTICULAR PURPOSE. See the GNU General Public License for more details. 
 26   
 27  You should have received a copy of the GNU General Public License along with 
 28  this program. If not, see <http://www.gnu.org/licenses/>.""" 
 29   
 30   


31 -class PerformanceVectors:


32      """Contains vectors of performance metrics at all possible threshold 
 33       
 34      @note: The performance statistics are calculated at every discreet 
 35      threshold and stored in vectors. 
 36       
 37      @note: This copies L{pscores} and L{nscores} *before* sorting! 
 38   
 39      @note: If L{utility_r} is None, we use ratio of negatives to positives in the data. 
 40       
 41      @group Passed to constructor: pscores, nscores, alpha, utility_r 
 42      @ivar pscores: Vector of scores for positive articles, in increasing order. 
 43      @ivar nscores: Vector of scores for negative articles, in increasing order. 
 44      @ivar alpha: Balance of recall and precision in the F measure. 
 45      @ivar utility_r: Value of a relevant article (irrelevant articles have 
 46      value -1). 
 47   
 48      @group From _confusion_vectors: uscores, PE, NE, TP, TN, FP, FN 
 49      @ivar uscores: Unique scores in increasing order. 
 50      @ivar PE: Number of positives with each score in L{uscores}. 
 51      @ivar NE: Number of negatives with each score in L{uscores}. 
 52      @ivar TP, TN, FP, FN: Vectors for confusion matrix at each distinct threshold. 
 53       
 54      @group From _ratio_vectors: TPR, FPR, PPV, FM, FMa, U 
 55      @ivar TPR: True positive rate at each threshold 
 56      @ivar FPR: False positive rate at each threshold 
 57      @ivar PPV: Positive predictive value at each threshold 
 58      @ivar FM: F measure at each threshold using alpha 
 59      @ivar FMa: F measure at each threshold using given alpha 
 60      @ivar U: Utility at each threshold 
 61           
 62      @group From _curve_areas: ROC_area, PR_area 
 63      @ivar ROC_area: Area under ROC curve (trapezoidal under-estimate) 
 64      @ivar PR_area: Aread under precision-recall curve. 
 65   
 66      @group From _roc_error: W, W_stderr 
 67      @ivar W: Area under ROC curve (better than trapezoidal area) 
 68      @ivar W_stderr: Standard error of area under ROC curve. 
 69   
 70      @group From _averaged_precision: AvPrec 
 71      @ivar AvPrec: Averaged precision (better than trapezoidal area) 
 72   
 73      @group From _breakeven: bep_index, breakeven 
 74      @ivar bep_index: Index into L{uscores} for break-even point.  
 75      @ivar breakeven: Value at the point where precision=recall. 
 76      """ 
 77   
 78   


79 -    def __init__(self, pscores, nscores, alpha, utility_r=None):


80          pscores = pscores.copy() 
 81          nscores = nscores.copy() 
 82          pscores.sort() 
 83          nscores.sort() 
 84          if utility_r is None: 
 85              utility_r = len(nscores)/len(pscores) 
 86          update(self, locals()) 
 87          self._confusion_vectors() 
 88          self._ratio_vectors(self.alpha) 
 89          self._curve_areas() 
 90          self._roc_error() 
 91          self._averaged_precision() 
 92          self._breakeven()

 93   
 94   


95 -    def _confusion_vectors(self):


96          """Calculates confusion matrix counts by iterating over pscores 
 97           
 98          Sets L{uscores}, L{PE}, L{NE}, L{TP}, L{TN}, L{FP}, L{FN} 
 99          """ 
100          s = self 
101          self.uscores = nx.unique(nx.concatenate((s.pscores,s.nscores))) 
102          vlen = len(s.uscores) 
103          P = len(s.pscores) 
104          N = len(s.nscores) 
105          self.PE = nx.zeros(vlen, nx.float32) # positives with given score 
106          self.NE = nx.zeros(vlen, nx.float32) # negatives with given score 
107          self.TP = nx.zeros(vlen, nx.float32) # true positives 
108          self.TN = nx.zeros(vlen, nx.float32) # true negatives 
109          self.FP = nx.zeros(vlen, nx.float32) # false positives 
110          self.FN = nx.zeros(vlen, nx.float32) # false negatives 
111          TN = 0 
112          FN = 0 
113          for idx, threshold in enumerate(s.uscores): 
114   
115              # Classify positives scoring < threshold as negative 
116              # (look up score for the next article to classify) 
117              while (FN < P) and (s.pscores[FN] < threshold): 
118                  FN += 1 
119              TP = P - FN # TP+FN=P 
120   
121              # pcount-FN = number of positives having threshold score 
122              pcount = FN # Start at FN, subtract it later 
123              while (pcount < P) and s.pscores[pcount] == threshold: 
124                  pcount += 1 
125              s.PE[idx] = pcount-FN 
126               
127              # Classify negatives scoring < threshold as negative 
128              while (TN < N) and (s.nscores[TN] < threshold): 
129                  TN += 1 
130              FP = N - TN  # TN+FP=N 
131   
132              # ncount-TN = number of negatives having threshold score 
133              ncount = TN # Start at TN and subtract it later 
134              while (ncount < N) and s.nscores[ncount] == threshold: 
135                  ncount += 1 
136              s.NE[idx] = ncount-TN 
137               
138              s.TP[idx] = TP 
139              s.TN[idx] = TN 
140              s.FP[idx] = FP 
141              s.FN[idx] = FN

142   
143   


144 -    def _ratio_vectors(self, alpha):


145          """Calculate performance using vector algebra 
146   
147          @param alpha: Weight of precision in calculating FMa 
148           
149          Sets L{TPR}, L{FPR}, L{PPV}, L{FM}, L{FMa}, L{U} 
150          """ 
151          s = self 
152          # TPR is recall 
153          self.TPR = s.TP / len(s.pscores) 
154          # FPR is 1-specificity 
155          self.FPR = s.FP / len(s.nscores) 
156          # PPV is precision 
157          self.PPV = s.TP / (s.TP + s.FP)  
158          self.PPV[s.TP+s.FP == 0] = 1.0 
159          # FM is F-Measure 
160          self.FM = 2 * s.TPR * s.PPV / (s.TPR + s.PPV)  
161          # FMa is the alpha-weighted F-Measures 
162          self.FMa = 1.0 / (alpha / s.PPV + (1 - alpha) / s.TPR) 
163          # U is the utility function 
164          self.U = (s.utility_r * s.TP - s.FP) / (self.utility_r * len(s.pscores))

165   
166   


167 -    def _curve_areas(self):


168          """Calculate areas under ROC and precision-recall curves 
169           
170          Uses trapz(y, x). TPR is decreasing as threshold climbs, so vectors 
171          have to be reversed. 
172           
173          This method underestimates ROC areas because boundary points (0,0) and 
174          (1,1) usually are not present in the data. Better to use L{_roc_error} 
175          which does not have that problem. 
176           
177          Sets L{ROC_area}, L{PR_area}""" 
178          from scipy.integrate import trapz 
179          self.ROC_area = trapz(self.TPR[::-1], self.FPR[::-1]) 
180          self.PR_area = trapz(self.PPV[::-1], self.TPR[::-1])

181   
182   


183 -    def _mergescores(self):


184          """Merges pscores and nscores in a single pass 
185           
186          @note: L{nscores} and L{pscores} must be in increasing order of score.         
187           
188          @return: Iterator over (score, relevance) in decreasing order of score. 
189          Relevance is True for members of pscores, and False for members of 
190          nscores. """ 
191          s = self 
192          p_idx = len(s.pscores)-1 
193          n_idx = len(s.nscores)-1 
194          while p_idx >= 0 or n_idx >= 0: 
195              if p_idx >= 0 and \ 
196              (n_idx < 0 or s.pscores[p_idx] >= s.nscores[n_idx]): 
197                  yield s.pscores[p_idx], True 
198                  p_idx -= 1 
199              elif n_idx >= 0 and \ 
200              (p_idx < 0 or s.nscores[n_idx] > s.pscores[p_idx]): 
201                  yield s.nscores[n_idx], False 
202                  n_idx -= 1

203   
204   


205 -    def _averaged_precision(self):


206          """Average the precision over each point of recall 
207           
208          Sets L{AvPrec}, which is precision averaged over each point where a 
209          relevant document is returned""" 
210          AvPrec = 0.0 
211          TP = 0 
212          FP = 0 
213          for score, relevant in self._mergescores(): 
214              if relevant: 
215                  TP += 1 
216                  AvPrec += TP/(TP+FP) 
217              else: 
218                  FP += 1 
219          self.AvPrec = AvPrec/TP

220   
221   


222 -    def _roc_error(self):


223          """Area under ROC and its standard error 
224           
225          Uses method of Hanley1982 to calculate standard error on the Wilcoxon 
226          statistic W, which corresponds to the area under the ROC by trapezoidal 
227          rule. 
228           
229          @note: The vectors r1 .. r7 correspond to rows of Table II in 
230          Hanley1982. 
231   
232          Sets L{W} and L{W_stderr} 
233          """ 
234          s = self 
235          # r1 is number of negatives with each score, 
236          # r2 is number of positives rated higher than each score 
237          # r3 is number of positives with each score 
238          # r4 is number of negatives rated lower than each score 
239          r1 = s.NE 
240          r2 = s.TP - s.PE 
241          r3 = s.PE 
242          r4 = s.TN 
243          r5 = r1 * r2 + 0.5 * r1 * r3 
244          r6 = r3 * (r4**2 + r4*r1 + (r1**2)/3) 
245          r7 = r1 * (r2**2 + r2*r3 + (r3**2)/3) 
246          N = float(len(s.nscores)) 
247          P = float(len(s.pscores)) 
248          W = r5.sum() / (N*P) 
249          Q2 = r6.sum() / (P * N**2) 
250          Q1 = r7.sum() / (N * P**2) 
251          W_stderr = nx.sqrt((W*(1-W)+(P-1)*(Q1-W**2)+(N-1)*(Q2-W**2))/(P*N)) 
252          #print W, Q1, Q2, W_stderr 
253          self.W = W 
254          self.W_stderr = W_stderr

255   
256   


257 -    def _breakeven(self):


258          """Calculate break-even point where precision equals recall. 
259          Sets L{breakeven}, L{bep_index} 
260          """ 
261          s = self 
262          diff = nx.absolute(nx.subtract(s.TPR, s.PPV)) 
263          self.bep_index = nx.nonzero(diff == nx.min(diff))[0][0] 
264          self.breakeven = 0.5*(s.TPR[s.bep_index]+s.PPV[s.bep_index])

265   
266   


267 -    def threshold_maximising(self, vector):


268          """Find threshold to maximise the given vector 
269          @return: The threshold score, and its index in L{uscores}""" 
270          idx = nx.nonzero(vector == nx.max(vector))[0][0] 
271          return self.uscores[idx], idx

272   
273   


274 -    def index_for(self, threshold):


275          """Calculate index into L{uscores} corresponding to given threshold. 
276          @return: The highest available threshold less than the specified one, 
277          and its index in L{uscores}.""" 
278          diffs = self.uscores - threshold 
279          idx = nx.nonzero(diffs == nx.min(diffs[diffs >= 0]))[0][0] 
280          return self.uscores[idx], idx

281   
282   


283 -    def matrix_for(self, index):


284          """Get confusion matrix at a threshold index into L{uscores}. 
285          @return: TP, TN, FP, FN representing the confusion matrix.""" 
286          TP = int(self.TP[index]) 
287          TN = int(self.TN[index]) 
288          FP = int(self.FP[index]) 
289          FN = int(self.FN[index]) 
290          return TP, TN, FP, FN

291   
292   


293 -    def metrics_for(self, index):


294          """Get L{PerformanceMetrics} at a threshold index into L{uscores}.""" 
295          TP, TN, FP, FN = self.matrix_for(index) 
296          return PerformanceMetrics(TP, TN, FP, FN, self.alpha, self.utility_r)

297   
298   
299   


300 -class PerformanceMetrics:


301      """Performance metrics derived from a particular confusion matrix. 
302       
303      @note: L{PerformanceRange} depends on all attributes being 
304      numerical (so that comparison operators work). 
305       
306      @group TP, TN, FP, FN, alpha, utility_r: Passed to constructor. 
307       
308      @ivar TP, TN, FP, FN: Confusion matrix. 
309   
310      @ivar alpha: Weight of precision in F measure calculation. 
311       
312      @ivar utility_r: Weight of a true positive (false positive is -1) (if 
313      None we use N/P). 
314       
315      @ivar P: Number of relevant items. 
316      @ivar N: Number of irrelevant items. 
317      @ivar A: Total number of items (P+N). 
318      @ivar T: Number of correct classifications. 
319      @ivar F: Number of incorrect classifications. 
320      @ivar TPR, FNR, TNR, FPR: Confusion matrix ratios. 
321      @ivar PPV, NPV: Positive and negative predictive value. 
322      @ivar accuracy: Equals T/A. 
323      @ivar enrichment: Equals precision/prevalence. 
324      @ivar error: Equals F/A. 
325      @ivar fmeasure: Harmonic mean of TPR and PPV [alpha=0.5] 
326      @ivar fmeasure_alpha: Alpha-weighted F measure [alpha!=0.5] 
327      @ivar precision: Equals PPV. 
328      @ivar prevalence: Equals P/A. 
329      @ivar recall: Equals TPR. 
330      @ivar specificity: Equals TNR. 
331      @ivar fp_tp_ratio: Equals FP/TP. 
332      """ 
333       


334 -    def __init__(self, TP, TN, FP, FN, alpha=0.5, utility_r=None):


335          # Sums of the confusion matrix 
336          P = TP + FN 
337          N = TN + FP 
338          A = P + N 
339          T = TP + TN 
340          F = FP + FN 
341          # Performance ratios 
342          TPR = TP/P if P > 0 else 0 # TPR = TP/P = sensitivity = recall 
343          FNR = FN/P if P > 0 else 0 # FNR = FN/P = 1-TP/P = 1-sensitivity = 1-recall 
344          TNR = TN/N if N > 0 else 0 # TNR = TN/N = specificity 
345          FPR = FP/N if N > 0 else 0 # FPR = FP/N = 1 - TN/N = 1-specificity 
346          PPV = TP/(TP+FP) if TP+FP>0 else 1 # PPV=precision 
347          NPV = TN/(TN+FN) if TN+FN>0 else 1 
348          # Derived performance ratios 
349          recall = TPR 
350          specificity = TNR 
351          precision = PPV 
352          accuracy = T/A if A > 0 else 0 
353          prevalence = P/A if A > 0 else 0 
354          fp_tp_ratio = FP/TP if TP > 0 else 0 
355          FDR = 1 - precision 
356          error = 1 - accuracy 
357          enrichment = precision / prevalence if prevalence > 0 else 0 
358          # Utility measure 
359          if utility_r is None: 
360              utility_r = N/P 
361          utility = (utility_r * TP - FP) / (utility_r * P) 
362          # F measure 
363          fmeasure, fmeasure_alpha = 0, 0 
364          if recall > 0 and precision > 0: 
365              fmeasure = 2 * TPR * PPV / (TPR + PPV) 
366              fmeasure_alpha = 1.0 / ((alpha/PPV) + ((1-alpha)/TPR)) 
367          update(self, locals())

368   
369   
370   


371 -class PredictedMetrics:


372      """Predict the performance metrics vectors for query results 
373      knowing only the true and false positive rates in testing. 
374       
375      This also requires specifiying the size of the database against 
376      which the query will be performed, and also the number of relevant 
377      documents guessed to be present in the database. 
378   
379      @group Passed to constructor: TPR, FPR, thresholds, relevant, total 
380      @ivar TPR: True Positive Rate in test corpus at each threshold (increasing) 
381      @ivar FPR: False Positive Rate in test corpus at each threshold (increasing) 
382      @ivar thresholds: Threshold scores corresponding to TPR and FPR (decreasing) 
383      @ivar relevant: Number of relevant articles in database 
384      @ivar total: Total number of articles in database 
385       
386      @group Calculated in constructor: prevalence, TP, FP, results, precision 
387      @ivar prevalence: Fraction of relevant articles in database 
388      @ivar TP: Predicted number of true positives at each threshold 
389      @ivar FP: Predicted number of false positives at each threshold 
390      @ivar results: Predicted number of results at each threshold (TP+FP) 
391      @ivar PPV: Predicted Precision (positive predictive value) at each threshold 
392      """ 
393       


394 -    def __init__(self, TPR, FPR, thresholds, relevant, total):


395          """Constructor, calculates the predicted statistics""" 
396          prevalence = relevant / total 
397          TP = TPR * relevant 
398          FP = FPR * (total - relevant) 
399          results = TP + FP 
400          PPV = TP / results 
401          update(self, locals())

402   
403   
404   


405 -class PerformanceRange:


406      """Given a threshold, find the minimum and maximum for the precision, 
407      recall across the validation folds. 
408   
409      @group Passed via constructor: pscores, nscores, nfolds, threshold, average 
410      @ivar pscores: Unsorted scores of positive documents. 
411      @ivar nscores: Unsorted scores of negative documents. 
412      @ivar nfolds: Number of cross validation folds 
413      @ivar threshold: Predict documents above this score to be positive. 
414      @ivar average: L{PerformanceMetrics} globally estimated using all folds 
415       
416      @ivar TP: Vector for number of TP in each fold 
417      @ivar TN: Vector for number of TN in each fold 
418      @ivar FP: Vector for number of FP in each fold 
419      @ivar FN: Vector for number of FN in each fold 
420       
421      @ivar minimum: L{PerformanceMetrics} with the miminum values across folds 
422      @ivar maximum: L{PerformanceMetrics} with the maximum values across folds 
423      """ 
424       


425 -    def __init__(self, pscores, nscores, nfolds, threshold, average):


426          """Parameters correspond to instance variables""" 
427          minimum = copy.copy(average) 
428          maximum = copy.copy(average) 
429          update(self, locals()) 
430          self._make_confusion_vectors() 
431          self._calculate_min_max()

432   
433   


434 -    def _calculate_min_max(self):


435          """Finds (min,max) of precision, etc., using the TP/TN/FP/FN vectors 
436          over the folds.""" 
437          for TP, TN, FP, FN in zip(self.TP, self.TN, self.FP, self.FN): 
438              metrics = PerformanceMetrics( 
439                  TP, TN, FP, FN, self.average.alpha, self.average.utility_r) 
440              for key, value in metrics.__dict__.iteritems(): 
441                  if value < getattr(self.minimum, key): 
442                      setattr(self.minimum, key, value) 
443                  if value > getattr(self.maximum, key): 
444                      setattr(self.maximum, key, value)

445   
446   


447 -    def _make_confusion_vectors(self):


448          """Finds TP, TN, FP, FN at the threshold over each validation fold""" 
449          for vname in ["TP", "TN", "FP", "FN"]: 
450              setattr(self, vname, nx.zeros(self.nfolds, nx.float32)) 
451          # Recreate the validation partitions in the score vectors 
452          pstarts, psizes = CrossValidator.make_partitions( 
453              len(self.pscores), self.nfolds) 
454          nstarts, nsizes = CrossValidator.make_partitions( 
455              len(self.nscores), self.nfolds) 
456          # Calculate confusion matrix within each vector 
457          for fold, (pstart,psize,nstart,nsize) in \ 
458              enumerate(zip(pstarts,psizes,nstarts,nsizes)): 
459              self._confusion_matrix(fold,  
460                  self.pscores[pstart:pstart+psize], 
461                  self.nscores[nstart:nstart+nsize])

462   
463       


464 -    def _confusion_matrix(self, fold, pos, neg):


465          """Find TP, TN, FP, FN at threshold inside a single validation fold. 
466          @param fold: Number of the cross validation fold 
467          @param pos: Scores for relevant articles in the fold 
468          @param neg: Scores for irrelevant articles in the fold""" 
469          # Find False Negatives and True Positives 
470          pos = nx.array(pos) 
471          pos.sort() 
472          P = len(pos) 
473          FN = 0 
474          while (FN < P) and (pos[FN] < self.threshold): 
475              FN += 1 
476          self.FN[fold] = FN 
477          self.TP[fold] = P - FN # TP+FN=P 
478          # Find True Negatives and False Positives 
479          neg = nx.array(neg) 
480          neg.sort() 
481          N = len(neg) 
482          TN = 0 
483          while (TN < N) and (neg[TN] < self.threshold): 
484              TN += 1 
485          self.TN[fold] = TN 
486          self.FP[fold] = N - TN # TN+FP=N

487   
488   


489 -    def stats_for(self, name):


490          """Return tuple of average, minimum and maximum values for the named 
491          statistic (must be an attribute name in L{PerformanceMetrics}""" 
492          return (getattr(self.average, name),  
493                  getattr(self.minimum, name), 
494                  getattr(self.maximum, name))

495   
496   


497 -    def fmt_stats(self, name, places=3):


498          """Return a string for the average, minimum and maximum values 
499          of the named statistic across folds""" 
500          numfmt = "%%.%df" % places 
501          if self.nfolds > 1: 
502              fmtstring = "%s (%s to %s)" % (numfmt,numfmt,numfmt) 
503              return fmtstring % self.stats_for(name) 
504          else: 
505              return numfmts % getattr(self.average, name)

506
```

  


| Trees | Indices | Help | | MScanner | | --- | |
| --- | --- | --- | --- | --- |

|  |  |
| --- | --- |
| Generated by Epydoc 3.0beta1 on Fri Nov 23 09:13:22 2007 | http://epydoc.sourceforge.net |
