# Supplementary material for: MScanner: a classifier for retrieving Medline citations
Source: BMC Bioinformatics. 2008 Feb 19;9:108. doi: 10.1186/1471-2105-9-108 (PMC2263023; doi:10.1186/1471-2105-9-108)
Supplement: Additional file 3 — Source code for MScanner. mscanner-20071123.zip is a ZIP archive containing the Python 2.5 source code for MScanner, licensed under the GNU General Public License. It also contains API documentation in HTML format. Updated versions will be made available at . [file 1471-2105-9-108-S3.zip › mscanner/help/api/mscanner.core.metrics.PerformanceMetrics-class.html]

xml version="1.0" encoding="ascii"?


mscanner.core.metrics.PerformanceMetrics


| Trees | Indices | Help | | MScanner | | --- | |
| --- | --- | --- | --- | --- |

|  |  |  |  |
| --- | --- | --- | --- |
| Package mscanner :: Package core :: Module metrics :: Class PerformanceMetrics | |  | | --- | | [hide private] | | [frames] | no frames] | |

# Class PerformanceMetrics

source code  
  
Performance metrics derived from a particular confusion matrix.  
  


---

**Note:**
PerformanceRange depends on all attributes being
numerical (so that comparison operators work).


|  |  |  |  |
| --- | --- | --- | --- |
| |  |  | | --- | --- | | Instance Methods | [hide private] | | |
|  | |  |  | | --- | --- | | \_\_init\_\_(self, TP, TN, FP, FN, alpha=0.5, utility\_r=None) | source code | |


|  |  |  |  |
| --- | --- | --- | --- |
| |  |  | | --- | --- | | Instance Variables | [hide private] | | |
|  | A  Total number of items (P+N). |
|  | F  Number of incorrect classifications. |
|  | FN  Confusion matrix. |
|  | FNR  Confusion matrix ratios. |
|  | FP  Confusion matrix. |
|  | FPR  Confusion matrix ratios. |
|  | N  Number of irrelevant items. |
|  | NPV  Positive and negative predictive value. |
|  | P  Number of relevant items. |
|  | PPV  Positive and negative predictive value. |
|  | T  Number of correct classifications. |
|  | TN  Confusion matrix. |
|  | TNR  Confusion matrix ratios. |
|  | TP  Confusion matrix. |
|  | TPR  Confusion matrix ratios. |
|  | accuracy  Equals T/A. |
|  | alpha  Weight of precision in F measure calculation. |
|  | enrichment  Equals precision/prevalence. |
|  | error  Equals F/A. |
|  | fmeasure  Harmonic mean of TPR and PPV [alpha=0.5] |
|  | fmeasure\_alpha  Alpha-weighted F measure [alpha!=0.5] |
|  | fp\_tp\_ratio  Equals FP/TP. |
|  | precision  Equals PPV. |
|  | prevalence  Equals P/A. |
|  | recall  Equals TPR. |
|  | specificity  Equals TNR. |
|  | utility\_r  Weight of a true positive (false positive is -1) (if None we use N/P). |

| Trees | Indices | Help | | MScanner | | --- | |
| --- | --- | --- | --- | --- |

|  |  |
| --- | --- |
| Generated by Epydoc 3.0beta1 on Fri Nov 23 09:13:21 2007 | http://epydoc.sourceforge.net |
