# Supplementary material for: MScanner: a classifier for retrieving Medline citations
Source: BMC Bioinformatics. 2008 Feb 19;9:108. doi: 10.1186/1471-2105-9-108 (PMC2263023; doi:10.1186/1471-2105-9-108)
Supplement: Additional file 3 — Source code for MScanner. mscanner-20071123.zip is a ZIP archive containing the Python 2.5 source code for MScanner, licensed under the GNU General Public License. It also contains API documentation in HTML format. Updated versions will be made available at . [file 1471-2105-9-108-S3.zip › mscanner/help/api/mscanner.core.metrics.PerformanceRange-class.html]

xml version="1.0" encoding="ascii"?


mscanner.core.metrics.PerformanceRange


| Trees | Indices | Help | | MScanner | | --- | |
| --- | --- | --- | --- | --- |

|  |  |  |  |
| --- | --- | --- | --- |
| Package mscanner :: Package core :: Module metrics :: Class PerformanceRange | |  | | --- | | [hide private] | | [frames] | no frames] | |

# Class PerformanceRange

source code  
  
Given a threshold, find the minimum and maximum for the precision,
recall across the validation folds.  
  


|  |  |  |  |
| --- | --- | --- | --- |
| |  |  | | --- | --- | | Instance Methods | [hide private] | | |
|  | |  |  | | --- | --- | | \_\_init\_\_(self, pscores, nscores, nfolds, threshold, average)  Parameters correspond to instance variables | source code | |
|  | |  |  | | --- | --- | | \_calculate\_min\_max(self)  Finds (min,max) of precision, etc., using the TP/TN/FP/FN vectors over the folds. | source code | |
|  | |  |  | | --- | --- | | \_make\_confusion\_vectors(self)  Finds TP, TN, FP, FN at the threshold over each validation fold | source code | |
|  | |  |  | | --- | --- | | \_confusion\_matrix(self, fold, pos, neg)  Find TP, TN, FP, FN at threshold inside a single validation fold. | source code | |
|  | |  |  | | --- | --- | | stats\_for(self, name)  Return tuple of average, minimum and maximum values for the named statistic (must be an attribute name in PerformanceMetrics | source code | |
|  | |  |  | | --- | --- | | fmt\_stats(self, name, places=3)  Return a string for the average, minimum and maximum values of the named statistic across folds | source code | |


|  |  |  |  |
| --- | --- | --- | --- |
| |  |  | | --- | --- | | Instance Variables | [hide private] | | |
|  | FN  Vector for number of FN in each fold |
|  | FP  Vector for number of FP in each fold |
|  | TN  Vector for number of TN in each fold |
|  | TP  Vector for number of TP in each fold |
|  | maximum  PerformanceMetrics with the maximum values across folds |
|  | minimum  PerformanceMetrics with the miminum values across folds |
| Passed via constructor | |
|  | average  PerformanceMetrics globally estimated using all folds |
|  | nfolds  Number of cross validation folds |
|  | nscores  Unsorted scores of negative documents. |
|  | pscores  Unsorted scores of positive documents. |
|  | threshold  Predict documents above this score to be positive. |


|  |  |  |  |
| --- | --- | --- | --- |
| |  |  | | --- | --- | | Method Details | [hide private] | | |

|  |  |  |
| --- | --- | --- |
| |  |  | | --- | --- | | \_confusion\_matrix(self, fold, pos, neg) | source code |  Find TP, TN, FP, FN at threshold inside a single validation fold. Parameters:  - **`fold`** - Number of the cross validation fold - **`pos`** - Scores for relevant articles in the fold - **`neg`** - Scores for irrelevant articles in the fold |

  


| Trees | Indices | Help | | MScanner | | --- | |
| --- | --- | --- | --- | --- |

|  |  |
| --- | --- |
| Generated by Epydoc 3.0beta1 on Fri Nov 23 09:13:21 2007 | http://epydoc.sourceforge.net |
