# Supplementary material for: MScanner: a classifier for retrieving Medline citations
Source: BMC Bioinformatics. 2008 Feb 19;9:108. doi: 10.1186/1471-2105-9-108 (PMC2263023; doi:10.1186/1471-2105-9-108)
Supplement: Additional file 3 — Source code for MScanner. mscanner-20071123.zip is a ZIP archive containing the Python 2.5 source code for MScanner, licensed under the GNU General Public License. It also contains API documentation in HTML format. Updated versions will be made available at . [file 1471-2105-9-108-S3.zip › mscanner/help/api/mscanner.core.metrics.PerformanceVectors-class.html]

xml version="1.0" encoding="ascii"?


mscanner.core.metrics.PerformanceVectors


| Trees | Indices | Help | | MScanner | | --- | |
| --- | --- | --- | --- | --- |

|  |  |  |  |
| --- | --- | --- | --- |
| Package mscanner :: Package core :: Module metrics :: Class PerformanceVectors | |  | | --- | | [hide private] | | [frames] | no frames] | |

# Class PerformanceVectors

source code  
  
Contains vectors of performance metrics at all possible threshold  
  


---

**Notes:**

- The performance statistics are calculated at every discreet
  threshold and stored in vectors.
- This copies pscores and nscores \*before\* sorting!
- If utility\_r is None, we use ratio of negatives to
  positives in the data.


|  |  |  |  |
| --- | --- | --- | --- |
| |  |  | | --- | --- | | Instance Methods | [hide private] | | |
|  | |  |  | | --- | --- | | \_\_init\_\_(self, pscores, nscores, alpha, utility\_r=None) | source code | |
|  | |  |  | | --- | --- | | \_confusion\_vectors(self)  Calculates confusion matrix counts by iterating over pscores | source code | |
|  | |  |  | | --- | --- | | \_ratio\_vectors(self, alpha)  Calculate performance using vector algebra | source code | |
|  | |  |  | | --- | --- | | \_curve\_areas(self)  Calculate areas under ROC and precision-recall curves | source code | |
|  | |  |  | | --- | --- | | \_mergescores(self)  Merges pscores and nscores in a single pass | source code | |
|  | |  |  | | --- | --- | | \_averaged\_precision(self)  Average the precision over each point of recall | source code | |
|  | |  |  | | --- | --- | | \_roc\_error(self)  Area under ROC and its standard error | source code | |
|  | |  |  | | --- | --- | | \_breakeven(self)  Calculate break-even point where precision equals recall. | source code | |
|  | |  |  | | --- | --- | | threshold\_maximising(self, vector)  Find threshold to maximise the given vector | source code | |
|  | |  |  | | --- | --- | | index\_for(self, threshold)  Calculate index into uscores corresponding to given threshold. | source code | |
|  | |  |  | | --- | --- | | matrix\_for(self, index)  Get confusion matrix at a threshold index into uscores. | source code | |
|  | |  |  | | --- | --- | | metrics\_for(self, index)  Get PerformanceMetrics at a threshold index into uscores. | source code | |


|  |  |  |  |
| --- | --- | --- | --- |
| |  |  | | --- | --- | | Instance Variables | [hide private] | | |
| Passed to constructor | |
|  | alpha  Balance of recall and precision in the F measure. |
|  | nscores  Vector of scores for negative articles, in increasing order. |
|  | pscores  Vector of scores for positive articles, in increasing order. |
|  | utility\_r  Value of a relevant article (irrelevant articles have value -1). |
| From \_confusion\_vectors | |
|  | FN  Vectors for confusion matrix at each distinct threshold. |
|  | FP  Vectors for confusion matrix at each distinct threshold. |
|  | NE  Number of negatives with each score in uscores. |
|  | PE  Number of positives with each score in uscores. |
|  | TN  Vectors for confusion matrix at each distinct threshold. |
|  | TP  Vectors for confusion matrix at each distinct threshold. |
|  | uscores  Unique scores in increasing order. |
| From \_ratio\_vectors | |
|  | FM  F measure at each threshold using alpha |
|  | FMa  F measure at each threshold using given alpha |
|  | FPR  False positive rate at each threshold |
|  | PPV  Positive predictive value at each threshold |
|  | TPR  True positive rate at each threshold |
|  | U  Utility at each threshold |
| From \_curve\_areas | |
|  | PR\_area  Aread under precision-recall curve. |
|  | ROC\_area  Area under ROC curve (trapezoidal under-estimate) |
| From \_roc\_error | |
|  | W  Area under ROC curve (better than trapezoidal area) |
|  | W\_stderr  Standard error of area under ROC curve. |
| From \_averaged\_precision | |
|  | AvPrec  Averaged precision (better than trapezoidal area) |
| From \_breakeven | |
|  | bep\_index  Index into uscores for break-even point. |
|  | breakeven  Value at the point where precision=recall. |


|  |  |  |  |
| --- | --- | --- | --- |
| |  |  | | --- | --- | | Method Details | [hide private] | | |

|  |  |  |
| --- | --- | --- |
| |  |  | | --- | --- | | \_confusion\_vectors(self) | source code |   Calculates confusion matrix counts by iterating over pscores Sets uscores, PE, NE, TP, TN, FP, FN |

|  |  |  |
| --- | --- | --- |
| |  |  | | --- | --- | | \_ratio\_vectors(self, alpha) | source code |  Calculate performance using vector algebra Parameters:  - **`alpha`** - Weight of precision in calculating FMa Sets TPR, FPR, PPV, FM, FMa, U |

|  |  |  |
| --- | --- | --- |
| |  |  | | --- | --- | | \_curve\_areas(self) | source code |   Calculate areas under ROC and precision-recall curves  Uses trapz(y, x). TPR is decreasing as threshold climbs, so vectors have to be reversed.  This method underestimates ROC areas because boundary points (0,0) and (1,1) usually are not present in the data. Better to use \_roc\_error which does not have that problem. Sets ROC\_area, PR\_area |

|  |  |  |
| --- | --- | --- |
| |  |  | | --- | --- | | \_mergescores(self) | source code |  Merges pscores and nscores in a single pass Returns:  Iterator over (score, relevance) in decreasing order of score. Relevance is True for members of pscores, and False for members of nscores.  **Note:** nscores and pscores must be in increasing order of score. |

|  |  |  |
| --- | --- | --- |
| |  |  | | --- | --- | | \_averaged\_precision(self) | source code |   Average the precision over each point of recall Sets AvPrec, which is precision averaged over each point where a relevant document is returned |

|  |  |  |
| --- | --- | --- |
| |  |  | | --- | --- | | \_roc\_error(self) | source code |   Area under ROC and its standard error Uses method of Hanley1982 to calculate standard error on the Wilcoxon statistic W, which corresponds to the area under the ROC by trapezoidal rule.   **Note:** The vectors r1 .. r7 correspond to rows of Table II in Hanley1982. Sets W and W\_stderr |

|  |  |  |
| --- | --- | --- |
| |  |  | | --- | --- | | \_breakeven(self) | source code |  Calculate break-even point where precision equals recall. Sets breakeven, bep\_index |

|  |  |  |
| --- | --- | --- |
| |  |  | | --- | --- | | threshold\_maximising(self, vector) | source code |  Find threshold to maximise the given vector Returns:  The threshold score, and its index in uscores |

|  |  |  |
| --- | --- | --- |
| |  |  | | --- | --- | | index\_for(self, threshold) | source code |  Calculate index into uscores corresponding to given threshold. Returns:  The highest available threshold less than the specified one, and its index in uscores. |

|  |  |  |
| --- | --- | --- |
| |  |  | | --- | --- | | matrix\_for(self, index) | source code |  Get confusion matrix at a threshold index into uscores. Returns:  TP, TN, FP, FN representing the confusion matrix. |

  


| Trees | Indices | Help | | MScanner | | --- | |
| --- | --- | --- | --- | --- |

|  |  |
| --- | --- |
| Generated by Epydoc 3.0beta1 on Fri Nov 23 09:13:21 2007 | http://epydoc.sourceforge.net |
