# Supplementary material for: MScanner: a classifier for retrieving Medline citations
Source: BMC Bioinformatics. 2008 Feb 19;9:108. doi: 10.1186/1471-2105-9-108 (PMC2263023; doi:10.1186/1471-2105-9-108)
Supplement: Additional file 3 — Source code for MScanner. mscanner-20071123.zip is a ZIP archive containing the Python 2.5 source code for MScanner, licensed under the GNU General Public License. It also contains API documentation in HTML format. Updated versions will be made available at . [file 1471-2105-9-108-S3.zip › mscanner/help/api/mscanner.core.metrics.PredictedMetrics-class.html]

xml version="1.0" encoding="ascii"?


mscanner.core.metrics.PredictedMetrics


| Trees | Indices | Help | | MScanner | | --- | |
| --- | --- | --- | --- | --- |

|  |  |  |  |
| --- | --- | --- | --- |
| Package mscanner :: Package core :: Module metrics :: Class PredictedMetrics | |  | | --- | | [hide private] | | [frames] | no frames] | |

# Class PredictedMetrics

source code  
  

Predict the performance metrics vectors for query results knowing only
the true and false positive rates in testing.

This also requires specifiying the size of the database against which
the query will be performed, and also the number of relevant documents
guessed to be present in the database.  
  


|  |  |  |  |
| --- | --- | --- | --- |
| |  |  | | --- | --- | | Instance Methods | [hide private] | | |
|  | |  |  | | --- | --- | | \_\_init\_\_(self, TPR, FPR, thresholds, relevant, total)  Constructor, calculates the predicted statistics | source code | |


|  |  |  |  |
| --- | --- | --- | --- |
| |  |  | | --- | --- | | Instance Variables | [hide private] | | |
|  | PPV  Predicted Precision (positive predictive value) at each threshold |
| Passed to constructor | |
|  | FPR  False Positive Rate in test corpus at each threshold (increasing) |
|  | TPR  True Positive Rate in test corpus at each threshold (increasing) |
|  | relevant  Number of relevant articles in database |
|  | thresholds  Threshold scores corresponding to TPR and FPR (decreasing) |
|  | total  Total number of articles in database |
| Calculated in constructor | |
|  | FP  Predicted number of false positives at each threshold |
|  | TP  Predicted number of true positives at each threshold |
|  | prevalence  Fraction of relevant articles in database |
|  | results  Predicted number of results at each threshold (TP+FP) |

| Trees | Indices | Help | | MScanner | | --- | |
| --- | --- | --- | --- | --- |

|  |  |
| --- | --- |
| Generated by Epydoc 3.0beta1 on Fri Nov 23 09:13:21 2007 | http://epydoc.sourceforge.net |
