# Supplementary material for: MScanner: a classifier for retrieving Medline citations
Source: BMC Bioinformatics. 2008 Feb 19;9:108. doi: 10.1186/1471-2105-9-108 (PMC2263023; doi:10.1186/1471-2105-9-108)
Supplement: Additional file 3 — Source code for MScanner. mscanner-20071123.zip is a ZIP archive containing the Python 2.5 source code for MScanner, licensed under the GNU General Public License. It also contains API documentation in HTML format. Updated versions will be made available at . [file 1471-2105-9-108-S3.zip › mscanner/help/api/mscanner.core.PerformanceRange-pysrc.html]

xml version="1.0" encoding="ascii"?


mscanner.core.PerformanceRange


| Trees | Indices | Help | | MScanner | | --- | |
| --- | --- | --- | --- | --- |

|  |  |  |  |
| --- | --- | --- | --- |
| Package mscanner :: Package core :: Module PerformanceRange | |  | | --- | | [hide private] | | [frames] | no frames] | |

# Source Code for Module mscanner.core.PerformanceRange

```
  1  """Given a threshold, find the minimum and maximum for the precision, recall, etc. 
  2  across the validation folds.""" 
  3   
  4  from __future__ import division 
  5  import numpy as nx 
  6  from mscanner.core.Validator import CrossValidator 
  7  from mscanner import update 
  8   
  9   
 10  __copyright__ = "2007 Graham Poulter" 
 11  __author__ = "Graham Poulter <http://graham.poulter.googlepages.com>" 
 12  __license__ = "GPL" 
 13   
 14   
 15   


16 -class PerformanceRange:


17      """Given a threshold, find the minimum and maximum for the precision, 
 18      recall across the validation folds. 
 19   
 20      @group pscores, nscores, nfolds, threshold: Passed via constructor 
 21       
 22      @ivar pscores: Unsorted scores of positive documents. 
 23      @ivar nscores: Unsorted scores of negative documents. 
 24      @ivar nfolds: Number of cross validation folds 
 25      @ivar threshold: Documents scoring above this are predicted positive. 
 26       
 27      @ivar TP: Vector with TP at threshold for each fold 
 28      @ivar FP: Vector with FP at threshold for each fold 
 29      @ivar TN: Vector with TN at threshold for each fold 
 30      @ivar FN: Vector with FN at threshold for each fold 
 31   
 32      @ivar precision: (min, max) of precision across all folds 
 33      @ivar recall: (min, max) of recall across all folds 
 34      @ivar fmeasure: (min, max) of F measure across all folds 
 35      """ 
 36       


37 -    def __init__(self, pscores, nscores, nfolds, threshold):


38          """Parameters correspond to instance variables""" 
 39          update(self, locals()) 
 40          self.do_confusion_vectors() 
 41          self.do_performance_range()

 42   
 43   


44 -    def do_confusion_vectors(self):


45          """Finds TP, TN, FP, FN at the threshold over each validation fold""" 
 46          for vname in ["TP", "TN", "FP", "FN"]: 
 47              setattr(self, vname, nx.zeros(self.nfolds, nx.float32)) 
 48          pstarts, psizes = CrossValidator.make_partitions( 
 49              len(self.pscores), self.nfolds) 
 50          nstarts, nsizes = CrossValidator.make_partitions( 
 51              len(self.nscores), self.nfolds) 
 52          for fold, (pstart,psize,nstart,nsize) in \ 
 53              enumerate(zip(pstarts,psizes,nstarts,nsizes)): 
 54              self.do_confusion_single( 
 55                  fold,  
 56                  self.pscores[pstart:pstart+psize], 
 57                  self.nscores[nstart:nstart+nsize])

 58   
 59   


60 -    def do_confusion_single(self, fold, pos, neg):


61          """Find TP, TN, FP, FN at threshold inside a single validation fold""" 
 62          # Find False Negatives and True Positives 
 63          pos = nx.array(pos) 
 64          pos.sort() 
 65          P = len(pos) 
 66          FN = 0 
 67          while (FN < P) and (pos[FN] < self.threshold): 
 68              FN += 1 
 69          self.FN[fold] = FN 
 70          self.TP[fold] = P - FN # TP+FN=P 
 71          # Find True Negatives and False Positives 
 72          neg = nx.array(neg) 
 73          neg.sort() 
 74          N = len(neg) 
 75          TN = 0 
 76          while (TN < N) and (neg[TN] < self.threshold): 
 77              TN += 1 
 78          self.TN[fold] = TN 
 79          self.FP[fold] = N - TN # TN+FP=N

 80   
 81   


82 -    def do_performance_range(self):


83          """Finds (min,max) of precision, etc., using the TP/TN/FP/FN vectors 
 84          over the folds.""" 
 85          for vname in ["precision", "recall", "fmeasure"]: 
 86              setattr(self, vname, (1.0, 0.0)) 
 87          for TP, FP, TN, FN in zip(self.TP, self.FP, self.TN, self.FN): 
 88              prec = (TP/(TP+FP)) if (TP+FP>0) else 0 
 89              rec = (TP/(TP+FN)) 
 90              F = 2 * prec * rec / (prec + rec) if (prec+rec>0) else 0 
 91              self._minimax("precision", prec) 
 92              self._minimax("recall", rec) 
 93              self._minimax("fmeasure", F)

 94       
 95   


96 -    def _minimax(self, varname, value):


97          """Given the name of a (min,max) attribute, update it 
 98          with a new value if that value extends the range. 
 99           
100          @param varname: Name of attribute to update 
101          @param value: If value is outside the range 
102          """ 
103          cmin, cmax = getattr(self, varname) 
104          if value < cmin: 
105              cmin = value 
106          if value > cmax: 
107              cmax = value 
108          setattr(self, varname, (cmin, cmax))

109
```

  


| Trees | Indices | Help | | MScanner | | --- | |
| --- | --- | --- | --- | --- |

|  |  |
| --- | --- |
| Generated by Epydoc 3.0beta1 on Fri Oct 26 21:01:07 2007 | http://epydoc.sourceforge.net |
