# Supplementary material for: MScanner: a classifier for retrieving Medline citations
Source: BMC Bioinformatics. 2008 Feb 19;9:108. doi: 10.1186/1471-2105-9-108 (PMC2263023; doi:10.1186/1471-2105-9-108)
Supplement: Additional file 3 — Source code for MScanner. mscanner-20071123.zip is a ZIP archive containing the Python 2.5 source code for MScanner, licensed under the GNU General Public License. It also contains API documentation in HTML format. Updated versions will be made available at . [file 1471-2105-9-108-S3.zip › mscanner/help/api/mscanner.core.PerformanceRange.PerformanceRange-class.html]

xml version="1.0" encoding="ascii"?


mscanner.core.PerformanceRange.PerformanceRange


| Trees | Indices | Help | | MScanner | | --- | |
| --- | --- | --- | --- | --- |

|  |  |  |  |
| --- | --- | --- | --- |
| Package mscanner :: Package core :: Module PerformanceRange :: Class PerformanceRange | |  | | --- | | [hide private] | | [frames] | no frames] | |

# Class PerformanceRange

source code  
  
Given a threshold, find the minimum and maximum for the precision,
recall across the validation folds.  
  


|  |  |  |  |
| --- | --- | --- | --- |
| |  |  | | --- | --- | | Instance Methods | [hide private] | | |
|  | |  |  | | --- | --- | | \_\_init\_\_(self, pscores, nscores, nfolds, threshold)  Parameters correspond to instance variables | source code | |
|  | |  |  | | --- | --- | | do\_confusion\_vectors(self)  Finds TP, TN, FP, FN at the threshold over each validation fold | source code | |
|  | |  |  | | --- | --- | | do\_confusion\_single(self, fold, pos, neg)  Find TP, TN, FP, FN at threshold inside a single validation fold | source code | |
|  | |  |  | | --- | --- | | do\_performance\_range(self)  Finds (min,max) of precision, etc., using the TP/TN/FP/FN vectors over the folds. | source code | |
|  | |  |  | | --- | --- | | \_minimax(self, varname, value)  Given the name of a (min,max) attribute, update it with a new value if that value extends the range. | source code | |


|  |  |  |  |
| --- | --- | --- | --- |
| |  |  | | --- | --- | | Instance Variables | [hide private] | | |
|  | FN  Vector with FN at threshold for each fold |
|  | FP  Vector with FP at threshold for each fold |
|  | TN  Vector with TN at threshold for each fold |
|  | TP  Vector with TP at threshold for each fold |
|  | fmeasure  (min, max) of F measure across all folds |
|  | nfolds  Number of cross validation folds |
|  | nscores  Unsorted scores of negative documents. |
|  | precision  (min, max) of precision across all folds |
|  | pscores  Unsorted scores of positive documents. |
|  | recall  (min, max) of recall across all folds |
|  | threshold  Documents scoring above this are predicted positive. |


|  |  |  |  |
| --- | --- | --- | --- |
| |  |  | | --- | --- | | Method Details | [hide private] | | |

|  |  |  |
| --- | --- | --- |
| |  |  | | --- | --- | | \_minimax(self, varname, value) | source code |  Given the name of a (min,max) attribute, update it with a new value if that value extends the range. Parameters:  - **`varname`** - Name of attribute to update - **`value`** - If value is outside the range |

  


| Trees | Indices | Help | | MScanner | | --- | |
| --- | --- | --- | --- | --- |

|  |  |
| --- | --- |
| Generated by Epydoc 3.0beta1 on Fri Oct 26 21:01:04 2007 | http://epydoc.sourceforge.net |
