# Supplementary material for: MScanner: a classifier for retrieving Medline citations
Source: BMC Bioinformatics. 2008 Feb 19;9:108. doi: 10.1186/1471-2105-9-108 (PMC2263023; doi:10.1186/1471-2105-9-108)
Supplement: Additional file 3 — Source code for MScanner. mscanner-20071123.zip is a ZIP archive containing the Python 2.5 source code for MScanner, licensed under the GNU General Public License. It also contains API documentation in HTML format. Updated versions will be made available at . [file 1471-2105-9-108-S3.zip › mscanner/help/api/mscanner.core.PerformanceStats-pysrc.html]

xml version="1.0" encoding="ascii"?


mscanner.core.PerformanceStats


| Trees | Indices | Help | | MScanner | | --- | |
| --- | --- | --- | --- | --- |

|  |  |  |  |
| --- | --- | --- | --- |
| Package mscanner :: Package core :: Module PerformanceStats | |  | | --- | | [hide private] | | [frames] | no frames] | |

# Source Code for Module mscanner.core.PerformanceStats

```
  1  """Calculates performance statistics given the scores of the positive and 
  2  negative citations""" 
  3   
  4  from __future__ import division 
  5  import numpy as nx 
  6   
  7   
  8  __copyright__ = "2007 Graham Poulter" 
  9  __author__ = "Graham Poulter <http://graham.poulter.googlepages.com>" 
 10  __license__ = """This program is free software: you can redistribute it and/or 
 11  modify it under the terms of the GNU General Public License as published by the 
 12  Free Software Foundation, either version 3 of the License, or (at your option) 
 13  any later version. 
 14   
 15  This program is distributed in the hope that it will be useful, but WITHOUT ANY 
 16  WARRANTY; without even the implied warranty of MERCHANTABILITY or FITNESS FOR A 
 17  PARTICULAR PURPOSE. See the GNU General Public License for more details. 
 18   
 19  You should have received a copy of the GNU General Public License along with 
 20  this program. If not, see <http://www.gnu.org/licenses/>.""" 
 21   
 22   


23 -class PerformanceStats:


24      """Performance statistics calculation after cross validation. 
 25   
 26      @group From constructor: pscores, nscores, alpha, P, N, A 
 27       
 28      @ivar pscores: Increasing scores of positive articles. 
 29   
 30      @ivar nscores: Increasing scores of negative articles. 
 31   
 32      @ivar alpha: Balance of recall and precision in the F measure. 
 33       
 34      @ivar utility_r: Utility of retrieving one relevant article (retrieving 
 35      an irrelevant article has utility -1).  If None, use ratio of 
 36      negatives to positives in the data. 
 37   
 38      @ivar P: Number of positive articles. 
 39   
 40      @ivar N: Number of negative articles. 
 41   
 42      @ivar A: Equal to L{P}+L{N}. 
 43       
 44       
 45      @group From counts: uscores, vlen, PE, NE, TP, FN, FP, TP 
 46       
 47      @ivar uscores: Unique scores in increasing order 
 48   
 49      @ivar vlen: Length of performance vectors (= length of L{uscores}) 
 50   
 51      @ivar PE: Number of positives with each score in L{uscores} 
 52   
 53      @ivar NE: Number of negatives with each score in L{uscores} 
 54       
 55      @ivar TP, FN, FP, TN: Vectors for confusion matrix at each distinct threshold 
 56       
 57       
 58      @group From make_ratio_vectors: TPR, FPR, PPV, FM, FMa 
 59       
 60      @ivar TPR, FPR, PPV, FM, FMa: Vectors of performance ratios at each 
 61      distinct threshold. 
 62       
 63       
 64      @group From make_curve_areas: ROC_area,PR_area 
 65       
 66      @ivar ROC_area: Area under ROC curve. 
 67       
 68      @ivar PR_area: Aread under precision-recall curve. 
 69       
 70       
 71      @ivar bep_index, breakeven: Breakeven point (where precision=recall), from 
 72      L{find_breakeven}. 
 73       
 74      @ivar threshold_index, threshold: Tuned threshold and its index, from 
 75      L{maximise_fmeasure}. 
 76       
 77      @ivar tuned: Tuned performance statistics, from L{get_tunedstats}. 
 78       
 79      @ivar W, W_stderr: Better area under ROC curve, from L{roc_error}. 
 80       
 81      @ivar AvPrec: Averaged precision, from L{averaged_precision}. 
 82      """ 
 83   
 84   


85 -    def __init__(self, pscores, nscores, alpha, utility_r=None):


86          """Constructor - parameters correspond to instance variables. 
 87   
 88          @note: Sorted copies are made of L{pscores} and L{nscores}. 
 89          """ 
 90          s = self 
 91          s.alpha = alpha 
 92          s.pscores = pscores.copy() 
 93          s.nscores = nscores.copy() 
 94          s.pscores.sort() 
 95          s.nscores.sort() 
 96          s.P = len(s.pscores) 
 97          s.N = len(s.nscores) 
 98          s.A = s.P + s.N 
 99          s.utility_r = s.N/s.P if utility_r is None else utility_r 
100          s.make_confusion_matrix() 
101          s.make_ratio_vectors(s.alpha) 
102          s.make_curve_areas() 
103          s.roc_error() 
104          s.averaged_precision() 
105          #s.maximise_fmeasure() 
106          s.maximise_utility() 
107          s.find_breakeven() 
108          s.get_tunedstats()

109   
110   


111 -    def make_confusion_matrix(self):


112          """Calculates confusion matrix counts by iterating over pscores 
113           
114          As a side effects, sets L{uscores}, L{vlen}, L{PE}, L{NE} 
115           
116          @return: L{TP}, L{TN}, L{FP}, L{FN} 
117          """ 
118          s = self 
119          s.uscores = nx.unique(nx.concatenate((s.pscores,s.nscores))) 
120          s.vlen = len(s.uscores) 
121          s.PE = nx.zeros(s.vlen, nx.float32) # positives with given score 
122          s.NE = nx.zeros(s.vlen, nx.float32) # negatives with given score 
123          s.TP = nx.zeros(s.vlen, nx.float32) # true positives 
124          s.TN = nx.zeros(s.vlen, nx.float32) # true negatives 
125          s.FP = nx.zeros(s.vlen, nx.float32) # false positives 
126          s.FN = nx.zeros(s.vlen, nx.float32) # false negatives 
127          TN = 0 
128          FN = 0 
129          for idx, threshold in enumerate(s.uscores): 
130   
131              # Classify positives scoring < threshold as negative 
132              # (look up score for the next article to classify) 
133              while (FN < s.P) and (s.pscores[FN] < threshold): 
134                  FN += 1 
135              TP = s.P - FN # TP+FN=P 
136   
137              # pcount-FN = number of positives having threshold score 
138              pcount = FN # Start at FN, subtract it later 
139              while (pcount < s.P) and s.pscores[pcount] == threshold: 
140                  pcount += 1 
141              s.PE[idx] = pcount-FN 
142               
143              # Classify negatives scoring < threshold as negative 
144              while (TN < s.N) and (s.nscores[TN] < threshold): 
145                  TN += 1 
146              FP = s.N - TN  # TN+FP=N 
147   
148              # ncount-TN = number of negatives having threshold score 
149              ncount = TN # Start at TN and subtract it later 
150              while (ncount < s.N) and s.nscores[ncount] == threshold: 
151                  ncount += 1 
152              s.NE[idx] = ncount-TN 
153               
154              s.TP[idx] = TP 
155              s.TN[idx] = TN 
156              s.FP[idx] = FP 
157              s.FN[idx] = FN 
158          return s.TP, s.TN, s.FP, s.FN

159   
160   


161 -    def make_ratio_vectors(self, alpha):


162          """Calculate performance using vector algebra 
163   
164          @param alpha: Weight of precision in calculating FMa 
165           
166          @return: L{TPR}, L{FPR}, L{PPV}, L{FM}, L{FMa} 
167          """ 
168          s = self 
169          # TPR is recall 
170          s.TPR = s.TP / s.P 
171          # FPR is 1-specificity 
172          s.FPR = s.FP / s.N 
173          # PPV is precision 
174          s.PPV = s.TP / (s.TP + s.FP)  
175          s.PPV[s.TP+s.FP == 0] = 1.0 
176          # FM is F-Measure 
177          s.FM = 2 * s.TPR * s.PPV / (s.TPR + s.PPV)  
178          # FMa is the alpha-weighted F-Measures 
179          s.FMa = 1 / (alpha / s.PPV + (1 - alpha) / s.TPR) 
180          # U is the utility function 
181          s.U = (s.utility_r * s.TP - s.FP) / (self.utility_r * s.P) 
182          return s.TPR, s.FPR, s.PPV, s.FM, s.FMa

183   
184   


185 -    def make_curve_areas(self):


186          """Calculate areas under ROC and precision-recall curves 
187           
188          Uses trapz(y, x). TPR is decreasing as threshold climbs, so vectors 
189          have to be reversed. 
190           
191          This method underestimates ROC areas because boundary points (0,0) and 
192          (1,1) usually are not present in the data. Better to use L{roc_error} 
193          which does not have that problem. 
194           
195          @return: L{ROC_area}, L{PR_area}""" 
196          from scipy.integrate import trapz 
197          s = self 
198          s.ROC_area = trapz(s.TPR[::-1], s.FPR[::-1]) 
199          s.PR_area = trapz(s.PPV[::-1], s.TPR[::-1]) 
200          return s.ROC_area, s.PR_area

201   
202   


203 -    def mergescores(self):


204          """Merged the contents of pscores and nscores in a single pass. 
205           
206          Expects L{nscores} and L{pscores} in increasing order of score.         
207           
208          @return: Iterator over (score, relevance) in decreasing order of score. 
209          Relevance is True for members of pscores, and False for members of 
210          nscores. """ 
211          s = self 
212          p_idx = s.P-1 
213          n_idx = s.N-1 
214          while p_idx >= 0 or n_idx >= 0: 
215              if p_idx >= 0 and \ 
216              (n_idx < 0 or s.pscores[p_idx] >= s.nscores[n_idx]): 
217                  yield s.pscores[p_idx], True 
218                  p_idx -= 1 
219              elif n_idx >= 0 and \ 
220              (p_idx < 0 or s.nscores[n_idx] > s.pscores[p_idx]): 
221                  yield s.nscores[n_idx], False 
222                  n_idx -= 1

223   
224   


225 -    def averaged_precision(self):


226          """Average the precision over each point of recall 
227           
228          @return: L{AvPrec}, the precision averaged over each point where a 
229          relevant document is returned""" 
230          AvPrec = 0.0 
231          TP = 0 
232          FP = 0 
233          for score, relevant in self.mergescores(): 
234              if relevant: 
235                  TP += 1 
236                  AvPrec += TP/(TP+FP) 
237              else: 
238                  FP += 1 
239          self.AvPrec = AvPrec/TP 
240          return self.AvPrec

241   
242   


243 -    def roc_error(self):


244          """Area under ROC and its standard error 
245           
246          Uses method of Hanley1982 to calculate standard error on the Wilcoxon 
247          statistic W, which corresponds to the area under the ROC by trapezoidal 
248          rule. 
249           
250          @note: The vectors r1 .. r7 correspond to rows of Table II in 
251          Hanley1982. 
252   
253          @return: L{W}, L{W_stderr} 
254          """ 
255          s = self 
256          # r1 is number of negatives with each score, 
257          # r2 is number of positives rated higher than each score 
258          # r3 is number of positives with each score 
259          # r4 is number of negatives rated lower than each score 
260          r1 = s.NE 
261          r2 = s.TP - s.PE 
262          r3 = s.PE 
263          r4 = s.TN 
264          r5 = r1 * r2 + 0.5 * r1 * r3 
265          r6 = r3 * (r4**2 + r4*r1 + (r1**2)/3) 
266          r7 = r1 * (r2**2 + r2*r3 + (r3**2)/3) 
267          N = float(s.N) 
268          P = float(s.P) 
269          W = r5.sum() / (N*P) 
270          Q2 = r6.sum() / (P * N**2) 
271          Q1 = r7.sum() / (N * P**2) 
272          W_stderr = nx.sqrt((W*(1-W)+(P-1)*(Q1-W**2)+(N-1)*(Q2-W**2))/(P*N)) 
273          #print W, Q1, Q2, W_stderr 
274          s.W = W 
275          s.W_stderr = W_stderr 
276          return W, W_stderr

277   
278   


279 -    def find_breakeven(self):


280          """Calculate break-even, where precision equals recall. 
281           
282          @return: L{bep_index}, L{breakeven} - index into pscores, 
283          and the recall/precision of the break-even point. 
284          """ 
285          s = self 
286          diff = nx.absolute(nx.subtract(s.TPR, s.PPV)) 
287          s.bep_index = nx.nonzero(diff == nx.min(diff))[0][0] 
288          s.breakeven = 0.5*(s.TPR[s.bep_index]+s.PPV[s.bep_index]) 
289          return s.bep_index, s.breakeven

290   
291   


292 -    def maximise_fmeasure(self):


293          """Point of maximum F measure 
294          @return: L{threshold} and L{threshold_index}""" 
295          s = self 
296          max_FMa = nx.max(s.FMa) 
297          s.threshold_index = nx.nonzero(s.FMa == max_FMa)[0][0] 
298          s.threshold = self.uscores[s.threshold_index] 
299          return s.threshold, s.threshold_index

300       
301       


302 -    def maximise_utility(self):


303          """Point of maximum utility 
304          @return: L{threshold} and L{threshold_index}""" 
305          s = self 
306          max_U = nx.max(s.U) 
307          s.threshold_index = nx.nonzero(s.U == max_U)[0][0] 
308          s.threshold = self.uscores[s.threshold_index] 
309          return s.threshold, s.threshold_index

310   
311   


312 -    def get_tunedstats(self):


313          """Performance at the chosen threshold (usually the point of maximum F 
314          measure). 
315           
316          @return: Storage object with these keys:: 
317            P, N, A, T, F      (summary of input) 
318            TP, FP, TN, FN     (confusion matrix) 
319            TPR, FNR, TNR, FPR (ratios) 
320            PPV, NPV           (ratios) 
321            accuracy           (T/A) 
322            enrichment         (precision/prevalence) 
323            error              (F/A) 
324            fmeasure           (harmonic mean of TPR and PPV [alpha=0.5]) 
325            fmeasure_alpha     (alpha-weighted F measure [alpha!=0.5]) 
326            fmeasure_max       (maximum of standard F measure [alpha=0.5]) 
327            precision          (PPV) 
328            prevalence         (P/A) 
329            recall             (TPR) 
330            specificity        (TNR) 
331            fp_tp_ratio        (FP/TP) 
332          """ 
333          TP = int(self.TP[self.threshold_index]) 
334          TN = int(self.TN[self.threshold_index]) 
335          FP = int(self.FP[self.threshold_index]) 
336          FN = int(self.FN[self.threshold_index]) 
337          P = self.P  # P = TP + FN 
338          N = self.N  # N = TN + FP 
339          A = self.A 
340          T = TP + TN 
341          F = FP + FN 
342          U = (self.utility_r * TP - FP) / (self.utility_r * P) 
343          U_max = nx.max(self.U) 
344          TPR, FNR, TNR, FPR, PPV, NPV, FDR = 0, 0, 0, 0, 0, 0, 0 
345          if TP + FN != 0: 
346              TPR = TP / (TP + FN) # TPR=TP/P = sensitivity = recall 
347              FNR = FN / (TP + FN) # FNR=FN/P = 1-TP/P = 1-sensitivity = 1-recall 
348          if TN + FP != 0: 
349              TNR = TN / (TN + FP) # TNR=TN/N = specificity 
350              FPR = FP / (TN + FP) # FPR=FP/N = 1 - TN/N = 1-specificity 
351          if TP + FP != 0: 
352              PPV = TP / (TP + FP) # PPV=precision 
353              FDR = FP / (TP + FP) # FDR=1-precision 
354          if TN + FN != 0: 
355              NPV = TN / (TN + FN) # NPV 
356          accuracy = T / A if A != 0 else 0 
357          prevalence = P / A if A != 0 else 0 
358          error = 1 - accuracy 
359          recall = TPR 
360          specificity = TNR 
361          precision = PPV 
362          fp_tp_ratio = FP/TP if TP != 0 else 0 
363          fmeasure, fmeasure_alpha, fmeasure_max = 0, 0, 0 
364          if recall > 0 and precision > 0: 
365              fmeasure = 2 * recall * precision / (recall + precision) 
366              fmeasure_alpha = 1.0 / ( (self.alpha / precision) +  
367                                       ((1 - self.alpha) / recall)) 
368              fmeasure_max = nx.max(self.FM) 
369          enrichment = 0 
370          if prevalence > 0: 
371              enrichment = precision / prevalence 
372          # Return local variables in a Storage object 
373          from mscanner.core.Storage import Storage 
374          self.tuned = Storage(locals()) 
375          del self.tuned.self 
376          return self.tuned

377
```

  


| Trees | Indices | Help | | MScanner | | --- | |
| --- | --- | --- | --- | --- |

|  |  |
| --- | --- |
| Generated by Epydoc 3.0beta1 on Fri Oct 26 21:01:07 2007 | http://epydoc.sourceforge.net |
