# Supplementary material for: MScanner: a classifier for retrieving Medline citations
Source: BMC Bioinformatics. 2008 Feb 19;9:108. doi: 10.1186/1471-2105-9-108 (PMC2263023; doi:10.1186/1471-2105-9-108)
Supplement: Additional file 3 — Source code for MScanner. mscanner-20071123.zip is a ZIP archive containing the Python 2.5 source code for MScanner, licensed under the GNU General Public License. It also contains API documentation in HTML format. Updated versions will be made available at . [file 1471-2105-9-108-S3.zip › mscanner/help/api/mscanner.core.PerformanceStats.PerformanceStats-class.html]

xml version="1.0" encoding="ascii"?


mscanner.core.PerformanceStats.PerformanceStats


| Trees | Indices | Help | | MScanner | | --- | |
| --- | --- | --- | --- | --- |

|  |  |  |  |
| --- | --- | --- | --- |
| Package mscanner :: Package core :: Module PerformanceStats :: Class PerformanceStats | |  | | --- | | [hide private] | | [frames] | no frames] | |

# Class PerformanceStats

source code  
  
Performance statistics calculation after cross validation.  
  


|  |  |  |  |
| --- | --- | --- | --- |
| |  |  | | --- | --- | | Instance Methods | [hide private] | | |
|  | |  |  | | --- | --- | | \_\_init\_\_(self, pscores, nscores, alpha, utility\_r=None)  Constructor - parameters correspond to instance variables. | source code | |
|  | |  |  | | --- | --- | | make\_confusion\_matrix(self)  Calculates confusion matrix counts by iterating over pscores | source code | |
|  | |  |  | | --- | --- | | make\_ratio\_vectors(self, alpha)  Calculate performance using vector algebra | source code | |
|  | |  |  | | --- | --- | | make\_curve\_areas(self)  Calculate areas under ROC and precision-recall curves | source code | |
|  | |  |  | | --- | --- | | mergescores(self)  Merged the contents of pscores and nscores in a single pass. | source code | |
|  | |  |  | | --- | --- | | averaged\_precision(self)  Average the precision over each point of recall | source code | |
|  | |  |  | | --- | --- | | roc\_error(self)  Area under ROC and its standard error | source code | |
|  | |  |  | | --- | --- | | find\_breakeven(self)  Calculate break-even, where precision equals recall. | source code | |
|  | |  |  | | --- | --- | | maximise\_fmeasure(self)  Point of maximum F measure | source code | |
|  | |  |  | | --- | --- | | maximise\_utility(self)  Point of maximum utility | source code | |
|  | |  |  | | --- | --- | | get\_tunedstats(self)  Performance at the chosen threshold (usually the point of maximum F measure). | source code | |


|  |  |  |  |
| --- | --- | --- | --- |
| |  |  | | --- | --- | | Instance Variables | [hide private] | | |
|  | AvPrec  Averaged precision, from averaged\_precision. |
|  | TN  Vectors for confusion matrix at each distinct threshold |
|  | W  Better area under ROC curve, from roc\_error. |
|  | W\_stderr  Better area under ROC curve, from roc\_error. |
|  | bep\_index  Breakeven point (where precision=recall), from find\_breakeven. |
|  | breakeven  Breakeven point (where precision=recall), from find\_breakeven. |
|  | threshold  Tuned threshold and its index, from maximise\_fmeasure. |
|  | threshold\_index  Tuned threshold and its index, from maximise\_fmeasure. |
|  | tuned  Tuned performance statistics, from get\_tunedstats. |
|  | utility\_r  Utility of retrieving one relevant article (retrieving an irrelevant article has utility -1). |
| From constructor | |
|  | A  Equal to P+N. |
|  | N  Number of negative articles. |
|  | P  Number of positive articles. |
|  | alpha  Balance of recall and precision in the F measure. |
|  | nscores  Increasing scores of negative articles. |
|  | pscores  Increasing scores of positive articles. |
| From counts | |
|  | FN  Vectors for confusion matrix at each distinct threshold |
|  | FP  Vectors for confusion matrix at each distinct threshold |
|  | NE  Number of negatives with each score in uscores |
|  | PE  Number of positives with each score in uscores |
|  | TP  Vectors for confusion matrix at each distinct threshold |
|  | uscores  Unique scores in increasing order |
|  | vlen  Length of performance vectors (= length of uscores) |
| From make\_ratio\_vectors | |
|  | FM  Vectors of performance ratios at each distinct threshold. |
|  | FMa  Vectors of performance ratios at each distinct threshold. |
|  | FPR  Vectors of performance ratios at each distinct threshold. |
|  | PPV  Vectors of performance ratios at each distinct threshold. |
|  | TPR  Vectors of performance ratios at each distinct threshold. |
| From make\_curve\_areas | |
|  | PR\_area  Aread under precision-recall curve. |
|  | ROC\_area  Area under ROC curve. |


|  |  |  |  |
| --- | --- | --- | --- |
| |  |  | | --- | --- | | Method Details | [hide private] | | |

|  |  |  |
| --- | --- | --- |
| |  |  | | --- | --- | | \_\_init\_\_(self, pscores, nscores, alpha, utility\_r=None)  *(Constructor)* | source code |  Constructor - parameters correspond to instance variables.   **Note:** Sorted copies are made of pscores and nscores. |

|  |  |  |
| --- | --- | --- |
| |  |  | | --- | --- | | make\_confusion\_matrix(self) | source code |   Calculates confusion matrix counts by iterating over pscores As a side effects, sets uscores, vlen, PE, NE Returns:  TP, TN, FP, FN |

|  |  |  |
| --- | --- | --- |
| |  |  | | --- | --- | | make\_ratio\_vectors(self, alpha) | source code |  Calculate performance using vector algebra Parameters:  - **`alpha`** - Weight of precision in calculating FMa  Returns:  TPR, FPR, PPV, FM, FMa |

|  |  |  |
| --- | --- | --- |
| |  |  | | --- | --- | | make\_curve\_areas(self) | source code |   Calculate areas under ROC and precision-recall curves  Uses trapz(y, x). TPR is decreasing as threshold climbs, so vectors have to be reversed. This method underestimates ROC areas because boundary points (0,0) and (1,1) usually are not present in the data. Better to use roc\_error which does not have that problem. Returns:  ROC\_area, PR\_area |

|  |  |  |
| --- | --- | --- |
| |  |  | | --- | --- | | mergescores(self) | source code |   Merged the contents of pscores and nscores in a single pass. Expects nscores and pscores in increasing order of score. Returns:  Iterator over (score, relevance) in decreasing order of score. Relevance is True for members of pscores, and False for members of nscores. |

|  |  |  |
| --- | --- | --- |
| |  |  | | --- | --- | | averaged\_precision(self) | source code |  Average the precision over each point of recall Returns:  AvPrec, the precision averaged over each point where a relevant document is returned |

|  |  |  |
| --- | --- | --- |
| |  |  | | --- | --- | | roc\_error(self) | source code |   Area under ROC and its standard error Uses method of Hanley1982 to calculate standard error on the Wilcoxon statistic W, which corresponds to the area under the ROC by trapezoidal rule. Returns:  W, W\_stderr  **Note:** The vectors r1 .. r7 correspond to rows of Table II in Hanley1982. |

|  |  |  |
| --- | --- | --- |
| |  |  | | --- | --- | | find\_breakeven(self) | source code |  Calculate break-even, where precision equals recall. Returns:  bep\_index, breakeven - index into pscores, and the recall/precision of the break-even point. |

|  |  |  |
| --- | --- | --- |
| |  |  | | --- | --- | | maximise\_fmeasure(self) | source code |  Point of maximum F measure Returns:  threshold and threshold\_index |

|  |  |  |
| --- | --- | --- |
| |  |  | | --- | --- | | maximise\_utility(self) | source code |  Point of maximum utility Returns:  threshold and threshold\_index |

|  |  |  |
| --- | --- | --- |
| |  |  | | --- | --- | | get\_tunedstats(self) | source code |  Performance at the chosen threshold (usually the point of maximum F measure). Returns:  Storage object with these keys:  ```  P, N, A, T, F      (summary of input)  TP, FP, TN, FN     (confusion matrix)  TPR, FNR, TNR, FPR (ratios)  PPV, NPV           (ratios)  accuracy           (T/A)  enrichment         (precision/prevalence)  error              (F/A)  fmeasure           (harmonic mean of TPR and PPV [alpha=0.5])  fmeasure_alpha     (alpha-weighted F measure [alpha!=0.5])  fmeasure_max       (maximum of standard F measure [alpha=0.5])  precision          (PPV)  prevalence         (P/A)  recall             (TPR)  specificity        (TNR)  fp_tp_ratio        (FP/TP) ``` |

  


|  |  |  |  |
| --- | --- | --- | --- |
| |  |  | | --- | --- | | Instance Variable Details | [hide private] | | |

|  |
| --- |
| utility\_rUtility of retrieving one relevant article (retrieving an irrelevant article has utility -1). If None, use ratio of negatives to positives in the data. |

  


| Trees | Indices | Help | | MScanner | | --- | |
| --- | --- | --- | --- | --- |

|  |  |
| --- | --- |
| Generated by Epydoc 3.0beta1 on Fri Oct 26 21:01:05 2007 | http://epydoc.sourceforge.net |
