# Supplementary material for: MScanner: a classifier for retrieving Medline citations
Source: BMC Bioinformatics. 2008 Feb 19;9:108. doi: 10.1186/1471-2105-9-108 (PMC2263023; doi:10.1186/1471-2105-9-108)
Supplement: Additional file 3 — Source code for MScanner. mscanner-20071123.zip is a ZIP archive containing the Python 2.5 source code for MScanner, licensed under the GNU General Public License. It also contains API documentation in HTML format. Updated versions will be made available at . [file 1471-2105-9-108-S3.zip › mscanner/help/api/mscanner.core.Plotter-pysrc.html]

xml version="1.0" encoding="ascii"?


mscanner.core.Plotter


| Trees | Indices | Help | | MScanner | | --- | |
| --- | --- | --- | --- | --- |

|  |  |  |  |
| --- | --- | --- | --- |
| Package mscanner :: Package core :: Module Plotter | |  | | --- | | [hide private] | | [frames] | no frames] | |

# Source Code for Module mscanner.core.Plotter

```
  1  """Plotting functions for all graphs produced in cross validation.""" 
  2   
  3  from __future__ import division 
  4  from Gnuplot import Data, Gnuplot 
  5  import logging 
  6  import numpy as nx 
  7   
  8   
  9  __copyright__ = "2007 Graham Poulter" 
 10  __author__ = "Graham Poulter <http://graham.poulter.googlepages.com>" 
 11  __license__ = """This program is free software: you can redistribute it and/or 
 12  modify it under the terms of the GNU General Public License as published by the 
 13  Free Software Foundation, either version 3 of the License, or (at your option) 
 14  any later version. 
 15   
 16  This program is distributed in the hope that it will be useful, but WITHOUT ANY 
 17  WARRANTY; without even the implied warranty of MERCHANTABILITY or FITNESS FOR A 
 18  PARTICULAR PURPOSE. See the GNU General Public License for more details. 
 19   
 20  You should have received a copy of the GNU General Public License along with 
 21  this program. If not, see <http://www.gnu.org/licenses/>.""" 
 22   
 23   


24 -class Plotter:


25      """Implements the plots used in MScanner 
 26       
 27      When adding a new analysis, the plotting function for its output 
 28      graphs should be added here. 
 29       
 30      @note: All methods take an fname parameter, which is the path to the PNG 
 31      file to which the graph will be written. 
 32       
 33      @ivar overwrite: If False, we no-op rather than overwrite an already 
 34      existing graph. 
 35       
 36      @ivar gnuplot: The captive Gnuplot instance. 
 37      """ 
 38   


39 -    def __init__(self, overwrite=True):


40          self.overwrite = overwrite 
 41          self.gnuplot = Gnuplot()

 42   
 43   


44 -    def plot_predictions(self, fname, predicted_low, predicted_high):


45          """Given L{PredictedMetrics} instance, plot the predicted query 
 46          performance (TPR and PPV vs number of results). 
 47           
 48          @note: Two predictions are given, corresponding to upper and lower 
 49          bound guesses at the number of  
 50          """ 
 51          if fname.exists() and not self.overwrite: return 
 52          g = self.gnuplot 
 53          logging.debug("Plotting prediction curve to %s", fname.basename()) 
 54          g.reset() 
 55          g.title("Prediction curve (TPR, PPV vs # results)") 
 56          g.ylabel("TPR, PPV") 
 57          g.xlabel("Number of results") 
 58          g("set terminal png") 
 59          g("set output '%s'" % fname) 
 60          # Only plot until 95% recall in both cases 
 61          pL = predicted_low 
 62          pH = predicted_high 
 63          seg = (pL.TPR < 0.95) | (pH.TPR < 0.95)  
 64          g.plot( 
 65              Data(pL.results[seg], pL.TPR[seg], title="TPR low", with="lines"), 
 66              Data(pL.results[seg], pL.PPV[seg], title="PPV low", with="lines"), 
 67              Data(pH.results[seg], pH.TPR[seg], title="TPR high", with="lines"), 
 68              Data(pH.results[seg], pH.PPV[seg], title="PPV high", with="lines"))

 69   
 70       


71 -    def plot_roc(self, fname, FPR, TPR, marker_FPR):


72          """ROC curve (TPR vs FPR)""" 
 73          if fname.exists() and not self.overwrite: return 
 74          g = self.gnuplot 
 75          logging.debug("Plotting ROC curve to %s", fname.basename()) 
 76          g.reset() 
 77          g.title("ROC curve (TPR vs FPR)") 
 78          g.ylabel("True Positive Rate (TPR)") 
 79          g.xlabel("False Positive Rate (FPR)") 
 80          g("set terminal png") 
 81          g("set output '%s'" % fname) 
 82          g.plot(Data(FPR, TPR, title="TPR", with="lines"), 
 83                 Data([marker_FPR, marker_FPR], [0,0.99], title="threshold", with="lines"))

 84   
 85   


86 -    def plot_precision(self, fname, TPR, PPV, marker_TPR):


87          """Precision vs recall""" 
 88          if fname.exists() and not self.overwrite: return 
 89          g = self.gnuplot 
 90          logging.debug("Plotting Precision-Recall curve to %s", fname.basename()) 
 91          g.reset() 
 92          g.title("Precision vs Recall") 
 93          g.ylabel("Precision") 
 94          g.xlabel("Recall") 
 95          g("set terminal png") 
 96          g("set output '%s'" % fname) 
 97          g.plot(Data(TPR, PPV, title="Precision", with="lines", smooth="csplines"), 
 98                 Data([marker_TPR, marker_TPR], [0,0.99], title="threshold", with="lines"))

 99   
100   


101 -    def plot_fmeasure(self, fname, pscores, TPR, PPV, FM, FMa, threshold):


102          """Precision, Recall, F-Measure vs threshold""" 
103          if fname.exists() and not self.overwrite: return 
104          g = self.gnuplot 
105          logging.debug("Plotting F-Measure curve to %s", fname.basename()) 
106          g.reset() 
107          g.title("Precision and Recall vs Threshold") 
108          g.ylabel("Precision, Recall, F-Measure, F-Measure Alpha") 
109          g.xlabel("Threshold Score") 
110          g("set terminal png") 
111          g("set output '%s'" % fname) 
112          g.plot(Data(pscores, TPR, title="Recall",     with="lines"), 
113                 Data(pscores, PPV, title="Precision",  with="lines"), 
114                 Data(pscores, FM,  title="F1 Measure", with="lines"), 
115                 Data(pscores, FMa, title="F Measure",  with="lines"), 
116                 Data([threshold, threshold], [0,0.99], title="threshold", with="lines"))

117   
118   
119      @staticmethod 


120 -    def bincount(data):


121          """Calculate the best number of histogram bins for the data 
122           
123          Uses the formula M{K = R/(2*IQR*N^(-1/3))} 
124           
125          @param data: Array of numbers, sorted in increasing order. 
126          """ 
127          N = len(data) # Number of data points 
128          IQR = data[3*N//4] - data[N//4] # Inter-Quartile Range 
129          R = data[-1] - data[0] # Range 
130          bins = R//(2*IQR*N**(-1/3)) # Number of bins 
131          #print N, IQR, R, bins 
132          return min(150, max(10, bins))

133   
134   


135 -    def plot_score_histogram(self, fname, pdata, ndata, threshold):


136          """Histograms for pos and neg scores, with line to mark threshold"""  
137          if fname.exists() and not self.overwrite: return 
138          g = self.gnuplot 
139          logging.debug("Plotting article score histogram to %s", fname.basename()) 
140          from itertools import chain 
141          py, px = nx.histogram(pdata, bins=self.bincount(pdata), normed=True) 
142          zy, zx = nx.histogram(ndata, bins=self.bincount(ndata), normed=True) 
143          g.reset() 
144          g("set terminal png") 
145          g("set output '%s'" % fname) 
146          g.title("Score Histograms") 
147          g.xlabel("Article Score") 
148          g.ylabel("Histogram Mass") 
149          ## Commented out arrow - rather adding a real line 
150          #g("set arrow from %f,0 to %f,%f nohead lw 4 " % ( 
151          #    threshold, threshold, max(chain(py,ny)))) 
152          g("set style fill solid 1.0") 
153          threshold_height = max(chain(py, zy)) 
154          g.plot(Data(px, py, title="Positives", with="boxes"), 
155                 Data(zx, zy, title="Negatives", with="boxes"), 
156                 Data([threshold, threshold], [0, threshold_height],  
157                      title="threshold", with="lines lw 3"))

158   
159   


160 -    def plot_feature_histogram(self, fname, scores):


161          """Histogram for feature scores 
162           
163          @param scores: List with scores of each feature""" 
164          if fname.exists() and not self.overwrite: return 
165          g = self.gnuplot 
166          logging.debug("Plotting feature score histogram to %s", fname.basename()) 
167          sscores = scores.copy() 
168          sscores.sort() 
169          y, x = nx.histogram(scores, bins=self.bincount(sscores)) 
170          g.reset() 
171          g.title("Feature Score Histogram") 
172          g.xlabel("Feature Score") 
173          g.ylabel("Histogram Mass") 
174          g("set logscale y") 
175          g("set terminal png") 
176          g("set output '%s'" % fname) 
177          g("set style fill solid 1.0") 
178          g.plot(Data(x, y, with="boxes"))

179   
180   
181   


182 -class DensityPlotter(Plotter):


183      """Adds plotting of estimated Probability Density Functions for 
184      article and feature scores. 
185       
186      @deprecated: These methods are too computationally 
187      expensive to use interactively (about 40 seconds per graph). 
188      """ 
189   
190   
191      @staticmethod 


192 -    def gaussian_kernel_pdf(values, npoints=512):


193          """Given 1D values, return the probability density function 
194           
195          @param values: Sorted list of floats representing the sample 
196           
197          @param npoints: Number of equal-spaced points at which to estimate the PDF 
198           
199          @return: (xvalues, yvalues) for y=f(x) of the pdf. 
200          """ 
201          from scipy import stats 
202          points = nx.linspace(values[0], values[-1], npoints) 
203          density = stats.kde.gaussian_kde(nx.array(values)).evaluate(points) 
204          return points, density

205   
206   


207 -    def plot_score_density(self, fname, pdata, ndata, threshold):


208          """Probability density of pos and neg scores, with line to mark threshold 
209       
210          @param pdata: Scores of positive documents 
211          @param ndata: Scores of negative documents 
212          @param threshold: Threshold score for counting a document positive 
213          """  
214          if fname.exists() and not self.overwrite: return 
215          g = self.gnuplot 
216          from itertools import chain 
217          logging.debug("Plotting article score density to %s", fname.basename()) 
218          px, py = self.gaussian_kernel_pdf(pdata) 
219          zx, zy = self.gaussian_kernel_pdf(ndata) 
220          overlap = calculateOverlap(px, py, zx, zy) 
221          g.reset() 
222          g.title("Article Score Densities") 
223          g.ylabel("Probability Density") 
224          g.xlabel("Article score") 
225          g("set terminal png") 
226          g("set output '%s'" % fname) 
227          threshold_height = max(chain(py, zy)) 
228          g.plot(Data([threshold, threshold], [0, threshold_height],  
229                      title="threshold", with="lines"), 
230                 Data(px, py, title="Positives", with="lines"), 
231                 Data(zx, zy, title="Negatives", with="lines")) 
232          return overlap

233   
234   


235 -    def plot_feature_density(self, fname, scores):


236          """Probability density function for feature scores""" 
237          if fname.exists() and not self.overwrite: return 
238          g = self.gnuplot 
239          logging.debug("Plotting feature score density to %s", fname.basename()) 
240          x, y = self.gaussian_kernel_pdf(scores, npoints=1024) 
241          g.reset() 
242          g.title("Feature Score Density") 
243          g.xlabel("Feature Score") 
244          g.ylabel("Probability Density") 
245          g("set terminal png") 
246          g("set output '%s'" % fname) 
247          g.plot(Data(x, y, with="lines"))

248
```

  


| Trees | Indices | Help | | MScanner | | --- | |
| --- | --- | --- | --- | --- |

|  |  |
| --- | --- |
| Generated by Epydoc 3.0beta1 on Fri Nov 23 09:13:24 2007 | http://epydoc.sourceforge.net |
