# Supplementary material for: MScanner: a classifier for retrieving Medline citations
Source: BMC Bioinformatics. 2008 Feb 19;9:108. doi: 10.1186/1471-2105-9-108 (PMC2263023; doi:10.1186/1471-2105-9-108)
Supplement: Additional file 3 — Source code for MScanner. mscanner-20071123.zip is a ZIP archive containing the Python 2.5 source code for MScanner, licensed under the GNU General Public License. It also contains API documentation in HTML format. Updated versions will be made available at . [file 1471-2105-9-108-S3.zip › mscanner/help/api/mscanner.core.Plotter.DensityPlotter-class.html]

xml version="1.0" encoding="ascii"?


mscanner.core.Plotter.DensityPlotter


| Trees | Indices | Help | | MScanner | | --- | |
| --- | --- | --- | --- | --- |

|  |  |  |  |
| --- | --- | --- | --- |
| Package mscanner :: Package core :: Module Plotter :: Class DensityPlotter | |  | | --- | | [hide private] | | [frames] | no frames] | |

# Class DensityPlotter

source code  
  

```
Plotter --+
          |
         DensityPlotter
```

---

Adds plotting of estimated Probability Density Functions for article
and feature scores.  
  


---

**Deprecated:**
These methods are too computationally expensive to use interactively
(about 40 seconds per graph).


|  |  |  |  |
| --- | --- | --- | --- |
| |  |  | | --- | --- | | Instance Methods | [hide private] | | |
|  | |  |  | | --- | --- | | plot\_score\_density(self, fname, pdata, ndata, threshold)  Probability density of pos and neg scores, with line to mark threshold | source code | |
|  | |  |  | | --- | --- | | plot\_feature\_density(self, fname, scores)  Probability density function for feature scores | source code | |
| **Inherited from `Plotter`**: `__init__`, `plot_feature_histogram`, `plot_fmeasure`, `plot_precision`, `plot_predictions`, `plot_roc`, `plot_score_histogram` | |


|  |  |  |  |
| --- | --- | --- | --- |
| |  |  | | --- | --- | | Static Methods | [hide private] | | |
|  | |  |  | | --- | --- | | gaussian\_kernel\_pdf(values, npoints=512)  Given 1D values, return the probability density function | source code | |
| **Inherited from `Plotter`**: `bincount` | |


|  |  |  |  |
| --- | --- | --- | --- |
| |  |  | | --- | --- | | Instance Variables | [hide private] | | |
| **Inherited from `Plotter`**: `gnuplot`, `overwrite` | |


|  |  |  |  |
| --- | --- | --- | --- |
| |  |  | | --- | --- | | Method Details | [hide private] | | |

|  |  |  |
| --- | --- | --- |
| |  |  | | --- | --- | | gaussian\_kernel\_pdf(values, npoints=512)  *Static Method* | source code |  Given 1D values, return the probability density function Parameters:  - **`values`** - Sorted list of floats representing the sample - **`npoints`** - Number of equal-spaced points at which to estimate the PDF  Returns:  (xvalues, yvalues) for y=f(x) of the pdf. |

|  |  |  |
| --- | --- | --- |
| |  |  | | --- | --- | | plot\_score\_density(self, fname, pdata, ndata, threshold) | source code |  Probability density of pos and neg scores, with line to mark threshold Parameters:  - **`pdata`** - Scores of positive documents - **`ndata`** - Scores of negative documents - **`threshold`** - Threshold score for counting a document positive |

  


| Trees | Indices | Help | | MScanner | | --- | |
| --- | --- | --- | --- | --- |

|  |  |
| --- | --- |
| Generated by Epydoc 3.0beta1 on Fri Nov 23 09:13:21 2007 | http://epydoc.sourceforge.net |
