# Supplementary material for: MScanner: a classifier for retrieving Medline citations
Source: BMC Bioinformatics. 2008 Feb 19;9:108. doi: 10.1186/1471-2105-9-108 (PMC2263023; doi:10.1186/1471-2105-9-108)
Supplement: Additional file 3 — Source code for MScanner. mscanner-20071123.zip is a ZIP archive containing the Python 2.5 source code for MScanner, licensed under the GNU General Public License. It also contains API documentation in HTML format. Updated versions will be made available at . [file 1471-2105-9-108-S3.zip › mscanner/help/api/mscanner.core.Plotter.Plotter-class.html]

xml version="1.0" encoding="ascii"?


mscanner.core.Plotter.Plotter


| Trees | Indices | Help | | MScanner | | --- | |
| --- | --- | --- | --- | --- |

|  |  |  |  |
| --- | --- | --- | --- |
| Package mscanner :: Package core :: Module Plotter :: Class Plotter | |  | | --- | | [hide private] | | [frames] | no frames] | |

# Class Plotter

source code  
  

Known Subclasses:
:   DensityPlotter

---

Implements the plots used in MScanner

When adding a new analysis, the plotting function for its output
graphs should be added here.  
  


---

**Note:**
All methods take an fname parameter, which is the path to the
PNG file to which the graph will be written.


|  |  |  |  |
| --- | --- | --- | --- |
| |  |  | | --- | --- | | Instance Methods | [hide private] | | |
|  | |  |  | | --- | --- | | \_\_init\_\_(self, overwrite=True) | source code | |
|  | |  |  | | --- | --- | | plot\_predictions(self, fname, predicted\_low, predicted\_high)  Given PredictedMetrics instance, plot the predicted query performance (TPR and PPV vs number of results). | source code | |
|  | |  |  | | --- | --- | | plot\_roc(self, fname, FPR, TPR, marker\_FPR)  ROC curve (TPR vs FPR) | source code | |
|  | |  |  | | --- | --- | | plot\_precision(self, fname, TPR, PPV, marker\_TPR)  Precision vs recall | source code | |
|  | |  |  | | --- | --- | | plot\_fmeasure(self, fname, pscores, TPR, PPV, FM, FMa, threshold)  Precision, Recall, F-Measure vs threshold | source code | |
|  | |  |  | | --- | --- | | plot\_score\_histogram(self, fname, pdata, ndata, threshold)  Histograms for pos and neg scores, with line to mark threshold | source code | |
|  | |  |  | | --- | --- | | plot\_feature\_histogram(self, fname, scores)  Histogram for feature scores | source code | |


|  |  |  |  |
| --- | --- | --- | --- |
| |  |  | | --- | --- | | Static Methods | [hide private] | | |
|  | |  |  | | --- | --- | | bincount(data)  Calculate the best number of histogram bins for the data | source code | |


|  |  |  |  |
| --- | --- | --- | --- |
| |  |  | | --- | --- | | Instance Variables | [hide private] | | |
|  | gnuplot  The captive Gnuplot instance. |
|  | overwrite  If False, we no-op rather than overwrite an already existing graph. |


|  |  |  |  |
| --- | --- | --- | --- |
| |  |  | | --- | --- | | Method Details | [hide private] | | |

|  |  |  |
| --- | --- | --- |
| |  |  | | --- | --- | | plot\_predictions(self, fname, predicted\_low, predicted\_high) | source code |  Given PredictedMetrics instance, plot the predicted query performance (TPR and PPV vs number of results).   **Note:** Two predictions are given, corresponding to upper and lower bound guesses at the number of |

|  |  |  |
| --- | --- | --- |
| |  |  | | --- | --- | | bincount(data)  *Static Method* | source code |   Calculate the best number of histogram bins for the data Uses the formula *K = R/(2\*IQR\*N^(-1/3))* Parameters:  - **`data`** - Array of numbers, sorted in increasing order. |

|  |  |  |
| --- | --- | --- |
| |  |  | | --- | --- | | plot\_feature\_histogram(self, fname, scores) | source code |  Histogram for feature scores Parameters:  - **`scores`** - List with scores of each feature |

  


| Trees | Indices | Help | | MScanner | | --- | |
| --- | --- | --- | --- | --- |

|  |  |
| --- | --- |
| Generated by Epydoc 3.0beta1 on Fri Nov 23 09:13:21 2007 | http://epydoc.sourceforge.net |
