# Supplementary material for: MScanner: a classifier for retrieving Medline citations
Source: BMC Bioinformatics. 2008 Feb 19;9:108. doi: 10.1186/1471-2105-9-108 (PMC2263023; doi:10.1186/1471-2105-9-108)
Supplement: Additional file 3 — Source code for MScanner. mscanner-20071123.zip is a ZIP archive containing the Python 2.5 source code for MScanner, licensed under the GNU General Public License. It also contains API documentation in HTML format. Updated versions will be made available at . [file 1471-2105-9-108-S3.zip › mscanner/help/api/mscanner.core.Plotter.RetrievalPlot-class.html]

xml version="1.0" encoding="ascii"?


mscanner.core.Plotter.RetrievalPlot


| Trees | Indices | Help | | MScanner | | --- | |
| --- | --- | --- | --- | --- |

|  |  |  |  |
| --- | --- | --- | --- |
| Package mscanner :: Package core :: Module Plotter :: Class RetrievalPlot | |  | | --- | | [hide private] | | [frames] | no frames] | |

# Class RetrievalPlot

source code  
  

```
Gnuplot._Gnuplot.Gnuplot --+
                           |
                          RetrievalPlot
```

---

Plotting function for comparative retrieval test  
  


|  |  |  |  |
| --- | --- | --- | --- |
| |  |  | | --- | --- | | Instance Methods | [hide private] | | |
|  | |  |  | | --- | --- | | plot\_retrieved\_positives(g, fname, nretrieved, total)  Proportion of testing set retrieved, versus result rank | source code | |
| **Inherited from `Gnuplot._Gnuplot.Gnuplot`**: `__call__`, `__init__`, `clear`, `hardcopy`, `interact`, `load`, `plot`, `refresh`, `replot`, `reset`, `save`, `set`, `set_boolean`, `set_label`, `set_range`, `set_string`, `splot`, `title`, `xlabel`, `ylabel`  **Inherited from `Gnuplot._Gnuplot.Gnuplot`** (private): `_add_to_queue`, `_clear_queue` | |


|  |  |  |  |
| --- | --- | --- | --- |
| |  |  | | --- | --- | | Class Variables | [hide private] | | |
| **Inherited from `Gnuplot._Gnuplot.Gnuplot`**: `optiontypes` | |

| Trees | Indices | Help | | MScanner | | --- | |
| --- | --- | --- | --- | --- |

|  |  |
| --- | --- |
| Generated by Epydoc 3.0beta1 on Mon Nov 12 17:36:25 2007 | http://epydoc.sourceforge.net |
