# Supplementary material for: MScanner: a classifier for retrieving Medline citations
Source: BMC Bioinformatics. 2008 Feb 19;9:108. doi: 10.1186/1471-2105-9-108 (PMC2263023; doi:10.1186/1471-2105-9-108)
Supplement: Additional file 3 — Source code for MScanner. mscanner-20071123.zip is a ZIP archive containing the Python 2.5 source code for MScanner, licensed under the GNU General Public License. It also contains API documentation in HTML format. Updated versions will be made available at . [file 1471-2105-9-108-S3.zip › mscanner/help/api/mscanner.core.QueryManager-pysrc.html]

xml version="1.0" encoding="ascii"?


mscanner.core.QueryManager


| Trees | Indices | Help | | MScanner | | --- | |
| --- | --- | --- | --- | --- |

|  |  |  |  |
| --- | --- | --- | --- |
| Package mscanner :: Package core :: Module QueryManager | |  | | --- | | [hide private] | | [frames] | no frames] | |

# Source Code for Module mscanner.core.QueryManager

```
  1  """Environment for performing query-based analyses.""" 
  2   
  3  from __future__ import with_statement 
  4  from __future__ import division 
  5   
  6  import codecs 
  7  import logging 
  8  import numpy as nx 
  9  import time 
 10  from contextlib import closing 
 11  import warnings 
 12  warnings.simplefilter("ignore", UserWarning) 
 13   
 14  from mscanner.configuration import rc 
 15  from mscanner.medline import Shelf 
 16  from mscanner.medline.Databases import Databases 
 17  from mscanner.core.FeatureScores import FeatureScores, FeatureCounts 
 18  from mscanner.core import CitationTable, iofuncs 
 19  from mscanner.fastscores.ScoreCalculator import ScoreCalculator 
 20  from mscanner.fastscores.FeatureCounter import FeatureCounter 
 21   
 22   
 23  __copyright__ = "2007 Graham Poulter" 
 24  __author__ = "Graham Poulter <http://graham.poulter.googlepages.com>" 
 25  __license__ = """This program is free software: you can redistribute it and/or 
 26  modify it under the terms of the GNU General Public License as published by the 
 27  Free Software Foundation, either version 3 of the License, or (at your option) 
 28  any later version. 
 29   
 30  This program is distributed in the hope that it will be useful, but WITHOUT ANY 
 31  WARRANTY; without even the implied warranty of MERCHANTABILITY or FITNESS FOR A 
 32  PARTICULAR PURPOSE. See the GNU General Public License for more details. 
 33   
 34  You should have received a copy of the GNU General Public License along with 
 35  this program. If not, see <http://www.gnu.org/licenses/>.""" 
 36   
 37   


38 -class QueryManager:


39      """Class for performing a single query 
 40   
 41      @group Passed via constructor: outdir, dataset, limit, env, threshold,  
 42      prior, mindate, maxdate, t_mindate, t_maxdate 
 43       
 44      @ivar outdir: Path to directory for output files, which is created if it 
 45      does not exist. 
 46       
 47      @ivar dataset: Title of the dataset to use when printing reports 
 48       
 49      @ivar limit: Maximum number of results (may be fewer due to threshold) 
 50       
 51      @param env: L{Databases} to use (if None, we open them just for us). 
 52   
 53      @ivar threshold: Decision threshold for the classifier (default should be 0). 
 54      Use None to retrieve everything up to the result limit. 
 55       
 56      @ivar prior: Prior score to add to all article scores.  Use None 
 57      to estimate from the relative sizes of the input data. 
 58       
 59      @ivar mindate, maxdate: Min/max YYYYMMDD integer for query results (ignore 
 60      articles outside this range). 
 61       
 62      @ivar t_mindate, t_maxdate: Min/max YYYYMMDD integer for counting feature 
 63      occurrences in Medline background corpus (defaults to mindate, maxdate). 
 64       
 65       
 66       
 67      @ivar timestamp: Time at the start of the operation. 
 68       
 69      @ivar pmids: Sequence of input PubMed IDs (list/vector) from L{_load_input} 
 70       
 71      @ivar featinfo: FeatureScores with feature scores, from L{query} 
 72       
 73      @ivar inputs: List of (pmid, score) for input PMIDs 
 74       
 75      @ivar results: List of (pmid, score) for result PMIDs 
 76       
 77      @ivar notfound_pmids: List of input PMIDs not found in the database 
 78       
 79      @ivar logfile: logging.FileHandler for logging to output directory 
 80      """ 
 81   
 82   


83 -    def __init__(self, outdir, dataset, limit, env=None,  
 84                   threshold=None, prior=None,  
 85                   mindate=None, maxdate=None,  
 86                   t_mindate=None, t_maxdate=None):


87          # Set attributes from parameters 
 88          self.outdir = outdir 
 89          self.dataset = dataset 
 90          self.limit = limit 
 91          self.threshold = threshold 
 92          self.prior = prior 
 93          self.mindate = mindate 
 94          self.maxdate = maxdate 
 95          self.t_mindate = mindate if t_mindate is None else t_mindate 
 96          self.t_maxdate = maxdate if t_maxdate is None else t_maxdate 
 97          # Create output dir 
 98          if not outdir.exists(): 
 99              outdir.makedirs() 
100              outdir.chmod(0777) 
101          # Set more attributes 
102          self.timestamp = time.time() 
103          self.env = env if env else Databases() 
104          self.pmids = None 
105          self.featinfo = None 
106          self.inputs = None 
107          self.results = None 
108          self.notfound_pmids = None 
109          self.logfile = iofuncs.open_logfile(self.outdir/rc.report_logfile)

110   
111   


112 -    def __del__(self):


113          iofuncs.close_logfile(self.logfile)

114   
115   


116 -    def query(self, input, train_exclude=None):


117          """Performs a query given PubMed IDs as input 
118           
119          @param input: Path to a list of PubMed IDs, or the list itself. 
120           
121          @param train_exclude: PMIDs to exclude from background when training 
122          """ 
123          logging.info("START: Query for %s", self.dataset) 
124          if not self._load_input(input): 
125              return 
126          self._make_feature_info(train_exclude) 
127          try: 
128              self._load_results() 
129          except IOError:  
130              self._make_results() 
131              self._save_results()

132           
133           


134 -    def _load_input(self, input):


135          """Construct L{pmids} and L{notfound_pmids}. 
136           
137          @param input: Path to file listing PubMed IDs, or something convertible 
138          to a set PubMed IDs. 
139           
140          @return: True on success, False on failure.""" 
141          if isinstance(input, basestring): 
142              logging.info("Loading PubMed IDs from %s", input.basename()) 
143              self.pmids, self.notfound_pmids, exclude = \ 
144                  iofuncs.read_pmids_careful(input, self.env.featdb) 
145              iofuncs.write_pmids( 
146                  self.outdir/rc.report_input_broken, self.notfound_pmids) 
147          else: 
148              self.pmids = input # Hope its a list/vector 
149          if len(self.pmids) > 0: 
150              return True 
151          else: 
152              logging.error("No valid PubMed IDs in %s", input.basename()) 
153              iofuncs.no_valid_pmids_page( 
154                  self.outdir/rc.report_index, self.dataset, self.notfound_pmids) 
155              return False

156   
157   


158 -    def _make_feature_info(self, train_exclude=None):


159          """Generate the L{featinfo} attribute using the L{pmids} 
160          as examples of relevant citations. 
161           
162          @param train_exclude: PMIDs to exclude from background when training 
163          """ 
164          logging.info("Making scores for %d features", len(self.env.featmap)) 
165          # Parameters for the FeatureScores instance 
166          self.featinfo = FeatureScores( 
167              featmap = self.env.featmap, 
168              pseudocount = rc.pseudocount, 
169              mask = self.env.featmap.get_type_mask(rc.exclude_types), 
170              make_scores = rc.make_scores, 
171              get_postmask = rc.get_postmask) 
172           
173          # Count features from the positive articles 
174          pdocs = len(self.pmids) 
175          pos_counts = FeatureCounts( 
176              len(self.env.featmap), self.env.featdb, self.pmids) 
177           
178          # Background is all of Medline minus input examples 
179          if self.t_mindate is None and self.t_maxdate is None: 
180              logging.info("Background PMIDs = Medline - input PMIDs") 
181              ndocs = self.env.featmap.numdocs - len(self.pmids) 
182              neg_counts = nx.array(self.env.featmap.counts, nx.int32) - pos_counts 
183           
184          # Background is Medline within a specific date range 
185          else: 
186              logging.info("Background PMIDs = Medline between %s and %s",  
187                           str(self.t_mindate), str(self.t_maxdate)) 
188              # Have the option to exclude more than just training PMIDs 
189              if train_exclude is None: 
190                  train_exclude = self.pmids 
191              ndocs, neg_counts = FeatureCounter( 
192                  docstream = rc.featurestream, 
193                  numdocs = self.env.featmap.numdocs, 
194                  numfeats = len(self.env.featmap), 
195                  mindate = self.t_mindate, 
196                  maxdate = self.t_maxdate, 
197                  exclude = train_exclude).c_counts() 
198           
199          # Evaluating feature scores from the counts 
200          self.featinfo.update(pos_counts, neg_counts, pdocs, ndocs, self.prior)

201   
202   


203 -    def _load_results(self):


204          """Read L{inputs} and L{results} from the report directory""" 
205          self.inputs = list(iofuncs.read_scores(self.outdir/rc.report_input_scores)) 
206          self.results = list(iofuncs.read_scores(self.outdir/rc.report_result_scores)) 
207          logging.info("Loaded saved results for %s", self.dataset)

208   
209   


210 -    def _save_results(self):


211          """Write L{inputs} and L{results} with scores in the report directory.""" 
212          iofuncs.write_scores(self.outdir/rc.report_input_scores,  
213                               self.inputs, sort=True) 
214          iofuncs.write_scores(self.outdir/rc.report_result_scores,  
215                               self.results, sort=True)

216   
217   


218 -    def _make_results(self):


219          """Perform the query to generate L{inputs} and L{results}""" 
220          # Calculate decreasing (score, PMID) for input PMIDs 
221          logging.info("Finding scores for %d input documents", len(self.pmids)) 
222          self.inputs = zip( 
223              self.featinfo.scores_of(self.env.featdb, self.pmids),  
224              self.pmids) 
225          self.inputs.sort(reverse=True) 
226          # Calculate results as decreasing (score, PMID) 
227          logging.info("Find scores of Medline between dates %s to %s",  
228                       str(self.mindate), str(self.maxdate)) 
229          self.results = ScoreCalculator( 
230              rc.featurestream, 
231              self.env.featmap.numdocs, 
232              self.featinfo.scores, 
233              self.featinfo.base+self.featinfo.prior, 
234              self.limit, 
235              self.threshold, 
236              self.mindate, 
237              self.maxdate, 
238              set(self.pmids), 
239              ).score() 
240          logging.info("Got %d results (limit %d)", len(self.results), self.limit)

241   
242   


243 -    def write_report(self, maxreport=None):


244          """Write the HTML report for the query results 
245           
246          @note: Article database lookups are carried out beforehand because 
247          lookups while doing template output is extremely slow. 
248           
249          @param maxreport: Largest number of records to write to the HTML reports 
250          (L{maxreport} may override the result limit). 
251          """ 
252          # Cancel report if there are no results 
253          if self.results is None: return 
254           
255          logging.debug("Creating report for data set %s", self.dataset) 
256           
257          # By default report all results 
258          if maxreport is None or maxreport > len(self.results): 
259              maxreport = len(self.results) 
260           
261          logging.debug("Writing features to %s", rc.report_term_scores) 
262          with codecs.open(self.outdir/rc.report_term_scores, "wb", "utf-8") as f: 
263              self.featinfo.write_csv(f) 
264           
265          logging.debug("Writing citations to %s", rc.report_input_citations) 
266          self.inputs.sort(reverse=True) 
267          inputs = [ (s,self.env.artdb[str(p)]) for s,p in self.inputs] 
268          CitationTable.write_citations( 
269              "input", self.dataset, inputs,  
270              self.outdir/rc.report_input_citations,  
271              rc.citations_per_file) 
272           
273          logging.debug("Writing citations to %s", rc.report_result_citations) 
274          self.results.sort(reverse=True) 
275          outputs = [ (s,self.env.artdb[str(p)]) for s,p in self.results[:maxreport] ] 
276          CitationTable.write_citations( 
277              "output", self.dataset, outputs, 
278              self.outdir/rc.report_result_citations,  
279              rc.citations_per_file) 
280           
281          # Write ALL output citations to a single HTML, and a zip file 
282          if len(outputs) > 0: 
283              logging.debug("Writing citations to %s", rc.report_result_all) 
284              outfname = self.outdir/rc.report_result_all 
285              zipfname = str(outfname + ".zip") 
286              CitationTable.write_citations( 
287                  "output", self.dataset, outputs, outfname, len(outputs)) 
288              from zipfile import ZipFile, ZIP_DEFLATED 
289              with closing(ZipFile(zipfname, "w", ZIP_DEFLATED)) as zf: 
290                  zf.write(str(outfname), str(outfname.basename())) 
291           
292          # Index.html 
293          logging.debug("FINISH: Writing %s for %s", rc.report_index, self.dataset) 
294          from Cheetah.Template import Template 
295          with iofuncs.FileTransaction(self.outdir/rc.report_index, "w") as ft: 
296              Template(file=str(rc.templates/"results.tmpl"),  
297                       filter="Filter", searchList=dict(QM=self)).respond(ft)

298
```

  


| Trees | Indices | Help | | MScanner | | --- | |
| --- | --- | --- | --- | --- |

|  |  |
| --- | --- |
| Generated by Epydoc 3.0beta1 on Fri Nov 23 09:13:24 2007 | http://epydoc.sourceforge.net |
