# Supplementary material for: MScanner: a classifier for retrieving Medline citations
Source: BMC Bioinformatics. 2008 Feb 19;9:108. doi: 10.1186/1471-2105-9-108 (PMC2263023; doi:10.1186/1471-2105-9-108)
Supplement: Additional file 3 — Source code for MScanner. mscanner-20071123.zip is a ZIP archive containing the Python 2.5 source code for MScanner, licensed under the GNU General Public License. It also contains API documentation in HTML format. Updated versions will be made available at . [file 1471-2105-9-108-S3.zip › mscanner/help/api/mscanner.core.QueryManager.QueryManager-class.html]

xml version="1.0" encoding="ascii"?


mscanner.core.QueryManager.QueryManager


| Trees | Indices | Help | | MScanner | | --- | |
| --- | --- | --- | --- | --- |

|  |  |  |  |
| --- | --- | --- | --- |
| Package mscanner :: Package core :: Module QueryManager :: Class QueryManager | |  | | --- | | [hide private] | | [frames] | no frames] | |

# Class QueryManager

source code  
  
Class for performing a single query  
  


|  |  |  |  |
| --- | --- | --- | --- |
| |  |  | | --- | --- | | Instance Methods | [hide private] | | |
|  | |  |  | | --- | --- | | \_\_init\_\_(self, outdir, dataset, limit, env=None, threshold=None, prior=None, mindate=None, maxdate=None, t\_mindate=None, t\_maxdate=None) | source code | |
|  | |  |  | | --- | --- | | \_\_del\_\_(self) | source code | |
|  | |  |  | | --- | --- | | query(self, input, train\_exclude=None)  Performs a query given PubMed IDs as input | source code | |
|  | |  |  | | --- | --- | | \_load\_input(self, input)  Construct pmids and notfound\_pmids. | source code | |
|  | |  |  | | --- | --- | | \_make\_feature\_info(self, train\_exclude=None)  Generate the featinfo attribute using the pmids as examples of relevant citations. | source code | |
|  | |  |  | | --- | --- | | \_load\_results(self)  Read inputs and results from the report directory | source code | |
|  | |  |  | | --- | --- | | \_save\_results(self)  Write inputs and results with scores in the report directory. | source code | |
|  | |  |  | | --- | --- | | \_make\_results(self)  Perform the query to generate inputs and results | source code | |
|  | |  |  | | --- | --- | | write\_report(self, maxreport=None)  Write the HTML report for the query results | source code | |


|  |  |  |  |
| --- | --- | --- | --- |
| |  |  | | --- | --- | | Instance Variables | [hide private] | | |
|  | featinfo  FeatureScores with feature scores, from query |
|  | inputs  List of (pmid, score) for input PMIDs |
|  | logfile  logging.FileHandler for logging to output directory |
|  | notfound\_pmids  List of input PMIDs not found in the database |
|  | pmids  Sequence of input PubMed IDs (list/vector) from \_load\_input |
|  | results  List of (pmid, score) for result PMIDs |
|  | timestamp  Time at the start of the operation. |
| Passed via constructor | |
|  | dataset  Title of the dataset to use when printing reports |
|  | limit  Maximum number of results (may be fewer due to threshold) |
|  | maxdate  Min/max YYYYMMDD integer for query results (ignore articles outside this range). |
|  | mindate  Min/max YYYYMMDD integer for query results (ignore articles outside this range). |
|  | outdir  Path to directory for output files, which is created if it does not exist. |
|  | prior  Prior score to add to all article scores. |
|  | t\_maxdate  Min/max YYYYMMDD integer for counting feature occurrences in Medline background corpus (defaults to mindate, maxdate). |
|  | t\_mindate  Min/max YYYYMMDD integer for counting feature occurrences in Medline background corpus (defaults to mindate, maxdate). |
|  | threshold  Decision threshold for the classifier (default should be 0). |


|  |  |  |  |
| --- | --- | --- | --- |
| |  |  | | --- | --- | | Method Details | [hide private] | | |

|  |  |  |
| --- | --- | --- |
| |  |  | | --- | --- | | \_\_init\_\_(self, outdir, dataset, limit, env=None, threshold=None, prior=None, mindate=None, maxdate=None, t\_mindate=None, t\_maxdate=None)  *(Constructor)* | source code |   Parameters:  - **`env`** - Databases to use (if None, we open them just for   us). |

|  |  |  |
| --- | --- | --- |
| |  |  | | --- | --- | | query(self, input, train\_exclude=None) | source code |  Performs a query given PubMed IDs as input Parameters:  - **`input`** - Path to a list of PubMed IDs, or the list itself. - **`train_exclude`** - PMIDs to exclude from background when training |

|  |  |  |
| --- | --- | --- |
| |  |  | | --- | --- | | \_load\_input(self, input) | source code |  Construct pmids and notfound\_pmids. Parameters:  - **`input`** - Path to file listing PubMed IDs, or something convertible to a   set PubMed IDs.  Returns:  True on success, False on failure. |

|  |  |  |
| --- | --- | --- |
| |  |  | | --- | --- | | \_make\_feature\_info(self, train\_exclude=None) | source code |  Generate the featinfo attribute using the pmids as examples of relevant citations. Parameters:  - **`train_exclude`** - PMIDs to exclude from background when training |

|  |  |  |
| --- | --- | --- |
| |  |  | | --- | --- | | write\_report(self, maxreport=None) | source code |  Write the HTML report for the query results Parameters:  - **`maxreport`** - Largest number of records to write to the HTML reports (`maxreport` may override the result limit).  **Note:** Article database lookups are carried out beforehand because lookups while doing template output is extremely slow. |

  


|  |  |  |  |
| --- | --- | --- | --- |
| |  |  | | --- | --- | | Instance Variable Details | [hide private] | | |

|  |
| --- |
| priorPrior score to add to all article scores. Use None to estimate from the relative sizes of the input data. |

|  |
| --- |
| thresholdDecision threshold for the classifier (default should be 0). Use None to retrieve everything up to the result limit. |

  


| Trees | Indices | Help | | MScanner | | --- | |
| --- | --- | --- | --- | --- |

|  |  |
| --- | --- |
| Generated by Epydoc 3.0beta1 on Fri Nov 23 09:13:21 2007 | http://epydoc.sourceforge.net |
