# Supplementary material for: MScanner: a classifier for retrieving Medline citations
Source: BMC Bioinformatics. 2008 Feb 19;9:108. doi: 10.1186/1471-2105-9-108 (PMC2263023; doi:10.1186/1471-2105-9-108)
Supplement: Additional file 3 — Source code for MScanner. mscanner-20071123.zip is a ZIP archive containing the Python 2.5 source code for MScanner, licensed under the GNU General Public License. It also contains API documentation in HTML format. Updated versions will be made available at . [file 1471-2105-9-108-S3.zip › mscanner/help/api/mscanner.core.Storage-pysrc.html]

xml version="1.0" encoding="ascii"?


mscanner.core.Storage


| Trees | Indices | Help | | MScanner | | --- | |
| --- | --- | --- | --- | --- |

|  |  |  |  |
| --- | --- | --- | --- |
| Package mscanner :: Package core :: Module Storage | |  | | --- | | [hide private] | | [frames] | no frames] | |

# Source Code for Module mscanner.core.Storage

```
 1  """Dictionary subclasses supporting dotted access""" 
 2   
 3   
 4  __copyright__ = "2007 Graham Poulter" 
 5  __author__ = "Graham Poulter <http://graham.poulter.googlepages.com>" 
 6  __license__ = """This program is free software: you can redistribute it and/or 
 7  modify it under the terms of the GNU General Public License as published by the 
 8  Free Software Foundation, either version 3 of the License, or (at your option) 
 9  any later version. 
10   
11  This program is distributed in the hope that it will be useful, but WITHOUT ANY 
12  WARRANTY; without even the implied warranty of MERCHANTABILITY or FITNESS FOR A 
13  PARTICULAR PURPOSE. See the GNU General Public License for more details. 
14   
15  You should have received a copy of the GNU General Public License along with 
16  this program. If not, see <http://www.gnu.org/licenses/>.""" 
17   
18   
19   


20 -class Storage(dict):


21      """Dictionary supporting d.foo attribute access to keys. 
22       
23      Raises AttributeError instead of KeyError when attribute-style access 
24      fails.""" 


25 -    def __getattr__(self, key):


26          try: 
27              return self[key] 
28          except KeyError, k: 
29              raise AttributeError, k

30   


31 -    def __setattr__(self, key, value):

32          self[key] = value

33   


34 -    def __delattr__(self, key):


35          try: 
36              del self[key] 
37          except KeyError, k: 
38              raise AttributeError, k

39   


40 -    def __str__(self):


41          return "Storage(\n" + \ 
42                 "\n".join("   " + k + " = " + repr(v) + ","  
43                           for k, v in self.iteritems()) + "\n)"

44   


45 -    def __repr__(self):

46          return '<Storage ' + dict.__repr__(self) + '>' 
47   
48   
49   


50 -class RCStorage(Storage):


51      """Dictionary with attribute access and auto-calling of stored functions. 
52       
53      @note: d.foo returns d.foo() if d['foo'] is callable 
54   
55      Example:: 
56          rc = RCStorage() 
57          rc.bar = 2 
58          rc.foo = lambda: rc.bar + 2 
59          rc.foo == 4 
60          rc.bar = 3 
61          rc.foo == 5 
62      """ 
63   


64 -    def __getattr__(self, key):


65          v = Storage.__getattr__(self, key) 
66          return (v() if hasattr(v, "__call__") else v)

67   


68 -    def __str__(self):


69          result = "RCStorage(" 
70          for k, v in self.iteritems(): 
71              if hasattr(v, "__call__"): 
72                  v = v() 
73              result += "\n   " + k + " = " + str(v) + "," 
74          return result+"\n)"

75
```

  


| Trees | Indices | Help | | MScanner | | --- | |
| --- | --- | --- | --- | --- |

|  |  |
| --- | --- |
| Generated by Epydoc 3.0beta1 on Fri Nov 23 09:13:22 2007 | http://epydoc.sourceforge.net |
