# Supplementary material for: MScanner: a classifier for retrieving Medline citations
Source: BMC Bioinformatics. 2008 Feb 19;9:108. doi: 10.1186/1471-2105-9-108 (PMC2263023; doi:10.1186/1471-2105-9-108)
Supplement: Additional file 3 — Source code for MScanner. mscanner-20071123.zip is a ZIP archive containing the Python 2.5 source code for MScanner, licensed under the GNU General Public License. It also contains API documentation in HTML format. Updated versions will be made available at . [file 1471-2105-9-108-S3.zip › mscanner/help/api/mscanner.core.Storage.RCStorage-class.html]

xml version="1.0" encoding="ascii"?


mscanner.core.Storage.RCStorage


| Trees | Indices | Help | | MScanner | | --- | |
| --- | --- | --- | --- | --- |

|  |  |  |  |
| --- | --- | --- | --- |
| Package mscanner :: Package core :: Module Storage :: Class RCStorage | |  | | --- | | [hide private] | | [frames] | no frames] | |

# Class RCStorage

source code  
  

```
object --+        
         |        
      dict --+    
             |    
       Storage --+
                 |
                RCStorage
```

---

Dictionary with attribute access and auto-calling of stored
functions.  
  


---

**Note:**

d.foo returns d.foo() if d['foo'] is callable

Example:

```
   rc = RCStorage()
   rc.bar = 2
   rc.foo = lambda: rc.bar + 2
   rc.foo == 4
   rc.bar = 3
   rc.foo == 5
```


|  |  |  |  |
| --- | --- | --- | --- |
| |  |  | | --- | --- | | Instance Methods | [hide private] | | |
|  | |  |  | | --- | --- | | \_\_getattr\_\_(self, key) | source code | |
|  | |  |  | | --- | --- | | \_\_str\_\_(self) | source code | |
| **Inherited from `Storage`**: `__delattr__`, `__repr__`, `__setattr__`  **Inherited from `dict`**: `__cmp__`, `__contains__`, `__delitem__`, `__eq__`, `__ge__`, `__getattribute__`, `__getitem__`, `__gt__`, `__hash__`, `__init__`, `__iter__`, `__le__`, `__len__`, `__lt__`, `__ne__`, `__new__`, `__setitem__`, `clear`, `copy`, `fromkeys`, `get`, `has_key`, `items`, `iteritems`, `iterkeys`, `itervalues`, `keys`, `pop`, `popitem`, `setdefault`, `update`, `values`  **Inherited from `object`**: `__reduce__`, `__reduce_ex__` | |


|  |  |  |  |
| --- | --- | --- | --- |
| |  |  | | --- | --- | | Properties | [hide private] | | |
| **Inherited from `object`**: `__class__` | |


|  |  |  |  |
| --- | --- | --- | --- |
| |  |  | | --- | --- | | Method Details | [hide private] | | |

|  |  |  |
| --- | --- | --- |
| |  |  | | --- | --- | | \_\_getattr\_\_(self, key)  *(Qualification operator)* | source code |   Overrides: Storage.\_\_getattr\_\_ |

|  |  |  |
| --- | --- | --- |
| |  |  | | --- | --- | | \_\_str\_\_(self)  *(Informal representation operator)* | source code |   Overrides: Storage.\_\_str\_\_ |

  


| Trees | Indices | Help | | MScanner | | --- | |
| --- | --- | --- | --- | --- |

|  |  |
| --- | --- |
| Generated by Epydoc 3.0beta1 on Fri Nov 23 09:13:21 2007 | http://epydoc.sourceforge.net |
