# Supplementary material for: MScanner: a classifier for retrieving Medline citations
Source: BMC Bioinformatics. 2008 Feb 19;9:108. doi: 10.1186/1471-2105-9-108 (PMC2263023; doi:10.1186/1471-2105-9-108)
Supplement: Additional file 3 — Source code for MScanner. mscanner-20071123.zip is a ZIP archive containing the Python 2.5 source code for MScanner, licensed under the GNU General Public License. It also contains API documentation in HTML format. Updated versions will be made available at . [file 1471-2105-9-108-S3.zip › mscanner/help/api/mscanner.core.Storage.Storage-class.html]

xml version="1.0" encoding="ascii"?


mscanner.core.Storage.Storage


| Trees | Indices | Help | | MScanner | | --- | |
| --- | --- | --- | --- | --- |

|  |  |  |  |
| --- | --- | --- | --- |
| Package mscanner :: Package core :: Module Storage :: Class Storage | |  | | --- | | [hide private] | | [frames] | no frames] | |

# Class Storage

source code  
  

```
object --+    
         |    
      dict --+
             |
            Storage
```

Known Subclasses:
:   RCStorage

---

Dictionary supporting d.foo attribute access to keys.

Raises AttributeError instead of KeyError when attribute-style access
fails.  
  


|  |  |  |  |
| --- | --- | --- | --- |
| |  |  | | --- | --- | | Instance Methods | [hide private] | | |
|  | |  |  | | --- | --- | | \_\_getattr\_\_(self, key) | source code | |
|  | |  |  | | --- | --- | | \_\_setattr\_\_(self, key, value)  x.\_\_setattr\_\_('name', value) <==> x.name = value | source code | |
|  | |  |  | | --- | --- | | \_\_delattr\_\_(self, key)  x.\_\_delattr\_\_('name') <==> del x.name | source code | |
|  | |  |  | | --- | --- | | \_\_str\_\_(self)  str(x) | source code | |
|  | |  |  | | --- | --- | | \_\_repr\_\_(self)  repr(x) | source code | |
| **Inherited from `dict`**: `__cmp__`, `__contains__`, `__delitem__`, `__eq__`, `__ge__`, `__getattribute__`, `__getitem__`, `__gt__`, `__hash__`, `__init__`, `__iter__`, `__le__`, `__len__`, `__lt__`, `__ne__`, `__new__`, `__setitem__`, `clear`, `copy`, `fromkeys`, `get`, `has_key`, `items`, `iteritems`, `iterkeys`, `itervalues`, `keys`, `pop`, `popitem`, `setdefault`, `update`, `values`  **Inherited from `object`**: `__reduce__`, `__reduce_ex__` | |


|  |  |  |  |
| --- | --- | --- | --- |
| |  |  | | --- | --- | | Properties | [hide private] | | |
| **Inherited from `object`**: `__class__` | |


|  |  |  |  |
| --- | --- | --- | --- |
| |  |  | | --- | --- | | Method Details | [hide private] | | |

|  |  |  |
| --- | --- | --- |
| |  |  | | --- | --- | | \_\_setattr\_\_(self, key, value) | source code |  x.\_\_setattr\_\_('name', value) <==> x.name = value Overrides: object.\_\_setattr\_\_ *(inherited documentation)* |

|  |  |  |
| --- | --- | --- |
| |  |  | | --- | --- | | \_\_delattr\_\_(self, key) | source code |  x.\_\_delattr\_\_('name') <==> del x.name Overrides: object.\_\_delattr\_\_ *(inherited documentation)* |

|  |  |  |
| --- | --- | --- |
| |  |  | | --- | --- | | \_\_str\_\_(self)  *(Informal representation operator)* | source code |  str(x) Overrides: object.\_\_str\_\_ *(inherited documentation)* |

|  |  |  |
| --- | --- | --- |
| |  |  | | --- | --- | | \_\_repr\_\_(self)  *(Representation operator)* | source code |  repr(x) Overrides: dict.\_\_repr\_\_ *(inherited documentation)* |

  


| Trees | Indices | Help | | MScanner | | --- | |
| --- | --- | --- | --- | --- |

|  |  |
| --- | --- |
| Generated by Epydoc 3.0beta1 on Fri Nov 23 09:13:21 2007 | http://epydoc.sourceforge.net |
