# Supplementary material for: MScanner: a classifier for retrieving Medline citations
Source: BMC Bioinformatics. 2008 Feb 19;9:108. doi: 10.1186/1471-2105-9-108 (PMC2263023; doi:10.1186/1471-2105-9-108)
Supplement: Additional file 3 — Source code for MScanner. mscanner-20071123.zip is a ZIP archive containing the Python 2.5 source code for MScanner, licensed under the GNU General Public License. It also contains API documentation in HTML format. Updated versions will be made available at . [file 1471-2105-9-108-S3.zip › mscanner/help/api/mscanner.core.ValidationManager-pysrc.html]

xml version="1.0" encoding="ascii"?


mscanner.core.ValidationManager


| Trees | Indices | Help | | MScanner | | --- | |
| --- | --- | --- | --- | --- |

|  |  |  |  |
| --- | --- | --- | --- |
| Package mscanner :: Package core :: Module ValidationManager | |  | | --- | | [hide private] | | [frames] | no frames] | |

# Source Code for Module mscanner.core.ValidationManager

```
  1  """Environment for performing cross-validation-based analyses""" 
  2   
  3  from __future__ import with_statement 
  4  from __future__ import division 
  5   
  6  import codecs 
  7  from itertools import chain, izip 
  8  import logging 
  9  import numpy as nx 
 10  import time 
 11   
 12  import warnings 
 13  warnings.simplefilter("ignore", UserWarning) 
 14   
 15  from mscanner.configuration import rc 
 16  from mscanner.medline.Databases import Databases 
 17  from mscanner.core import iofuncs 
 18  from mscanner.core.FeatureScores import FeatureScores, FeatureCounts 
 19  from mscanner.core.metrics import (PerformanceVectors, PerformanceRange,  
 20                                     PredictedMetrics) 
 21  from mscanner.core.Plotter import Plotter 
 22  from mscanner.core.Validator import CrossValidator 
 23   
 24   
 25  __copyright__ = "2007 Graham Poulter" 
 26  __author__ = "Graham Poulter <http://graham.poulter.googlepages.com>" 
 27  __license__ = """This program is free software: you can redistribute it and/or 
 28  modify it under the terms of the GNU General Public License as published by the 
 29  Free Software Foundation, either version 3 of the License, or (at your option) 
 30  any later version. 
 31   
 32  This program is distributed in the hope that it will be useful, but WITHOUT ANY 
 33  WARRANTY; without even the implied warranty of MERCHANTABILITY or FITNESS FOR A 
 34  PARTICULAR PURPOSE. See the GNU General Public License for more details. 
 35   
 36  You should have received a copy of the GNU General Public License along with 
 37  this program. If not, see <http://www.gnu.org/licenses/>.""" 
 38   
 39   
 40   


41 -class ValidationBase(object):


42      """Base class for all validation operations. 
 43       
 44      Derived classes need to calculate all attributes other than those set in 
 45      the constructor. The attributes are required by L{_write_report}. 
 46       
 47      @group Set in the constructor: env, outdir, dataset, timestamp 
 48   
 49      @ivar env: L{Databases} instance for accessing Medline 
 50   
 51      @ivar outdir: Path to directory for output files, which is created if it 
 52      does not exist. 
 53       
 54      @ivar dataset: Title of the dataset to use when printing reports 
 55   
 56      @ivar timestamp: Time at the start of the operation 
 57   
 58   
 59       
 60      @ivar pscores: Result scores for positive articles 
 61   
 62      @ivar nscores: Result scores for negative articles 
 63   
 64      @ivar featinfo: L{FeatureScores} instance for calculating feature scores 
 65   
 66      @ivar nfolds: Number of cross validation folds (may not be relevant) 
 67   
 68      @ivar notfound_pmids: List of input PMIDs not found in the database 
 69   
 70      @ivar metric_vectors: L{PerformanceVectors} instance 
 71   
 72      @ivar metric_range: L{PerformanceRange} instance 
 73       
 74      @ivar logfile: logging.FileHandler for logging to output directory 
 75      """ 
 76   
 77       


78 -    def __init__(self, outdir, dataset, env=None):


79          """Constructor""" 
 80          self.dataset = dataset 
 81          self.outdir = outdir 
 82          if not outdir.exists(): 
 83              outdir.makedirs() 
 84              outdir.chmod(0777) 
 85          self.env = env if env else Databases() 
 86          self.timestamp = time.time()  
 87          self.nfolds = None 
 88          self.pscores, self.nscores = None, None 
 89          self.featinfo = None 
 90          self.metric_vectors = None 
 91          self.metric_range = None 
 92          self.notfound_pmids = [] 
 93          self.logfile = iofuncs.open_logfile(self.outdir/rc.report_logfile)

 94   
 95   


96 -    def __del__(self):


97          iofuncs.close_logfile(self.logfile)

 98   
 99   


100 -    def _crossvalid_scores(self, positives, negatives):


101          """Calculate article scores under cross validation 
102           
103          @param positives: Vector of relevant PubMed IDs 
104           
105          @param negatives: Vector of irrelevant PubMed IDs 
106           
107          @note: Feature database lookups are slow so we cache them all 
108          beforehand in a dictionary. 
109           
110          @note: Before returning, we re-caculate feature scores in L{featinfo}  
111          using ALL of the training data. 
112           
113          @note: L{positives} and L{negatives} are scrambled so that they 
114          can be split into validation folds.  The returned scores correspond, 
115          so you can zip(positives, pscores) and zip(negatives, nscores) to 
116          pair up the scores with the articles. 
117           
118          @return: Two vectors, containing scores for the positive and negative 
119          articles respectively (unsorted for reconstruction of folds).""" 
120          self.validator = CrossValidator( 
121              featdb = dict((k,self.env.featdb[k]) for k in  
122                            chain(positives,negatives)), 
123              featinfo = self.featinfo, 
124              positives = positives, 
125              negatives = negatives, 
126              nfolds = self.nfolds) 
127          pscores, nscores = self.validator.validate() 
128          # Finally set feature scores using all available data 
129          self._update_featscores(positives, negatives) 
130          return pscores, nscores

131   
132   


133 -    def _get_performance(self, threshold=None):


134          """Calculate performance statistics. 
135           
136          @param threshold: Specify a particular threshold, or None to estimate 
137          using F measure.""" 
138          logging.info("Eval performance using alpha=%s, utility_r=%s",  
139                       str(rc.alpha), str(rc.utility_r)) 
140          v = PerformanceVectors(self.pscores, self.nscores, rc.alpha, rc.utility_r) 
141          self.metric_vectors = v 
142          if threshold is None: 
143              threshold, idx = v.threshold_maximising(v.FMa) 
144          else: 
145              threshold, idx = v.index_for(threshold) 
146          logging.info("Threshold is %f (uscores index=%d)", threshold, idx) 
147          average = v.metrics_for(idx) 
148          self.metric_range = PerformanceRange( 
149              self.pscores, self.nscores, self.nfolds, threshold, average)

150   
151   


152 -    def _init_featinfo(self):


153          """Initialise L{featinfo} for use in validation""" 
154          self.featinfo = FeatureScores( 
155              featmap = self.env.featmap,  
156              pseudocount = rc.pseudocount,  
157              mask = self.env.featmap.get_type_mask(rc.exclude_types), 
158              make_scores = rc.make_scores, 
159              get_postmask = rc.get_postmask)

160   
161   


162 -    def _update_featscores(self, pos, neg):


163          """Update the feature scores in L{featinfo} using the given  
164          vectors of positive and negative citations.""" 
165          self.featinfo.update( 
166              pos_counts = FeatureCounts( 
167                  len(self.env.featmap), self.env.featdb, pos), 
168              neg_counts = FeatureCounts( 
169                  len(self.env.featmap), self.env.featdb, neg), 
170              pdocs = len(pos), 
171              ndocs = len(neg))

172   
173   


174 -    def _write_report(self):


175          """Write an HTML validation report.  
176           
177          Only redraws figures for which output files do not already exist 
178          (likewise for term scores, but the index is always re-written).""" 
179          # Write term scores to file 
180          if not (self.outdir/rc.report_term_scores).exists(): 
181              logging.debug("Writing features scores to %s", rc.report_term_scores) 
182              with codecs.open(self.outdir/rc.report_term_scores, "wb", "utf-8") as f: 
183                  self.featinfo.write_csv(f) 
184          # Aliases for the performance data 
185          p = self.metric_vectors 
186          t = self.metric_range.average 
187          # Do not overwriting existing plots 
188          plotter = Plotter(overwrite=False)  
189          # Predicted precision/recall performance 
190          if hasattr(self, "pred_low") and hasattr(self, "pred_high"): 
191              plotter.plot_predictions(self.outdir/rc.report_prediction_img,  
192                                       self.pred_low, self.pred_high) 
193          # Report cross validation results instead of prediction results 
194          else: 
195              # ROC curve 
196              plotter.plot_roc( 
197                  self.outdir/rc.report_roc_img, p.FPR, p.TPR, t.FPR) 
198              # Precision-recall curve 
199              plotter.plot_precision( 
200                  self.outdir/rc.report_prcurve_img, p.TPR, p.PPV, t.TPR) 
201              # F-Measure curve 
202              plotter.plot_fmeasure( 
203                  self.outdir/rc.report_fmeasure_img, p.uscores, p.TPR, p.PPV,  
204                  p.FM, p.FMa, self.metric_range.threshold) 
205          # Article score histogram 
206          plotter.plot_score_histogram( 
207              self.outdir/rc.report_artscores_img, p.pscores, p.nscores,  
208              self.metric_range.threshold) 
209          # Feature score histogram 
210          plotter.plot_feature_histogram( 
211              self.outdir/rc.report_featscores_img, self.featinfo.scores) 
212          # Write index file 
213          logging.debug("FINISH: Writing %s for %s", rc.report_index, self.dataset) 
214          from Cheetah.Template import Template 
215          with iofuncs.FileTransaction(self.outdir/rc.report_index, "w") as ft: 
216              Template(file=str(rc.templates/"validation.tmpl"),  
217                       filter="Filter", searchList=dict(VM=self)).respond(ft)

218   
219   
220   


221 -def SplitValidation(ValidationBase):


222      """Carries out split-sample validation, as in the 2005 TREC 
223      Genomics Track categorisation task. 
224      """ 
225   
226      def validation(self, fptrain, fntrain, fptest, fntest): 
227          """Carry out split-sample validation.   
228           
229          @note: All corpora are represented as lists of PubMed IDs. 
230           
231          @param fptrain: File with positive training examples 
232           
233          @param fntrain: File with negative training examples 
234   
235          @param fptest: File with positive testing examples 
236   
237          @param fntest: File with negative testing examples 
238          """ 
239          logging.info("START: Split validation for %s", self.dataset) 
240          s = self 
241          s.nfolds = 1 # Effectively a single fold 
242          s.ptrain, s.ntrain = None, None 
243          s.ptest, s.ntest = None, None 
244          s.notfound_pmids = [] 
245          s.ptrain, broke, excl = iofuncs.read_pmids_careful(fptrain, s.env.featdb) 
246          s.ptest, broke, excl = iofuncs.read_pmids_careful(fptest, s.env.featdb) 
247          s.ntrain, broke, excl = iofuncs.read_pmids_careful( 
248              fntrain, s.env.featdb, set(s.ptrain)) 
249          s.ntest, broke, excl = iofuncs.read_pmids_careful( 
250              fntest, s.env.featdb, set(s.ptest)) 
251          if len(s.ptrain)>0 and len(s.ptest)>0 \ 
252             and len(s.ntrain)>0 and len(s.ntest)>0: 
253              s._init_featinfo() 
254              s._test_scores() 
255              s._get_performance() 
256              s._write_report() 
257          else: 
258              logging.error("At least one input file contained no valid PubMed IDs") 
259              return

260   
261   
262      def _test_scores(self): 
263          """Get performance statistics using split validation.  
264           
265          The training sample is used to calculate feature scores, which are then 
266          used to get the scores of the testing sample. Cross validation is used 
267          on the training sample to calculate a threshold optimising utility. The 
268          threshold is then applied to the testing sample to obtain performance 
269          metrics.""" 
270          s = self 
271          # Calculate cross-validated scores on training data 
272          train_pscores, train_nscores = s._crossvalid_scores(s.ptrain, s.ntrain) 
273          # Calculate split-sample scores on the testing data 
274          s.pscores = s.featinfo.scores_of(s.env.featdb, s.ptest) 
275          s.nscores = s.featinfo.scores_of(s.env.featdb, s.ntest) 
276          # Cross validation on training data for a threshold maximising utility 
277          trainperf = PerformanceVectors( 
278              train_pscores, train_nscores, rc.alpha, rc.utility_r) 
279          threshold = trainperf.threshold_maximising(trainperf.U)[0] 
280          # Apply that threshold to calculating performance on the test split 
281          self._get_performance(threshold) 
282   
283   
284   


285 -class CrossValidation(ValidationBase):


286      """Carries out N-fold cross validation. 
287   
288      @group Additional attributes: positives, negatives 
289   
290      @ivar positives: IDs of positive articles 
291   
292      @ivar negatives: IDs of negative articles 
293      """ 
294       
295   


296 -    def validation(self, pos, neg, nfolds=10):


297          """Loads data and perform cross validation to calculate scores 
298          on that data. 
299           
300          @note: This saves articles scores to the report directory, and  
301          if possible it will load load those scores instead of calculating 
302          from scratch. 
303           
304          @param pos, neg: Parameters for L{_load_input} 
305           
306          @param nfolds: Number of validation folds to use. 
307          """ 
308          logging.info("START: Cross validation for %s", self.dataset) 
309          # Keep our own number of folds attribute 
310          self.nfolds = nfolds 
311          self.notfound_pmids = [] 
312          self._init_featinfo() 
313          # Try to load saved results 
314          try: 
315              self.positives, self.pscores = iofuncs.read_scores_array( 
316                  self.outdir/rc.report_positives) 
317              self.negatives, self.nscores = iofuncs.read_scores_array( 
318                  self.outdir/rc.report_negatives) 
319              self._update_featscores(self.positives, self.negatives) 
320          # Failed to load, so perform cross validation 
321          except IOError: 
322              if not self._load_input(pos, neg): 
323                  return 
324              self.pscores, self.nscores = \ 
325                  self._crossvalid_scores(self.positives, self.negatives) 
326              iofuncs.write_scores(self.outdir/rc.report_positives, 
327                                   izip(self.pscores, self.positives)) 
328              iofuncs.write_scores(self.outdir/rc.report_negatives,  
329                                   izip(self.nscores, self.negatives))

330   
331   


332 -    def report_validation(self):


333          """Report cross validation results, using default threshold of 0""" 
334          if len(self.positives)>0 and len(self.negatives)>0: 
335              self._get_performance(0.0) 
336              self._write_report()

337   
338       


339 -    def report_predicted(self, relevant_low, relevant_high, medline_size):


340          """Experimental: report predicted query performance 
341           
342          @param relevant_low: Minimum expected relevant articles in Medline 
343           
344          @param relevant_high: Maximum expected relevant articles in Medline 
345   
346          @param medline_size: Number of articles in rest of Medline, or None 
347          to use L{Databases.article_list} minus relevant articles. 
348          """ 
349          if len(self.positives)>0 and len(self.negatives)>0: 
350              # Calculate the performance 
351              self._get_performance() 
352              if medline_size is None: 
353                  medline_size = len(self.env.article_list) - len(self.positives) 
354              v = self.metric_vectors 
355              self.pred_low = PredictedMetrics( 
356                  v.TPR, v.FPR, v.uscores, relevant_low, medline_size) 
357              self.pred_high = PredictedMetrics( 
358                  v.TPR, v.FPR, v.uscores, relevant_high, medline_size) 
359              self._write_report()

360   
361   


362 -    def _load_input(self, pos, neg):


363          """Sets L{positives} and L{negatives} by various means 
364           
365          @param pos: Path to file of input PubMed IDs, or something convertible 
366          to an integer array. 
367   
368          @param neg: Path to file of input negative PMIDs, or something 
369          convertible to integer array, or an integer representing the 
370          number of PubMed IDs to select at random from the database. 
371           
372          @return: True if the load was successful, False otherwise. 
373          """ 
374          if isinstance(pos, basestring): 
375              logging.info("Loading positive PubMed IDs from %s", pos.basename()) 
376              self.positives, self.notfound_pmids, exclude = \ 
377                  iofuncs.read_pmids_careful(pos, self.env.featdb) 
378          else: 
379              self.positives = nx.array(pos, nx.int32) 
380          if isinstance(neg, int): 
381              logging.info("Selecting %d random negative PubMed IDs" % neg) 
382              # Clamp number of negatives to the number available 
383              maxnegs = len(self.env.article_list) - len(self.positives) 
384              if neg > maxnegs: 
385                  neg = maxnegs 
386              # Take a sample of random citations 
387              self.negatives = self._random_subset( 
388                  neg, self.env.article_list, set(self.positives)) 
389          elif isinstance(neg, basestring): 
390              logging.info("Loading negative PubMed IDs from %s", neg.basename()) 
391              # Read list of negative PMIDs from disk 
392              self.negatives, notfound, exclude = iofuncs.read_pmids_careful( 
393                      neg, self.env.featdb, set(self.positives)) 
394              self.notfound_pmids = list(self.notfound_pmids) + list(notfound) 
395              iofuncs.write_pmids(self.outdir/rc.report_negatives_exclude, exclude) 
396          else: 
397              self.negatives = nx.array(neg, nx.int32) 
398          # Writing out broken PubMed IDs 
399          iofuncs.write_pmids( 
400              self.outdir/rc.report_input_broken, self.notfound_pmids) 
401          # Checking that we have the input 
402          if len(self.positives)>0 and len(self.negatives)>0: 
403              return True 
404          else: 
405              logging.error("No valid PubMed IDs in at least one input (error page)") 
406              iofuncs.no_valid_pmids_page( 
407                  self.outdir/rc.report_index, self.dataset, self.notfound_pmids) 
408              return False 
409          return True

410   
411   
412      @staticmethod 


413 -    def _random_subset(k, pool, exclude):


414          """Choose a random subset of k articles from pool 
415           
416          This is a good algorithm when the pool is large (say, 16 million 
417          items), we don't mind if the order of pool gets scrambled, and we have 
418          to exclude certain items from being selected. 
419           
420          @param k: Number of items to choose from pool 
421          @param pool: Array of items to choose from (will be scrambled!) 
422          @param exclude: Set of items that may not be chosen 
423          @return: A new array of the chosen items 
424          """ 
425          from random import randint 
426          import numpy as nx 
427          n = len(pool) 
428          assert 0 <= k <= n 
429          for i in xrange(k): 
430              # Non-selected items are in 0 ... n-i-1 
431              # Selected items are n-i ... n 
432              dest = n-i-1 
433              choice = randint(0, dest) # 0 ... n-i-1 inclusive 
434              while pool[choice] in exclude: 
435                  choice = randint(0, dest) 
436              # Move the chosen item to the end, where so it will be part of the 
437              # selected items in the next iteration. Note: this works using single 
438              # items - it but would break with slices due to their being views into 
439              # the vector. 
440              pool[dest], pool[choice] = pool[choice], pool[dest] 
441          # Phantom iteration: selected are n-k ... n 
442          return nx.array(pool[n-k:])

443
```

  


| Trees | Indices | Help | | MScanner | | --- | |
| --- | --- | --- | --- | --- |

|  |  |
| --- | --- |
| Generated by Epydoc 3.0beta1 on Fri Nov 23 09:13:24 2007 | http://epydoc.sourceforge.net |
