# Supplementary material for: MScanner: a classifier for retrieving Medline citations
Source: BMC Bioinformatics. 2008 Feb 19;9:108. doi: 10.1186/1471-2105-9-108 (PMC2263023; doi:10.1186/1471-2105-9-108)
Supplement: Additional file 3 — Source code for MScanner. mscanner-20071123.zip is a ZIP archive containing the Python 2.5 source code for MScanner, licensed under the GNU General Public License. It also contains API documentation in HTML format. Updated versions will be made available at . [file 1471-2105-9-108-S3.zip › mscanner/help/api/mscanner.core.ValidationManager.CrossValidation-class.html]

xml version="1.0" encoding="ascii"?


mscanner.core.ValidationManager.CrossValidation


| Trees | Indices | Help | | MScanner | | --- | |
| --- | --- | --- | --- | --- |

|  |  |  |  |
| --- | --- | --- | --- |
| Package mscanner :: Package core :: Module ValidationManager :: Class CrossValidation | |  | | --- | | [hide private] | | [frames] | no frames] | |

# Class CrossValidation

source code  
  

```
    object --+    
             |    
ValidationBase --+
                 |
                CrossValidation
```

---

Carries out N-fold cross validation.  
  


|  |  |  |  |
| --- | --- | --- | --- |
| |  |  | | --- | --- | | Instance Methods | [hide private] | | |
|  | |  |  | | --- | --- | | validation(self, pos, neg, nfolds=10)  Loads data and perform cross validation to calculate scores on that data. | source code | |
|  | |  |  | | --- | --- | | report\_validation(self)  Report cross validation results, using default threshold of 0 | source code | |
|  | |  |  | | --- | --- | | report\_predicted(self, relevant\_low, relevant\_high, medline\_size)  Experimental: report predicted query performance | source code | |
|  | |  |  | | --- | --- | | \_load\_input(self, pos, neg)  Sets positives and negatives by various means | source code | |
| **Inherited from `ValidationBase`**: `__del__`, `__init__`  **Inherited from `ValidationBase`** (private): `_crossvalid_scores`, `_get_performance`, `_init_featinfo`, `_update_featscores`, `_write_report`  **Inherited from `object`**: `__delattr__`, `__getattribute__`, `__hash__`, `__new__`, `__reduce__`, `__reduce_ex__`, `__repr__`, `__setattr__`, `__str__` | |


|  |  |  |  |
| --- | --- | --- | --- |
| |  |  | | --- | --- | | Static Methods | [hide private] | | |
|  | |  |  | | --- | --- | | \_random\_subset(k, pool, exclude)  Choose a random subset of k articles from pool | source code | |


|  |  |  |  |
| --- | --- | --- | --- |
| |  |  | | --- | --- | | Instance Variables | [hide private] | | |
| **Inherited from `ValidationBase`**: `featinfo`, `logfile`, `metric_range`, `metric_vectors`, `nfolds`, `notfound_pmids`, `nscores`, `pscores` | |
| Additional attributes | |
|  | negatives  IDs of negative articles |
|  | positives  IDs of positive articles |
| Set in the constructor | |
| **Inherited from `ValidationBase`**: `dataset`, `env`, `outdir`, `timestamp` | |


|  |  |  |  |
| --- | --- | --- | --- |
| |  |  | | --- | --- | | Properties | [hide private] | | |
| **Inherited from `object`**: `__class__` | |


|  |  |  |  |
| --- | --- | --- | --- |
| |  |  | | --- | --- | | Method Details | [hide private] | | |

|  |  |  |
| --- | --- | --- |
| |  |  | | --- | --- | | validation(self, pos, neg, nfolds=10) | source code |  Loads data and perform cross validation to calculate scores on that data. Parameters:  - **`pos`**, **`neg`** - Parameters for \_load\_input - **`nfolds`** - Number of validation folds to use.  **Note:** This saves articles scores to the report directory, and if possible it will load load those scores instead of calculating from scratch. |

|  |  |  |
| --- | --- | --- |
| |  |  | | --- | --- | | report\_predicted(self, relevant\_low, relevant\_high, medline\_size) | source code |  Experimental: report predicted query performance Parameters:  - **`relevant_low`** - Minimum expected relevant articles in Medline - **`relevant_high`** - Maximum expected relevant articles in Medline - **`medline_size`** - Number of articles in rest of Medline, or None to use Databases.article\_list minus relevant   articles. |

|  |  |  |
| --- | --- | --- |
| |  |  | | --- | --- | | \_load\_input(self, pos, neg) | source code |  Sets positives and negatives by various means Parameters:  - **`pos`** - Path to file of input PubMed IDs, or something convertible to   an integer array. - **`neg`** - Path to file of input negative PMIDs, or something convertible   to integer array, or an integer representing the number of PubMed   IDs to select at random from the database.  Returns:  True if the load was successful, False otherwise. |

|  |  |  |
| --- | --- | --- |
| |  |  | | --- | --- | | \_random\_subset(k, pool, exclude)  *Static Method* | source code |   Choose a random subset of k articles from pool This is a good algorithm when the pool is large (say, 16 million items), we don't mind if the order of pool gets scrambled, and we have to exclude certain items from being selected. Parameters:  - **`k`** - Number of items to choose from pool - **`pool`** - Array of items to choose from (will be scrambled!) - **`exclude`** - Set of items that may not be chosen  Returns:  A new array of the chosen items |

  


| Trees | Indices | Help | | MScanner | | --- | |
| --- | --- | --- | --- | --- |

|  |  |
| --- | --- |
| Generated by Epydoc 3.0beta1 on Fri Nov 23 09:13:21 2007 | http://epydoc.sourceforge.net |
