# Supplementary material for: MScanner: a classifier for retrieving Medline citations
Source: BMC Bioinformatics. 2008 Feb 19;9:108. doi: 10.1186/1471-2105-9-108 (PMC2263023; doi:10.1186/1471-2105-9-108)
Supplement: Additional file 3 — Source code for MScanner. mscanner-20071123.zip is a ZIP archive containing the Python 2.5 source code for MScanner, licensed under the GNU General Public License. It also contains API documentation in HTML format. Updated versions will be made available at . [file 1471-2105-9-108-S3.zip › mscanner/help/api/mscanner.core.ValidationManager.SplitValidation-class.html]

xml version="1.0" encoding="ascii"?


mscanner.core.ValidationManager.SplitValidation


| Trees | Indices | Help | | MScanner | | --- | |
| --- | --- | --- | --- | --- |

|  |  |  |  |
| --- | --- | --- | --- |
| Package mscanner :: Package core :: Module ValidationManager :: Class SplitValidation | |  | | --- | | [hide private] | | [frames] | no frames] | |

# Class SplitValidation

source code  
  

Known Subclasses:
:   ValidationManager

---

Carries out split-sample validation.  
  


|  |  |  |  |
| --- | --- | --- | --- |
| |  |  | | --- | --- | | Instance Methods | [hide private] | | |
|  | |  |  | | --- | --- | | \_\_init\_\_(self, outdir, env=None)  Constructor | source code | |
|  | |  |  | | --- | --- | | validation(self, fptrain, fntrain, fptest, fntest)  Carry out split-sample validation | source code | |
|  | |  |  | | --- | --- | | \_init\_featinfo(self)  Initialise featinfo for use in validation | source code | |
|  | |  |  | | --- | --- | | \_calc\_test\_scores(self)  Use training sample for feature scores, then calc scores for testing sample. | source code | |
|  | |  |  | | --- | --- | | \_calc\_performance(self)  Calculate performance statistics | source code | |
|  | |  |  | | --- | --- | | \_write\_report(self)  Write an HTML validation report. | source code | |


|  |  |  |  |
| --- | --- | --- | --- |
| |  |  | | --- | --- | | Instance Variables | [hide private] | | |
|  | featinfo  FeatureScores for calculating feature score |
|  | notfound\_pmids  List of input PMIDs not found in the database |
|  | nscores  Result scores for negative articles |
|  | performance  PerformanceStats instance |
|  | perfrange  PerformanceRange instance |
|  | pscores  Result scores for positive articles |
|  | timestamp  Time at the start of the operation |


|  |  |  |  |
| --- | --- | --- | --- |
| |  |  | | --- | --- | | Method Details | [hide private] | | |

|  |  |  |
| --- | --- | --- |
| |  |  | | --- | --- | | validation(self, fptrain, fntrain, fptest, fntest) | source code |  Carry out split-sample validation Parameters:  - **`fptrain`** - File with positive training examples - **`fntrain`** - File with negative training examples - **`fptest`** - File with positive testing examples - **`fntest`** - File with negative testing examples |

|  |  |  |
| --- | --- | --- |
| |  |  | | --- | --- | | \_write\_report(self) | source code |   Write an HTML validation report. Only redraws figures for which output files do not already exist (likewise for term scores, but the index is always re-written). |

  


| Trees | Indices | Help | | MScanner | | --- | |
| --- | --- | --- | --- | --- |

|  |  |
| --- | --- |
| Generated by Epydoc 3.0beta1 on Fri Oct 26 21:01:05 2007 | http://epydoc.sourceforge.net |
