# Supplementary material for: MScanner: a classifier for retrieving Medline citations
Source: BMC Bioinformatics. 2008 Feb 19;9:108. doi: 10.1186/1471-2105-9-108 (PMC2263023; doi:10.1186/1471-2105-9-108)
Supplement: Additional file 3 — Source code for MScanner. mscanner-20071123.zip is a ZIP archive containing the Python 2.5 source code for MScanner, licensed under the GNU General Public License. It also contains API documentation in HTML format. Updated versions will be made available at . [file 1471-2105-9-108-S3.zip › mscanner/help/api/mscanner.core.ValidationManager.ValidationBase-class.html]

xml version="1.0" encoding="ascii"?


mscanner.core.ValidationManager.ValidationBase


| Trees | Indices | Help | | MScanner | | --- | |
| --- | --- | --- | --- | --- |

|  |  |  |  |
| --- | --- | --- | --- |
| Package mscanner :: Package core :: Module ValidationManager :: Class ValidationBase | |  | | --- | | [hide private] | | [frames] | no frames] | |

# Class ValidationBase

source code  
  

```
object --+
         |
        ValidationBase
```

Known Subclasses:
:   CrossValidation

---

Base class for all validation operations.

Derived classes need to calculate all attributes other than those set
in the constructor. The attributes are required by \_write\_report.  
  


|  |  |  |  |
| --- | --- | --- | --- |
| |  |  | | --- | --- | | Instance Methods | [hide private] | | |
|  | |  |  | | --- | --- | | \_\_init\_\_(self, outdir, dataset, env=None)  Constructor | source code | |
|  | |  |  | | --- | --- | | \_\_del\_\_(self) | source code | |
|  | |  |  | | --- | --- | | \_crossvalid\_scores(self, positives, negatives)  Calculate article scores under cross validation | source code | |
|  | |  |  | | --- | --- | | \_get\_performance(self, threshold=None)  Calculate performance statistics. | source code | |
|  | |  |  | | --- | --- | | \_init\_featinfo(self)  Initialise featinfo for use in validation | source code | |
|  | |  |  | | --- | --- | | \_update\_featscores(self, pos, neg)  Update the feature scores in featinfo using the given vectors of positive and negative citations. | source code | |
|  | |  |  | | --- | --- | | \_write\_report(self)  Write an HTML validation report. | source code | |
| **Inherited from `object`**: `__delattr__`, `__getattribute__`, `__hash__`, `__new__`, `__reduce__`, `__reduce_ex__`, `__repr__`, `__setattr__`, `__str__` | |


|  |  |  |  |
| --- | --- | --- | --- |
| |  |  | | --- | --- | | Instance Variables | [hide private] | | |
|  | featinfo  FeatureScores instance for calculating feature scores |
|  | logfile  logging.FileHandler for logging to output directory |
|  | metric\_range  PerformanceRange instance |
|  | metric\_vectors  PerformanceVectors instance |
|  | nfolds  Number of cross validation folds (may not be relevant) |
|  | notfound\_pmids  List of input PMIDs not found in the database |
|  | nscores  Result scores for negative articles |
|  | pscores  Result scores for positive articles |
| Set in the constructor | |
|  | dataset  Title of the dataset to use when printing reports |
|  | env  Databases instance for accessing Medline |
|  | outdir  Path to directory for output files, which is created if it does not exist. |
|  | timestamp  Time at the start of the operation |


|  |  |  |  |
| --- | --- | --- | --- |
| |  |  | | --- | --- | | Properties | [hide private] | | |
| **Inherited from `object`**: `__class__` | |


|  |  |  |  |
| --- | --- | --- | --- |
| |  |  | | --- | --- | | Method Details | [hide private] | | |

|  |  |  |
| --- | --- | --- |
| |  |  | | --- | --- | | \_\_init\_\_(self, outdir, dataset, env=None)  *(Constructor)* | source code |  Constructor Overrides: object.\_\_init\_\_ |

|  |  |  |
| --- | --- | --- |
| |  |  | | --- | --- | | \_crossvalid\_scores(self, positives, negatives) | source code |  Calculate article scores under cross validation Parameters:  - **`positives`** - Vector of relevant PubMed IDs - **`negatives`** - Vector of irrelevant PubMed IDs  Returns:  Two vectors, containing scores for the positive and negative articles respectively (unsorted for reconstruction of folds).  **Notes:**  - Feature database lookups are slow so we cache them all   beforehand in a dictionary. - Before returning, we re-caculate feature scores in featinfo using ALL of the training data. - `positives` and `negatives` are scrambled so that they can be   split into validation folds. The returned scores correspond, so   you can zip(positives, pscores) and zip(negatives, nscores) to pair   up the scores with the articles. |

|  |  |  |
| --- | --- | --- |
| |  |  | | --- | --- | | \_get\_performance(self, threshold=None) | source code |  Calculate performance statistics. Parameters:  - **`threshold`** - Specify a particular threshold, or None to estimate using F   measure. |

|  |  |  |
| --- | --- | --- |
| |  |  | | --- | --- | | \_write\_report(self) | source code |   Write an HTML validation report. Only redraws figures for which output files do not already exist (likewise for term scores, but the index is always re-written). |

  


| Trees | Indices | Help | | MScanner | | --- | |
| --- | --- | --- | --- | --- |

|  |  |
| --- | --- |
| Generated by Epydoc 3.0beta1 on Fri Nov 23 09:13:21 2007 | http://epydoc.sourceforge.net |
