# Supplementary material for: MScanner: a classifier for retrieving Medline citations
Source: BMC Bioinformatics. 2008 Feb 19;9:108. doi: 10.1186/1471-2105-9-108 (PMC2263023; doi:10.1186/1471-2105-9-108)
Supplement: Additional file 3 — Source code for MScanner. mscanner-20071123.zip is a ZIP archive containing the Python 2.5 source code for MScanner, licensed under the GNU General Public License. It also contains API documentation in HTML format. Updated versions will be made available at . [file 1471-2105-9-108-S3.zip › mscanner/help/api/mscanner.core.ValidationManager.ValidationManager-class.html]

xml version="1.0" encoding="ascii"?


mscanner.core.ValidationManager.ValidationManager


| Trees | Indices | Help | | MScanner | | --- | |
| --- | --- | --- | --- | --- |

|  |  |  |  |
| --- | --- | --- | --- |
| Package mscanner :: Package core :: Module ValidationManager :: Class ValidationManager | |  | | --- | | [hide private] | | [frames] | no frames] | |

# Class ValidationManager

source code  
  

```
SplitValidation --+
                  |
                 ValidationManager
```

---

Carries out N-fold cross validation.  
  


|  |  |  |  |
| --- | --- | --- | --- |
| |  |  | | --- | --- | | Instance Methods | [hide private] | | |
|  | |  |  | | --- | --- | | \_\_init\_\_(self, outdir, env=None)  Constructor, as for SplitValidation | source code | |
|  | |  |  | | --- | --- | | validation(self, pospath, negpath, nfolds)  Loads data, performs validation, and writes report | source code | |
|  | |  |  | | --- | --- | | \_load\_input(self, pospath, negpath)  Load positive and negative PubMed IDs for validation | source code | |
|  | |  |  | | --- | --- | | \_make\_results(self)  Calculate pscores and nscores using cross validation | source code | |
|  | |  |  | | --- | --- | | \_general\_feature\_scores(self)  Calculate feature scores using all citations | source code | |
| **Inherited from `SplitValidation`** (private): `_calc_performance`, `_calc_test_scores`, `_init_featinfo`, `_write_report` | |


|  |  |  |  |
| --- | --- | --- | --- |
| |  |  | | --- | --- | | Static Methods | [hide private] | | |
|  | |  |  | | --- | --- | | make\_random\_subset(k, pool, exclude)  Choose a random subset of k articles from pool | source code | |


|  |  |  |  |
| --- | --- | --- | --- |
| |  |  | | --- | --- | | Instance Variables | [hide private] | | |
|  | negatives  IDs of negative articles |
|  | nfolds  Number of validation folds |
|  | positives  IDs of positive articles |
| **Inherited from `SplitValidation`**: `featinfo`, `notfound_pmids`, `nscores`, `performance`, `perfrange`, `pscores`, `timestamp` | |


|  |  |  |  |
| --- | --- | --- | --- |
| |  |  | | --- | --- | | Method Details | [hide private] | | |

|  |  |  |
| --- | --- | --- |
| |  |  | | --- | --- | | \_\_init\_\_(self, outdir, env=None)  *(Constructor)* | source code |  Constructor, as for SplitValidation Overrides: SplitValidation.\_\_init\_\_ |

|  |  |  |
| --- | --- | --- |
| |  |  | | --- | --- | | validation(self, pospath, negpath, nfolds) | source code |  Loads data, performs validation, and writes report Parameters:  - **`pospath`** - File with positive PubMed IDs - **`negpath`** - File with negative PubMed IDs (or None to select randomly from   Medline) - **`nfolds`** - Number of validation folds.  Overrides: SplitValidation.validation |

|  |  |  |
| --- | --- | --- |
| |  |  | | --- | --- | | \_load\_input(self, pospath, negpath) | source code |  Load positive and negative PubMed IDs for validation Parameters:  - **`pospath`** - Location of input positive PMIDs - **`negpath`** - Location of input negative PMIDs |

|  |  |  |
| --- | --- | --- |
| |  |  | | --- | --- | | make\_random\_subset(k, pool, exclude)  *Static Method* | source code |   Choose a random subset of k articles from pool This is better than the usual algorithm when the pool is large (say, 16 million items), we don't mind if the order of pool gets scrambled, and we have to exclude certain items from being selected. Parameters:  - **`k`** - Number of items to choose from pool - **`pool`** - Array of items to choose from (will be scrambled!) - **`exclude`** - Set of items that may not be chosen  Returns:  A new array of the chosen items |

|  |  |  |
| --- | --- | --- |
| |  |  | | --- | --- | | \_make\_results(self) | source code |   Calculate pscores and nscores using cross validation All the feature database lookups are cached beforehand, as lookups while busy with validation are slow. |

  


| Trees | Indices | Help | | MScanner | | --- | |
| --- | --- | --- | --- | --- |

|  |  |
| --- | --- |
| Generated by Epydoc 3.0beta1 on Fri Oct 26 21:01:05 2007 | http://epydoc.sourceforge.net |
