# Supplementary material for: MScanner: a classifier for retrieving Medline citations
Source: BMC Bioinformatics. 2008 Feb 19;9:108. doi: 10.1186/1471-2105-9-108 (PMC2263023; doi:10.1186/1471-2105-9-108)
Supplement: Additional file 3 — Source code for MScanner. mscanner-20071123.zip is a ZIP archive containing the Python 2.5 source code for MScanner, licensed under the GNU General Public License. It also contains API documentation in HTML format. Updated versions will be made available at . [file 1471-2105-9-108-S3.zip › mscanner/help/api/mscanner.core.Validator-pysrc.html]

xml version="1.0" encoding="ascii"?


mscanner.core.Validator


| Trees | Indices | Help | | MScanner | | --- | |
| --- | --- | --- | --- | --- |

|  |  |  |  |
| --- | --- | --- | --- |
| Package mscanner :: Package core :: Module Validator | |  | | --- | | [hide private] | | [frames] | no frames] | |

# Source Code for Module mscanner.core.Validator

```
  1  """Cross-validation and performance statistic calculation""" 
  2   
  3  from __future__ import division 
  4  import logging 
  5  import numpy as nx 
  6   
  7  from mscanner import update 
  8  from mscanner.core.FeatureScores import FeatureScores, FeatureCounts 
  9   
 10   
 11  __copyright__ = "2007 Graham Poulter" 
 12  __author__ = "Graham Poulter <http://graham.poulter.googlepages.com>" 
 13  __license__ = """This program is free software: you can redistribute it and/or 
 14  modify it under the terms of the GNU General Public License as published by the 
 15  Free Software Foundation, either version 3 of the License, or (at your option) 
 16  any later version. 
 17   
 18  This program is distributed in the hope that it will be useful, but WITHOUT ANY 
 19  WARRANTY; without even the implied warranty of MERCHANTABILITY or FITNESS FOR A 
 20  PARTICULAR PURPOSE. See the GNU General Public License for more details. 
 21   
 22  You should have received a copy of the GNU General Public License along with 
 23  this program. If not, see <http://www.gnu.org/licenses/>.""" 
 24   
 25   


26 -class CrossValidator:


27      """Cross-validated calculation of article scores. 
 28       
 29      @group Constructor Parameters: featdb,featinfo,positives,negatives,nfolds,alpha,postfilter 
 30   
 31      @ivar featdb: Mapping from doc id to list of feature ids 
 32       
 33      @ivar featinfo: L{FeatureScores} instance to handle training 
 34       
 35      @ivar positives: Array of positive PMIDs for validation 
 36       
 37      @ivar negatives: Array of negative PMIDs for validation 
 38       
 39      @ivar nfolds: Number of validation folds 
 40   
 41       
 42      @group From validate: pscores,nscores 
 43       
 44      @ivar pscores: Scores of positive articles after validation 
 45       
 46      @ivar nscores: Scores of negative articles after validation 
 47      """ 
 48   


49 -    def __init__(self, featdb, featinfo, positives,  negatives, nfolds):


50          """Constructor parameters set corresponding instance attributes.""" 
 51          pscores = None 
 52          nscores = None 
 53          update(self, locals())

 54   
 55   
 56      @staticmethod 


57 -    def make_partitions(nitems, nparts):


58          """Calculate partitions of input data for cross validation 
 59           
 60          @param nitems: Number of items to partition 
 61          @param nparts: Number of partitions 
 62          @return: List of start indeces, and list of lengths for partitions 
 63          """ 
 64          base, rem = divmod(nitems, nparts) 
 65          sizes = base * nx.ones(nparts, nx.int32) 
 66          sizes[:rem] += 1 
 67          starts = nx.zeros(nparts, nx.int32) 
 68          starts[1:] = nx.cumsum(sizes[:-1]) 
 69          return starts, sizes

 70   
 71   


72 -    def validate(self, randomise=True):


73          """Perform n-fold validation and return the raw performance measures 
 74           
 75          @param randomise: Randomise validation splits (use False for debugging) 
 76           
 77          @return: L{pscores}, L{nscores} 
 78          """ 
 79          s = self 
 80          pdocs = len(s.positives) 
 81          ndocs = len(s.negatives) 
 82          logging.debug("Cross-validating %d pos and %d neg items", pdocs, ndocs) 
 83          if randomise: 
 84              nx.random.shuffle(s.positives) 
 85              nx.random.shuffle(s.negatives) 
 86          s.pstarts, s.psizes = s.make_partitions(pdocs, s.nfolds) 
 87          s.nstarts, s.nsizes = s.make_partitions(ndocs, s.nfolds) 
 88          s.pscores = nx.zeros(pdocs, nx.float32) 
 89          s.nscores = nx.zeros(ndocs, nx.float32) 
 90          pcounts = FeatureCounts(len(s.featinfo), s.featdb, s.positives) 
 91          ncounts = FeatureCounts(len(s.featinfo), s.featdb, s.negatives) 
 92          for fold, (pstart,psize,nstart,nsize) in \ 
 93              enumerate(zip(s.pstarts,s.psizes,s.nstarts,s.nsizes)): 
 94              logging.debug("Fold %d: pstart = %d, psize = %s; nstart = %d, nsize = %d",  
 95                        fold, pstart, psize, nstart, nsize) 
 96              # Get new feature scores 
 97              s.featinfo.update( 
 98                  pos_counts = pcounts - FeatureCounts( 
 99                      len(s.featinfo), s.featdb,  
100                      s.positives[pstart:pstart+psize]),  
101                  neg_counts = ncounts - FeatureCounts( 
102                      len(s.featinfo), s.featdb,  
103                      s.negatives[nstart:nstart+nsize]), 
104                  pdocs = pdocs-psize,  
105                  ndocs = ndocs-nsize, 
106                  prior = nx.log(pdocs/ndocs), 
107              ) 
108              # Calculate the article scores for the test fold 
109              s.pscores[pstart:pstart+psize] = s.featinfo.scores_of( 
110                  s.featdb, s.positives[pstart:pstart+psize]) 
111              s.nscores[nstart:nstart+nsize] = s.featinfo.scores_of( 
112                  s.featdb, s.negatives[nstart:nstart+nsize]) 
113          return s.pscores, s.nscores

114   
115   
116   


117 -class LeaveOutValidator(CrossValidator):


118      """Instead of N-fold cross validation, this class performs leave 
119      out one validation in which all but one of the citations is used 
120      to train the feature scores, which are then used to calculate 
121      the score of the left out document. 
122       
123      This is a lot slower than cross validation, although performance metrics 
124      are a bit higher. We have optimised the calculation of scores by 
125      calculating counts for all articles and just subtracting 1 for each feature 
126      present in the left out article. 
127       
128      Also, this version only has one scoring method: background Medline for 
129      pseudocounts, with prior probability of observation being 50%. """ 
130       


131 -    def validate(self):


132          """Performs leave-out-one validation, returning the resulting scores. 
133           
134           
135          @return: L{pscores}, L{nscores} 
136          """ 
137          # Set up base feature scores 
138          pcounts = FeatureCounts(len(self.featinfo), self.featdb, self.positives) 
139          ncounts = FeatureCounts(len(self.featinfo), self.featdb, self.negatives) 
140          self.pscores = nx.zeros(len(self.positives), nx.float32) 
141          self.nscores = nx.zeros(len(self.negatives), nx.float32) 
142          pdocs = len(self.positives) 
143          ndocs = len(self.negatives) 
144          mask = self.featinfo.mask 
145          # Set up pseudocount 
146          if isinstance(self.featinfo.pseudocount, nx.ndarray): 
147              ps = self.featinfo.pseudocount 
148          else: 
149              ps = nx.zeros(len(self.featinfo), nx.float32) + self.featinfo.pseudocount 
150          marker = 0 
151          # Discount this article in feature score calculations 
152          def score_of(pmid, p_mod, n_mod): 
153              f = [fid for fid in self.featdb[doc] if not mask or not mask[fid]] 
154              return nx.sum(nx.log( 
155                  ((pcounts[f]+p_mod+ps[f])/(pdocs+p_mod+2*ps[f]))/ 
156                  ((ncounts[f]+n_mod+ps[f])/(ndocs+n_mod+2*ps[f]))))

157          # Get scores for positive articles 
158          for idx, doc in enumerate(self.positives): 
159              self.pscores[idx] = score_of(doc, -1, 0) 
160          # Get scores for negative articles 
161          for idx, doc in enumerate(self.negatives): 
162              self.nscores[idx] = score_of(doc, 0, -1) 
163          return self.pscores, self.nscores

164
```

  


| Trees | Indices | Help | | MScanner | | --- | |
| --- | --- | --- | --- | --- |

|  |  |
| --- | --- |
| Generated by Epydoc 3.0beta1 on Fri Nov 23 09:13:22 2007 | http://epydoc.sourceforge.net |
