# Supplementary material for: MScanner: a classifier for retrieving Medline citations
Source: BMC Bioinformatics. 2008 Feb 19;9:108. doi: 10.1186/1471-2105-9-108 (PMC2263023; doi:10.1186/1471-2105-9-108)
Supplement: Additional file 3 — Source code for MScanner. mscanner-20071123.zip is a ZIP archive containing the Python 2.5 source code for MScanner, licensed under the GNU General Public License. It also contains API documentation in HTML format. Updated versions will be made available at . [file 1471-2105-9-108-S3.zip › mscanner/help/api/mscanner.core.Validator.CrossValidator-class.html]

xml version="1.0" encoding="ascii"?


mscanner.core.Validator.CrossValidator


| Trees | Indices | Help | | MScanner | | --- | |
| --- | --- | --- | --- | --- |

|  |  |  |  |
| --- | --- | --- | --- |
| Package mscanner :: Package core :: Module Validator :: Class CrossValidator | |  | | --- | | [hide private] | | [frames] | no frames] | |

# Class CrossValidator

source code  
  

Known Subclasses:
:   LeaveOutValidator

---

Cross-validated calculation of article scores.  
  


|  |  |  |  |
| --- | --- | --- | --- |
| |  |  | | --- | --- | | Instance Methods | [hide private] | | |
|  | |  |  | | --- | --- | | \_\_init\_\_(self, featdb, featinfo, positives, negatives, nfolds)  Constructor parameters set corresponding instance attributes. | source code | |
|  | |  |  | | --- | --- | | validate(self, randomise=True)  Perform n-fold validation and return the raw performance measures | source code | |


|  |  |  |  |
| --- | --- | --- | --- |
| |  |  | | --- | --- | | Static Methods | [hide private] | | |
|  | |  |  | | --- | --- | | make\_partitions(nitems, nparts)  Calculate partitions of input data for cross validation | source code | |


|  |  |  |  |
| --- | --- | --- | --- |
| |  |  | | --- | --- | | Instance Variables | [hide private] | | |
| Constructor Parameters | |
|  | featdb  Mapping from doc id to list of feature ids |
|  | featinfo  FeatureScores instance to handle training |
|  | negatives  Array of negative PMIDs for validation |
|  | nfolds  Number of validation folds |
|  | positives  Array of positive PMIDs for validation |
| From validate | |
|  | nscores  Scores of negative articles after validation |
|  | pscores  Scores of positive articles after validation |


|  |  |  |  |
| --- | --- | --- | --- |
| |  |  | | --- | --- | | Method Details | [hide private] | | |

|  |  |  |
| --- | --- | --- |
| |  |  | | --- | --- | | make\_partitions(nitems, nparts)  *Static Method* | source code |  Calculate partitions of input data for cross validation Parameters:  - **`nitems`** - Number of items to partition - **`nparts`** - Number of partitions  Returns:  List of start indeces, and list of lengths for partitions |

|  |  |  |
| --- | --- | --- |
| |  |  | | --- | --- | | validate(self, randomise=True) | source code |  Perform n-fold validation and return the raw performance measures Parameters:  - **`randomise`** - Randomise validation splits (use False for debugging)  Returns:  pscores, nscores |

  


| Trees | Indices | Help | | MScanner | | --- | |
| --- | --- | --- | --- | --- |

|  |  |
| --- | --- |
| Generated by Epydoc 3.0beta1 on Fri Nov 23 09:13:21 2007 | http://epydoc.sourceforge.net |
