# Supplementary material for: MScanner: a classifier for retrieving Medline citations
Source: BMC Bioinformatics. 2008 Feb 19;9:108. doi: 10.1186/1471-2105-9-108 (PMC2263023; doi:10.1186/1471-2105-9-108)
Supplement: Additional file 3 — Source code for MScanner. mscanner-20071123.zip is a ZIP archive containing the Python 2.5 source code for MScanner, licensed under the GNU General Public License. It also contains API documentation in HTML format. Updated versions will be made available at . [file 1471-2105-9-108-S3.zip › mscanner/help/api/mscanner.core.Validator.LeaveOutValidator-class.html]

xml version="1.0" encoding="ascii"?


mscanner.core.Validator.LeaveOutValidator


| Trees | Indices | Help | | MScanner | | --- | |
| --- | --- | --- | --- | --- |

|  |  |  |  |
| --- | --- | --- | --- |
| Package mscanner :: Package core :: Module Validator :: Class LeaveOutValidator | |  | | --- | | [hide private] | | [frames] | no frames] | |

# Class LeaveOutValidator

source code  
  

```
CrossValidator --+
                 |
                LeaveOutValidator
```

---

Instead of N-fold cross validation, this class performs leave out one
validation in which all but one of the citations is used to train the
feature scores, which are then used to calculate the score of the left
out document.

This is a lot slower than cross validation, although performance
metrics are a bit higher. We have optimised the calculation of scores by
calculating counts for all articles and just subtracting 1 for each
feature present in the left out article.

Also, this version only has one scoring method: background Medline for
pseudocounts, with prior probability of observation being 50%.  
  


|  |  |  |  |
| --- | --- | --- | --- |
| |  |  | | --- | --- | | Instance Methods | [hide private] | | |
|  | |  |  | | --- | --- | | validate(self)  Performs leave-out-one validation, returning the resulting scores. | source code | |
| **Inherited from `CrossValidator`**: `__init__` | |


|  |  |  |  |
| --- | --- | --- | --- |
| |  |  | | --- | --- | | Static Methods | [hide private] | | |
| **Inherited from `CrossValidator`**: `make_partitions` | |


|  |  |  |  |
| --- | --- | --- | --- |
| |  |  | | --- | --- | | Instance Variables | [hide private] | | |
| Constructor Parameters | |
| **Inherited from `CrossValidator`**: `featdb`, `featinfo`, `negatives`, `nfolds`, `positives` | |
| From validate | |
| **Inherited from `CrossValidator`**: `nscores`, `pscores` | |


|  |  |  |  |
| --- | --- | --- | --- |
| |  |  | | --- | --- | | Method Details | [hide private] | | |

|  |  |  |
| --- | --- | --- |
| |  |  | | --- | --- | | validate(self) | source code |  Performs leave-out-one validation, returning the resulting scores. Returns:  pscores, nscores  Overrides: CrossValidator.validate |

  


| Trees | Indices | Help | | MScanner | | --- | |
| --- | --- | --- | --- | --- |

|  |  |
| --- | --- |
| Generated by Epydoc 3.0beta1 on Fri Nov 23 09:13:21 2007 | http://epydoc.sourceforge.net |
