# Supplementary material for: MScanner: a classifier for retrieving Medline citations
Source: BMC Bioinformatics. 2008 Feb 19;9:108. doi: 10.1186/1471-2105-9-108 (PMC2263023; doi:10.1186/1471-2105-9-108)
Supplement: Additional file 3 — Source code for MScanner. mscanner-20071123.zip is a ZIP archive containing the Python 2.5 source code for MScanner, licensed under the GNU General Public License. It also contains API documentation in HTML format. Updated versions will be made available at . [file 1471-2105-9-108-S3.zip › mscanner/help/api/mscanner.core.Validator.Validator-class.html]

xml version="1.0" encoding="ascii"?


mscanner.core.Validator.Validator


| Trees | Indices | Help | | MScanner | | --- | |
| --- | --- | --- | --- | --- |

|  |  |  |  |
| --- | --- | --- | --- |
| Package mscanner :: Package core :: Module Validator :: Class Validator | |  | | --- | | [hide private] | | [frames] | no frames] | |

# Class Validator

source code  
  
Cross-validated calculation of article scores.  
  


|  |  |  |  |
| --- | --- | --- | --- |
| |  |  | | --- | --- | | Instance Methods | [hide private] | | |
|  | |  |  | | --- | --- | | \_\_init\_\_(self, featdb, featinfo, positives, negatives, nfolds=10)  Constructor parameters set corresponding instance attributes. | source code | |
|  | |  |  | | --- | --- | | validate(self)  Carry out validation. | source code | |
|  | |  |  | | --- | --- | | nfold\_validate(self, randomise=True)  Perform n-fold validation and return the raw performance measures | source code | |
|  | |  |  | | --- | --- | | leaveout\_validate(self)  Performs leave-out-one validation, returning the resulting scores. | source code | |


|  |  |  |  |
| --- | --- | --- | --- |
| |  |  | | --- | --- | | Static Methods | [hide private] | | |
|  | |  |  | | --- | --- | | make\_partitions(nitems, nparts)  Calculate partitions of input data for cross validation | source code | |


|  |  |  |  |
| --- | --- | --- | --- |
| |  |  | | --- | --- | | Instance Variables | [hide private] | | |
| Constructor Parameters | |
|  | featdb  Mapping from doc id to list of feature ids |
|  | featinfo  FeatureScores instance for stuff about features |
|  | negatives  Array of negative PMIDs for validation |
|  | nfolds  Number of validation folds (0 for leave-out-one) |
|  | positives  Array of positive PMIDs for validation |
| From validate | |
|  | nscores  Scores of negative articles after validation |
|  | pscores  Scores of positive articles after validation |


|  |  |  |  |
| --- | --- | --- | --- |
| |  |  | | --- | --- | | Method Details | [hide private] | | |

|  |  |  |
| --- | --- | --- |
| |  |  | | --- | --- | | validate(self) | source code |   Carry out validation. When nfolds==0 we use leave-out-one instead of k-fold. Returns:  pscores, nscores |

|  |  |  |
| --- | --- | --- |
| |  |  | | --- | --- | | make\_partitions(nitems, nparts)  *Static Method* | source code |  Calculate partitions of input data for cross validation Parameters:  - **`nitems`** - Number of items to partition - **`nparts`** - Number of partitions  Returns:  List of start indeces, and list of lengths for partitions |

|  |  |  |
| --- | --- | --- |
| |  |  | | --- | --- | | nfold\_validate(self, randomise=True) | source code |  Perform n-fold validation and return the raw performance measures Parameters:  - **`randomise`** - Randomise validation splits (use False for debugging)  Returns:  pscores, nscores |

|  |  |  |
| --- | --- | --- |
| |  |  | | --- | --- | | leaveout\_validate(self) | source code |  Performs leave-out-one validation, returning the resulting scores. Returns:  pscores, nscores  **Note:** Feature scores use background pseudocount - no other methods implemented.  **Deprecated:** 10-fold is standard, and leave-out-one is rather slow. |

  


| Trees | Indices | Help | | MScanner | | --- | |
| --- | --- | --- | --- | --- |

|  |  |
| --- | --- |
| Generated by Epydoc 3.0beta1 on Fri Oct 26 15:56:43 2007 | http://epydoc.sourceforge.net |
