# Supplementary material for: MScanner: a classifier for retrieving Medline citations
Source: BMC Bioinformatics. 2008 Feb 19;9:108. doi: 10.1186/1471-2105-9-108 (PMC2263023; doi:10.1186/1471-2105-9-108)
Supplement: Additional file 3 — Source code for MScanner. mscanner-20071123.zip is a ZIP archive containing the Python 2.5 source code for MScanner, licensed under the GNU General Public License. It also contains API documentation in HTML format. Updated versions will be made available at . [file 1471-2105-9-108-S3.zip › mscanner/help/api/mscanner.fastscores-module.html]

xml version="1.0" encoding="ascii"?


mscanner.fastscores


| Trees | Indices | Help | | MScanner | | --- | |
| --- | --- | --- | --- | --- |

|  |  |  |  |
| --- | --- | --- | --- |
| Package mscanner :: Package fastscores | |  | | --- | | [hide private] | | [frames] | no frames] | |

# Package fastscores

source code  
  
Utilities for rapidly calculating article scores and feature counts
across a subset of Medline.  
  


|  |  |  |  |
| --- | --- | --- | --- |
| |  |  | | --- | --- | | Submodules | [hide private] | | |
| - **mscanner.fastscores.FeatureCounter**: *Calculates the number of occurrences of each feature for all   articles in Medline between two dates.* - **mscanner.fastscores.ScoreCalculator**: *Calculates citation scores* |

  


| Trees | Indices | Help | | MScanner | | --- | |
| --- | --- | --- | --- | --- |

|  |  |
| --- | --- |
| Generated by Epydoc 3.0beta1 on Fri Nov 23 09:13:20 2007 | http://epydoc.sourceforge.net |
