# Supplementary material for: MScanner: a classifier for retrieving Medline citations
Source: BMC Bioinformatics. 2008 Feb 19;9:108. doi: 10.1186/1471-2105-9-108 (PMC2263023; doi:10.1186/1471-2105-9-108)
Supplement: Additional file 3 — Source code for MScanner. mscanner-20071123.zip is a ZIP archive containing the Python 2.5 source code for MScanner, licensed under the GNU General Public License. It also contains API documentation in HTML format. Updated versions will be made available at . [file 1471-2105-9-108-S3.zip › mscanner/help/api/mscanner.fastscores.FeatureCounter-pysrc.html]

xml version="1.0" encoding="ascii"?


mscanner.fastscores.FeatureCounter


| Trees | Indices | Help | | MScanner | | --- | |
| --- | --- | --- | --- | --- |

|  |  |  |  |
| --- | --- | --- | --- |
| Package mscanner :: Package fastscores :: Module FeatureCounter | |  | | --- | | [hide private] | | [frames] | no frames] | |

# Source Code for Module mscanner.fastscores.FeatureCounter

```
 1  """Calculates the number of occurrences of each feature for 
 2  all articles in Medline between two dates.""" 
 3   
 4  from __future__ import division 
 5  import numpy as nx 
 6  from path import path 
 7  import struct 
 8   
 9  from mscanner import update 
10  from mscanner.medline.FeatureStream import FeatureStream 
11   
12   


13 -class FeatureCounter:


14      """Class for calculating feature counts in a subset of Medline. 
15   
16      @ivar docstream: Path to file containing feature vectors for documents to 
17      score, in L{mscanner.medline.FeatureStream.FeatureStream} format. 
18       
19      @ivar numdocs: Number of documents in the stream of feature vectors. 
20       
21      @ivar numfeats: Number of distinct features in Medline (length of the  
22      vector of feature counts). 
23   
24      @ivar mindate: YYYYMMDD integer: documents must have this date or later 
25      (default 11110101) 
26       
27      @ivar maxdate: YYYYMMDD integer: documents must have this date or earlier 
28      (default 33330303) 
29   
30      @ivar exclude: PMIDs that are not allowed to appear in the results     
31      """ 
32       
33      counter_path = path(__file__).dirname() / "_FeatureCounter" 
34      """Executable file for counting features in a file""" 
35       


36 -    def __init__(self, 
37                   docstream, 
38                   numdocs, 
39                   numfeats, 
40                   mindate=None, 
41                   maxdate=None, 
42                   exclude=set(), 
43                   ):


44          if mindate is None: mindate = 10110101 
45          if maxdate is None: maxdate = 30330303 
46          update(self, locals())

47   
48   


49 -    def py_counts(s):


50          """Simply iterate over the documents and count how 
51          many times each feature occurs in the specified range 
52           
53          @return: Number of documents counted, and vector of feature counts.""" 
54          featcounts = nx.zeros(s.numfeats, nx.int32) 
55          docs = FeatureStream(open(s.docstream, "rb")) 
56          ndocs = 0 
57          try: 
58              for docid, date, features in docs: 
59                  if (date >= s.mindate and date <= s.maxdate  
60                      and docid not in s.exclude): 
61                      featcounts[features] += 1 
62                      ndocs += 1 
63          finally: 
64              docs.close() 
65          return ndocs, featcounts

66   
67   


68 -    def c_counts(s):


69          """Pipes parameters to a C program that parses the stream 
70          of documents with features, which counts the number 
71          of occurrences of each feature, only considering documents 
72          added to Medline in the specified date range. 
73           
74          @return: Number of documents counted, and vector of feature counts.""" 
75          import subprocess as sp 
76          p = sp.Popen([ 
77              s.counter_path,  
78              s.docstream, 
79              str(s.numdocs), 
80              str(s.numfeats), 
81              str(s.mindate), 
82              str(s.maxdate), 
83              str(len(s.exclude)), 
84              ], stdout=sp.PIPE, stdin=sp.PIPE) 
85          p.stdin.write(nx.array(sorted(s.exclude))) 
86          # First integer of output is the number of documents parsed 
87          ndocs = struct.unpack("I", p.stdout.read(4))[0] 
88          # Then a vector of feature counts 
89          featcounts = nx.fromfile(p.stdout, nx.int32, s.numfeats) 
90          return ndocs, featcounts

91
```

  


| Trees | Indices | Help | | MScanner | | --- | |
| --- | --- | --- | --- | --- |

|  |  |
| --- | --- |
| Generated by Epydoc 3.0beta1 on Fri Nov 23 09:13:25 2007 | http://epydoc.sourceforge.net |
