# Supplementary material for: MScanner: a classifier for retrieving Medline citations
Source: BMC Bioinformatics. 2008 Feb 19;9:108. doi: 10.1186/1471-2105-9-108 (PMC2263023; doi:10.1186/1471-2105-9-108)
Supplement: Additional file 3 — Source code for MScanner. mscanner-20071123.zip is a ZIP archive containing the Python 2.5 source code for MScanner, licensed under the GNU General Public License. It also contains API documentation in HTML format. Updated versions will be made available at . [file 1471-2105-9-108-S3.zip › mscanner/help/api/mscanner.fastscores.FeatureCounter.FeatureCounter-class.html]

xml version="1.0" encoding="ascii"?


mscanner.fastscores.FeatureCounter.FeatureCounter


| Trees | Indices | Help | | MScanner | | --- | |
| --- | --- | --- | --- | --- |

|  |  |  |  |
| --- | --- | --- | --- |
| Package mscanner :: Package fastscores :: Module FeatureCounter :: Class FeatureCounter | |  | | --- | | [hide private] | | [frames] | no frames] | |

# Class FeatureCounter

source code  
  
Class for calculating feature counts in a subset of Medline.  
  


|  |  |  |  |
| --- | --- | --- | --- |
| |  |  | | --- | --- | | Instance Methods | [hide private] | | |
|  | |  |  | | --- | --- | | \_\_init\_\_(self, docstream, numdocs, numfeats, mindate=None, maxdate=None, exclude=`set([``])`) | source code | |
|  | |  |  | | --- | --- | | py\_counts(s)  Simply iterate over the documents and count how many times each feature occurs in the specified range | source code | |
|  | |  |  | | --- | --- | | c\_counts(s)  Pipes parameters to a C program that parses the stream of documents with features, which counts the number of occurrences of each feature, only considering documents added to Medline in the specified date range. | source code | |


|  |  |  |  |
| --- | --- | --- | --- |
| |  |  | | --- | --- | | Class Variables | [hide private] | | |
|  | counter\_path = `path(u'C:\\Documents and Settings\\Graham\\My D...`  Executable file for counting features in a file |


|  |  |  |  |
| --- | --- | --- | --- |
| |  |  | | --- | --- | | Instance Variables | [hide private] | | |
|  | docstream  Path to file containing feature vectors for documents to score, in mscanner.medline.FeatureStream.FeatureStream format. |
|  | exclude  PMIDs that are not allowed to appear in the results |
|  | maxdate  YYYYMMDD integer: documents must have this date or earlier (default 33330303) |
|  | mindate  YYYYMMDD integer: documents must have this date or later (default 11110101) |
|  | numdocs  Number of documents in the stream of feature vectors. |
|  | numfeats  Number of distinct features in Medline (length of the vector of feature counts). |


|  |  |  |  |
| --- | --- | --- | --- |
| |  |  | | --- | --- | | Method Details | [hide private] | | |

|  |  |  |
| --- | --- | --- |
| |  |  | | --- | --- | | py\_counts(s) | source code |  Simply iterate over the documents and count how many times each feature occurs in the specified range Returns:  Number of documents counted, and vector of feature counts. |

|  |  |  |
| --- | --- | --- |
| |  |  | | --- | --- | | c\_counts(s) | source code |  Pipes parameters to a C program that parses the stream of documents with features, which counts the number of occurrences of each feature, only considering documents added to Medline in the specified date range. Returns:  Number of documents counted, and vector of feature counts. |

  


|  |  |  |  |
| --- | --- | --- | --- |
| |  |  | | --- | --- | | Class Variable Details | [hide private] | | |

|  |  |
| --- | --- |
| counter\_pathExecutable file for counting features in a file   Value:  |  | | --- | | ``` path(u'C:\\Documents and Settings\\Graham\\My Documents\\data\\MScanne r\\mscanner\\fastscores\\_FeatureCounter') ``` | |

  


| Trees | Indices | Help | | MScanner | | --- | |
| --- | --- | --- | --- | --- |

|  |  |
| --- | --- |
| Generated by Epydoc 3.0beta1 on Fri Nov 23 09:13:21 2007 | http://epydoc.sourceforge.net |
