# Supplementary material for: MScanner: a classifier for retrieving Medline citations
Source: BMC Bioinformatics. 2008 Feb 19;9:108. doi: 10.1186/1471-2105-9-108 (PMC2263023; doi:10.1186/1471-2105-9-108)
Supplement: Additional file 3 — Source code for MScanner. mscanner-20071123.zip is a ZIP archive containing the Python 2.5 source code for MScanner, licensed under the GNU General Public License. It also contains API documentation in HTML format. Updated versions will be made available at . [file 1471-2105-9-108-S3.zip › mscanner/help/api/mscanner.fastscores.ScoreCalculator-pysrc.html]

xml version="1.0" encoding="ascii"?


mscanner.fastscores.ScoreCalculator


| Trees | Indices | Help | | MScanner | | --- | |
| --- | --- | --- | --- | --- |

|  |  |  |  |
| --- | --- | --- | --- |
| Package mscanner :: Package fastscores :: Module ScoreCalculator | |  | | --- | | [hide private] | | [frames] | no frames] | |

# Source Code for Module mscanner.fastscores.ScoreCalculator

```
  1  """Calculates citation scores""" 
  2   
  3  from __future__ import division 
  4  import logging 
  5  import numpy as nx 
  6  from path import path 
  7   
  8  from mscanner import update 
  9  from mscanner.configuration import rc 
 10  from mscanner.medline.FeatureStream import FeatureStream 
 11   
 12   
 13  __copyright__ = "2007 Graham Poulter" 
 14  __author__ = "Graham Poulter <http://graham.poulter.googlepages.com>" 
 15  __license__ = """This program is free software: you can redistribute it and/or 
 16  modify it under the terms of the GNU General Public License as published by the 
 17  Free Software Foundation, either version 3 of the License, or (at your option) 
 18  any later version. 
 19   
 20  This program is distributed in the hope that it will be useful, but WITHOUT ANY 
 21  WARRANTY; without even the implied warranty of MERCHANTABILITY or FITNESS FOR A 
 22  PARTICULAR PURPOSE. See the GNU General Public License for more details. 
 23   
 24  You should have received a copy of the GNU General Public License along with 
 25  this program. If not, see <http://www.gnu.org/licenses/>.""" 
 26   
 27   


28 -class ScoreCalculator:


29      """Different methods for calculating the scores of all documents in the 
 30      database.  The idea is to pick between them based on speed, since 
 31      the faster ones may not be available on certain platforms. 
 32       
 33      @ivar docstream: Path to file containing feature vectors for documents to 
 34      score, in L{mscanner.medline.FeatureStream.FeatureStream} format. 
 35       
 36      @ivar numdocs: Number of documents in the stream of feature vectors. 
 37       
 38      @ivar featscores: Numpy array of double-precision feature scores. 
 39       
 40      @ivar offset: Sum of the Bayesian prior score and the base log likelihood  
 41      of an article with no features. 
 42   
 43      @ivar limit: Maximum number of results to return. 
 44       
 45      @ivar threshold: Cutoff score for including an article in the results 
 46       
 47      @ivar mindate: YYYYMMDD integer: documents must have this date or later 
 48      (default 11110101) 
 49       
 50      @ivar maxdate: YYYYMMDD integer: documents must have this date or earlier 
 51      (default 33330303) 
 52   
 53      @ivar exclude: Set of PMIDs that are not allowed to appear in the results 
 54      """ 
 55       
 56      executable_path = path(__file__).dirname() / "_ScoreCalculator" 
 57      """Path to executable for article score calculation""" 
 58       
 59      dll_path = path(__file__).dirname() / "_ScoreCalculator.dll" 
 60      """Path to shared library for article score calculation""" 
 61   


62 -    def __init__(self, 
 63                   docstream, 
 64                   numdocs, 
 65                   featscores, 
 66                   offset, 
 67                   limit, 
 68                   threshold=None, 
 69                   mindate=None, 
 70                   maxdate=None, 
 71                   exclude=set(), 
 72                   ):


73          # Callers may want to pass None, but the C code needs numbers. 
 74          if threshold is None: threshold = -10000.0 
 75          if mindate is None: mindate = 10110101 
 76          if maxdate is None: maxdate = 30330303 
 77          update(self, locals())

 78   
 79   


80 -    def score(s):


81          """Meta-method to top-scoring PubMed IDs in Medline 
 82           
 83          @note: All implementations iterate over the document stream and to find 
 84          articles that are between mindate and maxdate, are not members of 
 85          exclude, and have scores above the threshold. 
 86           
 87          @note: This method picks between L{cscore_dll}, L{cscore_pipe} and 
 88          L{pyscore} in decreasing order of preference (due to speed). 
 89           
 90          @return: List of (score, PMID) in decreasing order of score 
 91          """ 
 92          score = s.cscore_dll 
 93          if s.dll_path.isfile(): 
 94              try:  
 95                  import ctypes 
 96              except ImportError:  
 97                  score = s.cscore_pipe 
 98          else: 
 99              score = s.cscore_pipe 
100          if score == s.cscore_pipe and not s.executable_path.isfile(): 
101              score = s.pyscore 
102          return score()

103   
104   


105 -    def pyscore(s):


106          """Pure python implementation of L{score}""" 
107          logging.info("Performing query using ScoreCalculator.pyscore") 
108          results = [(-100000, 0)] * s.limit 
109          import heapq 
110          ndocs = 0 
111          logging.debug("Calculating article scores") 
112          marker = 0 
113          docs = FeatureStream(open(s.docstream, "rb")) 
114          try: 
115              for idx, (docid, date, features) in enumerate(docs): 
116                  if idx == marker: 
117                      logging.debug("Scored %d citations so far", idx) 
118                      marker += 100000 
119                  if (docid in s.exclude or date < s.mindate or date > s.maxdate): 
120                      continue 
121                  score = s.offset + nx.sum(s.featscores[features]) 
122                  if score >= s.threshold: 
123                      ndocs += 1 
124                      if score >= results[0][0]: 
125                          heapq.heapreplace(results, (score,docid)) 
126          finally: 
127              docs.close() 
128          if ndocs > s.limit: 
129              ndocs = limit 
130          return heapq.nlargest(ndocs, results)

131   
132   


133 -    def cscore_pipe(s):


134          """Calculate article scores by piping to the cscore program""" 
135          logging.info("Performing query using ScoreCalculator.cscore_pipe") 
136          import struct 
137          import subprocess as sp 
138          p = sp.Popen([ 
139              s.executable_path,  
140              s.docstream, 
141              str(s.numdocs), 
142              str(len(s.featscores)), 
143              str(s.offset), 
144              str(s.limit+len(s.exclude)), 
145              str(s.threshold), 
146              str(s.mindate), 
147              str(s.maxdate), 
148              ], stdout=sp.PIPE, stdin=sp.PIPE) 
149          p.stdin.write(s.featscores.tostring()) 
150          output = p.stdout.read(8) 
151          count = 0 
152          # Go through results in decreasing order to filter them 
153          result = [] 
154          while output != "": 
155              score, pmid = struct.unpack("fI", output) 
156              if pmid not in s.exclude: 
157                  result.append((score, pmid)) 
158                  count += 1 
159                  if count >= s.limit: 
160                      break 
161              output = p.stdout.read(8) 
162          p.stdout.close() 
163          return result

164   
165   


166 -    def cscore_dll(s):


167          """Calculate article scores, using ctypes to call cscores""" 
168          logging.info("Performing query using ScoreCalculator.cscore_dll") 
169          from ctypes import cdll, byref, c_int, c_void_p, c_char_p, c_float, c_double 
170          import itertools 
171          import numpy as nx 
172          # Set up arguments and call cscore2 function using ctypes 
173          carray = lambda dtype: nx.ctypeslib.ndpointer( 
174              dtype=dtype, ndim=1, flags='CONTIGUOUS') 
175          o_numresults = c_int() 
176          cscore = cdll.LoadLibrary(s.dll_path) 
177          cscore.cscore.argtypes = [  
178              c_char_p,           # docstream 
179              c_int,              # numdocs 
180              c_int,              # len(featscores) 
181              c_float,            # offset 
182              c_int,              # limit 
183              c_float,            # threshold 
184              c_int,              # mindate 
185              c_int,              # maxdate 
186              carray(nx.float64), # featscores 
187              c_void_p,           # o_numresults 
188              carray(nx.float32), # o_scores 
189              carray(nx.int32),   # o_pmids 
190          ] 
191          output_size = s.limit + len(s.exclude) # extra space for exclusions 
192          o_scores = nx.zeros(output_size, dtype=nx.float32) 
193          o_pmids = nx.zeros(output_size, dtype=nx.int32) 
194          # Now call this monstrously paramaterised function 
195          cscore.cscore( 
196              s.docstream, 
197              s.numdocs, 
198              len(s.featscores),  
199              s.offset, 
200              output_size, 
201              s.threshold, 
202              s.mindate, 
203              s.maxdate, 
204              s.featscores, 
205              byref(o_numresults), 
206              o_scores,  
207              o_pmids) 
208          # Go through results in decreasing order to filter them 
209          count_filtered = 0 
210          count_total = 0 
211          result = [] 
212          if o_numresults.value == 0: 
213              return result 
214          for score, pmid in itertools.izip(o_scores, o_pmids): 
215              if pmid not in s.exclude: 
216                  result.append((score, pmid)) 
217                  count_filtered += 1 
218                  if count_filtered >= s.limit: 
219                      break 
220              count_total += 1 
221              if count_total >= o_numresults.value: 
222                  break 
223          return result

224
```

  


| Trees | Indices | Help | | MScanner | | --- | |
| --- | --- | --- | --- | --- |

|  |  |
| --- | --- |
| Generated by Epydoc 3.0beta1 on Fri Nov 23 09:13:24 2007 | http://epydoc.sourceforge.net |
