# Supplementary material for: MScanner: a classifier for retrieving Medline citations
Source: BMC Bioinformatics. 2008 Feb 19;9:108. doi: 10.1186/1471-2105-9-108 (PMC2263023; doi:10.1186/1471-2105-9-108)
Supplement: Additional file 3 — Source code for MScanner. mscanner-20071123.zip is a ZIP archive containing the Python 2.5 source code for MScanner, licensed under the GNU General Public License. It also contains API documentation in HTML format. Updated versions will be made available at . [file 1471-2105-9-108-S3.zip › mscanner/help/api/mscanner.fastscores.ScoreCalculator.ScoreCalculator-class.html]

xml version="1.0" encoding="ascii"?


mscanner.fastscores.ScoreCalculator.ScoreCalculator


| Trees | Indices | Help | | MScanner | | --- | |
| --- | --- | --- | --- | --- |

|  |  |  |  |
| --- | --- | --- | --- |
| Package mscanner :: Package fastscores :: Module ScoreCalculator :: Class ScoreCalculator | |  | | --- | | [hide private] | | [frames] | no frames] | |

# Class ScoreCalculator

source code  
  
Different methods for calculating the scores of all documents in the
database. The idea is to pick between them based on speed, since the
faster ones may not be available on certain platforms.  
  


|  |  |  |  |
| --- | --- | --- | --- |
| |  |  | | --- | --- | | Instance Methods | [hide private] | | |
|  | |  |  | | --- | --- | | \_\_init\_\_(self, docstream, numdocs, featscores, offset, limit, threshold=None, mindate=None, maxdate=None, exclude=`set([``])`) | source code | |
|  | |  |  | | --- | --- | | score(s)  Meta-method to top-scoring PubMed IDs in Medline | source code | |
|  | |  |  | | --- | --- | | pyscore(s)  Pure python implementation of score | source code | |
|  | |  |  | | --- | --- | | cscore\_pipe(s)  Calculate article scores by piping to the cscore program | source code | |
|  | |  |  | | --- | --- | | cscore\_dll(s)  Calculate article scores, using ctypes to call cscores | source code | |


|  |  |  |  |
| --- | --- | --- | --- |
| |  |  | | --- | --- | | Class Variables | [hide private] | | |
|  | executable\_path = `path(u'C:\\Documents and Settings\\Graham\\M...`  Path to executable for article score calculation |
|  | dll\_path = `path(u'C:\\Documents and Settings\\Graham\\My Docum...`  Path to shared library for article score calculation |


|  |  |  |  |
| --- | --- | --- | --- |
| |  |  | | --- | --- | | Instance Variables | [hide private] | | |
|  | docstream  Path to file containing feature vectors for documents to score, in mscanner.medline.FeatureStream.FeatureStream format. |
|  | exclude  Set of PMIDs that are not allowed to appear in the results |
|  | featscores  Numpy array of double-precision feature scores. |
|  | limit  Maximum number of results to return. |
|  | maxdate  YYYYMMDD integer: documents must have this date or earlier (default 33330303) |
|  | mindate  YYYYMMDD integer: documents must have this date or later (default 11110101) |
|  | numdocs  Number of documents in the stream of feature vectors. |
|  | offset  Sum of the Bayesian prior score and the base log likelihood of an article with no features. |
|  | threshold  Cutoff score for including an article in the results |


|  |  |  |  |
| --- | --- | --- | --- |
| |  |  | | --- | --- | | Method Details | [hide private] | | |

|  |  |  |
| --- | --- | --- |
| |  |  | | --- | --- | | score(s) | source code |  Meta-method to top-scoring PubMed IDs in Medline Returns:  List of (score, PMID) in decreasing order of score  **Notes:**  - All implementations iterate over the document stream and to find   articles that are between mindate and maxdate, are not members of   exclude, and have scores above the threshold. - This method picks between cscore\_dll, cscore\_pipe and pyscore in decreasing order of preference (due to   speed). |

  


|  |  |  |  |
| --- | --- | --- | --- |
| |  |  | | --- | --- | | Class Variable Details | [hide private] | | |

|  |  |
| --- | --- |
| executable\_pathPath to executable for article score calculation   Value:  |  | | --- | | ``` path(u'C:\\Documents and Settings\\Graham\\My Documents\\data\\MScanne r\\mscanner\\fastscores\\_ScoreCalculator') ``` | |

|  |  |
| --- | --- |
| dll\_pathPath to shared library for article score calculation   Value:  |  | | --- | | ``` path(u'C:\\Documents and Settings\\Graham\\My Documents\\data\\MScanne r\\mscanner\\fastscores\\_ScoreCalculator.dll') ``` | |

  


| Trees | Indices | Help | | MScanner | | --- | |
| --- | --- | --- | --- | --- |

|  |  |
| --- | --- |
| Generated by Epydoc 3.0beta1 on Fri Nov 23 09:13:21 2007 | http://epydoc.sourceforge.net |
