# Supplementary material for: MScanner: a classifier for retrieving Medline citations
Source: BMC Bioinformatics. 2008 Feb 19;9:108. doi: 10.1186/1471-2105-9-108 (PMC2263023; doi:10.1186/1471-2105-9-108)
Supplement: Additional file 3 — Source code for MScanner. mscanner-20071123.zip is a ZIP archive containing the Python 2.5 source code for MScanner, licensed under the GNU General Public License. It also contains API documentation in HTML format. Updated versions will be made available at . [file 1471-2105-9-108-S3.zip › mscanner/help/api/mscanner.htdocs.controller-pysrc.html]

xml version="1.0" encoding="ascii"?


mscanner.htdocs.controller


| Trees | Indices | Help | | MScanner | | --- | |
| --- | --- | --- | --- | --- |

|  |  |  |  |
| --- | --- | --- | --- |
| Package mscanner :: Package htdocs :: Module controller | |  | | --- | | [hide private] | | [frames] | no frames] | |

# Source Code for Module mscanner.htdocs.controller

```
 1  #!/export/home/medscan/local32/bin/python2.5 
 2   
 3  """Controller for the MScanner web interface  
 4   
 5  In this context, the view is the template code, and the model is the queue 
 6  programme.""" 
 7   
 8  __copyright__ = "2007 Graham Poulter" 
 9  __author__ = "Graham Poulter <http://graham.poulter.googlepages.com>" 
10  __license__ = """This program is free software: you can redistribute it and/or 
11  modify it under the terms of the GNU General Public License as published by the 
12  Free Software Foundation, either version 3 of the License, or (at your option) 
13  any later version. 
14   
15  This program is distributed in the hope that it will be useful, but WITHOUT ANY 
16  WARRANTY; without even the implied warranty of MERCHANTABILITY or FITNESS FOR A 
17  PARTICULAR PURPOSE. See the GNU General Public License for more details. 
18   
19  You should have received a copy of the GNU General Public License along with 
20  this program. If not, see <http://www.gnu.org/licenses/>.""" 
21   
22  import sys 
23  sys.path.insert(0,"/export/home/medscan") 
24  import web 
25   
26  from mscanner.htdocs import templates 
27   
28  # Set informative error handler 
29  web.webapi.internalerror = web.debugerror 
30   
31  # URLs for the application 
32  urls = ( 
33      '/', 'FrontPage', 
34      '/query', 'templates.query_logic.QueryPage', 
35      '/status', 'templates.status_logic.StatusPage', 
36      '/output', 'templates.output_logic.OutputPage', 
37      '/contact', 'templates.contact_logic.ContactPage', 
38  ) 
39  """Mapping between URLs and objects to process the requests""" 
40   


41 -class FrontPage:


42      """Front page of the site""" 


43 -    def GET(self):


44          """Return the front page for MScanner""" 
45          web.header('Content-Type', 'text/html; charset=utf-8')  
46          page = templates.front.front() 
47          print page

48   
49  if __name__ == "__main__": 
50      try: 
51          web.run(urls, globals()) 
52      except KeyboardInterrupt: 
53          pass 
54
```

  


| Trees | Indices | Help | | MScanner | | --- | |
| --- | --- | --- | --- | --- |

|  |  |
| --- | --- |
| Generated by Epydoc 3.0beta1 on Fri Nov 23 09:13:23 2007 | http://epydoc.sourceforge.net |
