# Supplementary material for: MScanner: a classifier for retrieving Medline citations
Source: BMC Bioinformatics. 2008 Feb 19;9:108. doi: 10.1186/1471-2105-9-108 (PMC2263023; doi:10.1186/1471-2105-9-108)
Supplement: Additional file 3 — Source code for MScanner. mscanner-20071123.zip is a ZIP archive containing the Python 2.5 source code for MScanner, licensed under the GNU General Public License. It also contains API documentation in HTML format. Updated versions will be made available at . [file 1471-2105-9-108-S3.zip › mscanner/help/api/mscanner.htdocs.forms-module.html]

xml version="1.0" encoding="ascii"?


mscanner.htdocs.forms


| Trees | Indices | Help | | MScanner | | --- | |
| --- | --- | --- | --- | --- |

|  |  |  |  |
| --- | --- | --- | --- |
| Package mscanner :: Package htdocs :: Module forms | |  | | --- | | [hide private] | | [frames] | no frames] | |

# Module forms

source code  
  
Programmatic form construction and validation  
  


---

**Note:**
Originally web.form (part of web.py by Aaron Swartz,
http://webpy.org). I (Graham Poulter) needed form validation, and
used web.form as a starting point. Virtually every line has been
modified, but the module architecture is due to Aaron.

Authors:
:   Aaron Swartz,
    Graham Poulter

**License:**
Public Domain (specified by Aaron Swartz)


|  |  |  |  |
| --- | --- | --- | --- |
| |  |  | | --- | --- | | Classes | [hide private] | | |
|  | Form  Programmatically construct a form |
|  | Input  Represents input widgets in the form |
|  | Textbox  Widget for a text input |
|  | Password  Widget for a password input |
|  | Checkbox  Widget for a checkbox input |
|  | Hidden  Widget for a hidden input |
|  | File  Widget for a file input |
|  | Button  Widget for a button. |
|  | Textarea  Widget for a <textarea> |
|  | Dropdown  Widget for <select> dropdown box |
|  | Radio  Widget for a set of radio buttons |
|  | Validator  Generic validator to pass to an Input or Form constructor. |
|  | RegexValidator  Tests that the value matches a particular regular expression |


|  |  |  |  |
| --- | --- | --- | --- |
| |  |  | | --- | --- | | Functions | [hide private] | | |
|  | |  |  | | --- | --- | | attrget(obj, attr, value=None)  Retrieve something either as dictionary key or instance attribute | source code | |
|  | |  |  | | --- | --- | | ischecked(value)  True if the Checkbox was pressed | source code | |
|  | |  |  | | --- | --- | | buttonpressed(value)  True if the Button was pressed | source code | |


|  |  |  |  |
| --- | --- | --- | --- |
| |  |  | | --- | --- | | Variables | [hide private] | | |
|  | notnull = `<mscanner.htdocs.forms.Validator instance at 0x014F1...`  Use to specify that the input should not be left empty |
|  | checkbox\_validator = `<mscanner.htdocs.forms.Validator instance...`  Use to be sure the checkbox has valid input |


|  |  |  |  |
| --- | --- | --- | --- |
| |  |  | | --- | --- | | Function Details | [hide private] | | |

|  |  |  |
| --- | --- | --- |
| |  |  | | --- | --- | | attrget(obj, attr, value=None) | source code |  Retrieve something either as dictionary key or instance attribute Parameters:  - **`obj`** - Thing to retrieve from - **`attr`** - Name of thing to retrieve - **`value`** - Default if attr is not found |

  


|  |  |  |  |
| --- | --- | --- | --- |
| |  |  | | --- | --- | | Variables Details | [hide private] | | |

|  |  |
| --- | --- |
| notnullUse to specify that the input should not be left empty   Value:  |  | | --- | | ``` Validator(bool, "Required") ``` | |

|  |  |
| --- | --- |
| checkbox\_validatorUse to be sure the checkbox has valid input   Value:  |  | | --- | | ``` Validator(lambda x: x== None or x== "on", "Bad checkbox") ``` | |

  


| Trees | Indices | Help | | MScanner | | --- | |
| --- | --- | --- | --- | --- |

|  |  |
| --- | --- |
| Generated by Epydoc 3.0beta1 on Fri Nov 23 09:13:20 2007 | http://epydoc.sourceforge.net |
