# Supplementary material for: MScanner: a classifier for retrieving Medline citations
Source: BMC Bioinformatics. 2008 Feb 19;9:108. doi: 10.1186/1471-2105-9-108 (PMC2263023; doi:10.1186/1471-2105-9-108)
Supplement: Additional file 3 — Source code for MScanner. mscanner-20071123.zip is a ZIP archive containing the Python 2.5 source code for MScanner, licensed under the GNU General Public License. It also contains API documentation in HTML format. Updated versions will be made available at . [file 1471-2105-9-108-S3.zip › mscanner/help/api/mscanner.htdocs.forms-pysrc.html]

xml version="1.0" encoding="ascii"?


mscanner.htdocs.forms


| Trees | Indices | Help | | MScanner | | --- | |
| --- | --- | --- | --- | --- |

|  |  |  |  |
| --- | --- | --- | --- |
| Package mscanner :: Package htdocs :: Module forms | |  | | --- | | [hide private] | | [frames] | no frames] | |

# Source Code for Module mscanner.htdocs.forms

```
  1  """Programmatic form construction and validation 
  2   
  3  @note: Originally web.form (part of web.py by Aaron Swartz, http://webpy.org). 
  4  I (Graham Poulter) needed form validation, and used web.form as a starting 
  5  point. Virtually every line has been modified, but the module architecture is 
  6  due to Aaron. 
  7   
  8  @author: Aaron Swartz  
  9  @author: Graham Poulter 
 10   
 11  @license: Public Domain (specified by Aaron Swartz) 
 12  """ 
 13   
 14  import copy 
 15  import re 
 16  import web 
 17  from web import utils, net 
 18   


19 -def attrget(obj, attr, value=None):


20      """Retrieve something either as dictionary key or instance attribute 
 21       
 22      @param obj: Thing to retrieve from 
 23      @param attr: Name of thing to retrieve 
 24      @param value: Default if attr is not found""" 
 25      if hasattr(obj, '__contains__') and attr in obj: return obj[attr] 
 26      if hasattr(obj, attr): return getattr(obj, attr) 
 27      return value

 28   


29 -class Form:


30      """Programmatically construct a form 
 31       
 32      @ivar inputs: List of input fields in the form 
 33      @ivar valid: True if the form is unfilled, or validly filled 
 34      @ivar note: Message about invalid stuff 
 35      @ivar validators: List of validators that operate on the whole form 
 36      """ 
 37       


38 -    def __init__(self, *inputs, **kw):


39          """Construct a form.  Positional parameters are the form inputs. 
 40           
 41          @param inputs: List of input fields in the form 
 42           
 43          @keyword validators: Optional keyword, providing, a list of additional 
 44          validators on the form besides the ones associated with an input.""" 
 45          self.inputs = inputs 
 46          self.valid = True 
 47          self.note = None 
 48          self.validators = kw.pop('validators', [])

 49   
 50   


51 -    def __call__(self, inputs=None):


52          """Call the original instance to create copies to fill. 
 53           
 54          @param inputs: Optional a storage object which fills the form.""" 
 55          newform = copy.deepcopy(self) 
 56          if inputs:  
 57              newform.validates(inputs) 
 58          return newform

 59   
 60   


61 -    def render(self):


62          """An HTML table rendering of the form inputs""" 
 63          e_row = '<tr class="error"><td colspan="2">%s</td></tr>\n' 
 64          i_row = '<tr class="input"><th>%s</th><td class="value">%s %s %s</td></tr>\n' 
 65          # Render non-hidden inputs 
 66          rows = [] 
 67          if self.note:  
 68              rows.append(e_row % self.note) 
 69          for i in self.inputs: 
 70              if not isinstance(i, Hidden): 
 71                  rows.append(i_row % (i.renderlabel(), i.pre, i.render(), i.post)) 
 72                  if i.note:  
 73                      rows.append(e_row % i.note) 
 74          out = "\n".join(['<table class="form">'] + rows + ["</table>"]) 
 75          # Now render the hidden inputs 
 76          out += "\n".join( 
 77              ["<div>"] +  
 78              [i.render() for i in self.inputs if isinstance(i, Hidden)] +  
 79              ["</div>"]) 
 80          return out

 81   
 82   


83 -    def render_errors(self):


84          errors = ["<li>%s: %s</li>" % (n,e) for n,e in  
 85                   self.errors.iteritems() if e is not None] 
 86          return "\n".join(["<p>Errors</p><ul>"] + errors + ["</ul>"])

 87       
 88       


89 -    def validates(self, source, _validate=True):


90          """Validate the form, also filling its values 
 91           
 92          @param source: Storage object from which to set form values 
 93           
 94          @returns: True/False about whether the form validates.""" 
 95          if hasattr(self, "_d"):  
 96              del self._d # Refresh the data property 
 97          isvalid = True 
 98          for i in self.inputs: 
 99              value = attrget(source, i.name) 
100              if _validate: 
101                  isvalid = i.validate(value) and isvalid 
102              else: 
103                  i.value = value 
104          if _validate: 
105              isvalid = self._validate(source) and isvalid 
106          self.valid = isvalid 
107          return isvalid

108   
109   


110 -    def _validate(self, source):


111          """Run additional validators for the form 
112           
113          @param source: Storage object containing form values         
114          """ 
115          for v in self.validators: 
116              if not v.valid(source): 
117                  self.note = v.msg 
118                  return False 
119          return True

120   
121   


122 -    def fill(self, source=None):


123          """Fill the form without validating 
124   
125          @param source: Storage object from which to set form values 
126          """ 
127          self.validates(source, _validate=False)

128       
129       


130 -    def __getitem__(self, key):


131          """Dictionary access to inputs. 
132           
133          @param key: Name of input to retrieve""" 
134          for x in self.inputs: 
135              if x.name == key: return x 
136          raise KeyError, key

137       
138       
139      @property 


140 -    def d(self):


141          """A storage dictionary of the form inputs (deleted by validates())""" 
142          try: 
143              return self._d 
144          except AttributeError: 
145              self._d = utils.storage([(i.name, i.value) for i in self.inputs]) 
146              return self._d

147       
148       
149      @property 


150 -    def errors(self):


151          """A storage dictionary of the form errors.""" 
152          return utils.storage([(i.name, i.note) for i in self.inputs])

153   
154   
155   


156 -class Input(object):


157      """Represents input widgets in the form 
158   
159      @ivar name: The name= attribute for the input 
160      @ivar validators: List of Validator to apply to the input 
161      @ivar label: Contents of the <label> for the input 
162      @ivar pre: Text before the input 
163      @ivar post: Text after the input 
164      @ivar id: For id= attribute (but defaults to name if not provided) 
165      @ivar attrs: Other attributes 
166   
167      @ivar note: Message set by the first validator that fails 
168      """ 
169       


170 -    def __init__(self, name, *validators, **attrs):


171          """Constructor - parameters correspond to instance variables. 
172           
173          @keyword class_: Specifies the class= attribute. 
174          """ 
175          self.name = name 
176          self.note = None 
177          self.validators = validators 
178          self.label = attrs.pop('label', name) 
179          self.value = attrs.pop('value', None) 
180          self.pre = attrs.pop('pre', "") 
181          self.post = attrs.pop('post', "") 
182          self.id = attrs.setdefault("id", name) 
183          if 'class_' in attrs:  
184              attrs['class'] = attrs['class_'] 
185              del attrs['class_'] 
186          self.attrs = attrs

187   
188   


189 -    def validate(self, value):


190          """Validate the input 
191           
192          @param value: Value to fill the input  
193           
194          @return: True if all validators work, otherwise False and sets the note 
195          to the validator message""" 
196          self.value = value 
197          for v in self.validators: 
198              if not v.valid(value): 
199                  self.note = v.msg 
200                  return False 
201          return True

202   
203   


204 -    def render(self):


205          """Render the <input> element itself""" 
206          raise NotImplementedError

207       
208       


209 -    def renderlabel(self):


210          """Render the label for the input""" 
211          return '<label for="%s">%s</label>' % (self.id, self.label)

212   
213   


214 -    def addatts(self):


215          """Render additional attributes within a tag""" 
216          str = "" 
217          for (n, v) in self.attrs.items(): 
218              str += ' %s="%s"' % (n, net.websafe(v)) 
219          return str

220       
221       
222       


223 -class Textbox(Input):


224      """Widget for a text input""" 
225       


226 -    def render(self):


227          value = ' value="%s"' % net.websafe(self.value) if self.value else "" 
228          return '<input type="text" name="%s"%s%s>' % ( 
229              net.websafe(self.name), value, self.addatts())

230   
231   
232       


233 -class Password(Input):


234      """Widget for a password input""" 
235       


236 -    def render(self):


237          value = ' value="%s"' % net.websafe(self.value) if self.value else "" 
238          return '<input type="password" name="%s"%s%s>' % ( 
239              net.websafe(self.name), value, self.addatts())

240   
241   
242   


243 -class Checkbox(Input):


244      """Widget for a checkbox input""" 
245       


246 -    def render(self):


247          checked = ' checked="checked"' if self.value else '' 
248          return '<input type="checkbox" name="%s"%s%s>' % ( 
249              net.websafe(self.name), checked, self.addatts())

250       
251   
252   


253 -class Hidden(Input):


254      """Widget for a hidden input""" 
255   


256 -    def render(self):


257          value = ' value="%s"' % net.websafe(self.value) if self.value else "" 
258          return '<input type="hidden" name="%s"%s>' % ( 
259              net.websafe(self.name), value)

260   
261   
262   


263 -class File(Input):


264      """Widget for a file input""" 
265       


266 -    def render(self):


267          value = ' value="%s"' % net.websafe(self.value) if self.value else "" 
268          return '<input type="file" name="%s"%s%s>' % ( 
269              net.websafe(self.name), value, self.addatts())

270       
271       
272       


273 -class Button(Input):


274      """Widget for a button. 
275       
276      @note: The form was submitted by pressing this button if the buttons' name 
277      is in web.inputs (with empty string for value) 
278      """ 
279       


280 -    def render(self):


281          x = '<button name="%s"%s>%s</button>' % ( 
282              self.name, self.addatts(), self.label) 
283          return x

284   
285   
286   


287 -class Textarea(Input):


288      """Widget for a <textarea>""" 
289       


290 -    def render(self):


291          value = net.websafe(self.value) if self.value else "" 
292          return '<textarea name="%s"%s>%s</textarea>' % ( 
293              net.websafe(self.name), self.addatts(), value)

294   
295   
296   


297 -class Dropdown(Input):


298      """Widget for <select> dropdown box""" 
299       


300 -    def __init__(self, name, args, *validators, **attrs):


301          """Constructor 
302           
303          @param args: List of values or (value,description) pairs for the 
304          dropdown box.""" 
305          self.args = args 
306          super(Dropdown, self).__init__(name, *validators, **attrs)

307   
308   


309 -    def render(self):


310          x = '<select name="%s"%s>\n' % ( 
311              net.websafe(self.name), self.addatts()) 
312          for arg in self.args: 
313              if type(arg) == tuple: 
314                  value, desc = arg 
315              else: 
316                  value, desc = arg, arg  
317              if self.value == value:  
318                  select_p = ' selected="selected"' 
319              else:  
320                  select_p = '' 
321              x += '<option%s value="%s">%s</option>\n' % ( 
322                  select_p, net.websafe(value), net.websafe(desc)) 
323          x += '</select>\n' 
324          return x

325   
326   
327   


328 -class Radio(Input):


329      """Widget for a set of radio buttons""" 
330       


331 -    def __init__(self, name, args, *validators, **attrs):


332          """Constructor 
333           
334          @param args: List of values or (value,description) pairs for radio 
335          buttons.""" 
336          self.args = args 
337          super(Radio, self).__init__(name, *validators, **attrs)

338   
339   


340 -    def renderlabel(self):


341          """Plain-text label: no unique ID for set of buttons""" 
342          return self.label

343   
344   


345 -    def render(self, only=None):


346          """Write a list of radio inputs.  
347           
348          @param only: Render just the radio button whose name matches.""" 
349          out = "" 
350          for arg in self.args: 
351              if type(arg) == tuple: 
352                  value, desc = arg 
353              else: 
354                  value, desc = arg, arg  
355              if only is not None and value != only: 
356                  continue 
357              if self.value == value:  
358                  select_p = ' checked="checked"' 
359              else:  
360                  select_p = '' 
361              out += '<span><input type="radio" name="%s" value="%s"%s> %s </span>' % \ 
362              (net.websafe(self.name), net.websafe(value), select_p, net.websafe(desc)) 
363          return out

364   
365   
366   


367 -class Validator:


368      """Generic validator to pass to an Input or Form constructor.""" 
369   


370 -    def __init__(self, test, msg):

371          """Constructor 
372           
373          @param test: Applied to input value when used as an input validator, 
374          and applied to the Storage source for the form when used as a form 
375          validator. 
376           
377          @param msg: To be assigned to the note when validator fails. 
378          """ 
379          utils.autoassign(self, locals())

380           


381 -    def __deepcopy__(self, memo):

382          return copy.copy(self) 
383       


384 -    def valid(self, value):


385          """Returns true if the test function succeeds""" 
386          try:  
387              return self.test(value) 
388          except:  
389              return False

390   
391   
392   


393 -class RegexValidator(Validator):


394      """Tests that the value matches a particular regular expression""" 
395   


396 -    def __init__(self, rexp, msg):


397          """Constructor 
398           
399          @param rexp: String containing the regular expression 
400          """ 
401          self.rexp = re.compile(rexp) 
402          self.msg = msg

403       


404 -    def valid(self, value):


405          return bool(self.rexp.match(value))

406   
407   
408   
409  notnull = Validator(bool, "Required") 
410  """Use to specify that the input should not be left empty""" 
411   
412   
413  checkbox_validator = Validator( 
414      lambda x: x == None or x == "on", "Bad checkbox") 
415  """Use to be sure the checkbox has valid input""" 
416   
417   


418 -def ischecked(value):


419      """True if the Checkbox was pressed""" 
420      return True if value == "on" else bool(value)

421   
422   


423 -def buttonpressed(value):


424      """True if the Button was pressed""" 
425      return True if value == "" else bool(value)

426
```

  


| Trees | Indices | Help | | MScanner | | --- | |
| --- | --- | --- | --- | --- |

|  |  |
| --- | --- |
| Generated by Epydoc 3.0beta1 on Fri Nov 23 09:13:25 2007 | http://epydoc.sourceforge.net |
