# Supplementary material for: MScanner: a classifier for retrieving Medline citations
Source: BMC Bioinformatics. 2008 Feb 19;9:108. doi: 10.1186/1471-2105-9-108 (PMC2263023; doi:10.1186/1471-2105-9-108)
Supplement: Additional file 3 — Source code for MScanner. mscanner-20071123.zip is a ZIP archive containing the Python 2.5 source code for MScanner, licensed under the GNU General Public License. It also contains API documentation in HTML format. Updated versions will be made available at . [file 1471-2105-9-108-S3.zip › mscanner/help/api/mscanner.htdocs.forms.Form-class.html]

xml version="1.0" encoding="ascii"?


mscanner.htdocs.forms.Form


| Trees | Indices | Help | | MScanner | | --- | |
| --- | --- | --- | --- | --- |

|  |  |  |  |
| --- | --- | --- | --- |
| Package mscanner :: Package htdocs :: Module forms :: Class Form | |  | | --- | | [hide private] | | [frames] | no frames] | |

# Class Form

source code  
  
Programmatically construct a form  
  


|  |  |  |  |
| --- | --- | --- | --- |
| |  |  | | --- | --- | | Instance Methods | [hide private] | | |
|  | |  |  | | --- | --- | | \_\_init\_\_(self, \*inputs, \*\*kw)  Construct a form. | source code | |
|  | |  |  | | --- | --- | | \_\_call\_\_(self, inputs=None)  Call the original instance to create copies to fill. | source code | |
|  | |  |  | | --- | --- | | render(self)  An HTML table rendering of the form inputs | source code | |
|  | |  |  | | --- | --- | | render\_errors(self) | source code | |
|  | |  |  | | --- | --- | | validates(self, source, \_validate=True)  Validate the form, also filling its values | source code | |
|  | |  |  | | --- | --- | | \_validate(self, source)  Run additional validators for the form | source code | |
|  | |  |  | | --- | --- | | fill(self, source=None)  Fill the form without validating | source code | |
|  | |  |  | | --- | --- | | \_\_getitem\_\_(self, key)  Dictionary access to inputs. | source code | |


|  |  |  |  |
| --- | --- | --- | --- |
| |  |  | | --- | --- | | Instance Variables | [hide private] | | |
|  | inputs  List of input fields in the form |
|  | note  Message about invalid stuff |
|  | valid  True if the form is unfilled, or validly filled |
|  | validators  List of validators that operate on the whole form |


|  |  |  |  |
| --- | --- | --- | --- |
| |  |  | | --- | --- | | Properties | [hide private] | | |
|  | d  A storage dictionary of the form inputs (deleted by validates()) |
|  | errors  A storage dictionary of the form errors. |


|  |  |  |  |
| --- | --- | --- | --- |
| |  |  | | --- | --- | | Method Details | [hide private] | | |

|  |  |  |
| --- | --- | --- |
| |  |  | | --- | --- | | \_\_init\_\_(self, \*inputs, \*\*kw)  *(Constructor)* | source code |  Construct a form. Positional parameters are the form inputs. Parameters:  - **`inputs`** - List of input fields in the form - **`validators`** - Optional keyword, providing, a list of additional validators   on the form besides the ones associated with an input. |

|  |  |  |
| --- | --- | --- |
| |  |  | | --- | --- | | \_\_call\_\_(self, inputs=None)  *(Call operator)* | source code |  Call the original instance to create copies to fill. Parameters:  - **`inputs`** - Optional a storage object which fills the form. |

|  |  |  |
| --- | --- | --- |
| |  |  | | --- | --- | | validates(self, source, \_validate=True) | source code |  Validate the form, also filling its values Parameters:  - **`source`** - Storage object from which to set form values  Returns:  True/False about whether the form validates. |

|  |  |  |
| --- | --- | --- |
| |  |  | | --- | --- | | \_validate(self, source) | source code |  Run additional validators for the form Parameters:  - **`source`** - Storage object containing form values |

|  |  |  |
| --- | --- | --- |
| |  |  | | --- | --- | | fill(self, source=None) | source code |  Fill the form without validating Parameters:  - **`source`** - Storage object from which to set form values |

|  |  |  |
| --- | --- | --- |
| |  |  | | --- | --- | | \_\_getitem\_\_(self, key)  *(Indexing operator)* | source code |  Dictionary access to inputs. Parameters:  - **`key`** - Name of input to retrieve |

  


|  |  |  |  |
| --- | --- | --- | --- |
| |  |  | | --- | --- | | Property Details | [hide private] | | |

|  |
| --- |
| dA storage dictionary of the form inputs (deleted by validates()) Get Method:  *unreachable*.d(self) - A storage dictionary of the form inputs (deleted by validates()) |

|  |
| --- |
| errorsA storage dictionary of the form errors. Get Method:  *unreachable*.errors(self) - A storage dictionary of the form errors. |

  


| Trees | Indices | Help | | MScanner | | --- | |
| --- | --- | --- | --- | --- |

|  |  |
| --- | --- |
| Generated by Epydoc 3.0beta1 on Fri Nov 23 09:13:21 2007 | http://epydoc.sourceforge.net |
