# Supplementary material for: MScanner: a classifier for retrieving Medline citations
Source: BMC Bioinformatics. 2008 Feb 19;9:108. doi: 10.1186/1471-2105-9-108 (PMC2263023; doi:10.1186/1471-2105-9-108)
Supplement: Additional file 3 — Source code for MScanner. mscanner-20071123.zip is a ZIP archive containing the Python 2.5 source code for MScanner, licensed under the GNU General Public License. It also contains API documentation in HTML format. Updated versions will be made available at . [file 1471-2105-9-108-S3.zip › mscanner/help/api/mscanner.htdocs.forms.Input-class.html]

xml version="1.0" encoding="ascii"?


mscanner.htdocs.forms.Input


| Trees | Indices | Help | | MScanner | | --- | |
| --- | --- | --- | --- | --- |

|  |  |  |  |
| --- | --- | --- | --- |
| Package mscanner :: Package htdocs :: Module forms :: Class Input | |  | | --- | | [hide private] | | [frames] | no frames] | |

# Class Input

source code  
  

```
object --+
         |
        Input
```

Known Subclasses:
:   Button,
    Checkbox,
    Dropdown,
    File,
    Hidden,
    Password,
    Radio,
    Textarea,
    Textbox

---

Represents input widgets in the form  
  


|  |  |  |  |
| --- | --- | --- | --- |
| |  |  | | --- | --- | | Instance Methods | [hide private] | | |
|  | |  |  | | --- | --- | | \_\_init\_\_(self, name, \*validators, \*\*attrs)  Constructor - parameters correspond to instance variables. | source code | |
|  | |  |  | | --- | --- | | validate(self, value)  Validate the input | source code | |
|  | |  |  | | --- | --- | | render(self)  Render the <input> element itself | source code | |
|  | |  |  | | --- | --- | | renderlabel(self)  Render the label for the input | source code | |
|  | |  |  | | --- | --- | | addatts(self)  Render additional attributes within a tag | source code | |
| **Inherited from `object`**: `__delattr__`, `__getattribute__`, `__hash__`, `__new__`, `__reduce__`, `__reduce_ex__`, `__repr__`, `__setattr__`, `__str__` | |


|  |  |  |  |
| --- | --- | --- | --- |
| |  |  | | --- | --- | | Instance Variables | [hide private] | | |
|  | attrs  Other attributes |
|  | id  For id= attribute (but defaults to name if not provided) |
|  | label  Contents of the <label> for the input |
|  | name  The name= attribute for the input |
|  | note  Message set by the first validator that fails |
|  | post  Text after the input |
|  | pre  Text before the input |
|  | validators  List of Validator to apply to the input |


|  |  |  |  |
| --- | --- | --- | --- |
| |  |  | | --- | --- | | Properties | [hide private] | | |
| **Inherited from `object`**: `__class__` | |


|  |  |  |  |
| --- | --- | --- | --- |
| |  |  | | --- | --- | | Method Details | [hide private] | | |

|  |  |  |
| --- | --- | --- |
| |  |  | | --- | --- | | \_\_init\_\_(self, name, \*validators, \*\*attrs)  *(Constructor)* | source code |  Constructor - parameters correspond to instance variables. Parameters:  - **`class_`** - Specifies the class= attribute.  Overrides: object.\_\_init\_\_ |

|  |  |  |
| --- | --- | --- |
| |  |  | | --- | --- | | validate(self, value) | source code |  Validate the input Parameters:  - **`value`** - Value to fill the input  Returns:  True if all validators work, otherwise False and sets the note to the validator message |

  


| Trees | Indices | Help | | MScanner | | --- | |
| --- | --- | --- | --- | --- |

|  |  |
| --- | --- |
| Generated by Epydoc 3.0beta1 on Fri Nov 23 09:13:21 2007 | http://epydoc.sourceforge.net |
