# Supplementary material for: MScanner: a classifier for retrieving Medline citations
Source: BMC Bioinformatics. 2008 Feb 19;9:108. doi: 10.1186/1471-2105-9-108 (PMC2263023; doi:10.1186/1471-2105-9-108)
Supplement: Additional file 3 — Source code for MScanner. mscanner-20071123.zip is a ZIP archive containing the Python 2.5 source code for MScanner, licensed under the GNU General Public License. It also contains API documentation in HTML format. Updated versions will be made available at . [file 1471-2105-9-108-S3.zip › mscanner/help/api/mscanner.htdocs.forms.Radio-class.html]

xml version="1.0" encoding="ascii"?


mscanner.htdocs.forms.Radio


| Trees | Indices | Help | | MScanner | | --- | |
| --- | --- | --- | --- | --- |

|  |  |  |  |
| --- | --- | --- | --- |
| Package mscanner :: Package htdocs :: Module forms :: Class Radio | |  | | --- | | [hide private] | | [frames] | no frames] | |

# Class Radio

source code  
  

```
object --+    
         |    
     Input --+
             |
            Radio
```

---

Widget for a set of radio buttons  
  


|  |  |  |  |
| --- | --- | --- | --- |
| |  |  | | --- | --- | | Instance Methods | [hide private] | | |
|  | |  |  | | --- | --- | | \_\_init\_\_(self, name, args, \*validators, \*\*attrs)  Constructor | source code | |
|  | |  |  | | --- | --- | | renderlabel(self)  Plain-text label: no unique ID for set of buttons | source code | |
|  | |  |  | | --- | --- | | render(self, only=None)  Write a list of radio inputs. | source code | |
| **Inherited from `Input`**: `addatts`, `validate`  **Inherited from `object`**: `__delattr__`, `__getattribute__`, `__hash__`, `__new__`, `__reduce__`, `__reduce_ex__`, `__repr__`, `__setattr__`, `__str__` | |


|  |  |  |  |
| --- | --- | --- | --- |
| |  |  | | --- | --- | | Instance Variables | [hide private] | | |
| **Inherited from `Input`**: `attrs`, `id`, `label`, `name`, `note`, `post`, `pre`, `validators` | |


|  |  |  |  |
| --- | --- | --- | --- |
| |  |  | | --- | --- | | Properties | [hide private] | | |
| **Inherited from `object`**: `__class__` | |


|  |  |  |  |
| --- | --- | --- | --- |
| |  |  | | --- | --- | | Method Details | [hide private] | | |

|  |  |  |
| --- | --- | --- |
| |  |  | | --- | --- | | \_\_init\_\_(self, name, args, \*validators, \*\*attrs)  *(Constructor)* | source code |  Constructor Parameters:  - **`args`** - List of values or (value,description) pairs for radio   buttons.  Overrides: Input.\_\_init\_\_ |

|  |  |  |
| --- | --- | --- |
| |  |  | | --- | --- | | renderlabel(self) | source code |  Plain-text label: no unique ID for set of buttons Overrides: Input.renderlabel |

|  |  |  |
| --- | --- | --- |
| |  |  | | --- | --- | | render(self, only=None) | source code |  Write a list of radio inputs. Parameters:  - **`only`** - Render just the radio button whose name matches.  Overrides: Input.render |

  


| Trees | Indices | Help | | MScanner | | --- | |
| --- | --- | --- | --- | --- |

|  |  |
| --- | --- |
| Generated by Epydoc 3.0beta1 on Fri Nov 23 09:13:21 2007 | http://epydoc.sourceforge.net |
