# Supplementary material for: MScanner: a classifier for retrieving Medline citations
Source: BMC Bioinformatics. 2008 Feb 19;9:108. doi: 10.1186/1471-2105-9-108 (PMC2263023; doi:10.1186/1471-2105-9-108)
Supplement: Additional file 3 — Source code for MScanner. mscanner-20071123.zip is a ZIP archive containing the Python 2.5 source code for MScanner, licensed under the GNU General Public License. It also contains API documentation in HTML format. Updated versions will be made available at . [file 1471-2105-9-108-S3.zip › mscanner/help/api/mscanner.htdocs.forms.RegexValidator-class.html]

xml version="1.0" encoding="ascii"?


mscanner.htdocs.forms.RegexValidator


| Trees | Indices | Help | | MScanner | | --- | |
| --- | --- | --- | --- | --- |

|  |  |  |  |
| --- | --- | --- | --- |
| Package mscanner :: Package htdocs :: Module forms :: Class RegexValidator | |  | | --- | | [hide private] | | [frames] | no frames] | |

# Class RegexValidator

source code  
  

```
Validator --+
            |
           RegexValidator
```

---

Tests that the value matches a particular regular expression  
  


|  |  |  |  |
| --- | --- | --- | --- |
| |  |  | | --- | --- | | Instance Methods | [hide private] | | |
|  | |  |  | | --- | --- | | \_\_init\_\_(self, rexp, msg)  Constructor | source code | |
|  | |  |  | | --- | --- | | valid(self, value)  Returns true if the test function succeeds | source code | |
| **Inherited from `Validator`**: `__deepcopy__` | |


|  |  |  |  |
| --- | --- | --- | --- |
| |  |  | | --- | --- | | Method Details | [hide private] | | |

|  |  |  |
| --- | --- | --- |
| |  |  | | --- | --- | | \_\_init\_\_(self, rexp, msg)  *(Constructor)* | source code |  Constructor Parameters:  - **`rexp`** - String containing the regular expression  Overrides: Validator.\_\_init\_\_ |

|  |  |  |
| --- | --- | --- |
| |  |  | | --- | --- | | valid(self, value) | source code |  Returns true if the test function succeeds Overrides: Validator.valid *(inherited documentation)* |

  


| Trees | Indices | Help | | MScanner | | --- | |
| --- | --- | --- | --- | --- |

|  |  |
| --- | --- |
| Generated by Epydoc 3.0beta1 on Fri Nov 23 09:13:21 2007 | http://epydoc.sourceforge.net |
