# Supplementary material for: MScanner: a classifier for retrieving Medline citations
Source: BMC Bioinformatics. 2008 Feb 19;9:108. doi: 10.1186/1471-2105-9-108 (PMC2263023; doi:10.1186/1471-2105-9-108)
Supplement: Additional file 3 — Source code for MScanner. mscanner-20071123.zip is a ZIP archive containing the Python 2.5 source code for MScanner, licensed under the GNU General Public License. It also contains API documentation in HTML format. Updated versions will be made available at . [file 1471-2105-9-108-S3.zip › mscanner/help/api/mscanner.htdocs.forms.Textbox-class.html]

xml version="1.0" encoding="ascii"?


mscanner.htdocs.forms.Textbox


| Trees | Indices | Help | | MScanner | | --- | |
| --- | --- | --- | --- | --- |

|  |  |  |  |
| --- | --- | --- | --- |
| Package mscanner :: Package htdocs :: Module forms :: Class Textbox | |  | | --- | | [hide private] | | [frames] | no frames] | |

# Class Textbox

source code  
  

```
object --+    
         |    
     Input --+
             |
            Textbox
```

---

Widget for a text input  
  


|  |  |  |  |
| --- | --- | --- | --- |
| |  |  | | --- | --- | | Instance Methods | [hide private] | | |
|  | |  |  | | --- | --- | | render(self)  Render the <input> element itself | source code | |
| **Inherited from `Input`**: `__init__`, `addatts`, `renderlabel`, `validate`  **Inherited from `object`**: `__delattr__`, `__getattribute__`, `__hash__`, `__new__`, `__reduce__`, `__reduce_ex__`, `__repr__`, `__setattr__`, `__str__` | |


|  |  |  |  |
| --- | --- | --- | --- |
| |  |  | | --- | --- | | Instance Variables | [hide private] | | |
| **Inherited from `Input`**: `attrs`, `id`, `label`, `name`, `note`, `post`, `pre`, `validators` | |


|  |  |  |  |
| --- | --- | --- | --- |
| |  |  | | --- | --- | | Properties | [hide private] | | |
| **Inherited from `object`**: `__class__` | |


|  |  |  |  |
| --- | --- | --- | --- |
| |  |  | | --- | --- | | Method Details | [hide private] | | |

|  |  |  |
| --- | --- | --- |
| |  |  | | --- | --- | | render(self) | source code |  Render the <input> element itself Overrides: Input.render *(inherited documentation)* |

  


| Trees | Indices | Help | | MScanner | | --- | |
| --- | --- | --- | --- | --- |

|  |  |
| --- | --- |
| Generated by Epydoc 3.0beta1 on Fri Nov 23 09:13:21 2007 | http://epydoc.sourceforge.net |
