# Supplementary material for: MScanner: a classifier for retrieving Medline citations
Source: BMC Bioinformatics. 2008 Feb 19;9:108. doi: 10.1186/1471-2105-9-108 (PMC2263023; doi:10.1186/1471-2105-9-108)
Supplement: Additional file 3 — Source code for MScanner. mscanner-20071123.zip is a ZIP archive containing the Python 2.5 source code for MScanner, licensed under the GNU General Public License. It also contains API documentation in HTML format. Updated versions will be made available at . [file 1471-2105-9-108-S3.zip › mscanner/help/api/mscanner.htdocs.forms.Validator-class.html]

xml version="1.0" encoding="ascii"?


mscanner.htdocs.forms.Validator


| Trees | Indices | Help | | MScanner | | --- | |
| --- | --- | --- | --- | --- |

|  |  |  |  |
| --- | --- | --- | --- |
| Package mscanner :: Package htdocs :: Module forms :: Class Validator | |  | | --- | | [hide private] | | [frames] | no frames] | |

# Class Validator

source code  
  

Known Subclasses:
:   RegexValidator

---

Generic validator to pass to an Input or Form constructor.  
  


|  |  |  |  |
| --- | --- | --- | --- |
| |  |  | | --- | --- | | Instance Methods | [hide private] | | |
|  | |  |  | | --- | --- | | \_\_init\_\_(self, test, msg)  Constructor | source code | |
|  | |  |  | | --- | --- | | \_\_deepcopy\_\_(self, memo) | source code | |
|  | |  |  | | --- | --- | | valid(self, value)  Returns true if the test function succeeds | source code | |


|  |  |  |  |
| --- | --- | --- | --- |
| |  |  | | --- | --- | | Method Details | [hide private] | | |

|  |  |  |
| --- | --- | --- |
| |  |  | | --- | --- | | \_\_init\_\_(self, test, msg)  *(Constructor)* | source code |  Constructor Parameters:  - **`test`** - Applied to input value when used as an input validator, and   applied to the Storage source for the form when used as a form   validator. - **`msg`** - To be assigned to the note when validator fails. |

  


| Trees | Indices | Help | | MScanner | | --- | |
| --- | --- | --- | --- | --- |

|  |  |
| --- | --- |
| Generated by Epydoc 3.0beta1 on Fri Nov 23 09:13:21 2007 | http://epydoc.sourceforge.net |
