# Supplementary material for: MScanner: a classifier for retrieving Medline citations
Source: BMC Bioinformatics. 2008 Feb 19;9:108. doi: 10.1186/1471-2105-9-108 (PMC2263023; doi:10.1186/1471-2105-9-108)
Supplement: Additional file 3 — Source code for MScanner. mscanner-20071123.zip is a ZIP archive containing the Python 2.5 source code for MScanner, licensed under the GNU General Public License. It also contains API documentation in HTML format. Updated versions will be made available at . [file 1471-2105-9-108-S3.zip › mscanner/help/api/mscanner.htdocs.queue-module.html]

xml version="1.0" encoding="ascii"?


mscanner.htdocs.queue


| Trees | Indices | Help | | MScanner | | --- | |
| --- | --- | --- | --- | --- |

|  |  |  |  |
| --- | --- | --- | --- |
| Package mscanner :: Package htdocs :: Module queue | |  | | --- | | [hide private] | | [frames] | no frames] | |

# Module queue

source code  
  

Queueing facility for the web frontend

Queueing program checks queue directory every second for new
descriptor files, and starts a query or validation operation. When the
operation completes, the descriptor file is moved to the output.

Example descriptor file for query:

```
   #operation = query
   #dataset = Whatever
   #limit = 500
   #threshold = 10.3
   #submitted = 23424123.3
   804133
   3214241
   ...
```

Example descriptor file for validation:

```
   #operation = validate
   #dataset = Whatever
   #numnegs = 100000
   #alpha = 0.5
   #submitted = 23424123.3
   804133
   3214241
   ...
```

  
  


---

**Author:**
Graham Poulter <http://graham.poulter.googlepages.com>

**Copyright:**
2007 Graham Poulter

**License:**
This program is free software: you can redistribute it and/or
modify it under the terms of the GNU General Public License as
published by the
Free Software Foundation, either version 3 of the License, or (at
your option)
any later version.
This program is distributed in the hope that it will be useful, but
WITHOUT ANY
WARRANTY; without even the implied warranty of MERCHANTABILITY or
FITNESS FOR A
PARTICULAR PURPOSE. See the GNU General Public License for more
details.
You should have received a copy of the GNU General Public License
along with
this program. If not, see <http://www.gnu.org/licenses/>.


|  |  |  |  |
| --- | --- | --- | --- |
| |  |  | | --- | --- | | Classes | [hide private] | | |
|  | QueueStatus  Describes the current state of the queue |


|  |  |  |  |
| --- | --- | --- | --- |
| |  |  | | --- | --- | | Functions | [hide private] | | |
|  | |  |  | | --- | --- | | parsebool(s)  Handler for converting strings to booleans | source code | |
|  | |  |  | | --- | --- | | read\_descriptor(fpath)  Reads a descriptor file, returning a dictionary of parameters. | source code | |
|  | |  |  | | --- | --- | | write\_descriptor(fpath, pmids, params)  Write parameters and PubMed IDs to the descriptor file. | source code | |
|  | |  |  | | --- | --- | | delete\_output(dataset)  Delete the output directory for the given task | source code | |
|  | |  |  | | --- | --- | | logit(probability) | source code | |
|  | |  |  | | --- | --- | | mainloop()  Look for descriptor files every second | source code | |
|  | |  |  | | --- | --- | | populate\_test\_queue()  Place some dummy queue files to test the queue operation | source code | |


|  |  |  |  |
| --- | --- | --- | --- |
| |  |  | | --- | --- | | Variables | [hide private] | | |
|  | descriptor\_keys = `{'captcha': <type 'str'>, 'dataset': <type '...` |


|  |  |  |  |
| --- | --- | --- | --- |
| |  |  | | --- | --- | | Function Details | [hide private] | | |

|  |  |  |
| --- | --- | --- |
| |  |  | | --- | --- | | read\_descriptor(fpath) | source code |   Reads a descriptor file, returning a dictionary of parameters. Each line is '#key = value'. We stops at the first line that not starting with '#'. Valid keys are in descriptor\_keys. The same file can be used with read\_pmids, which will ignores the lines beginning with '#'. Returns:  Storage object, with additional '\_filename' containing fpath. |

|  |  |  |
| --- | --- | --- |
| |  |  | | --- | --- | | write\_descriptor(fpath, pmids, params) | source code |  Write parameters and PubMed IDs to the descriptor file. Parameters:  - **`fpath`** - File to write - **`pmids`** - List of PubMed IDs, may be None - **`params`** - Dictionary to write. Values are converted with str(). Only   keys from descriptor\_keys are used. |

  


|  |  |  |  |
| --- | --- | --- | --- |
| |  |  | | --- | --- | | Variables Details | [hide private] | | |

|  |  |
| --- | --- |
| descriptor\_keys   Value:  |  | | --- | | ``` {'captcha': <type 'str'>,  'dataset': <type 'str'>,  'delcode': <type 'str'>,  'hidden': <function parsebool at 0x014FC570>,  'limit': <type 'int'>,  'mindate': <type 'int'>,  'minscore': <type 'float'>,  'numnegs': <type 'int'>, ... ``` | |

  


| Trees | Indices | Help | | MScanner | | --- | |
| --- | --- | --- | --- | --- |

|  |  |
| --- | --- |
| Generated by Epydoc 3.0beta1 on Fri Nov 23 09:13:20 2007 | http://epydoc.sourceforge.net |
