# Supplementary material for: MScanner: a classifier for retrieving Medline citations
Source: BMC Bioinformatics. 2008 Feb 19;9:108. doi: 10.1186/1471-2105-9-108 (PMC2263023; doi:10.1186/1471-2105-9-108)
Supplement: Additional file 3 — Source code for MScanner. mscanner-20071123.zip is a ZIP archive containing the Python 2.5 source code for MScanner, licensed under the GNU General Public License. It also contains API documentation in HTML format. Updated versions will be made available at . [file 1471-2105-9-108-S3.zip › mscanner/help/api/mscanner.htdocs.queue-pysrc.html]

xml version="1.0" encoding="ascii"?


mscanner.htdocs.queue


| Trees | Indices | Help | | MScanner | | --- | |
| --- | --- | --- | --- | --- |

|  |  |  |  |
| --- | --- | --- | --- |
| Package mscanner :: Package htdocs :: Module queue | |  | | --- | | [hide private] | | [frames] | no frames] | |

# Source Code for Module mscanner.htdocs.queue

```
  1  #!/usr/bin/env python 
  2   
  3  """Queueing facility for the web frontend 
  4   
  5  Queueing program checks queue directory every second for new descriptor files, 
  6  and starts a query or validation operation. When the operation completes, the 
  7  descriptor file is moved to the output. 
  8   
  9  Example descriptor file for query:: 
 10      #operation = query 
 11      #dataset = Whatever 
 12      #limit = 500 
 13      #threshold = 10.3 
 14      #submitted = 23424123.3 
 15      804133 
 16      3214241 
 17      ... 
 18       
 19  Example descriptor file for validation:: 
 20      #operation = validate 
 21      #dataset = Whatever 
 22      #numnegs = 100000 
 23      #alpha = 0.5 
 24      #submitted = 23424123.3 
 25      804133 
 26      3214241 
 27      ... 
 28  """ 
 29   
 30  from __future__ import with_statement 
 31  from __future__ import division 
 32   
 33  import logging 
 34  import math 
 35  import os 
 36  from path import path 
 37  import sys 
 38  import time 
 39   
 40  from mscanner.configuration import rc 
 41  from mscanner.medline.Databases import Databases 
 42  from mscanner.core.QueryManager import QueryManager 
 43  from mscanner.core.ValidationManager import CrossValidation 
 44  from mscanner.core import iofuncs 
 45  from mscanner.scripts import update 
 46   
 47   
 48  __copyright__ = "2007 Graham Poulter" 
 49  __author__ = "Graham Poulter <http://graham.poulter.googlepages.com>" 
 50  __license__ = """This program is free software: you can redistribute it and/or 
 51  modify it under the terms of the GNU General Public License as published by the 
 52  Free Software Foundation, either version 3 of the License, or (at your option) 
 53  any later version. 
 54   
 55  This program is distributed in the hope that it will be useful, but WITHOUT ANY 
 56  WARRANTY; without even the implied warranty of MERCHANTABILITY or FITNESS FOR A 
 57  PARTICULAR PURPOSE. See the GNU General Public License for more details. 
 58   
 59  You should have received a copy of the GNU General Public License along with 
 60  this program. If not, see <http://www.gnu.org/licenses/>.""" 
 61   
 62   


63 -def parsebool(s):


64      """Handler for converting strings to booleans""" 
 65      if isinstance(s, basestring): 
 66          s = s.strip() 
 67          if s == "0" or s == "False": 
 68              return False 
 69          elif s == "1" or s == "True": 
 70              return True 
 71          else: 
 72              raise ValueError("Failed to parse boolean: %s" % s) 
 73      else: 
 74          return bool(s)

 75   
 76   
 77  descriptor_keys = dict( 
 78      captcha=str,      # Captcha value in the form 
 79      dataset=str,      # Name of the input corpus 
 80      delcode=str,      # MD5 of deletion code 
 81      hidden=parsebool, # Whether to hide the output 
 82      limit=int,        # Upper limit on number of results 
 83      mindate=int,      # Minimum date to consider 
 84      minscore=float,   # Minimum classifier score to predict relevance 
 85      numnegs=int,      # Number of irrelevant articles for CV 
 86      operation=str,    # "retrieval" or "validation" 
 87      prevalence=float, # Estimated fraction of relevant articles in Medline 
 88      submitted=float,  # Timestamp when the task was submitted 
 89      ) 
 90   
 91   


92 -def read_descriptor(fpath):


93      """Reads a descriptor file, returning a dictionary of parameters. 
 94   
 95      Each line is '#key = value'. We stops at the first line that not starting 
 96      with '#'.  Valid keys are in L{descriptor_keys}. The same file can be used 
 97      with read_pmids, which will ignores the lines beginning with '#'. 
 98   
 99      @return: Storage object, with additional '_filename' containing fpath.""" 
100      from mscanner.core.Storage import Storage 
101      result = Storage() 
102      with open(fpath, "r") as f: 
103          line = f.readline() 
104          while line.startswith("#"): 
105              key, value = line[1:].split(" = ",1) 
106              value = value.strip() 
107              if value == "None":  
108                  value = None 
109              else: 
110                  value = descriptor_keys[key](value.strip()) 
111              result[key] = value 
112              line = f.readline() 
113          result["_filename"] = fpath 
114      return result

115   
116   


117 -def write_descriptor(fpath, pmids, params):


118      """Write parameters and PubMed IDs to the descriptor file. 
119      @param fpath: File to write 
120      @param pmids: List of PubMed IDs, may be None 
121      @param params: Dictionary to write. Values are converted with str(). Only 
122      keys from descriptor_keys are used.""" 
123      with open(fpath, "w") as f: 
124          fpath.chmod(0777) 
125          for key, value in params.iteritems(): 
126              if key in descriptor_keys:  
127                  f.write("#" + key + " = " + str(value) + "\n") 
128          if pmids is not None: 
129              for pmid in pmids: 
130                  f.write(str(pmid)+"\n")

131   
132   


133 -class QueueStatus:


134      """Describes the current state of the queue 
135       
136      @ivar tasklist: Descriptors of tasks in the queue, oldest first. 
137       
138      @ivar running: First member of L{tasklist}, which is being processed. 
139       
140      @ivar donelist: Completed tasks, oldest first. 
141       
142      @ivar status: Mapping from dataset to status code (DONE, RUNNING, WAITING) 
143       
144      @ivar _tasks: Mapping from dataset to task object 
145      """ 
146       
147      DONE = "done" 
148      RUNNING = "running" 
149      WAITING = "waiting" 
150       


151 -    def __init__(self, with_done=True):


152          """Constructor for the status 
153           
154          @param with_done: Set this to False if you don't need L{donelist}.""" 
155          self._load_tasklist() 
156          self.donelist = [] 
157          if with_done: self._load_donelist() 
158          self._load_maps()

159   
160       


161 -    def _load_tasklist(self):


162          """Populate L{tasklist}. 
163           
164          @note: We only load files that are older than 1/20th second. Without 
165          this we sometimes catch files half-written by the web interface. This 
166          in turn means query_logic.py has to wait 0.05 seconds before going to 
167          the status page, so that the task shows up. 
168          """ 
169          current_time = time.time() 
170          eligible_files = [f for f in rc.queue_path.files() \ 
171                            if f.mtime < current_time-0.05] 
172          self.tasklist = [read_descriptor(f) for f in eligible_files] 
173          self.tasklist.sort(key=lambda x:x.submitted) 
174          self.running = self.tasklist[0] if self.tasklist else None

175   
176   


177 -    def _load_donelist(self):


178          """Populate L{donelist}""" 
179          self.donelist = [] 
180          for fpath in rc.web_report_dir.dirs(): 
181              if (fpath/rc.report_descriptor).exists(): 
182                  self.donelist.append( 
183                      read_descriptor(fpath/rc.report_descriptor)) 
184          self.donelist.sort(key=lambda x:x.submitted)

185   
186   


187 -    def _load_maps(self):


188          """Calculate the L{status} and L{_tasks} mapping""" 
189          self.status = {} 
190          self._tasks = {} 
191          for task in self.tasklist: 
192              self.status[task.dataset] = self.WAITING 
193              self._tasks[task.dataset] = task 
194          for task in self.donelist: 
195              self.status[task.dataset] = self.DONE 
196              self._tasks[task.dataset] = task 
197          if self.tasklist: 
198              self.status[self.running.dataset] = self.RUNNING

199   
200       


201 -    def __getitem__(self, dataset):


202          """Retrieve the task descriptor for a given data set.""" 
203          return self._tasks.__getitem__(dataset)

204   
205   


206 -    def __contains__(self, dataset):


207          """Return whether given dataset exists""" 
208          return self._tasks.__contains__(dataset)

209   
210   


211 -    def position(self, dataset):


212          """Return distance of dataset from front of queue.""" 
213          for idx, d in enumerate(self.tasklist): 
214              if d.dataset == dataset: 
215                  return idx 
216          return None

217   
218       
219       
220   


221 -def delete_output(dataset):


222      """Delete the output directory for the given task""" 
223      logging.debug("Attempting to delete output for %s" % dataset) 
224      dirpath = rc.web_report_dir / dataset 
225      for fname in dirpath.files(): 
226          fname.remove() 
227      dirpath.rmdir()

228   
229   
230   


231 -def logit(probability):


232      if probability is None: 
233          return None 
234      else: 
235          return math.log(probability/(1-probability))

236   
237   


238 -def mainloop():


239      """Look for descriptor files every second""" 
240      env = None 
241      try: 
242          # time.time() of last output-cleaning 
243          last_clean = 0  
244          # time.time() of last database update 
245          last_update = 0  
246          while True: 
247              # Delete oldest outputs twice daily 
248              if time.time() - last_clean > 12*3600:   
249                  logging.info("Looking for old datasets") 
250                  queue = QueueStatus() 
251                  queue.donelist.reverse() # Newest first 
252                  for task in queue.donelist[100:]: 
253                      try: 
254                          delete_output(task.dataset) 
255                      except OSError: 
256                          pass # Failed to delete output 
257                  last_clean = time.time() 
258               
259              # Update the databases twice daily 
260              if time.time() - last_update > 12*3600: 
261                  if env is not None: env.close() 
262                  env = None 
263                  update.update_mscanner() 
264                  env = Databases() 
265                  env.article_list # long first load time 
266                  last_update = time.time() 
267               
268              # Perform any queued tasks 
269              queue = QueueStatus() 
270              task = queue.running 
271              if task is not None: 
272                  # The output directory for the task 
273                  outdir = rc.web_report_dir / task.dataset 
274                  logging.info("Starting %s for %s", task.operation, task.dataset) 
275                  # Update task file mod time for the status display 
276                  task._filename.utime(None)  
277                  try: 
278                      if task.operation == "retrieval": 
279                          QM = QueryManager( 
280                              outdir=outdir,  
281                              dataset=task.dataset, 
282                              limit=task.limit, 
283                              env=env, 
284                              threshold=task.minscore, 
285                              prior=logit(task.prevalence), 
286                              mindate=task.mindate, 
287                              maxdate=None, 
288                              ) 
289                          QM.query(task._filename) 
290                          time.sleep(5) 
291                          QM.write_report() 
292                          QM.__del__() 
293                      elif task.operation == "validate": 
294                          VM = CrossValidation( 
295                              outdir=outdir,  
296                              dataset=task.dataset, 
297                              env=env) 
298                          VM.validation(task._filename, task.numnegs) 
299                          VM.report_validation() 
300                          VM.__del__() 
301                      task._filename.move(outdir / "descriptor.txt") 
302                  except ValueError, e: 
303                      logging.exception(e) 
304              else: 
305                  # Nothing to do so sleep before the next iteration 
306                  time.sleep(1) 
307      finally: 
308          if env is not None: env.close()

309   
310   


311 -def populate_test_queue():


312      """Place some dummy queue files to test the queue operation""" 
313      from mscanner.core.Storage import Storage 
314      pmids = list(iofuncs.read_pmids(rc.corpora / "Test" / "gdsmall.txt")) 
315      task = Storage( 
316          captcha = "orange", 
317          dataset = "gdqtest_valid", 
318          hidden = False, 
319          limit = 500, 
320          mindate = 19700101, 
321          minscore = 0.0,  
322          numnegs = 1000,  
323          operation = "validate",  
324          prevalence = 0.01, 
325          submitted = time.time()) 
326      write_descriptor(rc.queue_path/task.dataset, pmids, task) 
327      task.operation = "retrieval" 
328      task.dataset = "gdqtest_query" 
329      task.submitted += 5 
330      write_descriptor(rc.queue_path/task.dataset, pmids, task)

331   
332   
333  if __name__ == "__main__": 
334      iofuncs.start_logger() 
335      if len(sys.argv) == 2 and sys.argv[1] == "test": 
336          populate_test_queue() 
337      try: 
338          mainloop() 
339      except KeyboardInterrupt: 
340          pass 
341      logging.shutdown() 
342
```

  


| Trees | Indices | Help | | MScanner | | --- | |
| --- | --- | --- | --- | --- |

|  |  |
| --- | --- |
| Generated by Epydoc 3.0beta1 on Fri Nov 23 09:13:23 2007 | http://epydoc.sourceforge.net |
