# Supplementary material for: MScanner: a classifier for retrieving Medline citations
Source: BMC Bioinformatics. 2008 Feb 19;9:108. doi: 10.1186/1471-2105-9-108 (PMC2263023; doi:10.1186/1471-2105-9-108)
Supplement: Additional file 3 — Source code for MScanner. mscanner-20071123.zip is a ZIP archive containing the Python 2.5 source code for MScanner, licensed under the GNU General Public License. It also contains API documentation in HTML format. Updated versions will be made available at . [file 1471-2105-9-108-S3.zip › mscanner/help/api/mscanner.htdocs.queue.QueueStatus-class.html]

xml version="1.0" encoding="ascii"?


mscanner.htdocs.queue.QueueStatus


| Trees | Indices | Help | | MScanner | | --- | |
| --- | --- | --- | --- | --- |

|  |  |  |  |
| --- | --- | --- | --- |
| Package mscanner :: Package htdocs :: Module queue :: Class QueueStatus | |  | | --- | | [hide private] | | [frames] | no frames] | |

# Class QueueStatus

source code  
  
Describes the current state of the queue  
  


|  |  |  |  |
| --- | --- | --- | --- |
| |  |  | | --- | --- | | Instance Methods | [hide private] | | |
|  | |  |  | | --- | --- | | \_\_init\_\_(self, with\_done=True)  Constructor for the status | source code | |
|  | |  |  | | --- | --- | | \_load\_tasklist(self)  Populate tasklist. | source code | |
|  | |  |  | | --- | --- | | \_load\_donelist(self)  Populate donelist | source code | |
|  | |  |  | | --- | --- | | \_load\_maps(self)  Calculate the status and \_tasks mapping | source code | |
|  | |  |  | | --- | --- | | \_\_getitem\_\_(self, dataset)  Retrieve the task descriptor for a given data set. | source code | |
|  | |  |  | | --- | --- | | \_\_contains\_\_(self, dataset)  Return whether given dataset exists | source code | |
|  | |  |  | | --- | --- | | position(self, dataset)  Return distance of dataset from front of queue. | source code | |


|  |  |  |  |
| --- | --- | --- | --- |
| |  |  | | --- | --- | | Class Variables | [hide private] | | |
|  | DONE = `'done'` |
|  | RUNNING = `'running'` |
|  | WAITING = `'waiting'` |


|  |  |  |  |
| --- | --- | --- | --- |
| |  |  | | --- | --- | | Instance Variables | [hide private] | | |
|  | \_tasks  Mapping from dataset to task object |
|  | donelist  Completed tasks, oldest first. |
|  | running  First member of tasklist, which is being processed. |
|  | status  Mapping from dataset to status code (DONE, RUNNING, WAITING) |
|  | tasklist  Descriptors of tasks in the queue, oldest first. |


|  |  |  |  |
| --- | --- | --- | --- |
| |  |  | | --- | --- | | Method Details | [hide private] | | |

|  |  |  |
| --- | --- | --- |
| |  |  | | --- | --- | | \_\_init\_\_(self, with\_done=True)  *(Constructor)* | source code |  Constructor for the status Parameters:  - **`with_done`** - Set this to False if you don't need donelist. |

|  |  |  |
| --- | --- | --- |
| |  |  | | --- | --- | | \_load\_tasklist(self) | source code |  Populate tasklist.   **Note:** We only load files that are older than 1/20th second. Without this we sometimes catch files half-written by the web interface. This in turn means query\_logic.py has to wait 0.05 seconds before going to the status page, so that the task shows up. |

  


| Trees | Indices | Help | | MScanner | | --- | |
| --- | --- | --- | --- | --- |

|  |  |
| --- | --- |
| Generated by Epydoc 3.0beta1 on Fri Nov 23 09:13:21 2007 | http://epydoc.sourceforge.net |
