# Supplementary material for: MScanner: a classifier for retrieving Medline citations
Source: BMC Bioinformatics. 2008 Feb 19;9:108. doi: 10.1186/1471-2105-9-108 (PMC2263023; doi:10.1186/1471-2105-9-108)
Supplement: Additional file 3 — Source code for MScanner. mscanner-20071123.zip is a ZIP archive containing the Python 2.5 source code for MScanner, licensed under the GNU General Public License. It also contains API documentation in HTML format. Updated versions will be made available at . [file 1471-2105-9-108-S3.zip › mscanner/help/api/mscanner.htdocs.templates-module.html]

xml version="1.0" encoding="ascii"?


mscanner.htdocs.templates


| Trees | Indices | Help | | MScanner | | --- | |
| --- | --- | --- | --- | --- |

|  |  |  |  |
| --- | --- | --- | --- |
| Package mscanner :: Package htdocs :: Package templates | |  | | --- | | [hide private] | | [frames] | no frames] | |

# Package templates

source code  
  


|  |  |  |  |
| --- | --- | --- | --- |
| |  |  | | --- | --- | | Submodules | [hide private] | | |
| - **mscanner.htdocs.templates.contact** - **mscanner.htdocs.templates.contact\_logic**: *web.py handler for the contact page* - **mscanner.htdocs.templates.front** - **mscanner.htdocs.templates.output** - **mscanner.htdocs.templates.output\_logic**: *web.py handler for the output listing page* - **mscanner.htdocs.templates.page** - **mscanner.htdocs.templates.query** - **mscanner.htdocs.templates.query\_logic**: *web.py handler for the query submission page* - **mscanner.htdocs.templates.status** - **mscanner.htdocs.templates.status\_logic**: *web.py handler for the status page* |

  


| Trees | Indices | Help | | MScanner | | --- | |
| --- | --- | --- | --- | --- |

|  |  |
| --- | --- |
| Generated by Epydoc 3.0beta1 on Fri Nov 23 09:13:20 2007 | http://epydoc.sourceforge.net |
