# Supplementary material for: MScanner: a classifier for retrieving Medline citations
Source: BMC Bioinformatics. 2008 Feb 19;9:108. doi: 10.1186/1471-2105-9-108 (PMC2263023; doi:10.1186/1471-2105-9-108)
Supplement: Additional file 3 — Source code for MScanner. mscanner-20071123.zip is a ZIP archive containing the Python 2.5 source code for MScanner, licensed under the GNU General Public License. It also contains API documentation in HTML format. Updated versions will be made available at . [file 1471-2105-9-108-S3.zip › mscanner/help/api/mscanner.htdocs.templates.contact-pysrc.html]

xml version="1.0" encoding="ascii"?


mscanner.htdocs.templates.contact


| Trees | Indices | Help | | MScanner | | --- | |
| --- | --- | --- | --- | --- |

|  |  |  |  |
| --- | --- | --- | --- |
| Package mscanner :: Package htdocs :: Package templates :: Module contact | |  | | --- | | [hide private] | | [frames] | no frames] | |

# Source Code for Module mscanner.htdocs.templates.contact

```
  1  #!/usr/bin/env python 
  2   
  3   
  4   
  5   
  6  ################################################## 
  7  ## DEPENDENCIES 
  8  import sys 
  9  import os 
 10  import os.path 
 11  from os.path import getmtime, exists 
 12  import time 
 13  import types 
 14  import __builtin__ 
 15  from Cheetah.Version import MinCompatibleVersion as RequiredCheetahVersion 
 16  from Cheetah.Version import MinCompatibleVersionTuple as RequiredCheetahVersionTuple 
 17  from Cheetah.Template import Template 
 18  from Cheetah.DummyTransaction import DummyTransaction 
 19  from Cheetah.NameMapper import NotFound, valueForName, valueFromSearchList, valueFromFrameOrSearchList 
 20  from Cheetah.CacheRegion import CacheRegion 
 21  import Cheetah.Filters as Filters 
 22  import Cheetah.ErrorCatchers as ErrorCatchers 
 23  from page import page 
 24   
 25  ################################################## 
 26  ## MODULE CONSTANTS 
 27  try: 
 28      True, False 
 29  except NameError: 
 30      True, False = (1==1), (1==0) 
 31  VFFSL=valueFromFrameOrSearchList 
 32  VFSL=valueFromSearchList 
 33  VFN=valueForName 
 34  currentTime=time.time 
 35  __CHEETAH_version__ = '2.0rc7' 
 36  __CHEETAH_versionTuple__ = (2, 0, 0, 'candidate', 7) 
 37  __CHEETAH_genTime__ = 1193401028.3199999 
 38  __CHEETAH_genTimestamp__ = 'Fri Oct 26 14:17:08 2007' 
 39  __CHEETAH_src__ = 'contact.tmpl' 
 40  __CHEETAH_srcLastModified__ = 'Fri Oct 26 14:17:08 2007' 
 41  __CHEETAH_docstring__ = 'Autogenerated by CHEETAH: The Python-Powered Template Engine' 
 42   
 43  if __CHEETAH_versionTuple__ < RequiredCheetahVersionTuple: 
 44      raise AssertionError( 
 45        'This template was compiled with Cheetah version' 
 46        ' %s. Templates compiled before version %s must be recompiled.'%( 
 47           __CHEETAH_version__, RequiredCheetahVersion)) 
 48   
 49  ################################################## 
 50  ## CLASSES 
 51   


52 -class contact(page):


53   
 54      ################################################## 
 55      ## CHEETAH GENERATED METHODS 
 56   
 57   


58 -    def __init__(self, *args, **KWs):


59   
 60          page.__init__(self, *args, **KWs) 
 61          if not self._CHEETAH__instanceInitialized: 
 62              cheetahKWArgs = {} 
 63              allowedKWs = 'searchList namespaces filter filtersLib errorCatcher'.split() 
 64              for k,v in KWs.items(): 
 65                  if k in allowedKWs: cheetahKWArgs[k] = v 
 66              self._initCheetahInstance(**cheetahKWArgs)

 67           
 68   


69 -    def title(self, **KWS):


70   
 71   
 72   
 73          ## CHEETAH: generated from #def title at line 3, col 1. 
 74          trans = KWS.get("trans") 
 75          if (not trans and not self._CHEETAH__isBuffering and not callable(self.transaction)): 
 76              trans = self.transaction # is None unless self.awake() was called 
 77          if not trans: 
 78              trans = DummyTransaction() 
 79              _dummyTrans = True 
 80          else: _dummyTrans = False 
 81          write = trans.response().write 
 82          SL = self._CHEETAH__searchList 
 83          _filter = self._CHEETAH__currentFilter 
 84           
 85          ######################################## 
 86          ## START - generated method body 
 87           
 88          write('Contact the Author\n') 
 89           
 90          ######################################## 
 91          ## END - generated method body 
 92           
 93          return _dummyTrans and trans.response().getvalue() or ""

 94           
 95   


96 -    def contents(self, **KWS):


97   
 98   
 99   
100          ## CHEETAH: generated from #def contents at line 7, col 1. 
101          trans = KWS.get("trans") 
102          if (not trans and not self._CHEETAH__isBuffering and not callable(self.transaction)): 
103              trans = self.transaction # is None unless self.awake() was called 
104          if not trans: 
105              trans = DummyTransaction() 
106              _dummyTrans = True 
107          else: _dummyTrans = False 
108          write = trans.response().write 
109          SL = self._CHEETAH__searchList 
110          _filter = self._CHEETAH__currentFilter 
111           
112          ######################################## 
113          ## START - generated method body 
114           
115          write('<div class="narrow">\n\n') 
116          if VFSL([locals()]+SL+[globals(), __builtin__],"getVar",False)("success", None) is not None: # generated from line 10, col 1 
117              write('    <p>\n') 
118              if VFSL([locals()]+SL+[globals(), __builtin__],"success",True): # generated from line 12, col 5 
119                  write('        Your message was sent successfully:\n') 
120              else: # generated from line 14, col 5 
121                  write('        Failed to send the email! Please contact \n        <a href="mailto:mscanner.w3f@gishpuppy.com">Graham poulter</a>.\n        Error: <pre>') 
122                  _v = VFSL([locals()]+SL+[globals(), __builtin__],"error",True) # '$error' on line 17, col 21 
123                  if _v is not None: write(_filter(_v, rawExpr='$error')) # from line 17, col 21. 
124                  write('</pre>\n') 
125              write('    </p>\n') 
126          write('\n<form action="contact" method="post">\n') 
127          _v = VFN(VFSL([locals()]+SL+[globals(), __builtin__],"inputs",True),"render",False)() # '$inputs.render()' on line 23, col 1 
128          if _v is not None: write(_filter(_v, rawExpr='$inputs.render()')) # from line 23, col 1. 
129          write(''' 
130  <p><input type="submit" value="Send Message"></p> 
131  </form> 
132   
133  </div><!--class=narrow--> 
134  ''') 
135           
136          ######################################## 
137          ## END - generated method body 
138           
139          return _dummyTrans and trans.response().getvalue() or ""

140           
141   


142 -    def writeBody(self, **KWS):


143   
144   
145   
146          ## CHEETAH: main method generated for this template 
147          trans = KWS.get("trans") 
148          if (not trans and not self._CHEETAH__isBuffering and not callable(self.transaction)): 
149              trans = self.transaction # is None unless self.awake() was called 
150          if not trans: 
151              trans = DummyTransaction() 
152              _dummyTrans = True 
153          else: _dummyTrans = False 
154          write = trans.response().write 
155          SL = self._CHEETAH__searchList 
156          _filter = self._CHEETAH__currentFilter 
157           
158          ######################################## 
159          ## START - generated method body 
160           
161          write('\n\n') 
162           
163          ######################################## 
164          ## END - generated method body 
165           
166          return _dummyTrans and trans.response().getvalue() or ""

167           
168      ################################################## 
169      ## CHEETAH GENERATED ATTRIBUTES 
170   
171   
172      _CHEETAH__instanceInitialized = False 
173   
174      _CHEETAH_version = __CHEETAH_version__ 
175   
176      _CHEETAH_versionTuple = __CHEETAH_versionTuple__ 
177   
178      _CHEETAH_genTime = __CHEETAH_genTime__ 
179   
180      _CHEETAH_genTimestamp = __CHEETAH_genTimestamp__ 
181   
182      _CHEETAH_src = __CHEETAH_src__ 
183   
184      _CHEETAH_srcLastModified = __CHEETAH_srcLastModified__ 
185   
186      _mainCheetahMethod_for_contact= 'writeBody'

187   
188  ## END CLASS DEFINITION 
189   
190  if not hasattr(contact, '_initCheetahAttributes'): 
191      templateAPIClass = getattr(contact, '_CHEETAH_templateClass', Template) 
192      templateAPIClass._addCheetahPlumbingCodeToClass(contact) 
193   
194   
195  # CHEETAH was developed by Tavis Rudd and Mike Orr 
196  # with code, advice and input from many other volunteers. 
197  # For more information visit http://www.CheetahTemplate.org/ 
198   
199  ################################################## 
200  ## if run from command line: 
201  if __name__ == '__main__': 
202      from Cheetah.TemplateCmdLineIface import CmdLineIface 
203      CmdLineIface(templateObj=contact()).run() 
204
```

  


| Trees | Indices | Help | | MScanner | | --- | |
| --- | --- | --- | --- | --- |

|  |  |
| --- | --- |
| Generated by Epydoc 3.0beta1 on Fri Nov 23 09:13:24 2007 | http://epydoc.sourceforge.net |
