# Supplementary material for: MScanner: a classifier for retrieving Medline citations
Source: BMC Bioinformatics. 2008 Feb 19;9:108. doi: 10.1186/1471-2105-9-108 (PMC2263023; doi:10.1186/1471-2105-9-108)
Supplement: Additional file 3 — Source code for MScanner. mscanner-20071123.zip is a ZIP archive containing the Python 2.5 source code for MScanner, licensed under the GNU General Public License. It also contains API documentation in HTML format. Updated versions will be made available at . [file 1471-2105-9-108-S3.zip › mscanner/help/api/mscanner.htdocs.templates.contact.contact-class.html]

xml version="1.0" encoding="ascii"?


mscanner.htdocs.templates.contact.contact


| Trees | Indices | Help | | MScanner | | --- | |
| --- | --- | --- | --- | --- |

|  |  |  |  |
| --- | --- | --- | --- |
| Package mscanner :: Package htdocs :: Package templates :: Module contact :: Class contact | |  | | --- | | [hide private] | | [frames] | no frames] | |

# Class contact

source code  
  

```
                 object --+                
                          |                
Cheetah.Servlet.BaseServlet --+            
                              |            
        Cheetah.Servlet.Servlet --+        
                                  |        
          Cheetah.Template.Template --+    
                                      |    
                              page.page --+
                                          |
                                         contact
```

---


|  |  |  |  |
| --- | --- | --- | --- |
| |  |  | | --- | --- | | Nested Classes | [hide private] | | |
| **Inherited from `Cheetah.Template.Template`**: `NonNumericInputError`  **Inherited from `Cheetah.Template.Template`** (private): `_CHEETAH_cacheRegionClass`, `_CHEETAH_cacheStoreClass`, `_CHEETAH_compilerClass`, `_CHEETAH_defaultPreprocessorClass` | |


|  |  |  |  |
| --- | --- | --- | --- |
| |  |  | | --- | --- | | Instance Methods | [hide private] | | |
|  | |  |  | | --- | --- | | \_\_init\_\_(self, \*args, \*\*KWs) | source code | |
|  | |  |  | | --- | --- | | title(self, \*\*KWS) | source code | |
|  | |  |  | | --- | --- | | contents(self, \*\*KWS) | source code | |
|  | |  |  | | --- | --- | | writeBody(self, \*\*KWS) | source code | |
| **Inherited from `page.page`**: `__str__`, `body`, `contents_outer`, `doctype`, `extraheaders`, `footer`, `footer_text`, `head`, `header`, `header_text`, `html`, `respond`, `statusblock`, `stdheaders`, `topmenu`, `wintitle`  **Inherited from `Cheetah.Template.Template`**: `errorCatcher`, `generatedClassCode`, `generatedModuleCode`, `getCacheRegion`, `getCacheRegions`, `getFileContents`, `getVar`, `hasVar`, `i18n`, `refreshCache`, `runAsMainProgram`, `searchList`, `shutdown`, `varExists`, `webInput`  **Inherited from `Cheetah.Template.Template`** (private): `_compile`, `_createCacheRegion`, `_getCacheStore`, `_getCacheStoreIdPrefix`, `_getTemplateAPIClassForIncludeDirectiveCompilation`, `_handleCheetahInclude`, `_initCheetahInstance`  **Inherited from `Cheetah.Servlet.Servlet`**: `awake`, `serverSidePath`, `sleep`  **Inherited from `object`**: `__delattr__`, `__getattribute__`, `__hash__`, `__new__`, `__reduce__`, `__reduce_ex__`, `__repr__`, `__setattr__` | |


|  |  |  |  |
| --- | --- | --- | --- |
| |  |  | | --- | --- | | Class Methods | [hide private] | | |
| **Inherited from `Cheetah.Template.Template`**: `compile`, `subclass`  **Inherited from `Cheetah.Template.Template`** (private): `_addCheetahPlumbingCodeToClass`, `_getCompilerClass`, `_getCompilerSettings`, `_normalizePreprocessorArg`, `_normalizePreprocessorSettings`, `_preprocessSource`, `_updateSettingsWithPreprocessTokens` | |


|  |  |  |  |
| --- | --- | --- | --- |
| |  |  | | --- | --- | | Class Variables | [hide private] | | |
|  | \_CHEETAH\_\_instanceInitialized = `False` |
|  | \_CHEETAH\_version = `'2.0rc7'` |
|  | \_CHEETAH\_versionTuple = `(2, 0, 0, 'candidate', 7)` |
|  | \_CHEETAH\_genTime = `1193401028.32` |
|  | \_CHEETAH\_genTimestamp = `'Fri Oct 26 14:17:08 2007'` |
|  | \_CHEETAH\_src = `'contact.tmpl'` |
|  | \_CHEETAH\_srcLastModified = `'Fri Oct 26 14:17:08 2007'` |
|  | \_mainCheetahMethod\_for\_contact = `'writeBody'` |
| **Inherited from `page.page`**: `base`  **Inherited from `page.page`** (private): `_mainCheetahMethod_for_page`  **Inherited from `Cheetah.Template.Template`** (private): `_CHEETAH_cacheCompilationResults`, `_CHEETAH_cacheDirForModuleFiles`, `_CHEETAH_cacheModuleFilesForTracebacks`, `_CHEETAH_cacheStore`, `_CHEETAH_cacheStoreIdPrefix`, `_CHEETAH_compileCache`, `_CHEETAH_compileLock`, `_CHEETAH_compilerSettings`, `_CHEETAH_defaultBaseclassForTemplates`, `_CHEETAH_defaultClassNameForTemplates`, `_CHEETAH_defaultMainMethodName`, `_CHEETAH_defaultMainMethodNameForTemplates`, `_CHEETAH_defaultModuleGlobalsForTemplates`, `_CHEETAH_defaultModuleNameForTemplates`, `_CHEETAH_generatedModuleCode`, `_CHEETAH_keepRefToGeneratedCode`, `_CHEETAH_preprocessors`, `_CHEETAH_requiredCheetahClassAttributes`, `_CHEETAH_requiredCheetahClassMethods`, `_CHEETAH_requiredCheetahMethods`, `_CHEETAH_useCompilationCache`  **Inherited from `Cheetah.Servlet.Servlet`**: `application`, `request`, `session`, `transaction`  **Inherited from `Cheetah.Servlet.BaseServlet`** (private): `_reusable`, `_threadSafe` | |


|  |  |  |  |
| --- | --- | --- | --- |
| |  |  | | --- | --- | | Properties | [hide private] | | |
| **Inherited from `object`**: `__class__` | |


|  |  |  |  |
| --- | --- | --- | --- |
| |  |  | | --- | --- | | Method Details | [hide private] | | |

|  |  |  |
| --- | --- | --- |
| |  |  | | --- | --- | | \_\_init\_\_(self, \*args, \*\*KWs)  *(Constructor)* | source code |   Overrides: page.page.\_\_init\_\_ |

|  |  |  |
| --- | --- | --- |
| |  |  | | --- | --- | | title(self, \*\*KWS) | source code |   Overrides: page.page.title |

|  |  |  |
| --- | --- | --- |
| |  |  | | --- | --- | | contents(self, \*\*KWS) | source code |   Overrides: page.page.contents |

  


| Trees | Indices | Help | | MScanner | | --- | |
| --- | --- | --- | --- | --- |

|  |  |
| --- | --- |
| Generated by Epydoc 3.0beta1 on Fri Nov 23 09:13:21 2007 | http://epydoc.sourceforge.net |
