# Supplementary material for: MScanner: a classifier for retrieving Medline citations
Source: BMC Bioinformatics. 2008 Feb 19;9:108. doi: 10.1186/1471-2105-9-108 (PMC2263023; doi:10.1186/1471-2105-9-108)
Supplement: Additional file 3 — Source code for MScanner. mscanner-20071123.zip is a ZIP archive containing the Python 2.5 source code for MScanner, licensed under the GNU General Public License. It also contains API documentation in HTML format. Updated versions will be made available at . [file 1471-2105-9-108-S3.zip › mscanner/help/api/mscanner.htdocs.templates.contact_logic-module.html]

xml version="1.0" encoding="ascii"?


mscanner.htdocs.templates.contact\_logic


| Trees | Indices | Help | | MScanner | | --- | |
| --- | --- | --- | --- | --- |

|  |  |  |  |
| --- | --- | --- | --- |
| Package mscanner :: Package htdocs :: Package templates :: Module contact\_logic | |  | | --- | | [hide private] | | [frames] | no frames] | |

# Module contact\_logic

source code  
  
web.py handler for the contact page  
  


---

**Author:**
Graham Poulter <http://graham.poulter.googlepages.com>

**Copyright:**
2007 Graham Poulter

**License:**
GPL


|  |  |  |  |
| --- | --- | --- | --- |
| |  |  | | --- | --- | | Classes | [hide private] | | |
|  | ContactPage  Form to contact the webmaster |


|  |  |  |  |
| --- | --- | --- | --- |
| |  |  | | --- | --- | | Variables | [hide private] | | |
|  | ContactForm = `forms.Form(forms.Textbox("captcha", forms.Valida...`  Structure for the form on the contact page |


|  |  |  |  |
| --- | --- | --- | --- |
| |  |  | | --- | --- | | Variables Details | [hide private] | | |

|  |  |
| --- | --- |
| ContactFormStructure for the form on the contact page   Value:  |  | | --- | | ``` forms.Form(forms.Textbox("captcha", forms.Validator(lambda x: x== "ora nge", "Should be the word 'orange'"), label= "The word 'orange'", size = 10), forms.Textbox("name", forms.Validator(lambda x: len(x) < 50, "S hould be less than 50 characters"), label= "Name (optional)", size= 35 ), forms.Textbox("email", forms.Validator(lambda x: len(x) < 80, "Shou ld be less than 80 characters"), label= "Email (optional)", size= 35),  forms.Textarea("message", forms.Validator(lambda x: len(x) < 2000, "S hould be less than 2000 characters"), label= "Message", rows= 10, cols ... ``` | |

  


| Trees | Indices | Help | | MScanner | | --- | |
| --- | --- | --- | --- | --- |

|  |  |
| --- | --- |
| Generated by Epydoc 3.0beta1 on Fri Nov 23 09:13:20 2007 | http://epydoc.sourceforge.net |
