# Supplementary material for: MScanner: a classifier for retrieving Medline citations
Source: BMC Bioinformatics. 2008 Feb 19;9:108. doi: 10.1186/1471-2105-9-108 (PMC2263023; doi:10.1186/1471-2105-9-108)
Supplement: Additional file 3 — Source code for MScanner. mscanner-20071123.zip is a ZIP archive containing the Python 2.5 source code for MScanner, licensed under the GNU General Public License. It also contains API documentation in HTML format. Updated versions will be made available at . [file 1471-2105-9-108-S3.zip › mscanner/help/api/mscanner.htdocs.templates.contact_logic-pysrc.html]

xml version="1.0" encoding="ascii"?


mscanner.htdocs.templates.contact\_logic


| Trees | Indices | Help | | MScanner | | --- | |
| --- | --- | --- | --- | --- |

|  |  |  |  |
| --- | --- | --- | --- |
| Package mscanner :: Package htdocs :: Package templates :: Module contact\_logic | |  | | --- | | [hide private] | | [frames] | no frames] | |

# Source Code for Module mscanner.htdocs.templates.contact\_logic

```
 1  """web.py handler for the contact page""" 
 2   
 3  import web 
 4  import contact 
 5  from mscanner.htdocs import forms 
 6   
 7  __copyright__ = "2007 Graham Poulter" 
 8  __author__ = "Graham Poulter <http://graham.poulter.googlepages.com>" 
 9  __license__ = "GPL" 
10   
11  ContactForm = forms.Form( 
12       
13      forms.Textbox( 
14          "captcha", 
15          forms.Validator(lambda x: x == "orange", "Should be the word 'orange'"), 
16          label="The word 'orange'", size=10), 
17       
18      forms.Textbox( 
19          "name", 
20          forms.Validator(lambda x: len(x) < 50, "Should be less than 50 characters"), 
21          label="Name (optional)", size=35), 
22       
23      forms.Textbox( 
24          "email", 
25          forms.Validator(lambda x: len(x) < 80, "Should be less than 80 characters"),     
26          label="Email (optional)", size=35),  
27       
28      forms.Textarea( 
29          "message", 
30          forms.Validator(lambda x: len(x) < 2000, "Should be less than 2000 characters"), 
31          label="Message", rows=10, cols=40), 
32   
33  ) 
34  """Structure for the form on the contact page""" 
35   
36   
37   


38 -class ContactPage:


39      """Form to contact the webmaster""" 
40       


41 -    def GET(self):


42          """Print the contact form""" 
43          web.header('Content-Type', 'text/html; charset=utf-8')  
44          page = contact.contact() 
45          page.inputs = ContactForm() 
46          print page

47       
48       


49 -    def POST(self):


50          """Submit the contact form.""" 
51          import re 
52          sanitize = lambda s: re.sub(r"[/!#$%^&*{}[]|\\]+", "", s) 
53          web.header('Content-Type', 'text/html; charset=utf-8')  
54          page = contact.contact() 
55          cform = ContactForm() 
56          if cform.validates(web.input()): 
57              email = sanitize(cform.d.email) or "nobody@maples.stanford.edu" 
58              name = sanitize(cform.d.name) 
59              message = sanitize(cform.d.message) 
60              import smtplib 
61              from email.mime.text import MIMEText 
62              from mscanner.configuration import rc 
63              msg = MIMEText(message) 
64              msg['Subject'] = "Contact from MScanner" 
65              msg['From'] = name + "<" + email + ">" 
66              msg['To'] = rc.webmaster_email 
67              server = smtplib.SMTP(rc.smtpserver) 
68              try: 
69                  server.sendmail(email, rc.webmaster_email, msg.as_string()) 
70              except Exception, e: 
71                  page.success = False 
72                  page.error = str(e) 
73                  page.inputs = cform 
74              else: 
75                  page.success = True 
76                  page.inputs = ContactForm() 
77              server.quit() 
78              print page 
79          else: 
80              page.inputs = cform 
81              print page

82
```

  


| Trees | Indices | Help | | MScanner | | --- | |
| --- | --- | --- | --- | --- |

|  |  |
| --- | --- |
| Generated by Epydoc 3.0beta1 on Fri Nov 23 09:13:24 2007 | http://epydoc.sourceforge.net |
