# Supplementary material for: MScanner: a classifier for retrieving Medline citations
Source: BMC Bioinformatics. 2008 Feb 19;9:108. doi: 10.1186/1471-2105-9-108 (PMC2263023; doi:10.1186/1471-2105-9-108)
Supplement: Additional file 3 — Source code for MScanner. mscanner-20071123.zip is a ZIP archive containing the Python 2.5 source code for MScanner, licensed under the GNU General Public License. It also contains API documentation in HTML format. Updated versions will be made available at . [file 1471-2105-9-108-S3.zip › mscanner/help/api/mscanner.htdocs.templates.front-pysrc.html]

xml version="1.0" encoding="ascii"?


mscanner.htdocs.templates.front


| Trees | Indices | Help | | MScanner | | --- | |
| --- | --- | --- | --- | --- |

|  |  |  |  |
| --- | --- | --- | --- |
| Package mscanner :: Package htdocs :: Package templates :: Module front | |  | | --- | | [hide private] | | [frames] | no frames] | |

# Source Code for Module mscanner.htdocs.templates.front

```
  1  #!/usr/bin/env python 
  2   
  3   
  4   
  5   
  6  ################################################## 
  7  ## DEPENDENCIES 
  8  import sys 
  9  import os 
 10  import os.path 
 11  from os.path import getmtime, exists 
 12  import time 
 13  import types 
 14  import __builtin__ 
 15  from Cheetah.Version import MinCompatibleVersion as RequiredCheetahVersion 
 16  from Cheetah.Version import MinCompatibleVersionTuple as RequiredCheetahVersionTuple 
 17  from Cheetah.Template import Template 
 18  from Cheetah.DummyTransaction import DummyTransaction 
 19  from Cheetah.NameMapper import NotFound, valueForName, valueFromSearchList, valueFromFrameOrSearchList 
 20  from Cheetah.CacheRegion import CacheRegion 
 21  import Cheetah.Filters as Filters 
 22  import Cheetah.ErrorCatchers as ErrorCatchers 
 23  from page import page 
 24   
 25  ################################################## 
 26  ## MODULE CONSTANTS 
 27  try: 
 28      True, False 
 29  except NameError: 
 30      True, False = (1==1), (1==0) 
 31  VFFSL=valueFromFrameOrSearchList 
 32  VFSL=valueFromSearchList 
 33  VFN=valueForName 
 34  currentTime=time.time 
 35  __CHEETAH_version__ = '2.0rc7' 
 36  __CHEETAH_versionTuple__ = (2, 0, 0, 'candidate', 7) 
 37  __CHEETAH_genTime__ = 1195588841.1489999 
 38  __CHEETAH_genTimestamp__ = 'Tue Nov 20 22:00:41 2007' 
 39  __CHEETAH_src__ = 'front.tmpl' 
 40  __CHEETAH_srcLastModified__ = 'Tue Nov 20 22:00:36 2007' 
 41  __CHEETAH_docstring__ = 'Autogenerated by CHEETAH: The Python-Powered Template Engine' 
 42   
 43  if __CHEETAH_versionTuple__ < RequiredCheetahVersionTuple: 
 44      raise AssertionError( 
 45        'This template was compiled with Cheetah version' 
 46        ' %s. Templates compiled before version %s must be recompiled.'%( 
 47           __CHEETAH_version__, RequiredCheetahVersion)) 
 48   
 49  ################################################## 
 50  ## CLASSES 
 51   


52 -class front(page):


53   
 54      ################################################## 
 55      ## CHEETAH GENERATED METHODS 
 56   
 57   


58 -    def __init__(self, *args, **KWs):


59   
 60          page.__init__(self, *args, **KWs) 
 61          if not self._CHEETAH__instanceInitialized: 
 62              cheetahKWArgs = {} 
 63              allowedKWs = 'searchList namespaces filter filtersLib errorCatcher'.split() 
 64              for k,v in KWs.items(): 
 65                  if k in allowedKWs: cheetahKWArgs[k] = v 
 66              self._initCheetahInstance(**cheetahKWArgs)

 67           
 68   


69 -    def title(self, **KWS):


70   
 71   
 72   
 73          ## CHEETAH: generated from #def title at line 3, col 1. 
 74          trans = KWS.get("trans") 
 75          if (not trans and not self._CHEETAH__isBuffering and not callable(self.transaction)): 
 76              trans = self.transaction # is None unless self.awake() was called 
 77          if not trans: 
 78              trans = DummyTransaction() 
 79              _dummyTrans = True 
 80          else: _dummyTrans = False 
 81          write = trans.response().write 
 82          SL = self._CHEETAH__searchList 
 83          _filter = self._CHEETAH__currentFilter 
 84           
 85          ######################################## 
 86          ## START - generated method body 
 87           
 88          write('MScanner\n') 
 89           
 90          ######################################## 
 91          ## END - generated method body 
 92           
 93          return _dummyTrans and trans.response().getvalue() or ""

 94           
 95   


96 -    def headertitle(self, **KWS):


97   
 98   
 99   
100          ## CHEETAH: generated from #def headertitle at line 7, col 1. 
101          trans = KWS.get("trans") 
102          if (not trans and not self._CHEETAH__isBuffering and not callable(self.transaction)): 
103              trans = self.transaction # is None unless self.awake() was called 
104          if not trans: 
105              trans = DummyTransaction() 
106              _dummyTrans = True 
107          else: _dummyTrans = False 
108          write = trans.response().write 
109          SL = self._CHEETAH__searchList 
110          _filter = self._CHEETAH__currentFilter 
111           
112          ######################################## 
113          ## START - generated method body 
114           
115          write('<h1>MScanner</h1><p style="margin:0">Retrieving Medline citations using examples</p>\n') 
116           
117          ######################################## 
118          ## END - generated method body 
119           
120          return _dummyTrans and trans.response().getvalue() or ""

121           
122   


123 -    def contents(self, **KWS):


124   
125   
126   
127          ## CHEETAH: generated from #def contents at line 11, col 1. 
128          trans = KWS.get("trans") 
129          if (not trans and not self._CHEETAH__isBuffering and not callable(self.transaction)): 
130              trans = self.transaction # is None unless self.awake() was called 
131          if not trans: 
132              trans = DummyTransaction() 
133              _dummyTrans = True 
134          else: _dummyTrans = False 
135          write = trans.response().write 
136          SL = self._CHEETAH__searchList 
137          _filter = self._CHEETAH__currentFilter 
138           
139          ######################################## 
140          ## START - generated method body 
141           
142          write('''<div class="narrow"> 
143   
144  <h2>Medline retrieval operation</h2> 
145   
146  <p>On the <a href="query">query</a> page, specify training examples  
147  of relevant articles as a list of PubMed IDs, and click "Submit".  
148  Help is available for the parameters, although the defaults should 
149  be fine for high-precision retrieval. Whole-medline classification  
150  takes about 90 seconds to complete.</p> 
151   
152  <p>See the <a href="static/sample/query/pg07">sample results</a> of a  
153  retrieval operation where the relevant examples were 1,595  
154  articles extracted from the Pharmacogenetics KnowledgeBase (<a 
155  href="http://www.pharmgkb.org">PharmGKB</a>).</p> 
156   
157   
158  <h2>Cross validation operation</h2>  
159   
160  <p>Classification performance depends on terms being enriched or depleted 
161  in the relevant articles relative to <a 
162  href="http://www.nlm.nih.gov/pubs/factsheets/medline.html">Medline</a>.   
163  Use cross validation to evaluate the Receiver Operating Characteristic 
164  and averaged precision against a set of random articles (small 
165  enough set to have very few relevant articles in it).</p> 
166   
167  <p>See the <a href="static/sample/valid/pg07">sample results</a> of 
168  10-fold cross validation of the pharmacogenetics articles against 100,000 
169  random articles in Medline.</p> 
170   
171   
172  <h2>Downloading the source code</h2> 
173   
174  <p>MScanner is released under the <a href="http://www.gnu.org/licenses"> GNU 
175  General Public License</a>. The latest revision is 2007.11.20. Download the <a 
176  href="static/source-20071120.zip">source archive</a>.</p> 
177   
178   
179  <h2>Sample training data</h2> 
180   
181  <p>Below are the results using the sample training corpora 
182  described in the MScanner paper.</p> 
183   
184  <table> 
185  <tr> 
186  <th>Training data</th> 
187  <th>Results</th> 
188  <th>Results</th> 
189  </tr> 
190  <tr> 
191  <td>PG07 (Pharmacogenetics, 1595 citations)</td> 
192  <td><a href="static/sample/query/pg07">retrieval</a> 
193  <td><a href="static/sample/valid/pg07">validation</a></td> 
194  </tr> 
195  <tr> 
196  <td>AIDSBio (AIDS and Bioethics, 10732 citations)</td> 
197  <td><a href="static/sample/query/aidsbio">retrieval</a></td> 
198  <td><a href="static/sample/valid/aidsbio">validation</a></td> 
199  </tr> 
200  <tr> 
201  <td>Radiology (splenic imaging, 67 citations)</td> 
202  <td><a href="static/sample/query/radiology">retrieval</a></td> 
203  <td><a href="static/sample/valid/radiology">validation</a></td> 
204  </tr> 
205  <tr> 
206  <td>Control (randomly selected, 10000 citations)</td> 
207  <td></td> 
208  <td><a href="static/sample/valid/control">validation</a></td> 
209  </tr> 
210  </table> 
211   
212  <h3>Browser compatibility</h3> 
213   
214  <p> 
215  This web interface has been tested with 
216  <a href="http://www.mozilla.com/firefox/">Mozilla Firefox 2</a>,  
217  <a href="http://www.microsoft.com/windows/downloads/ie/getitnow.mspx">Internet Explorer 7</a>,  
218  <a href="http://www.apple.com/safari/download/">Safari 3</a> and  
219  <a href="http://www.opera.com/download/">Opera 9</a>.   
220  Some features may not work in older browsers or with JavaScript disabled. 
221  </p> 
222   
223  </div><!--class=narrow--> 
224  ''') 
225           
226          ######################################## 
227          ## END - generated method body 
228           
229          return _dummyTrans and trans.response().getvalue() or ""

230           
231   


232 -    def writeBody(self, **KWS):


233   
234   
235   
236          ## CHEETAH: main method generated for this template 
237          trans = KWS.get("trans") 
238          if (not trans and not self._CHEETAH__isBuffering and not callable(self.transaction)): 
239              trans = self.transaction # is None unless self.awake() was called 
240          if not trans: 
241              trans = DummyTransaction() 
242              _dummyTrans = True 
243          else: _dummyTrans = False 
244          write = trans.response().write 
245          SL = self._CHEETAH__searchList 
246          _filter = self._CHEETAH__currentFilter 
247           
248          ######################################## 
249          ## START - generated method body 
250           
251          write(''' 
252   
253   
254  ''') 
255           
256          ######################################## 
257          ## END - generated method body 
258           
259          return _dummyTrans and trans.response().getvalue() or ""

260           
261      ################################################## 
262      ## CHEETAH GENERATED ATTRIBUTES 
263   
264   
265      _CHEETAH__instanceInitialized = False 
266   
267      _CHEETAH_version = __CHEETAH_version__ 
268   
269      _CHEETAH_versionTuple = __CHEETAH_versionTuple__ 
270   
271      _CHEETAH_genTime = __CHEETAH_genTime__ 
272   
273      _CHEETAH_genTimestamp = __CHEETAH_genTimestamp__ 
274   
275      _CHEETAH_src = __CHEETAH_src__ 
276   
277      _CHEETAH_srcLastModified = __CHEETAH_srcLastModified__ 
278   
279      _mainCheetahMethod_for_front= 'writeBody'

280   
281  ## END CLASS DEFINITION 
282   
283  if not hasattr(front, '_initCheetahAttributes'): 
284      templateAPIClass = getattr(front, '_CHEETAH_templateClass', Template) 
285      templateAPIClass._addCheetahPlumbingCodeToClass(front) 
286   
287   
288  # CHEETAH was developed by Tavis Rudd and Mike Orr 
289  # with code, advice and input from many other volunteers. 
290  # For more information visit http://www.CheetahTemplate.org/ 
291   
292  ################################################## 
293  ## if run from command line: 
294  if __name__ == '__main__': 
295      from Cheetah.TemplateCmdLineIface import CmdLineIface 
296      CmdLineIface(templateObj=front()).run() 
297
```

  


| Trees | Indices | Help | | MScanner | | --- | |
| --- | --- | --- | --- | --- |

|  |  |
| --- | --- |
| Generated by Epydoc 3.0beta1 on Fri Nov 23 09:13:24 2007 | http://epydoc.sourceforge.net |
