# Supplementary material for: MScanner: a classifier for retrieving Medline citations
Source: BMC Bioinformatics. 2008 Feb 19;9:108. doi: 10.1186/1471-2105-9-108 (PMC2263023; doi:10.1186/1471-2105-9-108)
Supplement: Additional file 3 — Source code for MScanner. mscanner-20071123.zip is a ZIP archive containing the Python 2.5 source code for MScanner, licensed under the GNU General Public License. It also contains API documentation in HTML format. Updated versions will be made available at . [file 1471-2105-9-108-S3.zip › mscanner/help/api/mscanner.htdocs.templates.output-pysrc.html]

xml version="1.0" encoding="ascii"?


mscanner.htdocs.templates.output


| Trees | Indices | Help | | MScanner | | --- | |
| --- | --- | --- | --- | --- |

|  |  |  |  |
| --- | --- | --- | --- |
| Package mscanner :: Package htdocs :: Package templates :: Module output | |  | | --- | | [hide private] | | [frames] | no frames] | |

# Source Code for Module mscanner.htdocs.templates.output

```
  1  #!/usr/bin/env python 
  2   
  3   
  4   
  5   
  6  ################################################## 
  7  ## DEPENDENCIES 
  8  import sys 
  9  import os 
 10  import os.path 
 11  from os.path import getmtime, exists 
 12  import time 
 13  import types 
 14  import __builtin__ 
 15  from Cheetah.Version import MinCompatibleVersion as RequiredCheetahVersion 
 16  from Cheetah.Version import MinCompatibleVersionTuple as RequiredCheetahVersionTuple 
 17  from Cheetah.Template import Template 
 18  from Cheetah.DummyTransaction import DummyTransaction 
 19  from Cheetah.NameMapper import NotFound, valueForName, valueFromSearchList, valueFromFrameOrSearchList 
 20  from Cheetah.CacheRegion import CacheRegion 
 21  import Cheetah.Filters as Filters 
 22  import Cheetah.ErrorCatchers as ErrorCatchers 
 23  from page import page 
 24  import time 
 25   
 26  ################################################## 
 27  ## MODULE CONSTANTS 
 28  try: 
 29      True, False 
 30  except NameError: 
 31      True, False = (1==1), (1==0) 
 32  VFFSL=valueFromFrameOrSearchList 
 33  VFSL=valueFromSearchList 
 34  VFN=valueForName 
 35  currentTime=time.time 
 36  __CHEETAH_version__ = '2.0rc7' 
 37  __CHEETAH_versionTuple__ = (2, 0, 0, 'candidate', 7) 
 38  __CHEETAH_genTime__ = 1193401029.1170001 
 39  __CHEETAH_genTimestamp__ = 'Fri Oct 26 14:17:09 2007' 
 40  __CHEETAH_src__ = 'output.tmpl' 
 41  __CHEETAH_srcLastModified__ = 'Fri Oct 26 14:17:08 2007' 
 42  __CHEETAH_docstring__ = 'Autogenerated by CHEETAH: The Python-Powered Template Engine' 
 43   
 44  if __CHEETAH_versionTuple__ < RequiredCheetahVersionTuple: 
 45      raise AssertionError( 
 46        'This template was compiled with Cheetah version' 
 47        ' %s. Templates compiled before version %s must be recompiled.'%( 
 48           __CHEETAH_version__, RequiredCheetahVersion)) 
 49   
 50  ################################################## 
 51  ## CLASSES 
 52   


53 -class output(page):


54   
 55      ################################################## 
 56      ## CHEETAH GENERATED METHODS 
 57   
 58   


59 -    def __init__(self, *args, **KWs):


60   
 61          page.__init__(self, *args, **KWs) 
 62          if not self._CHEETAH__instanceInitialized: 
 63              cheetahKWArgs = {} 
 64              allowedKWs = 'searchList namespaces filter filtersLib errorCatcher'.split() 
 65              for k,v in KWs.items(): 
 66                  if k in allowedKWs: cheetahKWArgs[k] = v 
 67              self._initCheetahInstance(**cheetahKWArgs)

 68           
 69   


70 -    def title(self, **KWS):


71   
 72   
 73   
 74          ## CHEETAH: generated from #def title at line 13, col 1. 
 75          trans = KWS.get("trans") 
 76          if (not trans and not self._CHEETAH__isBuffering and not callable(self.transaction)): 
 77              trans = self.transaction # is None unless self.awake() was called 
 78          if not trans: 
 79              trans = DummyTransaction() 
 80              _dummyTrans = True 
 81          else: _dummyTrans = False 
 82          write = trans.response().write 
 83          SL = self._CHEETAH__searchList 
 84          _filter = self._CHEETAH__currentFilter 
 85           
 86          ######################################## 
 87          ## START - generated method body 
 88           
 89          write('MScanner Output\n') 
 90           
 91          ######################################## 
 92          ## END - generated method body 
 93           
 94          return _dummyTrans and trans.response().getvalue() or ""

 95           
 96   


97 -    def contents(self, **KWS):


98   
 99   
100   
101          ## CHEETAH: generated from #def contents at line 17, col 1. 
102          trans = KWS.get("trans") 
103          if (not trans and not self._CHEETAH__isBuffering and not callable(self.transaction)): 
104              trans = self.transaction # is None unless self.awake() was called 
105          if not trans: 
106              trans = DummyTransaction() 
107              _dummyTrans = True 
108          else: _dummyTrans = False 
109          write = trans.response().write 
110          SL = self._CHEETAH__searchList 
111          _filter = self._CHEETAH__currentFilter 
112           
113          ######################################## 
114          ## START - generated method body 
115           
116          write('\n\n') 
117          #  Print errors if any 
118          _v = VFSL([locals()]+SL+[globals(), __builtin__],"getVar",False)("errors", "") # '$getVar("errors", "")' on line 22, col 1 
119          if _v is not None: write(_filter(_v, rawExpr='$getVar("errors", "")')) # from line 22, col 1. 
120          write(''' 
121   
122  <div class="narrow"> 
123   
124  ''') 
125          if VFSL([locals()]+SL+[globals(), __builtin__],"getVar",False)("target", None) is not None: # generated from line 26, col 1 
126              write('\n') 
127              if VFSL([locals()]+SL+[globals(), __builtin__],"getVar",False)("delete_error", None) is not None: # generated from line 28, col 3 
128                  write('    <p>Cannot delete <q>') 
129                  _v = VFSL([locals()]+SL+[globals(), __builtin__],"target",True) # '$target' on line 29, col 25 
130                  if _v is not None: write(_filter(_v, rawExpr='$target')) # from line 29, col 25. 
131                  write('</q>: ') 
132                  _v = VFSL([locals()]+SL+[globals(), __builtin__],"delete_error",True) # '$delete_error' on line 29, col 38 
133                  if _v is not None: write(_filter(_v, rawExpr='$delete_error')) # from line 29, col 38. 
134                  write('</p>\n') 
135              else: # generated from line 30, col 3 
136                  write('    <p>Task <q>') 
137                  _v = VFSL([locals()]+SL+[globals(), __builtin__],"target",True) # '$target' on line 31, col 16 
138                  if _v is not None: write(_filter(_v, rawExpr='$target')) # from line 31, col 16. 
139                  write('</q> has been deleted.</p>\n') 
140              write('\n') 
141              if VFSL([locals()]+SL+[globals(), __builtin__],"getVar",False)("download_error", None) is not None: # generated from line 34, col 3 
142                  write('    <p>Cannot download <q>') 
143                  _v = VFSL([locals()]+SL+[globals(), __builtin__],"target",True) # '$target' on line 35, col 27 
144                  if _v is not None: write(_filter(_v, rawExpr='$target')) # from line 35, col 27. 
145                  write('</q>: ') 
146                  _v = VFSL([locals()]+SL+[globals(), __builtin__],"download_error",True) # '$download_error' on line 35, col 40 
147                  if _v is not None: write(_filter(_v, rawExpr='$download_error')) # from line 35, col 40. 
148                  write('\n') 
149              write('  \n') 
150          write('\n<p>Outputs for ') 
151          _v = VFSL([locals()]+SL+[globals(), __builtin__],"len",False)(VFSL([locals()]+SL+[globals(), __builtin__],"queue.donelist",True)) # '$len($queue.donelist)' on line 40, col 16 
152          if _v is not None: write(_filter(_v, rawExpr='$len($queue.donelist)')) # from line 40, col 16. 
153          write(''' tasks follow. Queries for which the "hide" box 
154  was ticked are not listed.</p> 
155   
156  </div><!--class=narrow--> 
157   
158  <form id="output" action="" method="post"> 
159   
160    <table> 
161    <thead> 
162      <tr> 
163        <th>Selection</th> 
164        <th>Timestamp</th> 
165        <th>Operation</th> 
166        <th>Link to Output</th> 
167      </tr> 
168    </thead> 
169    <tbody> 
170  ''') 
171          for d in VFSL([locals()]+SL+[globals(), __builtin__],"queue.donelist",True): # generated from line 57, col 5 
172              write('    <tr>\n      <td class="del">\n        <input type="radio" name="dataset" value="') 
173              _v = VFSL([locals()]+SL+[globals(), __builtin__],"d.dataset",True) # '$d.dataset' on line 60, col 51 
174              if _v is not None: write(_filter(_v, rawExpr='$d.dataset')) # from line 60, col 51. 
175              write('''"> 
176        </td> 
177        <td class="timestamp"> 
178          ''') 
179              _v = VFN(VFSL([locals()]+SL+[globals(), __builtin__],"time",True),"strftime",False)("%Y/%m/%d %H:%M:%S GMT", VFN(VFSL([locals()]+SL+[globals(), __builtin__],"time",True),"gmtime",False)(VFSL([locals()]+SL+[globals(), __builtin__],"d.submitted",True))) # '$time.strftime("%Y/%m/%d %H:%M:%S GMT", $time.gmtime($d.submitted))' on line 63, col 9 
180              if _v is not None: write(_filter(_v, rawExpr='$time.strftime("%Y/%m/%d %H:%M:%S GMT", $time.gmtime($d.submitted))')) # from line 63, col 9. 
181              write(''' 
182        </td> 
183        <td class="operation"> 
184          ''') 
185              _v = VFSL([locals()]+SL+[globals(), __builtin__],"d.operation",True) # '$d.operation' on line 66, col 9 
186              if _v is not None: write(_filter(_v, rawExpr='$d.operation')) # from line 66, col 9. 
187              write(''' 
188        </td> 
189        <td class="dataset"> 
190          <a href="/static/output/''') 
191              _v = VFSL([locals()]+SL+[globals(), __builtin__],"d.dataset",True) # '$d.dataset' on line 69, col 33 
192              if _v is not None: write(_filter(_v, rawExpr='$d.dataset')) # from line 69, col 33. 
193              write('/">') 
194              _v = VFSL([locals()]+SL+[globals(), __builtin__],"d.dataset",True) # '$d.dataset' on line 69, col 46 
195              if _v is not None: write(_filter(_v, rawExpr='$d.dataset')) # from line 69, col 46. 
196              write('''</a> 
197        </td> 
198      </tr> 
199  ''') 
200          write('''  </tbody> 
201    </table> 
202     
203    <div class="narrow"> 
204      <p> 
205        Download selected as a zip file (0.5-2 megabytes,  
206        <input type="checkbox" name="omit_mesh" checked="checked"> omit term list to save 300k): 
207        <button type="submit" name="operation" value="download">download</button> 
208      </p> 
209      <p> 
210        Delete selected using deletion code:  
211        <input name="delcode" type="text" size="10"> 
212        <button type="submit" name="operation" value="delete">delete</button> 
213      </p> 
214    </div> 
215   
216  </form> 
217   
218  ''') 
219           
220          ######################################## 
221          ## END - generated method body 
222           
223          return _dummyTrans and trans.response().getvalue() or ""

224           
225   


226 -    def writeBody(self, **KWS):


227   
228   
229   
230          ## CHEETAH: main method generated for this template 
231          trans = KWS.get("trans") 
232          if (not trans and not self._CHEETAH__isBuffering and not callable(self.transaction)): 
233              trans = self.transaction # is None unless self.awake() was called 
234          if not trans: 
235              trans = DummyTransaction() 
236              _dummyTrans = True 
237          else: _dummyTrans = False 
238          write = trans.response().write 
239          SL = self._CHEETAH__searchList 
240          _filter = self._CHEETAH__currentFilter 
241           
242          ######################################## 
243          ## START - generated method body 
244           
245          write('\n') 
246          #  PARAMETERS 
247          #  
248          # $queue -- QueueStatus object where we get the list of done tasks 
249          # $errors -- Optional validation errors in the form 
250          # $target -- Optional target of delete/download op 
251          # $delete_errror -- Optional reason for being unable to delete $target 
252          # $download_errror -- Optional reason for being unable to download $target 
253          #  
254          write('\n\n') 
255           
256          ######################################## 
257          ## END - generated method body 
258           
259          return _dummyTrans and trans.response().getvalue() or ""

260           
261      ################################################## 
262      ## CHEETAH GENERATED ATTRIBUTES 
263   
264   
265      _CHEETAH__instanceInitialized = False 
266   
267      _CHEETAH_version = __CHEETAH_version__ 
268   
269      _CHEETAH_versionTuple = __CHEETAH_versionTuple__ 
270   
271      _CHEETAH_genTime = __CHEETAH_genTime__ 
272   
273      _CHEETAH_genTimestamp = __CHEETAH_genTimestamp__ 
274   
275      _CHEETAH_src = __CHEETAH_src__ 
276   
277      _CHEETAH_srcLastModified = __CHEETAH_srcLastModified__ 
278   
279      _mainCheetahMethod_for_output= 'writeBody'

280   
281  ## END CLASS DEFINITION 
282   
283  if not hasattr(output, '_initCheetahAttributes'): 
284      templateAPIClass = getattr(output, '_CHEETAH_templateClass', Template) 
285      templateAPIClass._addCheetahPlumbingCodeToClass(output) 
286   
287   
288  # CHEETAH was developed by Tavis Rudd and Mike Orr 
289  # with code, advice and input from many other volunteers. 
290  # For more information visit http://www.CheetahTemplate.org/ 
291   
292  ################################################## 
293  ## if run from command line: 
294  if __name__ == '__main__': 
295      from Cheetah.TemplateCmdLineIface import CmdLineIface 
296      CmdLineIface(templateObj=output()).run() 
297
```

  


| Trees | Indices | Help | | MScanner | | --- | |
| --- | --- | --- | --- | --- |

|  |  |
| --- | --- |
| Generated by Epydoc 3.0beta1 on Fri Nov 23 09:13:24 2007 | http://epydoc.sourceforge.net |
