# Supplementary material for: MScanner: a classifier for retrieving Medline citations
Source: BMC Bioinformatics. 2008 Feb 19;9:108. doi: 10.1186/1471-2105-9-108 (PMC2263023; doi:10.1186/1471-2105-9-108)
Supplement: Additional file 3 — Source code for MScanner. mscanner-20071123.zip is a ZIP archive containing the Python 2.5 source code for MScanner, licensed under the GNU General Public License. It also contains API documentation in HTML format. Updated versions will be made available at . [file 1471-2105-9-108-S3.zip › mscanner/help/api/mscanner.htdocs.templates.output_logic-module.html]

xml version="1.0" encoding="ascii"?


mscanner.htdocs.templates.output\_logic


| Trees | Indices | Help | | MScanner | | --- | |
| --- | --- | --- | --- | --- |

|  |  |  |  |
| --- | --- | --- | --- |
| Package mscanner :: Package htdocs :: Package templates :: Module output\_logic | |  | | --- | | [hide private] | | [frames] | no frames] | |

# Module output\_logic

source code  
  
web.py handler for the output listing page  
  


---

**Author:**
Graham Poulter <http://graham.poulter.googlepages.com>

**Copyright:**
2007 Graham Poulter

**License:**
GPL


|  |  |  |  |
| --- | --- | --- | --- |
| |  |  | | --- | --- | | Classes | [hide private] | | |
|  | OutputPage  Page linking to outputs |


|  |  |  |  |
| --- | --- | --- | --- |
| |  |  | | --- | --- | | Variables | [hide private] | | |
|  | OutputForm = `forms.Form(forms.Hidden("operation", forms.Valida...`  Structure for the form on the outputs page |


|  |  |  |  |
| --- | --- | --- | --- |
| |  |  | | --- | --- | | Variables Details | [hide private] | | |

|  |  |
| --- | --- |
| OutputFormStructure for the form on the outputs page   Value:  |  | | --- | | ``` forms.Form(forms.Hidden("operation", forms.Validator(lambda x: x in [" download", "delete"], "Invalid op")), forms.Checkbox("omit_mesh", form s.checkbox_validator), forms.Hidden("dataset", query_logic.dataset_val idator), forms.Hidden("delcode", query_logic.delcode_validator),) ``` | |

  


| Trees | Indices | Help | | MScanner | | --- | |
| --- | --- | --- | --- | --- |

|  |  |
| --- | --- |
| Generated by Epydoc 3.0beta1 on Fri Nov 23 09:13:20 2007 | http://epydoc.sourceforge.net |
