# Supplementary material for: MScanner: a classifier for retrieving Medline citations
Source: BMC Bioinformatics. 2008 Feb 19;9:108. doi: 10.1186/1471-2105-9-108 (PMC2263023; doi:10.1186/1471-2105-9-108)
Supplement: Additional file 3 — Source code for MScanner. mscanner-20071123.zip is a ZIP archive containing the Python 2.5 source code for MScanner, licensed under the GNU General Public License. It also contains API documentation in HTML format. Updated versions will be made available at . [file 1471-2105-9-108-S3.zip › mscanner/help/api/mscanner.htdocs.templates.output_logic-pysrc.html]

xml version="1.0" encoding="ascii"?


mscanner.htdocs.templates.output\_logic


| Trees | Indices | Help | | MScanner | | --- | |
| --- | --- | --- | --- | --- |

|  |  |  |  |
| --- | --- | --- | --- |
| Package mscanner :: Package htdocs :: Package templates :: Module output\_logic | |  | | --- | | [hide private] | | [frames] | no frames] | |

# Source Code for Module mscanner.htdocs.templates.output\_logic

```
  1  """web.py handler for the output listing page""" 
  2   
  3  __copyright__ = "2007 Graham Poulter" 
  4  __author__ = "Graham Poulter <http://graham.poulter.googlepages.com>" 
  5  __license__ = "GPL" 
  6   
  7  import web 
  8  import md5 
  9   
 10  import output, query_logic 
 11  from mscanner.htdocs import forms, queue 
 12  from mscanner.configuration import rc 
 13   
 14   
 15  OutputForm = forms.Form( 
 16      forms.Hidden( 
 17          "operation", 
 18          forms.Validator(lambda x: x in ["download", "delete"], "Invalid op")), 
 19       
 20      forms.Checkbox( 
 21          "omit_mesh", 
 22          forms.checkbox_validator), 
 23       
 24      forms.Hidden( 
 25          "dataset", 
 26          query_logic.dataset_validator), 
 27       
 28      forms.Hidden( 
 29          "delcode", 
 30          query_logic.delcode_validator), 
 31  ) 
 32  """Structure for the form on the outputs page""" 
 33   
 34   
 35   


36 -class OutputPage:


37      """Page linking to outputs""" 
 38       


39 -    def print_page(self, page):


40          """Add the final version of the queue and output the page""" 
 41          page.queue = queue.QueueStatus() 
 42          page.visible = [ d for d in page.queue.donelist  
 43              if "hidden" not in d or d.hidden == False ] 
 44          print page

 45   
 46   


47 -    def GET(self):


48          """Just list the available output directories""" 
 49          web.header('Content-Type', 'text/html; charset=utf-8')  
 50          page = output.output() 
 51          self.print_page(page)

 52           
 53           


54 -    def POST(self):


55          """Attempt to download or delete one of the outputs""" 
 56          web.header('Content-Type', 'text/html; charset=utf-8')  
 57          page = output.output() 
 58          oform = OutputForm() 
 59           
 60          # Errors in the form 
 61          if not oform.validates(web.input()): 
 62              e = ["<li>%s: %s</li>\n" % (n,e) for n,e in  
 63                   oform.errors.iteritems() if e is not None] 
 64              page.errors = "".join(["<p>Errors</p><ul>\n"]+e+["</ul>\n"]) 
 65              self.print_page(page) 
 66              return 
 67           
 68          # The thing we are trying to operate on 
 69          page.target = oform.d.dataset  
 70           
 71          # Deleting something 
 72          if oform.d.operation == "delete": 
 73              target = page.target 
 74              q = queue.QueueStatus() 
 75              if target not in q: 
 76                  page.delete_error = "there is no task with that name." 
 77              elif q.status[target] == q.RUNNING: 
 78                  page.delete_error = "MScanner is busy with the task." 
 79              elif q.status[target] in [q.WAITING, q.DONE]: 
 80                  md5code = md5.new(oform.d.delcode).hexdigest() 
 81                  if "delcode" in q[target] and md5code != q[target].delcode: 
 82                      page.delete_error = "incorrect deletion code." 
 83                  else: 
 84                      if q.status[target] == q.DONE: 
 85                          try: 
 86                              queue.delete_output(target) 
 87                          except OSError, e: 
 88                              page.delete_error = str(e) 
 89                      elif q.status[target] == q.WAITING: 
 90                          try: 
 91                              q[target]._filename.remove() 
 92                          except OSError, e: 
 93                              page.delete_error = str(e) 
 94              self.print_page(page) 
 95           
 96          # Save the output directory as a zip file for download 
 97          elif oform.d.operation == "download": 
 98              q = queue.QueueStatus() 
 99              if page.target not in q or q.status[page.target] is not q.DONE: 
100                  page.download_error = "Specified output is not available" 
101                  self.print_page(page) 
102              else: 
103                  outdir = rc.web_report_dir / page.target 
104                  outfile = outdir / (page.target + ".zip") 
105                  if not outfile.exists(): 
106                      from zipfile import ZipFile, ZIP_DEFLATED 
107                      zf = ZipFile(str(outfile), "w", ZIP_DEFLATED) 
108                      for fpath in outdir.files(): 
109                          # Omit existing zip files 
110                          if fpath.endswith(".zip"): 
111                              continue 
112                          # Omit MeSH terms if the user requests it 
113                          if fpath.basename() == rc.report_term_scores: 
114                              if forms.ischecked(oform.d.omit_mesh): 
115                                  continue 
116                          # Omit all-in-one result file 
117                          if fpath.basename() == rc.report_result_all: 
118                              continue 
119                          zf.write(str(fpath), str(fpath.basename())) 
120                      zf.close() 
121                      outfile.chmod(0777) 
122                  ds = web.urlquote(page.target) 
123                  web.seeother("static/output/" + ds + "/" + ds + ".zip")

124
```

  


| Trees | Indices | Help | | MScanner | | --- | |
| --- | --- | --- | --- | --- |

|  |  |
| --- | --- |
| Generated by Epydoc 3.0beta1 on Fri Nov 23 09:13:22 2007 | http://epydoc.sourceforge.net |
