# Supplementary material for: MScanner: a classifier for retrieving Medline citations
Source: BMC Bioinformatics. 2008 Feb 19;9:108. doi: 10.1186/1471-2105-9-108 (PMC2263023; doi:10.1186/1471-2105-9-108)
Supplement: Additional file 3 — Source code for MScanner. mscanner-20071123.zip is a ZIP archive containing the Python 2.5 source code for MScanner, licensed under the GNU General Public License. It also contains API documentation in HTML format. Updated versions will be made available at . [file 1471-2105-9-108-S3.zip › mscanner/help/api/mscanner.htdocs.templates.output_logic.OutputPage-class.html]

xml version="1.0" encoding="ascii"?


mscanner.htdocs.templates.output\_logic.OutputPage


| Trees | Indices | Help | | MScanner | | --- | |
| --- | --- | --- | --- | --- |

|  |  |  |  |
| --- | --- | --- | --- |
| Package mscanner :: Package htdocs :: Package templates :: Module output\_logic :: Class OutputPage | |  | | --- | | [hide private] | | [frames] | no frames] | |

# Class OutputPage

source code  
  
Page linking to outputs  
  


|  |  |  |  |
| --- | --- | --- | --- |
| |  |  | | --- | --- | | Instance Methods | [hide private] | | |
|  | |  |  | | --- | --- | | print\_page(self, page)  Add the final version of the queue and output the page | source code | |
|  | |  |  | | --- | --- | | GET(self)  Just list the available output directories | source code | |
|  | |  |  | | --- | --- | | POST(self)  Attempt to download or delete one of the outputs | source code | |

| Trees | Indices | Help | | MScanner | | --- | |
| --- | --- | --- | --- | --- |

|  |  |
| --- | --- |
| Generated by Epydoc 3.0beta1 on Fri Nov 23 09:13:21 2007 | http://epydoc.sourceforge.net |
