# Supplementary material for: MScanner: a classifier for retrieving Medline citations
Source: BMC Bioinformatics. 2008 Feb 19;9:108. doi: 10.1186/1471-2105-9-108 (PMC2263023; doi:10.1186/1471-2105-9-108)
Supplement: Additional file 3 — Source code for MScanner. mscanner-20071123.zip is a ZIP archive containing the Python 2.5 source code for MScanner, licensed under the GNU General Public License. It also contains API documentation in HTML format. Updated versions will be made available at . [file 1471-2105-9-108-S3.zip › mscanner/help/api/mscanner.htdocs.templates.page-pysrc.html]

xml version="1.0" encoding="ascii"?


mscanner.htdocs.templates.page


| Trees | Indices | Help | | MScanner | | --- | |
| --- | --- | --- | --- | --- |

|  |  |  |  |
| --- | --- | --- | --- |
| Package mscanner :: Package htdocs :: Package templates :: Module page | |  | | --- | | [hide private] | | [frames] | no frames] | |

# Source Code for Module mscanner.htdocs.templates.page

```
  1  #!/usr/bin/env python 
  2   
  3   
  4   
  5   
  6  ################################################## 
  7  ## DEPENDENCIES 
  8  import sys 
  9  import os 
 10  import os.path 
 11  from os.path import getmtime, exists 
 12  import time 
 13  import types 
 14  import __builtin__ 
 15  from Cheetah.Version import MinCompatibleVersion as RequiredCheetahVersion 
 16  from Cheetah.Version import MinCompatibleVersionTuple as RequiredCheetahVersionTuple 
 17  from Cheetah.Template import Template 
 18  from Cheetah.DummyTransaction import DummyTransaction 
 19  from Cheetah.NameMapper import NotFound, valueForName, valueFromSearchList, valueFromFrameOrSearchList 
 20  from Cheetah.CacheRegion import CacheRegion 
 21  import Cheetah.Filters as Filters 
 22  import Cheetah.ErrorCatchers as ErrorCatchers 
 23  import time 
 24   
 25  ################################################## 
 26  ## MODULE CONSTANTS 
 27  try: 
 28      True, False 
 29  except NameError: 
 30      True, False = (1==1), (1==0) 
 31  VFFSL=valueFromFrameOrSearchList 
 32  VFSL=valueFromSearchList 
 33  VFN=valueForName 
 34  currentTime=time.time 
 35  __CHEETAH_version__ = '2.0rc7' 
 36  __CHEETAH_versionTuple__ = (2, 0, 0, 'candidate', 7) 
 37  __CHEETAH_genTime__ = 1193401027.9920001 
 38  __CHEETAH_genTimestamp__ = 'Fri Oct 26 14:17:07 2007' 
 39  __CHEETAH_src__ = 'page.tmpl' 
 40  __CHEETAH_srcLastModified__ = 'Fri Oct 26 14:17:08 2007' 
 41  __CHEETAH_docstring__ = 'Autogenerated by CHEETAH: The Python-Powered Template Engine' 
 42   
 43  if __CHEETAH_versionTuple__ < RequiredCheetahVersionTuple: 
 44      raise AssertionError( 
 45        'This template was compiled with Cheetah version' 
 46        ' %s. Templates compiled before version %s must be recompiled.'%( 
 47           __CHEETAH_version__, RequiredCheetahVersion)) 
 48   
 49  ################################################## 
 50  ## CLASSES 
 51   


52 -class page(Template):


53   
 54      ################################################## 
 55      ## CHEETAH GENERATED METHODS 
 56   
 57   


58 -    def __init__(self, *args, **KWs):


59   
 60          Template.__init__(self, *args, **KWs) 
 61          if not self._CHEETAH__instanceInitialized: 
 62              cheetahKWArgs = {} 
 63              allowedKWs = 'searchList namespaces filter filtersLib errorCatcher'.split() 
 64              for k,v in KWs.items(): 
 65                  if k in allowedKWs: cheetahKWArgs[k] = v 
 66              self._initCheetahInstance(**cheetahKWArgs)

 67           
 68   


69 -    def doctype(self, **KWS):


70   
 71   
 72   
 73          ## CHEETAH: generated from #block doctype at line 1, col 1. 
 74          trans = KWS.get("trans") 
 75          if (not trans and not self._CHEETAH__isBuffering and not callable(self.transaction)): 
 76              trans = self.transaction # is None unless self.awake() was called 
 77          if not trans: 
 78              trans = DummyTransaction() 
 79              _dummyTrans = True 
 80          else: _dummyTrans = False 
 81          write = trans.response().write 
 82          SL = self._CHEETAH__searchList 
 83          _filter = self._CHEETAH__currentFilter 
 84           
 85          ######################################## 
 86          ## START - generated method body 
 87           
 88          write('<!DOCTYPE HTML PUBLIC "-//W3C//DTD HTML 4.01//EN" "http://www.w3.org/TR/html4/strict.dtd">\n') 
 89           
 90          ######################################## 
 91          ## END - generated method body 
 92           
 93          return _dummyTrans and trans.response().getvalue() or ""

 94           
 95   


96 -    def title(self, **KWS):


97   
 98   
 99   
100          ## CHEETAH: generated from #def title at line 8, col 1. 
101          trans = KWS.get("trans") 
102          if (not trans and not self._CHEETAH__isBuffering and not callable(self.transaction)): 
103              trans = self.transaction # is None unless self.awake() was called 
104          if not trans: 
105              trans = DummyTransaction() 
106              _dummyTrans = True 
107          else: _dummyTrans = False 
108          write = trans.response().write 
109          SL = self._CHEETAH__searchList 
110          _filter = self._CHEETAH__currentFilter 
111           
112          ######################################## 
113          ## START - generated method body 
114           
115          write('Sample Title\n') 
116           
117          ######################################## 
118          ## END - generated method body 
119           
120          return _dummyTrans and trans.response().getvalue() or ""

121           
122   


123 -    def wintitle(self, **KWS):


124   
125   
126   
127          ## CHEETAH: generated from #block wintitle at line 18, col 1. 
128          trans = KWS.get("trans") 
129          if (not trans and not self._CHEETAH__isBuffering and not callable(self.transaction)): 
130              trans = self.transaction # is None unless self.awake() was called 
131          if not trans: 
132              trans = DummyTransaction() 
133              _dummyTrans = True 
134          else: _dummyTrans = False 
135          write = trans.response().write 
136          SL = self._CHEETAH__searchList 
137          _filter = self._CHEETAH__currentFilter 
138           
139          ######################################## 
140          ## START - generated method body 
141           
142          write('<title>') 
143          _v = VFSL([locals()]+SL+[globals(), __builtin__],"title",True) # '$title' on line 19, col 8 
144          if _v is not None: write(_filter(_v, rawExpr='$title')) # from line 19, col 8. 
145          write('</title>\n') 
146           
147          ######################################## 
148          ## END - generated method body 
149           
150          return _dummyTrans and trans.response().getvalue() or ""

151           
152   


153 -    def stdheaders(self, **KWS):


154   
155   
156   
157          ## CHEETAH: generated from #block stdheaders at line 22, col 1. 
158          trans = KWS.get("trans") 
159          if (not trans and not self._CHEETAH__isBuffering and not callable(self.transaction)): 
160              trans = self.transaction # is None unless self.awake() was called 
161          if not trans: 
162              trans = DummyTransaction() 
163              _dummyTrans = True 
164          else: _dummyTrans = False 
165          write = trans.response().write 
166          SL = self._CHEETAH__searchList 
167          _filter = self._CHEETAH__currentFilter 
168           
169          ######################################## 
170          ## START - generated method body 
171           
172          write('<link rel="stylesheet" type="text/css" href="') 
173          _v = VFSL([locals()]+SL+[globals(), __builtin__],"base",True) # '$base' on line 23, col 46 
174          if _v is not None: write(_filter(_v, rawExpr='$base')) # from line 23, col 46. 
175          write('/static/mscanner.css">\n<link rel="shortcut icon" href="') 
176          _v = VFSL([locals()]+SL+[globals(), __builtin__],"base",True) # '$base' on line 24, col 33 
177          if _v is not None: write(_filter(_v, rawExpr='$base')) # from line 24, col 33. 
178          write('/favicon.png" type="image/x-icon">\n') 
179           
180          ######################################## 
181          ## END - generated method body 
182           
183          return _dummyTrans and trans.response().getvalue() or ""

184           
185   


186 -    def extraheaders(self, **KWS):


187   
188   
189   
190          ## CHEETAH: generated from #block extraheaders at line 27, col 1. 
191          trans = KWS.get("trans") 
192          if (not trans and not self._CHEETAH__isBuffering and not callable(self.transaction)): 
193              trans = self.transaction # is None unless self.awake() was called 
194          if not trans: 
195              trans = DummyTransaction() 
196              _dummyTrans = True 
197          else: _dummyTrans = False 
198          write = trans.response().write 
199          SL = self._CHEETAH__searchList 
200          _filter = self._CHEETAH__currentFilter 
201           
202          ######################################## 
203          ## START - generated method body 
204           
205           
206          ######################################## 
207          ## END - generated method body 
208           
209          return _dummyTrans and trans.response().getvalue() or ""

210           
211   


212 -    def head(self, **KWS):


213   
214   
215   
216          ## CHEETAH: generated from #block head at line 15, col 1. 
217          trans = KWS.get("trans") 
218          if (not trans and not self._CHEETAH__isBuffering and not callable(self.transaction)): 
219              trans = self.transaction # is None unless self.awake() was called 
220          if not trans: 
221              trans = DummyTransaction() 
222              _dummyTrans = True 
223          else: _dummyTrans = False 
224          write = trans.response().write 
225          SL = self._CHEETAH__searchList 
226          _filter = self._CHEETAH__currentFilter 
227           
228          ######################################## 
229          ## START - generated method body 
230           
231          write('<head>\n\n') 
232          self.wintitle(trans=trans) 
233          write('\n') 
234          self.stdheaders(trans=trans) 
235          write('\n') 
236          self.extraheaders(trans=trans) 
237          write('\n</head>\n') 
238           
239          ######################################## 
240          ## END - generated method body 
241           
242          return _dummyTrans and trans.response().getvalue() or ""

243           
244   


245 -    def statusblock(self, task, queue, **KWS):


246   
247   
248   
249          ## CHEETAH: generated from #def statusblock(task, queue) at line 33, col 1. 
250          trans = KWS.get("trans") 
251          if (not trans and not self._CHEETAH__isBuffering and not callable(self.transaction)): 
252              trans = self.transaction # is None unless self.awake() was called 
253          if not trans: 
254              trans = DummyTransaction() 
255              _dummyTrans = True 
256          else: _dummyTrans = False 
257          write = trans.response().write 
258          SL = self._CHEETAH__searchList 
259          _filter = self._CHEETAH__currentFilter 
260           
261          ######################################## 
262          ## START - generated method body 
263           
264          #  Print a table for task status 
265          #  task -- Storage object representing the task 
266          #  queue -- QueueStatus object 
267          write('''<table class="status"> 
268    <thead> 
269    <tr> 
270    <th colspan="2"> 
271      Task <q>''') 
272          _v = VFSL([locals()]+SL+[globals(), __builtin__],"task.dataset",True) # '$task.dataset' on line 41, col 13 
273          if _v is not None: write(_filter(_v, rawExpr='$task.dataset')) # from line 41, col 13. 
274          write('</q> (') 
275          _v = VFSL([locals()]+SL+[globals(), __builtin__],"task.operation",True) # '$task.operation' on line 41, col 32 
276          if _v is not None: write(_filter(_v, rawExpr='$task.operation')) # from line 41, col 32. 
277          write(''') 
278    </th> 
279    </tr> 
280    </thead> 
281    <tbody> 
282    <tr> 
283      <th>Task status</th> 
284      <td> 
285  ''') 
286          _v = VFN(VFSL([locals()]+SL+[globals(), __builtin__],"queue",True),"status",True)[VFSL([locals()]+SL+[globals(), __builtin__],"task.dataset",True)] 
287          if _v is not None: write(_filter(_v)) 
288          write('''    </td> 
289    </tr> 
290    <tr> 
291      <th>Destination</th> 
292      <td> 
293      <a href="/static/output/''') 
294          _v = VFSL([locals()]+SL+[globals(), __builtin__],"task.dataset",True) # '${task.dataset}' on line 55, col 29 
295          if _v is not None: write(_filter(_v, rawExpr='${task.dataset}')) # from line 55, col 29. 
296          write('/">') 
297          _v = VFSL([locals()]+SL+[globals(), __builtin__],"task.dataset",True) # '$task.dataset' on line 55, col 47 
298          if _v is not None: write(_filter(_v, rawExpr='$task.dataset')) # from line 55, col 47. 
299          write('''</a> 
300      </td> 
301    </tr> 
302    <tr> 
303      <th>Submitted at</th> 
304      <td> 
305      ''') 
306          _v = VFN(VFSL([locals()]+SL+[globals(), __builtin__],"time",True),"strftime",False)("%Y/%m/%d %H:%M:%S GMT", VFN(VFSL([locals()]+SL+[globals(), __builtin__],"time",True),"gmtime",False)(VFSL([locals()]+SL+[globals(), __builtin__],"task.submitted",True))) # '${time.strftime("%Y/%m/%d %H:%M:%S GMT", $time.gmtime($task.submitted))}' on line 62, col 5 
307          if _v is not None: write(_filter(_v, rawExpr='${time.strftime("%Y/%m/%d %H:%M:%S GMT", $time.gmtime($task.submitted))}')) # from line 62, col 5. 
308          write(''' 
309      </td> 
310    </tr> 
311  ''') 
312          if VFN(VFSL([locals()]+SL+[globals(), __builtin__],"queue",True),"status",True)[VFSL([locals()]+SL+[globals(), __builtin__],"task.dataset",True)] == VFSL([locals()]+SL+[globals(), __builtin__],"queue.RUNNING",True): # generated from line 65, col 3 
313              write('''  <tr> 
314      <th>Time spent running</th> 
315      <td> 
316  ''') 
317              #  Mod time is updated in queue.py when starting the task 
318              _v = "%d seconds" % int(VFN(VFSL([locals()]+SL+[globals(), __builtin__],"time",True),"time",False)() - VFSL([locals()]+SL+[globals(), __builtin__],"task._filename.mtime",True)) 
319              if _v is not None: write(_filter(_v)) 
320              write('''    </td> 
321    </tr> 
322    <tr> 
323      <th>Tasks remaining</th> 
324      <td> 
325  ''') 
326              _v = len(VFSL([locals()]+SL+[globals(), __builtin__],"queue.tasklist",True)) - 1 
327              if _v is not None: write(_filter(_v)) 
328              write('    </td>\n  </tr>  \n') 
329          elif VFN(VFSL([locals()]+SL+[globals(), __builtin__],"queue",True),"status",True)[VFSL([locals()]+SL+[globals(), __builtin__],"task.dataset",True)] == VFSL([locals()]+SL+[globals(), __builtin__],"queue.WAITING",True): # generated from line 79, col 3 
330              write('''  <tr> 
331      <th>Tasks to go</th> 
332      <td> 
333  ''') 
334              _v = VFN(VFSL([locals()]+SL+[globals(), __builtin__],"queue",True),"position",False)(VFSL([locals()]+SL+[globals(), __builtin__],"task.dataset",True)) 
335              if _v is not None: write(_filter(_v)) 
336              write('    </td>\n  </tr>\n') 
337          write('  </tbody>\n</table>\n') 
338           
339          ######################################## 
340          ## END - generated method body 
341           
342          return _dummyTrans and trans.response().getvalue() or ""

343           
344   


345 -    def header_text(self, **KWS):


346   
347   
348   
349          ## CHEETAH: generated from #block header_text at line 96, col 3. 
350          trans = KWS.get("trans") 
351          if (not trans and not self._CHEETAH__isBuffering and not callable(self.transaction)): 
352              trans = self.transaction # is None unless self.awake() was called 
353          if not trans: 
354              trans = DummyTransaction() 
355              _dummyTrans = True 
356          else: _dummyTrans = False 
357          write = trans.response().write 
358          SL = self._CHEETAH__searchList 
359          _filter = self._CHEETAH__currentFilter 
360           
361          ######################################## 
362          ## START - generated method body 
363           
364          write('  <h1>') 
365          _v = VFSL([locals()]+SL+[globals(), __builtin__],"title",True) # '$title' on line 97, col 7 
366          if _v is not None: write(_filter(_v, rawExpr='$title')) # from line 97, col 7. 
367          write('</h1>\n') 
368           
369          ######################################## 
370          ## END - generated method body 
371           
372          return _dummyTrans and trans.response().getvalue() or ""

373           
374   


375 -    def header(self, **KWS):


376   
377   
378   
379          ## CHEETAH: generated from #block header at line 94, col 1. 
380          trans = KWS.get("trans") 
381          if (not trans and not self._CHEETAH__isBuffering and not callable(self.transaction)): 
382              trans = self.transaction # is None unless self.awake() was called 
383          if not trans: 
384              trans = DummyTransaction() 
385              _dummyTrans = True 
386          else: _dummyTrans = False 
387          write = trans.response().write 
388          SL = self._CHEETAH__searchList 
389          _filter = self._CHEETAH__currentFilter 
390           
391          ######################################## 
392          ## START - generated method body 
393           
394          write('<div id="header">\n') 
395          self.header_text(trans=trans) 
396          write('</div>\n') 
397           
398          ######################################## 
399          ## END - generated method body 
400           
401          return _dummyTrans and trans.response().getvalue() or ""

402           
403   


404 -    def topmenu(self, **KWS):


405   
406   
407   
408          ## CHEETAH: generated from #block topmenu at line 102, col 1. 
409          trans = KWS.get("trans") 
410          if (not trans and not self._CHEETAH__isBuffering and not callable(self.transaction)): 
411              trans = self.transaction # is None unless self.awake() was called 
412          if not trans: 
413              trans = DummyTransaction() 
414              _dummyTrans = True 
415          else: _dummyTrans = False 
416          write = trans.response().write 
417          SL = self._CHEETAH__searchList 
418          _filter = self._CHEETAH__currentFilter 
419           
420          ######################################## 
421          ## START - generated method body 
422           
423          write('''<div id="topmenu"> 
424    <table> 
425      <tr> 
426        <td> 
427          <a href="/">Home</a> 
428        </td> 
429        <td> 
430          <a href="/query">Query</a> 
431        </td> 
432        <td> 
433          <a href="/status">Status</a> 
434        </td> 
435        <td> 
436          <a href="/output">Output</a> 
437        </td> 
438        <td> 
439          <a href="/contact">Contact Us</a> 
440        </td> 
441      </tr> 
442    </table> 
443  </div> 
444  ''') 
445           
446          ######################################## 
447          ## END - generated method body 
448           
449          return _dummyTrans and trans.response().getvalue() or ""

450           
451   


452 -    def contents(self, **KWS):


453   
454   
455   
456          ## CHEETAH: generated from #block contents at line 128, col 1. 
457          trans = KWS.get("trans") 
458          if (not trans and not self._CHEETAH__isBuffering and not callable(self.transaction)): 
459              trans = self.transaction # is None unless self.awake() was called 
460          if not trans: 
461              trans = DummyTransaction() 
462              _dummyTrans = True 
463          else: _dummyTrans = False 
464          write = trans.response().write 
465          SL = self._CHEETAH__searchList 
466          _filter = self._CHEETAH__currentFilter 
467           
468          ######################################## 
469          ## START - generated method body 
470           
471          write('CONTENTS\n') 
472           
473          ######################################## 
474          ## END - generated method body 
475           
476          return _dummyTrans and trans.response().getvalue() or ""

477           
478   


479 -    def contents_outer(self, **KWS):


480   
481   
482   
483          ## CHEETAH: generated from #block contents_outer at line 126, col 1. 
484          trans = KWS.get("trans") 
485          if (not trans and not self._CHEETAH__isBuffering and not callable(self.transaction)): 
486              trans = self.transaction # is None unless self.awake() was called 
487          if not trans: 
488              trans = DummyTransaction() 
489              _dummyTrans = True 
490          else: _dummyTrans = False 
491          write = trans.response().write 
492          SL = self._CHEETAH__searchList 
493          _filter = self._CHEETAH__currentFilter 
494           
495          ######################################## 
496          ## START - generated method body 
497           
498          write('<div id="contents">\n') 
499          self.contents(trans=trans) 
500          write('</div>\n') 
501           
502          ######################################## 
503          ## END - generated method body 
504           
505          return _dummyTrans and trans.response().getvalue() or ""

506           
507   


508 -    def footer_text(self, **KWS):


509   
510   
511   
512          ## CHEETAH: generated from #block footer_text at line 136, col 3. 
513          trans = KWS.get("trans") 
514          if (not trans and not self._CHEETAH__isBuffering and not callable(self.transaction)): 
515              trans = self.transaction # is None unless self.awake() was called 
516          if not trans: 
517              trans = DummyTransaction() 
518              _dummyTrans = True 
519          else: _dummyTrans = False 
520          write = trans.response().write 
521          SL = self._CHEETAH__searchList 
522          _filter = self._CHEETAH__currentFilter 
523           
524          ######################################## 
525          ## START - generated method body 
526           
527          write('  &copy; 2007 Graham Poulter\n') 
528           
529          ######################################## 
530          ## END - generated method body 
531           
532          return _dummyTrans and trans.response().getvalue() or ""

533           
534   


535 -    def footer(self, **KWS):


536   
537   
538   
539          ## CHEETAH: generated from #block footer at line 134, col 1. 
540          trans = KWS.get("trans") 
541          if (not trans and not self._CHEETAH__isBuffering and not callable(self.transaction)): 
542              trans = self.transaction # is None unless self.awake() was called 
543          if not trans: 
544              trans = DummyTransaction() 
545              _dummyTrans = True 
546          else: _dummyTrans = False 
547          write = trans.response().write 
548          SL = self._CHEETAH__searchList 
549          _filter = self._CHEETAH__currentFilter 
550           
551          ######################################## 
552          ## START - generated method body 
553           
554          write('<div id="footer">\n') 
555          self.footer_text(trans=trans) 
556          write('</div>\n') 
557           
558          ######################################## 
559          ## END - generated method body 
560           
561          return _dummyTrans and trans.response().getvalue() or ""

562           
563   


564 -    def body(self, **KWS):


565   
566   
567   
568          ## CHEETAH: generated from #block body at line 91, col 1. 
569          trans = KWS.get("trans") 
570          if (not trans and not self._CHEETAH__isBuffering and not callable(self.transaction)): 
571              trans = self.transaction # is None unless self.awake() was called 
572          if not trans: 
573              trans = DummyTransaction() 
574              _dummyTrans = True 
575          else: _dummyTrans = False 
576          write = trans.response().write 
577          SL = self._CHEETAH__searchList 
578          _filter = self._CHEETAH__currentFilter 
579           
580          ######################################## 
581          ## START - generated method body 
582           
583          write('<body>\n\n') 
584          self.header(trans=trans) 
585          write('\n') 
586          self.topmenu(trans=trans) 
587          write('\n') 
588          self.contents_outer(trans=trans) 
589          write('\n') 
590          self.footer(trans=trans) 
591          write('\n</body>\n') 
592           
593          ######################################## 
594          ## END - generated method body 
595           
596          return _dummyTrans and trans.response().getvalue() or ""

597           
598   


599 -    def html(self, **KWS):


600   
601   
602   
603          ## CHEETAH: generated from #block html at line 12, col 1. 
604          trans = KWS.get("trans") 
605          if (not trans and not self._CHEETAH__isBuffering and not callable(self.transaction)): 
606              trans = self.transaction # is None unless self.awake() was called 
607          if not trans: 
608              trans = DummyTransaction() 
609              _dummyTrans = True 
610          else: _dummyTrans = False 
611          write = trans.response().write 
612          SL = self._CHEETAH__searchList 
613          _filter = self._CHEETAH__currentFilter 
614           
615          ######################################## 
616          ## START - generated method body 
617           
618          write('<html>\n\n') 
619          self.head(trans=trans) 
620          write('\n\n') 
621          self.body(trans=trans) 
622          write('\n</html>\n') 
623           
624          ######################################## 
625          ## END - generated method body 
626           
627          return _dummyTrans and trans.response().getvalue() or ""

628           
629   


630 -    def respond(self, trans=None):


631   
632   
633   
634          ## CHEETAH: main method generated for this template 
635          if (not trans and not self._CHEETAH__isBuffering and not callable(self.transaction)): 
636              trans = self.transaction # is None unless self.awake() was called 
637          if not trans: 
638              trans = DummyTransaction() 
639              _dummyTrans = True 
640          else: _dummyTrans = False 
641          write = trans.response().write 
642          SL = self._CHEETAH__searchList 
643          _filter = self._CHEETAH__currentFilter 
644           
645          ######################################## 
646          ## START - generated method body 
647           
648          self.doctype(trans=trans) 
649          write('\n') 
650          #  Base URL for the website, for "absolute" links 
651          write('\n\n') 
652          self.html(trans=trans) 
653           
654          ######################################## 
655          ## END - generated method body 
656           
657          return _dummyTrans and trans.response().getvalue() or ""

658           
659      ################################################## 
660      ## CHEETAH GENERATED ATTRIBUTES 
661   
662   
663      _CHEETAH__instanceInitialized = False 
664   
665      _CHEETAH_version = __CHEETAH_version__ 
666   
667      _CHEETAH_versionTuple = __CHEETAH_versionTuple__ 
668   
669      _CHEETAH_genTime = __CHEETAH_genTime__ 
670   
671      _CHEETAH_genTimestamp = __CHEETAH_genTimestamp__ 
672   
673      _CHEETAH_src = __CHEETAH_src__ 
674   
675      _CHEETAH_srcLastModified = __CHEETAH_srcLastModified__ 
676   
677      base = "" 
678   
679      _mainCheetahMethod_for_page= 'respond'

680   
681  ## END CLASS DEFINITION 
682   
683  if not hasattr(page, '_initCheetahAttributes'): 
684      templateAPIClass = getattr(page, '_CHEETAH_templateClass', Template) 
685      templateAPIClass._addCheetahPlumbingCodeToClass(page) 
686   
687   
688  # CHEETAH was developed by Tavis Rudd and Mike Orr 
689  # with code, advice and input from many other volunteers. 
690  # For more information visit http://www.CheetahTemplate.org/ 
691   
692  ################################################## 
693  ## if run from command line: 
694  if __name__ == '__main__': 
695      from Cheetah.TemplateCmdLineIface import CmdLineIface 
696      CmdLineIface(templateObj=page()).run() 
697
```

  


| Trees | Indices | Help | | MScanner | | --- | |
| --- | --- | --- | --- | --- |

|  |  |
| --- | --- |
| Generated by Epydoc 3.0beta1 on Fri Nov 23 09:13:23 2007 | http://epydoc.sourceforge.net |
