# Supplementary material for: MScanner: a classifier for retrieving Medline citations
Source: BMC Bioinformatics. 2008 Feb 19;9:108. doi: 10.1186/1471-2105-9-108 (PMC2263023; doi:10.1186/1471-2105-9-108)
Supplement: Additional file 3 — Source code for MScanner. mscanner-20071123.zip is a ZIP archive containing the Python 2.5 source code for MScanner, licensed under the GNU General Public License. It also contains API documentation in HTML format. Updated versions will be made available at . [file 1471-2105-9-108-S3.zip › mscanner/help/api/mscanner.htdocs.templates.query-pysrc.html]

xml version="1.0" encoding="ascii"?


mscanner.htdocs.templates.query


| Trees | Indices | Help | | MScanner | | --- | |
| --- | --- | --- | --- | --- |

|  |  |  |  |
| --- | --- | --- | --- |
| Package mscanner :: Package htdocs :: Package templates :: Module query | |  | | --- | | [hide private] | | [frames] | no frames] | |

# Source Code for Module mscanner.htdocs.templates.query

```
  1  #!/usr/bin/env python 
  2   
  3   
  4   
  5   
  6  ################################################## 
  7  ## DEPENDENCIES 
  8  import sys 
  9  import os 
 10  import os.path 
 11  from os.path import getmtime, exists 
 12  import time 
 13  import types 
 14  import __builtin__ 
 15  from Cheetah.Version import MinCompatibleVersion as RequiredCheetahVersion 
 16  from Cheetah.Version import MinCompatibleVersionTuple as RequiredCheetahVersionTuple 
 17  from Cheetah.Template import Template 
 18  from Cheetah.DummyTransaction import DummyTransaction 
 19  from Cheetah.NameMapper import NotFound, valueForName, valueFromSearchList, valueFromFrameOrSearchList 
 20  from Cheetah.CacheRegion import CacheRegion 
 21  import Cheetah.Filters as Filters 
 22  import Cheetah.ErrorCatchers as ErrorCatchers 
 23  from page import page 
 24   
 25  ################################################## 
 26  ## MODULE CONSTANTS 
 27  try: 
 28      True, False 
 29  except NameError: 
 30      True, False = (1==1), (1==0) 
 31  VFFSL=valueFromFrameOrSearchList 
 32  VFSL=valueFromSearchList 
 33  VFN=valueForName 
 34  currentTime=time.time 
 35  __CHEETAH_version__ = '2.0rc7' 
 36  __CHEETAH_versionTuple__ = (2, 0, 0, 'candidate', 7) 
 37  __CHEETAH_genTime__ = 1195756172.0239999 
 38  __CHEETAH_genTimestamp__ = 'Thu Nov 22 20:29:32 2007' 
 39  __CHEETAH_src__ = 'query.tmpl' 
 40  __CHEETAH_srcLastModified__ = 'Thu Nov 22 20:20:58 2007' 
 41  __CHEETAH_docstring__ = 'Autogenerated by CHEETAH: The Python-Powered Template Engine' 
 42   
 43  if __CHEETAH_versionTuple__ < RequiredCheetahVersionTuple: 
 44      raise AssertionError( 
 45        'This template was compiled with Cheetah version' 
 46        ' %s. Templates compiled before version %s must be recompiled.'%( 
 47           __CHEETAH_version__, RequiredCheetahVersion)) 
 48   
 49  ################################################## 
 50  ## CLASSES 
 51   


52 -class query(page):


53   
 54      ################################################## 
 55      ## CHEETAH GENERATED METHODS 
 56   
 57   


58 -    def __init__(self, *args, **KWs):


59   
 60          page.__init__(self, *args, **KWs) 
 61          if not self._CHEETAH__instanceInitialized: 
 62              cheetahKWArgs = {} 
 63              allowedKWs = 'searchList namespaces filter filtersLib errorCatcher'.split() 
 64              for k,v in KWs.items(): 
 65                  if k in allowedKWs: cheetahKWArgs[k] = v 
 66              self._initCheetahInstance(**cheetahKWArgs)

 67           
 68   


69 -    def title(self, **KWS):


70   
 71   
 72   
 73          ## CHEETAH: generated from #def title at line 7, col 1. 
 74          trans = KWS.get("trans") 
 75          if (not trans and not self._CHEETAH__isBuffering and not callable(self.transaction)): 
 76              trans = self.transaction # is None unless self.awake() was called 
 77          if not trans: 
 78              trans = DummyTransaction() 
 79              _dummyTrans = True 
 80          else: _dummyTrans = False 
 81          write = trans.response().write 
 82          SL = self._CHEETAH__searchList 
 83          _filter = self._CHEETAH__currentFilter 
 84           
 85          ######################################## 
 86          ## START - generated method body 
 87           
 88          write('Submit Query or Validation ') 
 89           
 90          ######################################## 
 91          ## END - generated method body 
 92           
 93          return _dummyTrans and trans.response().getvalue() or ""

 94           
 95   


96 -    def extraheaders(self, **KWS):


97   
 98   
 99   
100          ## CHEETAH: generated from #def extraheaders at line 11, col 1. 
101          trans = KWS.get("trans") 
102          if (not trans and not self._CHEETAH__isBuffering and not callable(self.transaction)): 
103              trans = self.transaction # is None unless self.awake() was called 
104          if not trans: 
105              trans = DummyTransaction() 
106              _dummyTrans = True 
107          else: _dummyTrans = False 
108          write = trans.response().write 
109          SL = self._CHEETAH__searchList 
110          _filter = self._CHEETAH__currentFilter 
111           
112          ######################################## 
113          ## START - generated method body 
114           
115          #  Uncompressed javascript for debugging (77k) 
116          # <script type="text/javascript" src="$base/static/mootools.js"></script> 
117          #  Compressed javascript for production (13k) 
118          write('  <script type="text/javascript" src="') 
119          _v = VFSL([locals()]+SL+[globals(), __builtin__],"base",True) # '$base' on line 15, col 39 
120          if _v is not None: write(_filter(_v, rawExpr='$base')) # from line 15, col 39. 
121          write('/static/mootools.zip.js"></script>\n  \n') 
122          #  Inline Javascript 
123          write('''  <script type="text/javascript"> 
124      /*  
125      When a button of class "toggle" and id toggle_x is clicked, 
126      it will slide-toggle the target with id x. Targets are initially hidden. 
127      */ 
128      function create_sliders() { 
129        /* handler for button with id like toggle_x */ 
130        function toggle_handler() { 
131          $(this.id.substr(7)).slider.toggle(); // "toggle_x" -> "x" 
132        } 
133        /* for each toggle button, we link and hide its target */ 
134        var buttons = document.getElementsByTagName("button"); 
135        for(var i = 0; i < buttons.length; i++) { 
136          button = buttons[i]; 
137          if(button.className == 'toggle') { 
138            id = button.id.substr(7); 
139            target = $(id) 
140            if(target) { 
141              target.slider = new Fx.Slide(id, {"duration": 500}); 
142              target.slider.hide(); 
143              button.onclick = toggle_handler; 
144            } 
145          } 
146        } 
147      } 
148      window.onload = function() { 
149        $('fill_example').onclick = function () { 
150           $('dataset').value = "example-radiology-query" 
151           $('positives').value = "10390570\\n10555669\\n10587123\\n10658726\\n10702822\\n10747308\\n11092485\\n11812973\\n12216754\\n12438060\\n12708922\\n12828616\\n12945936\\n14510633\\n14628859\\n14700161\\n14752189\\n14914750\\n15256634\\n15310152\\n15351243\\n15405683\\n15480032\\n15520901\\n15717221\\n15718419\\n1592926\\n16009818\\n16019460\\n16154823\\n16189163\\n16272860\\n16299797\\n16344746\\n16415460\\n16488085\\n1727362\\n1899742\\n1928554\\n1950868\\n1984330\\n2406785\\n2731700\\n2806818\\n3275982\\n3279735\\n3416282\\n3840980\\n3871147\\n4040300\\n5327041\\n5368340\\n6497634\\n6783686\\n6856834\\n7034554\\n7480744\\n8190956\\n8208972\\n8316667\\n8372948\\n8615251\\n8911169\\n8988207\\n8988208\\n9754100\\n9922200\\n9922201\\n" 
152        } 
153        create_sliders(); 
154      } 
155    </script> 
156  ''') 
157           
158          ######################################## 
159          ## END - generated method body 
160           
161          return _dummyTrans and trans.response().getvalue() or ""

162           
163   


164 -    def form_row(self, name, **KWS):


165   
166   
167   
168          ## CHEETAH: generated from #def form_row(name) at line 53, col 1. 
169          trans = KWS.get("trans") 
170          if (not trans and not self._CHEETAH__isBuffering and not callable(self.transaction)): 
171              trans = self.transaction # is None unless self.awake() was called 
172          if not trans: 
173              trans = DummyTransaction() 
174              _dummyTrans = True 
175          else: _dummyTrans = False 
176          write = trans.response().write 
177          SL = self._CHEETAH__searchList 
178          _filter = self._CHEETAH__currentFilter 
179           
180          ######################################## 
181          ## START - generated method body 
182           
183          elem = VFSL([locals()]+SL+[globals(), __builtin__],"inputs",True)[name] 
184          write('<tr class="input">\n  <th>') 
185          _v = VFN(VFSL([locals()]+SL+[globals(), __builtin__],"elem",True),"renderlabel",False)() # '$elem.renderlabel()' on line 56, col 7 
186          if _v is not None: write(_filter(_v, rawExpr='$elem.renderlabel()')) # from line 56, col 7. 
187          write('</th>\n  <td class="value">\n    ') 
188          _v = VFSL([locals()]+SL+[globals(), __builtin__],"elem.pre",True) # '$elem.pre' on line 58, col 5 
189          if _v is not None: write(_filter(_v, rawExpr='$elem.pre')) # from line 58, col 5. 
190          write(' ') 
191          _v = VFN(VFSL([locals()]+SL+[globals(), __builtin__],"elem",True),"render",False)() # '$elem.render()' on line 58, col 15 
192          if _v is not None: write(_filter(_v, rawExpr='$elem.render()')) # from line 58, col 15. 
193          write(' ') 
194          _v = VFSL([locals()]+SL+[globals(), __builtin__],"elem.post",True) # '$elem.post' on line 58, col 30 
195          if _v is not None: write(_filter(_v, rawExpr='$elem.post')) # from line 58, col 30. 
196          write('\n') 
197          if VFSL([locals()]+SL+[globals(), __builtin__],"name",True) == "delcode": # generated from line 59, col 5 
198              write('    ') 
199              _v = VFN(VFSL([locals()]+SL+[globals(), __builtin__],"inputs",True)["hidden"],"render",False)() # '$inputs["hidden"].render()' on line 60, col 5 
200              if _v is not None: write(_filter(_v, rawExpr='$inputs["hidden"].render()')) # from line 60, col 5. 
201              write(' ') 
202              _v = VFN(VFSL([locals()]+SL+[globals(), __builtin__],"inputs",True)["hidden"],"renderlabel",False)() # '$inputs["hidden"].renderlabel()' on line 60, col 32 
203              if _v is not None: write(_filter(_v, rawExpr='$inputs["hidden"].renderlabel()')) # from line 60, col 32. 
204              write('\n') 
205          write('  </td>\n  <td class="help">\n    <button type="button" class="toggle" id="toggle_h_') 
206          _v = VFSL([locals()]+SL+[globals(), __builtin__],"name",True) # '$name' on line 64, col 55 
207          if _v is not None: write(_filter(_v, rawExpr='$name')) # from line 64, col 55. 
208          write('''">help</button> 
209    </td> 
210  </tr> 
211  ''') 
212          if VFSL([locals()]+SL+[globals(), __builtin__],"elem.note",True) is not None: # generated from line 67, col 1 
213              write('<tr class="error">\n  <td colspan="3">\n    ') 
214              _v = VFSL([locals()]+SL+[globals(), __builtin__],"elem.note",True) # '$elem.note' on line 70, col 5 
215              if _v is not None: write(_filter(_v, rawExpr='$elem.note')) # from line 70, col 5. 
216              write(''' 
217    </td> 
218  </tr> 
219  ''') 
220          write('<tr class="help">\n  <td colspan="3">\n    <div id="h_') 
221          _v = VFSL([locals()]+SL+[globals(), __builtin__],"name",True) # '$name' on line 76, col 16 
222          if _v is not None: write(_filter(_v, rawExpr='$name')) # from line 76, col 16. 
223          write('">\n      ') 
224          _v = VFSL([locals()]+SL+[globals(), __builtin__],"getattr",False)(self, "help_" + VFSL([locals()]+SL+[globals(), __builtin__],"name",True))() # '${getattr(self, "help_" + $name)()}' on line 77, col 7 
225          if _v is not None: write(_filter(_v, rawExpr='${getattr(self, "help_" + $name)()}')) # from line 77, col 7. 
226          write(''' 
227      </div> 
228    </td> 
229  </tr> 
230  ''') 
231           
232          ######################################## 
233          ## END - generated method body 
234           
235          return _dummyTrans and trans.response().getvalue() or ""

236           
237   


238 -    def help_positives(self, **KWS):


239   
240   
241   
242          ## CHEETAH: generated from #def help_positives at line 83, col 1. 
243          trans = KWS.get("trans") 
244          if (not trans and not self._CHEETAH__isBuffering and not callable(self.transaction)): 
245              trans = self.transaction # is None unless self.awake() was called 
246          if not trans: 
247              trans = DummyTransaction() 
248              _dummyTrans = True 
249          else: _dummyTrans = False 
250          write = trans.response().write 
251          SL = self._CHEETAH__searchList 
252          _filter = self._CHEETAH__currentFilter 
253           
254          ######################################## 
255          ## START - generated method body 
256           
257          write(''' A list of PubMed IDs, one per line, to serve 
258  as relevant training examples (the rest of Medline is used to approximate 
259  term frequencies in irrelevant articles). 
260  ''') 
261           
262          ######################################## 
263          ## END - generated method body 
264           
265          return _dummyTrans and trans.response().getvalue() or ""

266           
267   


268 -    def help_dataset(self, **KWS):


269   
270   
271   
272          ## CHEETAH: generated from #def help_dataset at line 88, col 1. 
273          trans = KWS.get("trans") 
274          if (not trans and not self._CHEETAH__isBuffering and not callable(self.transaction)): 
275              trans = self.transaction # is None unless self.awake() was called 
276          if not trans: 
277              trans = DummyTransaction() 
278              _dummyTrans = True 
279          else: _dummyTrans = False 
280          write = trans.response().write 
281          SL = self._CHEETAH__searchList 
282          _filter = self._CHEETAH__currentFilter 
283           
284          ######################################## 
285          ## START - generated method body 
286           
287          write(' The name of the task, to make it easier to locate on the\nresults page (restriced to letters, numbers and .,;:- punctuation)') 
288           
289          ######################################## 
290          ## END - generated method body 
291           
292          return _dummyTrans and trans.response().getvalue() or ""

293           
294   


295 -    def help_delcode(self, **KWS):


296   
297   
298   
299          ## CHEETAH: generated from #def help_delcode at line 91, col 1. 
300          trans = KWS.get("trans") 
301          if (not trans and not self._CHEETAH__isBuffering and not callable(self.transaction)): 
302              trans = self.transaction # is None unless self.awake() was called 
303          if not trans: 
304              trans = DummyTransaction() 
305              _dummyTrans = True 
306          else: _dummyTrans = False 
307          write = trans.response().write 
308          SL = self._CHEETAH__searchList 
309          _filter = self._CHEETAH__currentFilter 
310           
311          ######################################## 
312          ## START - generated method body 
313           
314          write(''' A short code that will be 
315  required if in order to delete the output (defaults to an empty code).  
316  You may select "hide output" to prevent the results from being listed  
317  on the <a href="output">output</a> page, in which case you need to  
318  bookmark the location of the results to find them later. 
319  ''') 
320           
321          ######################################## 
322          ## END - generated method body 
323           
324          return _dummyTrans and trans.response().getvalue() or ""

325           
326   


327 -    def help_limit(self, **KWS):


328   
329   
330   
331          ## CHEETAH: generated from #def help_limit at line 98, col 1. 
332          trans = KWS.get("trans") 
333          if (not trans and not self._CHEETAH__isBuffering and not callable(self.transaction)): 
334              trans = self.transaction # is None unless self.awake() was called 
335          if not trans: 
336              trans = DummyTransaction() 
337              _dummyTrans = True 
338          else: _dummyTrans = False 
339          write = trans.response().write 
340          SL = self._CHEETAH__searchList 
341          _filter = self._CHEETAH__currentFilter 
342           
343          ######################################## 
344          ## START - generated method body 
345           
346          write(' Upper limit on the number of citations to return (may be\nset between 100 and 10000). \n') 
347           
348          ######################################## 
349          ## END - generated method body 
350           
351          return _dummyTrans and trans.response().getvalue() or ""

352           
353   


354 -    def help_mindate(self, **KWS):


355   
356   
357   
358          ## CHEETAH: generated from #def help_mindate at line 102, col 1. 
359          trans = KWS.get("trans") 
360          if (not trans and not self._CHEETAH__isBuffering and not callable(self.transaction)): 
361              trans = self.transaction # is None unless self.awake() was called 
362          if not trans: 
363              trans = DummyTransaction() 
364              _dummyTrans = True 
365          else: _dummyTrans = False 
366          write = trans.response().write 
367          SL = self._CHEETAH__searchList 
368          _filter = self._CHEETAH__currentFilter 
369           
370          ######################################## 
371          ## START - generated method body 
372           
373          write(''' Filter Medline to contain only records completed after this 
374  date (useful for monitoring for new literature).  Using this option 
375  slows down retrieval by 30 seconds. Records are typically completed  
376  1-3 months after publication date. 
377  ''') 
378           
379          ######################################## 
380          ## END - generated method body 
381           
382          return _dummyTrans and trans.response().getvalue() or ""

383           
384   


385 -    def help_prevalence(self, **KWS):


386   
387   
388   
389          ## CHEETAH: generated from #def help_prevalence at line 108, col 1. 
390          trans = KWS.get("trans") 
391          if (not trans and not self._CHEETAH__isBuffering and not callable(self.transaction)): 
392              trans = self.transaction # is None unless self.awake() was called 
393          if not trans: 
394              trans = DummyTransaction() 
395              _dummyTrans = True 
396          else: _dummyTrans = False 
397          write = trans.response().write 
398          SL = self._CHEETAH__searchList 
399          _filter = self._CHEETAH__currentFilter 
400           
401          ######################################## 
402          ## START - generated method body 
403           
404          write(''' Advanced option: specify the fraction of Medline that is 
405  relevant (prevalence). By default we estimate this using on input size.  
406  Greater values yield more results by raising the prior probability of relevance.  
407  ''') 
408           
409          ######################################## 
410          ## END - generated method body 
411           
412          return _dummyTrans and trans.response().getvalue() or ""

413           
414   


415 -    def help_minscore(self, **KWS):


416   
417   
418   
419          ## CHEETAH: generated from #def help_minscore at line 113, col 1. 
420          trans = KWS.get("trans") 
421          if (not trans and not self._CHEETAH__isBuffering and not callable(self.transaction)): 
422              trans = self.transaction # is None unless self.awake() was called 
423          if not trans: 
424              trans = DummyTransaction() 
425              _dummyTrans = True 
426          else: _dummyTrans = False 
427          write = trans.response().write 
428          SL = self._CHEETAH__searchList 
429          _filter = self._CHEETAH__currentFilter 
430           
431          ######################################## 
432          ## START - generated method body 
433           
434          write(''' Advanced option: Only returns articles whose  
435  score (logarithm of the probability ratio for relevance vs irrelevance) 
436  is greater than this.  Default of 0 means to predict relevance when 
437  it is more probable than irrelevance. 
438  ''') 
439           
440          ######################################## 
441          ## END - generated method body 
442           
443          return _dummyTrans and trans.response().getvalue() or ""

444           
445   


446 -    def help_numnegs(self, **KWS):


447   
448   
449   
450          ## CHEETAH: generated from #def help_numnegs at line 120, col 1. 
451          trans = KWS.get("trans") 
452          if (not trans and not self._CHEETAH__isBuffering and not callable(self.transaction)): 
453              trans = self.transaction # is None unless self.awake() was called 
454          if not trans: 
455              trans = DummyTransaction() 
456              _dummyTrans = True 
457          else: _dummyTrans = False 
458          write = trans.response().write 
459          SL = self._CHEETAH__searchList 
460          _filter = self._CHEETAH__currentFilter 
461           
462          ######################################## 
463          ## START - generated method body 
464           
465          write(' In cross validation we select this many random Medline \nrecords to serve as irrelevant training examples.\n') 
466           
467          ######################################## 
468          ## END - generated method body 
469           
470          return _dummyTrans and trans.response().getvalue() or ""

471           
472   


473 -    def contents(self, **KWS):


474   
475   
476   
477          ## CHEETAH: generated from #def contents at line 125, col 1. 
478          trans = KWS.get("trans") 
479          if (not trans and not self._CHEETAH__isBuffering and not callable(self.transaction)): 
480              trans = self.transaction # is None unless self.awake() was called 
481          if not trans: 
482              trans = DummyTransaction() 
483              _dummyTrans = True 
484          else: _dummyTrans = False 
485          write = trans.response().write 
486          SL = self._CHEETAH__searchList 
487          _filter = self._CHEETAH__currentFilter 
488           
489          ######################################## 
490          ## START - generated method body 
491           
492          write('''<div class="narrow"> 
493   
494  <form id="query" action="" method="post"> 
495     
496    <fieldset> 
497      <legend>Standard Options</legend> 
498  ''') 
499          #  Auto captcha for this form, see if spambots are invalid anyway 
500          write('    ') 
501          _v = VFN(VFSL([locals()]+SL+[globals(), __builtin__],"inputs",True)["captcha"],"render",False)() # '$inputs["captcha"]render()' on line 133, col 5 
502          if _v is not None: write(_filter(_v, rawExpr='$inputs["captcha"]render()')) # from line 133, col 5. 
503          write('\n    <table>\n      ') 
504          _v = VFSL([locals()]+SL+[globals(), __builtin__],"form_row",False)("positives") # '$form_row("positives")' on line 135, col 7 
505          if _v is not None: write(_filter(_v, rawExpr='$form_row("positives")')) # from line 135, col 7. 
506          write('\n      ') 
507          _v = VFSL([locals()]+SL+[globals(), __builtin__],"form_row",False)("dataset") # '$form_row("dataset")' on line 136, col 7 
508          if _v is not None: write(_filter(_v, rawExpr='$form_row("dataset")')) # from line 136, col 7. 
509          write('\n      ') 
510          _v = VFSL([locals()]+SL+[globals(), __builtin__],"form_row",False)("delcode") # '$form_row("delcode")' on line 137, col 7 
511          if _v is not None: write(_filter(_v, rawExpr='$form_row("delcode")')) # from line 137, col 7. 
512          write(''' 
513      </table> 
514    </fieldset> 
515   
516    <fieldset> 
517      <legend> 
518        ''') 
519          _v = VFN(VFSL([locals()]+SL+[globals(), __builtin__],"inputs",True)["operation"],"render",False)("retrieval") # '$inputs["operation"].render("retrieval")' on line 143, col 7 
520          if _v is not None: write(_filter(_v, rawExpr='$inputs["operation"].render("retrieval")')) # from line 143, col 7. 
521          write(''' 
522      </legend> 
523      <table> 
524        ''') 
525          _v = VFSL([locals()]+SL+[globals(), __builtin__],"form_row",False)("limit") # '$form_row("limit")' on line 146, col 7 
526          if _v is not None: write(_filter(_v, rawExpr='$form_row("limit")')) # from line 146, col 7. 
527          write('\n      ') 
528          _v = VFSL([locals()]+SL+[globals(), __builtin__],"form_row",False)("mindate") # '$form_row("mindate")' on line 147, col 7 
529          if _v is not None: write(_filter(_v, rawExpr='$form_row("mindate")')) # from line 147, col 7. 
530          write('\n      ') 
531          _v = VFSL([locals()]+SL+[globals(), __builtin__],"form_row",False)("prevalence") # '$form_row("prevalence")' on line 148, col 7 
532          if _v is not None: write(_filter(_v, rawExpr='$form_row("prevalence")')) # from line 148, col 7. 
533          write('\n      ') 
534          _v = VFSL([locals()]+SL+[globals(), __builtin__],"form_row",False)("minscore") # '$form_row("minscore")' on line 149, col 7 
535          if _v is not None: write(_filter(_v, rawExpr='$form_row("minscore")')) # from line 149, col 7. 
536          write(''' 
537      </table> 
538    </fieldset> 
539   
540    <fieldset> 
541      <legend> 
542        ''') 
543          _v = VFN(VFSL([locals()]+SL+[globals(), __builtin__],"inputs",True)["operation"],"render",False)("validate") # '$inputs["operation"].render("validate")' on line 155, col 7 
544          if _v is not None: write(_filter(_v, rawExpr='$inputs["operation"].render("validate")')) # from line 155, col 7. 
545          write(''' 
546      </legend> 
547      <table> 
548        ''') 
549          _v = VFSL([locals()]+SL+[globals(), __builtin__],"form_row",False)("numnegs") # '$form_row("numnegs")' on line 158, col 7 
550          if _v is not None: write(_filter(_v, rawExpr='$form_row("numnegs")')) # from line 158, col 7. 
551          write(''' 
552      </table> 
553    </fieldset>  
554   
555    <p> 
556      <input type="submit"> 
557      <button type="button" id="fill_example">Example</button> 
558    </p> 
559   
560  </form> 
561   
562  </div> 
563  ''') 
564           
565          ######################################## 
566          ## END - generated method body 
567           
568          return _dummyTrans and trans.response().getvalue() or ""

569           
570   


571 -    def writeBody(self, **KWS):


572   
573   
574   
575          ## CHEETAH: main method generated for this template 
576          trans = KWS.get("trans") 
577          if (not trans and not self._CHEETAH__isBuffering and not callable(self.transaction)): 
578              trans = self.transaction # is None unless self.awake() was called 
579          if not trans: 
580              trans = DummyTransaction() 
581              _dummyTrans = True 
582          else: _dummyTrans = False 
583          write = trans.response().write 
584          SL = self._CHEETAH__searchList 
585          _filter = self._CHEETAH__currentFilter 
586           
587          ######################################## 
588          ## START - generated method body 
589           
590          write('\n') 
591          #  PARAMETERS 
592          # $inputs -- Form containing web browser input 
593          write(''' 
594   
595   
596   
597   
598   
599   
600   
601   
602   
603   
604   
605   
606   
607   
608  ''') 
609           
610          ######################################## 
611          ## END - generated method body 
612           
613          return _dummyTrans and trans.response().getvalue() or ""

614           
615      ################################################## 
616      ## CHEETAH GENERATED ATTRIBUTES 
617   
618   
619      _CHEETAH__instanceInitialized = False 
620   
621      _CHEETAH_version = __CHEETAH_version__ 
622   
623      _CHEETAH_versionTuple = __CHEETAH_versionTuple__ 
624   
625      _CHEETAH_genTime = __CHEETAH_genTime__ 
626   
627      _CHEETAH_genTimestamp = __CHEETAH_genTimestamp__ 
628   
629      _CHEETAH_src = __CHEETAH_src__ 
630   
631      _CHEETAH_srcLastModified = __CHEETAH_srcLastModified__ 
632   
633      _mainCheetahMethod_for_query= 'writeBody'

634   
635  ## END CLASS DEFINITION 
636   
637  if not hasattr(query, '_initCheetahAttributes'): 
638      templateAPIClass = getattr(query, '_CHEETAH_templateClass', Template) 
639      templateAPIClass._addCheetahPlumbingCodeToClass(query) 
640   
641   
642  # CHEETAH was developed by Tavis Rudd and Mike Orr 
643  # with code, advice and input from many other volunteers. 
644  # For more information visit http://www.CheetahTemplate.org/ 
645   
646  ################################################## 
647  ## if run from command line: 
648  if __name__ == '__main__': 
649      from Cheetah.TemplateCmdLineIface import CmdLineIface 
650      CmdLineIface(templateObj=query()).run() 
651
```

  


| Trees | Indices | Help | | MScanner | | --- | |
| --- | --- | --- | --- | --- |

|  |  |
| --- | --- |
| Generated by Epydoc 3.0beta1 on Fri Nov 23 09:13:23 2007 | http://epydoc.sourceforge.net |
