# Supplementary material for: MScanner: a classifier for retrieving Medline citations
Source: BMC Bioinformatics. 2008 Feb 19;9:108. doi: 10.1186/1471-2105-9-108 (PMC2263023; doi:10.1186/1471-2105-9-108)
Supplement: Additional file 3 — Source code for MScanner. mscanner-20071123.zip is a ZIP archive containing the Python 2.5 source code for MScanner, licensed under the GNU General Public License. It also contains API documentation in HTML format. Updated versions will be made available at . [file 1471-2105-9-108-S3.zip › mscanner/help/api/mscanner.htdocs.templates.query_logic-module.html]

xml version="1.0" encoding="ascii"?


mscanner.htdocs.templates.query\_logic


| Trees | Indices | Help | | MScanner | | --- | |
| --- | --- | --- | --- | --- |

|  |  |  |  |
| --- | --- | --- | --- |
| Package mscanner :: Package htdocs :: Package templates :: Module query\_logic | |  | | --- | | [hide private] | | [frames] | no frames] | |

# Module query\_logic

source code  
  
web.py handler for the query submission page  
  


---

**Author:**
Graham Poulter <http://graham.poulter.googlepages.com>

**Copyright:**
2007 Graham Poulter

**License:**
GPL


|  |  |  |  |
| --- | --- | --- | --- |
| |  |  | | --- | --- | | Classes | [hide private] | | |
|  | QueryPage  Submission form for queries or validation |


|  |  |  |  |
| --- | --- | --- | --- |
| |  |  | | --- | --- | | Functions | [hide private] | | |
|  | |  |  | | --- | --- | | parse\_pmids(pmids)  Parse a string into a list of integer PubMed IDs | source code | |
|  | |  |  | | --- | --- | | parse\_date(date\_code)  Convert YYYY/MM/DD date string to YYYYMMDD integer. | source code | |
|  | |  |  | | --- | --- | | date\_is\_valid(date\_code)  Must be a YYYY/MM/DD date string, before today | source code | |
|  | |  |  | | --- | --- | | task\_does\_not\_exist(dataset)  True if task does not exist in queue or output directory | source code | |
|  | |  |  | | --- | --- | | task\_exists(dataset)  True if task exists in queue or output directory | source code | |


|  |  |  |  |
| --- | --- | --- | --- |
| |  |  | | --- | --- | | Variables | [hide private] | | |
|  | delcode\_validator = `<mscanner.htdocs.forms.RegexValidator inst...`  Checks deletion code for valid format |
|  | dataset\_validator = `<mscanner.htdocs.forms.RegexValidator inst...`  Checks task name for valid format |
|  | QueryForm = `forms.Form(forms.Hidden("captcha", forms.Validator...`  Structure of the query form |
|  | form\_defaults = `{'captcha': 'orange', 'dataset': '', 'delcode'...`  Default values for the query form |


|  |  |  |  |
| --- | --- | --- | --- |
| |  |  | | --- | --- | | Variables Details | [hide private] | | |

|  |  |
| --- | --- |
| delcode\_validatorChecks deletion code for valid format   Value:  |  | | --- | | ``` forms.RegexValidator(r"^[ a-zA-Z0-9.;:_-]{0,10}$", "Should be 0-10 cha racters long, containing "+ "only letters, numbers and .;:,_- punctuat ion.") ``` | |

|  |  |
| --- | --- |
| dataset\_validatorChecks task name for valid format   Value:  |  | | --- | | ``` forms.RegexValidator(r"^[ a-zA-Z0-9.,;:_-]{1,30}$", "Should be 1-30 ch aracters long, containing "+ "only letters, numbers and .,;:_- punctua tion.") ``` | |

|  |  |
| --- | --- |
| QueryFormStructure of the query form   Value:  |  | | --- | | ``` forms.Form(forms.Hidden("captcha", forms.Validator(lambda x: x== "oran ge", "Should be the word 'orange'"), label= "Enter the word 'orange'") , forms.Textarea("positives", forms.Validator(lambda x: len(parse_pmid s(x)) > 0, "Should be numbers separated by line breaks"), label= "Inpu t Citations", rows= 3, cols= 10), forms.Textbox("dataset", dataset_val idator, forms.Validator(task_does_not_exist, "Task already exists"), l abel= "Task Name", size= 30), forms.Textbox("delcode", delcode_validat or, label= "Deletion Code", size= 8), forms.Checkbox("hidden", forms.c ... ``` | |

|  |  |
| --- | --- |
| form\_defaultsDefault values for the query form   Value:  |  | | --- | | ``` {'captcha': 'orange',  'dataset': '',  'delcode': '',  'hidden': False,  'limit': 1000,  'mindate': '0000/00/00',  'minscore': '0',  'numnegs': 50000, ... ``` | |

  


| Trees | Indices | Help | | MScanner | | --- | |
| --- | --- | --- | --- | --- |

|  |  |
| --- | --- |
| Generated by Epydoc 3.0beta1 on Fri Nov 23 09:13:20 2007 | http://epydoc.sourceforge.net |
