# Supplementary material for: MScanner: a classifier for retrieving Medline citations
Source: BMC Bioinformatics. 2008 Feb 19;9:108. doi: 10.1186/1471-2105-9-108 (PMC2263023; doi:10.1186/1471-2105-9-108)
Supplement: Additional file 3 — Source code for MScanner. mscanner-20071123.zip is a ZIP archive containing the Python 2.5 source code for MScanner, licensed under the GNU General Public License. It also contains API documentation in HTML format. Updated versions will be made available at . [file 1471-2105-9-108-S3.zip › mscanner/help/api/mscanner.htdocs.templates.query_logic-pysrc.html]

xml version="1.0" encoding="ascii"?


mscanner.htdocs.templates.query\_logic


| Trees | Indices | Help | | MScanner | | --- | |
| --- | --- | --- | --- | --- |

|  |  |  |  |
| --- | --- | --- | --- |
| Package mscanner :: Package htdocs :: Package templates :: Module query\_logic | |  | | --- | | [hide private] | | [frames] | no frames] | |

# Source Code for Module mscanner.htdocs.templates.query\_logic

```
  1  """web.py handler for the query submission page""" 
  2   
  3  __copyright__ = "2007 Graham Poulter" 
  4  __author__ = "Graham Poulter <http://graham.poulter.googlepages.com>" 
  5  __license__ = "GPL" 
  6   
  7  import web 
  8  import time 
  9  import md5 
 10  import sys 
 11   
 12  import query 
 13  from mscanner.htdocs import forms, queue 
 14  from mscanner.configuration import rc 
 15   
 16   


17 -def parse_pmids(pmids):


18      """Parse a string into a list of integer PubMed IDs""" 
 19      return [int(y) for y in pmids.split()]

 20   
 21   
 22  delcode_validator = forms.RegexValidator( 
 23      r"^[ a-zA-Z0-9.;:_-]{0,10}$",  
 24      "Should be 0-10 characters long, containing "+ 
 25      "only letters, numbers and .;:,_- punctuation.") 
 26  """Checks deletion code for valid format""" 
 27   
 28   
 29  dataset_validator = forms.RegexValidator( 
 30      r"^[ a-zA-Z0-9.,;:_-]{1,30}$", 
 31      "Should be 1-30 characters long, containing "+ 
 32      "only letters, numbers and .,;:_- punctuation.") 
 33  """Checks task name for valid format""" 
 34   
 35   
 36   


37 -def parse_date(date_code):


38      """Convert YYYY/MM/DD date string to YYYYMMDD integer.""" 
 39      year, month, day = date_code.split("/") 
 40      date = int("%04d%02d%02d" % (int(year),int(month),int(day))) 
 41      return date if date >= 19650101 else None

 42   
 43   


44 -def date_is_valid(date_code):


45      """Must be a YYYY/MM/DD date string, before today""" 
 46      try: 
 47          date = parse_date(date_code) 
 48          yesterday = int(time.strftime("%Y%m%d"))-1 
 49          return (date is None or date < yesterday) 
 50      except: 
 51          return False

 52   
 53   


54 -def task_does_not_exist(dataset):


55      """True if task does not exist in queue or output directory""" 
 56      return not task_exists(dataset)

 57   
 58   


59 -def task_exists(dataset):


60      """True if task exists in queue or output directory""" 
 61      return (rc.queue_path / dataset).isfile() or\ 
 62             (rc.web_report_dir / dataset).isdir()

 63   
 64   
 65  QueryForm = forms.Form( 
 66      forms.Hidden( 
 67          "captcha", 
 68          forms.Validator( 
 69              lambda x: x == "orange", "Should be the word 'orange'"), 
 70          label="Enter the word 'orange'"), 
 71       
 72      forms.Textarea( 
 73          "positives",  
 74          forms.Validator(lambda x: len(parse_pmids(x)) > 0, 
 75              "Should be numbers separated by line breaks"), 
 76          label="Input Citations", rows=3, cols=10), 
 77       
 78      forms.Textbox( 
 79          "dataset",  
 80          dataset_validator,  
 81          forms.Validator(task_does_not_exist, "Task already exists"), 
 82          label="Task Name", size=30), 
 83       
 84      forms.Textbox( 
 85          "delcode", delcode_validator, label="Deletion Code", size=8), 
 86       
 87      forms.Checkbox( 
 88          "hidden", forms.checkbox_validator, label="Hide output"), 
 89       
 90      forms.Textbox( 
 91          "limit",  
 92          forms.Validator(lambda x: 100 <= int(x) <= 10000, 
 93              "Should be between 100 and 10000."), 
 94          label="Result limit"), 
 95       
 96      forms.Textbox( 
 97          "mindate",  
 98          forms.Validator(date_is_valid,  
 99          "Date should be YYYY/MM/DD, and the day before yesterday at latest."), 
100          label="Minimum date", size=12), 
101   
102      forms.Textbox( 
103          "prevalence",  
104          forms.Validator(lambda x: x.strip() == "" or 1e-6 <= float(x) <= 0.1, 
105              "Should be empty, or a number between 0.000001 (10^-6) and 0.1"), 
106          label="Estimated prevalence", size=8), 
107       
108      forms.Textbox( 
109          "minscore",  
110          forms.Validator(lambda x: -1000 <= float(x) <= 1000, 
111              "Should be between -1000 and +1000."), 
112          label="Minimum score", size=8), 
113       
114      forms.Textbox( 
115          "numnegs",  
116          forms.Validator(lambda x: 100 <= int(x) <= 100000, 
117              "Should be between 100 and 100000."), 
118          label="Number of Negatives", size=8), 
119       
120      forms.Radio( 
121          "operation", 
122          [ ("retrieval", "Medline retrieval operation"),  
123            ("validate", "Cross validation operation") ], 
124          forms.Validator(lambda x: x in ["retrieval", "validate"],  
125                          "Invalid operation")), 
126  ) 
127  """Structure of the query form""" 
128   
129   
130  # Initial values to fill into the form (see queue.py for meanings) 
131  form_defaults = dict( 
132      captcha = "orange", 
133      delcode = "", 
134      dataset = "", 
135      hidden = False, 
136      limit = 1000, 
137      mindate = "0000/00/00", 
138      minscore = "0", 
139      numnegs = 50000, 
140      operation = "retrieval", 
141      positives = "", 
142      prevalence = "", 
143  ) 
144  """Default values for the query form""" 
145   
146   
147   


148 -class QueryPage:


149      """Submission form for queries or validation""" 
150       


151 -    def GET(self):


152          """Print the query form, filled with default values""" 
153          web.header('Content-Type', 'text/html; charset=utf-8')  
154          page = query.query() 
155          page.inputs = QueryForm() 
156          page.inputs.fill(form_defaults) 
157          print page

158   
159   


160 -    def POST(self):


161          """Submit the query form, maintains previous values""" 
162          web.header('Content-Type', 'text/html; charset=utf-8')  
163          qform = QueryForm() 
164          if qform.validates(web.input()): 
165              # Add a descriptor to the queue 
166              inputs = qform.d 
167              inputs.submitted = time.time() 
168              inputs.hidden = forms.ischecked(inputs.hidden) 
169              # MD5 hash the deletion code 
170              delcode_plain = inputs.delcode 
171              inputs.delcode = md5.new(delcode_plain).hexdigest() 
172              # Parse the date string to integer 
173              inputs.mindate = parse_date(inputs.mindate) 
174              # Check for default prevalence 
175              if inputs.prevalence.strip() == "": 
176                  inputs.prevalence = None  
177              queue.write_descriptor(rc.queue_path / inputs.dataset,  
178                                     parse_pmids(inputs.positives), inputs) 
179              # Show status page for the task 
180              time.sleep(0.05) # So we show up in the queue 
181              web.seeother("status?dataset=%s;delcode=%s" %  
182                           (inputs.dataset, web.urlquote(delcode_plain))) 
183          else: 
184              # Errors in the form, print it again 
185              page = query.query() 
186              page.queue = queue.QueueStatus(with_done=True) 
187              page.inputs = qform 
188              print page

189
```

  


| Trees | Indices | Help | | MScanner | | --- | |
| --- | --- | --- | --- | --- |

|  |  |
| --- | --- |
| Generated by Epydoc 3.0beta1 on Fri Nov 23 09:13:23 2007 | http://epydoc.sourceforge.net |
