# Supplementary material for: MScanner: a classifier for retrieving Medline citations
Source: BMC Bioinformatics. 2008 Feb 19;9:108. doi: 10.1186/1471-2105-9-108 (PMC2263023; doi:10.1186/1471-2105-9-108)
Supplement: Additional file 3 — Source code for MScanner. mscanner-20071123.zip is a ZIP archive containing the Python 2.5 source code for MScanner, licensed under the GNU General Public License. It also contains API documentation in HTML format. Updated versions will be made available at . [file 1471-2105-9-108-S3.zip › mscanner/help/api/mscanner.htdocs.templates.query_logic.QueryPage-class.html]

xml version="1.0" encoding="ascii"?


mscanner.htdocs.templates.query\_logic.QueryPage


| Trees | Indices | Help | | MScanner | | --- | |
| --- | --- | --- | --- | --- |

|  |  |  |  |
| --- | --- | --- | --- |
| Package mscanner :: Package htdocs :: Package templates :: Module query\_logic :: Class QueryPage | |  | | --- | | [hide private] | | [frames] | no frames] | |

# Class QueryPage

source code  
  
Submission form for queries or validation  
  


|  |  |  |  |
| --- | --- | --- | --- |
| |  |  | | --- | --- | | Instance Methods | [hide private] | | |
|  | |  |  | | --- | --- | | GET(self)  Print the query form, filled with default values | source code | |
|  | |  |  | | --- | --- | | POST(self)  Submit the query form, maintains previous values | source code | |

| Trees | Indices | Help | | MScanner | | --- | |
| --- | --- | --- | --- | --- |

|  |  |
| --- | --- |
| Generated by Epydoc 3.0beta1 on Fri Nov 23 09:13:21 2007 | http://epydoc.sourceforge.net |
