# Supplementary material for: MScanner: a classifier for retrieving Medline citations
Source: BMC Bioinformatics. 2008 Feb 19;9:108. doi: 10.1186/1471-2105-9-108 (PMC2263023; doi:10.1186/1471-2105-9-108)
Supplement: Additional file 3 — Source code for MScanner. mscanner-20071123.zip is a ZIP archive containing the Python 2.5 source code for MScanner, licensed under the GNU General Public License. It also contains API documentation in HTML format. Updated versions will be made available at . [file 1471-2105-9-108-S3.zip › mscanner/help/api/mscanner.htdocs.templates.status-pysrc.html]

xml version="1.0" encoding="ascii"?


mscanner.htdocs.templates.status


| Trees | Indices | Help | | MScanner | | --- | |
| --- | --- | --- | --- | --- |

|  |  |  |  |
| --- | --- | --- | --- |
| Package mscanner :: Package htdocs :: Package templates :: Module status | |  | | --- | | [hide private] | | [frames] | no frames] | |

# Source Code for Module mscanner.htdocs.templates.status

```
  1  #!/usr/bin/env python 
  2   
  3   
  4   
  5   
  6  ################################################## 
  7  ## DEPENDENCIES 
  8  import sys 
  9  import os 
 10  import os.path 
 11  from os.path import getmtime, exists 
 12  import time 
 13  import types 
 14  import __builtin__ 
 15  from Cheetah.Version import MinCompatibleVersion as RequiredCheetahVersion 
 16  from Cheetah.Version import MinCompatibleVersionTuple as RequiredCheetahVersionTuple 
 17  from Cheetah.Template import Template 
 18  from Cheetah.DummyTransaction import DummyTransaction 
 19  from Cheetah.NameMapper import NotFound, valueForName, valueFromSearchList, valueFromFrameOrSearchList 
 20  from Cheetah.CacheRegion import CacheRegion 
 21  import Cheetah.Filters as Filters 
 22  import Cheetah.ErrorCatchers as ErrorCatchers 
 23  from page import page 
 24   
 25  ################################################## 
 26  ## MODULE CONSTANTS 
 27  try: 
 28      True, False 
 29  except NameError: 
 30      True, False = (1==1), (1==0) 
 31  VFFSL=valueFromFrameOrSearchList 
 32  VFSL=valueFromSearchList 
 33  VFN=valueForName 
 34  currentTime=time.time 
 35  __CHEETAH_version__ = '2.0rc7' 
 36  __CHEETAH_versionTuple__ = (2, 0, 0, 'candidate', 7) 
 37  __CHEETAH_genTime__ = 1193401029.9449999 
 38  __CHEETAH_genTimestamp__ = 'Fri Oct 26 14:17:09 2007' 
 39  __CHEETAH_src__ = 'status.tmpl' 
 40  __CHEETAH_srcLastModified__ = 'Fri Oct 26 14:17:08 2007' 
 41  __CHEETAH_docstring__ = 'Autogenerated by CHEETAH: The Python-Powered Template Engine' 
 42   
 43  if __CHEETAH_versionTuple__ < RequiredCheetahVersionTuple: 
 44      raise AssertionError( 
 45        'This template was compiled with Cheetah version' 
 46        ' %s. Templates compiled before version %s must be recompiled.'%( 
 47           __CHEETAH_version__, RequiredCheetahVersion)) 
 48   
 49  ################################################## 
 50  ## CLASSES 
 51   


52 -class status(page):


53   
 54      ################################################## 
 55      ## CHEETAH GENERATED METHODS 
 56   
 57   


58 -    def __init__(self, *args, **KWs):


59   
 60          page.__init__(self, *args, **KWs) 
 61          if not self._CHEETAH__instanceInitialized: 
 62              cheetahKWArgs = {} 
 63              allowedKWs = 'searchList namespaces filter filtersLib errorCatcher'.split() 
 64              for k,v in KWs.items(): 
 65                  if k in allowedKWs: cheetahKWArgs[k] = v 
 66              self._initCheetahInstance(**cheetahKWArgs)

 67           
 68   


69 -    def title(self, **KWS):


70   
 71   
 72   
 73          ## CHEETAH: generated from #def title at line 9, col 1. 
 74          trans = KWS.get("trans") 
 75          if (not trans and not self._CHEETAH__isBuffering and not callable(self.transaction)): 
 76              trans = self.transaction # is None unless self.awake() was called 
 77          if not trans: 
 78              trans = DummyTransaction() 
 79              _dummyTrans = True 
 80          else: _dummyTrans = False 
 81          write = trans.response().write 
 82          SL = self._CHEETAH__searchList 
 83          _filter = self._CHEETAH__currentFilter 
 84           
 85          ######################################## 
 86          ## START - generated method body 
 87           
 88          write('MScanner Status Report\n') 
 89           
 90          ######################################## 
 91          ## END - generated method body 
 92           
 93          return _dummyTrans and trans.response().getvalue() or ""

 94           
 95   


96 -    def extraheaders(self, **KWS):


97   
 98   
 99   
100          ## CHEETAH: generated from #def extraheaders at line 13, col 1. 
101          trans = KWS.get("trans") 
102          if (not trans and not self._CHEETAH__isBuffering and not callable(self.transaction)): 
103              trans = self.transaction # is None unless self.awake() was called 
104          if not trans: 
105              trans = DummyTransaction() 
106              _dummyTrans = True 
107          else: _dummyTrans = False 
108          write = trans.response().write 
109          SL = self._CHEETAH__searchList 
110          _filter = self._CHEETAH__currentFilter 
111           
112          ######################################## 
113          ## START - generated method body 
114           
115          write('''<script type="text/javascript"> 
116      window.onload = function() { 
117          setTimeout(function() {window.location.reload()}, 10000); 
118      } 
119  </script> 
120  ''') 
121           
122          ######################################## 
123          ## END - generated method body 
124           
125          return _dummyTrans and trans.response().getvalue() or ""

126           
127   


128 -    def contents(self, **KWS):


129   
130   
131   
132          ## CHEETAH: generated from #def contents at line 21, col 1. 
133          trans = KWS.get("trans") 
134          if (not trans and not self._CHEETAH__isBuffering and not callable(self.transaction)): 
135              trans = self.transaction # is None unless self.awake() was called 
136          if not trans: 
137              trans = DummyTransaction() 
138              _dummyTrans = True 
139          else: _dummyTrans = False 
140          write = trans.response().write 
141          SL = self._CHEETAH__searchList 
142          _filter = self._CHEETAH__currentFilter 
143           
144          ######################################## 
145          ## START - generated method body 
146           
147          write('''<div class="narrow"> 
148   
149  <p>This page reloads every 10 seconds.</p> 
150   
151  ''') 
152          dataset = VFSL([locals()]+SL+[globals(), __builtin__],"inputs.d.dataset",True) 
153          write('\n') 
154          if VFSL([locals()]+SL+[globals(), __builtin__],"queue.running",True) is None: # generated from line 28, col 1 
155              write('    <p>There are no tasks waiting in the queue.</p>\n') 
156          elif VFSL([locals()]+SL+[globals(), __builtin__],"dataset",True) not in VFSL([locals()]+SL+[globals(), __builtin__],"queue",True): # generated from line 30, col 1 
157              write('    <p>The specified task <q>') 
158              _v = VFSL([locals()]+SL+[globals(), __builtin__],"dataset",True) # '$dataset' on line 31, col 30 
159              if _v is not None: write(_filter(_v, rawExpr='$dataset')) # from line 31, col 30. 
160              write('</q> was not found.</p>\n') 
161          write('\n') 
162          if VFSL([locals()]+SL+[globals(), __builtin__],"dataset",True) in VFSL([locals()]+SL+[globals(), __builtin__],"queue",True): # generated from line 34, col 1 
163              write('    ') 
164              _v = VFSL([locals()]+SL+[globals(), __builtin__],"statusblock",False)(VFSL([locals()]+SL+[globals(), __builtin__],"queue",True)[VFSL([locals()]+SL+[globals(), __builtin__],"dataset",True)], VFSL([locals()]+SL+[globals(), __builtin__],"queue",True)) # '$statusblock($queue[$dataset], $queue)' on line 35, col 5 
165              if _v is not None: write(_filter(_v, rawExpr='$statusblock($queue[$dataset], $queue)')) # from line 35, col 5. 
166              write('\n') 
167          write('\n') 
168          if VFSL([locals()]+SL+[globals(), __builtin__],"dataset",True) in VFSL([locals()]+SL+[globals(), __builtin__],"queue",True) and (VFN(VFSL([locals()]+SL+[globals(), __builtin__],"queue",True),"status",True)[VFSL([locals()]+SL+[globals(), __builtin__],"dataset",True)] == VFSL([locals()]+SL+[globals(), __builtin__],"queue.RUNNING",True)): # generated from line 38, col 1 
169              write('    ') 
170              _v = VFN(VFSL([locals()]+SL+[globals(), __builtin__],"inputs",True),"fill",False)({"dataset":"", "delcode":"", "operation":"delete"}) # '$inputs.fill({"dataset":"", "delcode":"", "operation":"delete"})' on line 39, col 5 
171              if _v is not None: write(_filter(_v, rawExpr='$inputs.fill({"dataset":"", "delcode":"", "operation":"delete"})')) # from line 39, col 5. 
172              write('\n') 
173          write('''     
174  <h2>Delete a task</h2> 
175  <form action="output" method="post"> 
176  ''') 
177          _v = VFN(VFSL([locals()]+SL+[globals(), __builtin__],"inputs",True),"render",False)() # '$inputs.render()' on line 44, col 1 
178          if _v is not None: write(_filter(_v, rawExpr='$inputs.render()')) # from line 44, col 1. 
179          write(''' 
180  <div><input type="submit" value="Delete task"></div> 
181  </form> 
182   
183  ''') 
184          # ### QUEUE CONTENTS ##### 
185          write('\n') 
186          if len(VFSL([locals()]+SL+[globals(), __builtin__],"queue.tasklist",True)) > 0: # generated from line 50, col 1 
187              write('''<h2>Tasks in the queue</h2> 
188   
189  <table> 
190  <thead> 
191    <tr> 
192      <th>Submitted at</th> 
193      <th>Type</th> 
194      <th>Name</th> 
195    </tr> 
196  </thead> 
197  <tbody> 
198  ''') 
199              for d in VFSL([locals()]+SL+[globals(), __builtin__],"queue.tasklist",True): # generated from line 62, col 3 
200                  write('  <tr>\n    <td class="timestamp">\n      ') 
201                  _v = VFN(VFSL([locals()]+SL+[globals(), __builtin__],"time",True),"strftime",False)("%Y/%m/%d %H:%M:%S GMT", VFN(VFSL([locals()]+SL+[globals(), __builtin__],"time",True),"gmtime",False)(VFSL([locals()]+SL+[globals(), __builtin__],"d.submitted",True))) # '$time.strftime("%Y/%m/%d %H:%M:%S GMT", $time.gmtime($d.submitted))' on line 65, col 7 
202                  if _v is not None: write(_filter(_v, rawExpr='$time.strftime("%Y/%m/%d %H:%M:%S GMT", $time.gmtime($d.submitted))')) # from line 65, col 7. 
203                  write('\n    </td>\n    <td class="operation">') 
204                  _v = VFSL([locals()]+SL+[globals(), __builtin__],"d.operation",True) # '$d.operation' on line 67, col 27 
205                  if _v is not None: write(_filter(_v, rawExpr='$d.operation')) # from line 67, col 27. 
206                  write('</td>\n    <td class="dataset">') 
207                  _v = VFSL([locals()]+SL+[globals(), __builtin__],"d.dataset",True) # '$d.dataset' on line 68, col 25 
208                  if _v is not None: write(_filter(_v, rawExpr='$d.dataset')) # from line 68, col 25. 
209                  write('</td>\n  </tr>\n') 
210              write('</tbody>\n</table>\n') 
211          write('\n') 
212          # ### LOG CONTENTS ##### 
213          write('\n') 
214          if len(VFSL([locals()]+SL+[globals(), __builtin__],"getVar",False)('log_lines', [])) > 0: # generated from line 77, col 1 
215              write('<h2>Contents of the log</h2>\n<pre>\n') 
216              _v = "".join(VFSL([locals()]+SL+[globals(), __builtin__],"log_lines",True)) 
217              if _v is not None: write(_filter(_v)) 
218              write('</pre>\n') 
219          write('\n</div><!--class=narrow-->\n') 
220           
221          ######################################## 
222          ## END - generated method body 
223           
224          return _dummyTrans and trans.response().getvalue() or ""

225           
226   


227 -    def writeBody(self, **KWS):


228   
229   
230   
231          ## CHEETAH: main method generated for this template 
232          trans = KWS.get("trans") 
233          if (not trans and not self._CHEETAH__isBuffering and not callable(self.transaction)): 
234              trans = self.transaction # is None unless self.awake() was called 
235          if not trans: 
236              trans = DummyTransaction() 
237              _dummyTrans = True 
238          else: _dummyTrans = False 
239          write = trans.response().write 
240          SL = self._CHEETAH__searchList 
241          _filter = self._CHEETAH__currentFilter 
242           
243          ######################################## 
244          ## START - generated method body 
245           
246          write('\n') 
247          #  PARAMETERS 
248          # $queue -- The QueueStatus object 
249          # $inputs -- Parameters passed by the web browser 
250          # $logcontents -- List of lines from the log file 
251          write(''' 
252   
253   
254  ''') 
255           
256          ######################################## 
257          ## END - generated method body 
258           
259          return _dummyTrans and trans.response().getvalue() or ""

260           
261      ################################################## 
262      ## CHEETAH GENERATED ATTRIBUTES 
263   
264   
265      _CHEETAH__instanceInitialized = False 
266   
267      _CHEETAH_version = __CHEETAH_version__ 
268   
269      _CHEETAH_versionTuple = __CHEETAH_versionTuple__ 
270   
271      _CHEETAH_genTime = __CHEETAH_genTime__ 
272   
273      _CHEETAH_genTimestamp = __CHEETAH_genTimestamp__ 
274   
275      _CHEETAH_src = __CHEETAH_src__ 
276   
277      _CHEETAH_srcLastModified = __CHEETAH_srcLastModified__ 
278   
279      _mainCheetahMethod_for_status= 'writeBody'

280   
281  ## END CLASS DEFINITION 
282   
283  if not hasattr(status, '_initCheetahAttributes'): 
284      templateAPIClass = getattr(status, '_CHEETAH_templateClass', Template) 
285      templateAPIClass._addCheetahPlumbingCodeToClass(status) 
286   
287   
288  # CHEETAH was developed by Tavis Rudd and Mike Orr 
289  # with code, advice and input from many other volunteers. 
290  # For more information visit http://www.CheetahTemplate.org/ 
291   
292  ################################################## 
293  ## if run from command line: 
294  if __name__ == '__main__': 
295      from Cheetah.TemplateCmdLineIface import CmdLineIface 
296      CmdLineIface(templateObj=status()).run() 
297
```

  


| Trees | Indices | Help | | MScanner | | --- | |
| --- | --- | --- | --- | --- |

|  |  |
| --- | --- |
| Generated by Epydoc 3.0beta1 on Fri Nov 23 09:13:25 2007 | http://epydoc.sourceforge.net |
