# Supplementary material for: MScanner: a classifier for retrieving Medline citations
Source: BMC Bioinformatics. 2008 Feb 19;9:108. doi: 10.1186/1471-2105-9-108 (PMC2263023; doi:10.1186/1471-2105-9-108)
Supplement: Additional file 3 — Source code for MScanner. mscanner-20071123.zip is a ZIP archive containing the Python 2.5 source code for MScanner, licensed under the GNU General Public License. It also contains API documentation in HTML format. Updated versions will be made available at . [file 1471-2105-9-108-S3.zip › mscanner/help/api/mscanner.htdocs.templates.status.status-class.html]

xml version="1.0" encoding="ascii"?


mscanner.htdocs.templates.status.status


| Trees | Indices | Help | | MScanner | | --- | |
| --- | --- | --- | --- | --- |

|  |  |  |  |
| --- | --- | --- | --- |
| Package mscanner :: Package htdocs :: Package templates :: Module status :: Class status | |  | | --- | | [hide private] | | [frames] | no frames] | |

# Class status

source code  
  

```
                 object --+                
                          |                
Cheetah.Servlet.BaseServlet --+            
                              |            
        Cheetah.Servlet.Servlet --+        
                                  |        
          Cheetah.Template.Template --+    
                                      |    
                              page.page --+
                                          |
                                         status
```

---


|  |  |  |  |
| --- | --- | --- | --- |
| |  |  | | --- | --- | | Nested Classes | [hide private] | | |
| **Inherited from `Cheetah.Template.Template`**: `NonNumericInputError`  **Inherited from `Cheetah.Template.Template`** (private): `_CHEETAH_cacheRegionClass`, `_CHEETAH_cacheStoreClass`, `_CHEETAH_compilerClass`, `_CHEETAH_defaultPreprocessorClass` | |


|  |  |  |  |
| --- | --- | --- | --- |
| |  |  | | --- | --- | | Instance Methods | [hide private] | | |
|  | |  |  | | --- | --- | | \_\_init\_\_(self, \*args, \*\*KWs)  a) compiles a new template OR b) instantiates an existing template. | source code | |
|  | |  |  | | --- | --- | | title(self, \*\*KWS) | source code | |
|  | |  |  | | --- | --- | | extraheaders(self, \*\*KWS) | source code | |
|  | |  |  | | --- | --- | | contents(self, \*\*KWS) | source code | |
|  | |  |  | | --- | --- | | writeBody(self, \*\*KWS) | source code | |
| **Inherited from `page.page`**: `__str__`, `body`, `contents_outer`, `doctype`, `footer`, `footer_text`, `head`, `header`, `header_text`, `html`, `respond`, `statusblock`, `stdheaders`, `topmenu`, `wintitle`  **Inherited from `Cheetah.Template.Template`**: `errorCatcher`, `generatedClassCode`, `generatedModuleCode`, `getCacheRegion`, `getCacheRegions`, `getFileContents`, `getVar`, `hasVar`, `i18n`, `refreshCache`, `runAsMainProgram`, `searchList`, `shutdown`, `varExists`, `webInput`  **Inherited from `Cheetah.Template.Template`** (private): `_compile`, `_createCacheRegion`, `_getCacheStore`, `_getCacheStoreIdPrefix`, `_getTemplateAPIClassForIncludeDirectiveCompilation`, `_handleCheetahInclude`, `_initCheetahInstance`  **Inherited from `Cheetah.Servlet.Servlet`**: `awake`, `serverSidePath`, `sleep`  **Inherited from `object`**: `__delattr__`, `__getattribute__`, `__hash__`, `__new__`, `__reduce__`, `__reduce_ex__`, `__repr__`, `__setattr__` | |


|  |  |  |  |
| --- | --- | --- | --- |
| |  |  | | --- | --- | | Class Methods | [hide private] | | |
| **Inherited from `Cheetah.Template.Template`**: `compile`, `subclass`  **Inherited from `Cheetah.Template.Template`** (private): `_addCheetahPlumbingCodeToClass`, `_getCompilerClass`, `_getCompilerSettings`, `_normalizePreprocessorArg`, `_normalizePreprocessorSettings`, `_preprocessSource`, `_updateSettingsWithPreprocessTokens` | |


|  |  |  |  |
| --- | --- | --- | --- |
| |  |  | | --- | --- | | Class Variables | [hide private] | | |
|  | \_CHEETAH\_\_instanceInitialized = `False` |
|  | \_CHEETAH\_version = `'2.0rc7'` |
|  | \_CHEETAH\_versionTuple = `(2, 0, 0, 'candidate', 7)` |
|  | \_CHEETAH\_genTime = `1193401029.94` |
|  | \_CHEETAH\_genTimestamp = `'Fri Oct 26 14:17:09 2007'` |
|  | \_CHEETAH\_src = `'status.tmpl'` |
|  | \_CHEETAH\_srcLastModified = `'Fri Oct 26 14:17:08 2007'` |
|  | \_mainCheetahMethod\_for\_status = `'writeBody'` |
| **Inherited from `page.page`**: `base`  **Inherited from `page.page`** (private): `_mainCheetahMethod_for_page`  **Inherited from `Cheetah.Template.Template`** (private): `_CHEETAH_cacheCompilationResults`, `_CHEETAH_cacheDirForModuleFiles`, `_CHEETAH_cacheModuleFilesForTracebacks`, `_CHEETAH_cacheStore`, `_CHEETAH_cacheStoreIdPrefix`, `_CHEETAH_compileCache`, `_CHEETAH_compileLock`, `_CHEETAH_compilerSettings`, `_CHEETAH_defaultBaseclassForTemplates`, `_CHEETAH_defaultClassNameForTemplates`, `_CHEETAH_defaultMainMethodName`, `_CHEETAH_defaultMainMethodNameForTemplates`, `_CHEETAH_defaultModuleGlobalsForTemplates`, `_CHEETAH_defaultModuleNameForTemplates`, `_CHEETAH_generatedModuleCode`, `_CHEETAH_keepRefToGeneratedCode`, `_CHEETAH_preprocessors`, `_CHEETAH_requiredCheetahClassAttributes`, `_CHEETAH_requiredCheetahClassMethods`, `_CHEETAH_requiredCheetahMethods`, `_CHEETAH_useCompilationCache`  **Inherited from `Cheetah.Servlet.Servlet`**: `application`, `request`, `session`, `transaction`  **Inherited from `Cheetah.Servlet.BaseServlet`** (private): `_reusable`, `_threadSafe` | |


|  |  |  |  |
| --- | --- | --- | --- |
| |  |  | | --- | --- | | Properties | [hide private] | | |
| **Inherited from `object`**: `__class__` | |


|  |  |  |  |
| --- | --- | --- | --- |
| |  |  | | --- | --- | | Method Details | [hide private] | | |

|  |  |  |
| --- | --- | --- |
| |  |  | | --- | --- | | \_\_init\_\_(self, \*args, \*\*KWs)  *(Constructor)* | source code |  ``` a) compiles a new template OR b) instantiates an existing template.  Read this docstring carefully as there are two distinct usage patterns. You should also read this class' main docstring.  a) to compile a new template:      t = Template(source=aSourceString)          # or       t = Template(file='some/path')          # or       t = Template(file=someFileObject)          # or      namespaces = [{'foo':'bar'}]                     t = Template(source=aSourceString, namespaces=namespaces)          # or       t = Template(file='some/path', namespaces=namespaces)       print t       b) to create an instance of an existing, precompiled template class:      ## i) first you need a reference to a compiled template class:      tclass = Template.compile(source=src) # or just Template.compile(src)          # or       tclass = Template.compile(file='some/path')          # or       tclass = Template.compile(file=someFileObject)          # or       # if you used the command line compiler or have Cheetah's ImportHooks      # installed your template class is also available via Python's      # standard import mechanism:      from ACompileTemplate import AcompiledTemplate as tclass            ## ii) then you create an instance      t = tclass(namespaces=namespaces)          # or       t = tclass(namespaces=namespaces, filter='RawOrEncodedUnicode')      print t  Arguments:   for usage pattern a)                If you are compiling a new template, you must provide either a     'source' or 'file' arg, but not both:                 - source (string or None)       - file (string path, file-like object, or None)      Optional args (see below for more) :       - compilerSettings        Default: Template._CHEETAH_compilerSettings=None                a dictionary of settings to override those defined in        DEFAULT_COMPILER_SETTINGS.  See        Cheetah.Template.DEFAULT_COMPILER_SETTINGS and the Users' Guide        for details.      You can pass the source arg in as a positional arg with this usage     pattern.  Use keywords for all other args.               for usage pattern b)     Do not use positional args with this usage pattern, unless your     template subclasses something other than Cheetah.Template and you     want to pass positional args to that baseclass.  E.g.:       dictTemplate = Template.compile('hello $name from $caller', baseclass=dict)       tmplvars = dict(name='world', caller='me')       print dictTemplate(tmplvars)     This usage requires all Cheetah args to be passed in as keyword args.    optional args for both usage patterns:      - namespaces (aka 'searchList')       Default: None              an optional list of namespaces (dictionaries, objects, modules,       etc.) which Cheetah will search through to find the variables       referenced in $placeholders.        If you provide a single namespace instead of a list, Cheetah will       automatically convert it into a list.                NOTE: Cheetah does NOT force you to use the namespaces search list       and related features.  It's on by default, but you can turn if off       using the compiler settings useSearchList=False or       useNameMapper=False.               - filter        Default: 'EncodeUnicode'                Which filter should be used for output filtering. This should        either be a string which is the name of a filter in the        'filtersLib' or a subclass of Cheetah.Filters.Filter. . See the        Users' Guide for more details.       - filtersLib        Default: Cheetah.Filters                A module containing subclasses of Cheetah.Filters.Filter. See the        Users' Guide for more details.        - errorCatcher        Default: None         This is a debugging tool. See the Users' Guide for more details.        Do not use this or the #errorCatcher diretive with live        production systems.    Do NOT mess with the args _globalSetVars or _preBuiltSearchList! ```   Overrides: page.page.\_\_init\_\_ |

|  |  |  |
| --- | --- | --- |
| |  |  | | --- | --- | | title(self, \*\*KWS) | source code |   Overrides: page.page.title |

|  |  |  |
| --- | --- | --- |
| |  |  | | --- | --- | | extraheaders(self, \*\*KWS) | source code |   Overrides: page.page.extraheaders |

|  |  |  |
| --- | --- | --- |
| |  |  | | --- | --- | | contents(self, \*\*KWS) | source code |   Overrides: page.page.contents |

  


| Trees | Indices | Help | | MScanner | | --- | |
| --- | --- | --- | --- | --- |

|  |  |
| --- | --- |
| Generated by Epydoc 3.0beta1 on Fri Nov 23 09:13:21 2007 | http://epydoc.sourceforge.net |
