# Supplementary material for: MScanner: a classifier for retrieving Medline citations
Source: BMC Bioinformatics. 2008 Feb 19;9:108. doi: 10.1186/1471-2105-9-108 (PMC2263023; doi:10.1186/1471-2105-9-108)
Supplement: Additional file 3 — Source code for MScanner. mscanner-20071123.zip is a ZIP archive containing the Python 2.5 source code for MScanner, licensed under the GNU General Public License. It also contains API documentation in HTML format. Updated versions will be made available at . [file 1471-2105-9-108-S3.zip › mscanner/help/api/mscanner.htdocs.templates.status_logic-module.html]

xml version="1.0" encoding="ascii"?


mscanner.htdocs.templates.status\_logic


| Trees | Indices | Help | | MScanner | | --- | |
| --- | --- | --- | --- | --- |

|  |  |  |  |
| --- | --- | --- | --- |
| Package mscanner :: Package htdocs :: Package templates :: Module status\_logic | |  | | --- | | [hide private] | | [frames] | no frames] | |

# Module status\_logic

source code  
  
web.py handler for the status page  
  


---

**Author:**
Graham Poulter <http://graham.poulter.googlepages.com>

**Copyright:**
2007 Graham Poulter

**License:**
GPL


|  |  |  |  |
| --- | --- | --- | --- |
| |  |  | | --- | --- | | Classes | [hide private] | | |
|  | StatusPage  Lists the current status of MScanner and a given task. |


|  |  |  |  |
| --- | --- | --- | --- |
| |  |  | | --- | --- | | Variables | [hide private] | | |
|  | StatusForm = `forms.Form(forms.Hidden("operation", forms.Valida...`  Structure for the delete-this-task form on the status page |


|  |  |  |  |
| --- | --- | --- | --- |
| |  |  | | --- | --- | | Variables Details | [hide private] | | |

|  |  |
| --- | --- |
| StatusFormStructure for the delete-this-task form on the status page   Value:  |  | | --- | | ``` forms.Form(forms.Hidden("operation", forms.Validator(lambda x: x== "de lete", "Invalid operation")), forms.Textbox("dataset", query_logic.dat aset_validator, forms.Validator(query_logic.task_exists, "Task does no t exist"), label= "Task name"), forms.Textbox("delcode", query_logic.d elcode_validator, label= "Deletion code"),) ``` | |

  


| Trees | Indices | Help | | MScanner | | --- | |
| --- | --- | --- | --- | --- |

|  |  |
| --- | --- |
| Generated by Epydoc 3.0beta1 on Fri Nov 23 09:13:20 2007 | http://epydoc.sourceforge.net |
