# Supplementary material for: MScanner: a classifier for retrieving Medline citations
Source: BMC Bioinformatics. 2008 Feb 19;9:108. doi: 10.1186/1471-2105-9-108 (PMC2263023; doi:10.1186/1471-2105-9-108)
Supplement: Additional file 3 — Source code for MScanner. mscanner-20071123.zip is a ZIP archive containing the Python 2.5 source code for MScanner, licensed under the GNU General Public License. It also contains API documentation in HTML format. Updated versions will be made available at . [file 1471-2105-9-108-S3.zip › mscanner/help/api/mscanner.htdocs.templates.status_logic-pysrc.html]

xml version="1.0" encoding="ascii"?


mscanner.htdocs.templates.status\_logic


| Trees | Indices | Help | | MScanner | | --- | |
| --- | --- | --- | --- | --- |

|  |  |  |  |
| --- | --- | --- | --- |
| Package mscanner :: Package htdocs :: Package templates :: Module status\_logic | |  | | --- | | [hide private] | | [frames] | no frames] | |

# Source Code for Module mscanner.htdocs.templates.status\_logic

```
 1  """web.py handler for the status page""" 
 2   
 3  __copyright__ = "2007 Graham Poulter" 
 4  __author__ = "Graham Poulter <http://graham.poulter.googlepages.com>" 
 5  __license__ = "GPL" 
 6   
 7  import web 
 8   
 9  import status, query_logic 
10  from mscanner.htdocs import forms, queue 
11  from mscanner.configuration import rc 
12   
13   
14  StatusForm = forms.Form( 
15      forms.Hidden( 
16          "operation", 
17          forms.Validator(lambda x: x == "delete", "Invalid operation")), 
18       
19      forms.Textbox( 
20          "dataset", 
21          query_logic.dataset_validator, 
22          forms.Validator(query_logic.task_exists, "Task does not exist"), 
23          label="Task name" 
24          ), 
25       
26      forms.Textbox( 
27          "delcode", 
28          query_logic.delcode_validator, 
29          label="Deletion code" 
30          ), 
31  ) 
32  """Structure for the delete-this-task form on the status page""" 
33   
34   
35   


36 -class StatusPage:


37      """Lists the current status of MScanner and a given task. 
38       
39      If the dataset and delcode parameters are given over the web, 
40      it provides a form for deleting the specified task. 
41      """ 
42       


43 -    def GET(self):


44          """Print the status page""" 
45          web.header('Content-Type', 'text/html; charset=utf-8')  
46          page = status.status() 
47          page.queue = queue.QueueStatus() 
48          page.log_lines = rc.logfile.lines()[-30:] 
49          page.inputs = StatusForm() 
50          dataset = "" # The task to print the status for 
51          if page.queue.running is not None: 
52              dataset = page.queue.running.dataset 
53          page.inputs.fill({"dataset":dataset, "delcode":"", "operation":"delete"}) 
54          # Have we been given a dataset to print status for? 
55          web_inputs = web.input() 
56          if "dataset" in web_inputs: 
57              web_inputs.operation = "delete" 
58              if "delcode" not in web_inputs: 
59                  web_inputs.delcode = ""  
60              page.inputs.validates(web_inputs) 
61          print page

62
```

  


| Trees | Indices | Help | | MScanner | | --- | |
| --- | --- | --- | --- | --- |

|  |  |
| --- | --- |
| Generated by Epydoc 3.0beta1 on Fri Nov 23 09:13:25 2007 | http://epydoc.sourceforge.net |
