# Supplementary material for: MScanner: a classifier for retrieving Medline citations
Source: BMC Bioinformatics. 2008 Feb 19;9:108. doi: 10.1186/1471-2105-9-108 (PMC2263023; doi:10.1186/1471-2105-9-108)
Supplement: Additional file 3 — Source code for MScanner. mscanner-20071123.zip is a ZIP archive containing the Python 2.5 source code for MScanner, licensed under the GNU General Public License. It also contains API documentation in HTML format. Updated versions will be made available at . [file 1471-2105-9-108-S3.zip › mscanner/help/api/mscanner.htdocs.testing-module.html]

xml version="1.0" encoding="ascii"?


mscanner.htdocs.testing


| Trees | Indices | Help | | MScanner | | --- | |
| --- | --- | --- | --- | --- |

|  |  |  |  |
| --- | --- | --- | --- |
| Package mscanner :: Package htdocs :: Module testing | |  | | --- | | [hide private] | | [frames] | no frames] | |

# Module testing

source code  
  

Simple tests using the web.py framework

Usage:

```
   python testing.py
```

Which starts the built-in web.py server on localhost:8080  
  


|  |  |  |  |
| --- | --- | --- | --- |
| |  |  | | --- | --- | | Classes | [hide private] | | |
|  | HelloPage  Simple page, e.g. |
|  | FormPage  Form testing page, on http://localhost:8080/form |


|  |  |  |  |
| --- | --- | --- | --- |
| |  |  | | --- | --- | | Functions | [hide private] | | |
|  | |  |  | | --- | --- | | pformat(obj)  Nicely format any python object | source code | |


|  |  |  |  |
| --- | --- | --- | --- |
| |  |  | | --- | --- | | Variables | [hide private] | | |
|  | urls = `('/hello/(.*)', 'HelloPage', '/form', 'FormPage')` |
|  | form\_template = `'\n<html>\n<head>\n<title>Test Form</title\n<s...` |
|  | TestForm = `forms.Form(forms.Textbox('text', forms.Validator(la...` |


|  |  |  |  |
| --- | --- | --- | --- |
| |  |  | | --- | --- | | Variables Details | [hide private] | | |

|  |  |
| --- | --- |
| form\_template   Value:  |  | | --- | | ``` ''' <html> <head> <title>Test Form</title <style type="text/css"> th { text-align: left; background-color: #EEEEEE; } tr.error { background-color: #FFEEEE; } </style> ... ``` | |

|  |  |
| --- | --- |
| TestForm   Value:  |  | | --- | | ``` forms.Form(forms.Textbox('text', forms.Validator(lambda x: len(x) < 3,  "Must be shorter than 3"), pre= "Before", post= "After", label= "Text  input", id= "different_id", class_= "aclass", size= 8), forms.Passwor d("password", label= "Password"), forms.Checkbox("checkbox", forms.che ckbox_validator, label= "Checkbox"), forms.Hidden("hidden", value= "no wai", label= "Hidden value"), forms.File("file", label= "Pick a file") , forms.Button("somebutton", label= "A button"), forms.Textarea("texta rea", label= "A text area"), forms.Dropdown("dropdown", ("X", "Y", "Z" ... ``` | |

  


| Trees | Indices | Help | | MScanner | | --- | |
| --- | --- | --- | --- | --- |

|  |  |
| --- | --- |
| Generated by Epydoc 3.0beta1 on Fri Nov 23 09:13:20 2007 | http://epydoc.sourceforge.net |
