# Supplementary material for: MScanner: a classifier for retrieving Medline citations
Source: BMC Bioinformatics. 2008 Feb 19;9:108. doi: 10.1186/1471-2105-9-108 (PMC2263023; doi:10.1186/1471-2105-9-108)
Supplement: Additional file 3 — Source code for MScanner. mscanner-20071123.zip is a ZIP archive containing the Python 2.5 source code for MScanner, licensed under the GNU General Public License. It also contains API documentation in HTML format. Updated versions will be made available at . [file 1471-2105-9-108-S3.zip › mscanner/help/api/mscanner.htdocs.testing-pysrc.html]

xml version="1.0" encoding="ascii"?


mscanner.htdocs.testing


| Trees | Indices | Help | | MScanner | | --- | |
| --- | --- | --- | --- | --- |

|  |  |  |  |
| --- | --- | --- | --- |
| Package mscanner :: Package htdocs :: Module testing | |  | | --- | | [hide private] | | [frames] | no frames] | |

# Source Code for Module mscanner.htdocs.testing

```
 1  #!/export/home/medscan/local32/bin/python2.5 
 2   
 3  """Simple tests using the web.py framework 
 4   
 5  Usage:: 
 6      python testing.py 
 7   
 8  Which starts the built-in web.py server on localhost:8080 
 9  """ 
10   
11  import sys 
12  sys.path.insert(0,"/export/home/medscan/source") 
13  from mscanner import configuration 
14   
15  import web 
16  web.webapi.internalerror = web.debugerror 
17  import forms 
18  import pprint 
19   


20 -def pformat(obj):


21      """Nicely format any python object""" 
22      return web.websafe(pprint.pformat(obj))

23   
24  urls = ( 
25      '/hello/(.*)', 'HelloPage', 
26      '/form', 'FormPage',) 
27   


28 -class HelloPage:


29      """Simple page, e.g. http://localhost:8080/hello/joe?times=5""" 


30 -    def GET(self, name):


31          i = web.input(times=1) 
32          if not name: 
33              name = 'world' 
34          for c in range(int(i.times)): 
35              print 'Hello,', name+'!'

36   
37  form_template = """ 
38  <html> 
39  <head> 
40  <title>Test Form</title 
41  <style type="text/css"> 
42  th { text-align: left; background-color: #EEEEEE; } 
43  tr.error { background-color: #FFEEEE; } 
44  </style> 
45  </head> 
46  <body> 
47  <p>%s</p> 
48  <form action="" method="post"> 
49  %s 
50  <p><input type="submit"></p> 
51  </form> 
52  </body> 
53  </html> 
54  """ 
55              
56  TestForm = forms.Form( 
57  forms.Textbox( 
58      'text',  
59      forms.Validator(lambda x: len(x) < 3, "Must be shorter than 3"), 
60      pre="Before", post="After", label="Text input",  
61      id="different_id", class_="aclass", size=8), 
62  forms.Password("password", label="Password"), 
63  forms.Checkbox("checkbox", forms.checkbox_validator, label="Checkbox"), 
64  forms.Hidden("hidden", value="nowai", label="Hidden value"), 
65  forms.File("file", label="Pick a file"), 
66  forms.Button("somebutton", label="A button"), 
67  forms.Textarea("textarea", label="A text area"), 
68  forms.Dropdown("dropdown", ("X","Y","Z"), label="A dropdown"), 
69  forms.Radio( 
70      'radio',  
71      [ ("a", "A"), ("b", "B"), ("c", "C")], 
72      forms.Validator(lambda x: x in ["a","b"], "Must choose a or b"), 
73      label="Radio buttons"), 
74  )     


75 -class FormPage:


76      """Form testing page, on http://localhost:8080/form"""     
77       


78 -    def GET(self):


79          form = TestForm() 
80          print form_template % ("", form.render())

81       


82 -    def POST(self):


83          input = web.input() 
84          form = TestForm(input) 
85          print form_template % ( 
86              pformat(input)+"<br>"+pformat(form.d), form.render())

87   
88  if __name__ == "__main__": 
89      try: 
90          web.run(urls, globals()) 
91      except KeyboardInterrupt: 
92          pass 
93
```

  


| Trees | Indices | Help | | MScanner | | --- | |
| --- | --- | --- | --- | --- |

|  |  |
| --- | --- |
| Generated by Epydoc 3.0beta1 on Fri Nov 23 09:13:23 2007 | http://epydoc.sourceforge.net |
