# Supplementary material for: MScanner: a classifier for retrieving Medline citations
Source: BMC Bioinformatics. 2008 Feb 19;9:108. doi: 10.1186/1471-2105-9-108 (PMC2263023; doi:10.1186/1471-2105-9-108)
Supplement: Additional file 3 — Source code for MScanner. mscanner-20071123.zip is a ZIP archive containing the Python 2.5 source code for MScanner, licensed under the GNU General Public License. It also contains API documentation in HTML format. Updated versions will be made available at . [file 1471-2105-9-108-S3.zip › mscanner/help/api/mscanner.medline-module.html]

xml version="1.0" encoding="ascii"?


mscanner.medline


| Trees | Indices | Help | | MScanner | | --- | |
| --- | --- | --- | --- | --- |

|  |  |  |  |
| --- | --- | --- | --- |
| Package mscanner :: Package medline | |  | | --- | | [hide private] | | [frames] | no frames] | |

# Package medline

source code  
  
Modules for creating, reading and updating the MScanner representation
of Medline  
  


|  |  |  |  |
| --- | --- | --- | --- |
| |  |  | | --- | --- | | Submodules | [hide private] | | |
| - **mscanner.medline.Article**: *Provides the Article class* - **mscanner.medline.Databases**: *For consumers of the database, this opens FeatureDatabase, FeatureMapping and the article list* - **mscanner.medline.FeatureDatabase**: *Maps PubMed IDs to feature vectors* - **mscanner.medline.FeatureMapping**: *Provides a mapping between features and integer IDs* - **mscanner.medline.FeatureStream**: *A class for rapid iteration over the records in Medline.* - **mscanner.medline.FileTracker**: *A simple persistant set of file names* - **mscanner.medline.MedlineCache**: *For updating the databases of articles and features* - **mscanner.medline.Shelf**: *Persistent shelf backed by Berkeley DB* |

  


| Trees | Indices | Help | | MScanner | | --- | |
| --- | --- | --- | --- | --- |

|  |  |
| --- | --- |
| Generated by Epydoc 3.0beta1 on Fri Nov 23 09:13:20 2007 | http://epydoc.sourceforge.net |
