# Supplementary material for: MScanner: a classifier for retrieving Medline citations
Source: BMC Bioinformatics. 2008 Feb 19;9:108. doi: 10.1186/1471-2105-9-108 (PMC2263023; doi:10.1186/1471-2105-9-108)
Supplement: Additional file 3 — Source code for MScanner. mscanner-20071123.zip is a ZIP archive containing the Python 2.5 source code for MScanner, licensed under the GNU General Public License. It also contains API documentation in HTML format. Updated versions will be made available at . [file 1471-2105-9-108-S3.zip › mscanner/help/api/mscanner.medline.Article-pysrc.html]

xml version="1.0" encoding="ascii"?


mscanner.medline.Article


| Trees | Indices | Help | | MScanner | | --- | |
| --- | --- | --- | --- | --- |

|  |  |  |  |
| --- | --- | --- | --- |
| Package mscanner :: Package medline :: Module Article | |  | | --- | | [hide private] | | [frames] | no frames] | |

# Source Code for Module mscanner.medline.Article

```
  1  """Provides the Article class""" 
  2   
  3   
  4  __copyright__ = "2007 Graham Poulter" 
  5  __author__ = "Graham Poulter <http://graham.poulter.googlepages.com>" 
  6  __license__ = """This program is free software: you can redistribute it and/or 
  7  modify it under the terms of the GNU General Public License as published by the 
  8  Free Software Foundation, either version 3 of the License, or (at your option) 
  9  any later version. 
 10   
 11  This program is distributed in the hope that it will be useful, but WITHOUT ANY 
 12  WARRANTY; without even the implied warranty of MERCHANTABILITY or FITNESS FOR A 
 13  PARTICULAR PURPOSE. See the GNU General Public License for more details. 
 14   
 15  You should have received a copy of the GNU General Public License along with 
 16  this program. If not, see <http://www.gnu.org/licenses/>.""" 
 17   
 18   


19 -class Article:


20      """Database record for a Medline citation. 
 21       
 22      The article is converted to a tuple which stored in a Berkeley DB 
 23      indexed by PubMed ID. 
 24   
 25      @ivar pmid: PubMed ID of the article (int) 
 26   
 27      @ivar title: Title of the article (string) 
 28   
 29      @ivar abstract: Abstract of the article (sring) 
 30   
 31      @ivar journal: Medline abbreviated journal title (string) 
 32   
 33      @ivar issn: Journal ISSN code (string) 
 34   
 35      @ivar date_completed: (year,month,day) as integers 
 36       
 37      @ivar year: Year of publication (int) 
 38       
 39      @ivar meshterms: MeSH as a list of (descriptor, qual, qual, ...) tuples 
 40   
 41      @ivar authors: Authors as a list of (initials, lastname) tuples of  
 42      """ 
 43       


44 -    def __init__(self, 
 45                   pmid=None, 
 46                   title=None, 
 47                   abstract=None, 
 48                   journal=None, 
 49                   issn=None, 
 50                   date_completed=None, 
 51                   pubyear=None, 
 52                   meshterms=None, 
 53                   authors=None):


54          """Constructor, where parameters set instance variables.""" 
 55          self.pmid = pmid 
 56          self.title = title 
 57          self.abstract = abstract 
 58          self.journal = journal 
 59          self.issn = issn 
 60          self.date_completed = date_completed 
 61          self.pubyear = pubyear 
 62          self.meshterms = meshterms if meshterms else [] 
 63          self.authors = authors if authors else []

 64   
 65   


66 -    def __repr__(self):


67          """Evaluatable representation of the object""" 
 68          import pprint as pp 
 69          s = ["Article("] 
 70          for k,v in self.__dict__.iteritems(): 
 71              s.append("%s=%s" % (k, pp.pformat(v))) 
 72          s[-1] += ")" 
 73          return "\n".join(s)

 74   
 75   
 76      @staticmethod 


77 -    def parse_medline_xml(stream):


78          """Generate Article objects by parsing a Medline XML file 
 79           
 80          @param stream: File-like object of MedlineCitation XML 
 81           
 82          @return: Iteratation over parsed Article objects 
 83          """ 
 84          import xml.etree.cElementTree as ET 
 85          context = ET.iterparse(stream, events=("start", "end")) 
 86          context = iter(context) 
 87          event, root = context.next() 
 88          for event, record in context: 
 89              if event == "end" and record.tag == "MedlineCitation": 
 90                  if record.get("Status") == "MEDLINE": 
 91                      r = Article() 
 92                      r.pmid = int(record.findtext("PMID")) 
 93                      dc = record.find("DateCompleted") 
 94                      r.date_completed = ( 
 95                          int(dc.findtext("Year")), 
 96                          int(dc.findtext("Month")), 
 97                          int(dc.findtext("Day"))) 
 98                      art = record.find("Article") 
 99                      r.issn = art.findtext("Journal/ISSN") 
100                      r.pubyear = art.findtext("Journal/JournalIssue/PubDate/Year") 
101                      if r.pubyear is not None: 
102                          r.pubyear = int(r.pubyear) 
103                      r.title = art.findtext("ArticleTitle") 
104                      r.abstract = art.findtext("Abstract/AbstractText") 
105                      r.authors = [(a.findtext("Initials"), a.findtext("LastName"))  
106                                        for a in art.findall("AuthorList/Author")] 
107                      r.journal = record.findtext("MedlineJournalInfo/MedlineTA") 
108                      for heading in record.findall("MeshHeadingList/MeshHeading"): 
109                          descriptor = heading.findtext("DescriptorName") 
110                          quals = [ q.text for q in heading.findall("QualifierName") ] 
111                          r.meshterms.append(tuple([descriptor] + quals)) 
112                      yield r 
113                  root.clear()

114
```

  


| Trees | Indices | Help | | MScanner | | --- | |
| --- | --- | --- | --- | --- |

|  |  |
| --- | --- |
| Generated by Epydoc 3.0beta1 on Fri Nov 23 09:13:24 2007 | http://epydoc.sourceforge.net |
