# Supplementary material for: MScanner: a classifier for retrieving Medline citations
Source: BMC Bioinformatics. 2008 Feb 19;9:108. doi: 10.1186/1471-2105-9-108 (PMC2263023; doi:10.1186/1471-2105-9-108)
Supplement: Additional file 3 — Source code for MScanner. mscanner-20071123.zip is a ZIP archive containing the Python 2.5 source code for MScanner, licensed under the GNU General Public License. It also contains API documentation in HTML format. Updated versions will be made available at . [file 1471-2105-9-108-S3.zip › mscanner/help/api/mscanner.medline.Article.Article-class.html]

xml version="1.0" encoding="ascii"?


mscanner.medline.Article.Article


| Trees | Indices | Help | | MScanner | | --- | |
| --- | --- | --- | --- | --- |

|  |  |  |  |
| --- | --- | --- | --- |
| Package mscanner :: Package medline :: Module Article :: Class Article | |  | | --- | | [hide private] | | [frames] | no frames] | |

# Class Article

source code  
  

Database record for a Medline citation.

The article is converted to a tuple which stored in a Berkeley DB
indexed by PubMed ID.  
  


|  |  |  |  |
| --- | --- | --- | --- |
| |  |  | | --- | --- | | Instance Methods | [hide private] | | |
|  | |  |  | | --- | --- | | \_\_init\_\_(self, pmid=None, title=None, abstract=None, journal=None, issn=None, date\_completed=None, pubyear=None, meshterms=None, authors=None)  Constructor, where parameters set instance variables. | source code | |
|  | |  |  | | --- | --- | | \_\_repr\_\_(self)  Evaluatable representation of the object | source code | |


|  |  |  |  |
| --- | --- | --- | --- |
| |  |  | | --- | --- | | Static Methods | [hide private] | | |
|  | |  |  | | --- | --- | | parse\_medline\_xml(stream)  Generate Article objects by parsing a Medline XML file | source code | |


|  |  |  |  |
| --- | --- | --- | --- |
| |  |  | | --- | --- | | Instance Variables | [hide private] | | |
|  | abstract  Abstract of the article (sring) |
|  | authors  Authors as a list of (initials, lastname) tuples of |
|  | date\_completed  (year,month,day) as integers |
|  | issn  Journal ISSN code (string) |
|  | journal  Medline abbreviated journal title (string) |
|  | meshterms  MeSH as a list of (descriptor, qual, qual, ...) tuples |
|  | pmid  PubMed ID of the article (int) |
|  | title  Title of the article (string) |
|  | year  Year of publication (int) |


|  |  |  |  |
| --- | --- | --- | --- |
| |  |  | | --- | --- | | Method Details | [hide private] | | |

|  |  |  |
| --- | --- | --- |
| |  |  | | --- | --- | | parse\_medline\_xml(stream)  *Static Method* | source code |  Generate Article objects by parsing a Medline XML file Parameters:  - **`stream`** - File-like object of MedlineCitation XML  Returns:  Iteratation over parsed Article objects |

  


| Trees | Indices | Help | | MScanner | | --- | |
| --- | --- | --- | --- | --- |

|  |  |
| --- | --- |
| Generated by Epydoc 3.0beta1 on Fri Nov 23 09:13:21 2007 | http://epydoc.sourceforge.net |
