# Supplementary material for: MScanner: a classifier for retrieving Medline citations
Source: BMC Bioinformatics. 2008 Feb 19;9:108. doi: 10.1186/1471-2105-9-108 (PMC2263023; doi:10.1186/1471-2105-9-108)
Supplement: Additional file 3 — Source code for MScanner. mscanner-20071123.zip is a ZIP archive containing the Python 2.5 source code for MScanner, licensed under the GNU General Public License. It also contains API documentation in HTML format. Updated versions will be made available at . [file 1471-2105-9-108-S3.zip › mscanner/help/api/mscanner.medline.Databases-pysrc.html]

xml version="1.0" encoding="ascii"?


mscanner.medline.Databases


| Trees | Indices | Help | | MScanner | | --- | |
| --- | --- | --- | --- | --- |

|  |  |  |  |
| --- | --- | --- | --- |
| Package mscanner :: Package medline :: Module Databases | |  | | --- | | [hide private] | | [frames] | no frames] | |

# Source Code for Module mscanner.medline.Databases

```
  1  """For consumers of the database, this opens L{FeatureDatabase},  
  2  L{FeatureMapping} and the article list""" 
  3   
  4  from __future__ import with_statement 
  5  import logging 
  6  import numpy as nx 
  7  from contextlib import closing 
  8  from mscanner.configuration import rc 
  9  from mscanner.medline.FeatureMapping import FeatureMapping 
 10  from mscanner.medline.FeatureDatabase import FeatureDatabase 
 11  from mscanner.medline import Shelf  
 12   
 13   
 14  __copyright__ = "2007 Graham Poulter" 
 15  __author__ = "Graham Poulter <http://graham.poulter.googlepages.com>" 
 16  __license__ = """This program is free software: you can redistribute it and/or 
 17  modify it under the terms of the GNU General Public License as published by the 
 18  Free Software Foundation, either version 3 of the License, or (at your option) 
 19  any later version. 
 20   
 21  This program is distributed in the hope that it will be useful, but WITHOUT ANY 
 22  WARRANTY; without even the implied warranty of MERCHANTABILITY or FITNESS FOR A 
 23  PARTICULAR PURPOSE. See the GNU General Public License for more details. 
 24   
 25  You should have received a copy of the GNU General Public License along with 
 26  this program. If not, see <http://www.gnu.org/licenses/>.""" 
 27   
 28   


29 -class Databases:


30      """The main interface to Medline used by the rest of the program. 
 31   
 32      The environment needs to be reloaded when databases are updated, 
 33      because L{featmap} and L{article_list} will have changed on disk. 
 34   
 35      @ivar artdb: Mmapping from PubMed ID to Article object 
 36   
 37      @ivar featdb: Mapping from PubMed ID to list of features 
 38   
 39      @ivar featmap: L{FeatureMapping} between feature names and feature IDs  
 40      (in particular, featmap[id] == feature string) 
 41       
 42      """ 
 43       


44 -    def __init__(self):


45          """Constructor for setting attributes to be used by the remaining 
 46          methods.""" 
 47          logging.info("Loading article databases") 
 48          self.featdb = FeatureDatabase(rc.featuredb, 'r') 
 49          self.featmap = FeatureMapping(rc.featuremap) 
 50          self.artdb = Shelf.open(rc.articledb, 'r')

 51   
 52   
 53      @property 


54 -    def article_list(self):


55          """Array with the PubMed IDs in the database. 
 56           
 57          @note: The rc.articlelist file is formatted as "PMID YYYYMMDD" one per line, 
 58          so we split and take the PubMed ID. 
 59           
 60          @note: At over 16 million members long, the property will 
 61          take a while to load the first time.""" 
 62          try: 
 63              return self._article_list 
 64          except AttributeError:  
 65              logging.info("Loading article list") 
 66              self._article_list = nx.array( 
 67                  [int(x.split()[0]) for x in rc.articlelist.lines()]) 
 68              return self._article_list

 69   
 70   


71 -    def close(self):


72          """Closes the feature and article databases""" 
 73          self.featdb.close() 
 74          self.artdb.close()

 75      __del__ = close

 76   
 77   


78 -def load_articles(db_path, pmids_path):


79      """Return Article objects given a file listing PubMed IDs, caching 
 80      the results in a pickle. 
 81   
 82      @note: The articles are cached in a pickle with ".pickle" added onto 
 83      the name of the PMID list file, so they are quick to load the second time. 
 84       
 85      @param db_path: Path to the database that maps PubMed IDs to Article objects. 
 86   
 87      @param pmids_path: Path to a text file listing one PubMed ID per line. 
 88   
 89      @return: List of Article objects in the order given in the text file. 
 90      """ 
 91      import cPickle 
 92      from mscanner.medline import Shelf 
 93      from contextlib import closing 
 94      from mscanner.core.iofuncs import read_pmids 
 95      from path import path # used in the line below 
 96      cache_path = path(pmids_path + ".pickle") 
 97      if cache_path.isfile(): 
 98          with open(cache_path, "rb") as f: 
 99              return cPickle.load(f) 
100      pmids = read_pmids(pmids_path) 
101      with closing(Shelf.open(db_path, "r")) as artdb: 
102          articles = [artdb[str(p)] for p in pmids] 
103      with open(cache_path, "wb") as f: 
104          cPickle.dump(articles, f, protocol=2) 
105      return articles

106
```

  


| Trees | Indices | Help | | MScanner | | --- | |
| --- | --- | --- | --- | --- |

|  |  |
| --- | --- |
| Generated by Epydoc 3.0beta1 on Fri Nov 23 09:13:25 2007 | http://epydoc.sourceforge.net |
