# Supplementary material for: MScanner: a classifier for retrieving Medline citations
Source: BMC Bioinformatics. 2008 Feb 19;9:108. doi: 10.1186/1471-2105-9-108 (PMC2263023; doi:10.1186/1471-2105-9-108)
Supplement: Additional file 3 — Source code for MScanner. mscanner-20071123.zip is a ZIP archive containing the Python 2.5 source code for MScanner, licensed under the GNU General Public License. It also contains API documentation in HTML format. Updated versions will be made available at . [file 1471-2105-9-108-S3.zip › mscanner/help/api/mscanner.medline.Databases.Databases-class.html]

xml version="1.0" encoding="ascii"?


mscanner.medline.Databases.Databases


| Trees | Indices | Help | | MScanner | | --- | |
| --- | --- | --- | --- | --- |

|  |  |  |  |
| --- | --- | --- | --- |
| Package mscanner :: Package medline :: Module Databases :: Class Databases | |  | | --- | | [hide private] | | [frames] | no frames] | |

# Class Databases

source code  
  

The main interface to Medline used by the rest of the program.

The environment needs to be reloaded when databases are updated,
because featmap and article\_list will have changed on disk.  
  


|  |  |  |  |
| --- | --- | --- | --- |
| |  |  | | --- | --- | | Instance Methods | [hide private] | | |
|  | |  |  | | --- | --- | | \_\_init\_\_(self)  Constructor for setting attributes to be used by the remaining methods. | source code | |
|  | |  |  | | --- | --- | | close(self)  Closes the feature and article databases | source code | |
|  | |  |  | | --- | --- | | \_\_del\_\_(self)  Closes the feature and article databases | source code | |


|  |  |  |  |
| --- | --- | --- | --- |
| |  |  | | --- | --- | | Instance Variables | [hide private] | | |
|  | artdb  Mmapping from PubMed ID to Article object |
|  | featdb  Mapping from PubMed ID to list of features |
|  | featmap  FeatureMapping between feature names and feature IDs (in particular, featmap[id] == feature string) |


|  |  |  |  |
| --- | --- | --- | --- |
| |  |  | | --- | --- | | Properties | [hide private] | | |
|  | article\_list  Array with the PubMed IDs in the database. |


|  |  |  |  |
| --- | --- | --- | --- |
| |  |  | | --- | --- | | Property Details | [hide private] | | |

|  |
| --- |
| article\_listArray with the PubMed IDs in the database. Get Method:  *unreachable*.article\_list(self) - Array with the PubMed IDs in the database.  **Notes:**  - The rc.articlelist file is formatted as "PMID   YYYYMMDD" one per line, so we split and take the PubMed   ID. - At over 16 million members long, the property will take a while   to load the first time. |

  


| Trees | Indices | Help | | MScanner | | --- | |
| --- | --- | --- | --- | --- |

|  |  |
| --- | --- |
| Generated by Epydoc 3.0beta1 on Fri Nov 23 09:13:21 2007 | http://epydoc.sourceforge.net |
