# Supplementary material for: MScanner: a classifier for retrieving Medline citations
Source: BMC Bioinformatics. 2008 Feb 19;9:108. doi: 10.1186/1471-2105-9-108 (PMC2263023; doi:10.1186/1471-2105-9-108)
Supplement: Additional file 3 — Source code for MScanner. mscanner-20071123.zip is a ZIP archive containing the Python 2.5 source code for MScanner, licensed under the GNU General Public License. It also contains API documentation in HTML format. Updated versions will be made available at . [file 1471-2105-9-108-S3.zip › mscanner/help/api/mscanner.medline.FeatureDatabase-pysrc.html]

xml version="1.0" encoding="ascii"?


mscanner.medline.FeatureDatabase


| Trees | Indices | Help | | MScanner | | --- | |
| --- | --- | --- | --- | --- |

|  |  |  |  |
| --- | --- | --- | --- |
| Package mscanner :: Package medline :: Module FeatureDatabase | |  | | --- | | [hide private] | | [frames] | no frames] | |

# Source Code for Module mscanner.medline.FeatureDatabase

```
  1  """Maps PubMed IDs to feature vectors""" 
  2   
  3  from bsddb import db 
  4  import numpy as nx 
  5  import logging 
  6  import struct 
  7   
  8   
  9  __copyright__ = "2007 Graham Poulter" 
 10  __author__ = "Graham Poulter <http://graham.poulter.googlepages.com>" 
 11  __license__ = """This program is free software: you can redistribute it and/or 
 12  modify it under the terms of the GNU General Public License as published by the 
 13  Free Software Foundation, either version 3 of the License, or (at your option) 
 14  any later version. 
 15   
 16  This program is distributed in the hope that it will be useful, but WITHOUT ANY 
 17  WARRANTY; without even the implied warranty of MERCHANTABILITY or FITNESS FOR A 
 18  PARTICULAR PURPOSE. See the GNU General Public License for more details. 
 19   
 20  You should have received a copy of the GNU General Public License along with 
 21  this program. If not, see <http://www.gnu.org/licenses/>.""" 
 22   
 23   


24 -class FeatureDatabase:


25      """Database for which PubMed ID is the key and array of Feature IDs are values 
 26      """ 
 27       


28 -    def __init__(self, filename=None, flags='c', mode=0660, dbenv=None, txn=None, dbname=None, ftype=nx.uint16):


29          """Initialise database 
 30   
 31          @param filename: Path to database file 
 32          @param flags: Opening flags (r,rw,w,c,n) 
 33          @param mode: Numeric file permissions 
 34          @param dbenv: Optional database environment 
 35          @param txn: Optional database transaction 
 36          @param dbname: Logical database name 
 37          @param ftype: Numpy numeric feature type 
 38          """ 
 39          self.ftype = ftype 
 40          if isinstance(flags, basestring): 
 41              if flags == 'r': 
 42                  flags = db.DB_RDONLY 
 43              elif flags == 'rw': 
 44                  flags = 0 
 45              elif flags == 'w': 
 46                  flags = db.DB_CREATE 
 47              elif flags == 'c': 
 48                  flags = db.DB_CREATE 
 49              elif flags == 'n': 
 50                  flags = db.DB_TRUNCATE | db.DB_CREATE 
 51              else: 
 52                  raise db.DBError("Flag %s is not in 'r', 'rw', 'w', 'c' or 'n'"  % str(flags)) 
 53          self.db = db.DB(dbenv) 
 54          self.db.open(filename, dbname, db.DB_HASH, flags, mode, txn=txn)

 55   
 56   


57 -    def close(self):


58          """Close the database.  Do not use this object after doing so""" 
 59          self.db.close()

 60      __del__ = close 
 61   
 62   


63 -    def getitem(self, key, txn=None):


64          """Return an ndarray object of values for a given key""" 
 65          buf = self.db.get(str(key), txn=txn) 
 66          if buf is None: 
 67              raise KeyError("Record %d not found in feature database" % key) 
 68          return nx.fromstring(buf, self.ftype)

 69   
 70   


71 -    def setitem(self, key, features, txn=None):


72          """Associate integer key with an ndarray object of values""" 
 73          if features.dtype != self.ftype: 
 74              raise ValueError("array type mismatch: " +  
 75                               str(features.dtype) + " for key " + str(key)) 
 76          try: 
 77              self.db.put(str(key), features.tostring(), txn=txn) 
 78          except ValueError, e: 
 79              logging.error("Failed to add to db " + str(key) + " : " +  str(features)) 
 80              raise

 81   
 82   


83 -    def delitem(self, key, txn=None):


84          """Delete a given key from the database""" 
 85          self.db.delete(str(key), txn=txn)

 86   
 87   
 88      # Bunch of dictionary-like methods 
 89   


90 -    def __getitem__(self, key):


91          return self.getitem(key)

 92   
 93   


94 -    def __setitem__(self, key, values):


95          self.setitem(key, values)

 96   
 97   


98 -    def __len__(self):


99          """Fast way to check number of items in database""" 
100          return self.db.stat()["ndata"]

101   
102   


103 -    def __contains__(self, key):


104          """Test for document ID membership.  Converts ID to a string first.""" 
105          return self.db.has_key(str(key))

106   
107   


108 -    def keys(self):


109          """Return list of PubMed IDs in the database""" 
110          return [ k for k in self ]

111   
112   


113 -    def __iter__(self):


114          """Iterate over PubMed IDs in the database""" 
115          cur = self.db.cursor() 
116          rec = cur.first(dlen=0, doff=0) 
117          while rec is not None: 
118              yield rec[0] 
119              rec = cur.next(dlen=0, doff=0) 
120          cur.close()

121   
122   


123 -    def iteritems(self):


124          """Iterate over (PMID, ndarray) pairs in the database""" 
125          cur = self.db.cursor() 
126          rec = cur.first() 
127          while rec is not None: 
128              yield rec[0], nx.fromstring(rec[1],self.ftype) 
129              rec = cur.next() 
130          cur.close()

131
```

  


| Trees | Indices | Help | | MScanner | | --- | |
| --- | --- | --- | --- | --- |

|  |  |
| --- | --- |
| Generated by Epydoc 3.0beta1 on Fri Nov 23 09:13:23 2007 | http://epydoc.sourceforge.net |
