# Supplementary material for: MScanner: a classifier for retrieving Medline citations
Source: BMC Bioinformatics. 2008 Feb 19;9:108. doi: 10.1186/1471-2105-9-108 (PMC2263023; doi:10.1186/1471-2105-9-108)
Supplement: Additional file 3 — Source code for MScanner. mscanner-20071123.zip is a ZIP archive containing the Python 2.5 source code for MScanner, licensed under the GNU General Public License. It also contains API documentation in HTML format. Updated versions will be made available at . [file 1471-2105-9-108-S3.zip › mscanner/help/api/mscanner.medline.FeatureDatabase.FeatureDatabase-class.html]

xml version="1.0" encoding="ascii"?


mscanner.medline.FeatureDatabase.FeatureDatabase


| Trees | Indices | Help | | MScanner | | --- | |
| --- | --- | --- | --- | --- |

|  |  |  |  |
| --- | --- | --- | --- |
| Package mscanner :: Package medline :: Module FeatureDatabase :: Class FeatureDatabase | |  | | --- | | [hide private] | | [frames] | no frames] | |

# Class FeatureDatabase

source code  
  
Database for which PubMed ID is the key and array of Feature IDs are
values  
  


|  |  |  |  |
| --- | --- | --- | --- |
| |  |  | | --- | --- | | Instance Methods | [hide private] | | |
|  | |  |  | | --- | --- | | \_\_init\_\_(self, filename=None, flags=`'``c``'`, mode=432, dbenv=None, txn=None, dbname=None, ftype=<type 'numpy.uint16'>)  Initialise database | source code | |
|  | |  |  | | --- | --- | | close(self)  Close the database. | source code | |
|  | |  |  | | --- | --- | | \_\_del\_\_(self)  Close the database. | source code | |
|  | |  |  | | --- | --- | | getitem(self, key, txn=None)  Return an ndarray object of values for a given key | source code | |
|  | |  |  | | --- | --- | | setitem(self, key, features, txn=None)  Associate integer key with an ndarray object of values | source code | |
|  | |  |  | | --- | --- | | delitem(self, key, txn=None)  Delete a given key from the database | source code | |
|  | |  |  | | --- | --- | | \_\_getitem\_\_(self, key) | source code | |
|  | |  |  | | --- | --- | | \_\_setitem\_\_(self, key, values) | source code | |
|  | |  |  | | --- | --- | | \_\_len\_\_(self)  Fast way to check number of items in database | source code | |
|  | |  |  | | --- | --- | | \_\_contains\_\_(self, key)  Test for document ID membership. | source code | |
|  | |  |  | | --- | --- | | keys(self)  Return list of PubMed IDs in the database | source code | |
|  | |  |  | | --- | --- | | \_\_iter\_\_(self)  Iterate over PubMed IDs in the database | source code | |
|  | |  |  | | --- | --- | | iteritems(self)  Iterate over (PMID, ndarray) pairs in the database | source code | |


|  |  |  |  |
| --- | --- | --- | --- |
| |  |  | | --- | --- | | Method Details | [hide private] | | |

|  |  |  |
| --- | --- | --- |
| |  |  | | --- | --- | | \_\_init\_\_(self, filename=None, flags=`'``c``'`, mode=432, dbenv=None, txn=None, dbname=None, ftype=<type 'numpy.uint16'>)  *(Constructor)* | source code |  Initialise database Parameters:  - **`filename`** - Path to database file - **`flags`** - Opening flags (r,rw,w,c,n) - **`mode`** - Numeric file permissions - **`dbenv`** - Optional database environment - **`txn`** - Optional database transaction - **`dbname`** - Logical database name - **`ftype`** - Numpy numeric feature type |

|  |  |  |
| --- | --- | --- |
| |  |  | | --- | --- | | close(self) | source code |  Close the database. Do not use this object after doing so |

|  |  |  |
| --- | --- | --- |
| |  |  | | --- | --- | | \_\_del\_\_(self)  *(Destructor)* | source code |  Close the database. Do not use this object after doing so |

|  |  |  |
| --- | --- | --- |
| |  |  | | --- | --- | | \_\_contains\_\_(self, key)  *(In operator)* | source code |  Test for document ID membership. Converts ID to a string first. |

  


| Trees | Indices | Help | | MScanner | | --- | |
| --- | --- | --- | --- | --- |

|  |  |
| --- | --- |
| Generated by Epydoc 3.0beta1 on Fri Nov 23 09:13:21 2007 | http://epydoc.sourceforge.net |
