# Supplementary material for: MScanner: a classifier for retrieving Medline citations
Source: BMC Bioinformatics. 2008 Feb 19;9:108. doi: 10.1186/1471-2105-9-108 (PMC2263023; doi:10.1186/1471-2105-9-108)
Supplement: Additional file 3 — Source code for MScanner. mscanner-20071123.zip is a ZIP archive containing the Python 2.5 source code for MScanner, licensed under the GNU General Public License. It also contains API documentation in HTML format. Updated versions will be made available at . [file 1471-2105-9-108-S3.zip › mscanner/help/api/mscanner.medline.FeatureDatabase.FeatureStream-class.html]

xml version="1.0" encoding="ascii"?


mscanner.medline.FeatureDatabase.FeatureStream


| Trees | Indices | Help | | MScanner | | --- | |
| --- | --- | --- | --- | --- |

|  |  |  |  |
| --- | --- | --- | --- |
| Package mscanner :: Package medline :: Module FeatureDatabase :: Class FeatureStream | |  | | --- | | [hide private] | | [frames] | no frames] | |

# Class FeatureStream

source code  
  
Binary file of records consisting of PubMed ID, record date and
feature vector.  
  


|  |  |  |  |
| --- | --- | --- | --- |
| |  |  | | --- | --- | | Instance Methods | [hide private] | | |
|  | |  |  | | --- | --- | | \_\_init\_\_(self, stream) | source code | |
|  | |  |  | | --- | --- | | close(self)  Close the underlying file | source code | |
|  | |  |  | | --- | --- | | write(self, pmid, date, features)  Add a record to the stream | source code | |
|  | |  |  | | --- | --- | | \_\_iter\_\_(self)  Iterate over tuples of (PubMed ID, YYYYMMDD, features). | source code | |


|  |  |  |  |
| --- | --- | --- | --- |
| |  |  | | --- | --- | | Instance Variables | [hide private] | | |
|  | stream  File or other object supporting read/write/close in binary mode. |


|  |  |  |  |
| --- | --- | --- | --- |
| |  |  | | --- | --- | | Method Details | [hide private] | | |

|  |  |  |
| --- | --- | --- |
| |  |  | | --- | --- | | write(self, pmid, date, features) | source code |  Add a record to the stream Parameters:  - **`pmid`** - PubMed ID (string or integer) - **`date`** - Either (year,month,day), or YYYMMDD integer date for the   record - **`features`** - Numpy array of uint16 feature IDs |

|  |  |  |
| --- | --- | --- |
| |  |  | | --- | --- | | \_\_iter\_\_(self) | source code |  Iterate over tuples of (PubMed ID, YYYYMMDD, features). The first two are integers, and the last is a numpy arrays of uint16. |

  


| Trees | Indices | Help | | MScanner | | --- | |
| --- | --- | --- | --- | --- |

|  |  |
| --- | --- |
| Generated by Epydoc 3.0beta1 on Mon Nov 12 18:55:18 2007 | http://epydoc.sourceforge.net |
