# Supplementary material for: MScanner: a classifier for retrieving Medline citations
Source: BMC Bioinformatics. 2008 Feb 19;9:108. doi: 10.1186/1471-2105-9-108 (PMC2263023; doi:10.1186/1471-2105-9-108)
Supplement: Additional file 3 — Source code for MScanner. mscanner-20071123.zip is a ZIP archive containing the Python 2.5 source code for MScanner, licensed under the GNU General Public License. It also contains API documentation in HTML format. Updated versions will be made available at . [file 1471-2105-9-108-S3.zip › mscanner/help/api/mscanner.medline.FeatureMapping-pysrc.html]

xml version="1.0" encoding="ascii"?


mscanner.medline.FeatureMapping


| Trees | Indices | Help | | MScanner | | --- | |
| --- | --- | --- | --- | --- |

|  |  |  |  |
| --- | --- | --- | --- |
| Package mscanner :: Package medline :: Module FeatureMapping | |  | | --- | | [hide private] | | [frames] | no frames] | |

# Source Code for Module mscanner.medline.FeatureMapping

```
  1  """Provides a mapping between features and integer IDs""" 
  2   
  3  from __future__ import with_statement 
  4  import codecs 
  5  import numpy as nx 
  6   
  7   
  8  __copyright__ = "2007 Graham Poulter" 
  9  __author__ = "Graham Poulter <http://graham.poulter.googlepages.com>" 
 10  __license__ = """This program is free software: you can redistribute it and/or 
 11  modify it under the terms of the GNU General Public License as published by the 
 12  Free Software Foundation, either version 3 of the License, or (at your option) 
 13  any later version. 
 14   
 15  This program is distributed in the hope that it will be useful, but WITHOUT ANY 
 16  WARRANTY; without even the implied warranty of MERCHANTABILITY or FITNESS FOR A 
 17  PARTICULAR PURPOSE. See the GNU General Public License for more details. 
 18   
 19  You should have received a copy of the GNU General Public License along with 
 20  this program. If not, see <http://www.gnu.org/licenses/>.""" 
 21   
 22   


23 -class FeatureMapping:


24      """Persistent mapping between string features and feature IDs 
 25   
 26      Feature types used with L{__getitem__}, L{get_type_mask} and 
 27      L{add_article} are "mesh", "qual", "issn". A feature string could have more 
 28      than one type. 
 29       
 30      This is really a table with columns (id,type,name,count), and keys of id 
 31      and (type,name). 
 32   
 33      @ivar featfile: Path to text file with list of terms 
 34   
 35      @ivar featfile_new: Temporary feature file used while writing 
 36       
 37      @ivar numdocs: Number of documents used in creating the mapping 
 38       
 39      @ivar features: List, such that features[id] == (name,type) 
 40       
 41      @ivar feature_ids: Mapping, such that feature_ids[type][name] == id 
 42       
 43      @ivar counts: List, such that counts[id] == number of occurrences.  For 
 44      score calculation this is the only column needed. 
 45      """ 
 46   


47 -    def __init__(self, featfile=None):


48          """Initialise the database, setting L{featfile}""" 
 49          self.featfile = featfile 
 50          if self.featfile is not None: 
 51              self.featfile_new = featfile+".new" 
 52          self.numdocs = 0 
 53          self.features = [] 
 54          self.feature_ids = {} 
 55          self.counts = [] 
 56          if featfile is not None and self.featfile.exists(): 
 57              self.load()

 58           


59 -    def load(self):


60          """Load feature mapping mapping from file as a tab-separated 
 61          table for tuples (feature, type, count) with ID being the 
 62          0-based index in the file. 
 63          """ 
 64          self.features = [] 
 65          self.feature_ids = {} 
 66          with codecs.open(self.featfile, "rb", "utf-8") as f: 
 67              self.numdocs = int(f.readline().strip()) 
 68              for fid, line in enumerate(f): 
 69                  feat, ftype, count = line.strip().split("\t") 
 70                  self.features.append((feat,ftype)) 
 71                  self.counts.append(int(count)) 
 72                  if ftype not in self.feature_ids: 
 73                      self.feature_ids[ftype] = {feat:fid} 
 74                  else: 
 75                      self.feature_ids[ftype][feat] = fid

 76   


77 -    def dump(self):


78          """Write the feature mapping to disk as a table of 
 79          (name, type, count) where line number is ID+1""" 
 80          if self.featfile is None: 
 81              return 
 82          with codecs.open(self.featfile_new, "wb", "utf-8") as f: 
 83              f.write("%s\n" % self.numdocs) 
 84              for (feat, ftype), count in zip(self.features, self.counts): 
 85                  f.write(feat+"\t"+ftype+"\t"+str(count)+"\n") 
 86          if self.featfile.isfile(): 
 87              self.featfile.remove() 
 88          self.featfile_new.rename(self.featfile)

 89   


90 -    def __getitem__(self, key):


91          """Given a feature ID, return (feature, feature type). Given (feature, 
 92          feature type), returns feature ID""" 
 93          if isinstance(key, int): 
 94              return self.features[key] 
 95          elif isinstance(key, tuple) and len(key) == 2: 
 96              return self.feature_ids[key[1]][key[0]] 
 97          else: 
 98              raise KeyError("Invalid key: %s" % str(key))

 99   


100 -    def __len__(self):


101          """Return number of distinct features""" 
102          return len(self.features)

103   


104 -    def get_type_mask(self, exclude_types):


105          """Get a mask for excluded features 
106   
107          @param exclude_types: Types of features to exclude 
108   
109          @return: Boolean array for excluded features (but returns None if 
110          exclude_types is None)  
111          """ 
112          if not exclude_types: 
113              return None 
114          exclude_feats = nx.zeros(len(self.features), nx.bool) 
115          for ftype in exclude_types: 
116              for fid in self.feature_ids[ftype].itervalues(): 
117                  exclude_feats[fid] = True 
118          return exclude_feats

119   


120 -    def add_article(self, **kwargs):


121          """Add an article, given lists of features of different types. 
122           
123          @note: Dynamically creates new features IDs and feature types as necessary. 
124           
125          @param kwargs: Mapping from feature types to lists of features for that 
126          type. e.g. C{mesh=["Term A","Term B"]} 
127           
128          @return: Numpy array of uint16 feature IDs 
129          """ 
130          result = [] 
131          self.numdocs += 1 
132          for ftype, fstrings in kwargs.iteritems(): 
133              if ftype not in self.feature_ids: 
134                  self.feature_ids[ftype] = {} 
135              fdict = self.feature_ids[ftype] 
136              for feat in fstrings: 
137                  if feat not in fdict: 
138                      featid = len(self.features) 
139                      self.features.append((feat,ftype)) 
140                      self.counts.append(1) 
141                      fdict[feat] = featid 
142                  else: 
143                      self.counts[fdict[feat]] += 1 
144                  result.append(fdict[feat]) 
145          return nx.array(result, nx.uint16)

146
```

  


| Trees | Indices | Help | | MScanner | | --- | |
| --- | --- | --- | --- | --- |

|  |  |
| --- | --- |
| Generated by Epydoc 3.0beta1 on Fri Nov 23 09:13:24 2007 | http://epydoc.sourceforge.net |
