# Supplementary material for: MScanner: a classifier for retrieving Medline citations
Source: BMC Bioinformatics. 2008 Feb 19;9:108. doi: 10.1186/1471-2105-9-108 (PMC2263023; doi:10.1186/1471-2105-9-108)
Supplement: Additional file 3 — Source code for MScanner. mscanner-20071123.zip is a ZIP archive containing the Python 2.5 source code for MScanner, licensed under the GNU General Public License. It also contains API documentation in HTML format. Updated versions will be made available at . [file 1471-2105-9-108-S3.zip › mscanner/help/api/mscanner.medline.FeatureMapping.FeatureMapping-class.html]

xml version="1.0" encoding="ascii"?


mscanner.medline.FeatureMapping.FeatureMapping


| Trees | Indices | Help | | MScanner | | --- | |
| --- | --- | --- | --- | --- |

|  |  |  |  |
| --- | --- | --- | --- |
| Package mscanner :: Package medline :: Module FeatureMapping :: Class FeatureMapping | |  | | --- | | [hide private] | | [frames] | no frames] | |

# Class FeatureMapping

source code  
  

Persistent mapping between string features and feature IDs

Feature types used with \_\_getitem\_\_, get\_type\_mask and add\_article are "mesh", "qual",
"issn". A feature string could have more than one type.

This is really a table with columns (id,type,name,count), and keys of
id and (type,name).  
  


|  |  |  |  |
| --- | --- | --- | --- |
| |  |  | | --- | --- | | Instance Methods | [hide private] | | |
|  | |  |  | | --- | --- | | \_\_init\_\_(self, featfile=None)  Initialise the database, setting featfile | source code | |
|  | |  |  | | --- | --- | | load(self)  Load feature mapping mapping from file as a tab-separated table for tuples (feature, type, count) with ID being the 0-based index in the file. | source code | |
|  | |  |  | | --- | --- | | dump(self)  Write the feature mapping to disk as a table of (name, type, count) where line number is ID+1 | source code | |
|  | |  |  | | --- | --- | | \_\_getitem\_\_(self, key)  Given a feature ID, return (feature, feature type). | source code | |
|  | |  |  | | --- | --- | | \_\_len\_\_(self)  Return number of distinct features | source code | |
|  | |  |  | | --- | --- | | get\_type\_mask(self, exclude\_types)  Get a mask for excluded features | source code | |
|  | |  |  | | --- | --- | | add\_article(self, \*\*kwargs)  Add an article, given lists of features of different types. | source code | |


|  |  |  |  |
| --- | --- | --- | --- |
| |  |  | | --- | --- | | Instance Variables | [hide private] | | |
|  | counts  List, such that counts[id] == number of occurrences. |
|  | featfile  Path to text file with list of terms |
|  | featfile\_new  Temporary feature file used while writing |
|  | feature\_ids  Mapping, such that feature\_ids[type][name] == id |
|  | features  List, such that features[id] == (name,type) |
|  | numdocs  Number of documents used in creating the mapping |


|  |  |  |  |
| --- | --- | --- | --- |
| |  |  | | --- | --- | | Method Details | [hide private] | | |

|  |  |  |
| --- | --- | --- |
| |  |  | | --- | --- | | \_\_getitem\_\_(self, key)  *(Indexing operator)* | source code |  Given a feature ID, return (feature, feature type). Given (feature, feature type), returns feature ID |

|  |  |  |
| --- | --- | --- |
| |  |  | | --- | --- | | get\_type\_mask(self, exclude\_types) | source code |  Get a mask for excluded features Parameters:  - **`exclude_types`** - Types of features to exclude  Returns:  Boolean array for excluded features (but returns None if exclude\_types is None) |

|  |  |  |
| --- | --- | --- |
| |  |  | | --- | --- | | add\_article(self, \*\*kwargs) | source code |  Add an article, given lists of features of different types. Parameters:  - **`kwargs`** - Mapping from feature types to lists of features for that type.   e.g. `mesh=["Term A","Term   B"]`  Returns:  Numpy array of uint16 feature IDs  **Note:** Dynamically creates new features IDs and feature types as necessary. |

  


|  |  |  |  |
| --- | --- | --- | --- |
| |  |  | | --- | --- | | Instance Variable Details | [hide private] | | |

|  |
| --- |
| countsList, such that counts[id] == number of occurrences. For score calculation this is the only column needed. |

  


| Trees | Indices | Help | | MScanner | | --- | |
| --- | --- | --- | --- | --- |

|  |  |
| --- | --- |
| Generated by Epydoc 3.0beta1 on Fri Nov 23 09:13:22 2007 | http://epydoc.sourceforge.net |
