# Supplementary material for: MScanner: a classifier for retrieving Medline citations
Source: BMC Bioinformatics. 2008 Feb 19;9:108. doi: 10.1186/1471-2105-9-108 (PMC2263023; doi:10.1186/1471-2105-9-108)
Supplement: Additional file 3 — Source code for MScanner. mscanner-20071123.zip is a ZIP archive containing the Python 2.5 source code for MScanner, licensed under the GNU General Public License. It also contains API documentation in HTML format. Updated versions will be made available at . [file 1471-2105-9-108-S3.zip › mscanner/help/api/mscanner.medline.FeatureStream-module.html]

xml version="1.0" encoding="ascii"?


mscanner.medline.FeatureStream


| Trees | Indices | Help | | MScanner | | --- | |
| --- | --- | --- | --- | --- |

|  |  |  |  |
| --- | --- | --- | --- |
| Package mscanner :: Package medline :: Module FeatureStream | |  | | --- | | [hide private] | | [frames] | no frames] | |

# Module FeatureStream

source code  
  
A class for rapid iteration over the records in Medline.  
  


---

**Author:**
Graham Poulter <http://graham.poulter.googlepages.com>

**Copyright:**
2007 Graham Poulter

**License:**
This program is free software: you can redistribute it and/or
modify it under the terms of the GNU General Public License as
published by the
Free Software Foundation, either version 3 of the License, or (at
your option)
any later version.
This program is distributed in the hope that it will be useful, but
WITHOUT ANY
WARRANTY; without even the implied warranty of MERCHANTABILITY or
FITNESS FOR A
PARTICULAR PURPOSE. See the GNU General Public License for more
details.
You should have received a copy of the GNU General Public License
along with
this program. If not, see <http://www.gnu.org/licenses/>.


|  |  |  |  |
| --- | --- | --- | --- |
| |  |  | | --- | --- | | Classes | [hide private] | | |
|  | FeatureStream  Class for reading/writing a binary stream of Medline records, consisting of PubMed ID, record completion date and a vector of Feature IDs for features present in the record. |


|  |  |  |  |
| --- | --- | --- | --- |
| |  |  | | --- | --- | | Functions | [hide private] | | |
|  | |  |  | | --- | --- | | Date2Integer(date)  Given (year,month,day), return the integer representation | source code | |
|  | |  |  | | --- | --- | | Integer2Date(intdate)  Given a YYYYMMDD integer, return (year,month,day) | source code | |

| Trees | Indices | Help | | MScanner | | --- | |
| --- | --- | --- | --- | --- |

|  |  |
| --- | --- |
| Generated by Epydoc 3.0beta1 on Fri Nov 23 09:13:20 2007 | http://epydoc.sourceforge.net |
