# Supplementary material for: MScanner: a classifier for retrieving Medline citations
Source: BMC Bioinformatics. 2008 Feb 19;9:108. doi: 10.1186/1471-2105-9-108 (PMC2263023; doi:10.1186/1471-2105-9-108)
Supplement: Additional file 3 — Source code for MScanner. mscanner-20071123.zip is a ZIP archive containing the Python 2.5 source code for MScanner, licensed under the GNU General Public License. It also contains API documentation in HTML format. Updated versions will be made available at . [file 1471-2105-9-108-S3.zip › mscanner/help/api/mscanner.medline.FeatureStream-pysrc.html]

xml version="1.0" encoding="ascii"?


mscanner.medline.FeatureStream


| Trees | Indices | Help | | MScanner | | --- | |
| --- | --- | --- | --- | --- |

|  |  |  |  |
| --- | --- | --- | --- |
| Package mscanner :: Package medline :: Module FeatureStream | |  | | --- | | [hide private] | | [frames] | no frames] | |

# Source Code for Module mscanner.medline.FeatureStream

```
 1  """A class for rapid iteration over the records in Medline.""" 
 2   
 3  import numpy as nx 
 4  import struct 
 5   
 6   
 7  __copyright__ = "2007 Graham Poulter" 
 8  __author__ = "Graham Poulter <http://graham.poulter.googlepages.com>" 
 9  __license__ = """This program is free software: you can redistribute it and/or 
10  modify it under the terms of the GNU General Public License as published by the 
11  Free Software Foundation, either version 3 of the License, or (at your option) 
12  any later version. 
13   
14  This program is distributed in the hope that it will be useful, but WITHOUT ANY 
15  WARRANTY; without even the implied warranty of MERCHANTABILITY or FITNESS FOR A 
16  PARTICULAR PURPOSE. See the GNU General Public License for more details. 
17   
18  You should have received a copy of the GNU General Public License along with 
19  this program. If not, see <http://www.gnu.org/licenses/>.""" 
20   
21   


22 -class FeatureStream:


23      """Class for reading/writing a binary stream of Medline records, consisting 
24      of PubMed ID, record completion date and a vector of Feature IDs for 
25      features present in the record.  This stream is  
26       
27      @ivar stream: File-like object (read/write/close, binary strings).""" 
28   


29 -    def __init__(self, stream):


30          self.stream = stream

31   
32   


33 -    def close(self):


34          """Close the underlying stream.""" 
35          self.stream.close()

36   
37   


38 -    def write(self, pmid, date, features):


39          """Add a record to the stream 
40           
41          @param pmid: PubMed ID (string or integer). 
42           
43          @param date: Either (year,month,day), or YYYMMDD integer date for the 
44          record. 
45           
46          @param features: Numpy array of uint16 feature IDs.""" 
47          if features.dtype != nx.uint16: 
48              raise ValueError("Array dtype must be uint16, not %s" % str(features.dtype)) 
49          if isinstance(date, tuple): 
50              date = Date2Integer(date) 
51          self.stream.write(struct.pack("IIH", int(pmid), date, len(features))) 
52          features.tofile(self.stream)

53   
54   


55 -    def __iter__(self):


56          """Iterate over tuples of (PubMed ID, YYYYMMDD, features). The first 
57          two are integers, and the last is a numpy arrays of uint16. 
58           
59          @note: Rather use the C programs for ScoreCalculator and FeatureCounter 
60          in the L{mscanner.fastscores} package.""" 
61          header_len = 4+4+2 # IIH header 
62          head = self.stream.read(header_len) 
63          while len(head) == header_len: 
64              pmid, date, alen = struct.unpack("IIH", head) 
65              yield (pmid, date, nx.fromfile(self.stream, nx.uint16, alen)) 
66              head = self.stream.read(header_len)

67   
68   


69 -def Date2Integer(date):


70      """Given (year,month,day), return the integer representation""" 
71      return date[0]*10000 + date[1]*100 + date[2]

72   
73   


74 -def Integer2Date(intdate):


75      """Given a YYYYMMDD integer, return (year,month,day)""" 
76      return intdate//10000, (intdate%10000)//100, intdate%100

77
```

  


| Trees | Indices | Help | | MScanner | | --- | |
| --- | --- | --- | --- | --- |

|  |  |
| --- | --- |
| Generated by Epydoc 3.0beta1 on Fri Nov 23 09:13:23 2007 | http://epydoc.sourceforge.net |
