# Supplementary material for: MScanner: a classifier for retrieving Medline citations
Source: BMC Bioinformatics. 2008 Feb 19;9:108. doi: 10.1186/1471-2105-9-108 (PMC2263023; doi:10.1186/1471-2105-9-108)
Supplement: Additional file 3 — Source code for MScanner. mscanner-20071123.zip is a ZIP archive containing the Python 2.5 source code for MScanner, licensed under the GNU General Public License. It also contains API documentation in HTML format. Updated versions will be made available at . [file 1471-2105-9-108-S3.zip › mscanner/help/api/mscanner.medline.FeatureStream.FeatureStream-class.html]

xml version="1.0" encoding="ascii"?


mscanner.medline.FeatureStream.FeatureStream


| Trees | Indices | Help | | MScanner | | --- | |
| --- | --- | --- | --- | --- |

|  |  |  |  |
| --- | --- | --- | --- |
| Package mscanner :: Package medline :: Module FeatureStream :: Class FeatureStream | |  | | --- | | [hide private] | | [frames] | no frames] | |

# Class FeatureStream

source code  
  
Class for reading/writing a binary stream of Medline records,
consisting of PubMed ID, record completion date and a vector of Feature
IDs for features present in the record. This stream is  
  


|  |  |  |  |
| --- | --- | --- | --- |
| |  |  | | --- | --- | | Instance Methods | [hide private] | | |
|  | |  |  | | --- | --- | | \_\_init\_\_(self, stream) | source code | |
|  | |  |  | | --- | --- | | close(self)  Close the underlying stream. | source code | |
|  | |  |  | | --- | --- | | write(self, pmid, date, features)  Add a record to the stream | source code | |
|  | |  |  | | --- | --- | | \_\_iter\_\_(self)  Iterate over tuples of (PubMed ID, YYYYMMDD, features). | source code | |


|  |  |  |  |
| --- | --- | --- | --- |
| |  |  | | --- | --- | | Instance Variables | [hide private] | | |
|  | stream  File-like object (read/write/close, binary strings). |


|  |  |  |  |
| --- | --- | --- | --- |
| |  |  | | --- | --- | | Method Details | [hide private] | | |

|  |  |  |
| --- | --- | --- |
| |  |  | | --- | --- | | write(self, pmid, date, features) | source code |  Add a record to the stream Parameters:  - **`pmid`** - PubMed ID (string or integer). - **`date`** - Either (year,month,day), or YYYMMDD integer date for the   record. - **`features`** - Numpy array of uint16 feature IDs. |

|  |  |  |
| --- | --- | --- |
| |  |  | | --- | --- | | \_\_iter\_\_(self) | source code |  Iterate over tuples of (PubMed ID, YYYYMMDD, features). The first two are integers, and the last is a numpy arrays of uint16.   **Note:** Rather use the C programs for ScoreCalculator and FeatureCounter in the mscanner.fastscores package. |

  


| Trees | Indices | Help | | MScanner | | --- | |
| --- | --- | --- | --- | --- |

|  |  |
| --- | --- |
| Generated by Epydoc 3.0beta1 on Fri Nov 23 09:13:22 2007 | http://epydoc.sourceforge.net |
