# Supplementary material for: MScanner: a classifier for retrieving Medline citations
Source: BMC Bioinformatics. 2008 Feb 19;9:108. doi: 10.1186/1471-2105-9-108 (PMC2263023; doi:10.1186/1471-2105-9-108)
Supplement: Additional file 3 — Source code for MScanner. mscanner-20071123.zip is a ZIP archive containing the Python 2.5 source code for MScanner, licensed under the GNU General Public License. It also contains API documentation in HTML format. Updated versions will be made available at . [file 1471-2105-9-108-S3.zip › mscanner/help/api/mscanner.medline.FileTracker-pysrc.html]

xml version="1.0" encoding="ascii"?


mscanner.medline.FileTracker


| Trees | Indices | Help | | MScanner | | --- | |
| --- | --- | --- | --- | --- |

|  |  |  |  |
| --- | --- | --- | --- |
| Package mscanner :: Package medline :: Module FileTracker | |  | | --- | | [hide private] | | [frames] | no frames] | |

# Source Code for Module mscanner.medline.FileTracker

```
 1  """A simple persistant set of file names""" 
 2   
 3  from __future__ import with_statement 
 4  from path import path 
 5   
 6   
 7  __copyright__ = "2007 Graham Poulter" 
 8  __author__ = "Graham Poulter <http://graham.poulter.googlepages.com>" 
 9  __license__ = """This program is free software: you can redistribute it and/or 
10  modify it under the terms of the GNU General Public License as published by the 
11  Free Software Foundation, either version 3 of the License, or (at your option) 
12  any later version. 
13   
14  This program is distributed in the hope that it will be useful, but WITHOUT ANY 
15  WARRANTY; without even the implied warranty of MERCHANTABILITY or FITNESS FOR A 
16  PARTICULAR PURPOSE. See the GNU General Public License for more details. 
17   
18  You should have received a copy of the GNU General Public License along with 
19  this program. If not, see <http://www.gnu.org/licenses/>.""" 
20   
21   


22 -class FileTracker(set):


23      """A persistent set for tracking of processed files. 
24       
25      @ivar trackfile: Path for saving/loading the list of precessed files 
26      """ 
27   


28 -    def __init__(self, trackfile=None):


29          """Constructor - sets L{trackfile}""" 
30          self.trackfile = trackfile 
31          if isinstance(trackfile, path) and trackfile.isfile(): 
32              self.update(trackfile.lines(retain=False))

33   


34 -    def dump(self):


35          """Write the list of tracked files, one per line""" 
36          if self.trackfile is None: 
37              return 
38          tfnew = self.trackfile + ".new" 
39          tfnew.write_lines(sorted(self)) 
40          if self.trackfile.isfile(): 
41              self.trackfile.remove() 
42          tfnew.rename(self.trackfile)

43   


44 -    def add(self, fname):


45          """Add fname.basename() to the set""" 
46          set.add(self, fname.basename())

47   


48 -    def toprocess(self, paths):


49          """Filter for files that have not been processed yet 
50           
51          @param paths: List of paths to consider 
52           
53          @return: Those members of L{paths} whose base names are not in the set 
54          """ 
55          return sorted(f for f in paths if f.basename() not in self)

56
```

  


| Trees | Indices | Help | | MScanner | | --- | |
| --- | --- | --- | --- | --- |

|  |  |
| --- | --- |
| Generated by Epydoc 3.0beta1 on Fri Nov 23 09:13:23 2007 | http://epydoc.sourceforge.net |
