# Supplementary material for: MScanner: a classifier for retrieving Medline citations
Source: BMC Bioinformatics. 2008 Feb 19;9:108. doi: 10.1186/1471-2105-9-108 (PMC2263023; doi:10.1186/1471-2105-9-108)
Supplement: Additional file 3 — Source code for MScanner. mscanner-20071123.zip is a ZIP archive containing the Python 2.5 source code for MScanner, licensed under the GNU General Public License. It also contains API documentation in HTML format. Updated versions will be made available at . [file 1471-2105-9-108-S3.zip › mscanner/help/api/mscanner.medline.FileTracker.FileTracker-class.html]

xml version="1.0" encoding="ascii"?


mscanner.medline.FileTracker.FileTracker


| Trees | Indices | Help | | MScanner | | --- | |
| --- | --- | --- | --- | --- |

|  |  |  |  |
| --- | --- | --- | --- |
| Package mscanner :: Package medline :: Module FileTracker :: Class FileTracker | |  | | --- | | [hide private] | | [frames] | no frames] | |

# Class FileTracker

source code  
  

```
object --+    
         |    
       set --+
             |
            FileTracker
```

---

A persistent set for tracking of processed files.  
  


|  |  |  |  |
| --- | --- | --- | --- |
| |  |  | | --- | --- | | Instance Methods | [hide private] | | |
|  | |  |  | | --- | --- | | \_\_init\_\_(self, trackfile=None)  Constructor - sets trackfile | source code | |
|  | |  |  | | --- | --- | | dump(self)  Write the list of tracked files, one per line | source code | |
|  | |  |  | | --- | --- | | add(self, fname)  Add fname.basename() to the set | source code | |
|  | |  |  | | --- | --- | | toprocess(self, paths)  Filter for files that have not been processed yet | source code | |
| **Inherited from `set`**: `__and__`, `__cmp__`, `__contains__`, `__eq__`, `__ge__`, `__getattribute__`, `__gt__`, `__hash__`, `__iand__`, `__ior__`, `__isub__`, `__iter__`, `__ixor__`, `__le__`, `__len__`, `__lt__`, `__ne__`, `__new__`, `__or__`, `__rand__`, `__reduce__`, `__repr__`, `__ror__`, `__rsub__`, `__rxor__`, `__sub__`, `__xor__`, `clear`, `copy`, `difference`, `difference_update`, `discard`, `intersection`, `intersection_update`, `issubset`, `issuperset`, `pop`, `remove`, `symmetric_difference`, `symmetric_difference_update`, `union`, `update`  **Inherited from `object`**: `__delattr__`, `__reduce_ex__`, `__setattr__`, `__str__` | |


|  |  |  |  |
| --- | --- | --- | --- |
| |  |  | | --- | --- | | Instance Variables | [hide private] | | |
|  | trackfile  Path for saving/loading the list of precessed files |


|  |  |  |  |
| --- | --- | --- | --- |
| |  |  | | --- | --- | | Properties | [hide private] | | |
| **Inherited from `object`**: `__class__` | |


|  |  |  |  |
| --- | --- | --- | --- |
| |  |  | | --- | --- | | Method Details | [hide private] | | |

|  |  |  |
| --- | --- | --- |
| |  |  | | --- | --- | | \_\_init\_\_(self, trackfile=None)  *(Constructor)* | source code |  Constructor - sets trackfile Overrides: set.\_\_init\_\_ |

|  |  |  |
| --- | --- | --- |
| |  |  | | --- | --- | | add(self, fname) | source code |  Add fname.basename() to the set Overrides: set.add |

|  |  |  |
| --- | --- | --- |
| |  |  | | --- | --- | | toprocess(self, paths) | source code |  Filter for files that have not been processed yet Parameters:  - **`paths`** - List of paths to consider  Returns:  Those members of `paths` whose base names are not in the set |

  


| Trees | Indices | Help | | MScanner | | --- | |
| --- | --- | --- | --- | --- |

|  |  |
| --- | --- |
| Generated by Epydoc 3.0beta1 on Fri Nov 23 09:13:22 2007 | http://epydoc.sourceforge.net |
