# Supplementary material for: MScanner: a classifier for retrieving Medline citations
Source: BMC Bioinformatics. 2008 Feb 19;9:108. doi: 10.1186/1471-2105-9-108 (PMC2263023; doi:10.1186/1471-2105-9-108)
Supplement: Additional file 3 — Source code for MScanner. mscanner-20071123.zip is a ZIP archive containing the Python 2.5 source code for MScanner, licensed under the GNU General Public License. It also contains API documentation in HTML format. Updated versions will be made available at . [file 1471-2105-9-108-S3.zip › mscanner/help/api/mscanner.medline.MedlineCache-pysrc.html]

xml version="1.0" encoding="ascii"?


mscanner.medline.MedlineCache


| Trees | Indices | Help | | MScanner | | --- | |
| --- | --- | --- | --- | --- |

|  |  |  |  |
| --- | --- | --- | --- |
| Package mscanner :: Package medline :: Module MedlineCache | |  | | --- | | [hide private] | | [frames] | no frames] | |

# Source Code for Module mscanner.medline.MedlineCache

```
  1  """For updating the databases of articles and features""" 
  2   
  3  from __future__ import with_statement 
  4  from bsddb import db 
  5  import gzip 
  6  import logging 
  7  from path import path 
  8   
  9  from mscanner.medline import Shelf 
 10  from mscanner.medline.Article import Article 
 11  from mscanner.medline.FeatureDatabase import FeatureDatabase 
 12  from mscanner.medline.FeatureStream import FeatureStream, Date2Integer 
 13  from mscanner.medline.FileTracker import FileTracker 
 14  from mscanner.medline.FeatureMapping import FeatureMapping 
 15   
 16   
 17  __copyright__ = "2007 Graham Poulter" 
 18  __author__ = "Graham Poulter <http://graham.poulter.googlepages.com>" 
 19  __license__ = """This program is free software: you can redistribute it and/or 
 20  modify it under the terms of the GNU General Public License as published by the 
 21  Free Software Foundation, either version 3 of the License, or (at your option) 
 22  any later version. 
 23   
 24  This program is distributed in the hope that it will be useful, but WITHOUT ANY 
 25  WARRANTY; without even the implied warranty of MERCHANTABILITY or FITNESS FOR A 
 26  PARTICULAR PURPOSE. See the GNU General Public License for more details. 
 27   
 28  You should have received a copy of the GNU General Public License along with 
 29  this program. If not, see <http://www.gnu.org/licenses/>.""" 
 30   
 31   


32 -class MedlineCache:

 33      """Class for updating the Article DB, FeatureMapping, FeatureDatabase, 
 34      FeatureStream, PMID list, and FileTracker. 
 35       
 36      @ivar featmap: A FeatureMapping object for mapping string features to IDs 
 37      @ivar db_env_home: Path to DB home directory  
 38      @ivar article_db: Path to article database 
 39      @ivar feature_db: Path to feature database 
 40      @ivar feature_stream: Path to feature stream file 
 41      @ivar article_list: Path to list of article PMIDs 
 42      @ivar narticles_path: Path to file containing the total number of PMIDs 
 43      @ivar processed_path: Path to list of processed files 
 44      @ivar use_transactions: If false, disable transaction engine 
 45      """ 
 46   


47 -    def __init__( 
 48          self, 
 49          featmap, 
 50          db_env_home, 
 51          article_db, 
 52          feature_db, 
 53          feature_stream, 
 54          article_list, 
 55          processed_path, 
 56          narticles_path, 
 57          use_transactions=True):


58          """Constructor parameters set corresponding instance variables.""" 
 59          self.db_env_home = db_env_home 
 60          self.featmap = featmap 
 61          self.article_db = article_db 
 62          self.feature_db = feature_db 
 63          self.feature_stream = feature_stream 
 64          self.article_list = article_list 
 65          self.processed_path = processed_path 
 66          self.narticles_path = narticles_path 
 67          self.use_transactions = use_transactions 
 68          self.recover = False

 69   
 70   


71 -    def create_dbenv(self):


72          """Create a Berkeley DB environment for transactions 
 73           
 74          @return: DBEnv instance""" 
 75          if not self.db_env_home.isdir(): 
 76              self.db_env_home.mkdir() 
 77          dbenv = db.DBEnv() 
 78          dbenv.set_lg_max(128*1024*1024) # 128Mb log files 
 79          dbenv.set_tx_max(1) # 1 transaction at a time 
 80          dbenv.set_cachesize(0, 8*1024*1024) # 8Mb shared cache 
 81          flags = db.DB_INIT_MPOOL|db.DB_CREATE 
 82          if self.use_transactions: 
 83              flags |= db.DB_INIT_TXN 
 84          if self.recover: 
 85              flags |= db.DB_RECOVER # might use db.DB_RECOVER_FATAL 
 86          dbenv.open(self.db_env_home, flags) 
 87          return dbenv

 88   
 89   


90 -    def _article_features(self, article):


91          """Given an article object, return its feature vector, 
 92          using L{featmap} to create new features as necessary""" 
 93          # Get MeSH headings, qualifiers and ISSN from article 
 94          headings = list() 
 95          quals = list() 
 96          for term in article.meshterms: 
 97              headings.append(term[0]) 
 98              if(len(term)>1): 
 99                  for q in term[1:]: 
100                      if q not in quals: 
101                          quals.append(q) 
102          issns = [article.issn] if article.issn is not None else [] 
103          # Get the feature vector while possibly them to the feature mapping 
104          return self.featmap.add_article(mesh=headings, qual=quals, issn=issns)

105   
106   


107 -    def add_articles(self, articles, dbenv):


108          """Store Articles and feature lists in the databases 
109           
110          Databases are opened and closed inside each call, so that the user can 
111          Ctrl-C during the timed delay between files without corrupting the 
112          database. Using transactions has too much overhead in time, and space 
113          used by the log files. 
114           
115          @param articles: Iterator over Article objects 
116           
117          @param dbenv: Database environment to use 
118          """ 
119          logging.info("Starting transaction to add articles") 
120          txn = dbenv.txn_begin() if self.use_transactions else None 
121          try: 
122              artdb = Shelf.open(self.article_db, dbenv=dbenv, txn=txn) 
123              meshfeatdb = FeatureDatabase(self.feature_db, dbenv=dbenv, txn=txn) 
124              featstream = FeatureStream(open(self.feature_stream,"ab")) 
125              if not self.narticles_path.isfile(): 
126                  narticles = len(meshfeatdb) 
127              else: 
128                  narticles = int(self.narticles_path.text()) 
129              pmidlist = [] # List of lines to add to articles file 
130              for art in articles: 
131                  # Refuse to add duplicates 
132                  if art.pmid in meshfeatdb:  
133                      continue 
134                  # Store record in article database 
135                  artdb[str(art.pmid)] = art 
136                  # Add PubMed ID to the list of Medline 
137                  pmidlist.append("%d %d" % ( 
138                      art.pmid, Date2Integer(art.date_completed))) 
139                  # Calculate the feature vector 
140                  featids = self._article_features(art) 
141                  # Associate PubMed ID with the feature vector 
142                  meshfeatdb.setitem(art.pmid, featids, txn) 
143                  # Also add (PMID, date, features) to a fast-iteration stream 
144                  featstream.write(art.pmid, art.date_completed, featids) 
145              artdb.close() 
146              meshfeatdb.close() 
147              featstream.close() 
148              # Update the list of PubMed IDs in Medline 
149              self.article_list.write_lines(pmidlist, append=True) 
150              # Update the number of PubMed IDs in Medline 
151              self.narticles_path.write_text(str(narticles+len(pmidlist))+"\n") 
152              self.featmap.dump() 
153              if txn is not None: 
154                  txn.commit() 
155          except Exception, e: 
156              if txn is not None: 
157                  logging.exception("Aborting Transaction: Error %s", e) 
158                  txn.abort() 
159              raise 
160          else: 
161              if txn is not None: 
162                  logging.info("Committed transaction") 
163              return len(pmidlist)

164   
165   


166 -    def add_directory(self, medlinedir, save_delay=5):


167          """Adds articles from XML files to MScanner's databases 
168           
169          @param medlinedir: Path to a directory containing .xml.gz 
170          files 
171           
172          @param save_delay: Pause this many seconds between calls to 
173          L{add_articles}""" 
174          import time 
175          filenames = medlinedir.files("*.xml") + medlinedir.files("*.xml.gz") 
176          tracker = FileTracker(self.processed_path) 
177          toprocess = tracker.toprocess(filenames) 
178          dbenv = self.create_dbenv() 
179          for idx, filename in enumerate(toprocess): 
180              logging.info("Adding to cache: file %d out of %d (%s)",  
181                       idx+1, len(toprocess), filename.name) 
182              for t in xrange(save_delay): 
183                  logging.debug("Saving in %d seconds...", save_delay-t) 
184                  time.sleep(1) 
185              logging.debug("Parsing XML file %s", filename.basename()) 
186              try: 
187                  if filename.endswith(".gz"): 
188                      infile = gzip.open(filename, 'r') 
189                  else: 
190                      infile = open(filename, 'r') 
191                  numadded = self.add_articles(Article.parse_medline_xml(infile), dbenv) 
192              finally: 
193                  infile.close() 
194              logging.debug("Added %d articles", numadded) 
195              tracker.add(filename) 
196              tracker.dump() 
197              logging.info("Completed file %d out of %d (%s)",  
198                       idx+1, len(toprocess), filename.name) 
199          dbenv.close()

200
```

  


| Trees | Indices | Help | | MScanner | | --- | |
| --- | --- | --- | --- | --- |

|  |  |
| --- | --- |
| Generated by Epydoc 3.0beta1 on Fri Nov 23 09:13:22 2007 | http://epydoc.sourceforge.net |
