# Supplementary material for: MScanner: a classifier for retrieving Medline citations
Source: BMC Bioinformatics. 2008 Feb 19;9:108. doi: 10.1186/1471-2105-9-108 (PMC2263023; doi:10.1186/1471-2105-9-108)
Supplement: Additional file 3 — Source code for MScanner. mscanner-20071123.zip is a ZIP archive containing the Python 2.5 source code for MScanner, licensed under the GNU General Public License. It also contains API documentation in HTML format. Updated versions will be made available at . [file 1471-2105-9-108-S3.zip › mscanner/help/api/mscanner.medline.MedlineCache.MedlineCache-class.html]

xml version="1.0" encoding="ascii"?


mscanner.medline.MedlineCache.MedlineCache


| Trees | Indices | Help | | MScanner | | --- | |
| --- | --- | --- | --- | --- |

|  |  |  |  |
| --- | --- | --- | --- |
| Package mscanner :: Package medline :: Module MedlineCache :: Class MedlineCache | |  | | --- | | [hide private] | | [frames] | no frames] | |

# Class MedlineCache

source code  
  
Class for updating the Article DB, FeatureMapping, FeatureDatabase,
FeatureStream, PMID list, and FileTracker.  
  


|  |  |  |  |
| --- | --- | --- | --- |
| |  |  | | --- | --- | | Instance Methods | [hide private] | | |
|  | |  |  | | --- | --- | | \_\_init\_\_(self, featmap, db\_env\_home, article\_db, feature\_db, feature\_stream, article\_list, processed\_path, narticles\_path, use\_transactions=True)  Constructor parameters set corresponding instance variables. | source code | |
|  | |  |  | | --- | --- | | create\_dbenv(self)  Create a Berkeley DB environment for transactions | source code | |
|  | |  |  | | --- | --- | | \_article\_features(self, article)  Given an article object, return its feature vector, using featmap to create new features as necessary | source code | |
|  | |  |  | | --- | --- | | add\_articles(self, articles, dbenv)  Store Articles and feature lists in the databases | source code | |
|  | |  |  | | --- | --- | | add\_directory(self, medlinedir, save\_delay=5)  Adds articles from XML files to MScanner's databases | source code | |


|  |  |  |  |
| --- | --- | --- | --- |
| |  |  | | --- | --- | | Instance Variables | [hide private] | | |
|  | article\_db  Path to article database |
|  | article\_list  Path to list of article PMIDs |
|  | db\_env\_home  Path to DB home directory |
|  | featmap  A FeatureMapping object for mapping string features to IDs |
|  | feature\_db  Path to feature database |
|  | feature\_stream  Path to feature stream file |
|  | narticles\_path  Path to file containing the total number of PMIDs |
|  | processed\_path  Path to list of processed files |
|  | use\_transactions  If false, disable transaction engine |


|  |  |  |  |
| --- | --- | --- | --- |
| |  |  | | --- | --- | | Method Details | [hide private] | | |

|  |  |  |
| --- | --- | --- |
| |  |  | | --- | --- | | create\_dbenv(self) | source code |  Create a Berkeley DB environment for transactions Returns:  DBEnv instance |

|  |  |  |
| --- | --- | --- |
| |  |  | | --- | --- | | add\_articles(self, articles, dbenv) | source code |   Store Articles and feature lists in the databases Databases are opened and closed inside each call, so that the user can Ctrl-C during the timed delay between files without corrupting the database. Using transactions has too much overhead in time, and space used by the log files. Parameters:  - **`articles`** - Iterator over Article objects - **`dbenv`** - Database environment to use |

|  |  |  |
| --- | --- | --- |
| |  |  | | --- | --- | | add\_directory(self, medlinedir, save\_delay=5) | source code |  Adds articles from XML files to MScanner's databases Parameters:  - **`medlinedir`** - Path to a directory containing .xml.gz files - **`save_delay`** - Pause this many seconds between calls to add\_articles |

  


| Trees | Indices | Help | | MScanner | | --- | |
| --- | --- | --- | --- | --- |

|  |  |
| --- | --- |
| Generated by Epydoc 3.0beta1 on Fri Nov 23 09:13:22 2007 | http://epydoc.sourceforge.net |
