# Supplementary material for: MScanner: a classifier for retrieving Medline citations
Source: BMC Bioinformatics. 2008 Feb 19;9:108. doi: 10.1186/1471-2105-9-108 (PMC2263023; doi:10.1186/1471-2105-9-108)
Supplement: Additional file 3 — Source code for MScanner. mscanner-20071123.zip is a ZIP archive containing the Python 2.5 source code for MScanner, licensed under the GNU General Public License. It also contains API documentation in HTML format. Updated versions will be made available at . [file 1471-2105-9-108-S3.zip › mscanner/help/api/mscanner.medline.Shelf-pysrc.html]

xml version="1.0" encoding="ascii"?


mscanner.medline.Shelf


| Trees | Indices | Help | | MScanner | | --- | |
| --- | --- | --- | --- | --- |

|  |  |  |  |
| --- | --- | --- | --- |
| Package mscanner :: Package medline :: Module Shelf | |  | | --- | | [hide private] | | [frames] | no frames] | |

# Source Code for Module mscanner.medline.Shelf

```
  1  """Persistent shelf backed by Berkeley DB""" 
  2   
  3  import bsddb 
  4  import cPickle 
  5  import os 
  6  import unittest 
  7  from path import path 
  8  from UserDict import DictMixin 
  9  import zlib 
 10   
 11   
 12  __copyright__ = "2007 Graham Poulter" 
 13  __author__ = "Graham Poulter <http://graham.poulter.googlepages.com>" 
 14  __license__ = """This program is free software: you can redistribute it and/or 
 15  modify it under the terms of the GNU General Public License as published by the 
 16  Free Software Foundation, either version 3 of the License, or (at your option) 
 17  any later version. 
 18   
 19  This program is distributed in the hope that it will be useful, but WITHOUT ANY 
 20  WARRANTY; without even the implied warranty of MERCHANTABILITY or FITNESS FOR A 
 21  PARTICULAR PURPOSE. See the GNU General Public License for more details. 
 22   
 23  You should have received a copy of the GNU General Public License along with 
 24  this program. If not, see <http://www.gnu.org/licenses/>.""" 
 25   
 26   


27 -def open(filename, flags='c', mode=0660, dbenv=None, txn=None, dbname=None, compress=True):


28      """Open a shelf with Berkeley DB backend 
 29   
 30      @param flags: One of 'r','rw','w','c','n'. Optionally specify flags such as 
 31      db.DB_CREATE, db.DB_TRUNCATE, db.DB_RDONLY, db.DB_AUTO_COMMIT.  
 32       
 33      @param dbenv: Provides a db.DBEnv environment 
 34       
 35      @param txn: Optional transaction for the opening operation. 
 36       
 37      @param dbname: Selects a sub-database from the file. 
 38       
 39      @param compress: If True, also gzip the pickles in the shelf. 
 40       
 41      @return: L{Shelf} using the opened database. 
 42      """ 
 43      if isinstance(flags, basestring): 
 44          if flags == 'r': 
 45              flags = bsddb.db.DB_RDONLY 
 46          elif flags == 'rw': 
 47              flags = 0 
 48          elif flags == 'w': 
 49              flags = bsddb.db.DB_CREATE 
 50          elif flags == 'c': 
 51              flags = bsddb.db.DB_CREATE 
 52          elif flags == 'n': 
 53              flags = bsddb.db.DB_TRUNCATE | db.DB_CREATE 
 54          else: 
 55              raise bsddb.db.DBError("Flag %s is not in 'r', 'rw', 'w', 'c' or 'n'"  % str(flags)) 
 56      database = bsddb.db.DB(dbenv) 
 57      database.open(filename, dbname, bsddb.db.DB_HASH, flags, mode, txn=txn) 
 58      return Shelf(database, txn, compress)

 59   


60 -class Shelf(DictMixin):


61      """A shelf built upon a bsddb DB object.""" 
 62   


63 -    def __init__(self, database, txn=None, do_compression=True):


64          """Initialise shelf with a db.DB object 
 65           
 66          @param database: Instance of a db database 
 67           
 68          @param txn: Optional transaction context for shelf operations 
 69   
 70          @param do_compression: If True, compress pickles with zlib. 
 71          """ 
 72          self.db = database 
 73          self.do_compression = do_compression 
 74          self.set_txn(txn) 
 75          if self.do_compression: 
 76              self.compress = zlib.compress 
 77              self.decompress = zlib.decompress 
 78          else: 
 79              self.compress = lambda x:x 
 80              self.decompress = lambda x:x

 81   


82 -    def set_txn(self, txn=None):


83          """Set the transaction to use for database operations""" 
 84          self.txn = txn

 85   


86 -    def close(self):


87          """Close the underlying database.  Shelf must not be used afterwards.""" 
 88          self.db.close()

 89   


90 -    def __del__(self):


91          self.db.close()

 92   


93 -    def __len__(self):


94          return self.db.stat()["ndata"]

 95   


96 -    def __getitem__(self, key):


97          v = self.db.get(key, txn=self.txn) 
 98          if v is None: raise KeyError("Key %s not in database" % repr(key)) 
 99          return cPickle.loads(self.decompress(v))

100   


101 -    def __setitem__(self, key, value):


102          self.db.put(key, self.compress(cPickle.dumps(value, protocol=2)), self.txn)

103   


104 -    def __delitem__(self, key):


105          self.db.delete(key, self.txn)

106   


107 -    def keys(self):


108          return self.db.keys(self.txn)

109   


110 -    def items(self):


111          return list(self.iteritems())

112   


113 -    def values(self):


114          return [ v for k,v in self.iteritems() ]

115   


116 -    def __contains__(self, key):


117          return self.db.has_key(key, self.txn)

118   


119 -    def iteritems(self):


120          cur = self.db.cursor(self.txn) 
121          rec = cur.first() 
122          while rec is not None: 
123              yield rec[0], cPickle.loads(self.decompress(rec[1])) 
124              rec = cur.next() 
125          cur.close()

126           


127 -    def __iter__(self):


128          cur = self.db.cursor(self.txn) 
129          rec = cur.first(dlen=0, doff=0) 
130          while rec is not None: 
131              yield rec[0] 
132              rec = cur.next(dlen=0, doff=0) 
133          cur.close()

134
```

  


| Trees | Indices | Help | | MScanner | | --- | |
| --- | --- | --- | --- | --- |

|  |  |
| --- | --- |
| Generated by Epydoc 3.0beta1 on Fri Nov 23 09:13:22 2007 | http://epydoc.sourceforge.net |
