# Supplementary material for: MScanner: a classifier for retrieving Medline citations
Source: BMC Bioinformatics. 2008 Feb 19;9:108. doi: 10.1186/1471-2105-9-108 (PMC2263023; doi:10.1186/1471-2105-9-108)
Supplement: Additional file 3 — Source code for MScanner. mscanner-20071123.zip is a ZIP archive containing the Python 2.5 source code for MScanner, licensed under the GNU General Public License. It also contains API documentation in HTML format. Updated versions will be made available at . [file 1471-2105-9-108-S3.zip › mscanner/help/api/mscanner.medline.Shelf.Shelf-class.html]

xml version="1.0" encoding="ascii"?


mscanner.medline.Shelf.Shelf


| Trees | Indices | Help | | MScanner | | --- | |
| --- | --- | --- | --- | --- |

|  |  |  |  |
| --- | --- | --- | --- |
| Package mscanner :: Package medline :: Module Shelf :: Class Shelf | |  | | --- | | [hide private] | | [frames] | no frames] | |

# Class Shelf

source code  
  

```
UserDict.DictMixin --+
                     |
                    Shelf
```

---

A shelf built upon a bsddb DB object.  
  


|  |  |  |  |
| --- | --- | --- | --- |
| |  |  | | --- | --- | | Instance Methods | [hide private] | | |
|  | |  |  | | --- | --- | | \_\_init\_\_(self, database, txn=None, do\_compression=True)  Initialise shelf with a db.DB object | source code | |
|  | |  |  | | --- | --- | | set\_txn(self, txn=None)  Set the transaction to use for database operations | source code | |
|  | |  |  | | --- | --- | | close(self)  Close the underlying database. | source code | |
|  | |  |  | | --- | --- | | \_\_del\_\_(self) | source code | |
|  | |  |  | | --- | --- | | \_\_len\_\_(self) | source code | |
|  | |  |  | | --- | --- | | \_\_getitem\_\_(self, key) | source code | |
|  | |  |  | | --- | --- | | \_\_setitem\_\_(self, key, value) | source code | |
|  | |  |  | | --- | --- | | \_\_delitem\_\_(self, key) | source code | |
|  | |  |  | | --- | --- | | keys(self) | source code | |
|  | |  |  | | --- | --- | | items(self) | source code | |
|  | |  |  | | --- | --- | | values(self) | source code | |
|  | |  |  | | --- | --- | | \_\_contains\_\_(self, key) | source code | |
|  | |  |  | | --- | --- | | iteritems(self) | source code | |
|  | |  |  | | --- | --- | | \_\_iter\_\_(self) | source code | |
| **Inherited from `UserDict.DictMixin`**: `__cmp__`, `__repr__`, `clear`, `get`, `has_key`, `iterkeys`, `itervalues`, `pop`, `popitem`, `setdefault`, `update` | |


|  |  |  |  |
| --- | --- | --- | --- |
| |  |  | | --- | --- | | Method Details | [hide private] | | |

|  |  |  |
| --- | --- | --- |
| |  |  | | --- | --- | | \_\_init\_\_(self, database, txn=None, do\_compression=True)  *(Constructor)* | source code |  Initialise shelf with a db.DB object Parameters:  - **`database`** - Instance of a db database - **`txn`** - Optional transaction context for shelf operations - **`do_compression`** - If True, compress pickles with zlib. |

|  |  |  |
| --- | --- | --- |
| |  |  | | --- | --- | | close(self) | source code |  Close the underlying database. Shelf must not be used afterwards. |

|  |  |  |
| --- | --- | --- |
| |  |  | | --- | --- | | \_\_len\_\_(self)  *(Length operator)* | source code |   Overrides: UserDict.DictMixin.\_\_len\_\_ |

|  |  |  |
| --- | --- | --- |
| |  |  | | --- | --- | | items(self) | source code |   Overrides: UserDict.DictMixin.items |

|  |  |  |
| --- | --- | --- |
| |  |  | | --- | --- | | values(self) | source code |   Overrides: UserDict.DictMixin.values |

|  |  |  |
| --- | --- | --- |
| |  |  | | --- | --- | | \_\_contains\_\_(self, key)  *(In operator)* | source code |   Overrides: UserDict.DictMixin.\_\_contains\_\_ |

|  |  |  |
| --- | --- | --- |
| |  |  | | --- | --- | | iteritems(self) | source code |   Overrides: UserDict.DictMixin.iteritems |

|  |  |  |
| --- | --- | --- |
| |  |  | | --- | --- | | \_\_iter\_\_(self) | source code |   Overrides: UserDict.DictMixin.\_\_iter\_\_ |

  


| Trees | Indices | Help | | MScanner | | --- | |
| --- | --- | --- | --- | --- |

|  |  |
| --- | --- |
| Generated by Epydoc 3.0beta1 on Fri Nov 23 09:13:22 2007 | http://epydoc.sourceforge.net |
