# Supplementary material for: MScanner: a classifier for retrieving Medline citations
Source: BMC Bioinformatics. 2008 Feb 19;9:108. doi: 10.1186/1471-2105-9-108 (PMC2263023; doi:10.1186/1471-2105-9-108)
Supplement: Additional file 3 — Source code for MScanner. mscanner-20071123.zip is a ZIP archive containing the Python 2.5 source code for MScanner, licensed under the GNU General Public License. It also contains API documentation in HTML format. Updated versions will be made available at . [file 1471-2105-9-108-S3.zip › mscanner/help/api/mscanner.scripts-module.html]

xml version="1.0" encoding="ascii"?


mscanner.scripts


| Trees | Indices | Help | | MScanner | | --- | |
| --- | --- | --- | --- | --- |

|  |  |  |  |
| --- | --- | --- | --- |
| Package mscanner :: Package scripts | |  | | --- | | [hide private] | | [frames] | no frames] | |

# Package scripts

source code  
  
Executable scripts, some of which perform utility functions, and
others which carry out analyses for the MScanner paper results.  
  


|  |  |  |  |
| --- | --- | --- | --- |
| |  |  | | --- | --- | | Submodules | [hide private] | | |
| - **mscanner.scripts.dbhelper**: *Utility functions for working with files containing lists of   PubMed IDs, and Berkeley DBs containing pickled Articles, and for   regenerating the FeatureStream and article list.* - **mscanner.scripts.latexplots**: *Draws publication-quality plots for use in the paper* - **mscanner.scripts.query**: *Performs queries using datasets from the MScanner paper* - **mscanner.scripts.retrievaltest**: *Performs retrieval test analysis, where a subset of the input is   used to query, and the results are compared against the remainder   of the input.* - **mscanner.scripts.update**: *Update the MScanner database with new articles* - **mscanner.scripts.validate**: *Calculates cross validation results for the MScanner paper.* |

  


| Trees | Indices | Help | | MScanner | | --- | |
| --- | --- | --- | --- | --- |

|  |  |
| --- | --- |
| Generated by Epydoc 3.0beta1 on Fri Nov 23 09:13:20 2007 | http://epydoc.sourceforge.net |
