# Supplementary material for: MScanner: a classifier for retrieving Medline citations
Source: BMC Bioinformatics. 2008 Feb 19;9:108. doi: 10.1186/1471-2105-9-108 (PMC2263023; doi:10.1186/1471-2105-9-108)
Supplement: Additional file 3 — Source code for MScanner. mscanner-20071123.zip is a ZIP archive containing the Python 2.5 source code for MScanner, licensed under the GNU General Public License. It also contains API documentation in HTML format. Updated versions will be made available at . [file 1471-2105-9-108-S3.zip › mscanner/help/api/mscanner.scripts.cmpscores-pysrc.html]

xml version="1.0" encoding="ascii"?


mscanner.scripts.cmpscores


| Trees | Indices | Help | | MScanner | | --- | |
| --- | --- | --- | --- | --- |

|  |  |  |  |
| --- | --- | --- | --- |
| Package mscanner :: Package scripts :: Module cmpscores | |  | | --- | | [hide private] | | [frames] | no frames] | |

# Source Code for Module mscanner.scripts.cmpscores

```
 1  #!/usr/bin/env python 
 2   
 3  """Tests different scoring methods to see whether there is a 
 4  substantial difference in performance""" 
 5   
 6  __copyright__ = "2007 Graham Poulter" 
 7  __author__ = "Graham Poulter <http://graham.poulter.googlepages.com>" 
 8  __license__ = "GPL" 
 9   
10  import sys 
11   
12  from mscanner.configuration import rc, start_logger 
13  from mscanner.medline.Databases import Databases 
14  from mscanner.core.Validator import CrossValidator 
15   
16   
17  score_methods = [ 
18      "scores_offsetonly", 
19      "scores_withabsence", 
20      "scores_newpseudo", 
21      "scores_oldpseudo", 
22      "scores_rubin" ] 
23   
24   


25 -def do_comparisons():


26      env = Databases() 
27      #train_rel = rc.corpora / "pharmgkb-070205.txt" 
28      #train_irrel = rc.corpora / "medline07-100k.txt" 
29      train_rel = rc.corpora / "genedrug-small.txt" 
30      train_irrel = rc.articlelist 
31      for method in score_methods: 
32          rc.dataset = "pg07-" + method 
33          v = CrossValidation(rc.working / "cmpscores" / rc.dataset, env) 
34          v.validation(train_rel, train_irrel) 
35      env.close()

36       
37   
38  if __name__ == "__main__": 
39      start_logger() 
40      do_comparisons() 
41
```

  


| Trees | Indices | Help | | MScanner | | --- | |
| --- | --- | --- | --- | --- |

|  |  |
| --- | --- |
| Generated by Epydoc 3.0beta1 on Thu Nov 08 18:36:51 2007 | http://epydoc.sourceforge.net |
