# Supplementary material for: MScanner: a classifier for retrieving Medline citations
Source: BMC Bioinformatics. 2008 Feb 19;9:108. doi: 10.1186/1471-2105-9-108 (PMC2263023; doi:10.1186/1471-2105-9-108)
Supplement: Additional file 3 — Source code for MScanner. mscanner-20071123.zip is a ZIP archive containing the Python 2.5 source code for MScanner, licensed under the GNU General Public License. It also contains API documentation in HTML format. Updated versions will be made available at . [file 1471-2105-9-108-S3.zip › mscanner/help/api/mscanner.scripts.db2stream-module.html]

xml version="1.0" encoding="ascii"?


mscanner.scripts.db2stream


| Trees | Indices | Help | | MScanner | | --- | |
| --- | --- | --- | --- | --- |

|  |  |  |  |
| --- | --- | --- | --- |
| Package mscanner :: Package scripts :: Module db2stream | |  | | --- | | [hide private] | | [frames] | no frames] | |

# Module db2stream

source code  
  

Copy a FeatureDatabase to a FeatureStream

Usage:

```
   ./db2stream.py <dbfile> <outputfile>
```

This is to regenerate the FeatureStream (used by the cscore program to
perform fast queries), if it becomes corrupted but the FeatureDatabase is
ok.  
  


---

**Copyright:**
2007 Graham Poulter

**License:**
This source file is free software. It comes without any
warranty, to the extent permitted by applicable law. You can
redistribute it and/or modify it under the Do Whatever You Want
Public License. Terms and conditions:

0. Do Whatever You Want


|  |  |  |  |
| --- | --- | --- | --- |
| |  |  | | --- | --- | | Functions | [hide private] | | |
|  | |  |  | | --- | --- | | main(dbfile, streamfile) | source code | |

| Trees | Indices | Help | | MScanner | | --- | |
| --- | --- | --- | --- | --- |

|  |  |
| --- | --- |
| Generated by Epydoc 3.0beta1 on Thu Nov 08 18:36:48 2007 | http://epydoc.sourceforge.net |
