# Supplementary material for: MScanner: a classifier for retrieving Medline citations
Source: BMC Bioinformatics. 2008 Feb 19;9:108. doi: 10.1186/1471-2105-9-108 (PMC2263023; doi:10.1186/1471-2105-9-108)
Supplement: Additional file 3 — Source code for MScanner. mscanner-20071123.zip is a ZIP archive containing the Python 2.5 source code for MScanner, licensed under the GNU General Public License. It also contains API documentation in HTML format. Updated versions will be made available at . [file 1471-2105-9-108-S3.zip › mscanner/help/api/mscanner.scripts.db2stream-pysrc.html]

xml version="1.0" encoding="ascii"?


mscanner.scripts.db2stream


| Trees | Indices | Help | | MScanner | | --- | |
| --- | --- | --- | --- | --- |

|  |  |  |  |
| --- | --- | --- | --- |
| Package mscanner :: Package scripts :: Module db2stream | |  | | --- | | [hide private] | | [frames] | no frames] | |

# Source Code for Module mscanner.scripts.db2stream

```
 1  #!/usr/bin/env python 
 2   
 3  """Copy a FeatureDatabase to a FeatureStream 
 4   
 5  Usage:: 
 6      ./db2stream.py <dbfile> <outputfile> 
 7       
 8  This is to regenerate the FeatureStream (used by the cscore program to perform 
 9  fast queries), if it becomes corrupted but the FeatureDatabase is ok. 
10   
11  @copyright: 2007 Graham Poulter 
12   
13  @license: This source file is free software. It comes without any warranty, to 
14  the extent permitted by applicable law. You can redistribute it and/or modify 
15  it under the Do Whatever You Want Public License. Terms and conditions:  
16     0. Do Whatever You Want 
17  """ 
18   
19  import sys 
20   


21 -def main(dbfile, streamfile):


22      from mscanner.FeatureDatabase import FeatureDatabase, FeatureStream 
23      d = FeatureDatabase(dbfile, 'r') 
24      s = FeatureStream(open(streamfile, "wb")) 
25      for key, val in d.iteritems(): 
26          s.write(key, val) 
27      d.close() 
28      s.close()

29   
30  if __name__ == "__main__": 
31      main(sys.argv[1], sys.argv[2]) 
32
```

  


| Trees | Indices | Help | | MScanner | | --- | |
| --- | --- | --- | --- | --- |

|  |  |
| --- | --- |
| Generated by Epydoc 3.0beta1 on Thu Nov 08 18:36:51 2007 | http://epydoc.sourceforge.net |
