# Supplementary material for: MScanner: a classifier for retrieving Medline citations
Source: BMC Bioinformatics. 2008 Feb 19;9:108. doi: 10.1186/1471-2105-9-108 (PMC2263023; doi:10.1186/1471-2105-9-108)
Supplement: Additional file 3 — Source code for MScanner. mscanner-20071123.zip is a ZIP archive containing the Python 2.5 source code for MScanner, licensed under the GNU General Public License. It also contains API documentation in HTML format. Updated versions will be made available at . [file 1471-2105-9-108-S3.zip › mscanner/help/api/mscanner.scripts.dbhelper-module.html]

xml version="1.0" encoding="ascii"?


mscanner.scripts.dbhelper


| Trees | Indices | Help | | MScanner | | --- | |
| --- | --- | --- | --- | --- |

|  |  |  |  |
| --- | --- | --- | --- |
| Package mscanner :: Package scripts :: Module dbhelper | |  | | --- | | [hide private] | | [frames] | no frames] | |

# Module dbhelper

source code  
  

Utility functions for working with files containing lists of PubMed
IDs, and Berkeley DBs containing pickled Articles, and for regenerating
the FeatureStream and article list.

Usage:

```
   ./dbhelper.py function_name arg1 arg2 [...]
```

Please read the source code for the different functions available.  
  


---

**Copyright:**
2007 Graham Poulter

**License:**
This source file is free software. It comes without any
warranty, to the extent permitted by applicable law. You can
redistribute it and/or modify it under the Do Whatever You Want
Public License. Terms and conditions:

0. Do Whatever You Want


|  |  |  |  |
| --- | --- | --- | --- |
| |  |  | | --- | --- | | Functions | [hide private] | | |
|  | |  |  | | --- | --- | | listkeys(dbfile, outfile)  List the keys in a Berkeley database. | source code | |
|  | |  |  | | --- | --- | | regen\_stream(artdb, featdb, featstream)  Use an Article Shelf and FeatureDatabase to create a FeatureStream. | source code | |
|  | |  |  | | --- | --- | | regen\_article\_list(artdb, artlist)  Regenerate the article list from the article database | source code | |
|  | |  |  | | --- | --- | | pmid\_dates(artdb, infile, outfile)  Get dates for PMIDs listed in `infile`, writing PMID,date pairs to `outfile` in increasing order of date. | source code | |
|  | |  |  | | --- | --- | | select\_lines(infile, outfile, mindate=`'``00000000``'`, maxdate=`'``99999999``'`, N=`'``0``'`)  Select random PMIDs from `infile` and write them to `outfile`. | source code | |


|  |  |  |  |
| --- | --- | --- | --- |
| |  |  | | --- | --- | | Function Details | [hide private] | | |

|  |  |  |
| --- | --- | --- |
| |  |  | | --- | --- | | listkeys(dbfile, outfile) | source code |  List the keys in a Berkeley database. Parameters:  - **`dbfile`** - Path to Berkeley DB - **`outfile`** - Path to write database keys one per line |

|  |  |  |
| --- | --- | --- |
| |  |  | | --- | --- | | regen\_stream(artdb, featdb, featstream) | source code |  Use an Article Shelf and FeatureDatabase to create a FeatureStream. Parameters:  - **`artdb`** - Path to Shelf with Article objects - **`featdb`** - Path to FeatureDatabase - **`featstream`** - Path to write re-generated FeatureStream to |

|  |  |  |
| --- | --- | --- |
| |  |  | | --- | --- | | regen\_article\_list(artdb, artlist) | source code |  Regenerate the article list from the article database Parameters:  - **`artdb`** - Path to Shelf with Article objects - **`artlist`** - Path to write PubMed IDs and YYYYMMDD lines to. |

|  |  |  |
| --- | --- | --- |
| |  |  | | --- | --- | | pmid\_dates(artdb, infile, outfile) | source code |  Get dates for PMIDs listed in `infile`, writing PMID,date pairs to `outfile` in increasing order of date. Parameters:  - **`artdb`** - Path to Shelf with Article objects - **`infile`** - Path to PubMed IDs (PMID lines) - **`outfile`** - Path to write PMID YYYYMMDD lines to. |

|  |  |  |
| --- | --- | --- |
| |  |  | | --- | --- | | select\_lines(infile, outfile, mindate=`'``00000000``'`, maxdate=`'``99999999``'`, N=`'``0``'`) | source code |  Select random PMIDs from `infile` and write them to `outfile`. Parameters:  - **`infile`** - Read PMID YYYYMMDD lines from this path. - **`outfile`** - Write selected lines to this path. - **`N`** - (string) Number of lines to output (N="0" outputs   all matching) - **`mindate`**, **`maxdate`** - Only consider YYYYMMDD strings between these  Returns:  Selected lines as (PMID,YYYYMMDD) pairs of strings |

  


| Trees | Indices | Help | | MScanner | | --- | |
| --- | --- | --- | --- | --- |

|  |  |
| --- | --- |
| Generated by Epydoc 3.0beta1 on Fri Nov 23 09:13:20 2007 | http://epydoc.sourceforge.net |
