# Supplementary material for: MScanner: a classifier for retrieving Medline citations
Source: BMC Bioinformatics. 2008 Feb 19;9:108. doi: 10.1186/1471-2105-9-108 (PMC2263023; doi:10.1186/1471-2105-9-108)
Supplement: Additional file 3 — Source code for MScanner. mscanner-20071123.zip is a ZIP archive containing the Python 2.5 source code for MScanner, licensed under the GNU General Public License. It also contains API documentation in HTML format. Updated versions will be made available at . [file 1471-2105-9-108-S3.zip › mscanner/help/api/mscanner.scripts.dbhelper-pysrc.html]

xml version="1.0" encoding="ascii"?


mscanner.scripts.dbhelper


| Trees | Indices | Help | | MScanner | | --- | |
| --- | --- | --- | --- | --- |

|  |  |  |  |
| --- | --- | --- | --- |
| Package mscanner :: Package scripts :: Module dbhelper | |  | | --- | | [hide private] | | [frames] | no frames] | |

# Source Code for Module mscanner.scripts.dbhelper

```
  1  #!/usr/bin/env python 
  2   
  3  """Utility functions for working with files containing lists of PubMed IDs, 
  4  and Berkeley DBs containing pickled Articles, and for regenerating 
  5  the FeatureStream and article list. 
  6   
  7  Usage:: 
  8      ./dbhelper.py function_name arg1 arg2 [...] 
  9   
 10  Please read the source code for the different functions available. 
 11   
 12  @copyright: 2007 Graham Poulter 
 13   
 14  @license: This source file is free software. It comes without any warranty, to 
 15  the extent permitted by applicable law. You can redistribute it and/or modify 
 16  it under the Do Whatever You Want Public License. Terms and conditions:  
 17     0. Do Whatever You Want 
 18  """ 
 19   
 20  from __future__ import with_statement 
 21  from bsddb import db 
 22  from contextlib import closing 
 23  import random 
 24  import sys 
 25  from path import path 
 26   
 27  from mscanner.medline.FeatureDatabase import FeatureDatabase 
 28  from mscanner.medline.FeatureStream import FeatureStream, Date2Integer 
 29  from mscanner.medline import Shelf 
 30   
 31       


32 -def listkeys(dbfile, outfile):


33      """List the keys in a Berkeley database. 
 34       
 35      @param dbfile: Path to Berkeley DB 
 36      @param outfile: Path to write database keys one per line 
 37      """ 
 38      d = db.DB() 
 39      d.open(dbfile, None, db.DB_HASH, db.DB_RDONLY) 
 40      cur = d.cursor() 
 41      f = open(outfile, "w") 
 42      rec = cur.first(dlen=0, doff=0) 
 43      while rec is not None: 
 44          f.write(rec[0]+"\n") 
 45          rec = cur.next(dlen=0, doff=0) 
 46      cur.close() 
 47      d.close() 
 48      f.close()

 49   
 50   


51 -def regen_stream(artdb, featdb, featstream):


52      """Use an Article Shelf and FeatureDatabase to create a FeatureStream. 
 53      @param artdb: Path to Shelf with Article objects 
 54      @param featdb: Path to FeatureDatabase 
 55      @param featstream: Path to write re-generated FeatureStream to 
 56      """ 
 57      adb = Shelf.open(artdb, "r") 
 58      fd = FeatureDatabase(featdb, "r") 
 59      fs = FeatureStream(open(featstream, "wb")) 
 60      for idx, (pmid, art) in enumerate(adb.iteritems()): 
 61          if idx % 10000 == 0: 
 62              print "Completed %d" % idx 
 63          fs.write(pmid, art.date_completed, fd[pmid]) 
 64      fs.close() 
 65      fd.close() 
 66      adb.close()

 67   
 68   


69 -def regen_article_list(artdb, artlist):


70      """Regenerate the article list from the article database 
 71      @param artdb: Path to Shelf with Article objects 
 72      @param artlist: Path to write PubMed IDs and YYYYMMDD lines to.""" 
 73      adb = Shelf.open(artdb, "r") 
 74      f = open(artlist, "w") 
 75      for idx, (pmid, art) in enumerate(adb.iteritems()): 
 76          if idx % 10000 == 0: 
 77              print "Completed %d" % idx 
 78          f.write("%s %08d\n" % (pmid, Date2Integer(art.date_completed))) 
 79      f.close() 
 80      adb.close()

 81       
 82       


83 -def pmid_dates(artdb, infile, outfile):


84      """Get dates for PMIDs listed in L{infile}, writing PMID,date pairs 
 85      to L{outfile} in increasing order of date. 
 86      @param artdb: Path to Shelf with Article objects 
 87      @param infile: Path to PubMed IDs (PMID lines) 
 88      @param outfile: Path to write PMID YYYYMMDD lines to.""" 
 89      lines = [] 
 90      adb = Shelf.open(artdb, "r") 
 91      input = open(infile, "r") 
 92      for line in input: 
 93          if line.startswith("#"): continue 
 94          pmid = int(line.split()[0]) 
 95          try: 
 96              date = Date2Integer(adb[str(pmid)].date_completed) 
 97              lines.append((pmid,date)) 
 98          except KeyError, e: 
 99              print e.message 
100      adb.close() 
101      input.close() 
102      lines.sort(key=lambda x:x[1]) 
103      with open(outfile, "w") as f: 
104          for line in lines: 
105              f.write("%s %08d\n" % line)

106       
107   


108 -def select_lines(infile, outfile, mindate="00000000", maxdate="99999999", N="0"):


109      """Select random PMIDs from L{infile} and write them to L{outfile}. 
110      @param infile: Read PMID YYYYMMDD lines from this path. 
111      @param outfile: Write selected lines to this path. 
112      @param N: (string) Number of lines to output (N="0" outputs all matching) 
113      @param mindate, maxdate: Only consider YYYYMMDD strings between these 
114      @return: Selected lines as (PMID,YYYYMMDD) pairs of strings 
115      """ 
116      lines = [] 
117      N = int(N) 
118      input = open(infile, "r") 
119      for line in input: 
120          if line.startswith("#"): continue 
121          pmid, date = line.strip().split() 
122          if date >= mindate and date <= maxdate: 
123              lines.append((pmid,date)) 
124      input.close() 
125      if N > 0: 
126          lines = random.sample(lines, N) 
127      lines.sort(key=lambda x:x[1]) 
128      with open(outfile, "w") as f: 
129          for line in lines: 
130              f.write("%s %s\n" % line) 
131      return lines

132   
133   
134  if __name__ == "__main__": 
135      action = sys.argv[1] 
136      if action == "listkeys": 
137          listkeys(*sys.argv[2:4]) 
138      elif action == "regen_stream": 
139          regen_stream(sys.argv[2:5]) 
140      elif action == "regen_article_list": 
141          regen_article_list(sys.argv[2:4]) 
142      elif action == "pmid_dates": 
143          pmid_dates(*sys.argv[2:5]) 
144      elif action == "select_lines": 
145          select_lines(*sys.argv[2:7]) 
146
```

  


| Trees | Indices | Help | | MScanner | | --- | |
| --- | --- | --- | --- | --- |

|  |  |
| --- | --- |
| Generated by Epydoc 3.0beta1 on Fri Nov 23 09:13:25 2007 | http://epydoc.sourceforge.net |
