# Supplementary material for: MScanner: a classifier for retrieving Medline citations
Source: BMC Bioinformatics. 2008 Feb 19;9:108. doi: 10.1186/1471-2105-9-108 (PMC2263023; doi:10.1186/1471-2105-9-108)
Supplement: Additional file 3 — Source code for MScanner. mscanner-20071123.zip is a ZIP archive containing the Python 2.5 source code for MScanner, licensed under the GNU General Public License. It also contains API documentation in HTML format. Updated versions will be made available at . [file 1471-2105-9-108-S3.zip › mscanner/help/api/mscanner.scripts.latexplots-module.html]

xml version="1.0" encoding="ascii"?


mscanner.scripts.latexplots


| Trees | Indices | Help | | MScanner | | --- | |
| --- | --- | --- | --- | --- |

|  |  |  |  |
| --- | --- | --- | --- |
| Package mscanner :: Package scripts :: Module latexplots | |  | | --- | | [hide private] | | [frames] | no frames] | |

# Module latexplots

source code  
  

Draws publication-quality plots for use in the paper

The figures are:

```
   Figure 1. Four score density plots
   Figure 2. ROC curve overlay
   Figure 3. PR curve overlay
   Figure 4. P,R,F1,Fa curve for AIDSBio to demo optimisation
```

  
  


---

**Author:**
Graham Poulter <http://graham.poulter.googlepages.com>

**Copyright:**
2007 Graham Poulter

**License:**
This program is free software: you can redistribute it and/or
modify it under the terms of the GNU General Public License as
published by the
Free Software Foundation, either version 3 of the License, or (at
your option)
any later version.
This program is distributed in the hope that it will be useful, but
WITHOUT ANY
WARRANTY; without even the implied warranty of MERCHANTABILITY or
FITNESS FOR A
PARTICULAR PURPOSE. See the GNU General Public License for more
details.
You should have received a copy of the GNU General Public License
along with
this program. If not, see <http://www.gnu.org/licenses/>.


|  |  |  |  |
| --- | --- | --- | --- |
| |  |  | | --- | --- | | Functions | [hide private] | | |
|  | |  |  | | --- | --- | | smooth(x, y, xn=400)  Resample a curve (x,y) using interpolation. | source code | |
|  | |  |  | | --- | --- | | read\_featscores(indir, dataset)  Read feature scores for a dataset | source code | |
|  | |  |  | | --- | --- | | load\_stats(indir, dataset, title, alpha=0.5)  Read statistics based on score data | source code | |
|  | |  |  | | --- | --- | | gplot(x, y, ls, label, pos=0.6, usemarker=False)  Wraps plot to add a single marker marker instead of lots | source code | |
|  | |  |  | | --- | --- | | custom\_show(fname, doshow=False, type=`'``eps``'`)  Either shows an interactive plot, or writes to EPS followed by convertion to PDF (since matplotlib's PDF backend is buggy) | source code | |
|  | |  |  | | --- | --- | | plot\_score\_density(fname, statlist)  Plots four score density plots in a grid | source code | |
|  | |  |  | | --- | --- | | plot\_score\_histogram(fname, pscores, nscores)  Plot histograms for pos/neg scores, with line to mark threshold | source code | |
|  | |  |  | | --- | --- | | plot\_featscore\_histogram(fname, fscores)  Plot histogram for individual feature scores | source code | |
|  | |  |  | | --- | --- | | plot\_roc(fname, statlist)  Plots ROC curves overlayed | source code | |
|  | |  |  | | --- | --- | | plot\_precision(fname, statlist)  Plots PR curves overlayed | source code | |
|  | |  |  | | --- | --- | | do\_iedb(fname)  Plots retrieval test results for 20% of PharmGKB to see how MScanner and PubMed compare at retrieving the remaining 80%. | source code | |
|  | |  |  | | --- | --- | | do\_publication()  Draws figures for the BMC paper: including densities, ROC curve, PR curve, and PRF curve. | source code | |
|  | |  |  | | --- | --- | | do\_testplots()  Tests the plot functions using some old smaller datasets | source code | |
|  | |  |  | | --- | --- | | do\_subdirplots(subdirs)  Plots selected graphs for the datasets passed as parameters | source code | |


|  |  |  |  |
| --- | --- | --- | --- |
| |  |  | | --- | --- | | Variables | [hide private] | | |
|  | interactive = `False` |
|  | npoints = `400` |
|  | mscanner\_dir = `path(u'C:\\Documents and Settings\\Graham\\My D...` |
|  | source\_dir = `path(u'C:\\Documents and Settings\\Graham\\My Doc...` |
|  | outdir = `path(u'C:\\Documents and Settings\\Graham\\My Documen...` |
|  | Complex0 = `'F'` |
|  | Complex16 = `'F'` |
|  | Complex8 = `'F'` |
|  | Float0 = `'f'` |
|  | Float16 = `'f'` |
|  | Float8 = `'f'` |
|  | Int0 = `'b'` |
|  | divide\_safe = `<ufunc 'divide'>` |
|  | floor\_divide = `<ufunc 'floor_divide'>` |
|  | invert = `<ufunc 'invert'>` |
|  | left\_shift = `<ufunc 'left_shift'>` |
|  | remainder = `<ufunc 'remainder'>` |
|  | right\_shift = `<ufunc 'right_shift'>` |
|  | true\_divide = `<ufunc 'true_divide'>` |


|  |  |  |  |
| --- | --- | --- | --- |
| |  |  | | --- | --- | | Function Details | [hide private] | | |

|  |  |  |
| --- | --- | --- |
| |  |  | | --- | --- | | smooth(x, y, xn=400) | source code |  Resample a curve (x,y) using interpolation. xn is either a float with the new number of x value to evaluate the curve at, or an array of x values. |

|  |  |  |
| --- | --- | --- |
| |  |  | | --- | --- | | load\_stats(indir, dataset, title, alpha=0.5) | source code |  Read statistics based on score data Parameters:  - **`indir`** - Directory in which to find data sets - **`dataset`** - Subdirectory name for the particular data set - **`title`** - Name to put on the graphs (typically same as dataset) - **`alpha`** - What alpha to use when recalcing performance |

|  |  |  |
| --- | --- | --- |
| |  |  | | --- | --- | | plot\_score\_density(fname, statlist) | source code |  Plots four score density plots in a grid Parameters:  - **`statlist`** - A tuple of a four PerformanceVectors objects |

  


|  |  |  |  |
| --- | --- | --- | --- |
| |  |  | | --- | --- | | Variables Details | [hide private] | | |

|  |  |
| --- | --- |
| mscanner\_dir   Value:  |  | | --- | | ``` path(u'C:\\Documents and Settings\\Graham\\My Documents\\data\\MScanne r') ``` | |

|  |  |
| --- | --- |
| source\_dir   Value:  |  | | --- | | ``` path(u'C:\\Documents and Settings\\Graham\\My Documents\\data\\MScanne r\\output') ``` | |

|  |  |
| --- | --- |
| outdir   Value:  |  | | --- | | ``` path(u'C:\\Documents and Settings\\Graham\\My Documents\\temporary') ``` | |

  


| Trees | Indices | Help | | MScanner | | --- | |
| --- | --- | --- | --- | --- |

|  |  |
| --- | --- |
| Generated by Epydoc 3.0beta1 on Fri Nov 23 09:13:20 2007 | http://epydoc.sourceforge.net |
