# Supplementary material for: MScanner: a classifier for retrieving Medline citations
Source: BMC Bioinformatics. 2008 Feb 19;9:108. doi: 10.1186/1471-2105-9-108 (PMC2263023; doi:10.1186/1471-2105-9-108)
Supplement: Additional file 3 — Source code for MScanner. mscanner-20071123.zip is a ZIP archive containing the Python 2.5 source code for MScanner, licensed under the GNU General Public License. It also contains API documentation in HTML format. Updated versions will be made available at . [file 1471-2105-9-108-S3.zip › mscanner/help/api/mscanner.scripts.latexplots-pysrc.html]

xml version="1.0" encoding="ascii"?


mscanner.scripts.latexplots


| Trees | Indices | Help | MScanner |
| --- | --- | --- | --- |

Package mscanner ::
Package scripts ::
Module latexplots

[hide private]

[frames] | no frames]

# Source Code for Module mscanner.scripts.latexplots

```
  1  #!/usr/bin/env python 
  2   
  3  """ 
  4  Draws publication-quality plots for use in the paper 
  5   
  6  The figures are:: 
  7      Figure 1. Four score density plots 
  8      Figure 2. ROC curve overlay 
  9      Figure 3. PR curve overlay 
 10      Figure 4. P,R,F1,Fa curve for AIDSBio to demo optimisation 
 11   
 12  """ 
 13   
 14  from __future__ import with_statement 
 15  from __future__ import division 
 16   
 17  import logging 
 18  from path import path 
 19  from pylab import * 
 20   
 21  from mscanner.configuration import rc as mrc 
 22  from mscanner.core import iofuncs 
 23  from mscanner.core.Plotter import Plotter, DensityPlotter 
 24  from mscanner.core.metrics import PerformanceVectors 
 25  from mscanner.scripts import retrievaltest 
 26   
 27   
 28  __copyright__ = "2007 Graham Poulter" 
 29  __author__ = "Graham Poulter <http://graham.poulter.googlepages.com>" 
 30  __license__ = """This program is free software: you can redistribute it and/or 
 31  modify it under the terms of the GNU General Public License as published by the 
 32  Free Software Foundation, either version 3 of the License, or (at your option) 
 33  any later version. 
 34   
 35  This program is distributed in the hope that it will be useful, but WITHOUT ANY 
 36  WARRANTY; without even the implied warranty of MERCHANTABILITY or FITNESS FOR A 
 37  PARTICULAR PURPOSE. See the GNU General Public License for more details. 
 38   
 39  You should have received a copy of the GNU General Public License along with 
 40  this program. If not, see <http://www.gnu.org/licenses/>.""" 
 41   
 42  interactive = False 
 43  npoints = 400 
 44  mscanner_dir = path(r"C:\Documents and Settings\Graham\My Documents\data\MScanner") 
 45  source_dir = mscanner_dir / "output" 
 46  outdir = path(r"C:\Documents and Settings\Graham\My Documents\temporary") 
 47   
 48   
 49  rc("figure", figsize=(8,6), dpi=100) 
 50  rc("figure.subplot", hspace=0.3) 
 51  rc("font", family="serif", serif="cmr10", monospace="cmtt10") 
 52  rc("legend", axespad=0.04, labelsep=0.015, pad=0.2) 
 53  rc("lines", linewidth=1.0) 
 54  rc("savefig", dpi=100) 
 55  rc("xtick.major", pad=6.0) 
 56  rc("ytick.major", pad=6.0) 
 57   
 58   


59 -def smooth(x, y, xn=npoints):


60      """Resample a curve (x,y) using interpolation. xn is either a float with the 
 61      new number of x value to evaluate the curve at, or an array of x values. 
 62      """ 
 63      X = xn 
 64      if not isinstance(X, arraytype): 
 65          X = linspace(x[0], x[-1], xn) 
 66      import scipy.interpolate 
 67      interpolator = scipy.interpolate.interp1d(x, y, bounds_error=False) 
 68      Y = interpolator(X) 
 69      return X, Y

 70   
 71   


72 -def read_featscores(indir, dataset):


73      """Read feature scores for a dataset""" 
 74      with open(indir/dataset/mrc.report_term_scores, "r") as f: 
 75          f.readline() 
 76          return array([float(s.split(",",1)[0]) for s in f])

 77   
 78   


79 -def load_stats(indir, dataset, title, alpha=0.5):


80      """Read statistics based on score data 
 81       
 82      @param indir: Directory in which to find data sets 
 83      @param dataset: Subdirectory name for the particular data set 
 84      @param title: Name to put on the graphs (typically same as dataset) 
 85      @param alpha: What alpha to use when recalcing performance 
 86      """ 
 87      logging.info("Reading dataset %s", dataset) 
 88      if not (indir/dataset).isdir(): 
 89          raise ValueError("Could not directory %s" % (indir/dataset))  
 90      pscores = array([s[0] for s in iofuncs.read_scores( 
 91          indir/dataset/mrc.report_positives)]) 
 92      nscores = array([s[0] for s in iofuncs.read_scores( 
 93          indir/dataset/mrc.report_negatives)]) 
 94      stats = PerformanceVectors(pscores, nscores, alpha) 
 95      stats.title = title 
 96      stats.threshold, idx = stats.threshold_maximising(stats.FMa) 
 97      stats.tuned = stats.metrics_for(idx) 
 98      return stats

 99   
100   


101 -def gplot(x, y, ls, label, pos=0.6, usemarker=False):


102      """Wraps plot to add a single marker marker instead of lots""" 
103      if usemarker: 
104          i = int(pos*len(x)) 
105          plot(x,y,ls[0:2]) 
106          plot([x[i]],[y[i]],ls,label=label) 
107      else: 
108          plot(x,y,ls[0:2],label=label)

109   
110   


111 -def custom_show(fname, doshow=interactive, type="eps"):


112      """Either shows an interactive plot, or writes to EPS followed by 
113      convertion to PDF (since matplotlib's PDF backend is buggy)""" 
114      if interactive:  
115          show() 
116      try: 
117          fullname = outdir/fname + "." + type 
118          savefig(fullname) 
119          if type == "eps": 
120              from subprocess import call 
121              call(["epstopdf", fullname], shell=True) 
122              import os 
123              os.remove(fullname) 
124      finally: 
125          close()

126   
127   


128 -def plot_score_density(fname, statlist):


129      """Plots four score density plots in a grid 
130   
131      @param statlist: A tuple of a four PerformanceVectors objects 
132      """ 
133      logging.info("Plotting score densities to %s", fname) 
134      for idx, s in enumerate(statlist): 
135          px, py = DensityPlotter.gaussian_kernel_pdf(s.pscores) 
136          nx, ny = DensityPlotter.gaussian_kernel_pdf(s.nscores) 
137          subplot(2,2,idx+1) 
138          title(s.title) 
139          line_pos, = plot(px, py, color='red', label=r"Relevant") 
140          line_neg, = plot(nx, ny, color='blue', label=r"Irrelevant") 
141          line_threshold = axvline( 
142              s.uscores[s.bep_index], color='green', linewidth=1, label=r"Threshold") 
143          if idx == 0 or idx == 2: 
144              ylabel("Probability density") 
145          if idx == 2 or idx == 3: 
146              xlabel("Article score") 
147          if idx == 0: 
148              legend(loc="upper left") 
149      custom_show(fname)

150   
151   
152   


153 -def plot_score_histogram(fname, pscores, nscores):


154      """Plot histograms for pos/neg scores, with line to mark threshold""" 
155      logging.info("Plotting article score histogram to %s", fname) 
156      ##title("Article Score Histograms") 
157      xlabel("Article Score") 
158      ylabel("Article Density") 
159      p_n, p_bins, p_patches = hist(pscores, bins=Plotter.bincount(pscores), normed=True) 
160      n_n, n_bins, n_patches = hist(nscores, bins=Plotter.bincount(nscores), normed=True) 
161      setp(p_patches, 'facecolor', 'r', 'alpha', 0.50, 'linewidth', 0.0) 
162      setp(n_patches, 'facecolor', 'b', 'alpha', 0.50, 'linewidth', 0.0) 
163      #p_y = normpdf(p_bins, mean(pscores), std(pscores)) 
164      #n_y = normpdf(n_bins, mean(nscores), std(nscores)) 
165      #p_l = plot(p_bins, p_y, 'r--', label=r"$\rm{Relevants}$") 
166      #n_l = plot(n_bins, n_y, 'b--', label=r"$\rm{Irrelevant}$") 
167      custom_show(fname)

168   
169   


170 -def plot_featscore_histogram(fname, fscores):


171      """Plot histogram for individual feature scores""" 
172      logging.info("Plotting feature score histogram to %s", fname) 
173      ##title("Feature Score Histogram") 
174      xlabel("Feature Score") 
175      ylabel("Number of Features") 
176      fscores.sort() 
177      n, bins, patches = hist(fscores, bins=Plotter.bincount(fscores)) 
178      setp(patches, 'facecolor', 'r', 'linewidth', 0.0) 
179      custom_show(fname)

180   
181   


182 -def plot_roc(fname, statlist):


183      """Plots ROC curves overlayed""" 
184      logging.info("Plotting ROC grid to %s", fname) 
185      figure(figsize=(10,5)) 
186      formats = ["r-s", "b-D", "g-h", "c-"] 
187      values = [smooth(s.TPR[::-1], s.FPR[::-1]) for s in statlist] 
188      # Plot complete ROC curve 
189      subplot(121) 
190      ##title(r"ROC curves") 
191      ylabel(r"True Positive Rate (Recall)") 
192      xlabel(r"False Positive Rate (1-Specificity)") 
193      for (TPR, FPR), fmt  in zip(values, formats): 
194          plot(FPR, TPR, fmt[0:2]) 
195      axis([0.0, 1.0, 0.0, 1.0]) 
196      # Plot zoomed in ROC curve 
197      subplot(122) 
198      ##title(r"Magnified ROC") 
199      xlabel(r"False Positive Rate (1-Specificity)") 
200      for (TPR, FPR), fmt, s  in zip(values, formats, statlist): 
201          plot(FPR, TPR, fmt[0:2], label=s.title) 
202      legend(loc="lower right") 
203      axis([0.0, 0.01, 0, 1.0]) 
204      custom_show(fname)

205   
206   


207 -def plot_precision(fname, statlist):


208      """Plots PR curves overlayed""" 
209      logging.info("Plotting PR curve to %s", fname) 
210      ##title(r"Precision versus Recall") 
211      ylabel(r"Precision") 
212      xlabel(r"Recall") 
213      # Dotted line for break-even point 
214      plot([0.0, 1.0], [0.0, 1.0], "k:") 
215      # Pairs of TPR and PPV vectors for plotting 
216      formats = ["r-s", "b-D", "g-h", "c-o"] 
217      for s, fmt in zip(statlist, formats): 
218          TPR, PPV = smooth(s.TPR[::-1], s.PPV[::-1]) 
219          gplot(TPR, PPV, fmt, label=r"$\rm{"+s.title+r"}$", pos=0.5) 
220      # Place X marks at threshold 
221      #plot([s.tuned.TPR for s in statlist],  
222      #     [s.tuned.PPV for s in statlist], 
223      #     "kx", markeredgewidth=2) 
224      # Draw legends 
225      legend(loc=(0.5, 0.15)) 
226      axis([0.0, 1.0, 0.0, 1.0]) 
227      custom_show(fname)

228   
229   
230  #### FUNCTIONS THAT USE THE ABOVE #### 
231   


232 -def do_iedb(fname):


233      """Plots retrieval test results for 20% of PharmGKB to see how MScanner 
234      and PubMed compare at retrieving the remaining 80%. 
235      """ 
236      logging.info("Plotting Retrieval curve for PG07") 
237      indir = source_dir / "11.21 IEDB Query" 
238      iedb_precision = 0.306415 
239      lines = (indir/"perf_vs_rank.txt").lines() 
240      N = len(lines) 
241      precision = zeros(N, Float32) 
242      recall = zeros(N, Float32) 
243      ranks = zeros(N, Int32) 
244      for i, line in enumerate(lines): 
245          if i == 0: continue 
246          _rank, _TP, _rec, _prec = line.strip().split(", ") 
247          #print _rank, _TP, _rec, _prec 
248          ranks[i] = int(_rank) 
249          recall[i] = float(_rec) 
250          precision[i] = float(_prec) 
251      ylabel(r"Precision, Recall") 
252      xlabel(r"Rank") 
253      plot(ranks, recall, "r", label="MScanner recall") 
254      plot(ranks, precision, "b", label="MScanner precision") 
255      plot(ranks, ranks/N, 'c', label="IEDB query recall") 
256      plot(ranks, zeros(N)+iedb_precision, 'g', label="IEDB query precision") 
257      # Draw legends 
258      legend(loc=(0.05, 0.7)) 
259      axis([0.0, N, 0.0, 1.0]) 
260      custom_show(fname)

261   
262   


263 -def do_publication():


264      """Draws figures for the BMC paper: including densities, ROC curve, PR 
265      curve, and PRF curve. """ 
266      indir = source_dir / "11.21 Cross Validation" 
267      aidsbio = load_stats(indir, "aidsbio", "AIDSBio") 
268      radiology = load_stats(indir, "radiology", "Radiology") 
269      pg07 = load_stats(indir, "pg07", "PG07") 
270      control = load_stats(indir, "control", "Control") 
271      all = (aidsbio, radiology, pg07, control) 
272      #plot_score_density("fig3_density", all) 
273      plot_roc("fig4_roc", all)

274      #plot_precision("fig5_pr", all) 
275   
276   
277   


278 -def do_testplots():


279      """Tests the plot functions using some old smaller datasets""" 
280      global indir 
281      indir = source_dir / "Old Validation" / "070223 CV10 Daniel 2s" 
282      if not indir.isdir(): 
283          raise ValueError("Cannot find %s" % indir) 
284      pg04 = load_stats(indir, "pg04-vs-30k", "PG04") 
285      pg07 = load_stats(indir, "pg07-vs-30k", "PG07") 
286      plot_score_density("test_density", (pg04,pg07,pg07,pg04)) 
287      plot_roc("test_roc", (pg04,pg07)) 
288      plot_precision("test_pr", (pg04,pg07))

289   
290   
291   


292 -def do_subdirplots(subdirs):


293      """Plots selected graphs for the datasets passed as parameters""" 
294      statlist = [load_stats(source_dir/path(d), d, d) for d in subdirs] 
295      fscores = [read_featscores(source_dir/path(d), d) for d in subdirs] 
296      #plot_score_density("cus_density", stats) 
297      #plot_roc("cus_roc", statlist) 
298      #plot_precision("cus_pr", statlist) 
299      for stats, fscores in zip(statlist, fscores): 
300          #plot_fmeasure("cus_%s_prf" % stats.title, stats) 
301          plot_score_histogram( 
302              "cus_%s_arthist" % stats.title, stats.pscores, stats.nscores) 
303          plot_featscore_histogram( 
304              "cus_%s_feathist" % stats.title, fscores)

305   
306   
307  if __name__ == "__main__": 
308      iofuncs.start_logger(logfile=False) 
309      if len(sys.argv) != 2: 
310          print "Please provide a Python expression" 
311      else: 
312          eval(sys.argv[1]) 
313      logging.shutdown() 
314
```

  


| Trees | Indices | Help | MScanner |
| --- | --- | --- | --- |

|  |  |
| --- | --- |
| Generated by Epydoc 3.0beta1 on Fri Nov 23 09:13:25 2007 | http://epydoc.sourceforge.net |
