# Supplementary material for: MScanner: a classifier for retrieving Medline citations
Source: BMC Bioinformatics. 2008 Feb 19;9:108. doi: 10.1186/1471-2105-9-108 (PMC2263023; doi:10.1186/1471-2105-9-108)
Supplement: Additional file 3 — Source code for MScanner. mscanner-20071123.zip is a ZIP archive containing the Python 2.5 source code for MScanner, licensed under the GNU General Public License. It also contains API documentation in HTML format. Updated versions will be made available at . [file 1471-2105-9-108-S3.zip › mscanner/help/api/mscanner.scripts.listdbkeys-module.html]

xml version="1.0" encoding="ascii"?


mscanner.scripts.listdbkeys


| Trees | Indices | Help | MScanner |
| --- | --- | --- | --- |

Package mscanner ::
Package scripts ::
Module listdbkeys

[hide private]

[frames] | no frames]

# Module listdbkeys

source code  
  

Extract a list of PubMed IDs from a FeatureDatabase

Usage:

```
   ./listdbkeys.py <dbfile> <outputfile>
```

Iterate through the Berkeley DB named <dbfile>, and print its
keys one per line to <outputfile>.  
  


---

**Copyright:**
2007 Graham Poulter

**License:**
This source file is free software. It comes without any
warranty, to the extent permitted by applicable law. You can
redistribute it and/or modify it under the Do Whatever You Want
Public License. Terms and conditions:

0. Do Whatever You Want


|  |  |
| --- | --- |
| Functions | [hide private] |

|  |  |
| --- | --- |
| main(dbfile, outputfile) | source code |

| Trees | Indices | Help | MScanner |
| --- | --- | --- | --- |

|  |  |
| --- | --- |
| Generated by Epydoc 3.0beta1 on Thu Nov 08 18:36:48 2007 | http://epydoc.sourceforge.net |
