# Supplementary material for: MScanner: a classifier for retrieving Medline citations
Source: BMC Bioinformatics. 2008 Feb 19;9:108. doi: 10.1186/1471-2105-9-108 (PMC2263023; doi:10.1186/1471-2105-9-108)
Supplement: Additional file 3 — Source code for MScanner. mscanner-20071123.zip is a ZIP archive containing the Python 2.5 source code for MScanner, licensed under the GNU General Public License. It also contains API documentation in HTML format. Updated versions will be made available at . [file 1471-2105-9-108-S3.zip › mscanner/help/api/mscanner.scripts.query-module.html]

xml version="1.0" encoding="ascii"?


mscanner.scripts.query


| Trees | Indices | Help | MScanner |
| --- | --- | --- | --- |

Package mscanner ::
Package scripts ::
Module query

[hide private]

[frames] | no frames]

# Module query

source code  
  

Performs queries using datasets from the MScanner paper

Usage:

```
   python query.py <dataset> [ <dataset> ... ]
```

Example:

```
   python query.py pg07 radiology aids
```

  
  


---

**Author:**
Graham Poulter <http://graham.poulter.googlepages.com>

**Copyright:**
2007 Graham Poulter

**License:**
This program is free software: you can redistribute it and/or
modify it under the terms of the GNU General Public License as
published by the
Free Software Foundation, either version 3 of the License, or (at
your option)
any later version.
This program is distributed in the hope that it will be useful, but
WITHOUT ANY
WARRANTY; without even the implied warranty of MERCHANTABILITY or
FITNESS FOR A
PARTICULAR PURPOSE. See the GNU General Public License for more
details.
You should have received a copy of the GNU General Public License
along with
this program. If not, see <http://www.gnu.org/licenses/>.


|  |  |
| --- | --- |
| Functions | [hide private] |

|  |  |
| --- | --- |
| do\_query(\*datasets) | source code |


|  |  |
| --- | --- |
| Variables | [hide private] |

dataset\_map = `{'aidsbio': 'Paper/aidsbio.txt', 'gdsmall': 'Tes...`  
Mapping from dataset code to input file


|  |  |
| --- | --- |
| Variables Details | [hide private] |

### dataset\_map

Mapping from dataset code to input file

Value:
:   ```
    {'aidsbio': 'Paper/aidsbio.txt',
     'gdsmall': 'Test/gdsmall.txt',
     'invalid': 'Test/invalid.txt',
     'pg07': 'Paper/pg07_full.txt',
     'radiology': 'Paper/radiology.txt'}
    ```

  


| Trees | Indices | Help | MScanner |
| --- | --- | --- | --- |

|  |  |
| --- | --- |
| Generated by Epydoc 3.0beta1 on Fri Nov 23 09:13:20 2007 | http://epydoc.sourceforge.net |
