# Supplementary material for: MScanner: a classifier for retrieving Medline citations
Source: BMC Bioinformatics. 2008 Feb 19;9:108. doi: 10.1186/1471-2105-9-108 (PMC2263023; doi:10.1186/1471-2105-9-108)
Supplement: Additional file 3 — Source code for MScanner. mscanner-20071123.zip is a ZIP archive containing the Python 2.5 source code for MScanner, licensed under the GNU General Public License. It also contains API documentation in HTML format. Updated versions will be made available at . [file 1471-2105-9-108-S3.zip › mscanner/help/api/mscanner.scripts.query-pysrc.html]

xml version="1.0" encoding="ascii"?


mscanner.scripts.query


| Trees | Indices | Help | MScanner |
| --- | --- | --- | --- |

Package mscanner ::
Package scripts ::
Module query

[hide private]

[frames] | no frames]

# Source Code for Module mscanner.scripts.query

```
 1  #!/usr/bin/env python 
 2   
 3  """Performs queries using datasets from the MScanner paper 
 4   
 5  Usage:: 
 6   
 7      python query.py <dataset> [ <dataset> ... ] 
 8   
 9  Example:: 
10   
11      python query.py pg07 radiology aids 
12  """ 
13   
14  __copyright__ = "2007 Graham Poulter" 
15  __author__ = "Graham Poulter <http://graham.poulter.googlepages.com>" 
16  __license__ = """This program is free software: you can redistribute it and/or 
17  modify it under the terms of the GNU General Public License as published by the 
18  Free Software Foundation, either version 3 of the License, or (at your option) 
19  any later version. 
20   
21  This program is distributed in the hope that it will be useful, but WITHOUT ANY 
22  WARRANTY; without even the implied warranty of MERCHANTABILITY or FITNESS FOR A 
23  PARTICULAR PURPOSE. See the GNU General Public License for more details. 
24   
25  You should have received a copy of the GNU General Public License along with 
26  this program. If not, see <http://www.gnu.org/licenses/>.""" 
27   
28  import sys 
29   
30  from mscanner.configuration import rc 
31  from mscanner.core import iofuncs 
32  from mscanner.core.QueryManager import QueryManager 
33  from mscanner.medline.Databases import Databases 
34   
35   
36  dataset_map = { 
37      "aidsbio"     : "Paper/aidsbio.txt", 
38      "pg07"        : "Paper/pg07_full.txt", 
39      "radiology"   : "Paper/radiology.txt", 
40      "gdsmall"     : "Test/gdsmall.txt", 
41      "invalid"     : "Test/invalid.txt", 
42  } 
43  """Mapping from dataset code to input file""" 
44   
45   


46 -def do_query(*datasets):


47      env = Databases() 
48      for dataset in datasets: 
49          if dataset not in dataset_map: 
50              raise ValueError("Invalid Data Set %s" % dataset) 
51          QM = QueryManager(rc.working / "query" / dataset, dataset, limit=500, 
52                            env=env, threshold=0, prior=None) 
53          QM.query(rc.corpora / dataset_map[dataset]) 
54          QM.write_report() 
55      env.close()

56       
57   
58  if __name__ == "__main__": 
59      iofuncs.start_logger() 
60      do_query(*sys.argv[1:]) 
61
```

  


| Trees | Indices | Help | MScanner |
| --- | --- | --- | --- |

|  |  |
| --- | --- |
| Generated by Epydoc 3.0beta1 on Fri Nov 23 09:13:24 2007 | http://epydoc.sourceforge.net |
