# Supplementary material for: MScanner: a classifier for retrieving Medline citations
Source: BMC Bioinformatics. 2008 Feb 19;9:108. doi: 10.1186/1471-2105-9-108 (PMC2263023; doi:10.1186/1471-2105-9-108)
Supplement: Additional file 3 — Source code for MScanner. mscanner-20071123.zip is a ZIP archive containing the Python 2.5 source code for MScanner, licensed under the GNU General Public License. It also contains API documentation in HTML format. Updated versions will be made available at . [file 1471-2105-9-108-S3.zip › mscanner/help/api/mscanner.scripts.randlines-pysrc.html]

xml version="1.0" encoding="ascii"?


mscanner.scripts.randlines


| Trees | Indices | Help | MScanner |
| --- | --- | --- | --- |

Package mscanner ::
Package scripts ::
Module randlines

[hide private]

[frames] | no frames]

# Source Code for Module mscanner.scripts.randlines

```
 1  #!/usr/bin/env python 
 2   
 3  """Selects a random subset of lines from one file and writes them to another 
 4   
 5  Usage:: 
 6      random-pmids.py [input file] [output file] [number of lines] 
 7   
 8  @copyright: (C) 2007 Graham Poulter 
 9   
10  @license: This source file is free software. It comes without any warranty, to 
11  the extent permitted by applicable law. You can redistribute it and/or modify 
12  it under the Do Whatever You Want Public License. Terms and conditions:  
13     0. Do Whatever You Want 
14  """ 
15   
16  import random 
17  from path import path 
18  import sys 
19   


20 -def main(infile, outfile, nlines):


21      if not infile.isfile(): 
22          raise ValueError("%s does not exist" % infile) 
23      if outfile.isfile(): 
24          raise ValueError("%s already exists!" % outfile) 
25      outfile.write_lines(random.sample(infile.lines(), nlines))

26   
27  if __name__ == "__main__": 
28      main(path(sys.argv[1]), path(sys.argv[2]), int(sys.argv[3])) 
29
```

  


| Trees | Indices | Help | MScanner |
| --- | --- | --- | --- |

|  |  |
| --- | --- |
| Generated by Epydoc 3.0beta1 on Tue Nov 13 13:05:31 2007 | http://epydoc.sourceforge.net |
