# Supplementary material for: MScanner: a classifier for retrieving Medline citations
Source: BMC Bioinformatics. 2008 Feb 19;9:108. doi: 10.1186/1471-2105-9-108 (PMC2263023; doi:10.1186/1471-2105-9-108)
Supplement: Additional file 3 — Source code for MScanner. mscanner-20071123.zip is a ZIP archive containing the Python 2.5 source code for MScanner, licensed under the GNU General Public License. It also contains API documentation in HTML format. Updated versions will be made available at . [file 1471-2105-9-108-S3.zip › mscanner/help/api/mscanner.scripts.retrievaltest-module.html]

xml version="1.0" encoding="ascii"?


mscanner.scripts.retrievaltest


| Trees | Indices | Help | MScanner |
| --- | --- | --- | --- |

Package mscanner ::
Package scripts ::
Module retrievaltest

[hide private]

[frames] | no frames]

# Module retrievaltest

source code  
  
Performs retrieval test analysis, where a subset of the input is used
to query, and the results are compared against the remainder of the
input.  
  


---

**Author:**
Graham Poulter <http://graham.poulter.googlepages.com>

**Copyright:**
2007 Graham Poulter

**License:**
GPL


|  |  |
| --- | --- |
| Functions | [hide private] |

|  |  |
| --- | --- |
| truepos\_vs\_rank(results, test)  Evaluate true positives as function. | source code |

|  |  |
| --- | --- |
| plot\_rank\_performance(fname, textfname, tp\_vs\_rank, total\_relevant, total\_irrelevant)  Plot recall and precision versus rank. | source code |

|  |  |
| --- | --- |
| compare\_iedb\_query(test=False)  We compare MScanner retrieval to the complex PubMed query used to create the gold standard for the IEDB classifier. | source code |


|  |  |
| --- | --- |
| Function Details | [hide private] |

|  |  |
| --- | --- |
| truepos\_vs\_rank(results, test) | source code |

Evaluate true positives as function.

Parameters:
:   - **`results`** - Result PubMed IDs in decreasing order of score
    - **`test`** - Set of known relevant articles (to be counted as true
      positives)

Returns:
:   Vector of true positives at each rank (false positives = rank
    - true positives).

|  |  |
| --- | --- |
| plot\_rank\_performance(fname, textfname, tp\_vs\_rank, total\_relevant, total\_irrelevant) | source code |

Plot recall and precision versus rank.

Parameters:
:   - **`fname`** - File in which to draw the graph
    - **`textfname`** - File in which to write the text version of the graph
    - **`tp_vs_rank`** - Vector where the values are the number of true positives at
      rank (rank = vector index+1).
    - **`total_relevant`** - Total number of relevant articles to be found.
    - **`compare_irrelevant`** - The number of irrelevant articles retrieved by the query that
      was manually filtered to yield the `total_relevant`. We use this to calculate the
      precision of the PubMed query against which we are comparing.

  


| Trees | Indices | Help | MScanner |
| --- | --- | --- | --- |

|  |  |
| --- | --- |
| Generated by Epydoc 3.0beta1 on Fri Nov 23 09:13:20 2007 | http://epydoc.sourceforge.net |
