# Supplementary material for: MScanner: a classifier for retrieving Medline citations
Source: BMC Bioinformatics. 2008 Feb 19;9:108. doi: 10.1186/1471-2105-9-108 (PMC2263023; doi:10.1186/1471-2105-9-108)
Supplement: Additional file 3 — Source code for MScanner. mscanner-20071123.zip is a ZIP archive containing the Python 2.5 source code for MScanner, licensed under the GNU General Public License. It also contains API documentation in HTML format. Updated versions will be made available at . [file 1471-2105-9-108-S3.zip › mscanner/help/api/mscanner.scripts.retrievaltest-pysrc.html]

xml version="1.0" encoding="ascii"?


mscanner.scripts.retrievaltest


| Trees | Indices | Help | MScanner |
| --- | --- | --- | --- |

Package mscanner ::
Package scripts ::
Module retrievaltest

[hide private]

[frames] | no frames]

# Source Code for Module mscanner.scripts.retrievaltest

```
  1  #!/usr/bin/env python 
  2   
  3  """Performs retrieval test analysis, where a subset of the input is used to 
  4  query, and the results are compared against the remainder of the input.""" 
  5   
  6  from __future__ import division 
  7   
  8  __copyright__ = "2007 Graham Poulter" 
  9  __author__ = "Graham Poulter <http://graham.poulter.googlepages.com>" 
 10  __license__ = "GPL" 
 11   
 12  import itertools 
 13  import logging 
 14  import numpy as nx 
 15  from path import path 
 16  import pprint as pp 
 17  import random 
 18  import sys 
 19   
 20  from mscanner.configuration import rc 
 21  from mscanner.core import iofuncs 
 22  from mscanner.core.QueryManager import QueryManager 
 23  from mscanner.medline.Databases import Databases 
 24  from mscanner.medline.FeatureStream import Date2Integer 
 25   
 26   


27 -def truepos_vs_rank(results, test):


28      """Evaluate true positives as function. 
 29   
 30      @param results: Result PubMed IDs in decreasing order of score 
 31       
 32      @param test: Set of known relevant articles (to be counted as true positives) 
 33   
 34      @return: Vector of true positives at each rank (false 
 35      positives = rank - true positives).""" 
 36      assert isinstance(test, set) 
 37      TP_total = nx.zeros(len(results), nx.int32) 
 38      for idx, pmid in enumerate(results): 
 39          TP_total[idx] = TP_total[idx-1] if idx > 0 else 0 
 40          if pmid in test: 
 41              TP_total[idx] += 1 
 42      return TP_total

 43   
 44   


45 -def plot_rank_performance(fname, textfname, tp_vs_rank, total_relevant, total_irrelevant):


46      """Plot recall and precision versus rank. 
 47   
 48      @param fname: File in which to draw the graph 
 49       
 50      @param textfname: File in which to write the text version of the graph 
 51   
 52      @param tp_vs_rank: Vector where the values are the number of true positives 
 53      at rank (rank = vector index+1).  
 54       
 55      @param total_relevant: Total number of relevant articles to be found. 
 56       
 57      @param compare_irrelevant: The number of irrelevant articles retrieved by 
 58      the query that was manually filtered to yield the L{total_relevant}. We use 
 59      this to calculate the precision of the PubMed query against which we are 
 60      comparing. """ 
 61      logging.debug("Plotting Retrieval curve to %s", fname.basename()) 
 62      from Gnuplot import Gnuplot, Data 
 63      g = Gnuplot() 
 64      g.title("Query performance against positive data") 
 65      g.ylabel("Recall & Precision") 
 66      g.xlabel("Rank") 
 67      g("set terminal png") 
 68      g("set output '%s'" % fname) 
 69      ranks = nx.arange(1,len(tp_vs_rank)+1) 
 70      recall = tp_vs_rank / total_relevant 
 71      precision = tp_vs_rank / ranks 
 72      total_query = total_relevant + total_irrelevant 
 73      q_prec = total_relevant/total_query 
 74      q_recall = (q_prec * ranks) / total_relevant 
 75      iofuncs.write_lines(textfname, ["%d, %d, %.3f, %.3f" % x for x in  
 76          itertools.izip(itertools.count(), tp_vs_rank, recall, precision)], 
 77          "Rank, TP, Recall, Precision") 
 78      logging.info("There are %d relevant and %d irrelevant in Query results",  
 79                   total_relevant, total_irrelevant) 
 80      logging.info("At end, Query recall is 1.0 and precision is %f", q_prec) 
 81      logging.info("At end, MScanner recall is %f and precision is %f", 
 82                   recall[-1], precision[-1]) 
 83      g.plot(Data(ranks, recall, title="Recall", with="lines"), 
 84             Data(ranks, precision, title="Precision", with="lines"), 
 85             Data(ranks, q_recall, title="Query Recall", with="lines"), 
 86             Data([0,total_query], [q_prec,q_prec], title="Query Precision", with="lines"))

 87   
 88   
 89   


90 -def compare_iedb_query(test=False):


91      """We compare MScanner retrieval to the complex PubMed query 
 92      used to create the gold standard for the IEDB classifier.""" 
 93      dataset = "iedb_query" 
 94      outdir = rc.working / "query" / dataset 
 95      if not outdir.exists(): outdir.makedirs() 
 96      mindate, maxdate = 20040101, 20041231 
 97      t_mindate, t_maxdate = mindate, maxdate 
 98      trainpos_file = rc.corpora / "IEDB" / "iedb_pos_not2004.txt" 
 99      testpos_file = rc.corpora / "IEDB" / "iedb_pos_2004.txt" 
100      testneg_file = rc.corpora / "IEDB" / "iedb_neg_2004.txt" 
101      N_testnegs = len(testneg_file.lines()) 
102      testpos = iofunc.read_pmids(testpos_file) 
103      limit = len(testpos) + N_testnegs 
104      QM = QueryManager(outdir, dataset, limit,  
105                        mindate=mindate, maxdate=maxdate,  
106                        t_mindate=t_mindate, t_maxdate=t_maxdate) 
107      QM.query(trainpos_file) 
108      mscanner_results = [p for s,p in QM.results] 
109      truepos = truepos_vs_rank(mscanner_results, testpos) 
110      plot_rank_performance(outdir/"pr_rank.png",  
111                            outdir/"pr_rank.txt", 
112                            truepos, len(testpos), 
113                            compare_irrelevant=N_testnegs) 
114      QM.write_report(maxreport=1000)

115       
116   
117   
118  if __name__ == "__main__": 
119      iofuncs.start_logger() 
120      if len(sys.argv) != 2: 
121          print "Please provide a Python expression to execute" 
122      else: 
123          eval(sys.argv[1]) 
124      logging.shutdown() 
125
```

  


| Trees | Indices | Help | MScanner |
| --- | --- | --- | --- |

|  |  |
| --- | --- |
| Generated by Epydoc 3.0beta1 on Fri Nov 23 09:13:23 2007 | http://epydoc.sourceforge.net |
