# Supplementary material for: MScanner: a classifier for retrieving Medline citations
Source: BMC Bioinformatics. 2008 Feb 19;9:108. doi: 10.1186/1471-2105-9-108 (PMC2263023; doi:10.1186/1471-2105-9-108)
Supplement: Additional file 3 — Source code for MScanner. mscanner-20071123.zip is a ZIP archive containing the Python 2.5 source code for MScanner, licensed under the GNU General Public License. It also contains API documentation in HTML format. Updated versions will be made available at . [file 1471-2105-9-108-S3.zip › mscanner/help/api/mscanner.scripts.retrievaltest.RetrievalTest-class.html]

xml version="1.0" encoding="ascii"?


mscanner.scripts.retrievaltest.RetrievalTest


| Trees | Indices | Help | MScanner |
| --- | --- | --- | --- |

Package mscanner ::
Package scripts ::
Module retrievaltest ::
Class RetrievalTest

[hide private]

[frames] | no frames]

# Class RetrievalTest

source code  
  

```
core.QueryManager.QueryManager --+
                                 |
                                RetrievalTest
```

---

Performs retrieval-testing analysis, in which a subset of the data is
used to query, and the results are tested against the rest of the
data.  
  


|  |  |
| --- | --- |
| Instance Methods | [hide private] |

|  |  |
| --- | --- |
| retrieval\_query(self, input, test\_proportion)  Splits the input into training and testing, plots how many testing PMIDs the query returns when trained on the training PMIDs. | source code |

|  |  |
| --- | --- |
| report\_tp\_fp(self) | source code |

**Inherited from `core.QueryManager.QueryManager`**:
`__init__`,
`query`

**Inherited from `core.QueryManager.QueryManager`** (private):
`_load_input`,
`_load_results`,
`_make_feature_info`,
`_make_results`,
`_save_results`,
`_write_report`


|  |  |
| --- | --- |
| Class Variables | [hide private] |

test\_pmids\_file = `path(u'retrieval_test.txt')`  
Name of file with the gold standard against which the query is
tested

retrieval\_stats = `path(u'retrieval_stats.txt')`  
Name of file with cumulative count of retrieved test PMIDs

tpfp\_graph = `path(u'retrieval.png')`  
Name of retrieval vs rank graph

test\_proportion = `0.2`  
Proportion of data to use in retrieval test


|  |  |
| --- | --- |
| Instance Variables | [hide private] |

**Inherited from `core.QueryManager.QueryManager`**:
`featinfo`,
`inputs`,
`notfound_pmids`,
`pmids`,
`results`,
`timestamp`

Passed via constructor

**Inherited from `core.QueryManager.QueryManager`**:
`dataset`,
`limit`,
`maxdate`,
`mindate`,
`outdir`,
`threshold`


|  |  |
| --- | --- |
| Method Details | [hide private] |

|  |  |
| --- | --- |
| retrieval\_query(self, input, test\_proportion) | source code |

Splits the input into training and testing, plots how many testing
PMIDs the query returns when trained on the training PMIDs.

Returns:
:   Array with cumulative test PMIDs as function of rank

  


| Trees | Indices | Help | MScanner |
| --- | --- | --- | --- |

|  |  |
| --- | --- |
| Generated by Epydoc 3.0beta1 on Tue Nov 13 13:05:28 2007 | http://epydoc.sourceforge.net |
