# Supplementary material for: MScanner: a classifier for retrieving Medline citations
Source: BMC Bioinformatics. 2008 Feb 19;9:108. doi: 10.1186/1471-2105-9-108 (PMC2263023; doi:10.1186/1471-2105-9-108)
Supplement: Additional file 3 — Source code for MScanner. mscanner-20071123.zip is a ZIP archive containing the Python 2.5 source code for MScanner, licensed under the GNU General Public License. It also contains API documentation in HTML format. Updated versions will be made available at . [file 1471-2105-9-108-S3.zip › mscanner/help/api/mscanner.scripts.update-pysrc.html]

xml version="1.0" encoding="ascii"?


mscanner.scripts.update


| Trees | Indices | Help | MScanner |
| --- | --- | --- | --- |

Package mscanner ::
Package scripts ::
Module update

[hide private]

[frames] | no frames]

# Source Code for Module mscanner.scripts.update

```
 1  #!/usr/bin/env python 
 2   
 3  """Update the MScanner database with new articles 
 4   
 5  Usage:: 
 6      python update.py [somepickle] 
 7   
 8  If a path to a pickle is given, load Article objects from the Pickle into 
 9  the MScanner database. 
10   
11  With no arguments, look for new XML files in the Medline path and add their 
12  contents to the database.  
13  """ 
14   
15  from __future__ import with_statement 
16   
17  __copyright__ = "2007 Graham Poulter" 
18  __author__ = "Graham Poulter <http://graham.poulter.googlepages.com>" 
19  __license__ = """This program is free software: you can redistribute it and/or 
20  modify it under the terms of the GNU General Public License as published by the 
21  Free Software Foundation, either version 3 of the License, or (at your option) 
22  any later version. 
23   
24  This program is distributed in the hope that it will be useful, but WITHOUT ANY 
25  WARRANTY; without even the implied warranty of MERCHANTABILITY or FITNESS FOR A 
26  PARTICULAR PURPOSE. See the GNU General Public License for more details. 
27   
28  You should have received a copy of the GNU General Public License along with 
29  this program. If not, see <http://www.gnu.org/licenses/>.""" 
30   
31  import cPickle 
32  import logging 
33  import sys 
34   
35  from mscanner.medline.MedlineCache import MedlineCache 
36  from mscanner.medline.FeatureMapping import FeatureMapping 
37  from mscanner.configuration import rc 
38  from mscanner.core import iofuncs 
39   
40   


41 -def update_mscanner(pickle=None):


42      """Look for MScanner updates in the rc.medline direcotry 
43       
44      @param pickle: Path to a pickle of Article objects to be added instead. 
45      """ 
46      medcache = MedlineCache( 
47              FeatureMapping(rc.featuremap), 
48              rc.db_env_home, 
49              rc.articledb, 
50              rc.featuredb, 
51              rc.featurestream, 
52              rc.articlelist, 
53              rc.processed, 
54              rc.narticles, 
55              rc.use_transactions, 
56              ) 
57      # Load articles from a pickle 
58      if pickle is not None: 
59          logging.info("Updating MScanner from " + pickle ) 
60          with open(pickle , "rb") as f: 
61              articles = cPickle.load(f) 
62          dbenv = medcache.create_dbenv() 
63          medcache.add_articles(articles, dbenv) 
64          dbenv.close() 
65      # Parse articles from XML directory 
66      else: 
67          logging.info("Updating MScanner from " + rc.medline.relpath()) 
68          medcache.add_directory(rc.medline, rc.save_delay)

69   
70   
71  if __name__ == "__main__": 
72      iofuncs.start_logger(logfile=False) 
73      if len(sys.argv) == 1: 
74          update_mscanner() 
75      elif len(sys.argv) == 2: 
76          update_mscanner(sys.argv[1]) 
77      else: 
78          print __doc__ 
79      logging.shutdown() 
80
```

  


| Trees | Indices | Help | MScanner |
| --- | --- | --- | --- |

|  |  |
| --- | --- |
| Generated by Epydoc 3.0beta1 on Fri Nov 23 09:13:23 2007 | http://epydoc.sourceforge.net |
