# Supplementary material for: MScanner: a classifier for retrieving Medline citations
Source: BMC Bioinformatics. 2008 Feb 19;9:108. doi: 10.1186/1471-2105-9-108 (PMC2263023; doi:10.1186/1471-2105-9-108)
Supplement: Additional file 3 — Source code for MScanner. mscanner-20071123.zip is a ZIP archive containing the Python 2.5 source code for MScanner, licensed under the GNU General Public License. It also contains API documentation in HTML format. Updated versions will be made available at . [file 1471-2105-9-108-S3.zip › mscanner/help/api/mscanner.scripts.validate-pysrc.html]

xml version="1.0" encoding="ascii"?


mscanner.scripts.validate


| Trees | Indices | Help | MScanner |
| --- | --- | --- | --- |

Package mscanner ::
Package scripts ::
Module validate

[hide private]

[frames] | no frames]

# Source Code for Module mscanner.scripts.validate

```
  1  #!/usr/bin/env python 
  2   
  3  """Calculates cross validation results for the MScanner paper. 
  4   
  5  Choose the action by using a Python expression:: 
  6      python validate.py 'validate("aids-vs-500k")' 
  7  """ 
  8   
  9  from path import path 
 10  import sys 
 11   
 12  from mscanner.configuration import rc 
 13  from mscanner.core.ValidationManager import SplitValidation, CrossValidation 
 14  from mscanner.core import iofuncs 
 15  from mscanner.medline.Databases import Databases 
 16   
 17  __copyright__ = "2007 Graham Poulter" 
 18  __author__ = "Graham Poulter <http://graham.poulter.googlepages.com>" 
 19  __license__ = """This program is free software: you can redistribute it and/or 
 20  modify it under the terms of the GNU General Public License as published by the 
 21  Free Software Foundation, either version 3 of the License, or (at your option) 
 22  any later version. 
 23   
 24  This program is distributed in the hope that it will be useful, but WITHOUT ANY 
 25  WARRANTY; without even the implied warranty of MERCHANTABILITY or FITNESS FOR A 
 26  PARTICULAR PURPOSE. See the GNU General Public License for more details. 
 27   
 28  You should have received a copy of the GNU General Public License along with 
 29  this program. If not, see <http://www.gnu.org/licenses/>.""" 
 30   
 31   
 32  dataset_map = { 
 33  "aidsbio":       ("Paper/aidsbio.txt", "Paper/medline100k.txt"), 
 34  "pg07":          ("Paper/pg07_full.txt", "Paper/medline100k.txt"), 
 35  "radiology":     ("Paper/radiology.txt", "Paper/medline100k.txt"), 
 36  "control":       ("Paper/control.txt", "Paper/medline100k.txt"), 
 37  "gdsmall":       ("Test/gdsmall.txt", 30000), 
 38  } 
 39  """Map data set to pair of (positive,negative) paths for PubMed IDs.""" 
 40   
 41   


42 -def validate(*datasets):


43      """Perform cross-validation analysis on the main data sets""" 
 44      env = Databases() 
 45      for dataset in datasets: 
 46          if dataset not in dataset_map: 
 47              raise ValueError("Invalid Data Set %s" % dataset) 
 48          pos, neg = dataset_map[dataset] 
 49          if not isinstance(pos, path): 
 50              pos = rc.corpora / pos 
 51          if isinstance(neg, str): 
 52              neg = rc.corpora / neg 
 53          op = CrossValidation(rc.working / "valid" / dataset, dataset, env) 
 54          op.validation(pos, neg) 
 55          op.report_validation() 
 56          #op.report_predicted(1000, 10000, 16000000) 
 57      env.close()

 58   
 59   
 60       


61 -def compare_trec_genomics():


62      """Performs split-sample validation on the 2005 TREC Genomics track 
 63      categorisation subtasks (Allele, Expression, GO, Tumor).""" 
 64      env = Databases() 
 65      ntest = rc.corpora / "TREC" / "NEG_test.txt" 
 66      ntrain = rc.corpora / "TREC" / "NEG_train.txt" 
 67      for ds, Ur in [("A",17.0), ("E",64.0), ("G",11.0), ("T",231.0)]: 
 68          rc.utility_r = Ur 
 69          dataset = "TREC_%s" % ds 
 70          ptrain = rc.corpora / "TREC" / (ds + "train.txt") 
 71          ptest = rc.corpora / "TREC" / (ds + "test.txt") 
 72          op = SplitValidation(rc.working / "valid" / dataset, dataset, env) 
 73          op.validation(ptrain, ntrain, ptest, ntest) 
 74      env.close()

 75   
 76   
 77   


78 -def compare_iedb_valid():


79      """Performs cross validation using the IEDB gold standard data set""" 
 80      env = Databases() 
 81      basedir = rc.working / "valid" / "iedb" 
 82      if not basedir.exists(): basedir.mkdir() 
 83      rc.utility_r = None 
 84      #for fbase in "ac", "allergen", "er", "other": 
 85      for dataset in ("iedb",): 
 86          pos = rc.corpora / "IEDB" / ("%s_pos.txt" % fbase) 
 87          neg = rc.corpora / "IEDB" / ("%s_neg.txt" % fbase) 
 88          op = CrossValidation(basedir / dataset, dataset, env) 
 89          op.validation(pos, neg) 
 90          op.report_validation() 
 91      env.close()

 92       
 93       
 94  if __name__ == "__main__": 
 95      iofuncs.start_logger() 
 96      if len(sys.argv) != 2: 
 97          print "Please provide a Python expression to execute" 
 98      else: 
 99          eval(sys.argv[1]) 
100   
101   
102  ''' 
103  def compare_score_methods(): 
104      """Compare cross validation performance using different feature 
105      score calculation methods on the PharmGKB data set.""" 
106      score_methods = [ 
107          "scores_bayes", 
108          "scores_noabsence", 
109          "scores_rubin" ] 
110      env = Databases() 
111      #train_rel = rc.corpora / "pharmgkb-070205.txt" 
112      #train_irrel = rc.corpora / "medline07-100k.txt" 
113      train_rel = rc.corpora / "genedrug-small.txt" 
114      train_irrel = rc.articlelist 
115      for method in score_methods: 
116          dataset = "pg07-" + method 
117          op = CrossValidation(rc.working / "cmpscores" / dataset, dataset, env) 
118          op.validation(train_rel, train_irrel) 
119          op.report_validation() 
120      env.close() 
121       
122  def issn_features(): 
123      """Perform cross validation on for AIDSBio vs 100k, excluding ISSN features. 
124       
125      The results show that adding ISSN features produces better 
126      averaged precision in cross-validation.""" 
127      rc.exclude_types = ["issn"] 
128      pos, neg = dataset_map["aidsbio"] 
129      op = CrossValidation(rc.working / "valid" / "aidsbio-noissn",  
130                           "AIDSBio without ISSN features") 
131      op.validation(rc.corpora / pos, rc.corpora / neg) 
132      op.report_validation() 
133      op.env.close() 
134   
135  def control_modality(): 
136      """Cross validation of Random10K against Medline100K results 
137      in multiple modes in the article score distributions. 
138       
139      The distribution becomes unimodal about zero if we eliminate features that 
140      do not occur in positive citations. There are thousands of such features 
141      all of which have a negative feature scores of roughly the same value (-9). 
142      Random occurrences of 0,1,2 or 3 of those -9 features in test citations 
143      causes the score of that citation to be shifted by -9, -18, -27 from 
144      zero.""" 
145      rc.get_postmask = "mask_nonpositives" 
146      pos, neg = dataset_map["control"] 
147      op = CrossValidation(rc.working / "valid" / "modality", 
148                           "Removing Control corpus multi-modality") 
149      op.validation(rc.corpora / pos, rc.corpora / neg) 
150      op.report_validation() 
151      op.env.close() 
152  ''' 
153
```

  


| Trees | Indices | Help | MScanner |
| --- | --- | --- | --- |

|  |  |
| --- | --- |
| Generated by Epydoc 3.0beta1 on Fri Nov 23 09:13:25 2007 | http://epydoc.sourceforge.net |
