# Supplementary material for: MScanner: a classifier for retrieving Medline citations
Source: BMC Bioinformatics. 2008 Feb 19;9:108. doi: 10.1186/1471-2105-9-108 (PMC2263023; doi:10.1186/1471-2105-9-108)
Supplement: Additional file 3 — Source code for MScanner. mscanner-20071123.zip is a ZIP archive containing the Python 2.5 source code for MScanner, licensed under the GNU General Public License. It also contains API documentation in HTML format. Updated versions will be made available at . [file 1471-2105-9-108-S3.zip › mscanner/help/api/redirect.html]

Epydoc Redirect Page


### Epydoc Auto-redirect page

When javascript is enabled, this page will redirect URLs of
the form redirect.html#*dotted.name* to the
documentation for the object with the given fully-qualified
dotted name.
