# Supplementary material for: MScanner: a classifier for retrieving Medline citations
Source: BMC Bioinformatics. 2008 Feb 19;9:108. doi: 10.1186/1471-2105-9-108 (PMC2263023; doi:10.1186/1471-2105-9-108)
Supplement: Additional file 3 — Source code for MScanner. mscanner-20071123.zip is a ZIP archive containing the Python 2.5 source code for MScanner, licensed under the GNU General Public License. It also contains API documentation in HTML format. Updated versions will be made available at . [file 1471-2105-9-108-S3.zip › mscanner/help/api/toc-everything.html]

xml version="1.0" encoding="ascii"?


Everything


# Everything

---

## All Classes

Cheetah.CacheRegion.CacheItem  
 Cheetah.CacheRegion.CacheRegion  
 Cheetah.CacheStore.MemoryCacheStore  
 Cheetah.Compiler.AutoClassCompiler  
 Cheetah.Compiler.AutoMethodCompiler  
 Cheetah.Compiler.MethodCompiler  
 Cheetah.Compiler.ModuleCompiler  
 Cheetah.Parser.\_HighLevelParser  
 Cheetah.SettingsManager.ConfigParserCaseSensitive  
 Cheetah.Template.TemplatePreprocessor  
 Cheetah.Utils.WebInputMixin.NonNumericInputError  
 mscanner.core.FeatureScores.FeatureScores  
 mscanner.core.Plotter.DensityPlotter  
 mscanner.core.Plotter.Plotter  
 mscanner.core.QueryManager.QueryManager  
 mscanner.core.Storage.RCStorage  
 mscanner.core.Storage.Storage  
 mscanner.core.ValidationManager.CrossValidation  
 mscanner.core.ValidationManager.ValidationBase  
 mscanner.core.Validator.CrossValidator  
 mscanner.core.Validator.LeaveOutValidator  
 mscanner.core.iofuncs.FileTransaction  
 mscanner.core.metrics.PerformanceMetrics  
 mscanner.core.metrics.PerformanceRange  
 mscanner.core.metrics.PerformanceVectors  
 mscanner.core.metrics.PredictedMetrics  
 mscanner.fastscores.FeatureCounter.FeatureCounter  
 mscanner.fastscores.ScoreCalculator.ScoreCalculator  
 mscanner.htdocs.controller.FrontPage  
 mscanner.htdocs.forms.Button  
 mscanner.htdocs.forms.Checkbox  
 mscanner.htdocs.forms.Dropdown  
 mscanner.htdocs.forms.File  
 mscanner.htdocs.forms.Form  
 mscanner.htdocs.forms.Hidden  
 mscanner.htdocs.forms.Input  
 mscanner.htdocs.forms.Password  
 mscanner.htdocs.forms.Radio  
 mscanner.htdocs.forms.RegexValidator  
 mscanner.htdocs.forms.Textarea  
 mscanner.htdocs.forms.Textbox  
 mscanner.htdocs.forms.Validator  
 mscanner.htdocs.queue.QueueStatus  
 mscanner.htdocs.templates.contact.contact  
 mscanner.htdocs.templates.contact\_logic.ContactPage  
 mscanner.htdocs.templates.front.front  
 mscanner.htdocs.templates.output.output  
 mscanner.htdocs.templates.output\_logic.OutputPage  
 mscanner.htdocs.templates.page.page  
 mscanner.htdocs.templates.query.query  
 mscanner.htdocs.templates.query\_logic.QueryPage  
 mscanner.htdocs.templates.status.status  
 mscanner.htdocs.templates.status\_logic.StatusPage  
 mscanner.htdocs.testing.FormPage  
 mscanner.htdocs.testing.HelloPage  
 mscanner.medline.Article.Article  
 mscanner.medline.Databases.Databases  
 mscanner.medline.FeatureDatabase.FeatureDatabase  
 mscanner.medline.FeatureMapping.FeatureMapping  
 mscanner.medline.FeatureStream.FeatureStream  
 mscanner.medline.FileTracker.FileTracker  
 mscanner.medline.MedlineCache.MedlineCache  
 mscanner.medline.Shelf.Shelf  

## All Functions

mscanner.core.CitationTable.CitationTable  
 mscanner.core.CitationTable.write\_citations  
 mscanner.core.FeatureScores.FeatureCounts  
 mscanner.core.ValidationManager.SplitValidation  
 mscanner.core.iofuncs.close\_logfile  
 mscanner.core.iofuncs.no\_valid\_pmids\_page  
 mscanner.core.iofuncs.open\_logfile  
 mscanner.core.iofuncs.read\_pmids  
 mscanner.core.iofuncs.read\_pmids\_array  
 mscanner.core.iofuncs.read\_pmids\_careful  
 mscanner.core.iofuncs.read\_scores  
 mscanner.core.iofuncs.read\_scores\_array  
 mscanner.core.iofuncs.start\_logger  
 mscanner.core.iofuncs.write\_lines  
 mscanner.core.iofuncs.write\_pmids  
 mscanner.core.iofuncs.write\_scores  
 mscanner.htdocs.forms.attrget  
 mscanner.htdocs.forms.buttonpressed  
 mscanner.htdocs.forms.ischecked  
 mscanner.htdocs.queue.delete\_output  
 mscanner.htdocs.queue.logit  
 mscanner.htdocs.queue.mainloop  
 mscanner.htdocs.queue.parsebool  
 mscanner.htdocs.queue.populate\_test\_queue  
 mscanner.htdocs.queue.read\_descriptor  
 mscanner.htdocs.queue.write\_descriptor  
 mscanner.htdocs.templates.query\_logic.date\_is\_valid  
 mscanner.htdocs.templates.query\_logic.parse\_date  
 mscanner.htdocs.templates.query\_logic.parse\_pmids  
 mscanner.htdocs.templates.query\_logic.task\_does\_not\_exist  
 mscanner.htdocs.templates.query\_logic.task\_exists  
 mscanner.htdocs.testing.pformat  
 mscanner.medline.Databases.load\_articles  
 mscanner.medline.FeatureStream.Date2Integer  
 mscanner.medline.FeatureStream.Integer2Date  
 mscanner.medline.Shelf.open  
 mscanner.scripts.dbhelper.listkeys  
 mscanner.scripts.dbhelper.pmid\_dates  
 mscanner.scripts.dbhelper.regen\_article\_list  
 mscanner.scripts.dbhelper.regen\_stream  
 mscanner.scripts.dbhelper.select\_lines  
 mscanner.scripts.latexplots.custom\_show  
 mscanner.scripts.latexplots.do\_iedb  
 mscanner.scripts.latexplots.do\_publication  
 mscanner.scripts.latexplots.do\_subdirplots  
 mscanner.scripts.latexplots.do\_testplots  
 mscanner.scripts.latexplots.gplot  
 mscanner.scripts.latexplots.load\_stats  
 mscanner.scripts.latexplots.plot\_featscore\_histogram  
 mscanner.scripts.latexplots.plot\_precision  
 mscanner.scripts.latexplots.plot\_roc  
 mscanner.scripts.latexplots.plot\_score\_density  
 mscanner.scripts.latexplots.plot\_score\_histogram  
 mscanner.scripts.latexplots.read\_featscores  
 mscanner.scripts.latexplots.smooth  
 mscanner.scripts.query.do\_query  
 mscanner.scripts.retrievaltest.compare\_iedb\_query  
 mscanner.scripts.retrievaltest.plot\_rank\_performance  
 mscanner.scripts.retrievaltest.truepos\_vs\_rank  
 mscanner.scripts.update.update\_mscanner  
 mscanner.scripts.validate.compare\_iedb\_valid  
 mscanner.scripts.validate.compare\_trec\_genomics  
 mscanner.scripts.validate.validate  

## All Variables

mscanner.htdocs.controller.urls  
 mscanner.htdocs.forms.checkbox\_validator  
 mscanner.htdocs.forms.notnull  
 mscanner.htdocs.queue.descriptor\_keys  
 mscanner.htdocs.templates.contact.\_\_CHEETAH\_docstring\_\_  
 mscanner.htdocs.templates.contact.\_\_CHEETAH\_genTime\_\_  
 mscanner.htdocs.templates.contact.\_\_CHEETAH\_genTimestamp\_\_  
 mscanner.htdocs.templates.contact.\_\_CHEETAH\_srcLastModified\_\_  
 mscanner.htdocs.templates.contact.\_\_CHEETAH\_src\_\_  
 mscanner.htdocs.templates.contact.\_\_CHEETAH\_versionTuple\_\_  
 mscanner.htdocs.templates.contact.\_\_CHEETAH\_version\_\_  
 mscanner.htdocs.templates.contact\_logic.ContactForm  
 mscanner.htdocs.templates.front.\_\_CHEETAH\_docstring\_\_  
 mscanner.htdocs.templates.front.\_\_CHEETAH\_genTime\_\_  
 mscanner.htdocs.templates.front.\_\_CHEETAH\_genTimestamp\_\_  
 mscanner.htdocs.templates.front.\_\_CHEETAH\_srcLastModified\_\_  
 mscanner.htdocs.templates.front.\_\_CHEETAH\_src\_\_  
 mscanner.htdocs.templates.front.\_\_CHEETAH\_versionTuple\_\_  
 mscanner.htdocs.templates.front.\_\_CHEETAH\_version\_\_  
 mscanner.htdocs.templates.output.\_\_CHEETAH\_docstring\_\_  
 mscanner.htdocs.templates.output.\_\_CHEETAH\_genTime\_\_  
 mscanner.htdocs.templates.output.\_\_CHEETAH\_genTimestamp\_\_  
 mscanner.htdocs.templates.output.\_\_CHEETAH\_srcLastModified\_\_  
 mscanner.htdocs.templates.output.\_\_CHEETAH\_src\_\_  
 mscanner.htdocs.templates.output.\_\_CHEETAH\_versionTuple\_\_  
 mscanner.htdocs.templates.output.\_\_CHEETAH\_version\_\_  
 mscanner.htdocs.templates.output\_logic.OutputForm  
 mscanner.htdocs.templates.page.\_\_CHEETAH\_docstring\_\_  
 mscanner.htdocs.templates.page.\_\_CHEETAH\_genTime\_\_  
 mscanner.htdocs.templates.page.\_\_CHEETAH\_genTimestamp\_\_  
 mscanner.htdocs.templates.page.\_\_CHEETAH\_srcLastModified\_\_  
 mscanner.htdocs.templates.page.\_\_CHEETAH\_src\_\_  
 mscanner.htdocs.templates.page.\_\_CHEETAH\_versionTuple\_\_  
 mscanner.htdocs.templates.page.\_\_CHEETAH\_version\_\_  
 mscanner.htdocs.templates.query.\_\_CHEETAH\_docstring\_\_  
 mscanner.htdocs.templates.query.\_\_CHEETAH\_genTime\_\_  
 mscanner.htdocs.templates.query.\_\_CHEETAH\_genTimestamp\_\_  
 mscanner.htdocs.templates.query.\_\_CHEETAH\_srcLastModified\_\_  
 mscanner.htdocs.templates.query.\_\_CHEETAH\_src\_\_  
 mscanner.htdocs.templates.query.\_\_CHEETAH\_versionTuple\_\_  
 mscanner.htdocs.templates.query.\_\_CHEETAH\_version\_\_  
 mscanner.htdocs.templates.query\_logic.QueryForm  
 mscanner.htdocs.templates.query\_logic.dataset\_validator  
 mscanner.htdocs.templates.query\_logic.delcode\_validator  
 mscanner.htdocs.templates.query\_logic.form\_defaults  
 mscanner.htdocs.templates.status.\_\_CHEETAH\_docstring\_\_  
 mscanner.htdocs.templates.status.\_\_CHEETAH\_genTime\_\_  
 mscanner.htdocs.templates.status.\_\_CHEETAH\_genTimestamp\_\_  
 mscanner.htdocs.templates.status.\_\_CHEETAH\_srcLastModified\_\_  
 mscanner.htdocs.templates.status.\_\_CHEETAH\_src\_\_  
 mscanner.htdocs.templates.status.\_\_CHEETAH\_versionTuple\_\_  
 mscanner.htdocs.templates.status.\_\_CHEETAH\_version\_\_  
 mscanner.htdocs.templates.status\_logic.StatusForm  
 mscanner.htdocs.testing.TestForm  
 mscanner.htdocs.testing.form\_template  
 mscanner.htdocs.testing.urls  
 mscanner.scripts.latexplots.Complex0  
 mscanner.scripts.latexplots.Complex16  
 mscanner.scripts.latexplots.Complex8  
 mscanner.scripts.latexplots.Float0  
 mscanner.scripts.latexplots.Float16  
 mscanner.scripts.latexplots.Float8  
 mscanner.scripts.latexplots.Int0  
 mscanner.scripts.latexplots.divide\_safe  
 mscanner.scripts.latexplots.floor\_divide  
 mscanner.scripts.latexplots.interactive  
 mscanner.scripts.latexplots.invert  
 mscanner.scripts.latexplots.left\_shift  
 mscanner.scripts.latexplots.mscanner\_dir  
 mscanner.scripts.latexplots.npoints  
 mscanner.scripts.latexplots.outdir  
 mscanner.scripts.latexplots.remainder  
 mscanner.scripts.latexplots.right\_shift  
 mscanner.scripts.latexplots.source\_dir  
 mscanner.scripts.latexplots.true\_divide  
 mscanner.scripts.query.dataset\_map  
 mscanner.scripts.validate.dataset\_map  

---

[hide private]
