# Supplementary material for: MScanner: a classifier for retrieving Medline citations
Source: BMC Bioinformatics. 2008 Feb 19;9:108. doi: 10.1186/1471-2105-9-108 (PMC2263023; doi:10.1186/1471-2105-9-108)
Supplement: Additional file 3 — Source code for MScanner. mscanner-20071123.zip is a ZIP archive containing the Python 2.5 source code for MScanner, licensed under the GNU General Public License. It also contains API documentation in HTML format. Updated versions will be made available at . [file 1471-2105-9-108-S3.zip › mscanner/help/api/toc-mscanner.core.iofuncs-module.html]

xml version="1.0" encoding="ascii"?


iofuncs


# Module iofuncs

---

## Classes

FileTransaction  

## Functions

close\_logfile  
 no\_valid\_pmids\_page  
 open\_logfile  
 read\_pmids  
 read\_pmids\_array  
 read\_pmids\_careful  
 read\_scores  
 read\_scores\_array  
 start\_logger  
 write\_lines  
 write\_pmids  
 write\_scores  

---

[hide private]
