# Supplementary material for: MScanner: a classifier for retrieving Medline citations
Source: BMC Bioinformatics. 2008 Feb 19;9:108. doi: 10.1186/1471-2105-9-108 (PMC2263023; doi:10.1186/1471-2105-9-108)
Supplement: Additional file 3 — Source code for MScanner. mscanner-20071123.zip is a ZIP archive containing the Python 2.5 source code for MScanner, licensed under the GNU General Public License. It also contains API documentation in HTML format. Updated versions will be made available at . [file 1471-2105-9-108-S3.zip › mscanner/help/api/toc-mscanner.htdocs.forms-module.html]

xml version="1.0" encoding="ascii"?


forms


# Module forms

---

## Classes

Button  
 Checkbox  
 Dropdown  
 File  
 Form  
 Hidden  
 Input  
 Password  
 Radio  
 RegexValidator  
 Textarea  
 Textbox  
 Validator  

## Functions

attrget  
 buttonpressed  
 ischecked  

## Variables

checkbox\_validator  
 notnull  

---

[hide private]
