# Supplementary material for: MScanner: a classifier for retrieving Medline citations
Source: BMC Bioinformatics. 2008 Feb 19;9:108. doi: 10.1186/1471-2105-9-108 (PMC2263023; doi:10.1186/1471-2105-9-108)
Supplement: Additional file 3 — Source code for MScanner. mscanner-20071123.zip is a ZIP archive containing the Python 2.5 source code for MScanner, licensed under the GNU General Public License. It also contains API documentation in HTML format. Updated versions will be made available at . [file 1471-2105-9-108-S3.zip › mscanner/help/api/toc-mscanner.htdocs.queue-module.html]

xml version="1.0" encoding="ascii"?


queue


# Module queue

---

## Classes

QueueStatus  

## Functions

delete\_output  
 logit  
 mainloop  
 parsebool  
 populate\_test\_queue  
 read\_descriptor  
 write\_descriptor  

## Variables

descriptor\_keys  

---

[hide private]
