# Supplementary material for: MScanner: a classifier for retrieving Medline citations
Source: BMC Bioinformatics. 2008 Feb 19;9:108. doi: 10.1186/1471-2105-9-108 (PMC2263023; doi:10.1186/1471-2105-9-108)
Supplement: Additional file 3 — Source code for MScanner. mscanner-20071123.zip is a ZIP archive containing the Python 2.5 source code for MScanner, licensed under the GNU General Public License. It also contains API documentation in HTML format. Updated versions will be made available at . [file 1471-2105-9-108-S3.zip › mscanner/help/api/toc-mscanner.htdocs.templates.query_logic-module.html]

xml version="1.0" encoding="ascii"?


query\_logic


# Module query\_logic

---

## Classes

QueryPage  

## Functions

date\_is\_valid  
 parse\_date  
 parse\_pmids  
 task\_does\_not\_exist  
 task\_exists  

## Variables

QueryForm  
 dataset\_validator  
 delcode\_validator  
 form\_defaults  

---

[hide private]
