# Supplementary material for: MScanner: a classifier for retrieving Medline citations
Source: BMC Bioinformatics. 2008 Feb 19;9:108. doi: 10.1186/1471-2105-9-108 (PMC2263023; doi:10.1186/1471-2105-9-108)
Supplement: Additional file 3 — Source code for MScanner. mscanner-20071123.zip is a ZIP archive containing the Python 2.5 source code for MScanner, licensed under the GNU General Public License. It also contains API documentation in HTML format. Updated versions will be made available at . [file 1471-2105-9-108-S3.zip › mscanner/help/api/toc-mscanner.scripts.latexplots-module.html]

xml version="1.0" encoding="ascii"?


latexplots


# Module latexplots

---

## Functions

custom\_show  
 do\_iedb  
 do\_publication  
 do\_subdirplots  
 do\_testplots  
 gplot  
 load\_stats  
 plot\_featscore\_histogram  
 plot\_precision  
 plot\_roc  
 plot\_score\_density  
 plot\_score\_histogram  
 read\_featscores  
 smooth  

## Variables

Complex0  
 Complex16  
 Complex8  
 Float0  
 Float16  
 Float8  
 Int0  
 divide\_safe  
 floor\_divide  
 interactive  
 invert  
 left\_shift  
 mscanner\_dir  
 npoints  
 outdir  
 remainder  
 right\_shift  
 source\_dir  
 true\_divide  

---

[hide private]
