# Supplementary material for: MScanner: a classifier for retrieving Medline citations
Source: BMC Bioinformatics. 2008 Feb 19;9:108. doi: 10.1186/1471-2105-9-108 (PMC2263023; doi:10.1186/1471-2105-9-108)
Supplement: Additional file 3 — Source code for MScanner. mscanner-20071123.zip is a ZIP archive containing the Python 2.5 source code for MScanner, licensed under the GNU General Public License. It also contains API documentation in HTML format. Updated versions will be made available at . [file 1471-2105-9-108-S3.zip › mscanner/help/api/toc.html]

xml version="1.0" encoding="ascii"?


Table of Contents


# Table of Contents

---

Everything
  

## Modules

mscanner.core  
 mscanner.core.CitationTable  
 mscanner.core.FeatureScores  
 mscanner.core.Plotter  
 mscanner.core.QueryManager  
 mscanner.core.Storage  
 mscanner.core.ValidationManager  
 mscanner.core.Validator  
 mscanner.core.iofuncs  
 mscanner.core.metrics  
 mscanner.fastscores  
 mscanner.fastscores.FeatureCounter  
 mscanner.fastscores.ScoreCalculator  
 mscanner.htdocs  
 mscanner.htdocs.controller  
 mscanner.htdocs.forms  
 mscanner.htdocs.queue  
 mscanner.htdocs.templates  
 mscanner.htdocs.templates.contact  
 mscanner.htdocs.templates.contact\_logic  
 mscanner.htdocs.templates.front  
 mscanner.htdocs.templates.output  
 mscanner.htdocs.templates.output\_logic  
 mscanner.htdocs.templates.page  
 mscanner.htdocs.templates.query  
 mscanner.htdocs.templates.query\_logic  
 mscanner.htdocs.templates.status  
 mscanner.htdocs.templates.status\_logic  
 mscanner.htdocs.testing  
 mscanner.medline  
 mscanner.medline.Article  
 mscanner.medline.Databases  
 mscanner.medline.FeatureDatabase  
 mscanner.medline.FeatureMapping  
 mscanner.medline.FeatureStream  
 mscanner.medline.FileTracker  
 mscanner.medline.MedlineCache  
 mscanner.medline.Shelf  
 mscanner.scripts  
 mscanner.scripts.dbhelper  
 mscanner.scripts.latexplots  
 mscanner.scripts.query  
 mscanner.scripts.retrievaltest  
 mscanner.scripts.update  
 mscanner.scripts.validate  

---

[hide private]
