# Supplementary material for: MScanner: a classifier for retrieving Medline citations
Source: BMC Bioinformatics. 2008 Feb 19;9:108. doi: 10.1186/1471-2105-9-108 (PMC2263023; doi:10.1186/1471-2105-9-108)
Supplement: Additional file 3 — Source code for MScanner. mscanner-20071123.zip is a ZIP archive containing the Python 2.5 source code for MScanner, licensed under the GNU General Public License. It also contains API documentation in HTML format. Updated versions will be made available at . [file 1471-2105-9-108-S3.zip › mscanner/help/install.html]

xml version="1.0" encoding="utf-8" ?


MScanner: Retrieving Medline citations using examples


# MScanner: Retrieving Medline citations using examples

|  |  |
| --- | --- |
| Author: | Graham Poulter |
| Copyright: | 2007 Graham Poulter |

MScanner is a binary classifier operating on all of Medline. It learns
to predict relevant articles, given a set of relevant training
examples.

This source code is for running a local version of MScanner. Use
cases might include basing another classifier off the source code, or
tying MScanner results into a natural language processing pipeline.

# Installation

> - Unzip the source distribution into some directory INSTALL. On
>   a Unix-like system, INSTALL might be /home/joe/mscanner.
> - Create any directories required by the Directory Layout.
> - Add INSTALL/mscanner to the PYTHONPATH environment variable.
>   If done correctly, you should be able to import mscanner in a
>   Python shell.
> - Go to INSTALL/mscanner/fastscores and run GNU make to
>   compile the cscore.c program (Windows .exe and dll are
>   provided)
> - Install the Dependencies
> - Set up the Configuration
> - Place the input Input Data in the correct locations.
> - Run the unit tests by going into INSTALL/mscanner/tests and
>   running python \_\_init\_\_.py
> - Run INSTALL/mscanner/scripts/update.py to parse Medline and create
>   the databases in INSTALL/data/cache.
> - Run the Web Interface or use INSTALL/mscanner/scripts/query.py as
>   as starting point for constructing a custom query operation.

# Directory Layout

The structure of the INSTALL directory after extraction is as follows:

```
mscanner/doc -- INSTALL.txt
mscanner/doc/api -- Generated API documentation in HTML
mscanner/core -- Core MScanner package
mscanner/fastscores -- C utilities for fast classification
mscanner/htdocs -- Web interface package
mscanner/htdocs/static -- Static data available over the web
mscanner/htdocs/static/output -- Where web outputs are placed
mscanner/medline -- Modules for parsing and storing Medline and features
mscanner/scripts -- Executables (database utilities and customised analyses)
mscanner/tests -- Unit tests
```

Additionally create these directories to hold data:

```
data/cache -- MScanner databases
data/corpora -- Input files
data/medline -- The downloaded Medline distribution
data/queue -- Web interface task queue
```

# Input Data

MScanner requires the Medline Baseline distribution to generate the
database. Please contact the National Libraries of Medicine for a
license. MScanner looks for the .xml.gz files of the FTP distribution
of Baseline in INSTALL/data/medline (the rc.medline parameter)

Training the classifier requires PubMed IDs of relevant training
examples. See the MScanner publication for more information. Data
sets should be placed in INSTALL/data/corpora (set by the
rc.corpora parameter)

# Dependencies

Python
:   The Python programming language (version 2.5)

Numpy
:   For vector operations.

path
:   For path operations.

Cheetah
:   Templating engine for generating HTML outputs.

Gnuplot-py
:   For plotting cross validation output graphs. Gnuplot-py requires
    Gnuplot to be installed.

web.py
:   Python web application framework (for running the web interface).

# Web Interface

The web interface requires web.py to be installed. It can be run
under Apache as a CGI script, although web.py also has a built-in
webserver.

Start the queue process:

> Execute INSTALL/mscanner/htdocs/queue.py. This long-running
> process watches INSTALL/data/queue for descriptor files
> deposited by the web interface, performs the requested
> query/validation tasks, and deposits the output in the static HTML
> directory.

Start the internal web server:

> Execute INSTALL/mscanner/htdocs/controller.py. The web server
> will run on http://localhost:8080. The web interface takes user
> input, reports on status of MScanner, and places descriptor files in
> the queue directory on submission of a query.

Or: run the web interface under Apache:

> Configure Apache to use controller.py as a CGI script. Without
> URL rewriting you will have to access pages as
> http://localhost/controller.py/form.

## Re-generating API Documentation

API documentation is linked from the MScanner home page, and may
already be provided under INSTALL/mscanner/doc/api.

To re-generate the documentation, first install Epydoc , then navigate to
the the INSTALL/mscanner directory, and run:

```
epydoc.py --config=doc/epydoc.txt
```

To convert INSTALL/mscanner/doc/INSTALL.txt (which is written in the
reStructuredText format) into INSTALL.html, first install
Docutils, then navigate to INSTALL/mscanner/doc and run:

```
rst2html.py INSTALL.txt > INSTALL.html
```

# Configuration

Configuration is in INSTALL/mscanner/configuration.py, which has
MScanner resource parameters (and their descriptions). The rc
object holds all the global constants used by the MScanner library.

See the scripts in INSTALL/mscanner/scripts for examples of using
the MScanner API. Help on the API is in INSTALL/mscanner/help/api.
